# Global blood miRNA profiling unravels early signatures of immunogenicity of Ebola vaccine rVSVΔG-ZEBOV-GP

**DOI:** 10.1016/j.isci.2023.108574

**Published:** 2023-11-23

**Authors:** Eleonora Vianello, Josefine Persson, Björn Andersson, Suzanne van Veen, Thomaz Lüscher Dias, Francesco Santoro, Malin Östensson, Ogonna Obudulu, Christopher Agbajogu, Sara Torkzadeh, VSV-EBOVAC, Selidji Todagbe Agnandji, Rafi Ahmed, Jenna Anderson, Floriane Auderset, Philip Bejon, Luisa Borgianni, Jessica Brosnahan, Annalisa Ciabattini, Olivier Engler, Marielle C. Haks, Ali M. Harandi, Donald Gray Heppner, Alice Gerlini, Angela Huttner, Peter G. Kremsner, Donata Medaglini, Thomas Monath, Francis Ndungu, Patricia Njuguna, Tom H.M. Ottenhoff, David Pejoski, Mark Page, Gianni Pozzi, Francesco Santoro, Claire-Anne Siegrist, VSV-EBOPLUS Consortia, Selidji Todagbe Agnandji, Luisa Borgianni, Annalisa Ciabattini, Sheri Dubey, Micheal J. Eichberg, Olivier Engler, Patrícia Gonzalez-Dias, Paulin Ndong Essone, Ali M. Harandi, Alice Gerlini, Angela Huttner, Lumeka Kabwende, Peter Gottfried Kremsner, Donata Medaglini, Helder Nakaya, Sravya S. Nakka, Tom H.M. Ottenhoff, Mariëlle C. Haks, Josefine Persson, Gianni Pozzi, Sylvia Rothenberger, Francesco Santoro, Claire-Anne Siegrist, Suzanne van Veen, Eleonora Vianello, Helder I. Nakaya, Donata Medaglini, Claire-Anne Siegrist, Tom H.M. Ottenhoff, Ali M. Harandi

**Affiliations:** 1 Department of Infectious Diseases, Leiden University Medical Center, Leiden, the Netherlands; 2 Department of Microbiology and Immunology, Institute of Biomedicine, Sahlgrenska Academy, University of Gothenburg, Gothenburg, Sweden; 3 Bioinformatics Core Facility, Sahlgrenska Academy, University of Gothenburg, Gothenburg, Sweden; 4 Scientific Platform Pasteur-University of São Paulo, São Paulo, Brazil; 5 Department of Medical Biotechnologies, University of Siena, Italy; 6 Hospital Israelita Albert Einstein, São Paulo, Brazil; 7 Centre for Vaccinology, University Hospitals of Geneva and Faculty of Medicine, Geneva, Switzerland; 8 Vaccine Evaluation Center, BC Children’s Hospital Research Institute, University of British Columbia, Vancouver, Canada

**Keywords:** Health sciences, Molecular biology, Immunology, Virology

## Abstract

The vectored Ebola vaccine rVSVΔG-ZEBOV-GP elicits protection against Ebola Virus Disease (EVD). In a study of forty-eight healthy adult volunteers who received either the rVSVΔG-ZEBOV-GP vaccine or placebo, we profiled intracellular microRNAs (miRNAs) from whole blood cells (WB) and circulating miRNAs from serum-derived extracellular vesicles (EV) at baseline and longitudinally following vaccination. Further, we identified early miRNA signatures associated with ZEBOV-specific IgG antibody responses at baseline and up to one year post-vaccination, and pinpointed target mRNA transcripts and pathways correlated to miRNAs whose expression was altered after vaccination by using systems biology approaches.

Several miRNAs were differentially expressed (DE) and miRNA signatures predicted high or low IgG ZEBOV-specific antibody levels with high classification performance. The top miRNA discriminators were WB-miR-6810, EV-miR-7151-3p, and EV-miR-4426. An eight-miRNA antibody predictive signature was associated with immune-related target mRNAs and pathways.

These findings provide valuable insights into early blood biomarkers associated with rVSVΔG-ZEBOV-GP vaccine-induced IgG antibody responses.

## Highlights

• RVSV-ZEBOV-GP vaccination modulates human blood intracellular and circulating miRNome
• Baseline and early post-vaccination miRNAs correlate with IgG antibody responses
• Early post-vaccination miRNA signatures predict D28 and D360 IgG antibody levels
• An eight-miRNA signature correlates with immune-related target mRNAs and pathways

## Graphical abstract

**Figure:**
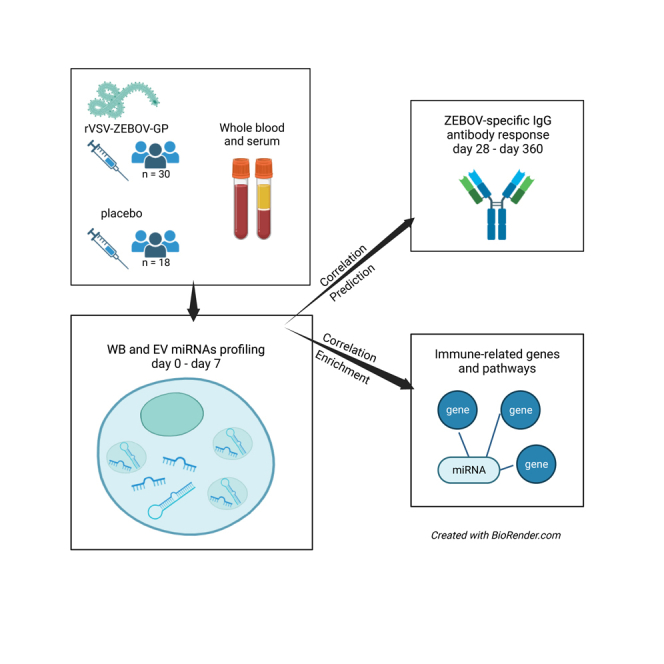

## Introduction

The Ebola virus is a zoonotic pathogen that can cause Ebola virus disease (EVD) in humans, characterized by acute fever illness, gastrointestinal symptoms, hemorrhage and multiple organ failure, with a high case-fatality rate (up to 90%).^1^ The EVD outbreak in West Africa, starting at the end of 2013, prompted the World Health Organization (WHO) to initiate fast-track phase 1/2 clinical trials of experimental vaccines targeting EVD.^2^ One of them was a recombinant vesicular stomatitis virus vector expressing the Zaire Ebola virus glycoprotein (rVSVΔG-ZEBOV-GP), recently approved by the European and the U.S. regulatory agencies under the name Ervebo. This vaccine was shown to be safe and immunogenic after a single dose, albeit with some transient reactogenicity,^3^^,^^4^ and highly efficacious in a ring vaccination trial conducted during the EVD outbreak in Guinea^5^^,^^6^ and during the latest outbreaks in the Democratic Republic of the Congo.^7^

Deciphering immunological and molecular signatures of the rVSVΔG-ZEBOV-GP vaccine is valuable as it is the only Ebola vaccine that has demonstrated clinical efficacy. Combining conventional serology and clinical data with data generated by omics technologies, such as transcriptomics,^8^^,^^9^ miRNomics and metabolomics, using systems biology approaches may lead to understandings that can inform future clinical vaccine trials as well as for innovations that can lead to novel vaccine strategies.^10^

MicroRNAs (miRNAs) are post-transcriptional regulators of gene expression. When binding to complementary sequences of mRNA, typically in the 3′ un-translated region, miRNA can cause degradation of mRNA and/or repress translation.^11^ A single mature miRNA can have profound effects on protein expression as shown for innate and adaptive immune responses.^12^^,^^13^^,^^14^^,^^15^^,^^16^ Blood miRNAs can be found in the intracellular and circulating compartments, both in free form and associated with extracellular vesicles (EV),^17^^,^^18^ and offer great potential as biomarkers owing to their stability in blood.^19^^,^^20^

Within the framework of the EU-financed consortium VSV-EBOPLUS, we gained access to whole blood (WB) PAXgene and serum samples from a subset of healthy adults that received the vaccine at a dose of 2 × 10^7^ pfu (the dose used in Ervebo) or placebo in a randomized, double-blinded, placebo-controlled, dose-finding clinical trial of rVSVΔG-ZEBOV-GP vaccine conducted at several U.S. sites.^21^ RNAs extracted from WB and serum-derived EV were subjected to a global sequencing analysis of miRNA transcripts followed by systems biology analyses.

We herein report miRNA profiles of WB and EV collected from healthy adults longitudinally for 7 days after a single vaccination with rVSVΔG-ZEBOV-GP. We also defined baseline and early blood miRNAs that correlate with the magnitude of ZEBOV- GP-specific IgG antibodies and even predict high or low antibody responses during the first-year post-vaccination. Finally, we assessed the immune-related mRNA targets of the miRNAs that predicted the IgG antibody response. These findings contribute to our understanding of potential early blood miRNA biomarkers associated with the development of IgG antibody responses in individuals vaccinated with the rVSVΔG-ZEBOV-GP vaccine.

## Results

### Global blood miRNA changes induced by rVSVΔG-ZEBOV-GP vaccination over time

Changes in the miRNome were profiled, in response to the rVSVΔG-ZEBOV-GP Ebola vaccine during the first week following rVSVΔG-ZEBOV-GP vaccination, in both WB and serum-isolated EV from a subset of adult volunteers, enrolled in a randomized, double-blind, controlled vaccine trial in the U.S.^21^ The first week (D1-D7) following vaccination was selected for the miRNA profiling based on our previous transcriptomic studies in which the most pronounced gene expression perturbations in blood of the cohort used in this study as well as three other cohorts were observed between D1 and D7 after rVSVΔG-ZEBOV-GP vaccination.^8^^,^^9^

**Figure 1:**
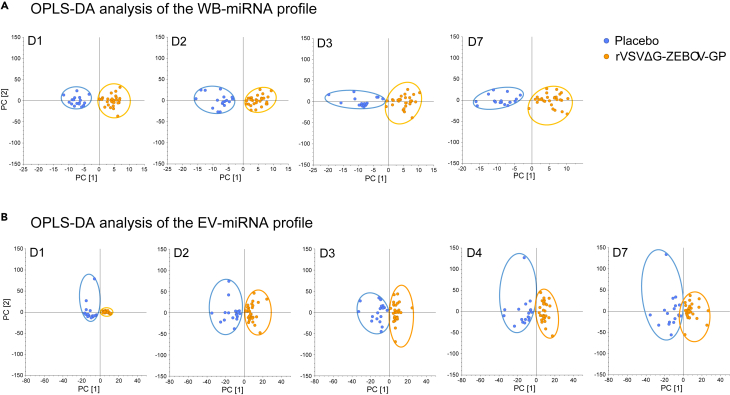
Data overview of the WB-miRNA and the EV-miRNA profile during the first week after rVSVΔG-ZEBOV-GP vaccination Blood samples were retrieved from individuals that received either the rVSVΔG-ZEBOV-GP Ebola vaccine or placebo and processed for miRNomic profiling. The normalized and baseline-adjusted Log2-transformed sequencing data were modeled in two-dimensional OPLS-DA plots, a statistical multivariate data analysis technique. The horizontal direction of the plots displays the inter-group variation, while the intra-group variation can be seen in the vertical direction of the plots. (A) Score plots of OPLS-DA analysis of the WB-miRNA profile identified (1 + 1+0) components at day (D) 1, D2, D3, and D7. The models explained 16–21% (R^2^X WB-miRNA) of the inter-group variance and 81–89% (R^2^Y WB-miRNA) of the of the intra-group variation. (B) Score plots of OPLS-DA analysis of the EV-miRNA profile identified (1 + 1+0) components at D1, D2, D3, D4, and D7. The models explained 9–36% (R^2^X EV-miRNA) of the inter-group variance and 85–92% (R^2^Y EV-miRNAs) of the of the intra-group variation.

Firstly, the normalized and baseline-adjusted Log2-transformed sequencing data were modeled in two-dimensional OPLS-DA plots, a supervised statistical multivariate data analysis technique, used for discriminant analysis (Figures 1 and S1). Adjustment of data to pre-vaccination (D0) values improved the separation between the vaccine group and the placebo (Figure S1), therefore baseline-adjusted data were used for the following analyses. The rVSVΔG-ZEBOV-GP group was distinguishably separated from the placebo group, and PC1 accounted for most of the inter-group variation at all time points (Figure 1). The models explained 16–21% (R^2^X WB-miRNA) and 9–36% (R^2^X EV-miRNA) of the variance in the data retrieved from WB (Figure 1A) and EV (Figure 1B), respectively. The intra-group variation (y axis) was low in all groups, but with a few deviating samples in the EV-placebos. The models explained 81–89% (R^2^Y WB-miRNA) (Figure 1A) and 85–92% (R^2^Y EV-miRNA) (Figure 1B) of the intra-group variance.

### Identification of differentially expressed miRNA transcripts induced following rVSVΔG-ZEBOV-GP vaccine

**Figure 2:**
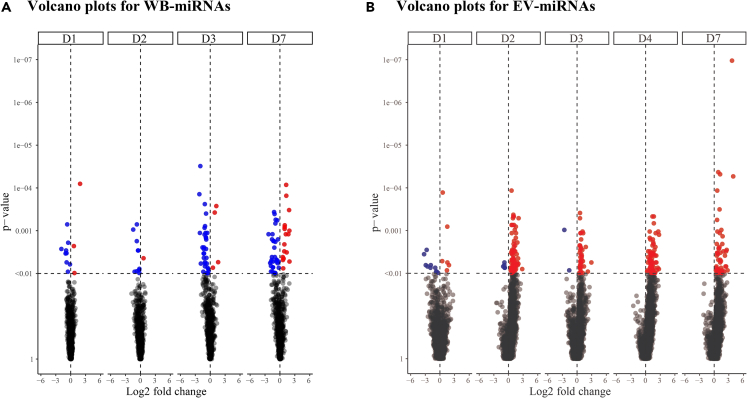
Differential expression of WB-miRNAs and EV-miRNAs early after rVSVΔG-ZEBOV-GP vaccination Normalized and baseline-adjusted Log2-transformed read counts were used to construct volcano plots displaying the differential miRNA expression in individuals given the rVSVΔG-ZEBOV-GP vaccine in comparison with the placebo group. The analyses were performed with Mann-Whitney test. Each data point represents one miRNA. Significant (p < 0.01) miRNAs with a higher or lower expression level are highlighted in red or blue, respectively. The p values are shown on a -Log10 scale for better visualization. Each dotted, horizontal line indicates the significance threshold (p < 0.01) and each dotted, vertical line indicates the border between lower (negative Log2 fold change value) or higher (positive Log2 fold change value). (A) Volcano plots for WB-miRNA at day (D) 1, D2, D3, and D7. (B) Volcano plots for EV-miRNA at D1, D2, D3, D4, and D7.

**Figure 3:**
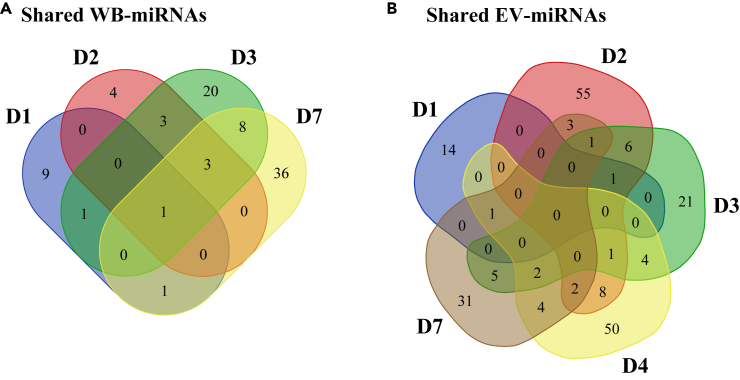
Shared significantly differentially expressed miRNAs at sampling time points post-vaccination The data over differential miRNA expression, in individuals given the rVSVΔG-ZEBOV-GP vaccine in comparison with the placebo group, were derived according to Figure 2 and used to create Venn diagrams. (A) The number of significantly (p < 0.01) differentially expressed miRNAs in WB at day (D) 1, D2, D3, and D7 after vaccination. (B) The number of significantly (p < 0.01) differentially expressed EV-derived miRNAs at D1, D2, D3, D4, and D7 after vaccination.

Next, the significant differences in the miRNA expression in WB and EV after rVSVΔG-ZEBOV-GP vaccination were evaluated. Despite the observed spread in the data, several DE miRNAs (p < 0.01) were found in the vaccine group as compared to the placebo group (Figure 2). As early as D1 post-vaccination, 12 miRNAs in WB resulted DE (nine reduced, three elevated) (Figure 2A; Table S1A; Figure S2A, Data S1). For the remaining three time points (D2, D3, and D7), most of the DE miRNAs were reduced in expression. The peak of the response in WB was detected at D7 with 49 DE miRNAs (32 reduced, 17 elevated). The WB-miRNA with the overall most significant p value was miR-199b (p < 0.0001), identified at D3, being also statistically significantly reduced at D2 (p = 9.41 × 10^−4^) and D7 (p = 0.002). In the EV compartment, 16 miRNA transcripts showed statistically significant change at D1, in response to the vaccine, most of which were present at a lower level (ten reduced, six elevated) (Figure 2B; Table S1B; Figure S2B, Data S2). For the remaining four time points (D2, D3, D4, and D7), most of the significant miRNAs displayed an increased expression. The peak of the response was seen at D2, with 77 DE transcripts (five reduced, 72 elevated). At D3, a total of 41 miRNAs (two reduced, 39 elevated) differed as compared to the placebos. Only 72 and 49 mRNA transcripts with an increased expression were detected at D4 and D7 post-vaccination, respectively. The EV-miRNA with the overall most significant p value was miR-4735-3p (p < 0.0001) and a 13-fold increase at D7 compared with the placebo group. This miRNA also showed significant increases at D2 (p = 6.45 × 10^−3^) and D4 (p = 3.86 × 10^−3^). Besides miR-199b andmiR-4735-3p, from WB and EV compartments, respectively, 3 WB-transcripts and 4 EV-transcripts significantly changed during at least three sequential sampling days (Figure 3). In WB, miR-29a (D1, D2, D3, and D7), miR-25 (D2, D3, and D7), and miR-4772 (D2, D3, and D7) were significantly downregulated (Figure 3A). In EV, miR-192-5p (D1, D2, and D3), piR-019420/gb/DQ596670 (D2, D3, and D4), miR-4423-3p (D3, D4, and D7), and miR-4732-5p (D3, D4, and D7) were significantly upregulated (Figure 3B). These results showed changes induced in miRNA profiles of whole blood (both cellular and extracellular vesicle) early after rVSVΔG-ZEBOV-GP vaccination.

### Correlation between early blood miRNA profile induced following vaccination and IgG antibody responses

**Figure 4:**
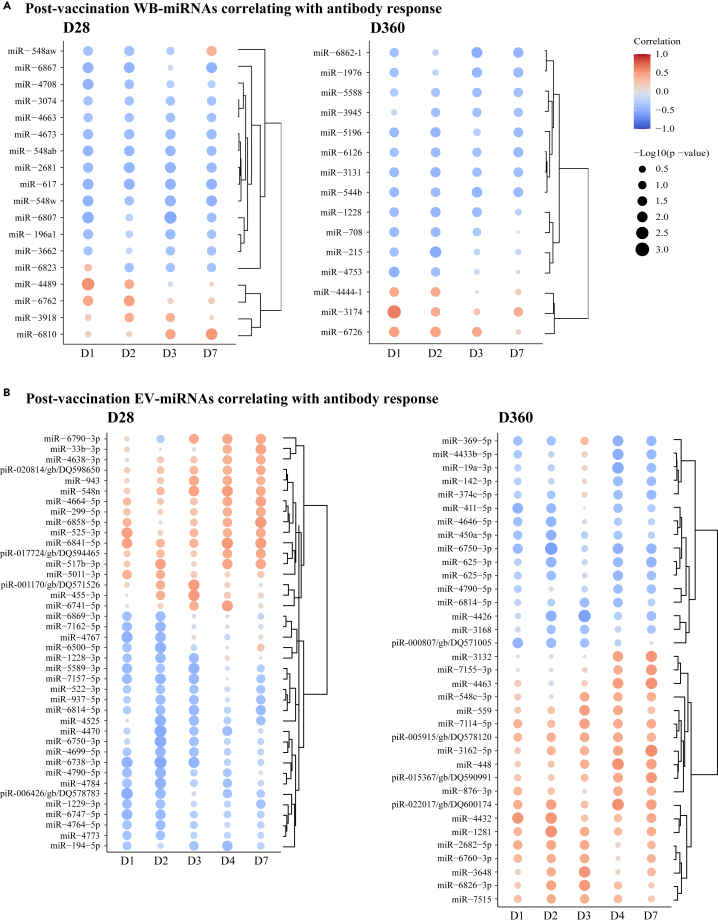
Correlation between post-vaccination miRNA profile and antibody responses Immunogenicity data were used to analyze the relation between miRNA expression in vaccinees and vaccine-induced ZEBOV-GP-specific antibodies. Specifically, Pearson correlation regressions were performed between day (D) 28 and D360 antibody titers (EU/mL) and normalized and baseline-adjusted Log2-transformed miRNA values. The size of each dot corresponds to the -Log10 p value in the correlation analysis. Only transcripts that correlated at minimum two time points were included. *p* < 0.05 was considered significant in the correlation analysis for WB-miRNAs and EV-miRNAs. Red color indicates positive correlation, while blue color indicates inverse correlation. The strenght of the colors corresponds to the size of the correlation coefficient. (A) Heatmaps displaying the correlation between WB-miRNA levels, at D1, D2, D3, and D7, and specific antibodies post-vaccination. (B) Heatmaps displaying the correlation between EV-miRNA levels, at D1, D2, D3, D4, and D7, and specific antibodies post-vaccination.

We sought to assess the correlation between early miRNA transcripts and ZEBOV-GP-specific IgG antibody responses up to one year after VSVΔG-ZEBOV-GP vaccination (Figure S3). ZEBOV GP specific IgG antibody titers at D28, the peak of the antibody response, and D360, the latest time point available, together with the miRNA data obtained from the study participants were used to perform correlation regressions. The levels of several miRNAs, measured during the first week after vaccination, statistically significantly correlated with the IgG antibody titers at D28 and D360 post-vaccination. Only miRNA transcripts that correlated significantly (p < 0.05) with IgG antibody titers on at least two sequential time points were selected for further analyses. In WB, four and three miRNAs displayed positive correlation (positive Pearson coefficient r) with the antibody titer at D28 and D360, respectively, while 14 and 12 miRNAs correlated inversely (negative r) (Figure 4A; Tables S2A and S2B; Figure S4A). In EV, 17 and 19 miRNAs correlated positively with the antibody titer at D28 and D360, respectively, while 23 and 16 miRNAs correlated inversely (Figure 4B; Tables S2D and S2E; Figure S4B). The strongest WB-miRNAs that positively correlated with the IgG antibody response at D28 and D360 were miR-4489 (D1: r = 0.54; p = 0.0025) and miR-3174 (D1: r = 0.62; p = 0.0005), respectively, while the strongest WB-miRNAs that negatively correlated with the IgG antibody response at D28 and D360 were miR-6807 (D3: r = −0.54; p = 0.0028) and miR-215 (D2: r = −0.52; p = 0.0055), respectively (Figure 4A; Tables S2A and S2B; Figure S4A). All EV-miRNAs that displayed the strongest correlation with the IgG antibody response at D28 correlated inversely with IgG antibody magnitude, and included miR-6738-3p (D2: r = −0.55; p = 0.0022). The most statistically significant EV-miRNAs that correlated with the antibody titer at D360 were miR-3162-5p (D7: r = 0.56; p = 0.0024) and miR-6750-3p (D2: r = −0.59; p = 0.0012) (Figure 4B; Tables S2D and S2E; Figure S4B). WB-miRNAs correlating with antibody response at D28 and D360 were unique to individual time points. However, one of the early-measured EV-transcripts, miR-6750-3p, correlated inversely with the vaccine-induced IgG antibody response both at D28 and D360 post-vaccination. These data document WB- and EV-miRNAs that correlate with the magnitude of ZEBOV-specific IgG antibody response induced up to 1 year after rVSVΔG-ZEBOV-GP vaccination.

### Correlation between baseline miRNAs and IgG antibody responses

**Figure 5:**
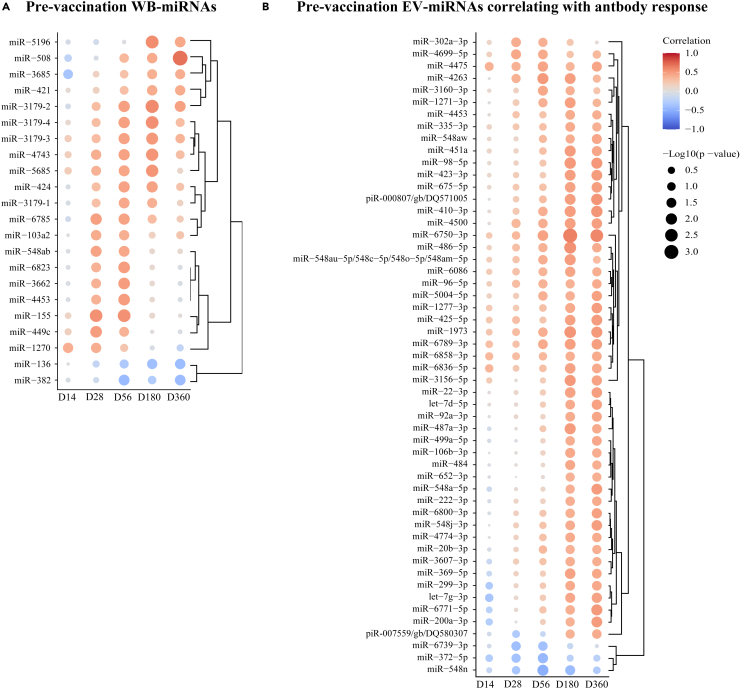
Correlation between pre-vaccination miRNA profile and post-vaccination antibody levels Immunogenicity data were used to analyze the relation between baseline miRNA expression in vaccinees and vaccine-induced ZEBOV-GP-specific antibodies. Specifically, Pearson correlation regressions were performed between day (D) 14, D28, D56, D180, and D360 antibody titers (EU/mL) and normalized Log2-transformed baseline miRNA values. The size of each dot corresponds to the -Log10 p value in the correlation analysis. Only transcripts that correlated at minimum two time points were included. p < 0.05 was considered significant in the correlation analysis for WB-miRNAs and EV-miRNAs. Red color indicates positive correlation, while blue color indicates inverse correlation. The strenght of the colors corresponds to the size of the correlation coefficient. (A) Heatmap displaying the correlation between WB-miRNA levels at D0 and specific antibodies post-vaccination. (B) Heatmap displaying the correlation between EV-miRNA levels at D0 and specific antibodies post-vaccination.

The importance of the association between the expression profiles at baseline (D0) and the IgG antibody responses following vaccination has recently drawn wide attention.^22^ Hence, we decided to explore the baseline miRNA expression and its relationship to individual vaccine ZEBOV-GP-specific IgG antibody responses (Figure 5; Table S2). The D0 sequencing data from the rVSVΔG-ZEBOV-GP group were used to perform correlation regressions with specific IgG antibody titers at D14, D28, D56, D180 and D360. Only transcripts that correlated significantly with at least two sequential IgG antibody time points were displayed (p < 0.05). Twenty WB-miRNAs and 50 EV-miRNAs showed positive correlation, while two WB-miRNAs and three EV-miRNAs showed inverse correlation with the ZEBOV-specific IgG antibody titers. The baseline WB-miRNA miR-508 showed the strongest significant positive correlation with the antibody titer (D360: r = 0.68; p = 0.0001), while miR-382 showed the strongest significant inverse correlation (D56: r = −0.43; p = 0.0214) (Figure 5A; Table S2C; Figure S5A). The baseline EV-miRNAs that displayed the strongest significant positive and inverse correlation with the antibody titer were miR-6750-3p (D180: r = 0.62; p = 0.0004) and miR-372-5p (D56: r = −0.50; p = 0.0061), respectively (Figure 5B; Table S2F; Figure S5B). Herein, we defined baseline miRNAs that correlate with the magnitude of ZEBOV-specific IgG antibody response induced up to one year after rVSVΔG-ZEBOV-GP vaccination.

### Identification of early miRNA signatures that predict ZEBOV-specific IgG antibody levels using machine learning algorithms

**Figure 6:**
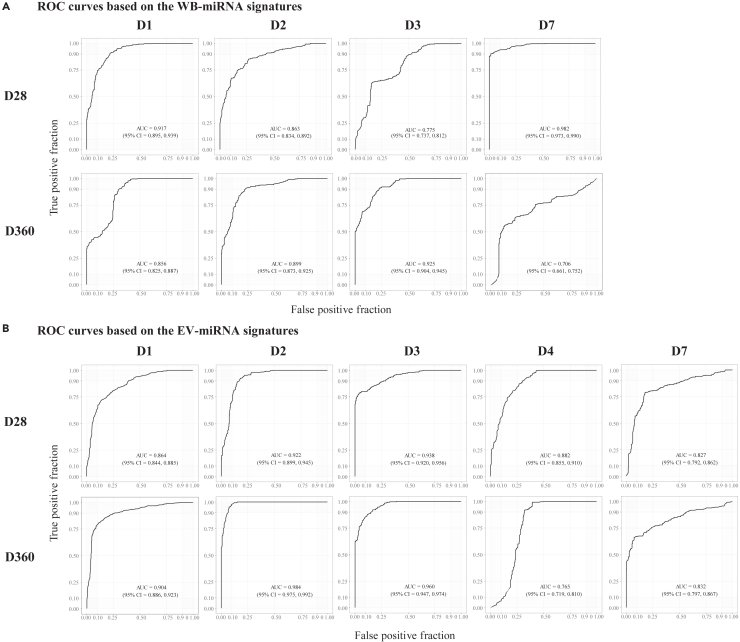
Prediction of vaccine-induced antibody responses based on the distinct WB- or EV-miRNA expression Normalized and baseline-adjusted Log2-transformed miRNA values and immunogenicity data were retrieved from rVSVΔG-ZEBOV-GP recipients. The levels of ZEBOV-GP-specific antibodies (EU/mL) were classified as low or high by using the median. Signatures including the most relevant miRNAs in predicting antibody levels were identified by using Recursive Feature Elimination (RFE). The machine learning algorithm Random Forest was used to evaluate the performance of each RFE-identified miRNA signature in classifying the antibody levels at day (D)28 and D360. K-fold cross validation (10-fold, 20 repeats) was used to evaluate the quality of the model. The classification performance of the model was assessed by evaluating the receiver operating characteristic (ROC) curve and area under the curve (AUC) including 95% confidence intervals (CI). (A) ROC curves over WB-miRNA time points (D1, D2, D3, and D7) and ZEBOV-GP-specific antibody levels at D28 and D360 post-vaccination. (B) ROC curves over EV-miRNA time points (D1, D2, D3, D4, and D7) and ZEBOV-GP-specific antibody levels at D28 and D360 post-vaccination.

Next, we employed machine learning algorithms (*Recursive Feature Elimination* and *Random Forest*) to identify early WB and EV miRNA signatures with a refined classification between low and high levels of ZEBOV-specific IgG antibodies at D28 and D360 post-vaccination. The ROC-curves show how well each miRNA signature discriminates between individuals developing low and high IgG antibody levels post-vaccination. WB-miRNAs signatures showed high classification performance (range AUC = 0.706–0.982) at all time points. The highest and the lowest AUCs were observed for the eleven-D7- and the two-D7-miRNAs signatures that predicted IgG antibody titers at D28 (AUC = 0.982) and D360 (AUC = 0.706), respectively (Figure 6A; Table S3A). Further, EV-miRNAs signatures displayed a high classification performance (range AUC = 0.765–0.984) at all time points. The highest and the lowest AUCs were detected for the thirty-D2 and the two-D4-miRNAs signatures that predicted antibody levels at D360 (AUCs = 0.984 and 0.765, respectively) (Figure 6B; Table S3B).

**Figure 7:**
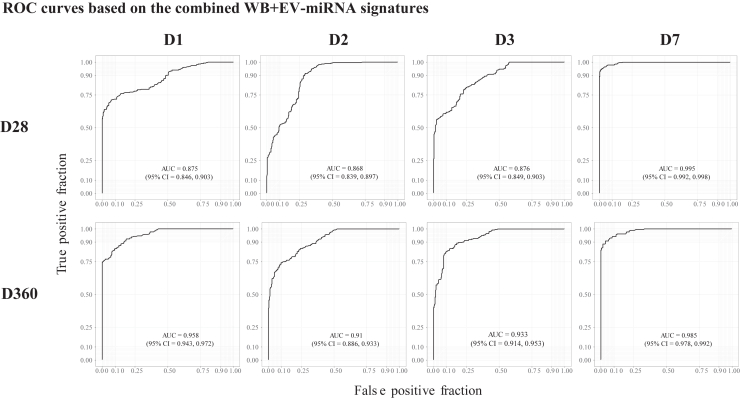
Prediction of vaccine-induced antibody responses based on the combined WB- and EV-miRNA expression Normalized and baseline-adjusted Log2-transformed miRNA values and immunogenicity data were retrieved from rVSVΔG-ZEBOV-GP recipients. Signatures including the most relevant miRNAs in predicting ZEBOV-GP-specific antibodies (EU/mL) levels, specified by using the median, were identified by using Recursive Feature Elimination (RFE). The machine learning algorithm Random Forest was used to evaluate the performance of each RFE-identified miRNA signature in classifying the antibody levels at day (D) 28 and D360. K-fold cross validation (10-fold, 20 repeats) was used to evaluate the quality of the model. The classification performance of the model was assessed by evaluating the receiver operating characteristic (ROC) curve and area under the curve (AUC) including 95% confidence intervals (CI). ROC curves are built over WB- and EV-miRNA time points (D1, D2, D3, and D7) and ZEBOV-GP-specific antibody levels at D28 and D360 post-vaccination.

By integrating the WB- and the EV-miRNA expression datasets, we identified combined WB + EV-miRNA signatures at each time point post-vaccination that showed an overall improved classification performance (range AUC = 0.868–0.995) in predicting the antibody levels (Figure 7). The highest and the lowest AUCs were observed for the fourty-D7 and the two-D2-miRNAs signatures that predict antibody levels at D28 (AUCs = 0.995 and 0.868, respectively) (Figure and Table S3C). Interestingly, the majority (61/79) of miRNAs identified in the combined signature belonged to the EV compartment. The miRNA transcripts that had a “variable importance in projection” (VIP) > 20% were used to perform a comparison of expression levels between the low and the high antibody groups. In total, the expression level of 14 WB- and 24 EV-transcripts differed significantly (p < 0.05 and <0.01, respectively) between the low and high antibody groups (Figures S6 and S7). The most significant WB-miRNA was miR-6810 (p = 0.0011), while the most significant EV-miRNA was miR-7151-3p (p < 0.0001), both having a higher expression level in individuals with high ZEBOV-GP-specific IgG antibodies 28 days and one year after vaccination, respectively. In the combined signatures, miR-4426 from the EV compartment was the most significant (p < 0.0001), showing a higher expression level in individuals with low ZEBOV-GP-specific IgG antibodies one year post-vaccination (Figure S8).

### Enrichment of target mRNA transcripts and pathways of the predictive miRNAs

**Figure 8:**
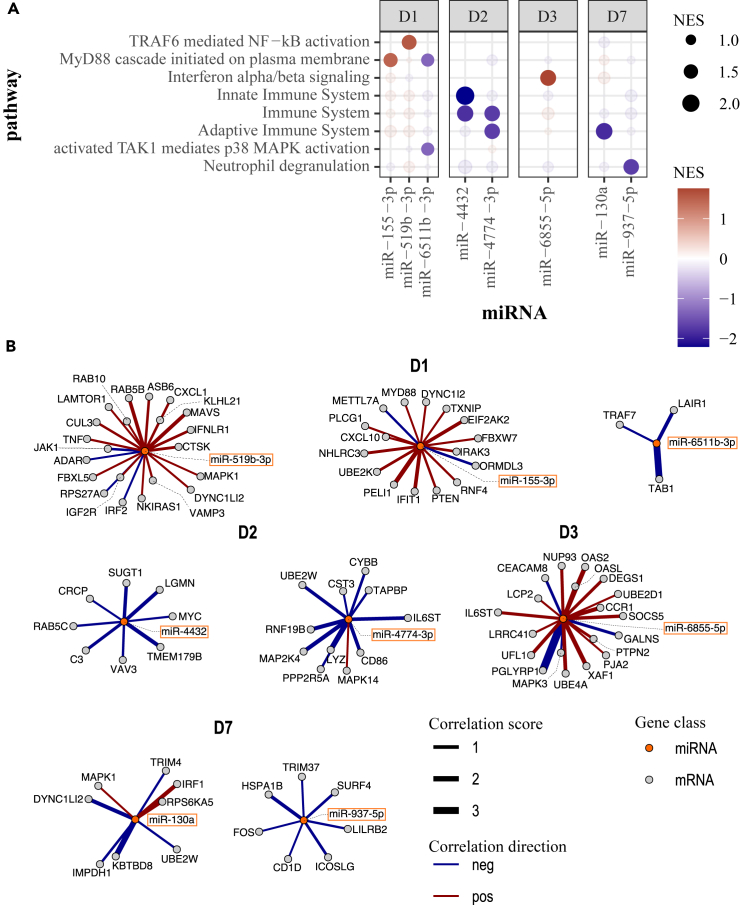
Enriched immune-related pathways of mRNA targets of antibody predictive miRNAs (A) Immune-related pathways associated with known targets of predictive miRNAs. Targets were ranked by their correlation coefficient with their corresponding miRNA in each post-vaccination day to run the Gene Set Enrichment Analysis. (B) Networks of correlated targets involved in immune-related pathways in each day post-vaccination. miRNAs are represented at the center, and their correlated target genes are connected via edges. Edges are colored to indicate the direction of correlation (positive: red, negative: blue) and scaled proportionally to the strength of the correlation, as denoted by the correlation score (correlation coefficient × -log_10_[p value]).

Finally, we integrated the WB- and EV- miRNAs most predictive of IgG antibody levels following vaccination (Figures S6 and S8) across all shared timepoints post-vaccination (D1, D2, D3, and D7) with whole genome transcriptomics data obtained from the same cohort. Known target mRNAs retrieved from IPA and miRNet databases were selected from the correlation analysis between miRNA expression and mRNA expression from the same blood samples of vaccinees. The gene set enrichment analysis performed to assess the significance of the miRNA-mRNA associations (Table S4) showed a signature comprising eight miRNAs whose associated mRNA targets were significantly enriched for immune-related functions (Figure 8; Table S5). Interestingly, several genes involved in key innate immune pathways, such as MyD88 cascade, activation of NF-kB, and interferon signaling (Figure 8A) showed a positive correlation with miR-155-3p (D1), miR-519b-3p (D1), and miR-6855-5p (D3). Conversely, genes involved in the activation of the p38 MAPK innate imune-signaling pathway and neutrophil degranulation inversely correlated with miR-6511b-3p at D1 and miR-937-5p at D7, respectively. Further, miR-4774-3p and miR-130a were negatively correlated with genes involved in adaptive immunity at D2 and D7, respectively (Figure 8A).

## Discussion

In the current study, we profiled longitudinally the global miRNA expression changes in the intracellular and the extracellular compartments of blood within one week after administration of the Ebola vaccine rVSVΔG-ZEBOV-GP. Further, we identified early miRNA signatures that were associated with, and could predict, the vaccine-induced IgG antibody response.

Several miRNAs identified herein have previously been reported to be involved in various infections as well as in shaping immune responses.^23^ miR-199b and miR-4735-3p were the most significant DE WB- and EV-miRNAs, respectively. Similar to our findings, the miR-199 family, which consists of miR-199a and miR-199b, has been reported to be down-regulated during H3N2 influenza infection, while reported to be upregulated in *Streptococcus pneumoniae* infection.^24^ Selective inhibition of miR-199b was also shown to modulate expression of genes involved in antiviral responses in natural killer cells of patients with severe and/or recurrent herpes simplex virus type 1 infection.^25^ miR-4735-3p has previously been described to target mammalian target of rapamycin (mTOR)-dependent signaling, by interacting with the long non-coding (lnc) RNA Cancer Susceptibility 2 (CASC2) that plays an important role in inhibiting proliferation and migration of adenocarcinoma cells.^26^ Of note, the kinase mTOR is involved in various cellular and metabolic processes that regulate the development of the immune response.^27^

To allow their use in clinical settings, biomarkers need to be long-lived in biological fluids. Thus, transcripts whose change is sustained over a longer period are of particular interest due to their potential use as biomarkers for vaccine-induced responses. In WB, the expression of miR-25 and miR-29a was significantly reduced on sequential days. Interestingly, their alterations have been reported in several virus-associated diseases, such as influenza A virus infection,^28^ hepatitis B infection,^29^ and hepatitis C virus infection.^30^ Further, dysregulation of miR-25 has been related to the development of sepsis-associated encephalopathy (SAE).^31^ Luo et al. reported that miRNA-25-3p is involved in inflammasome and microglia activation in SAE mice, by regulation of TLR4 and NLRP3 expression, corroborating its importance in inflammatory responses. In EV, miR-192-5p was elevated during the first three days after vaccination but it was not observed to be correlated with the IgG antibody titer. Interestingly, EV miR-192-5p was reported to suppress hyperinflammation and improve influenza vaccination efficacy by increasing specific IgG antibody production in aged mice.^32^ Moreover, miR-192-5p has previously been identified as a circulating marker of inflammation in a study defining plasma biomarkers for non-infective systemic inflammatory response syndrome, and was shown to positively correlate with levels of peroxiredoxin 1 (Prdx-1) that is released by immune cells during inflammation.^33^^,^^34^^,^^35^ Further, EV-miR-4732-5p, which increased during the second half of the week post-vaccination, has been reported to be involved in inhibition of *Shigella* infection.^36^

In case of an EVD outbreak, a quick vaccine-induced immunity would offer a great value. Additionally, the duration of a specific immune response following vaccination is an essential aspect of an efficacious vaccine in order to prevent disease. Hence, to find early biomarkers that correlate with and even predict antibody levels early and/or late after vaccination would be desirable. In our work, we pinpointed early miRNAs that correlated with and/or could predict the magnitude of ZEBOV-GP-specific IgG antibodies. Among them, miR-136 has previously been linked to antiviral activity against H5N1 influenza A virus^37^ as well as to be involved in hepatitis C virus infection.^38^ Furthermore, miR-448 has been associated with inflammatory responses through involving STAT1-signaling during *Mycobacterium tuberculosis* infection^39^ and TLR4-mediated macrophage polarization in diabetes,^40^ while miR-19a-3p is known to modulate NF-κB-signaling.^41^ We also found that expression of WB-miR-6810 and EV-miR-4426 strongly correlates with the antibody titer and significantly classifies high and low antibody levels. However, little is known about their function in the biological systems.^42^^,^^43^ Further, miR-155-3p was defined among the DE miRNAs as a good classifier of vaccine-induced IgG antibody response, which showed correlation with expression of genes involved in TLR signaling in the blood of the US cohort. miR-155 is among the most extensively studied miRNAs with documented role in regulation of immune responses such as antibody production and macrophage polarization.^44^^,^^45^ Another strong predictor of antibody levels following rVSVΔG-ZEBOV-GP vaccination was miR-130a which has been shown to regulate macrophage’s fibrogenesis in inflammation^46^ and presented an inverse correlation with adaptive immunity genes in the blood of vaccinees.

Baseline signatures predictive of a protective humoral response following vaccination can inform strategies to enhance the vaccine efficacy. Discovery of pre-vaccination baseline biomarkers has recently been considered.^22^ In the current study, we identified several miRNAs that correlate with the vaccine-induced IgG antibody response. The baseline WB-miRNAs showing the strongest positive and inverse correlation with the humoral response were miR-508 and miR-382, respectively. Intriguingly, several studies reported that these miRNAs are implicated in different diseases via PI3K/AKT pathway regulation.^47^^,^^48^^,^^49^ PI3K/Akt signaling pathway is a crucial intracellular signal transduction pathway with multi-faceted effects on cell metabolism, growth, proliferation, and survival, PI3K/Akt signaling pathway. It has been reported to be dysregulated during viral infections^50^ and to be involved in intracellular control of *Salmonella typhimurium* and *Mycobacterium tuberculosis* infection.^51^ Three of the baseline EV-miRNAs that strongly positively correlated with antibody responses were miR-486-5p, miR-410-3p, and miR-98-5p. Of note, miR-486-5p and miR-410-3p were identified as markers in sepsis.^52^^,^^53^ Further, the miR-486-5p and miR-410-3p along with miR-98-5p have been shown to regulate cytokine response via the NF-κB pathway^54^ and IL-6 signaling,^55^ respectively.

So far, only a limited number of reports studied miRNA changes after vaccination in humans.^56^^,^^57^^,^^58^^,^^59^^,^^60^ In line with our findings, Miyashita and colleagues showed that miR-625-3p significantly correlated with the antibody titer after COVID-19 vaccination^56^ and miR-451a levels were highly expressed in individuals vaccinated with influenza vaccine.^57^ The studies on Ebola vaccination with the rVSVΔG-ZEBOV-GP vaccine^59^ and EVD^60^ profiled circulating miRNAs only, although not vesicle-bound. In particular, although RT-qPCR-based and not global miRNA profiling, Fischer’s study reported miR-199a-5p as differentially regulated, as observed in our study. Two of the downregulated WB miRNAs at D3 in our study (miR-23a-3p and miR-221-3p) matched two of their top ten downregulated circulating miRNAs at the same time point. However, none of the miRNAs correlating with ZEBOV-GP IgG antibody titers in their study were detected as significant correlates in our study.^59^ Finally, the circulating miR-20a-5p and miR-320a were differentially regulated in our and Duy’s studies, miR-320a being upregulated in both,^60^ albeit none of the miRNAs showed correlation with viral load in our work.

This report describes miRNome modulations, in both the intracellular and circulating parts of the blood, at baseline and early after vaccination. Importantly, we have identified miRNAs at early time points post-vaccination that were correlated with the rVSVΔG-ZEBOV-GP vaccine immunogenicity one month and one year following vaccination. These miRNA transcripts may potentially be used as potential liquid biomarkers to predict the magnitude and durability of vaccine-induced antibody responses. Finally, certain baseline miRNAs significantly correlate with the vaccine-induced IgG antibody response, suggesting that these transcripts may be used as putative baseline biomarkers to predict vaccine-induced antibody response. These results warrant further exploration on mechanisms underlying the differentially expressed miRNAs to enhance our understandings of miRNAs’ roles in immunological pathways and vaccine effects.

### Limitations of the study

The current study has limitations. Firstly, we acknowledge the descriptive nature of our research, which primarily focused on miRNA profiling following vaccination without investigating the function of the identified miRNAs in relation to immune responses. While our study identifies potential biomarkers of vaccine-induced IgG antibody responses, the direct involvement of these miRNAs in immunological processes remains to be thoroughly investigated. Secondly, the sample size used in this study was relatively small. Additionally, the analysis relied on a single cohort for miRNome profiling and identification of miRNA predictive signatures of the vaccine-induced antibody response, and as such the defined signatures warrant future validation in an independent cohort. Finally, this study has limitations in data analysis. In particular, the WB dataset included the miRNAs in their precursor form, therefore the annotations of the strand, which is present in the mature form of miRNAs, were not included.

## STAR★Methods

### Key resources table

**Table:** 

REAGENT or RESOURCE	SOURCE	IDENTIFIER
**Biological samples**		

Whole blood from healthy adult vaccinated with rVSVΔG-ZEBOV-GP vaccine or placebo	Merck Sharp & Dohme Corp. (MSD)	NCT02314923 or V920-004
Serum from healthy adult vaccinated with rVSVΔG-ZEBOV-GP vaccine or placebo	Merck Sharp & Dohme Corp. (MSD)	NCT02314923 or V920-004

**Critical commercial assays**		

PAXgene blood miRNA kit	PreAnalytiX	N/A
Ion AmpliSeq™ Transcriptome Human Gene Expression Kit	Thermo Fisher Scientific	N/A
Ion PI Hi-Q Sequencing 200 Kit	Thermo Fisher Scientific	N/A
ExoRNeasy Serum/Plasma kit	Qiagen	N/A
QIAseq miRNA library kit	Qiagen	N/A

**Deposited data**		

miRNA-sequencing raw and processed data	NCBI-GEO	Accession numbers: GSE240572 and GSE242207

**Software and algorithms**		

R software	R Core Team^63^	N/A
Calculate and draw custom Venn diagrams.	https://bioinformatics.psb.ugent.be/webtools/Venn/	N/A
Simca v17	Sartorius/Umetrics	N/A

### Resource availability

#### Lead contact

Further information and requests for resources should be directed to and will be fulfilled by the lead contact, Eleonora Vianello (e.vianello@lumc.nl).

#### Materials availability

This study did not generate new unique reagents.

#### Data and code availability

• miRNA-sequencing raw and processed data have been deposited to NCBI Gene Expression Omnibus (GEO) and are publicly available as of the date of publication. Accession numbers are GSE240572 and GSE242207.
• Any additional data and information are available from the lead contact upon reasonable request. Requests will be assessed for scientific rigor before being granted, and a material transfer agreement might be required.
• This paper does not report original code. Requests for code should be directed to the lead contacts.

### Experimental model and subject details

A miRNomic profiling was conducted on RNA samples, derived from either WB (intracellular) or serum-isolated EV (extracellular), from a subset of forty eight healthy individuals enrolled in a randomized, double-blinded, placebo-controlled, dose-finding clinical trial conducted at several U.S. sites (NCT02314923 or V920-004). The same study participants were used for both sample types. All participants provided written informed consent and the study was approved by the Chesapeake Institutional Review Board (Columbia, MD, USA) and by the Crescent City Institutional Review Board (New Orleans, LA, USA). The participants included in this analysis received a single intramuscular vaccination with 1.0 mL of 2×10^7^ plaque-forming units (pfu) of the rVSVΔG-ZEBOV-GP vaccine (n = 30) (Merck Sharp & Dohme Corp. (MSD), a subsidiary of Merck & Co., Inc., Kenilworth) or 0.9% saline (placebo, n = 18). The individuals that received the vaccine consisted of 53% female and 47% male (16/14), with an average age of 37 years (range 19–57), while the individuals that received the placebo consisted of 39% female and 61% male (7/11), with an average age of 34 years (range 18–60) Venous blood samples were drawn from the study participants pre- and post-vaccination. Samples were collected between December 26, 2014, and June 23, 2016. The WB and the separated serum fraction were stored at −80°C prior to usage. The samples, in addition to clinical data, were made accessible through the Innovative Medicines Initiative 2-supported VSV-EBOPLUS consortium.^61^

### Methods details

#### WB-RNA isolation, cDNA library construction, miRNA sequencing, and bioinformatics analysis

WB was collected in PAXgene blood RNA tubes (PreAnalytiX, Hombrechtikon, Switzerland) (time points: D0, D1, D2, D3, and D7). WB-RNAs, including miRNAs, were isolated by using the PAXgene blood miRNA kit (PreAnalytiX), according to the manufacturer’s automated protocol. This kit guarantees the extraction of intracellular RNA and miRNA upon cell lysis and stabilization of intracellular RNA during whole blood collection in PAXgene blood RNA tubes (PreAnalytiX). The elutes were quantified with a Qubit fluorometer (ThermoFisher Scientific, Wilmington, DE, USA) by using the RNA Broad Range assay kit (ThermoFisher Scientific). Samples were stored at −80°C prior to cDNA synthesis. RNA quality was assessed and libraries for miRNA sequencing were prepared, quantified, and sequenced at GenomeScan BV (Leiden, The Netherlands). Briefly, RNA quality was assessed by using Agilent RNA (15 nt) fragment analysis (Agilent, Santa Clara, CA, USA). Libraries were prepared using the NEBNext Multiplex Small RNA Library Prep Kit for Illumina (New England BioLabs, Ipswich, MA, USA) (Index Primers 1–48, #7560S). Their quality and yield were measured by Agilent HS NGS fragment analysis (Agilent). The final products have approximate size of 150 bp. Due to quality issues with a few WB-RNA samples, one vaccinee and one placebo, were removed from further analyses. The 1.1 nM libraries, derived from the WB RNAs, were sequenced using the NovaSeq 6000 sequencer with a 150 bp paired-end protocol. Between 9 and 41 million reads were obtained for each sample (average of 17 million reads per sample). Bioinformatics analysis was performed at Future Genomics Technologies BV (Leiden, The Netherlands). Briefly, adapters and indexes have been removed by using cutadapt v2.10. Reads were aligned to GRCh38 by using bowtie2 v2.3.5.1. Alignments were processed using samtools v1.9 to obtain a sorted bam-file which was used as input in HTSeq.count v0.13.0. No information on the pre-miRNA stem-loop strand was available for the WB-data.

#### cDNA library construction and mRNA sequencing

50 ng of total RNA extracted from WB was reverse transcribed to produce cDNA, using SuperScript VILO cDNA Synthesis Kit (Thermo Fisher Scientific). cDNA was used to prepare libraries with the Ion AmpliSeq Transcriptome Human Gene Expression Kit (Thermo Fisher Scientific), according to the manufacturer’s recommendation. This kit amplifies 20,812 human genes. Libraries were barcoded with Ion Xpress Barcode Adapters and purified using Agencourt AMPure XP Magnetic Beads (Beckman Coulter, Brea, CA, US). Libraries were quantified using Ion Library TaqMan Quantitation Kit (Thermo Fisher Scientific) on a QuantStudio 5 Real-Time PCR System (Thermo Fisher Scientific). Libraries were diluted to a concentration of 40 p.m. and sequenced on chips (Ion PI Chip Kit v3, Thermo Fisher Scientific) loaded using Ion Chef System (Thermo Fisher Scientific) and the IonPI Hi-Q Chef Kit (Thermo Fisher Scientific) according to the manufacturer’s protocol. Sequencing reactions were performed on an Ion Proton Sequencer (Thermo Fisher Scientific) using Ion PI Hi-Q Sequencing 200 Kit (Thermo Fisher Scientific). Raw data counts were obtained from Ion Proton Sequencer.

#### EV-RNA isolation, cDNA library construction, miRNA sequencing, and bioinformatics analysis

Sera were collected from blood samples (time points: D0, D1, D2, D3, D4, and D7). ExoRNeasy Serum/Plasma kit (Qiagen) was used to isolate EV-RNAs, according to the manufacturer’s instructions. After thawing, 500 μL of each serum sample was centrifuged at 16,000 × *g* for 10 min at 4°C to sediment larger particles. Exosomes and other extracellular vesicles were separated, using a membrane-based system, from 450 μL of centrifuged sera. Purified vesicles were analyzed by both scanning and transmission electron microscopy, which showed round-shaped structures ranging from 50 to 250 nm in diameter. The majority of the vesicles had the size of exosomes, i.e., 30–100 nm^62^ (data not shown). Total RNAs were extracted and eluted in 14 μL nuclease-free water and stored at −80°C prior to cDNA synthesis. The construction of cDNA libraries was performed using QIAseq miRNA library kit and corresponding index kit (Qiagen), according to manufacturer’s instructions. Briefly, the 3′ and 5′ ends of the RNAs were ligated sequentially to adapters. Next, each sample was tagged with a unique molecular index (UMI) during reverse transcription. A cleanup of the resulting cDNA product was performed using magnetic beads. The cDNA libraries were amplified with a universal forward primer and a reverse primer that assigned a unique custom index to each sample. The amplified libraries were cleaned up using magnetic beads. Each library concentration was determined using Qubit dsDNA HS assay kit and Qubit 3.0 fluorimeter (Thermo Fisher). Library quality control was accomplished with Tapestation HS-D1000 system (Agilent) analysis. The expected peak for the library constructs was around 173 bp. A limited number of samples possessed a second peak (>25% of the height of the miRNA peak) for a larger fragment and was therefore re-purified with magnetic beads as well as re-analyzed with regards to concentration and purity. One vaccinee EV sample was excluded from further use due to persisting quality problems despite re-purification. The library samples, each with a concentration of 4 nM, were pooled in three separate batches (n = 96, n = 96 and n = 90). The concentration and purity of the pools were checked before submitted for sequencing. The 4 nM library pools, derived from extracellular vesicular RNA, were run on an Illumina NovaSeq 6000 sequencer with a 75 bp single read protocol, using the 75-cycle high output kit (Illumina). Between 11 and 33 million reads were obtained for each sample (average of 20 million reads per sample). The retrieved output data came in FASTQ format. Sequences were quantified according to the unique molecular index (UMI) counts for each transcript, using a primary analysis software tool (www.geneglobe.qiagen.com/analyze).

### Quantification and statistical analysis

Counts from WB and EV samples were normalized by sequencing depth, dividing each sample with the column sums and multiplying them by 1 M. Baseline adjustment was performed individually by dividing the normalized data at each time point post-vaccination by D0. All values were Log2-transfomed before further analyses. The R software^63^ v4.0.1 was used for the analyses.

Orthogonal Partial Least Square Discriminant Analysis (OPLS-DA) was used to evaluate how well the rVSVΔG-ZEBOV-GP group clustered from the placebo group at each time point in the baseline-adjusted miRNA data.^64^^,^^65^^,^^66^ Evaluation of the models were based on the OPLS-DA performance measures R^2^ and Q^2^ (the closer to 1, the better model performance). The Simca v17 (Sartorius/Umetrics, Goettingen, Germany) was used for the analyses, with the default procedure of a 7-fold cross-validated ANOVA for significance testing, and as a diagnostic tool for assessing the reliability of the OPLS-DA models.^67^

Differential expression analysis (DEA) was done using the baseline-adjusted values to identify DE miRNAs by comparing the vaccinees versus the placebos. The Mann-Whitney test statistics was applied. The fold change between the groups was calculated using the means. miRNAs with p < 0.01 were considered DE. The results of this analysis were visualized with Volcano plots (using the R package *ggplot2*^68^ v3.3.5). Venn diagrams were generated using the online tool to calculate and draw custom Venn diagrams.^69^

To correlate miRNAs at early time points post-vaccination (D1-D7) with immunogenicity at D28 and D360, a pairwise Pearson correlation analysis between miRNA expression in vaccinees and ZEBOV-GP-specific antibody titer (EU/mL) was carried out using the baseline adjusted Log2-transformed miRNA values. Data on ZEBOV-GP-specific IgG antibody titers of the study participants were obtained from a clinical database provided by MSD. Briefly, the serum samples were assessed by Q2 Solutions, Durham, NC, formerly Focus Diagnostics, using a ZEBOV-GP specific ELISA based on the assay developed by the Filovirus Animal Non-Clinical Group (FANG).^70^ For this analysis only rVSVΔG-ZEBOV-GP samples were used. When applicable, five miRNA time points (D1, D2, D3, D4, and D7) and an early (D28) and a late (D360) antibody time point were considered for the correlation analyses. Pearson correlation analysis was also performed between the baseline miRNA expression (day 0) and antibody titers at D14, D28, D56, D180, and D360 post-vaccination. Results were visualized with correlation heatmaps (using the R package *heatmaply*^71^ v1.1.0).

A prediction model was developed by feature selection, using the Recursive Feature Elimination (RFE) (R package *caret*^72^ v6.0.90), to identify the most relevant miRNAs that predict the antibody titer (target variable) at D28 and D360 post-vaccination. Only data from the rVSVΔG-ZEBOV-GP group were included. The levels of ZEBOV-GP-specific IgG antibodies (EU/mL) detected at each time point were divided into low or high by using the median of the respective time point. The machine learning algorithm Random Forest (RF) (R *randomForest*^73^ v4.6.14) was then used to rank the RFE-identified baseline-adjusted Log2-transformed miRNA values, from D1-D7, that performed best to predict the antibody level at each time point. K-fold cross validation (10-fold, 20 repeats) was used to evaluate the quality of the model. The classification performance of the model was assessed by evaluating the receiver operating characteristic (ROC) curve and area under the curve (AUC), including 95% confidence intervals (CI). For each random forest model a variable importance score (Variable Importance in Projection (VIP)) was calculated to quantify the importance of each feature kept in the final model. Using a relative influence cut-off of >20 (the most important feature was scaled to an importance of 100) boxplots were produced for each feature comparing the miRNA levels between the low and high ZEBOV-specific IgG antibody groups.

mRNA targets of the miRNA signatures that better predicted high and low antibody levels were acquired from the Ingenuity Pathway Analysis (IPA) web-interface and miRNet database via the *miRNetR* R package. The expression of mRNAs on each post-vaccination day were baseline- and log2-normalized with respect to D0 expression, mirroring what was performed for miRNAs. Twenty patients which had matching miRNA and mRNA expression data available were used to perform correlation analysis. Pairwise Pearson correlations were performed between the baseline normalized expression of predictive miRNAs and their known mRNA targets using the *rcorr* function in the R package *Hmisc* v5.0-1. Correlation pairs with a p < 0.05 were considered significant. Target mRNAs were ranked according to the correlation coefficient with respect to their corresponding predictive miRNA. These ranked lists of mRNAs correlated to predictive miRNAs were used to run a gene set enrichment enalysis (GSEA) using the *fgsea* function of the fgsea^74^ R package and Reactome gene-pathway associations.^75^ Significantly enriched pathways were those with an adjusted p < 0.05. Immune-related pathways were selected among those significantly enriched. The *fgsea* Normalized Enrichment Score (NES) was used as a reference to determine whether the selected pathways were enriched for mRNAs positively (NES>0) or negatively (NES<0) correlated with predictive miRNA expression. Networks of miRNA and their correlated target mRNAs were constructed using the miRNAs and mRNAs associated with the pathways enriched in the GSEA. The selected mRNAs were those composing the leading-edge of the *fgsea* enrichment. Leading-edge genes correspond to the top positively or negatively correlated mRNAs that are also members of the enriched Reactome pathways. The leading-edge subset can be interpreted as the core of a gene set that accounts for the enrichment signal. The R packages *igraph* and *ggraph* were used to build and plot the networks, respectively. Edges were colored by the direction of the miRNA-mRNA correlation and the thickness was set to be proportioinal to the absolute value of the correlation coefficient.

## Supplemental information

Document S1. Figures S1‒S8 and Data S1 and S2

Table S1. Differential Expression Analysis, related to Figure 2 and 3 Table S1A. Differential expression analysis for WB-miRNAs, vaccinees vs. placebos. Baseline-adjusted Log2-transformed data. Mann-Whitney test with Benjamini-Hochberg correction for multiple testing. Table S1B. Differential expression analysis for EV-miRNAs, vaccinees versus placebos. Baseline-adjusted Log2-transformed data. Mann-Whitney test with Benjamini-Hochberg correction for multiple testing.

Table S2. Pearson correlation between miRNAs and antibody concentration at sampling timepoints, related to Figure 4 and 5 Table S2A. Pearson correlation between WB-miRNAs at D1, D2, D3, and D7 and ZEBOV-GP-specific antibody concentration at D28. Baseline-adjusted Log2-transformed data. Table S2B. Pearson correlation between WB-miRNAs at D1, D2, D3, and D7 and ZEBOV-GP-specific antibody concentration at D360. Baseline-adjusted Log2-transformed data. Table S2C. Pearson correlation between WB-miRNAs at baseline (D0) and ZEBOV-GP-specific antibody concentration at D14, D28, D56, D180, and D360. Log2-transformed data. Table S2D. Pearson correlation between EV-miRNAs at D1, D2, D3, D4 and D7 and ZEBOV-GP-specific antibody concentration at D28. Baseline-adjusted Log2-transformed data. Table S2E. Pearson correlation between EV-miRNAs at D1, D2, D3, D4 and D7 and ZEBOV-GP-specific antibody concentration at D360. Baseline-adjusted Log2-transformed data. Table S2F. Pearson correlation between EV-miRNAs at baseline (D0) and ZEBOV-GP-specific antibody concentration at D14, D28, D56, D180, and D360. Log2-transformed data.

Table S3. miRNA signatures that predict antibody levels, related to Figure 6 and 7 Table S3A. WB-miRNA signatures that can predict antibody levels (low or high) in vaccinees. Table S3B. EV-miRNA signatures that can predict antibody levels (low or high) in vaccinees. Table S3C. Combined WB- and EV-miRNA signatures that can predict antibody levels (low or high) in vaccinees.

Table S4. Correlation between miRNA that predict the antibody levels and the mRNA targets, related to Figure 8

Table S5. Immune-related pathways associated with mRNA targets of miRNAs that predict antibody levels, related to Figure 8
