# Supplementary material for: Global blood miRNA profiling unravels early signatures of immunogenicity of Ebola vaccine rVSVΔG-ZEBOV-GP
Source: iScience. 2023 Nov 23;26(12):108574. doi: 10.1016/j.isci.2023.108574 (PMC10755791; doi:10.1016/j.isci.2023.108574)
Supplement: Document S1. Figures S1‒S8 and Data S1 and S2 [file mmc1.pdf]

iScience, Volume *26*

Supplemental information

Global blood miRNA profiling unravels early signatures of immunogenicity of Ebola vaccine rVSVΔG-ZEBOV-GP

Eleonora Vianello, Josefine Persson, Björn Andersson, Suzanne van Veen, Thomaz Lüscher Dias, Francesco Santoro, Malin Östensson, Ogonna Obudulu, Christopher Agbajogu, Sara Torkzadeh, VSV-EBOVAC, VSV-EBOPLUS Consortia, Helder I. Nakaya, Donata Medaglini, Claire-Anne Siegrist, Tom H.M. Ottenhoff, and Ali M. Harandi

Supplemental Information

Figure S1

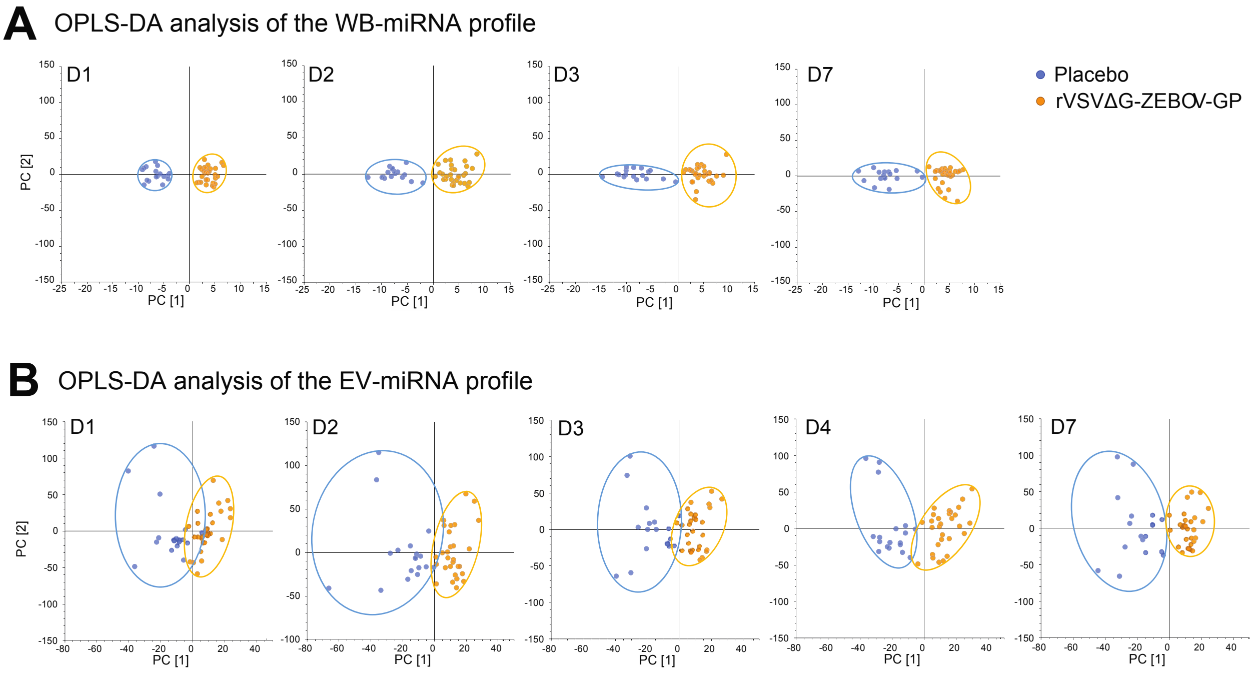

Figure S1. Data overview of the WB-miRNA and the EV-miRNA profile during the first week after rVSVΔG-ZEBOV-GP vaccination, Related to Figure 1.
The normalized and Log2-transformed sequencing data were modelled in two-dimensional OPLS-DA plots, a statistical multivariate data analysis technique. The horizontal direction of the plots displays the inter-group variation, while the intra-group variation can be seen in the vertical direction of the plots. (A) Score plots of OPLS-DA analysis of the WB-miRNA profile identified (1+1+0) components at day (D) 1, D2, D3, and D7. The models explained 12-16% (R2X WB-miRNA) of the inter-group variance and 85-92% (R2Y WB-miRNA) of the of the intra-group variation. (B) Score plots of OPLS-DA analysis of the EV-miRNA profile identified (1+1+0) components at D1, D2, D3, D4, and D7. The models explained 47-53% (R2X EV-miRNA) of the inter-group variance and 61-79% (R2Y EV-miRNAs) of the of the intra-group variation.

Figure S2

A Kinetics of the top 5 differentially expressed WB-miRNAs

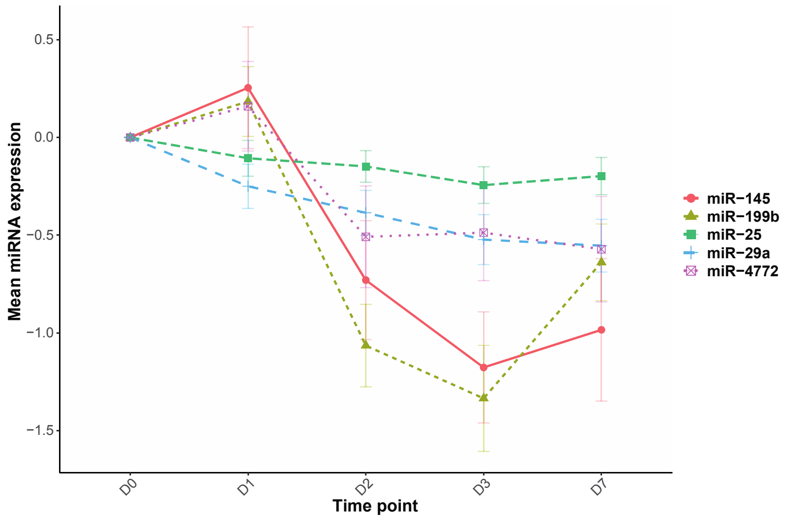

B Kinetics of the top 5 differentially expressed EV-miRNAs

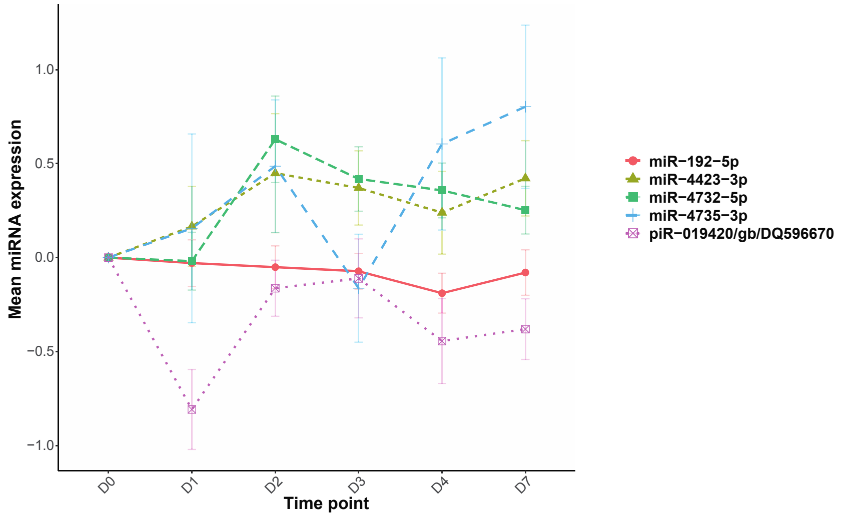

Figure S2. Expression kinetics of the top 5 differentially expressed WB and EV miRNAs, Related to Figure 2 and 3.

The Log2-transformed and baseline adjusted expression data of the top 5 differentially expressed miRNAs from WB and EV compartment in individuals that received the rVSVΔG-ZEBOV-GP vaccine were used. The line plot per each miRNA shows the mean expression over time (D0-D7). The standard error is reported as bar per each miRNA and time point. (A) WB-miRNAs. (B) EV-miRNAs.

Figure S3

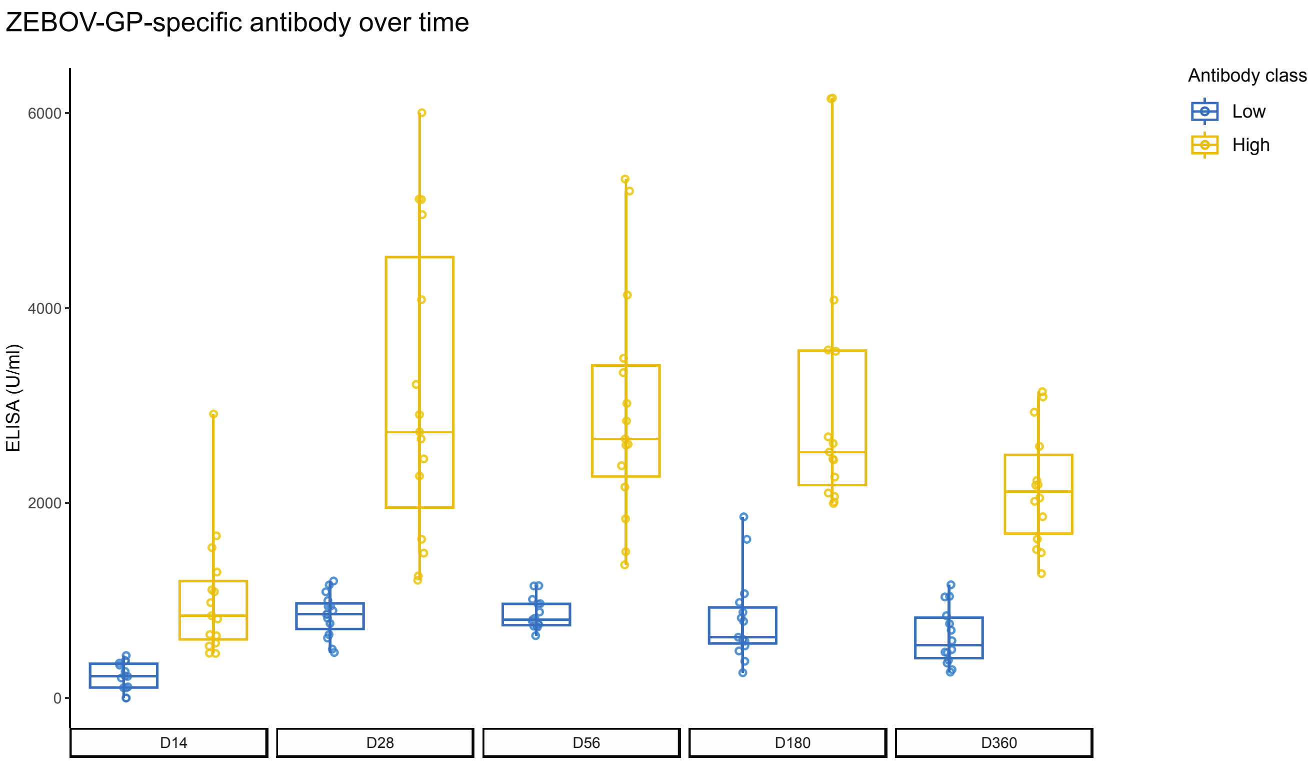

Figure S3. ZEBOV-GP-specific IgG antibody levels, Related to Figure 4 and 5.
ZEBOV-GP-specific antibody titers from the study participants receiving the rVSVΔG-ZEBOV-GP vaccine were obtained from the MSD-provided clinical database. The levels were classified as either low or high, determined by using the median per each timepoint (D14, D28, D56, D180, D360) and shown by using box-and-whiskers plots (5-95 percentiles).

Figure S4

A Kinetics of the top 5 WB-miRNAs that correlate with the antibody titer

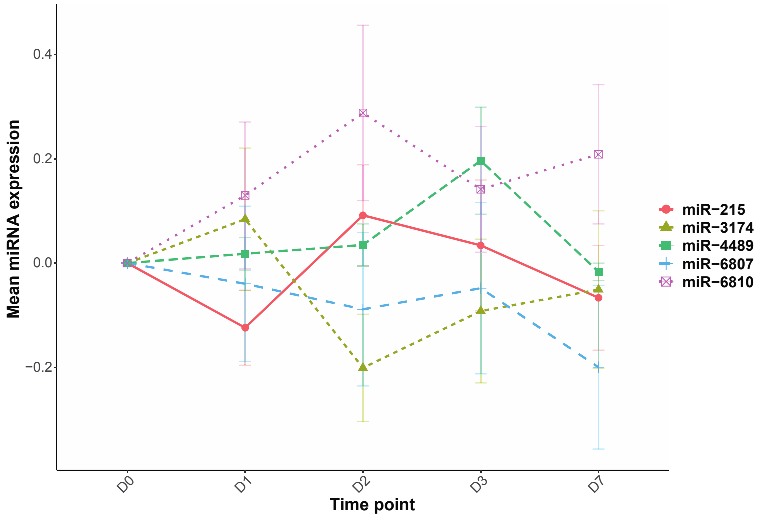

B Kinetics of the top 5 EV-miRNAs that correlate with the antibody titer

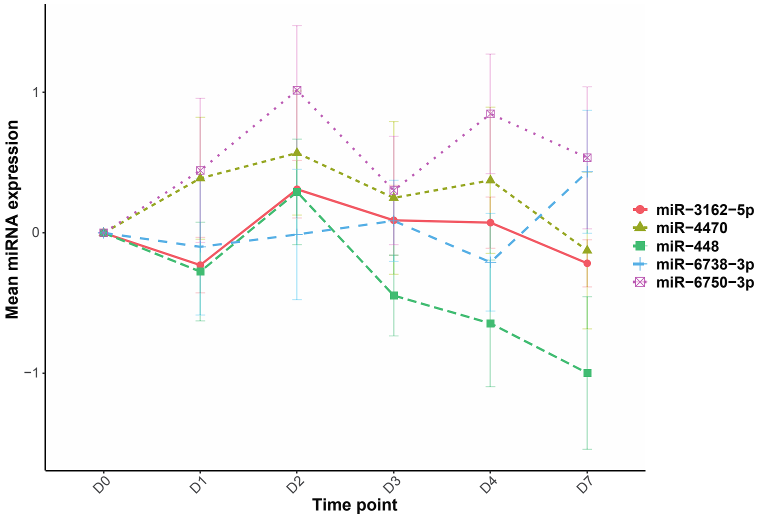

Figure S4. Expression kinetics of the top 5 WB and EV miRNAs that correlate with the ZEBOV-GP-specific IgG antibody titer, Related to Figure 4.
The Log2-transformed and baseline adjusted expression data of the top 5 miRNAs from WB and EV compartment that correlate with the ZEBOV-GP-specific IgG antibody titer in individuals that received the rVSVΔG-ZEBOV-GP vaccine were used. The line plot per each miRNA shows the mean expression over time (D0-D7). The standard error is reported as bar per each miRNA and time point. (A) WB-miRNAs. (B) EV-miRNAs.

Figure S5

A Kinetics of the top 5 baseline WB-miRNAs that correlate with the antibody titer

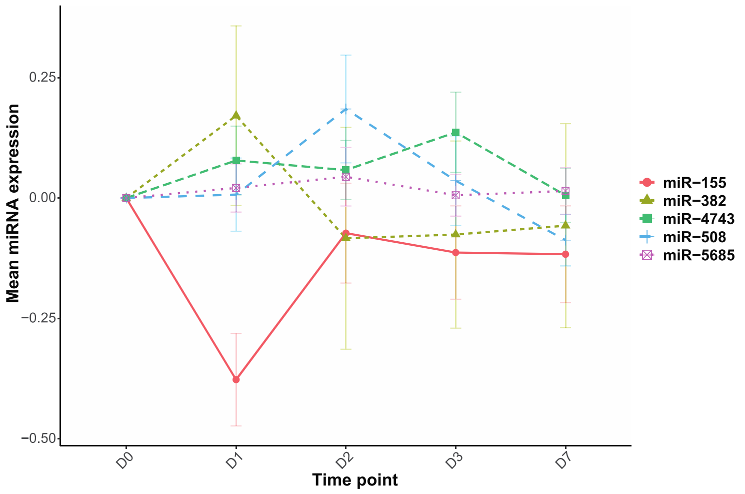

B Kinetics of the top 5 baseline EV-miRNAs that correlate with the antibody titer

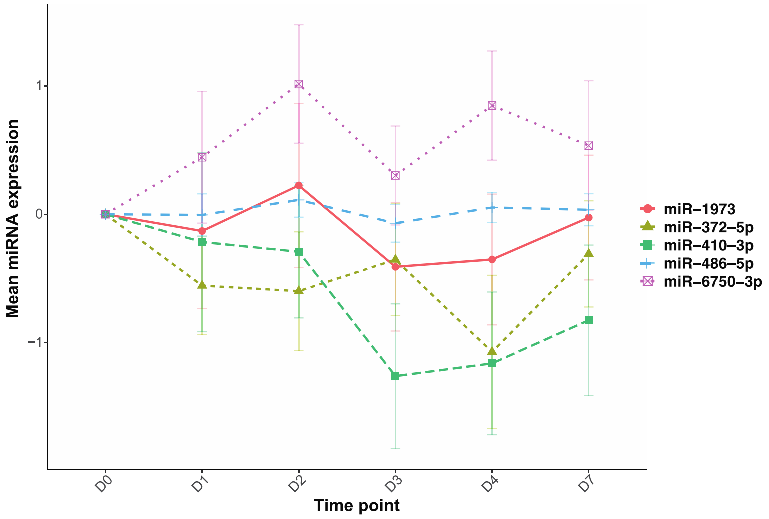

Figure S5. Expression kinetics of the top 5 WB and EV miRNAs at baseline that correlate with the ZEBOV-GP-specific IgG antibody titer, Related to Figure 5.

The Log2-transformed expression data of the top 5 miRNAs from WB and EV compartment at baseline that correlate with the ZEBOV-GP-specific IgG antibody titer in individuals that received the rVSVΔG-ZEBOV-GP vaccine were used. The line plot per each miRNA shows the mean expression over time (D0-D7). The standard error is reported as bar per each miRNA and time point. (A) WB-miRNAs. (B) EV-miRNAs.

Figure S6

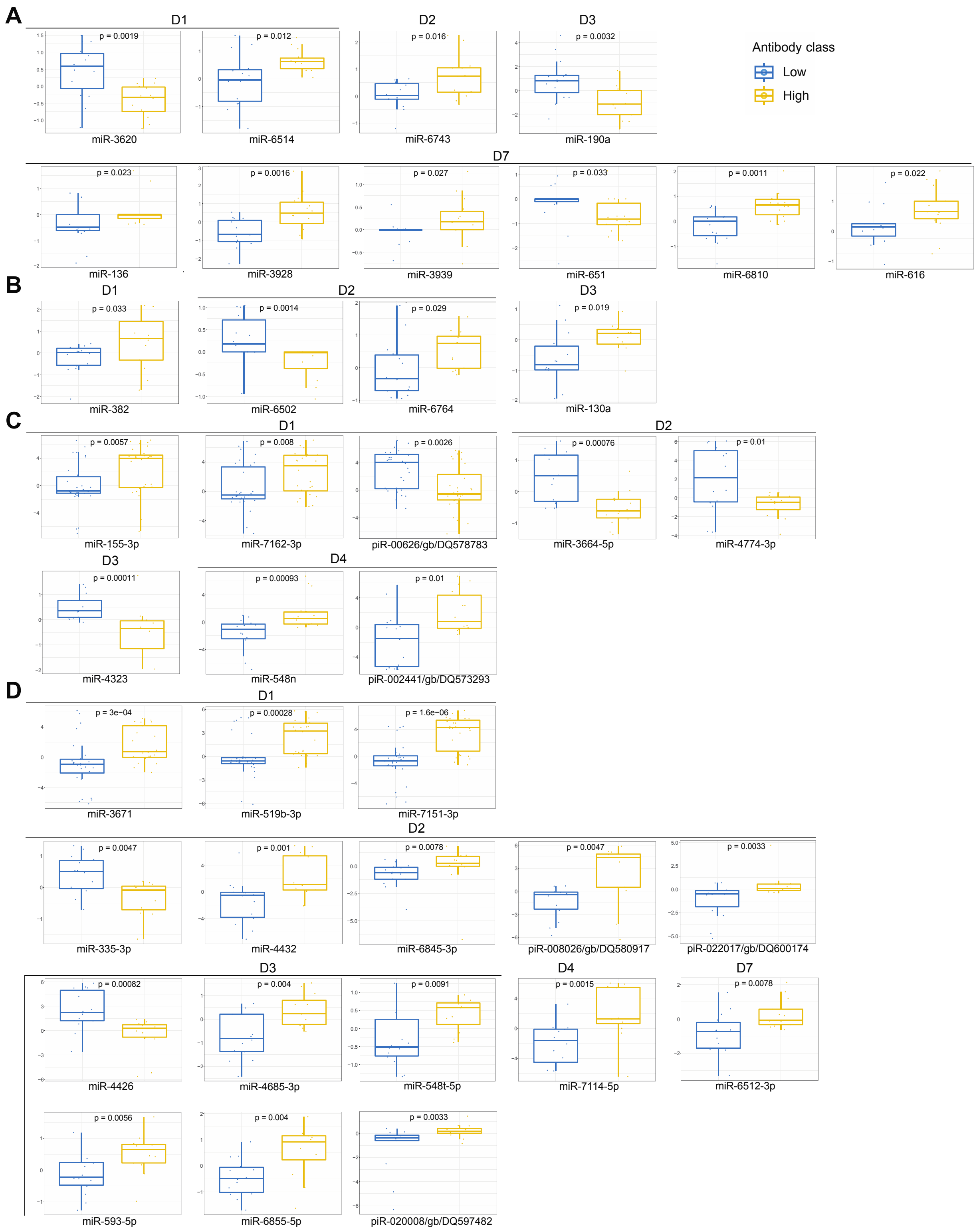

A
D1
p = 0.0019
miR-3620
p = 0.012
miR-6514
D2
p = 0.016
miR-6743
D3
p = 0.0032
miR-190a
Antibody class
Low
High
D7
p = 0.023
miR-136
p = 0.0016
miR-3928
p = 0.027
miR-3939
p = 0.033
miR-651
p = 0.0011
miR-6810
p = 0.022
miR-616
B
D1
p = 0.033
miR-382
D2
p = 0.0014
miR-6502
p = 0.029
miR-6764
D3
p = 0.019
miR-130a
C
D1
p = 0.0057
miR-155-3p
p = 0.008
miR-7162-3p
p = 0.0026
piR-00626/gb/DQ578783
D2
p = 0.00076
miR-3664-5p
p = 0.01
miR-4774-3p
D3
p = 0.00011
miR-4323
D4
p = 0.00093
miR-548n
p = 0.01
piR-002441/gb/DQ573293
D
D1
p = 3e−04
miR-3671
p = 0.00028
miR-519b-3p
p = 1.6e−06
miR-7151-3p
D2
p = 0.0047
miR-335-3p
p = 0.001
miR-4432
p = 0.0078
miR-6845-3p
p = 0.0047
piR-008026/gb/DQ580917
p = 0.0033
piR-022017/gb/DQ600174
D3
p = 0.00082
miR-4426
p = 0.004
miR-4685-3p
p = 0.0091
miR-548t-5p
D4
p = 0.0015
miR-7114-5p
D7
p = 0.0078
miR-6512-3p
p = 0.0056
miR-593-5p
p = 0.004
miR-6855-5p
p = 0.0033
piR-020008/gb/DQ597482

Figure S6. Most representative miRNAs at sampling time points that predict high and low antibody levels at D28 and D360, Related to Figure 6.

The most representative miRNAs have been selected considering Variable Importance in Projection (VIP) > 20% and in which the difference between the low and high antibody classes was significant with p-value < 0.05 or p < 0.01 for WB-miRNAs and EV-miRNAs, respectively. The y-axis represents the baseline-adjusted Log2-transformed miRNA expression. Box-and-whiskers plots (5-95 percentiles) display the results for (A) WB – antibody D28. (B) WB – antibody D360. (C) EV – antibody D28. (D) EV – antibody D360.

Figure S7

A Kinetics of the top 5 WB-miRNAs that predict antibody levels

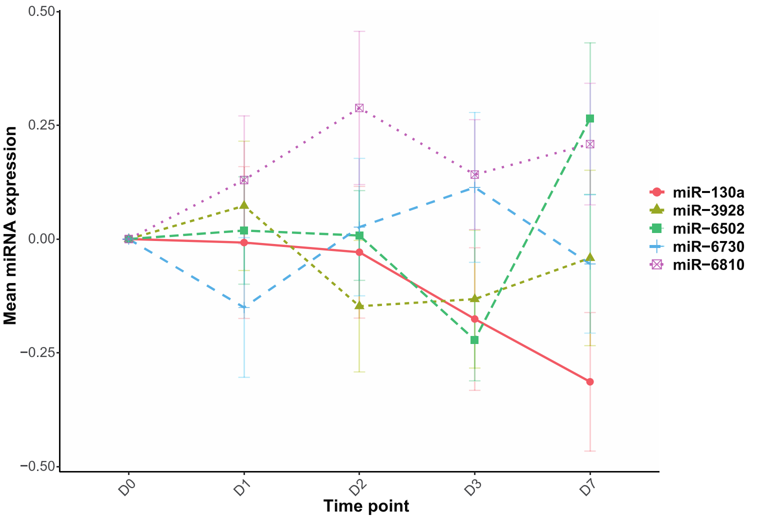

B Kinetics of the top 5 EV-miRNAs that predict antibody levels

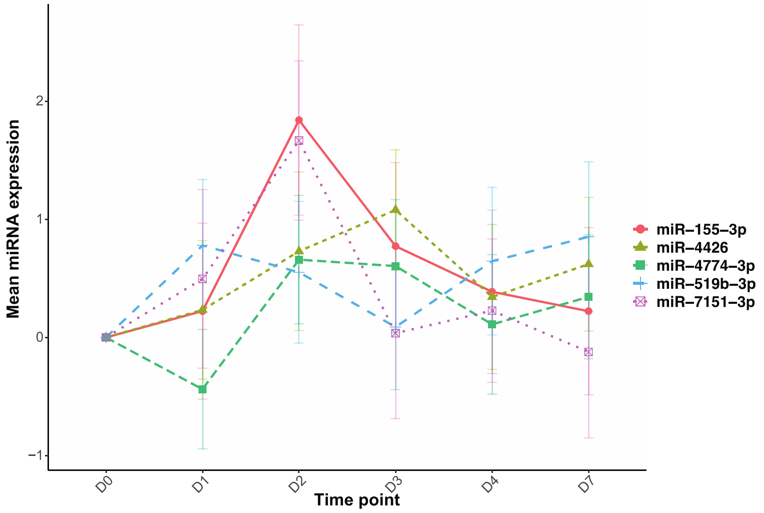

Figure S7. Expression kinetics of the top 5 WB and EV miRNAs that predict high and low ZEBOV-GP-specific IgG antibody levels, Related to Figure 6 and 7.

The Log2-transformed and baseline adjusted expression data of the top 5 miRNAs from WB and EV compartment that predict the ZEBOV-GP-specific IgG antibody levels in individuals that received the rVSVΔG-ZEBOV-GP vaccine were used. The line plot per each miRNA shows the mean expression over time (D0-D7). The standard error is reported as bar per each miRNA and time point. (A) WB-miRNAs. (B) EV-miRNAs.

Figure S8

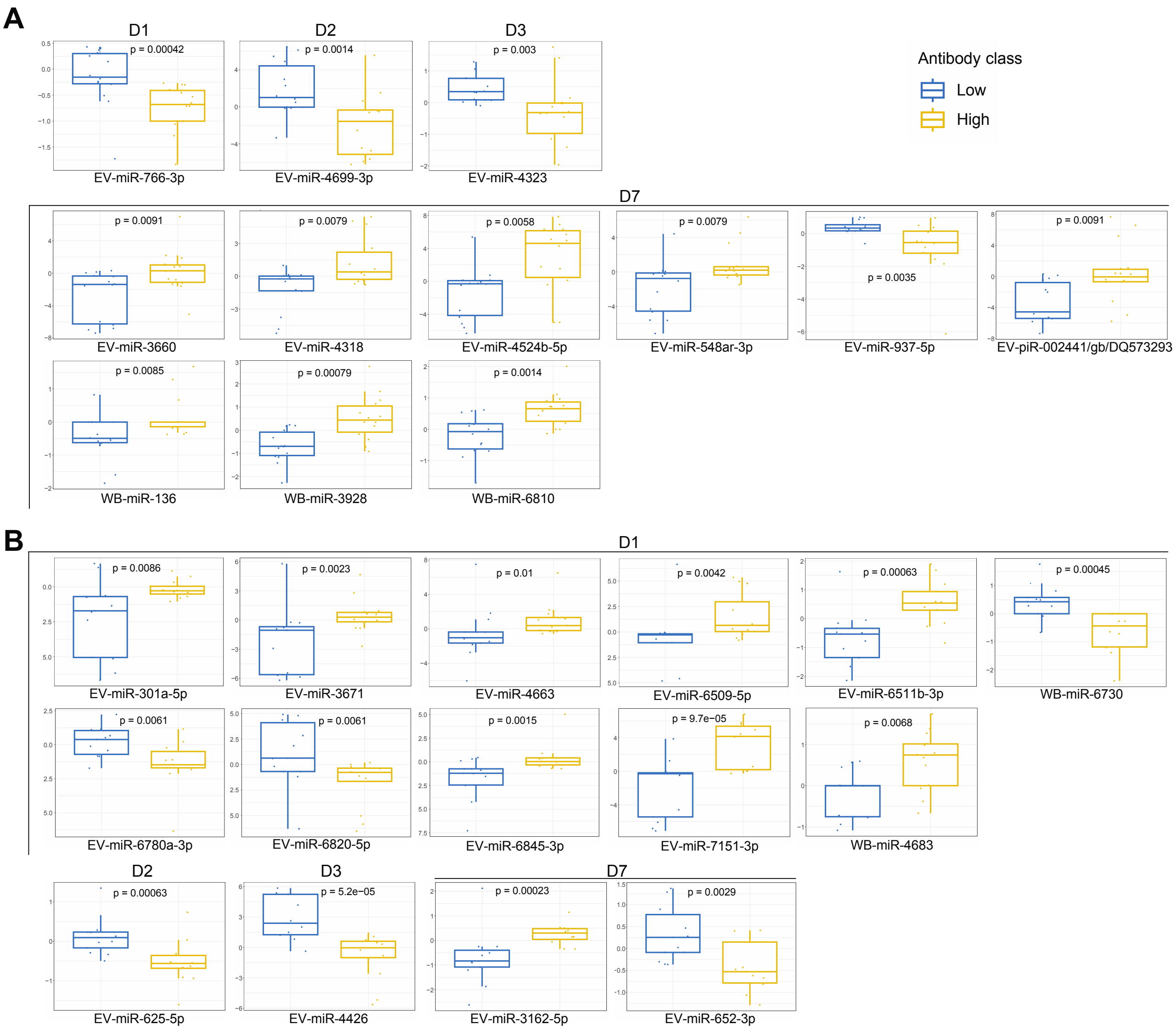

Figure S8. Most representative miRNAs of the combined WB+EV miRNA signatures at sampling time points that predict high and low antibody levels at D28 and D360, Related to Figure 7.

The most representative miRNAs have been selected considering Variable Importance in Projection (VIP) > 20% and in which the difference between the low and high antibody classes was significant with $p < 0.01$. The y-axis represents the baseline-adjusted Log2-transformed miRNA expression. Box-and-whiskers plots (5-95 percentiles) display the results for (A) Antibody D28. (B) Antibody D360.

Data S1

Data S1. Expression kinetics of the full list of the differentially expressed WB-miRNAs, Related to Figure 2 and 3.

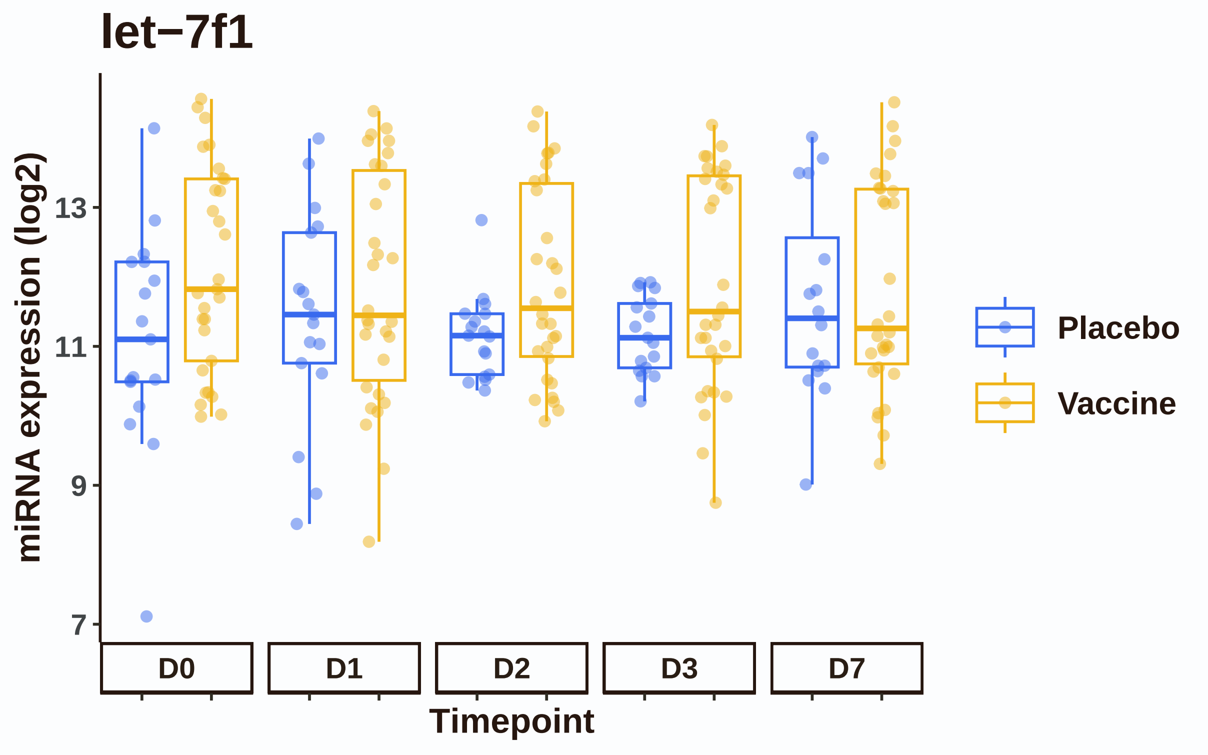
let−7f1
miRNA expression (log2)
13
11
9
7
D0
D1
D2
D3
D7
Timepoint
Placebo
Vaccine

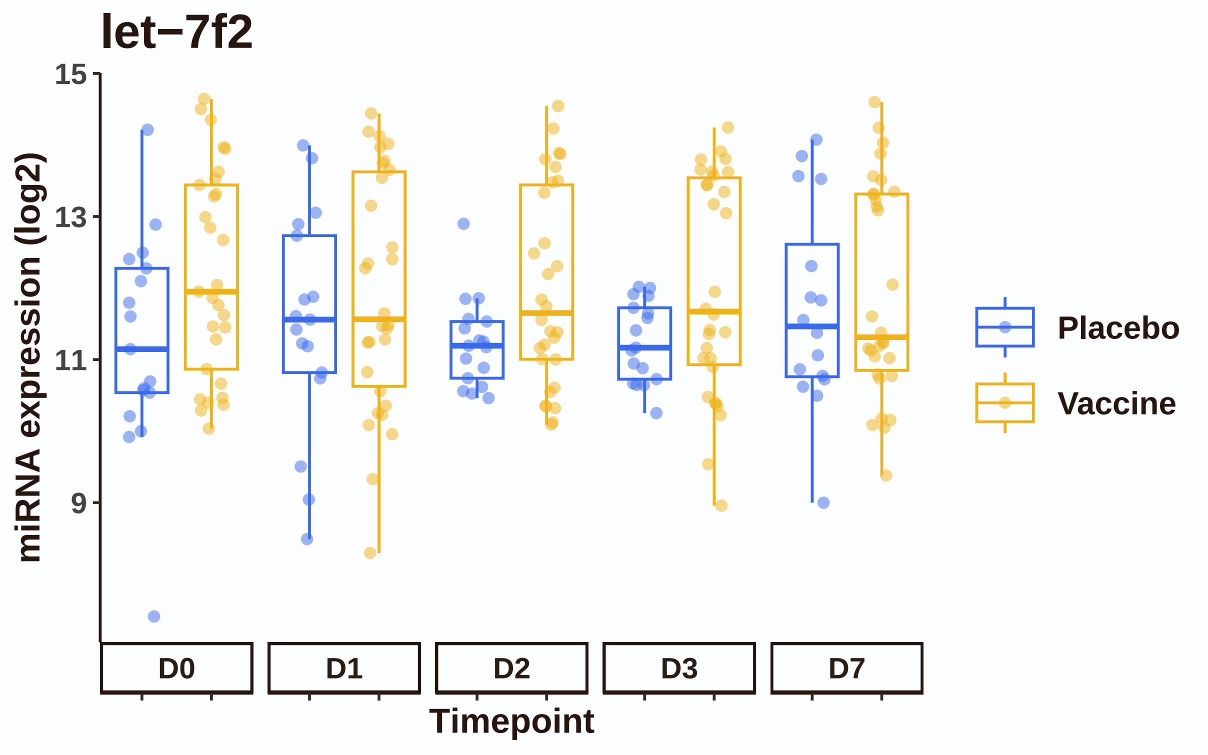

let−7f2
15
13
11
9
miRNA expression (log2)
D0
D1
D2
D3
D7
Timepoint
Placebo
Vaccine

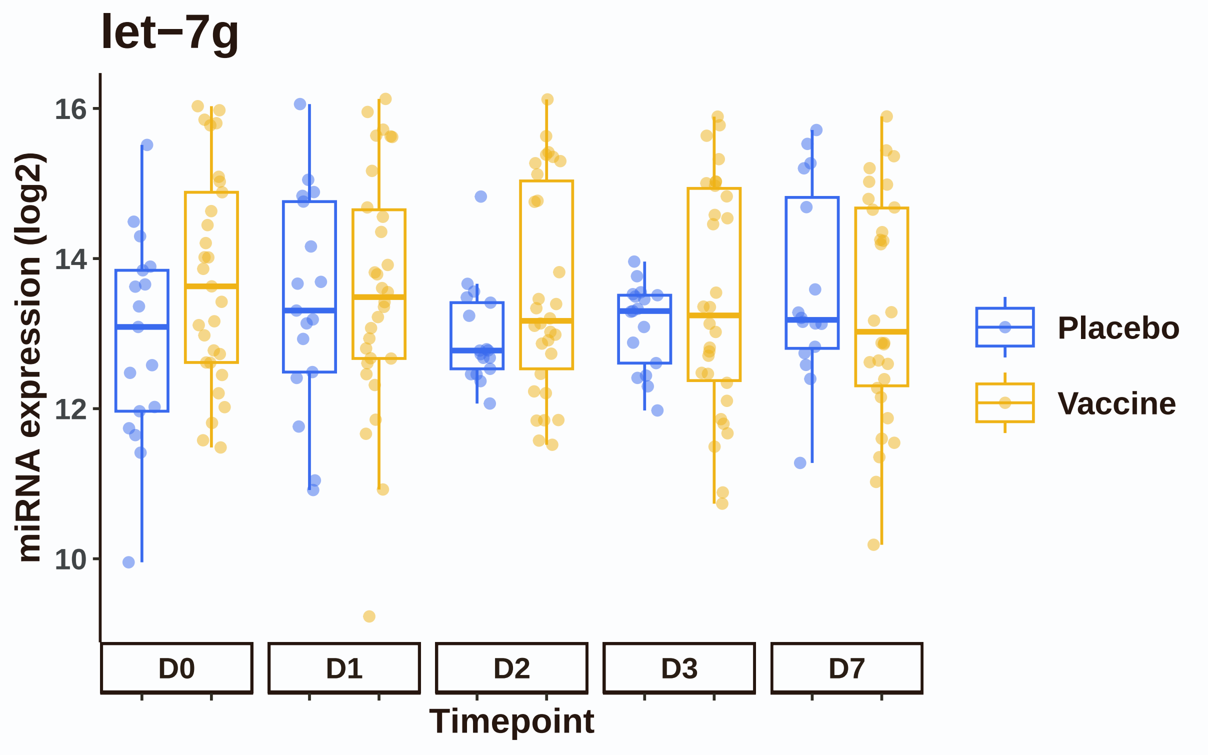

let−7g
miRNA expression (log2)
16
14
12
10
D0
D1
D2
D3
D7
Timepoint
Placebo
Vaccine

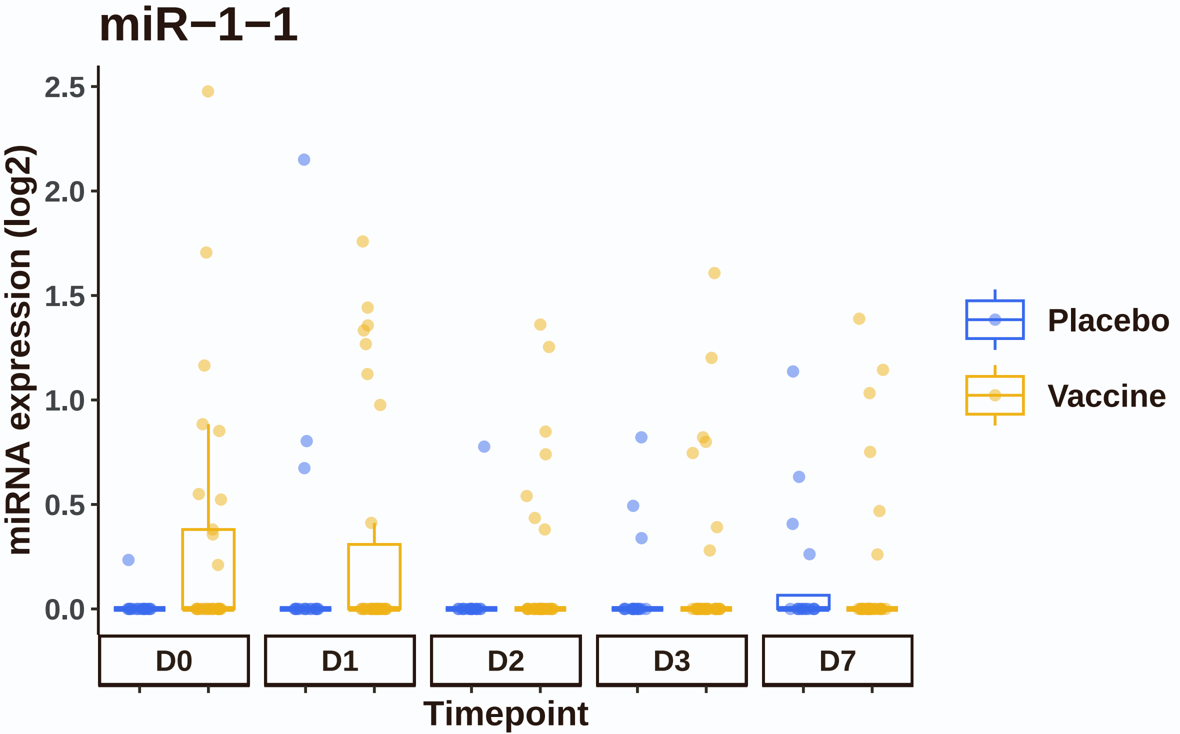

miR−1−1
miRNA expression (log2)
2.5
2.0
1.5
1.0
0.5
0.0
D0
D1
D2
D3
D7
Timepoint
Placebo
Vaccine

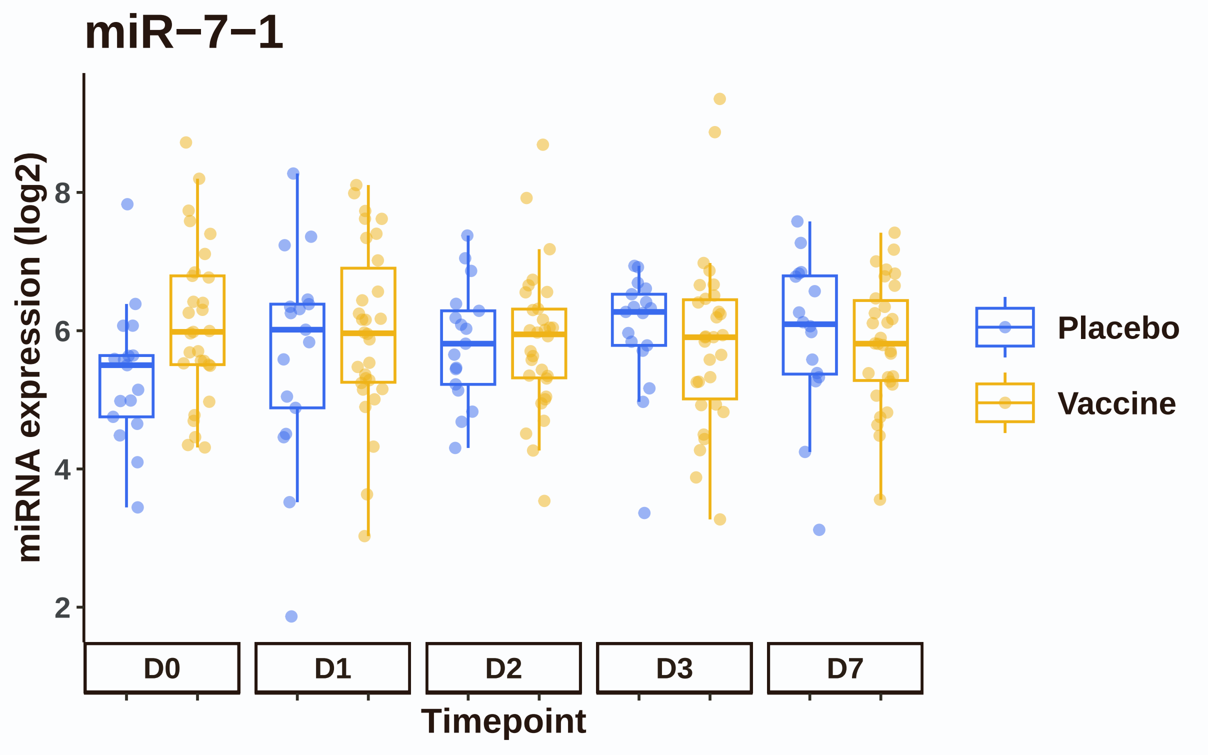
miR−7−1
miRNA expression (log2)
8
6
4
2
D0
D1
D2
D3
D7
Timepoint
Placebo
Vaccine

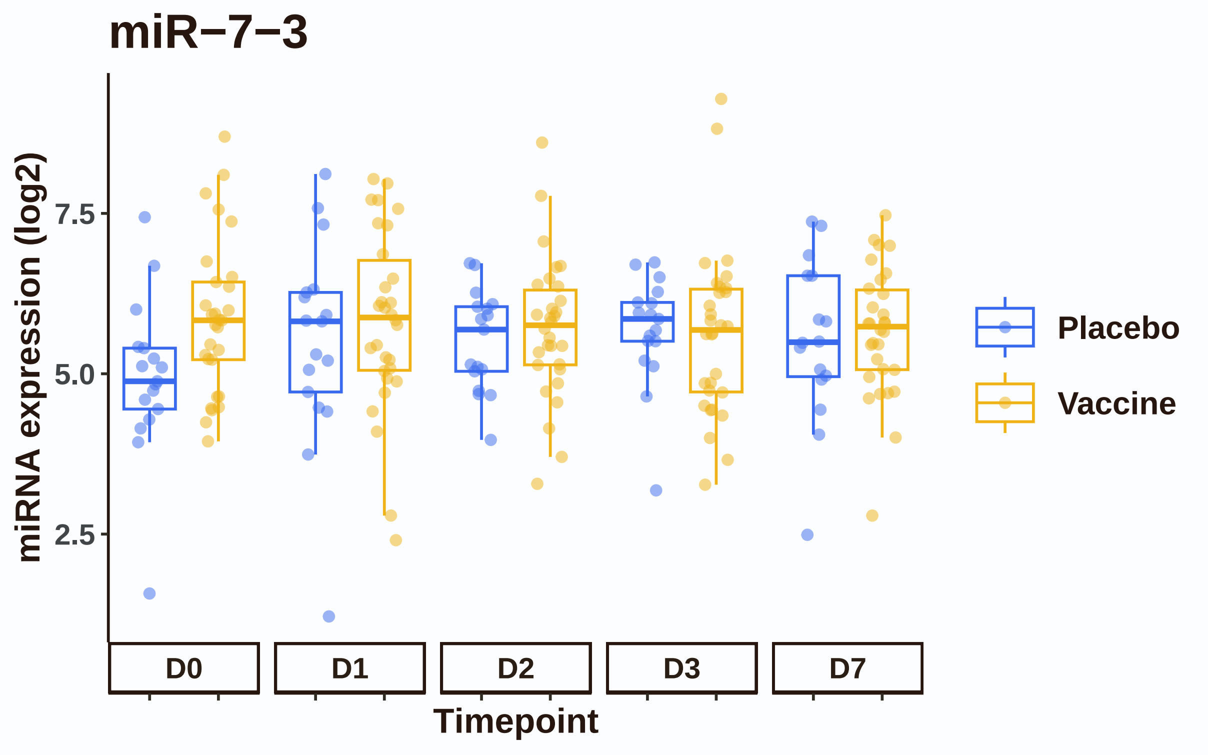
miR−7−3
miRNA expression (log2)
7.5
5.0
2.5
D0
D1
D2
D3
D7
Timepoint
Placebo
Vaccine

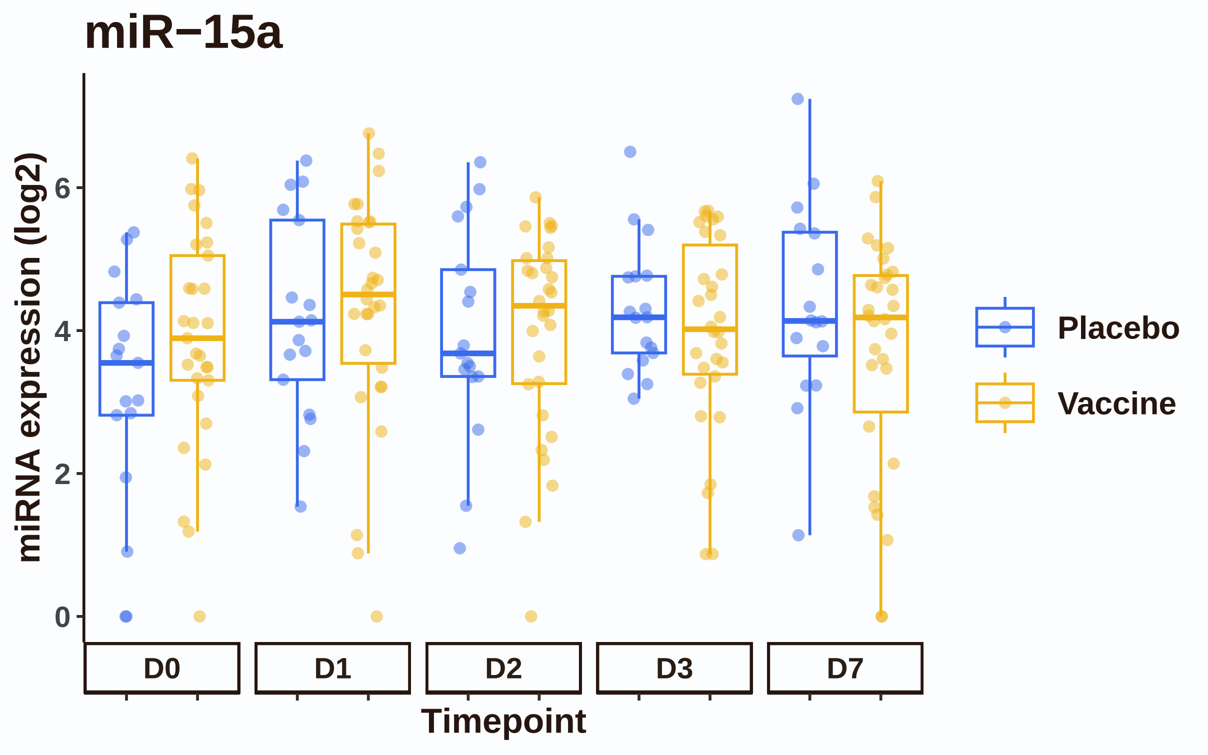

miR−15a
miRNA expression (log2)
0
2
4
6
D0
D1
D2
D3
D7
Timepoint
Placebo
Vaccine

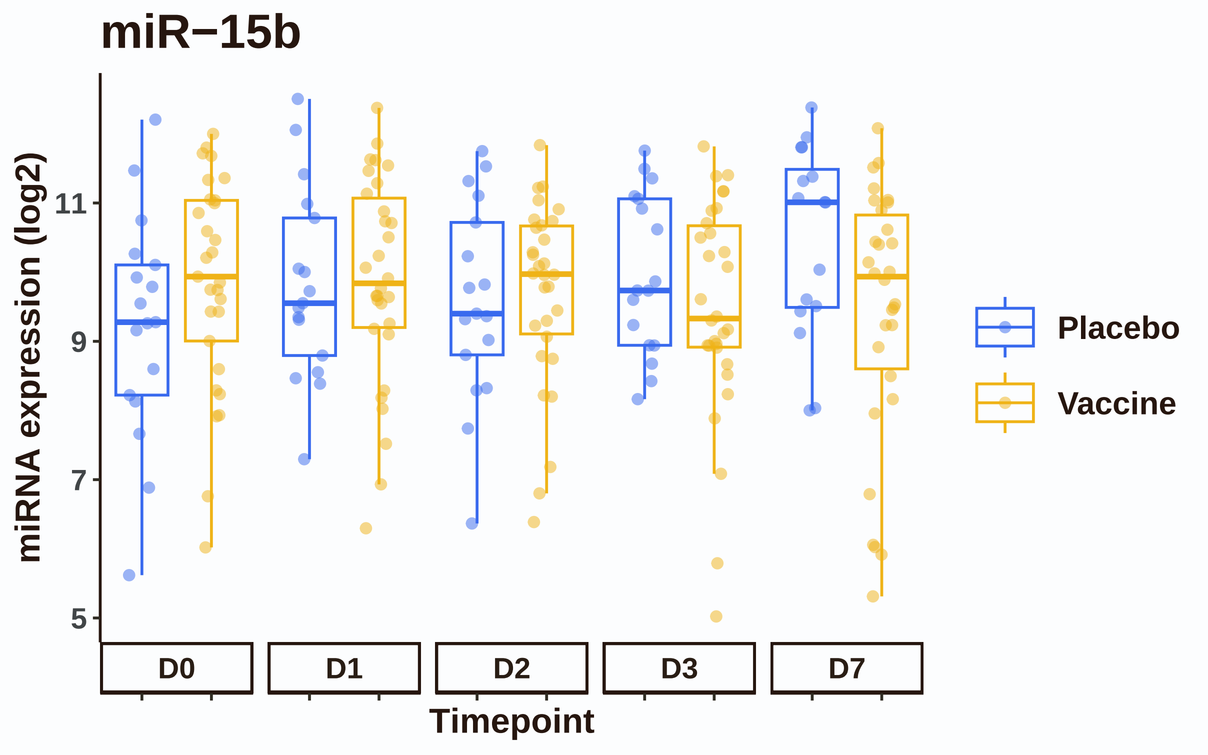
miR−15b
miRNA expression (log2)
11
9
7
5
D0
D1
D2
D3
D7
Timepoint
Placebo
Vaccine

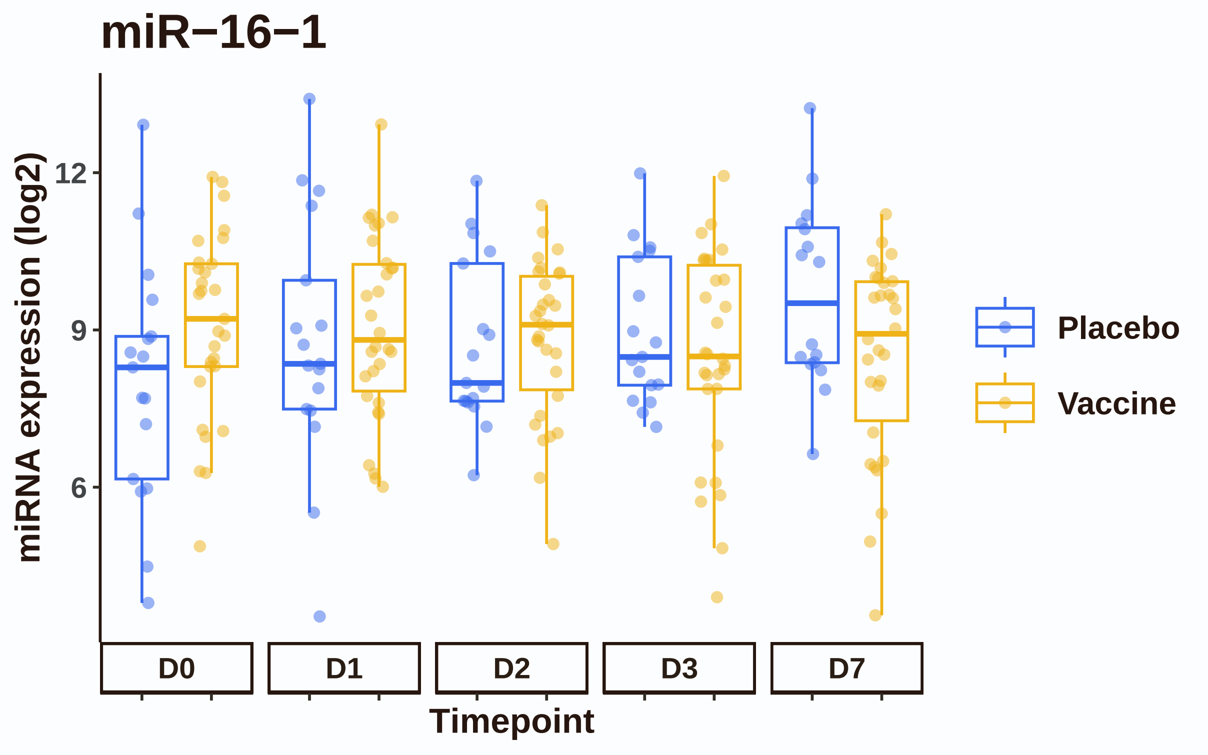

miR−16−1
miRNA expression (log2)
12
9
6
D0
D1
D2
D3
D7
Timepoint
Placebo
Vaccine

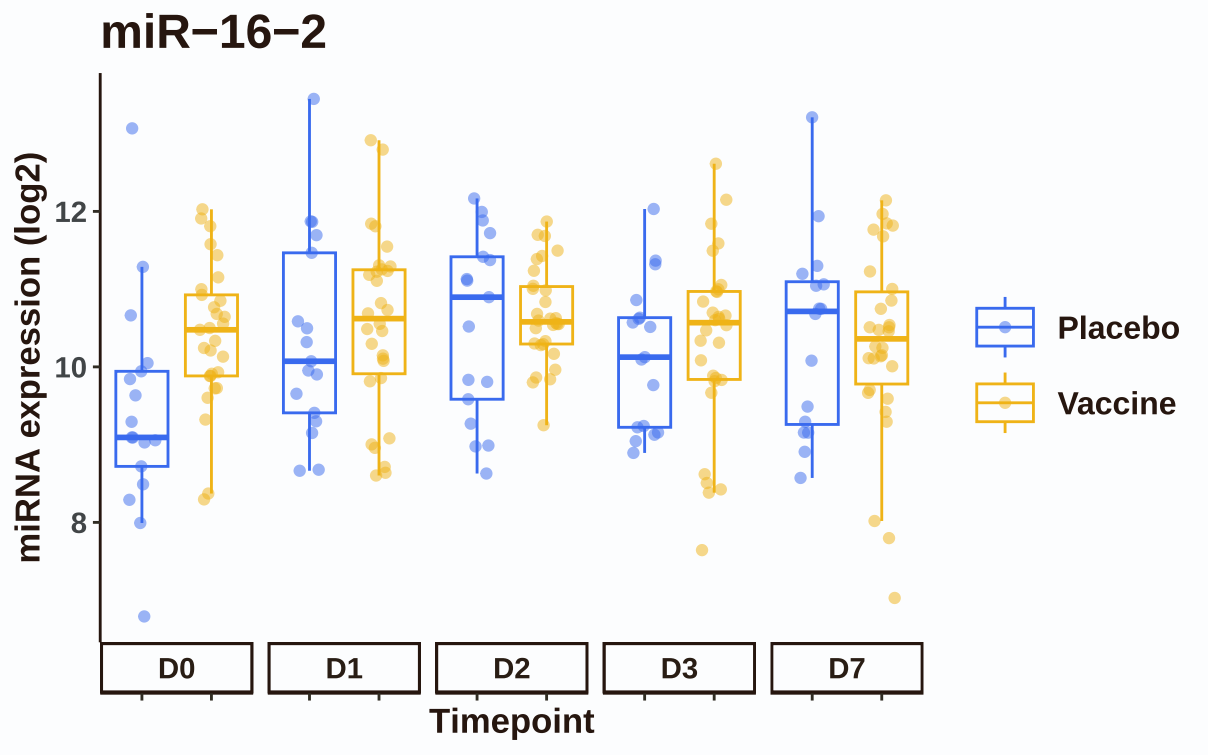
miR−16−2
miRNA expression (log2)
12
10
8
D0
D1
D2
D3
D7
Timepoint
Placebo
Vaccine

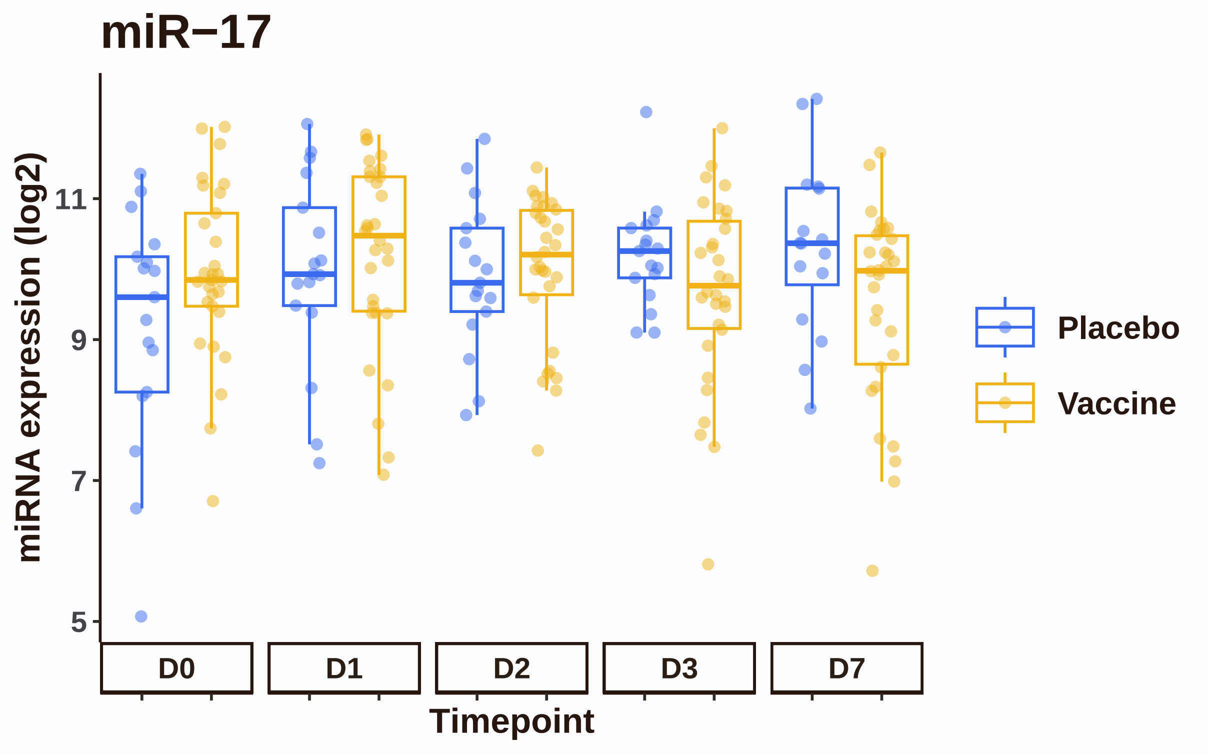

miR−17
miRNA expression (log2)
11
9
7
5
D0
D1
D2
D3
D7
Timepoint
Placebo
Vaccine

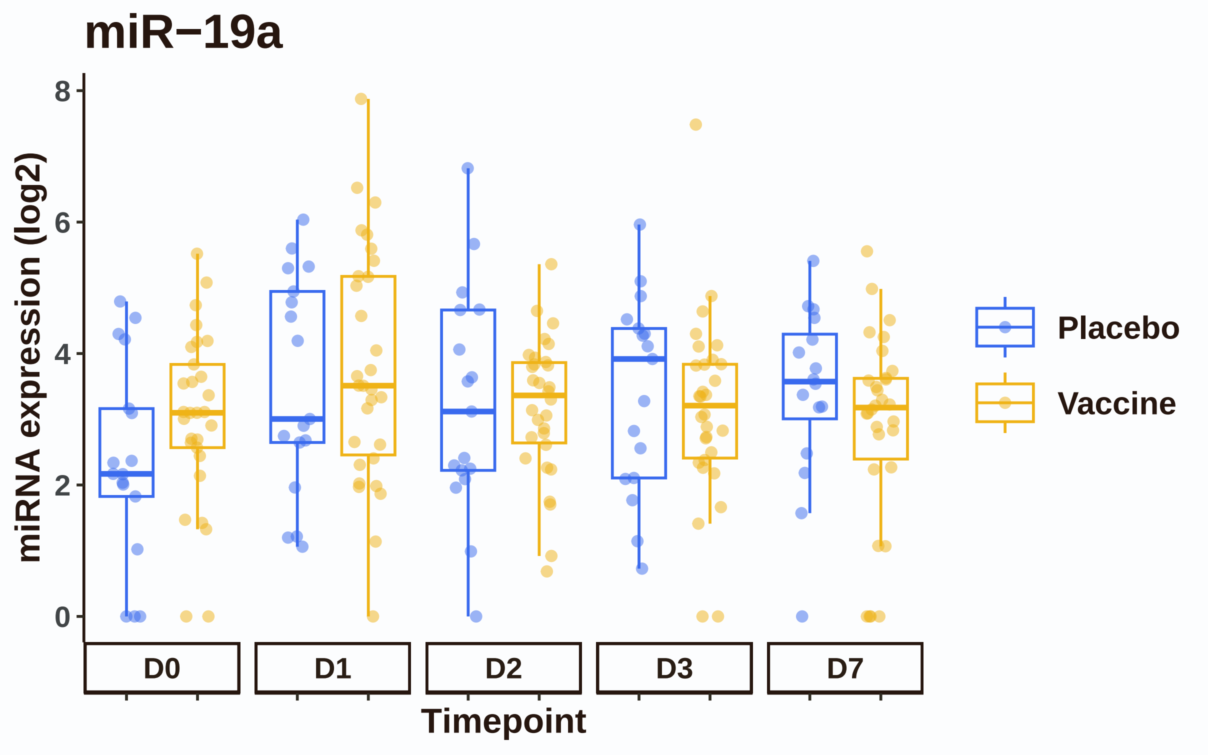

miR−19a
miRNA expression (log2)
0
2
4
6
8
D0
D1
D2
D3
D7
Timepoint
Placebo
Vaccine

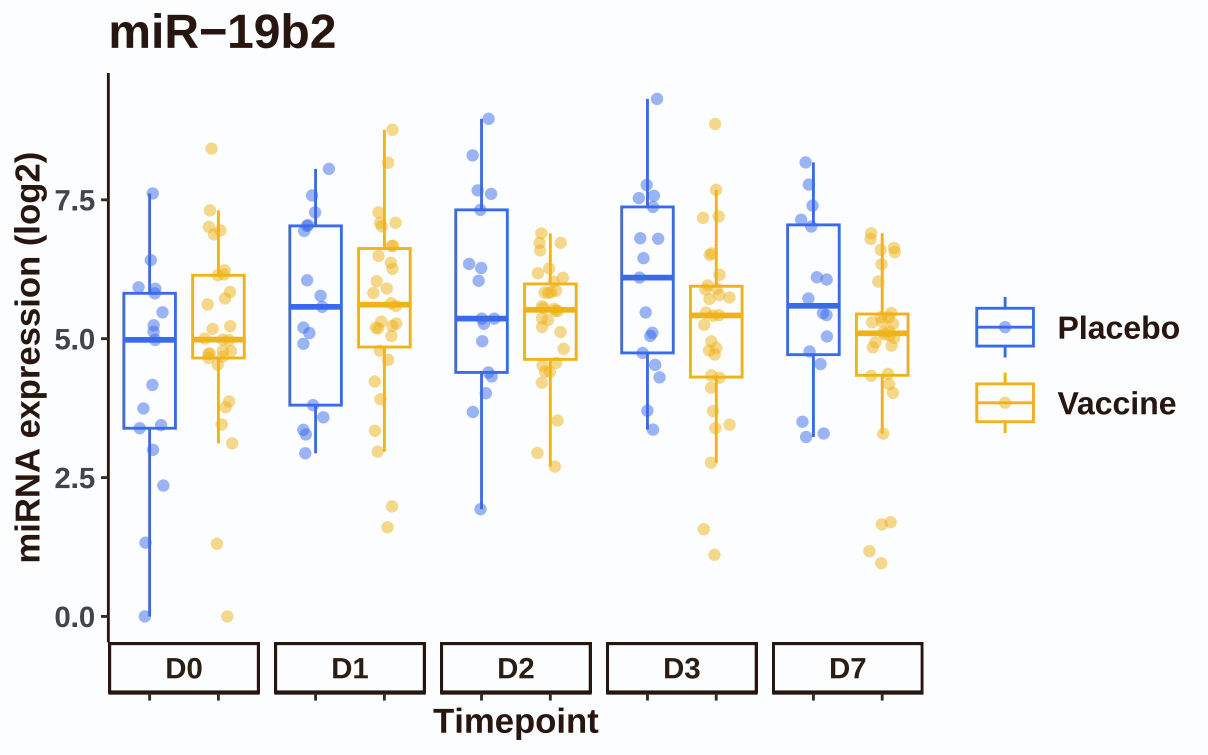

miR−19b2
miRNA expression (log2)
0.0
2.5
5.0
7.5
D0
D1
D2
D3
D7
Timepoint
Placebo
Vaccine

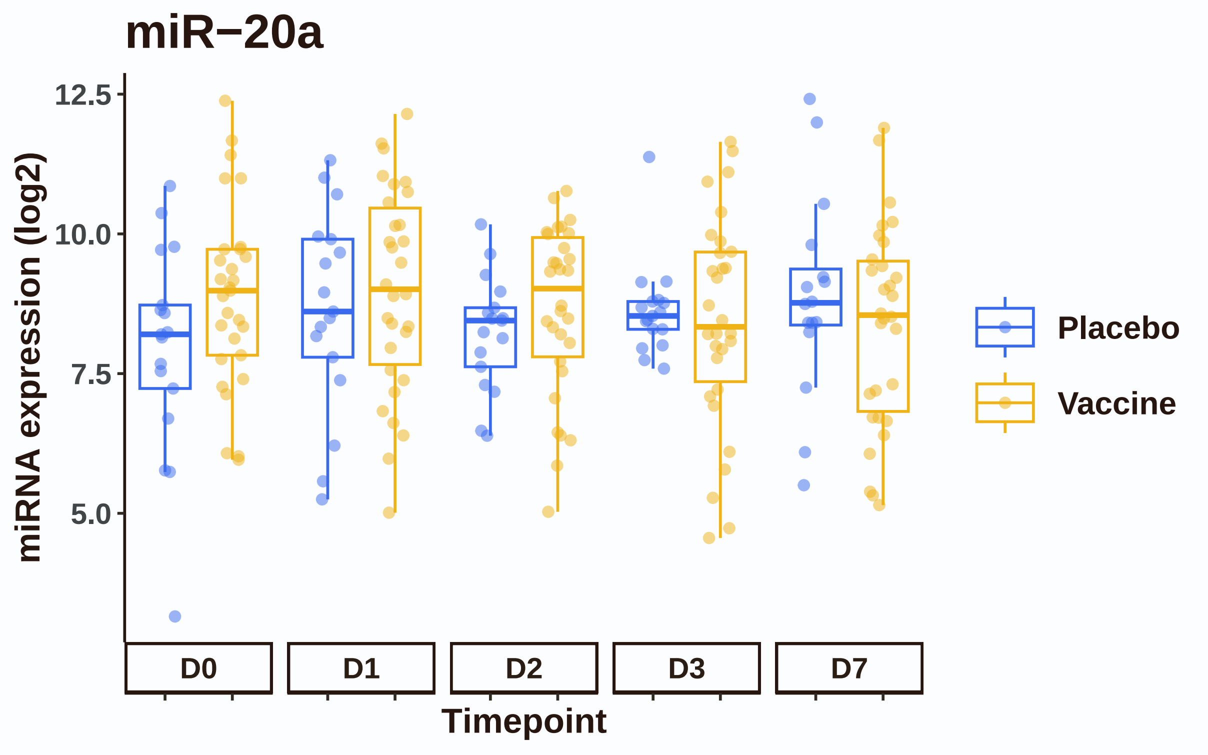
miR−20a
miRNA expression (log2)
12.5
10.0
7.5
5.0
D0
D1
D2
D3
D7
Timepoint
Placebo
Vaccine

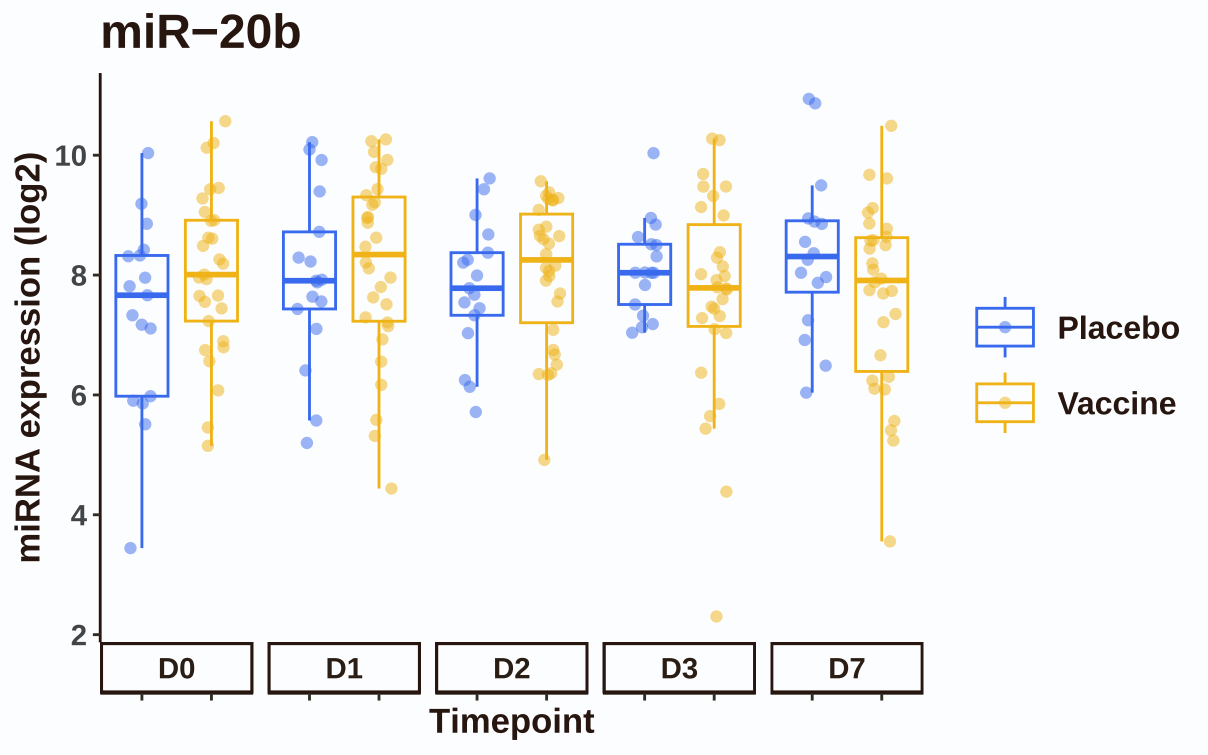
miR−20b
miRNA expression (log2)
10
8
6
4
2
D0
D1
D2
D3
D7
Timepoint
Placebo
Vaccine

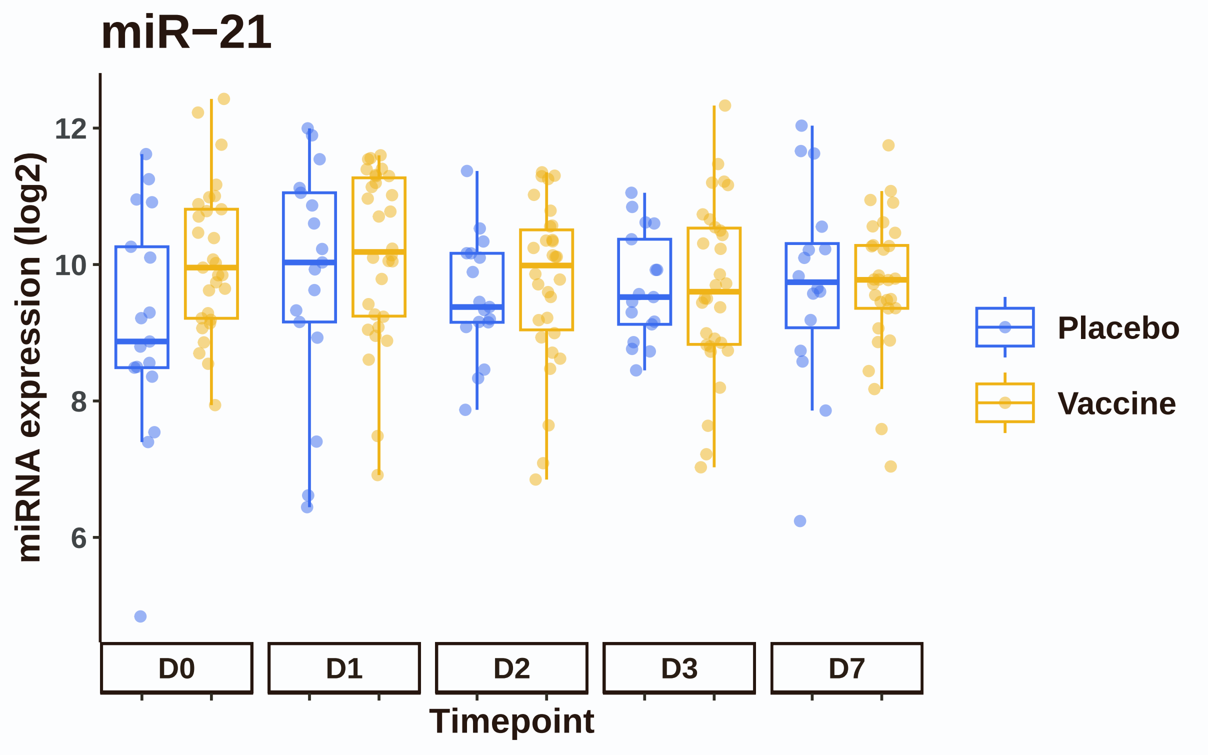

miR−21
miRNA expression (log2)
12
10
8
6
D0
D1
D2
D3
D7
Timepoint
Placebo
Vaccine

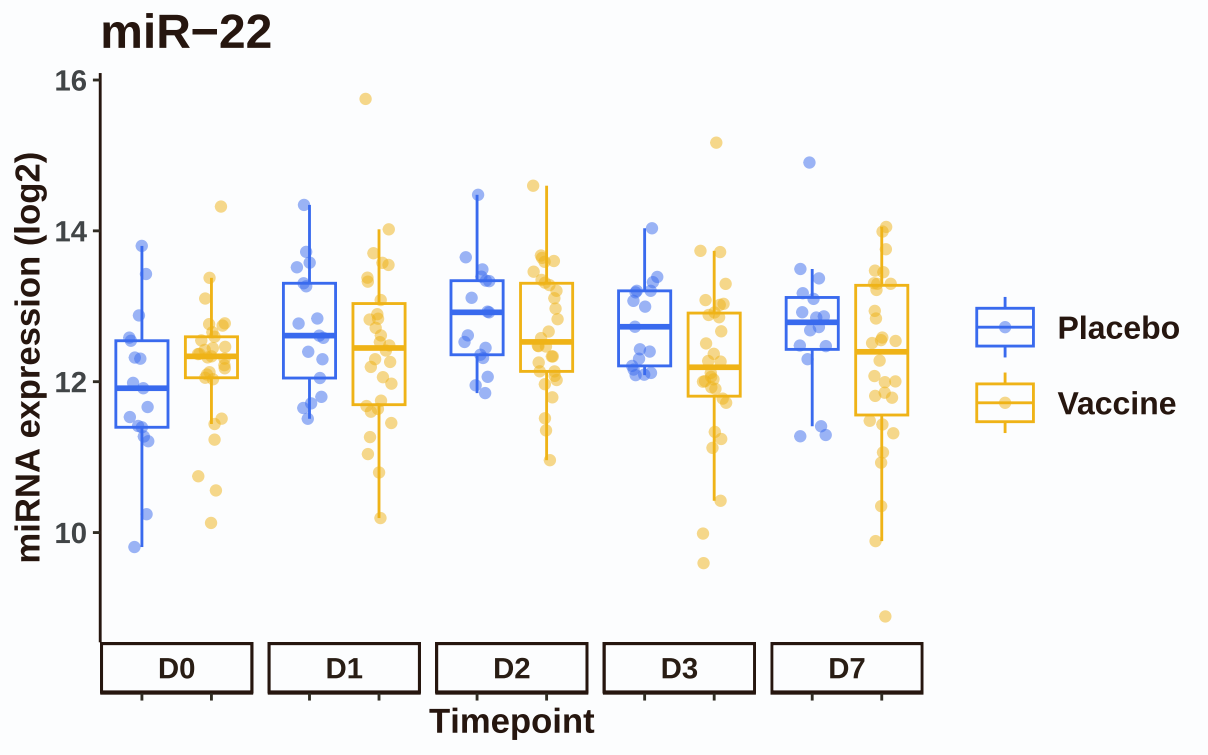

miR−22
miRNA expression (log2)
16
14
12
10
D0
D1
D2
D3
D7
Timepoint
Placebo
Vaccine

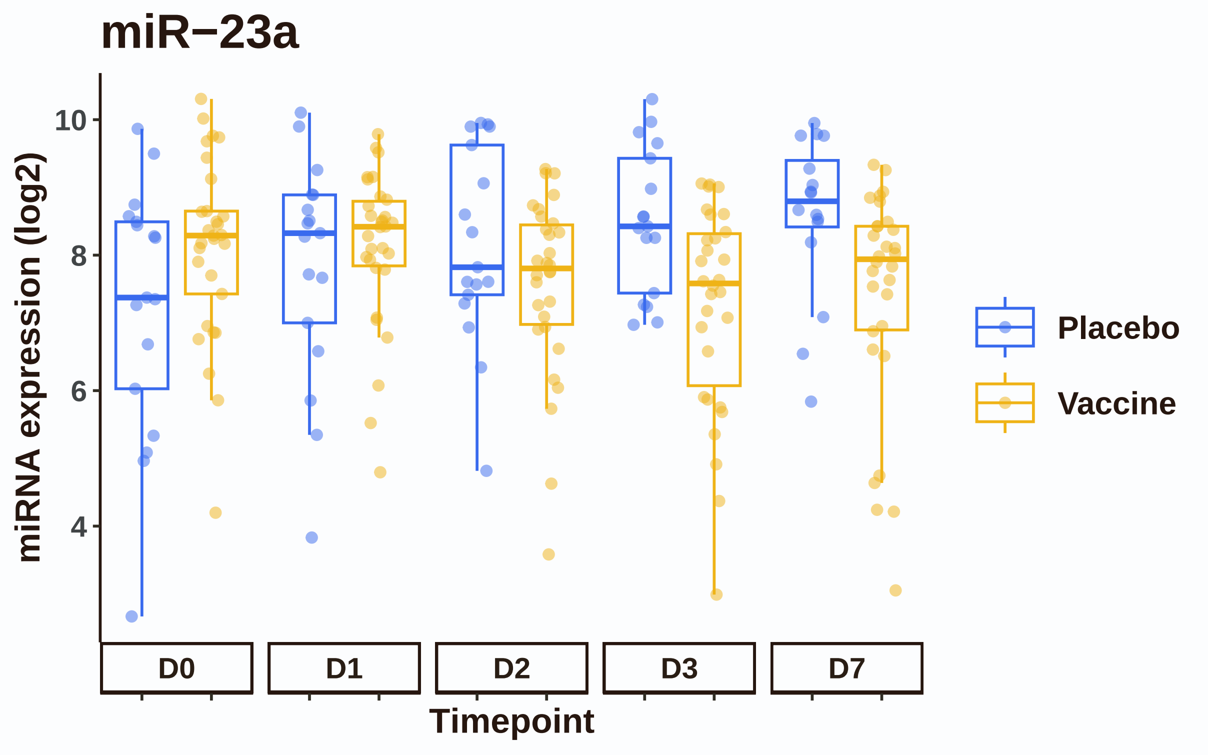

miR−23a
miRNA expression (log2)
10
8
6
4
D0
D1
D2
D3
D7
Timepoint
Placebo
Vaccine

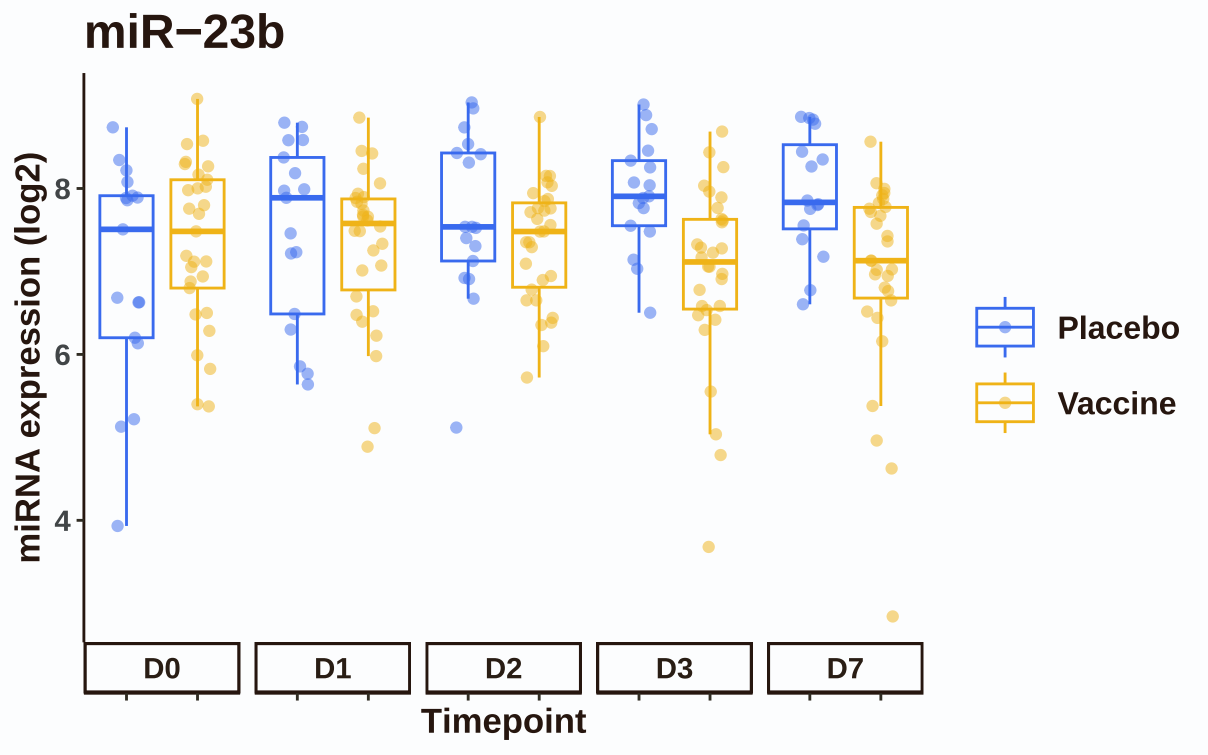
miR−23b
miRNA expression (log2)
8
6
4
D0
D1
D2
D3
D7
Timepoint
Placebo
Vaccine

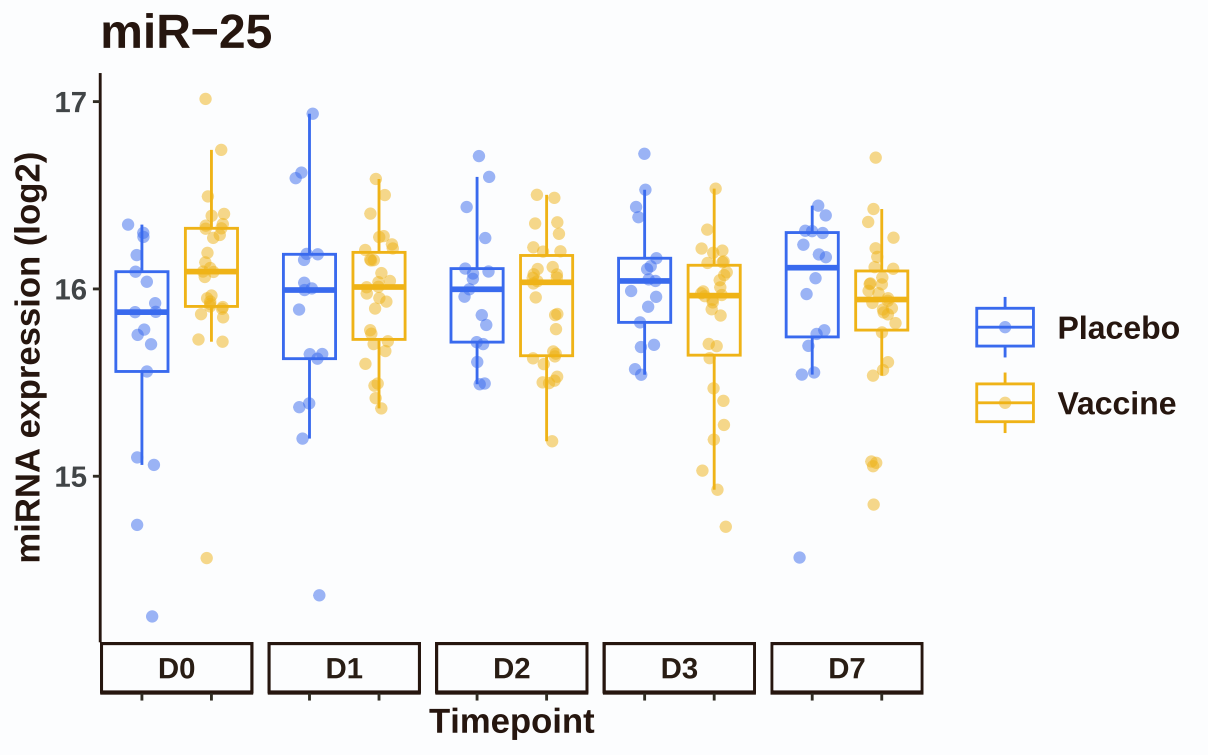
miR−25
miRNA expression (log2)
17
16
15
D0
D1
D2
D3
D7
Timepoint
Placebo
Vaccine

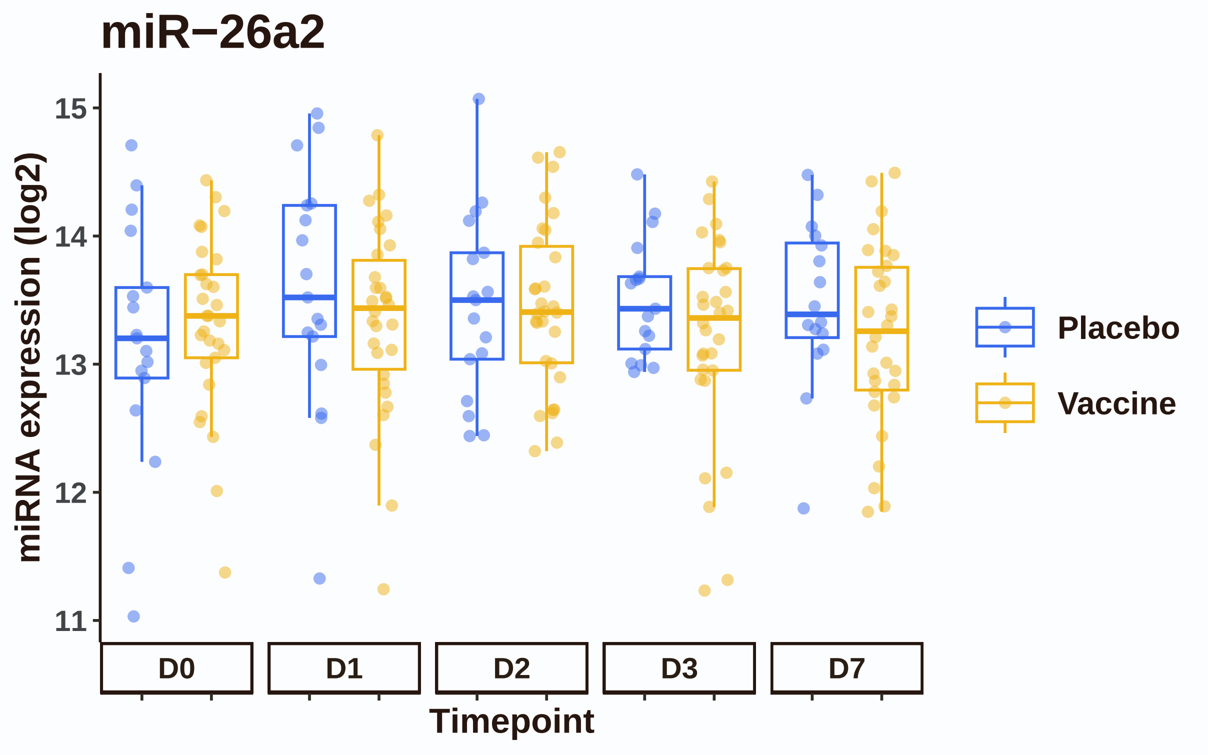

miR−26a2
miRNA expression (log2)
15
14
13
12
11
D0
D1
D2
D3
D7
Timepoint
Placebo
Vaccine

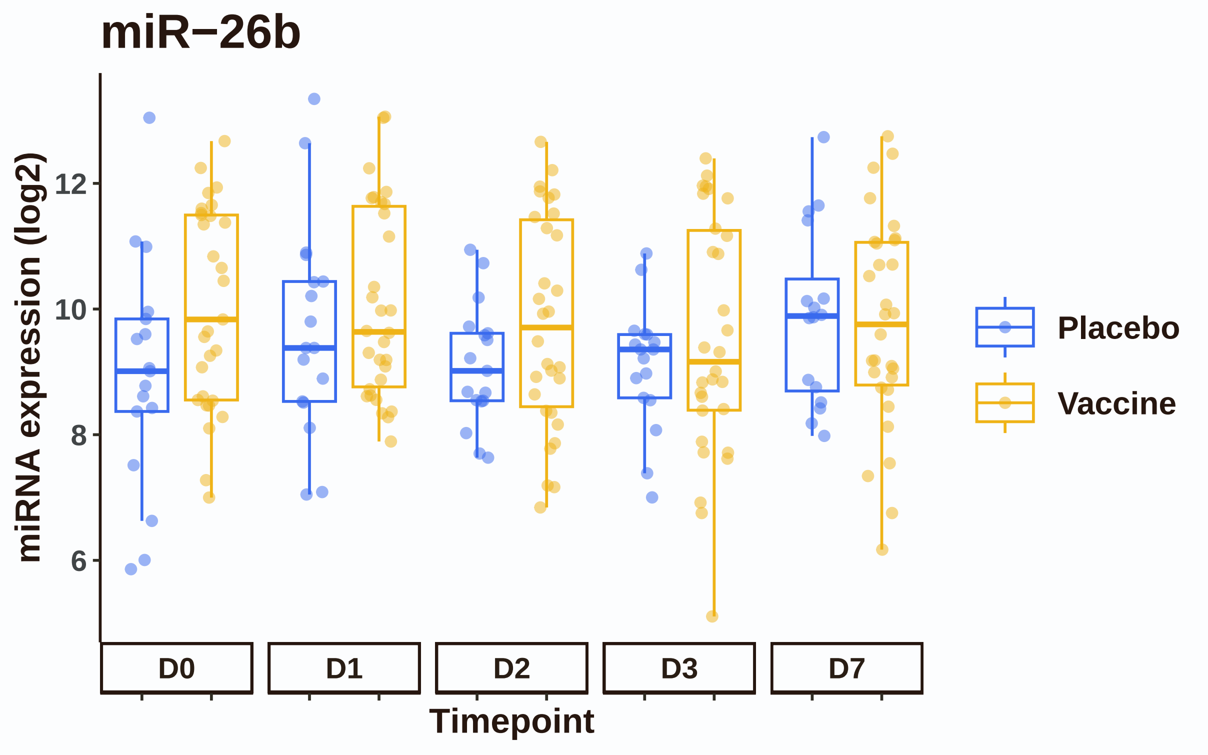
miR−26b
miRNA expression (log2)
12
10
8
6
D0
D1
D2
D3
D7
Timepoint
Placebo
Vaccine

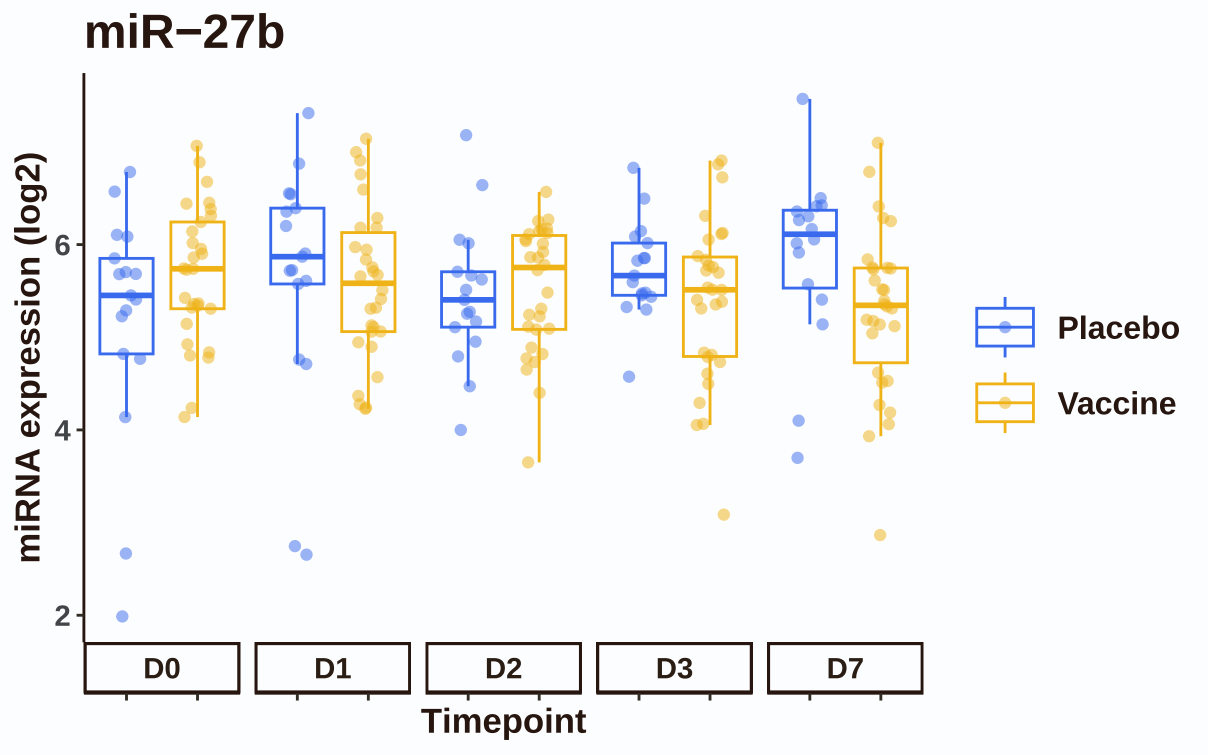

miR−27b
miRNA expression (log2)
6
4
2
D0
D1
D2
D3
D7
Timepoint
Placebo
Vaccine

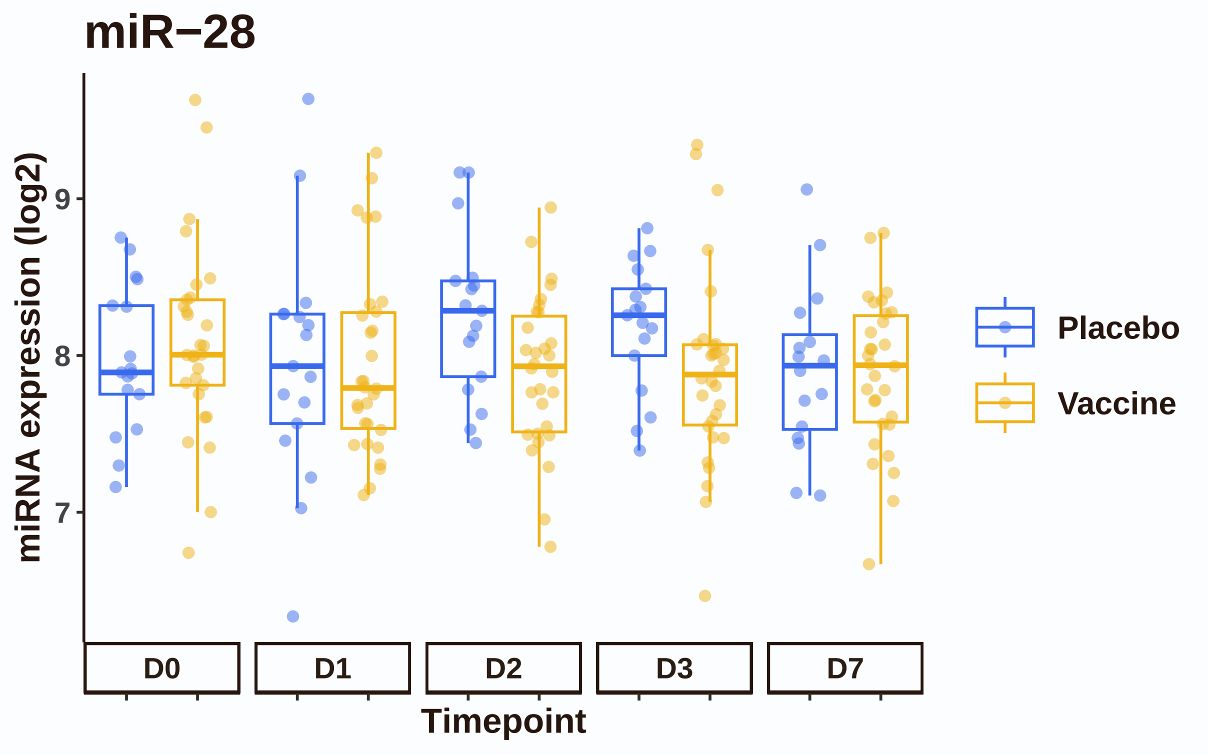
miR−28
miRNA expression (log2)
9
8
7
D0
D1
D2
D3
D7
Timepoint
Placebo
Vaccine

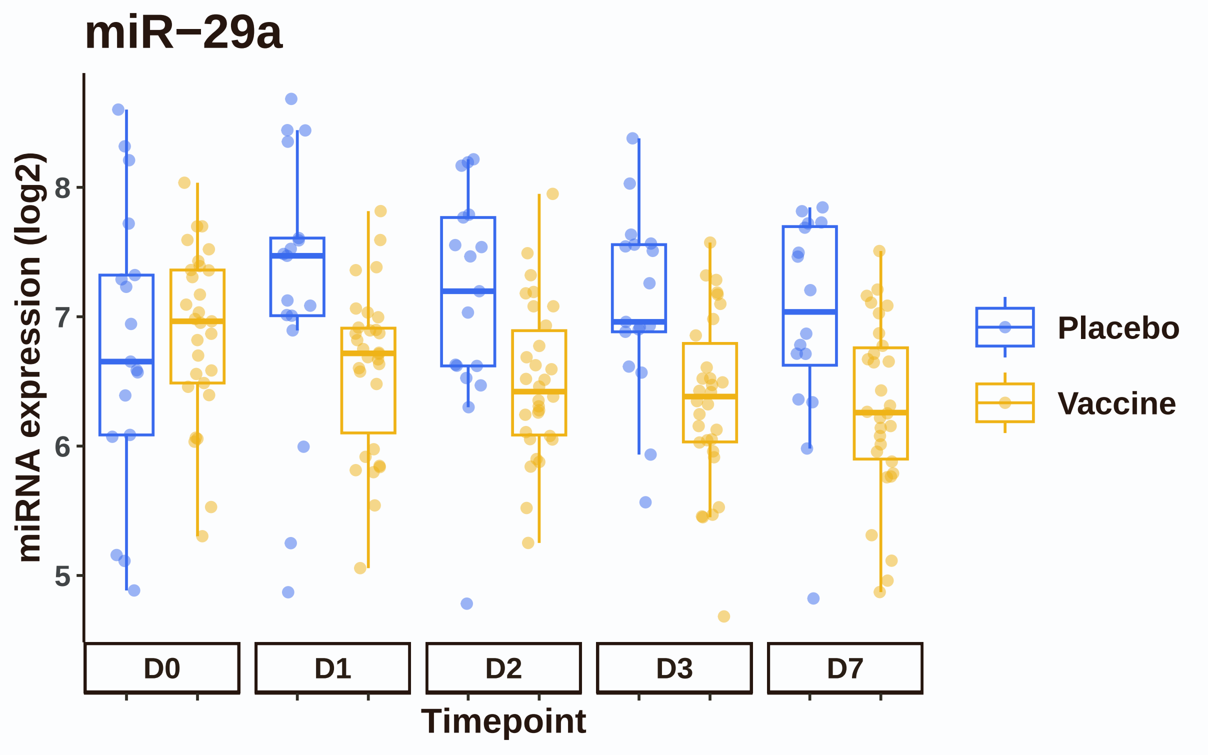
miR−29a
miRNA expression (log2)
8
7
6
5
D0
D1
D2
D3
D7
Timepoint
Placebo
Vaccine

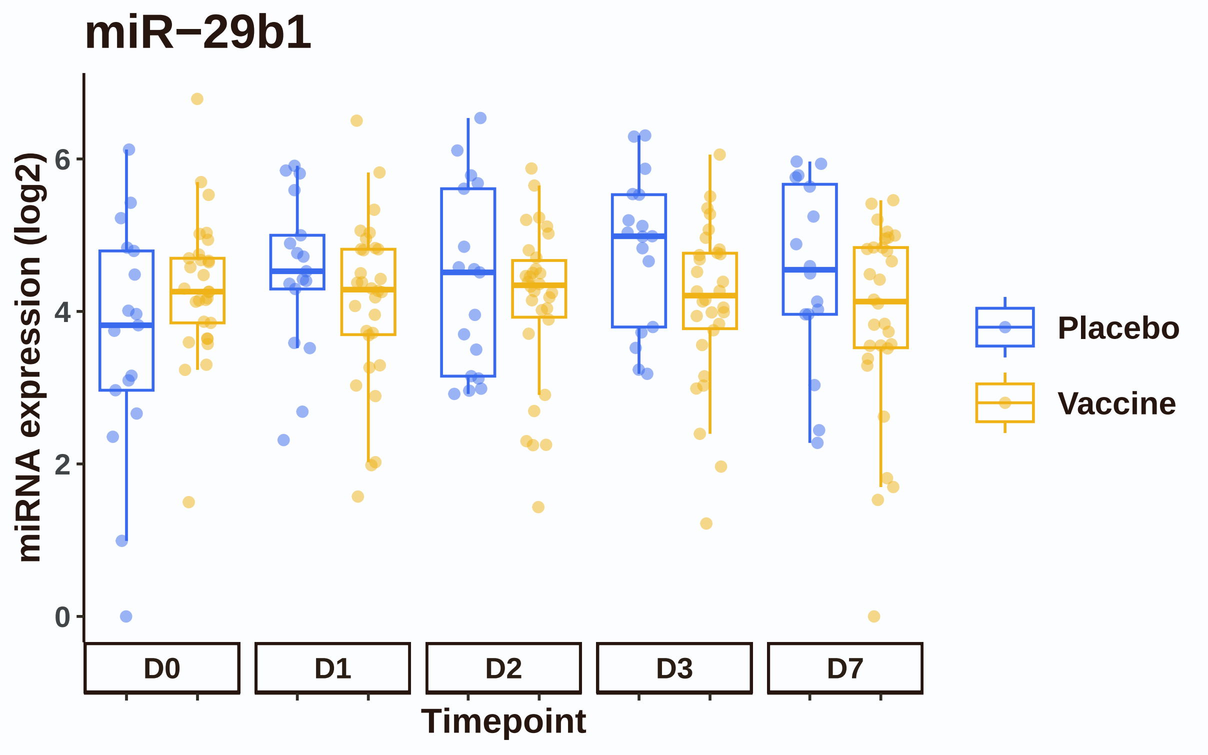
miR−29b1
miRNA expression (log2)
0
2
4
6
D0
D1
D2
D3
D7
Timepoint
Placebo
Vaccine

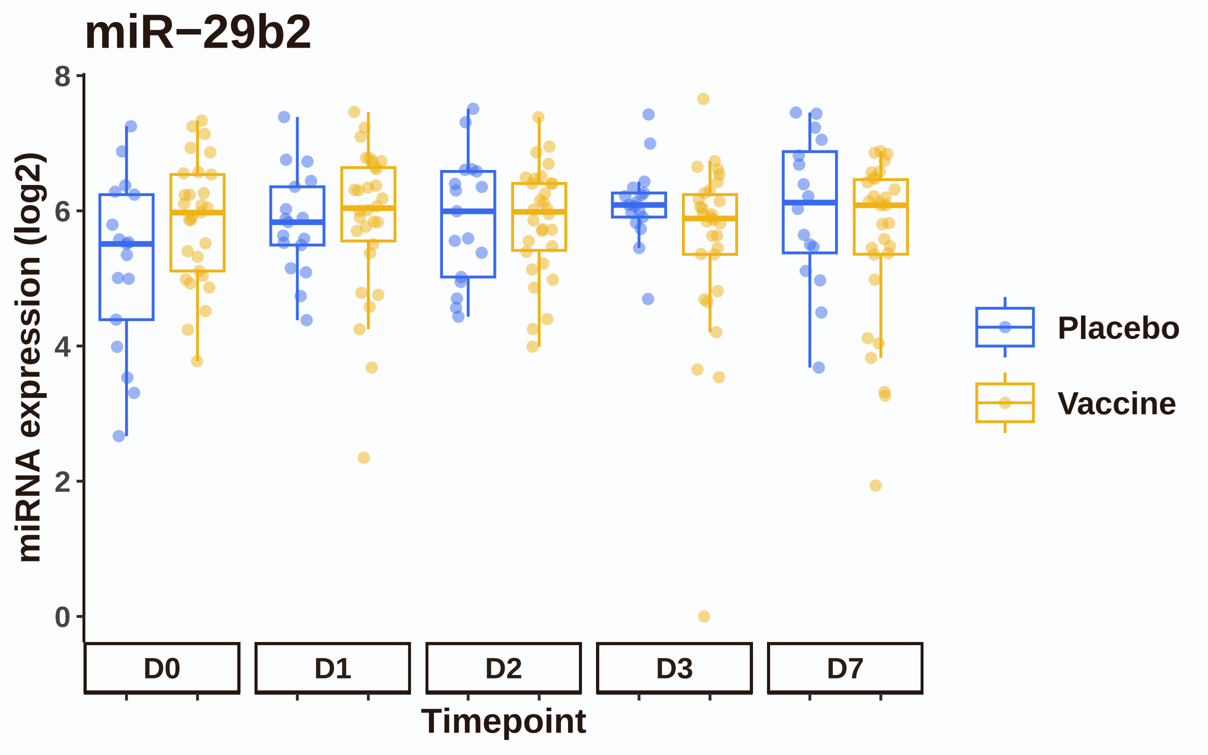
miR−29b2
miRNA expression (log2)
8
6
4
2
0
D0
D1
D2
D3
D7
Timepoint
Placebo
Vaccine

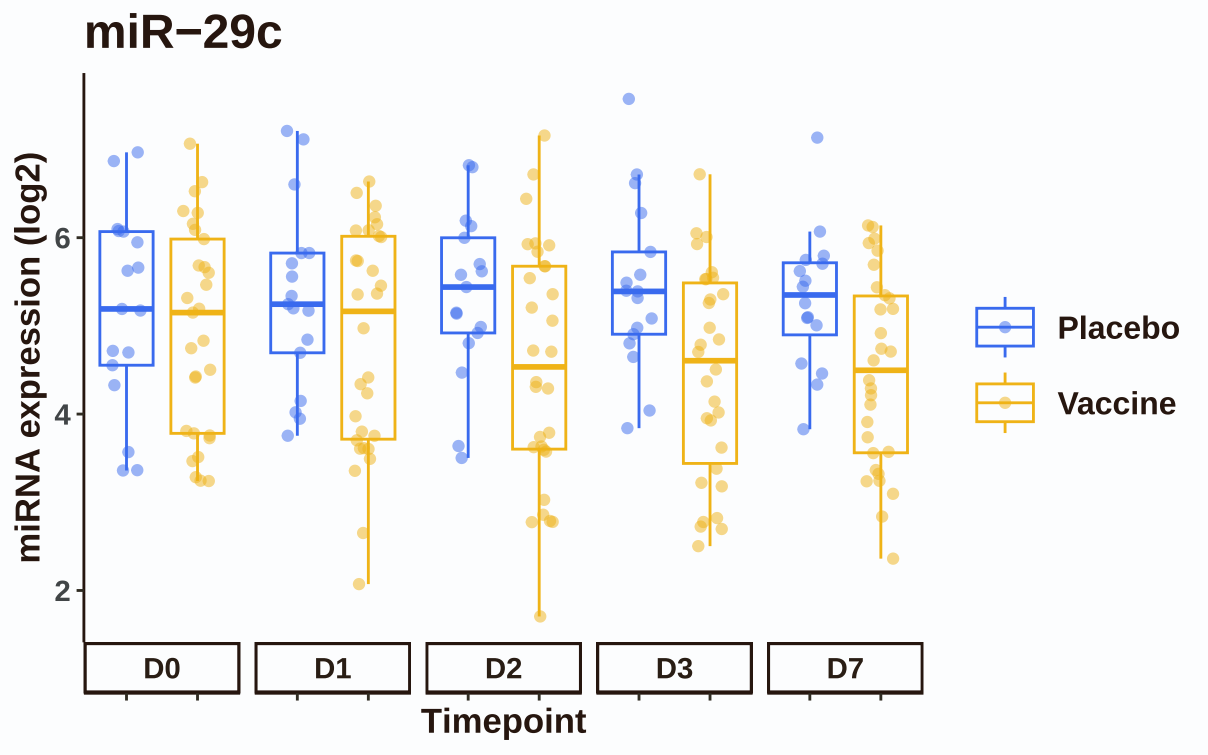
miR−29c
miRNA expression (log2)
6
4
2
D0
D1
D2
D3
D7
Timepoint
Placebo
Vaccine

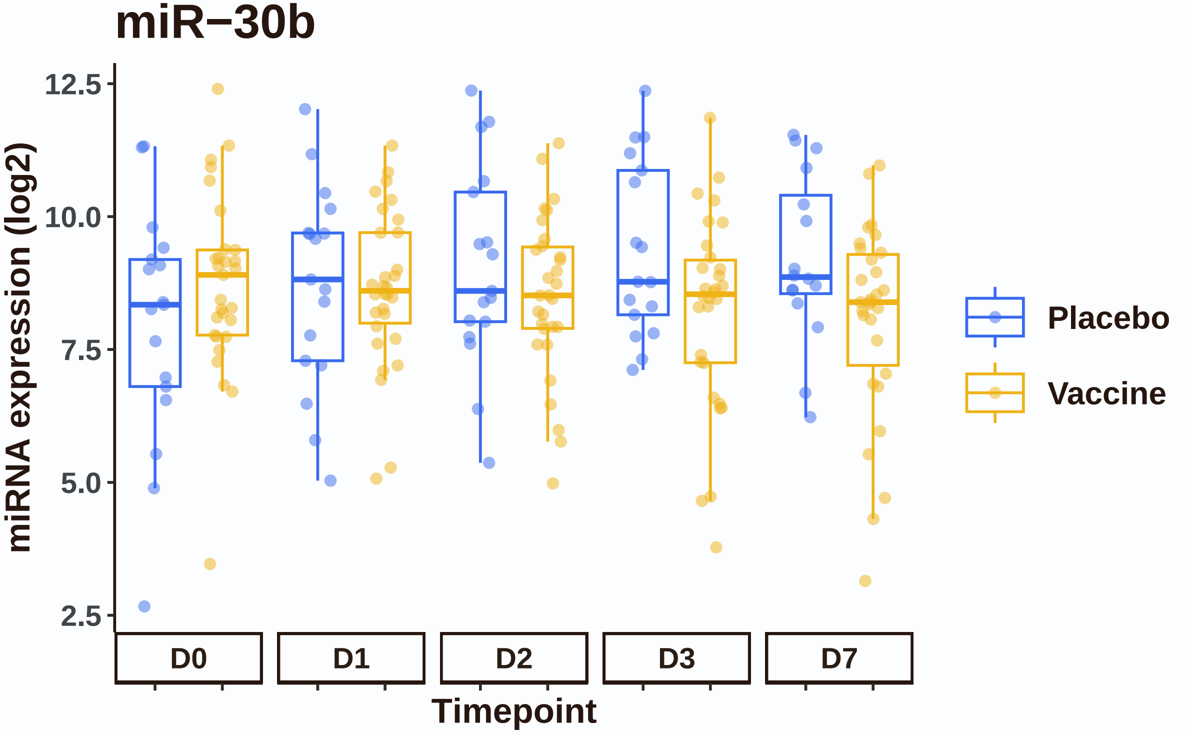

miR−30b
miRNA expression (log2)
12.5
10.0
7.5
5.0
2.5
D0
D1
D2
D3
D7
Timepoint
Placebo
Vaccine

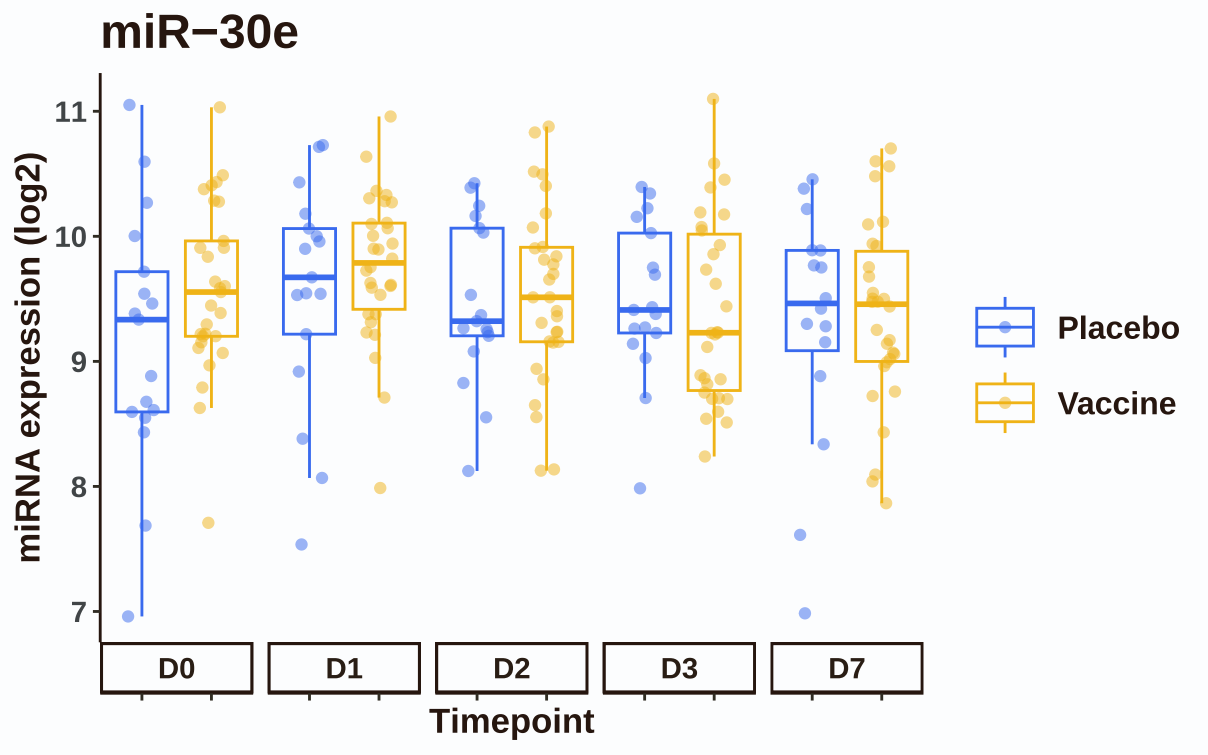

miR−30e
miRNA expression (log2)
11
10
9
8
7
D0
D1
D2
D3
D7
Timepoint
Placebo
Vaccine

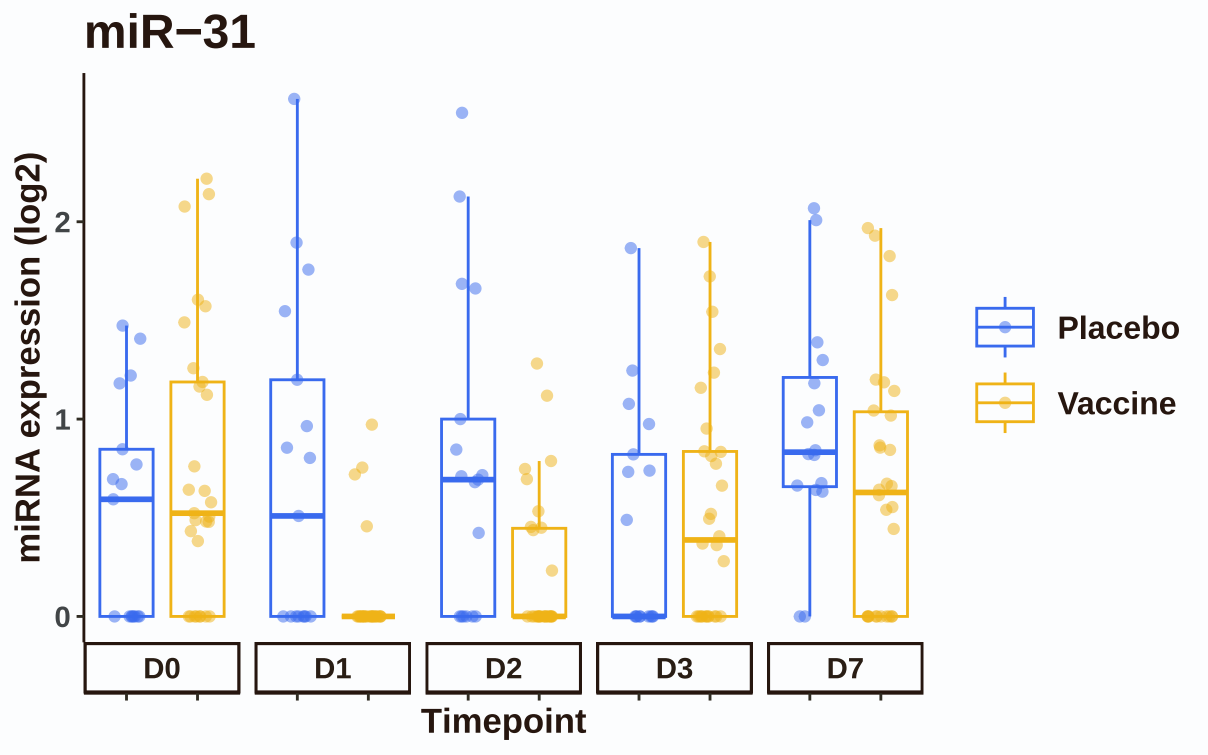

miR−31
miRNA expression (log2)
2
1
0
D0
D1
D2
D3
D7
Timepoint
Placebo
Vaccine

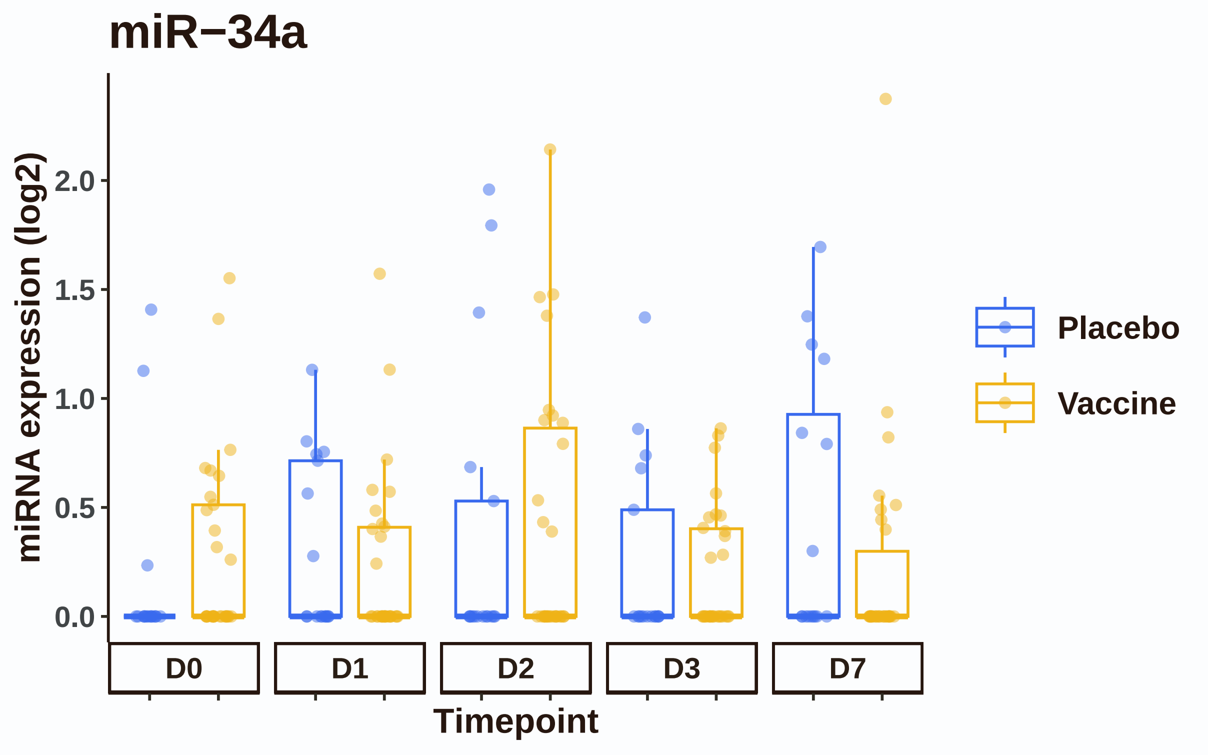

miR−34a
miRNA expression (log2)
2.0
1.5
1.0
0.5
0.0
D0
D1
D2
D3
D7
Timepoint
Placebo
Vaccine

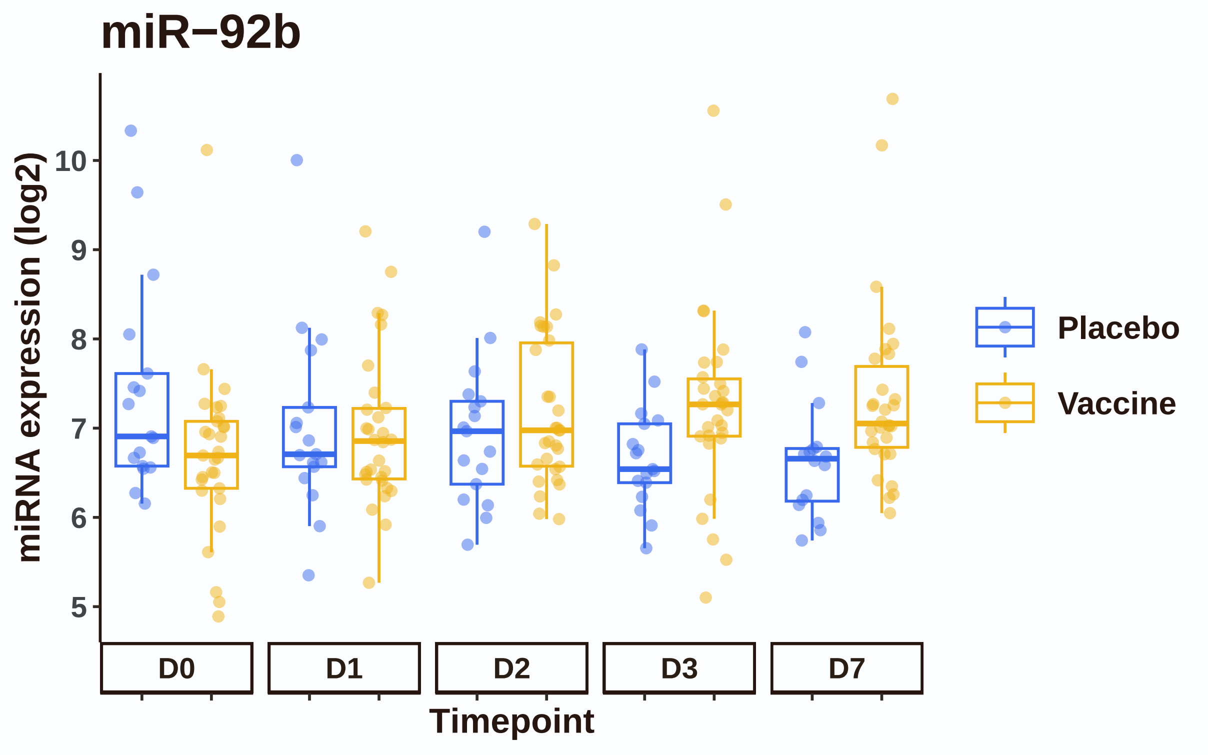
miR−92b
miRNA expression (log2)
10
9
8
7
6
5
D0
D1
D2
D3
D7
Timepoint
Placebo
Vaccine

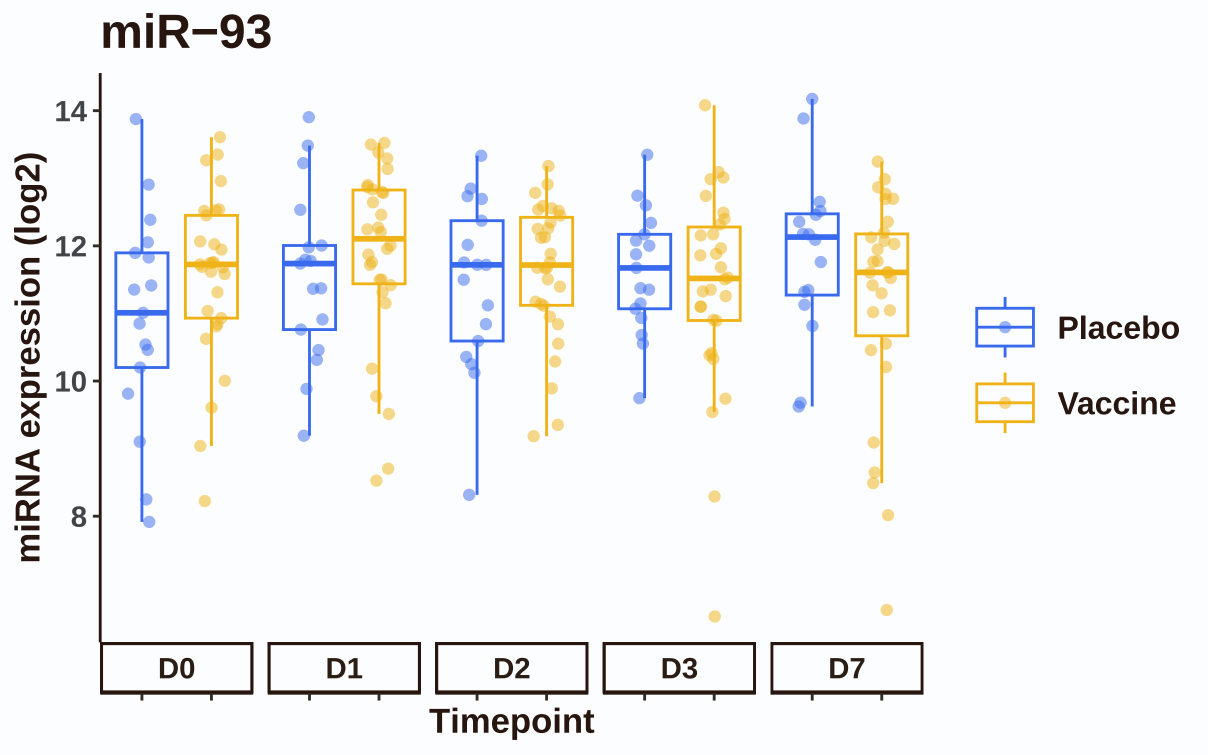

miR−93
miRNA expression (log2)
14
12
10
8
D0
D1
D2
D3
D7
Timepoint
Placebo
Vaccine

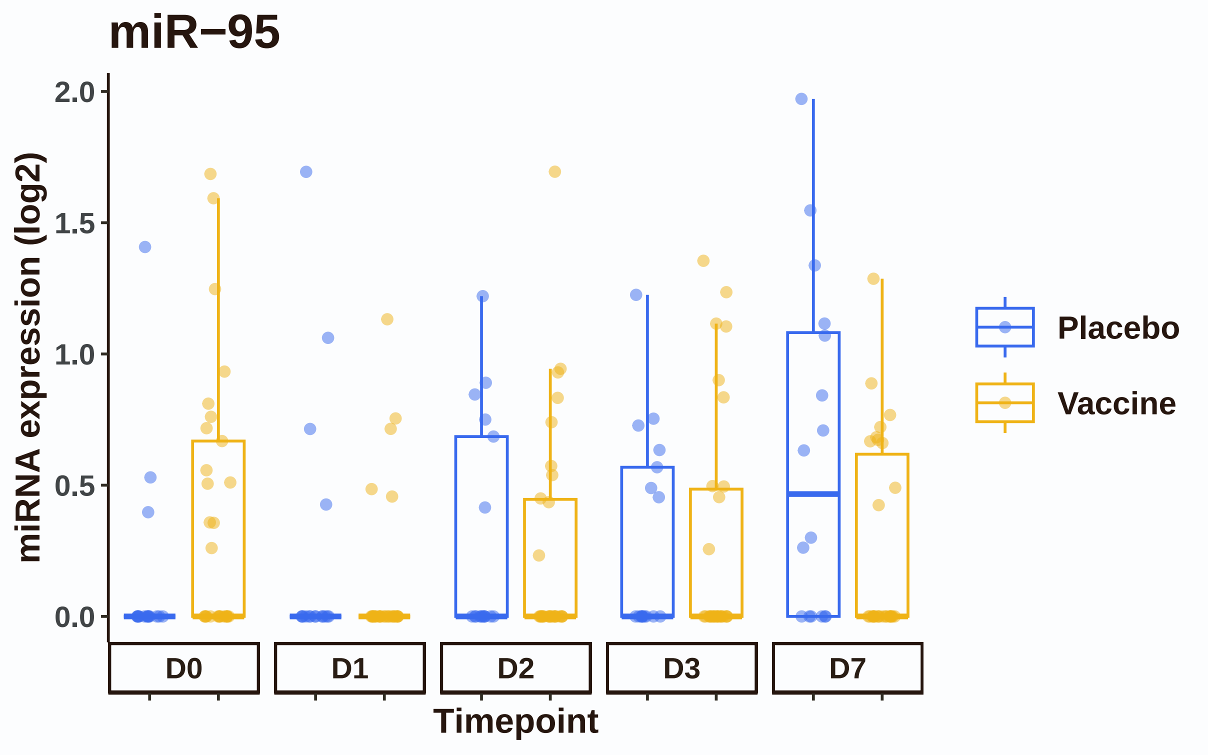

miR−95
miRNA expression (log2)
2.0
1.5
1.0
0.5
0.0
D0
D1
D2
D3
D7
Timepoint
Placebo
Vaccine

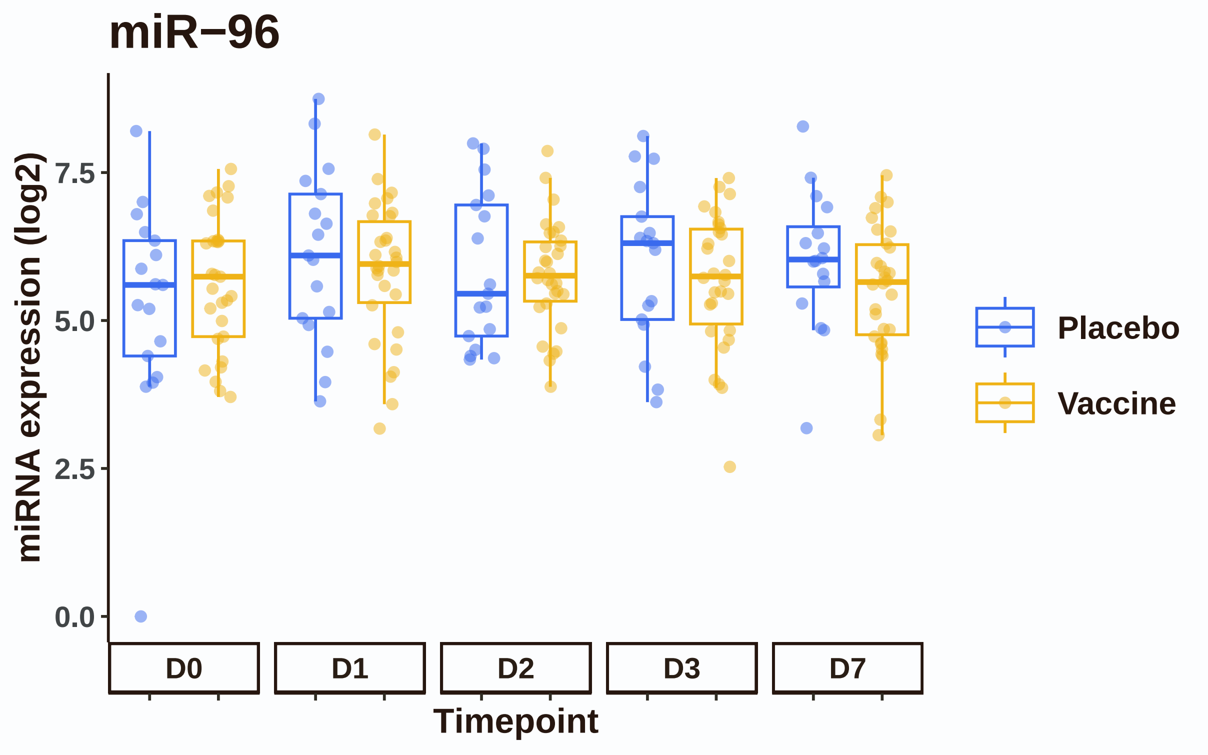

miR−96
miRNA expression (log2)
7.5
5.0
2.5
0.0
D0
D1
D2
D3
D7
Timepoint
Placebo
Vaccine

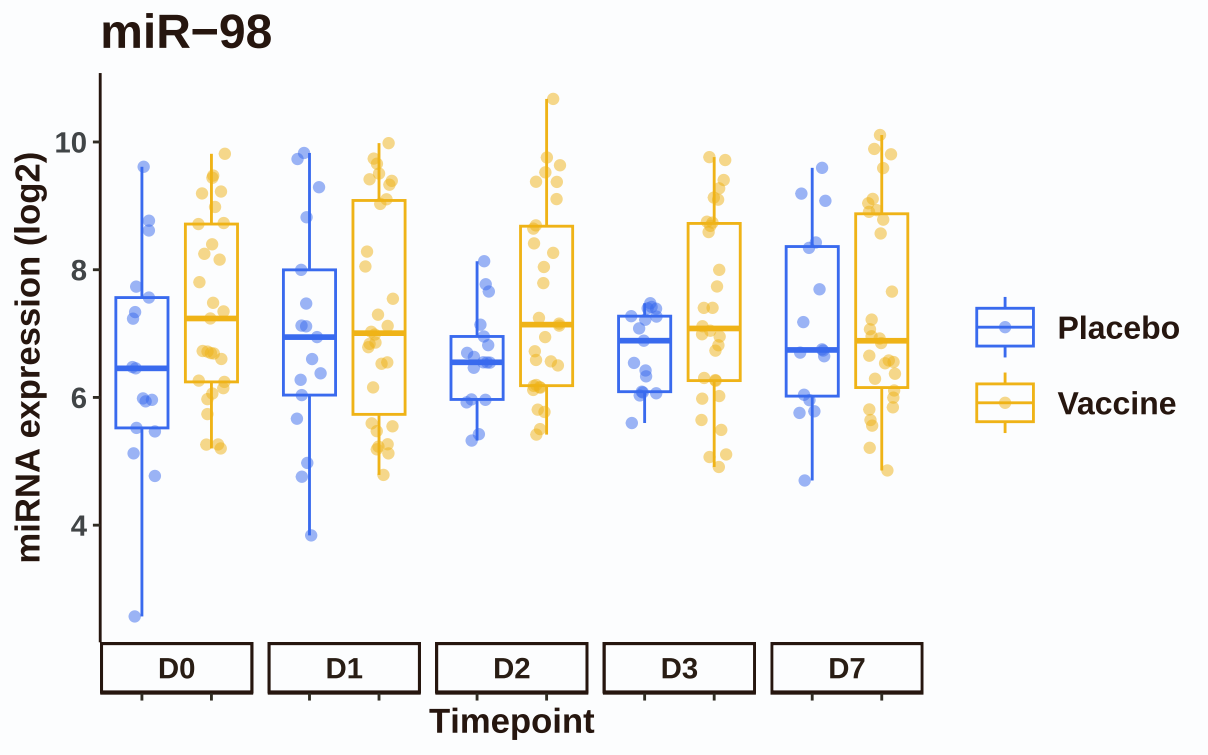

miR−98
miRNA expression (log2)
10
8
6
4
D0
D1
D2
D3
D7
Timepoint
Placebo
Vaccine

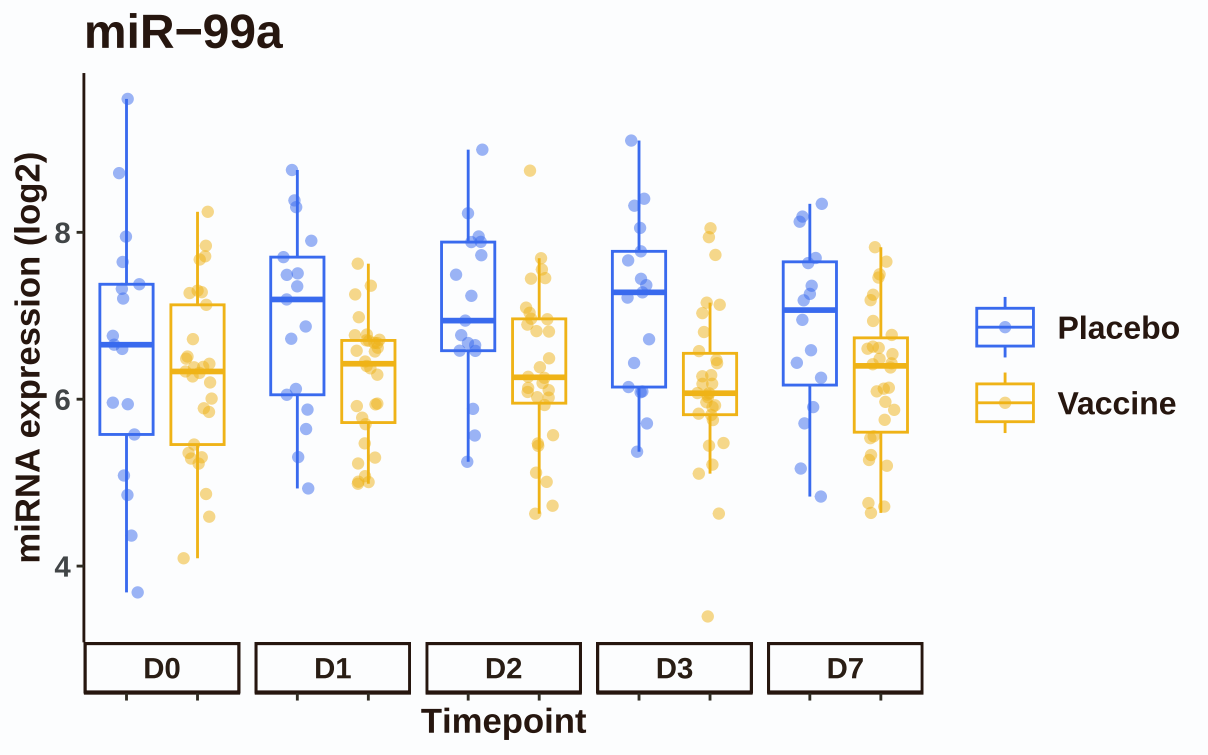

miR−99a
miRNA expression (log2)
8
6
4
D0
D1
D2
D3
D7
Timepoint
Placebo
Vaccine

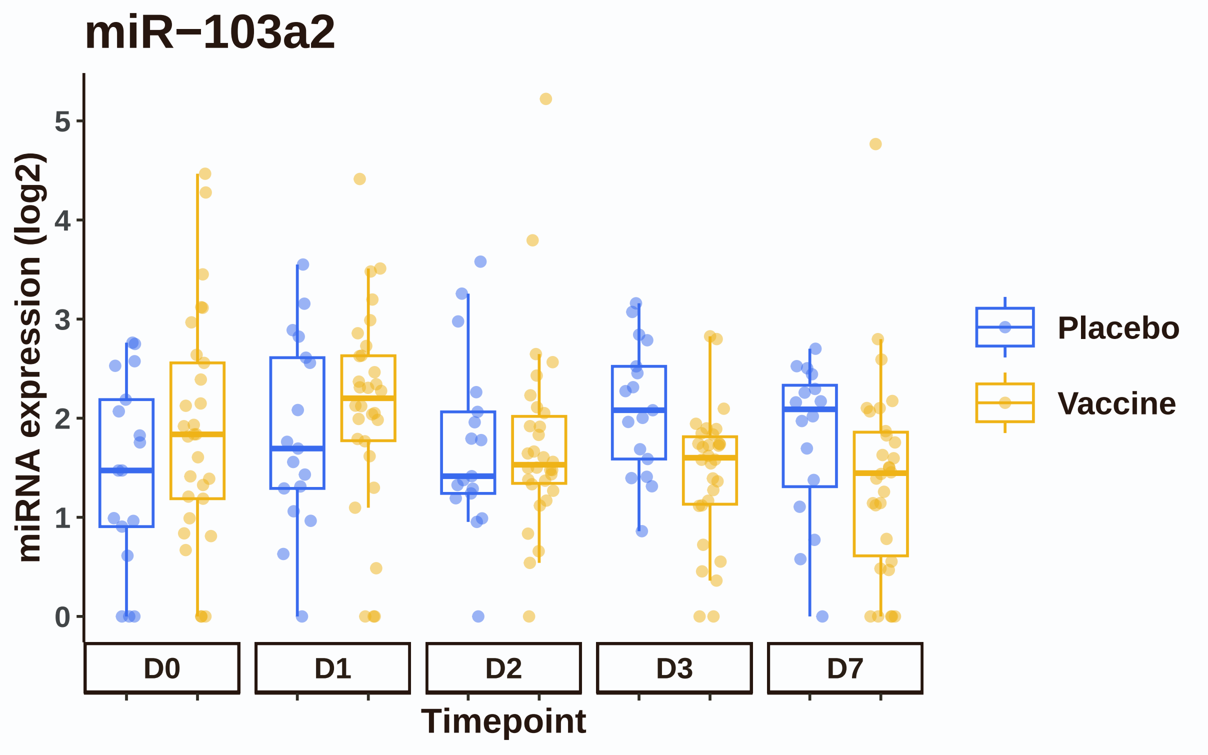
miR−103a2
miRNA expression (log2)
0
1
2
3
4
5
D0
D1
D2
D3
D7
Timepoint
Placebo
Vaccine

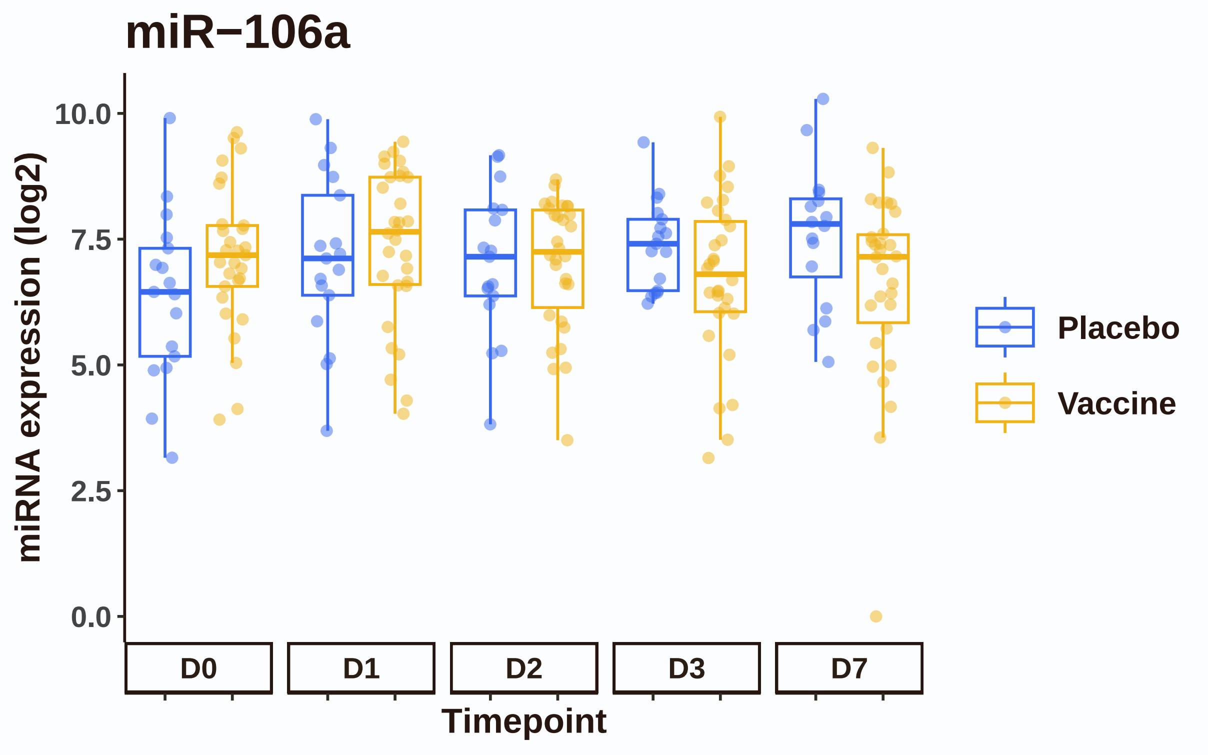

miR−106a
miRNA expression (log2)
10.0
7.5
5.0
2.5
0.0
D0
D1
D2
D3
D7
Timepoint
Placebo
Vaccine

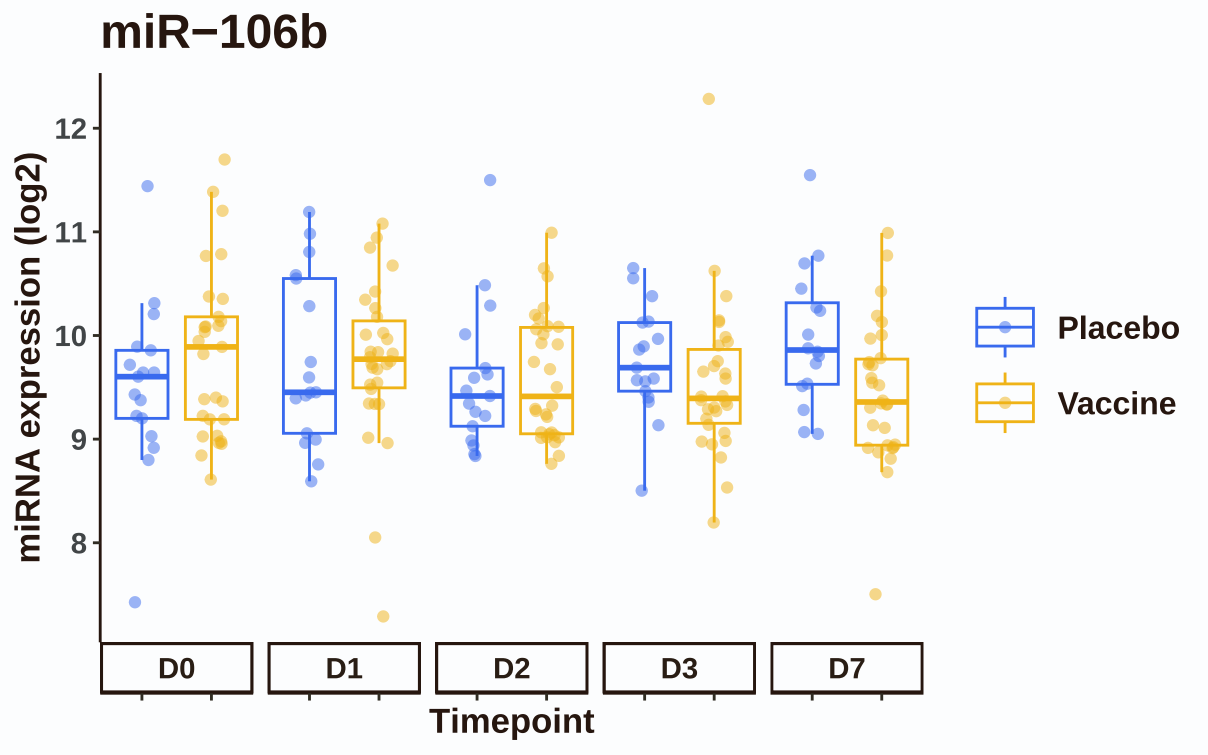
miR−106b
miRNA expression (log2)
12
11
10
9
8
D0
D1
D2
D3
D7
Timepoint
Placebo
Vaccine

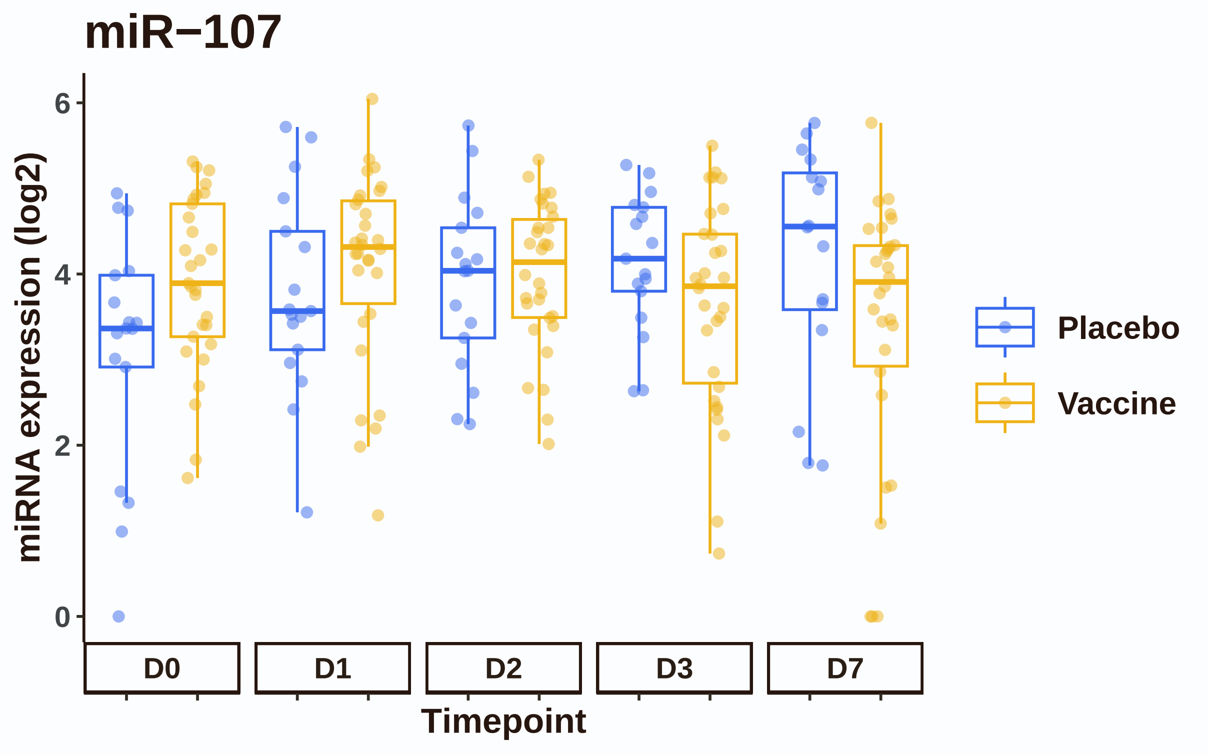
miR−107
miRNA expression (log2)
0
2
4
6
D0
D1
D2
D3
D7
Timepoint
Placebo
Vaccine

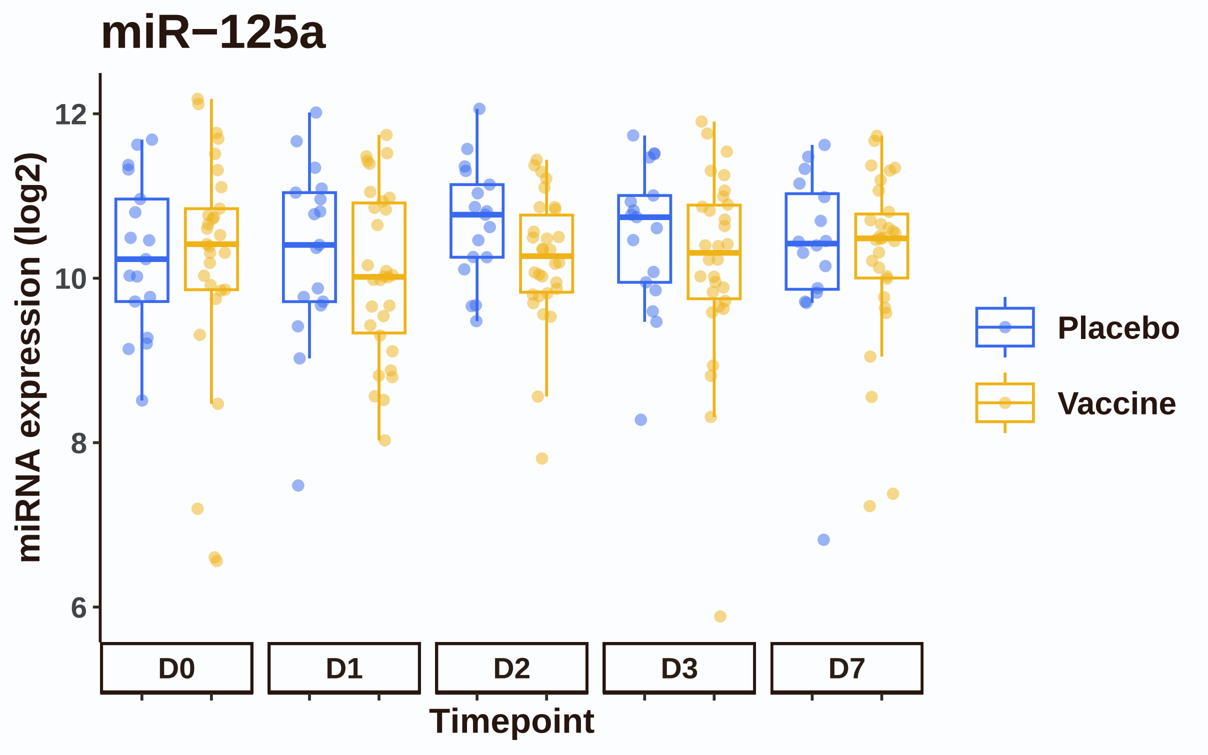

miR−125a
miRNA expression (log2)
12
10
8
6
D0
D1
D2
D3
D7
Timepoint
Placebo
Vaccine

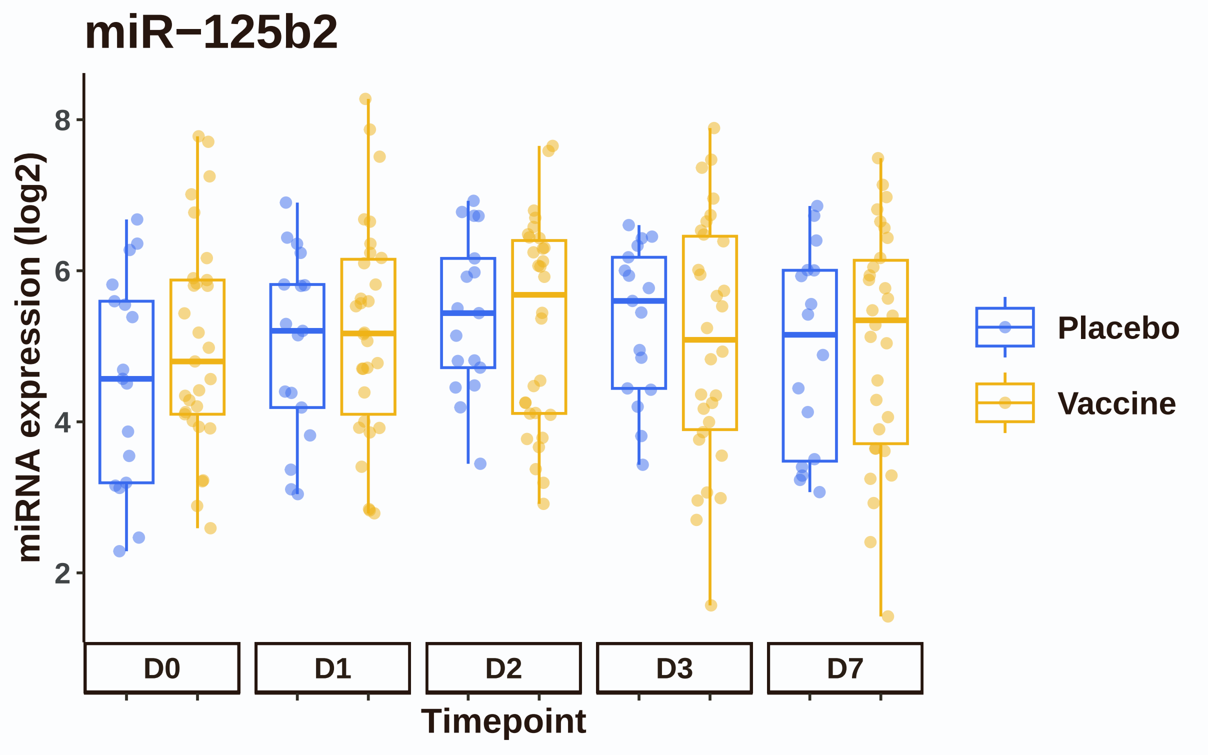

miR−125b2
miRNA expression (log2)
8
6
4
2
D0
D1
D2
D3
D7
Timepoint
Placebo
Vaccine

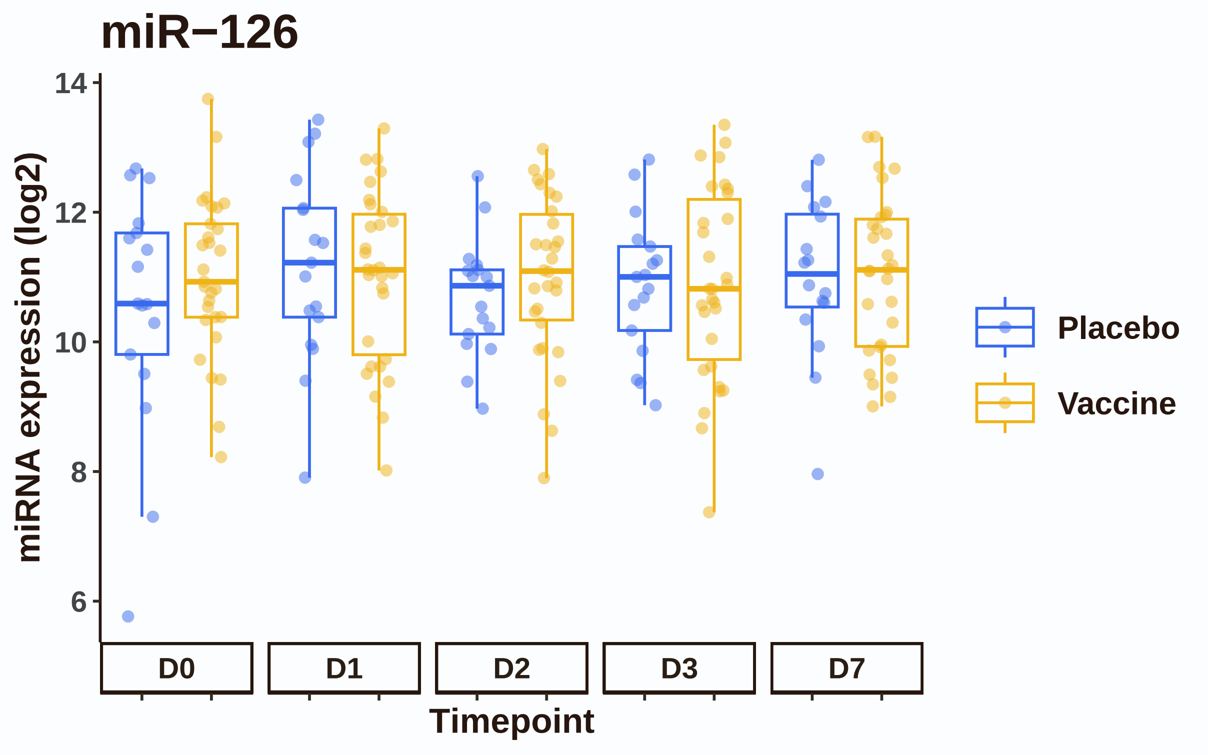

miR−126
miRNA expression (log2)
14
12
10
8
6
D0
D1
D2
D3
D7
Timepoint
Placebo
Vaccine

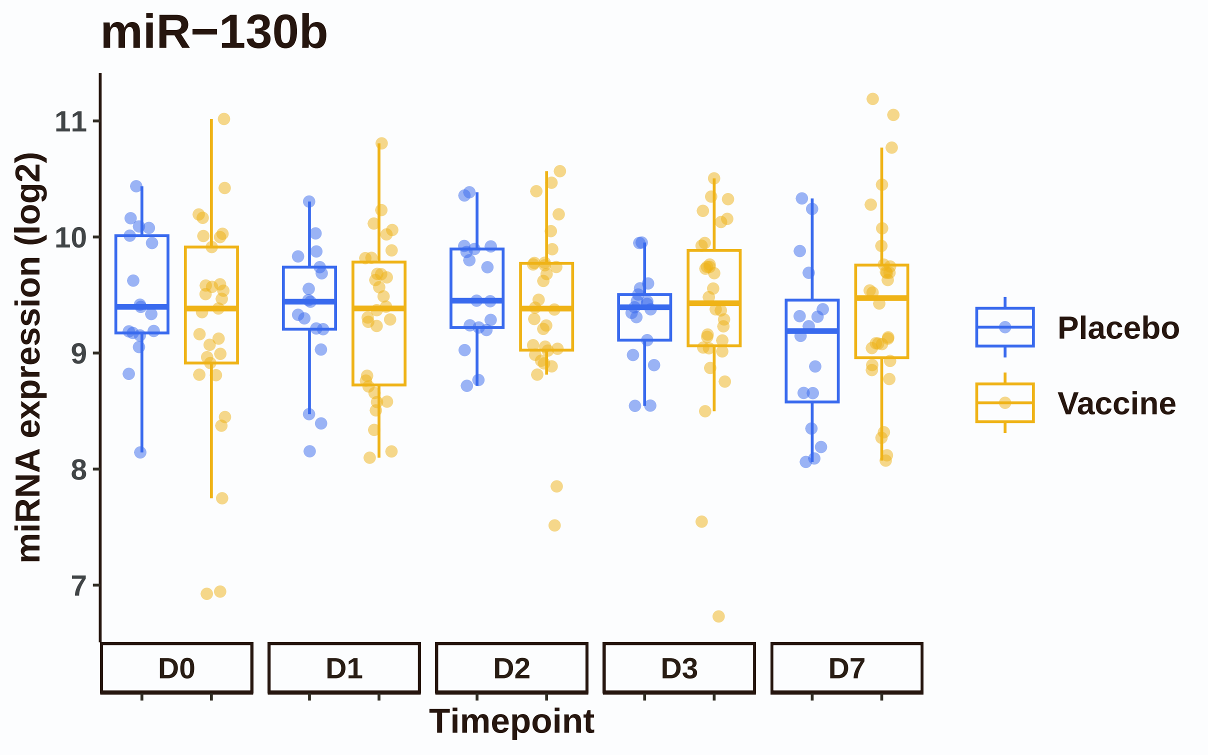
miR−130b
miRNA expression (log2)
11
10
9
8
7
D0
D1
D2
D3
D7
Timepoint
Placebo
Vaccine

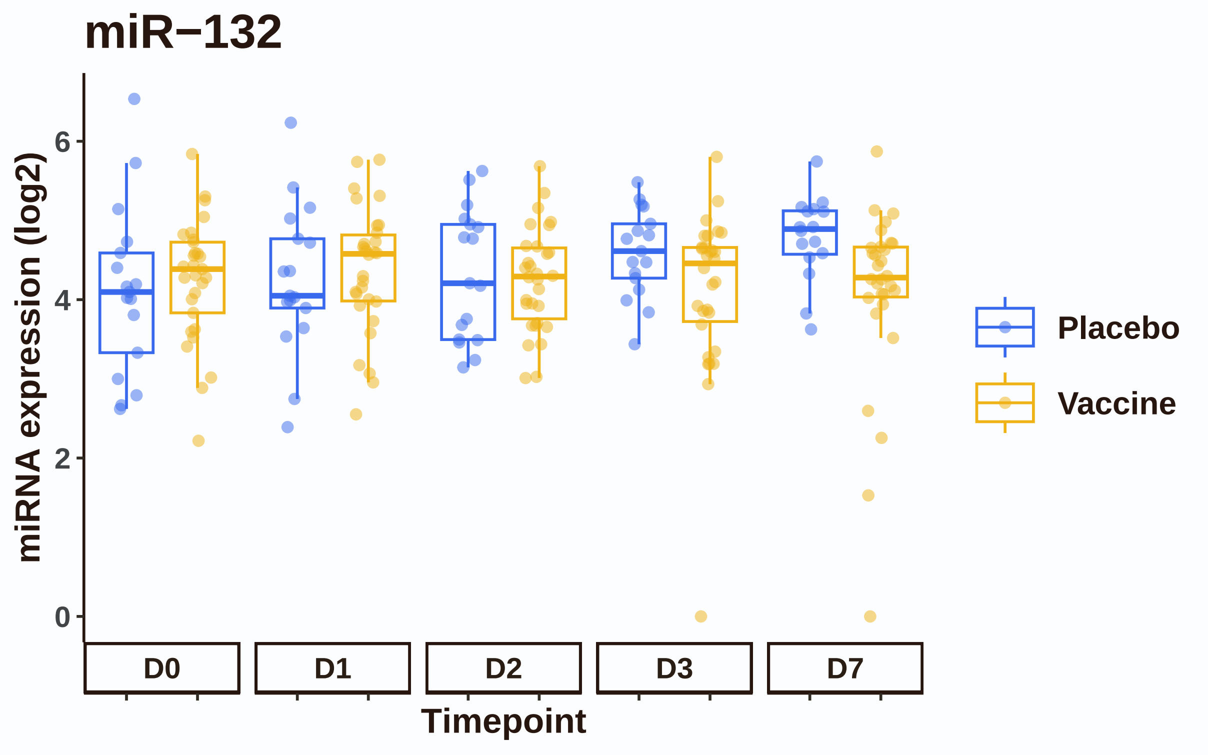
miR−132
miRNA expression (log2)
6
4
2
0
D0
D1
D2
D3
D7
Timepoint
Placebo
Vaccine

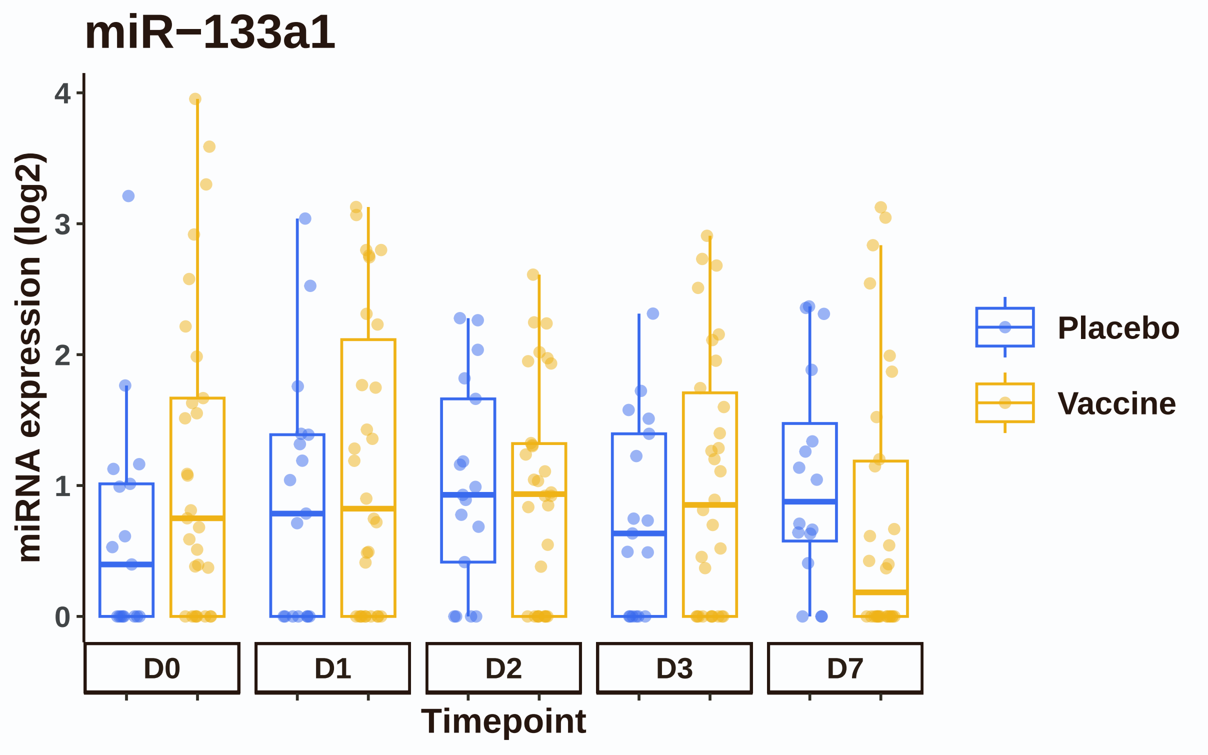

miR−133a1
miRNA expression (log2)
0
1
2
3
4
D0
D1
D2
D3
D7
Timepoint
Placebo
Vaccine

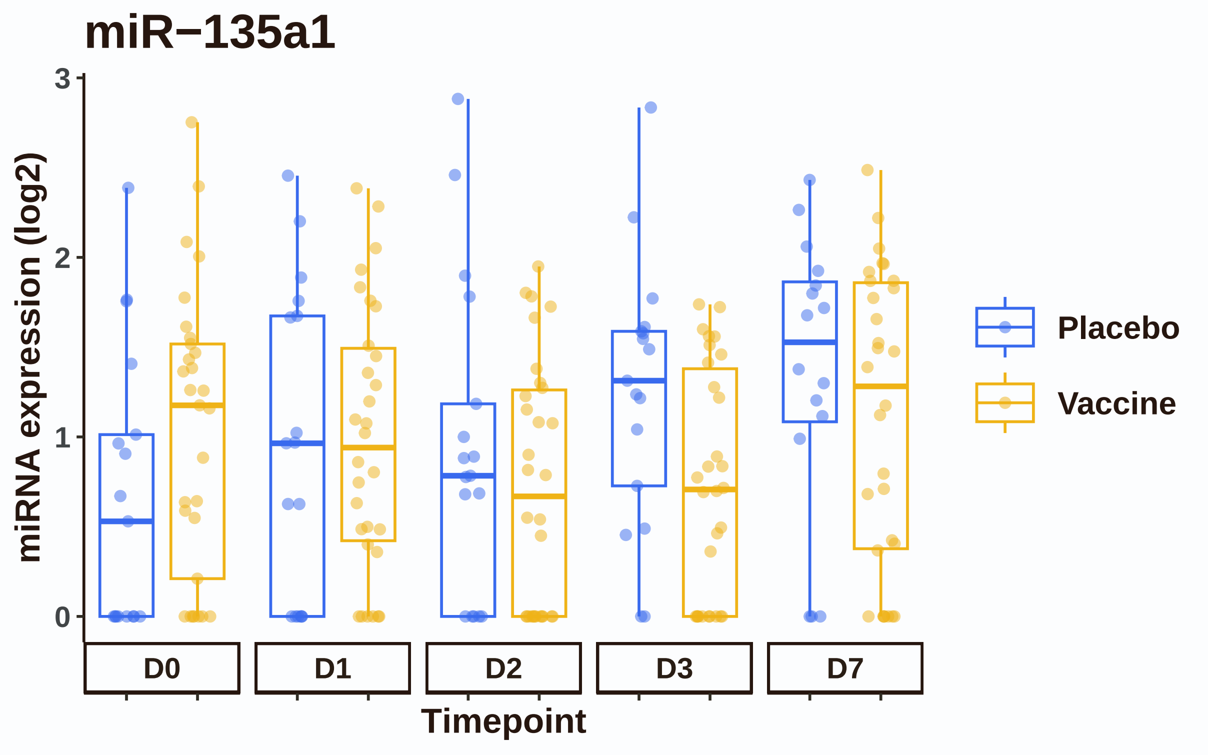
miR−135a1
miRNA expression (log2)
0
1
2
3
D0
D1
D2
D3
D7
Timepoint
Placebo
Vaccine

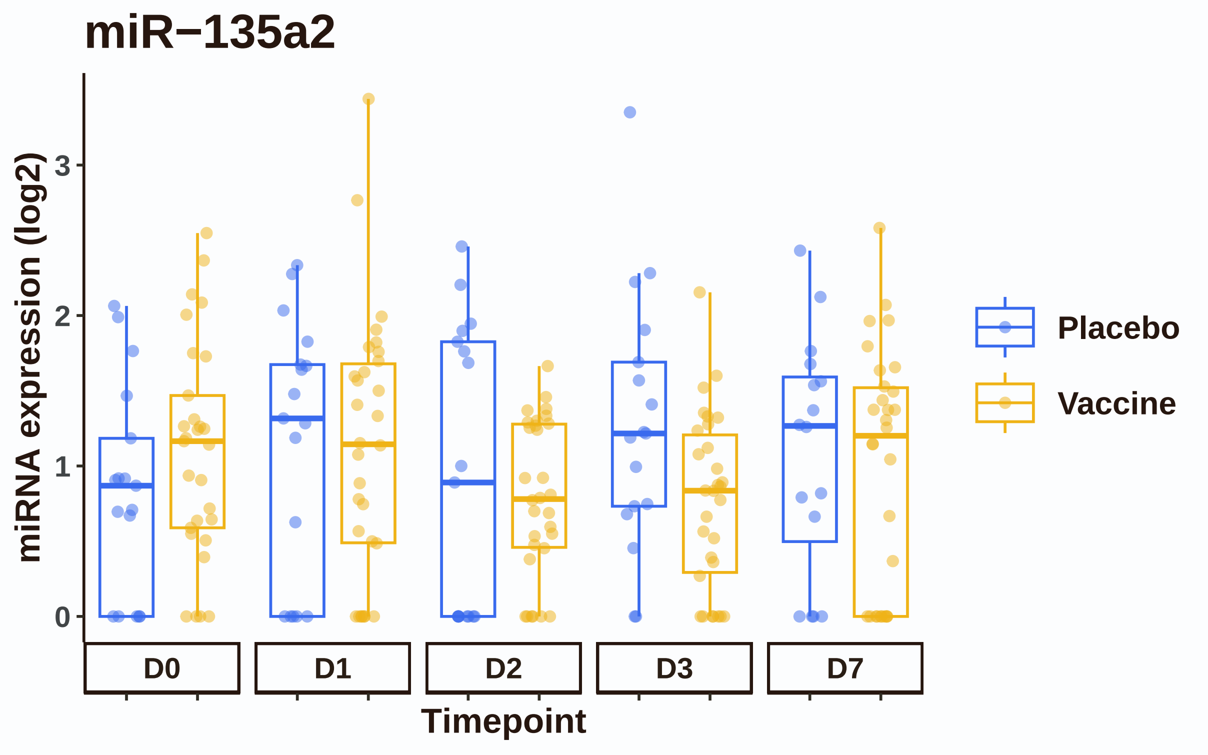

miR−135a2
miRNA expression (log2)
0
1
2
3
D0
D1
D2
D3
D7
Timepoint
Placebo
Vaccine

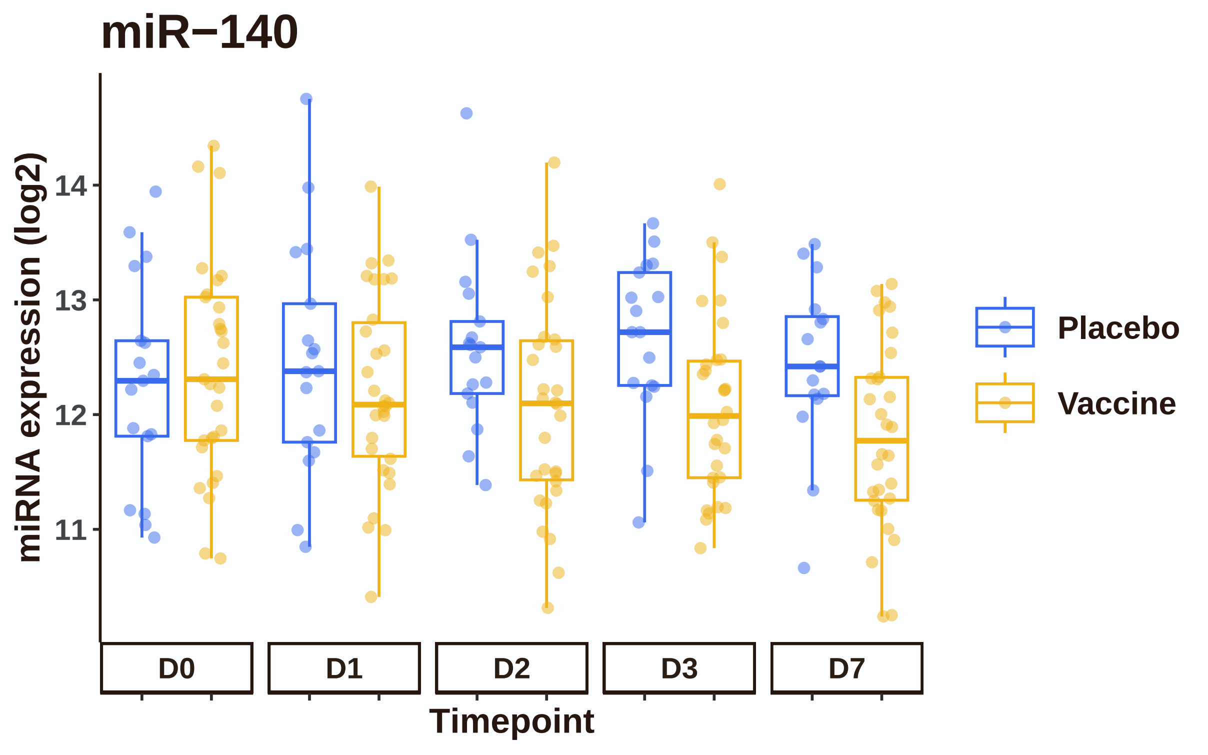

miR−140
miRNA expression (log2)
14
13
12
11
D0
D1
D2
D3
D7
Timepoint
Placebo
Vaccine

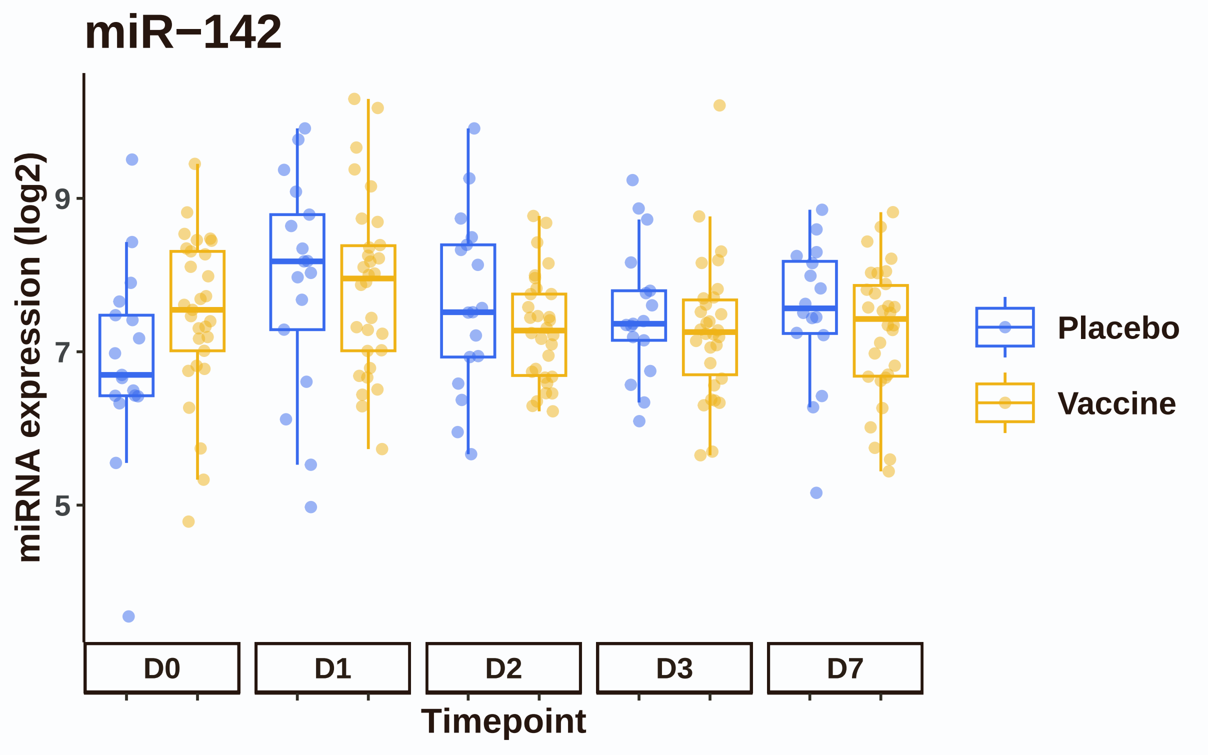
miR−142
miRNA expression (log2)
9
7
5
D0
D1
D2
D3
D7
Timepoint
Placebo
Vaccine

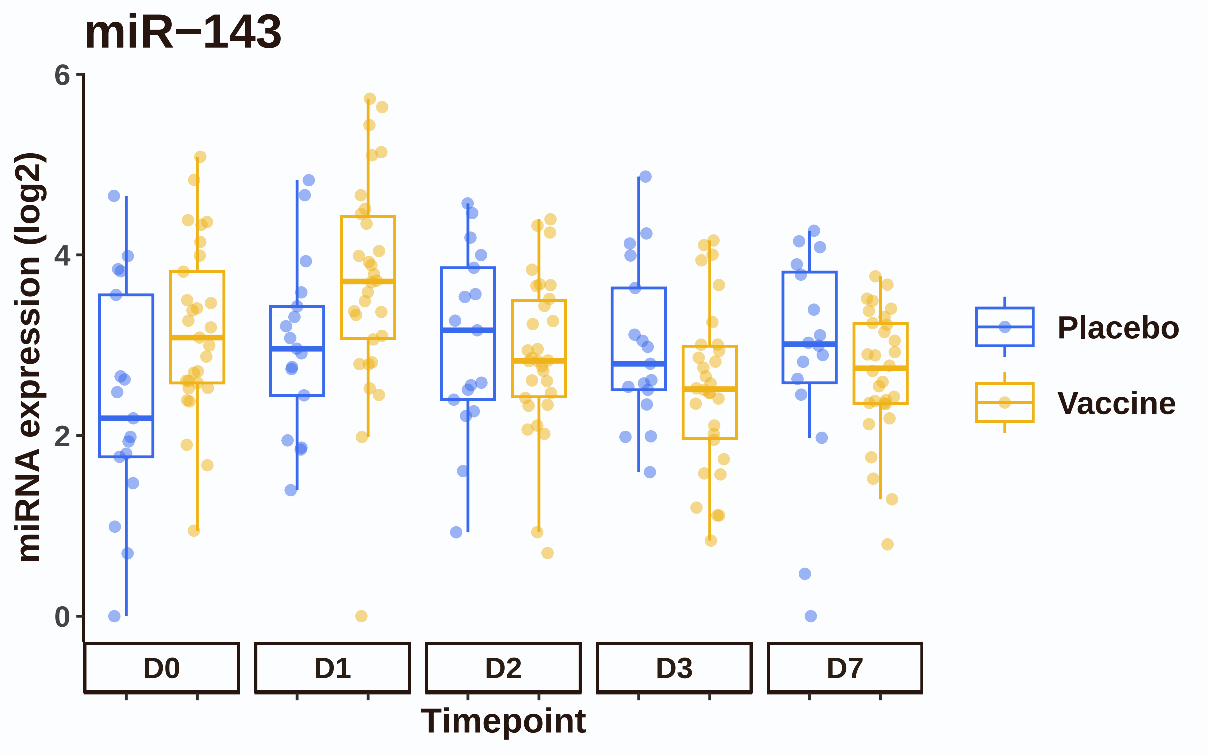
miR−143
miRNA expression (log2)
6
4
2
0
D0
D1
D2
D3
D7
Timepoint
Placebo
Vaccine

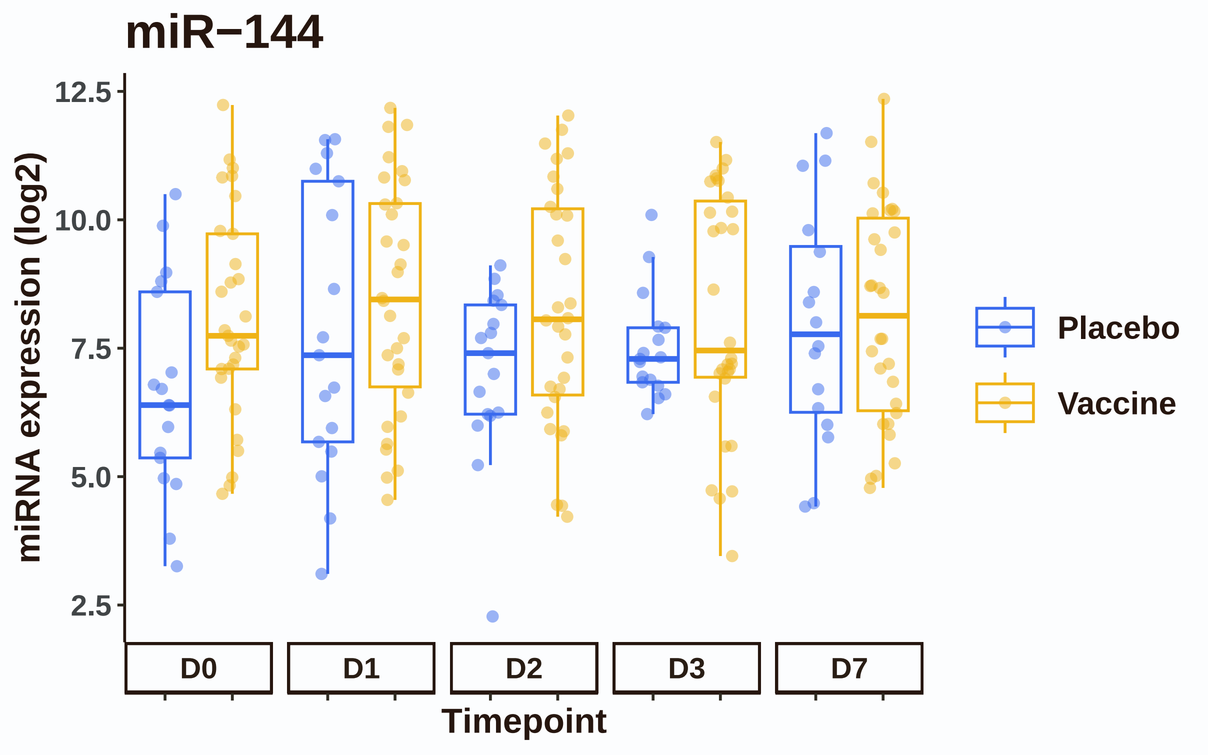
miR−144
miRNA expression (log2)
12.5
10.0
7.5
5.0
2.5
D0
D1
D2
D3
D7
Timepoint
Placebo
Vaccine

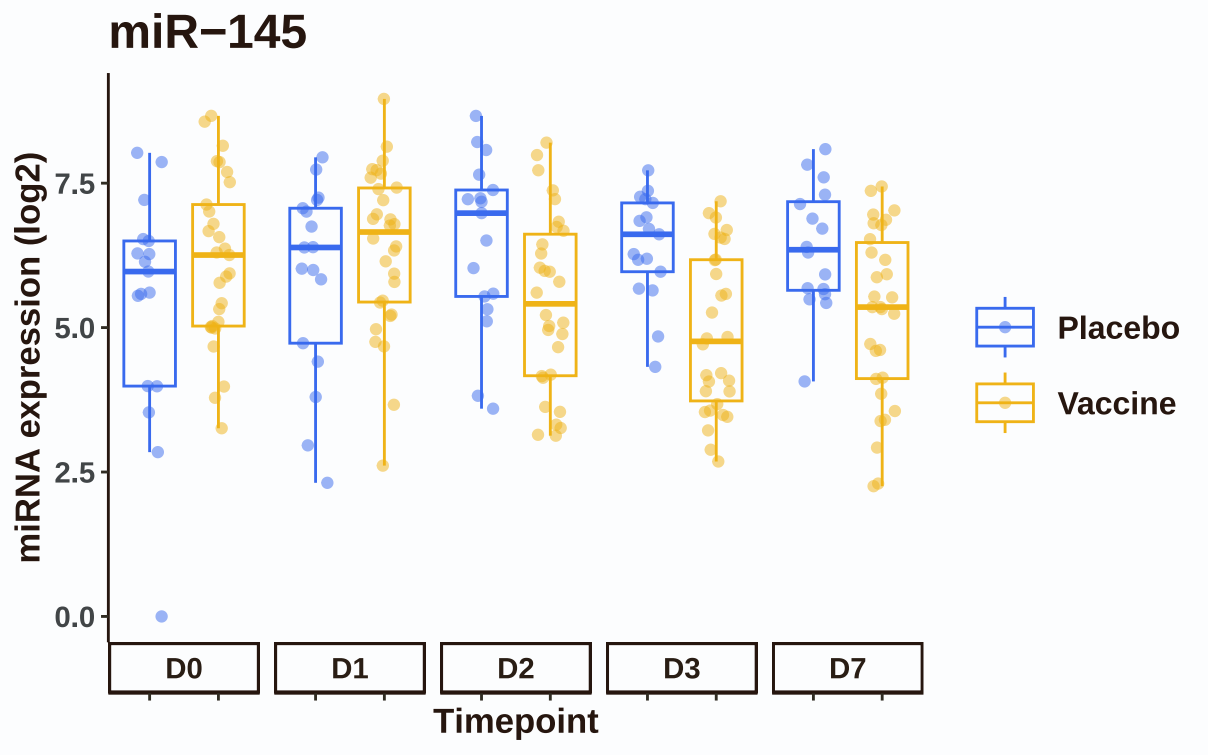
miR−145
miRNA expression (log2)
7.5
5.0
2.5
0.0
D0
D1
D2
D3
D7
Timepoint
Placebo
Vaccine

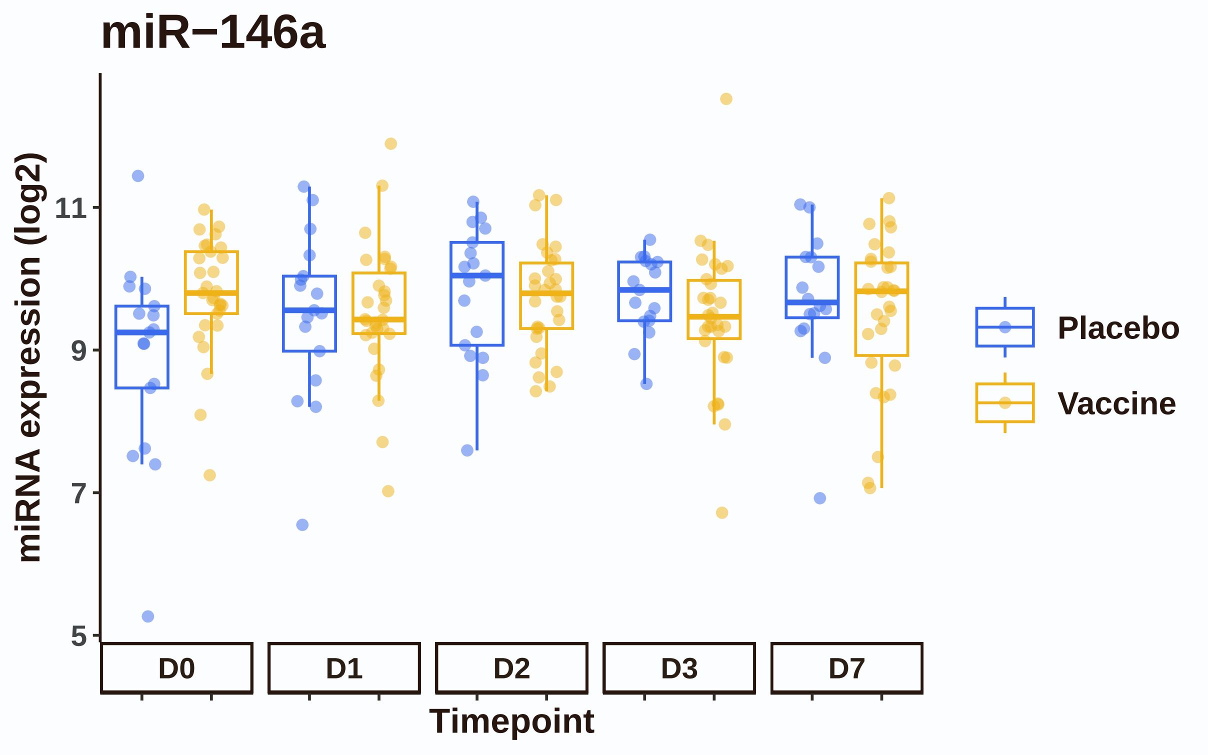

miR−146a
miRNA expression (log2)
11
9
7
5
D0
D1
D2
D3
D7
Timepoint
Placebo
Vaccine

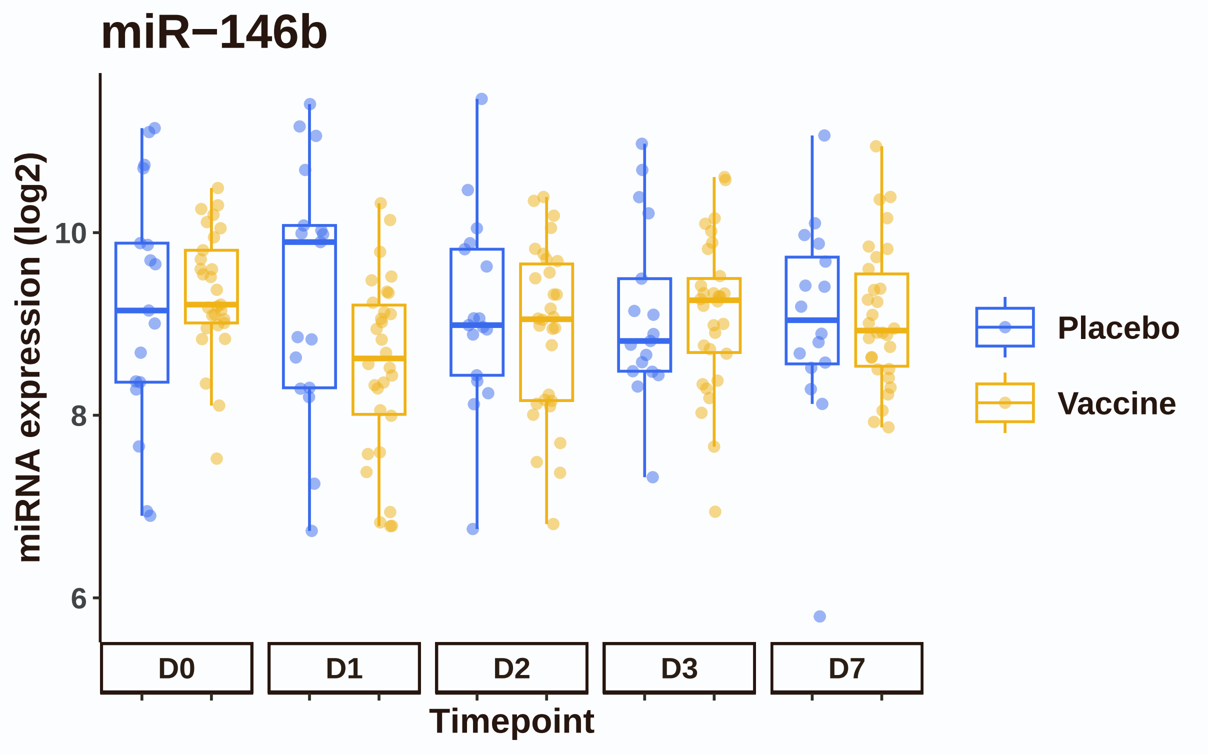
miR−146b
miRNA expression (log2)
10
8
6
D0
D1
D2
D3
D7
Timepoint
Placebo
Vaccine

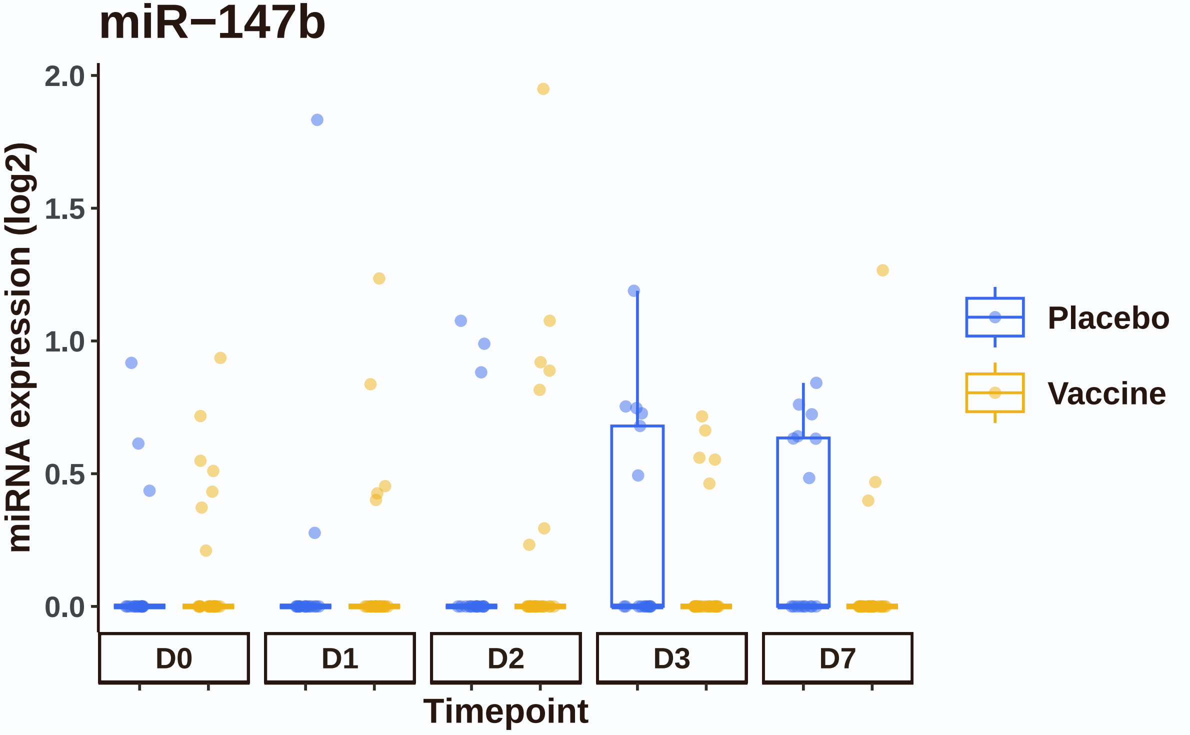

miR−147b
miRNA expression (log2)
2.0
1.5
1.0
0.5
0.0
D0
D1
D2
D3
D7
Timepoint
Placebo
Vaccine

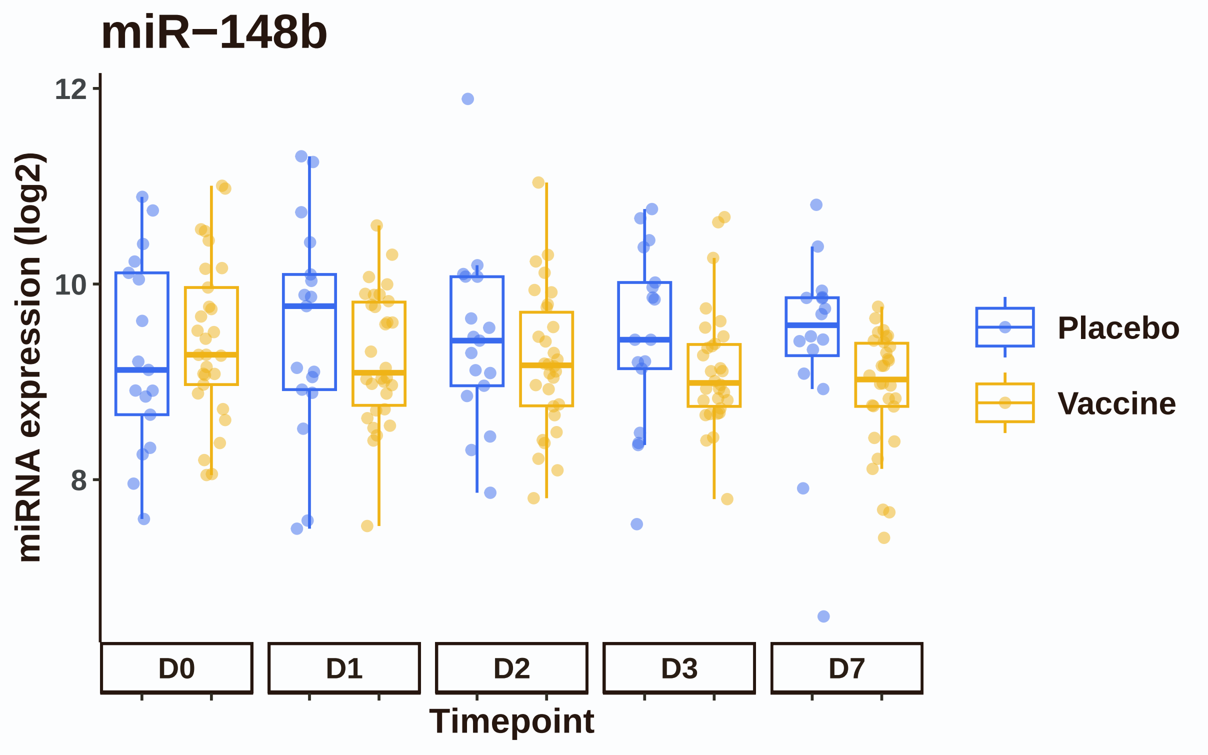
miR−148b
miRNA expression (log2)
12
10
8
D0
D1
D2
D3
D7
Timepoint
Placebo
Vaccine

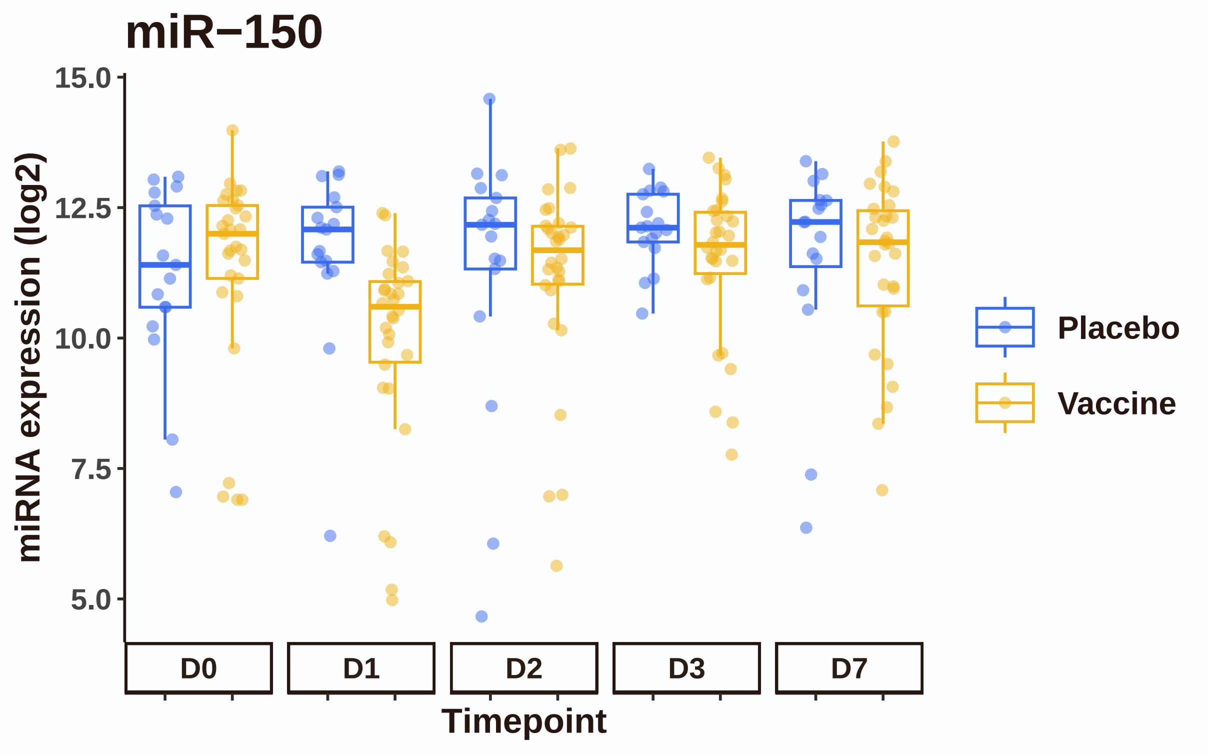
miR−150
miRNA expression (log2)
15.0
12.5
10.0
7.5
5.0
D0
D1
D2
D3
D7
Timepoint
Placebo
Vaccine

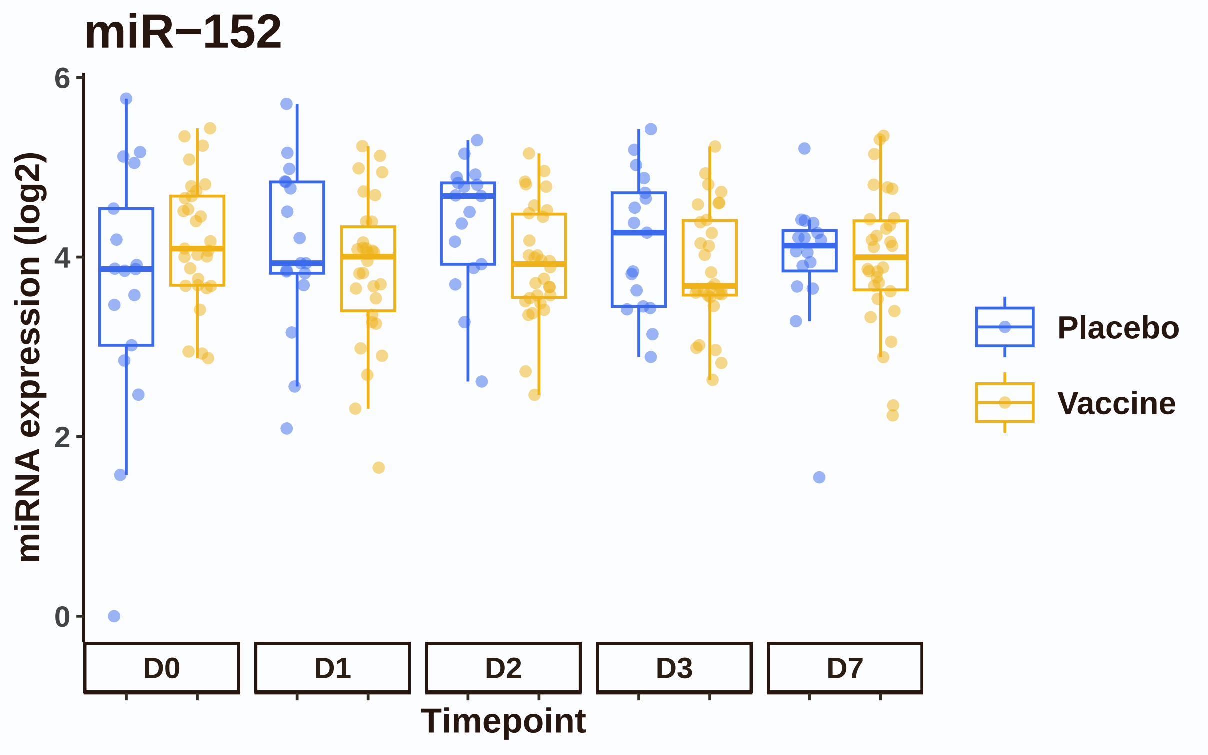

miR−152
miRNA expression (log2)
6
4
2
0
D0
D1
D2
D3
D7
Timepoint
Placebo
Vaccine

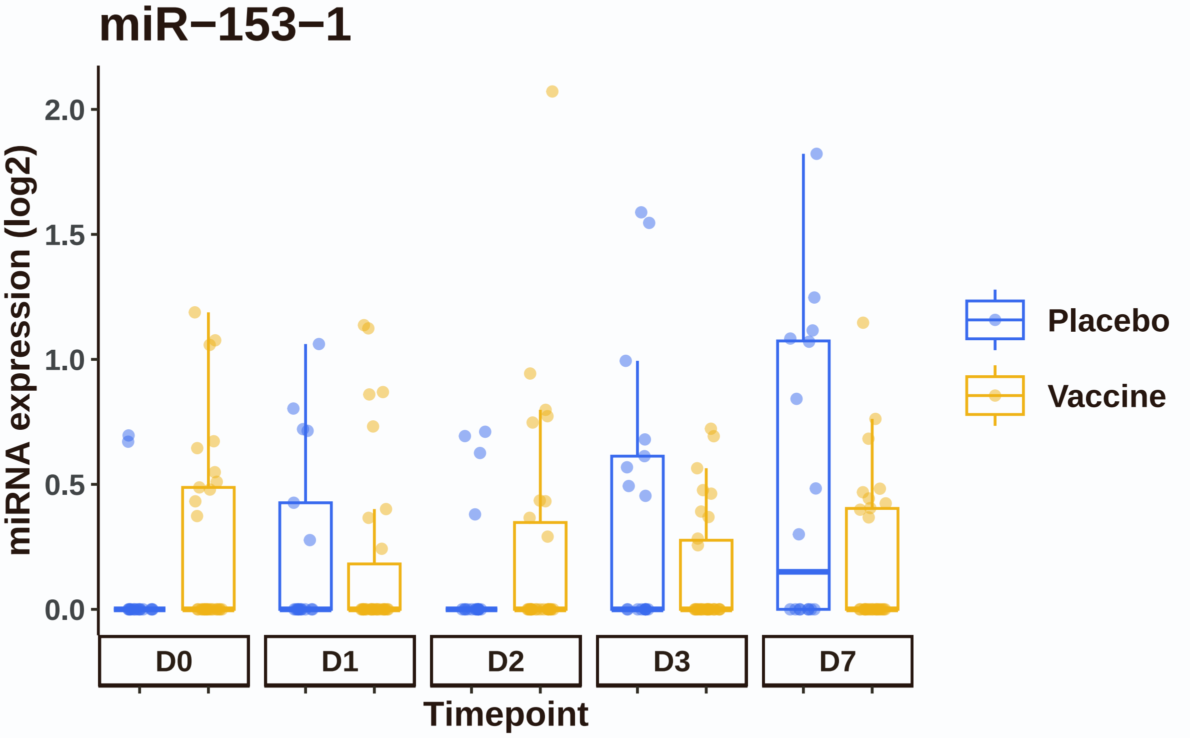

miR−153−1
miRNA expression (log2)
2.0
1.5
1.0
0.5
0.0
D0
D1
D2
D3
D7
Timepoint
Placebo
Vaccine

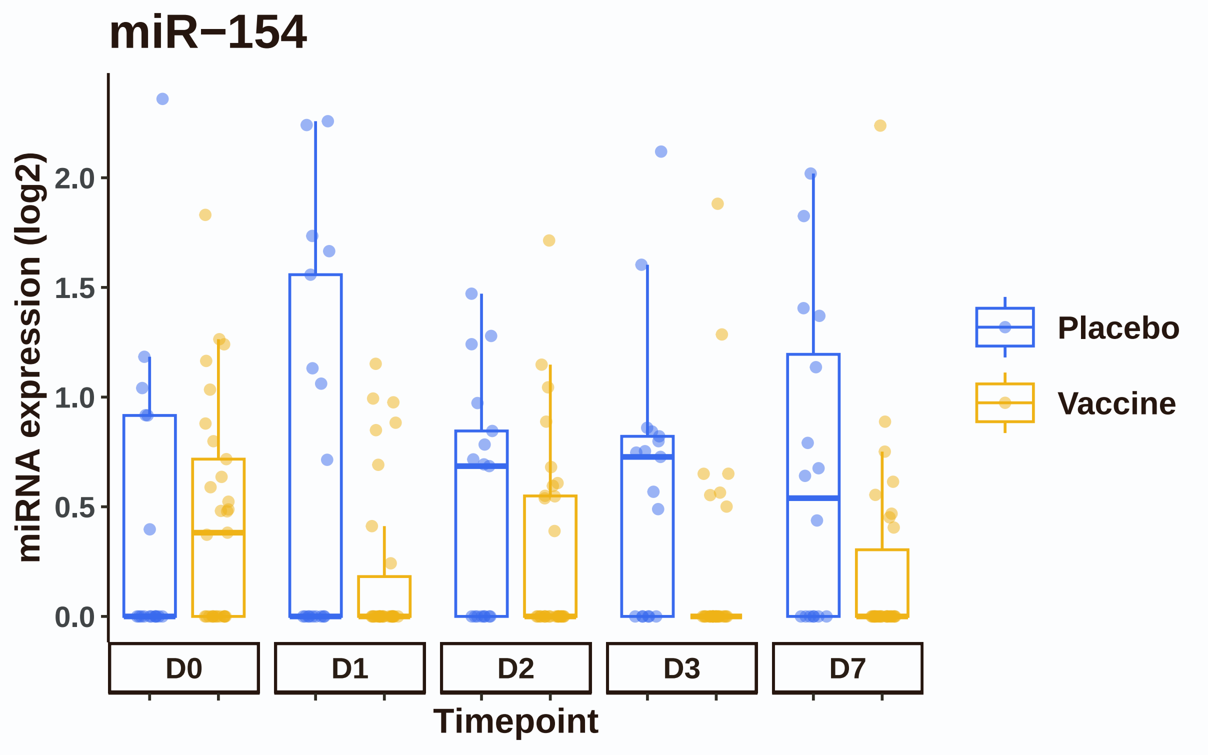
miR−154
miRNA expression (log2)
2.0
1.5
1.0
0.5
0.0
D0
D1
D2
D3
D7
Timepoint
Placebo
Vaccine

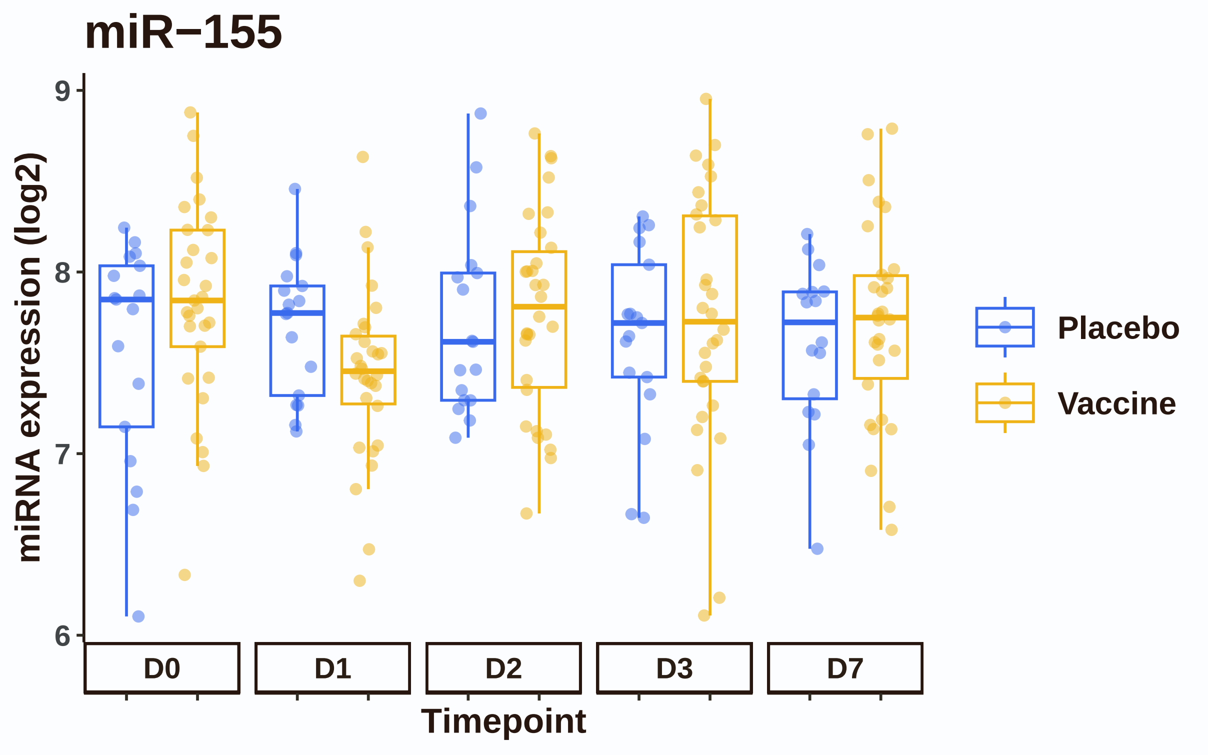

miR−155
miRNA expression (log2)
9
8
7
6
D0
D1
D2
D3
D7
Timepoint
Placebo
Vaccine

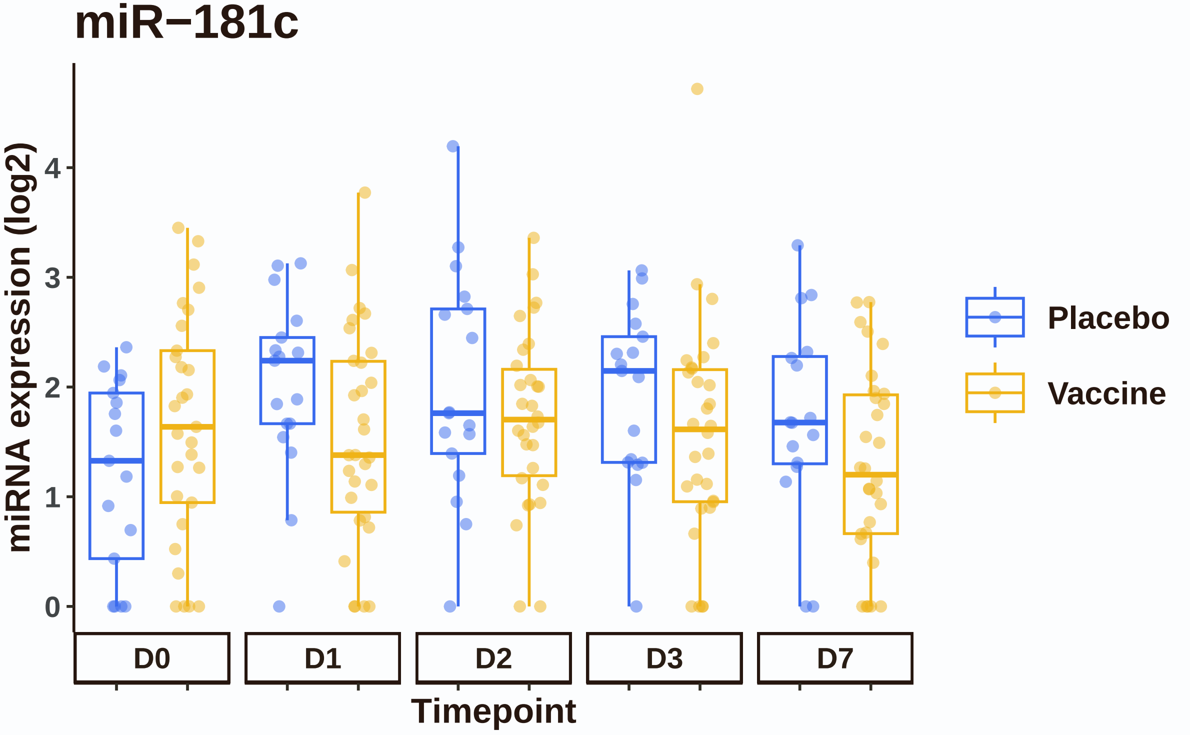
miR−181c
miRNA expression (log2)
0
1
2
3
4
D0
D1
D2
D3
D7
Timepoint
Placebo
Vaccine

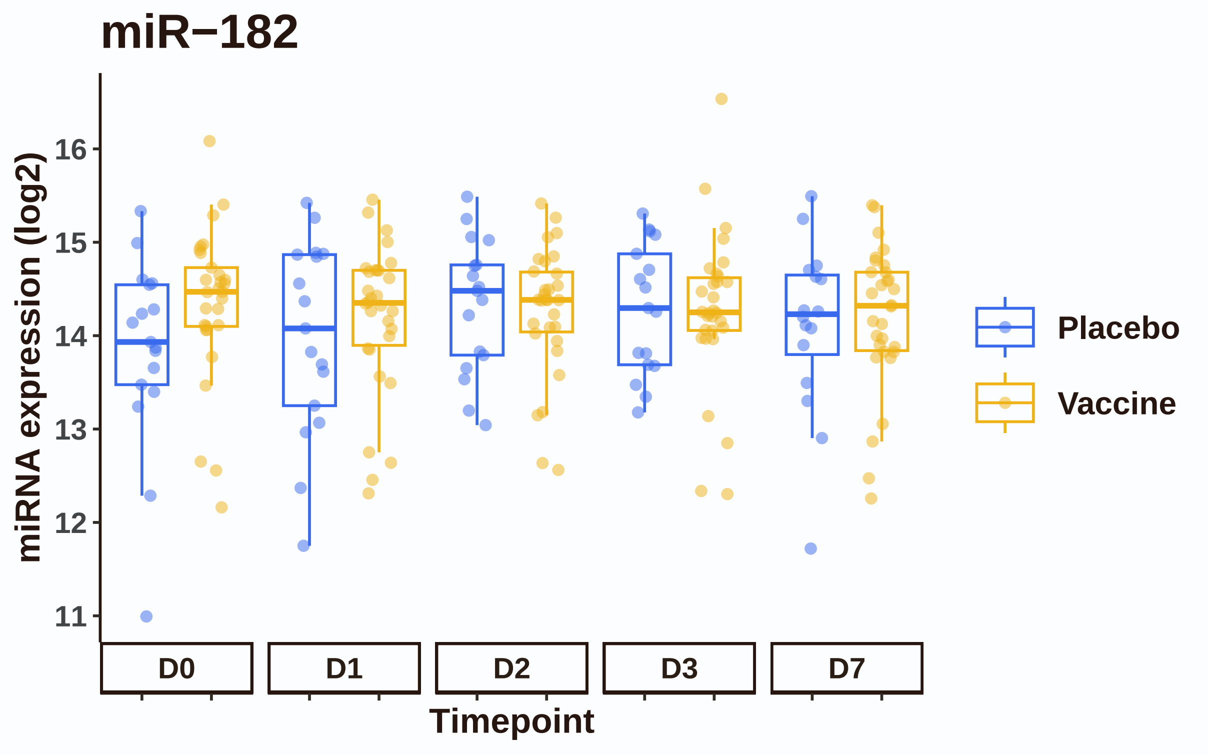
miR−182
miRNA expression (log2)
16
15
14
13
12
11
D0
D1
D2
D3
D7
Timepoint
Placebo
Vaccine

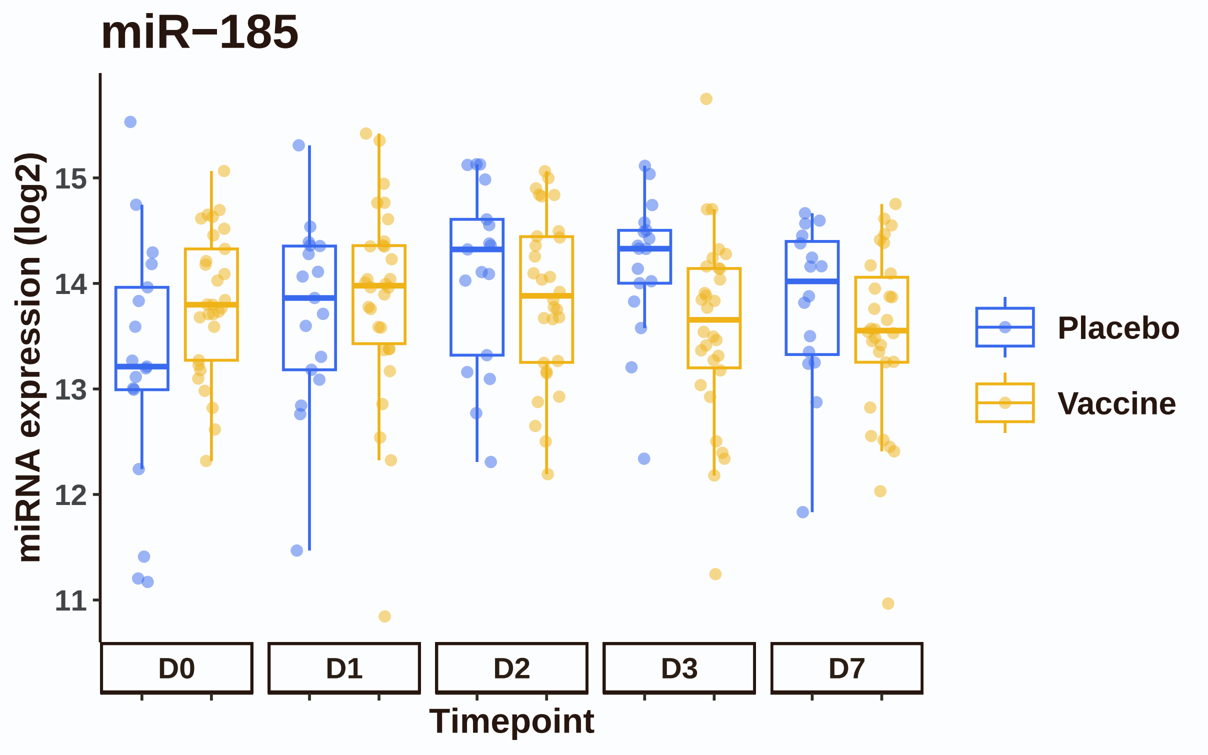
miR−185
miRNA expression (log2)
15
14
13
12
11
D0
D1
D2
D3
D7
Timepoint
Placebo
Vaccine

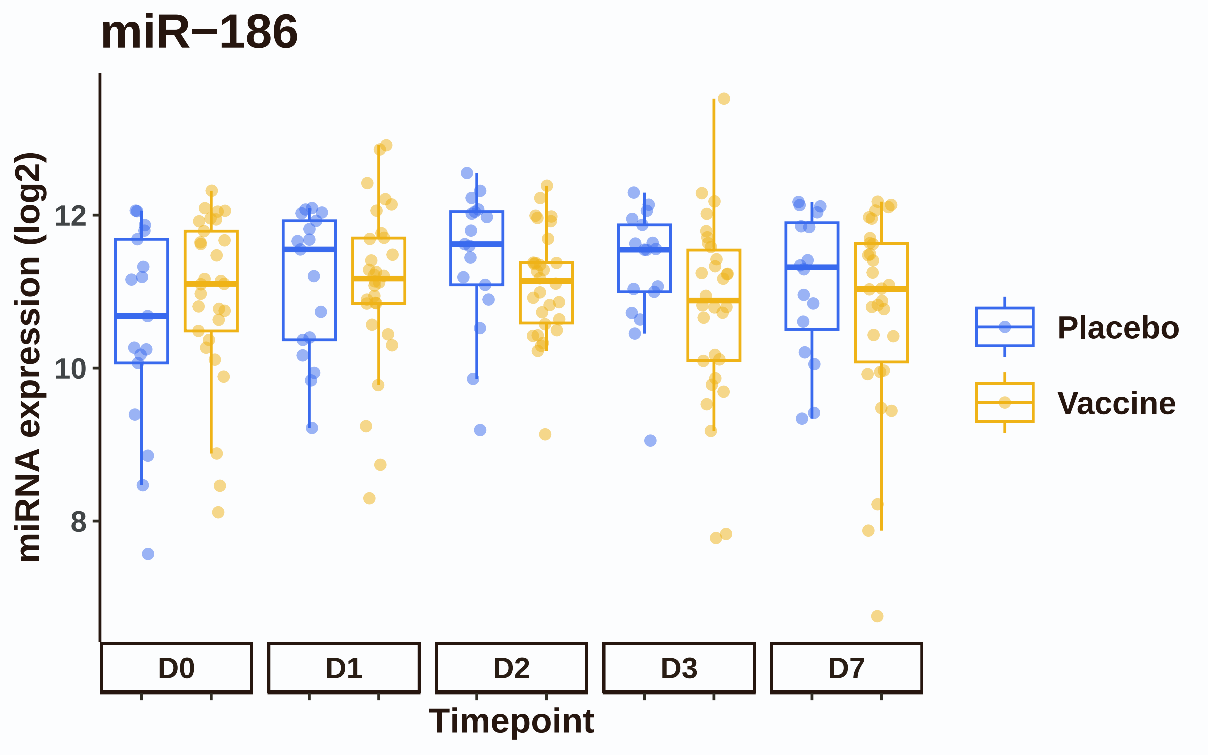

miR−186
miRNA expression (log2)
8
10
12
D0
D1
D2
D3
D7
Timepoint
Placebo
Vaccine

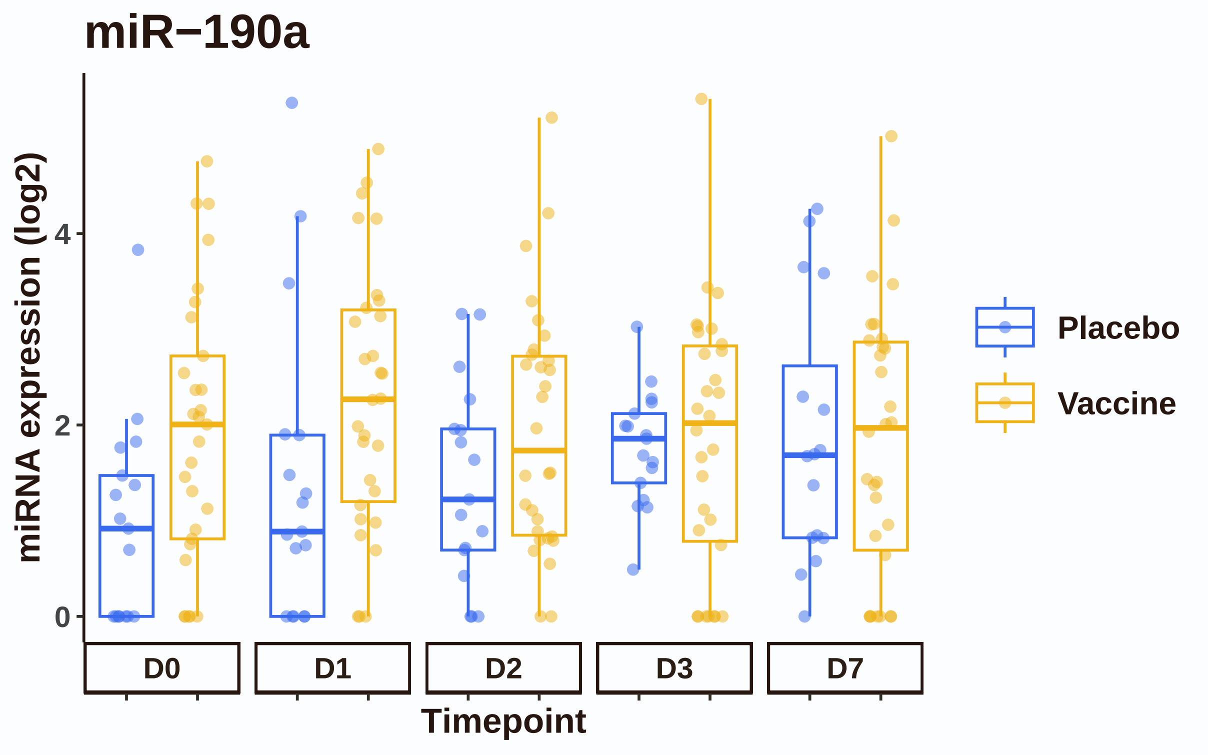

miR−190a
miRNA expression (log2)
0
2
4
D0
D1
D2
D3
D7
Timepoint
Placebo
Vaccine

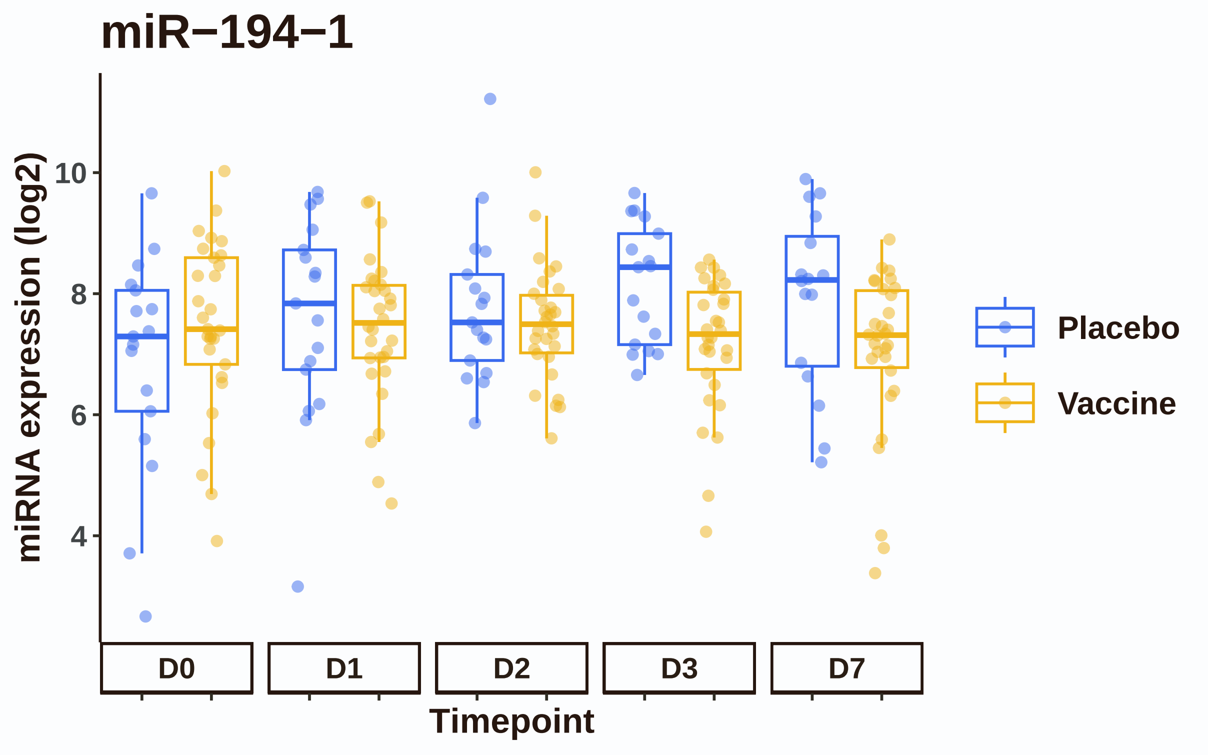
miR−194−1
miRNA expression (log2)
10
8
6
4
D0
D1
D2
D3
D7
Timepoint
Placebo
Vaccine

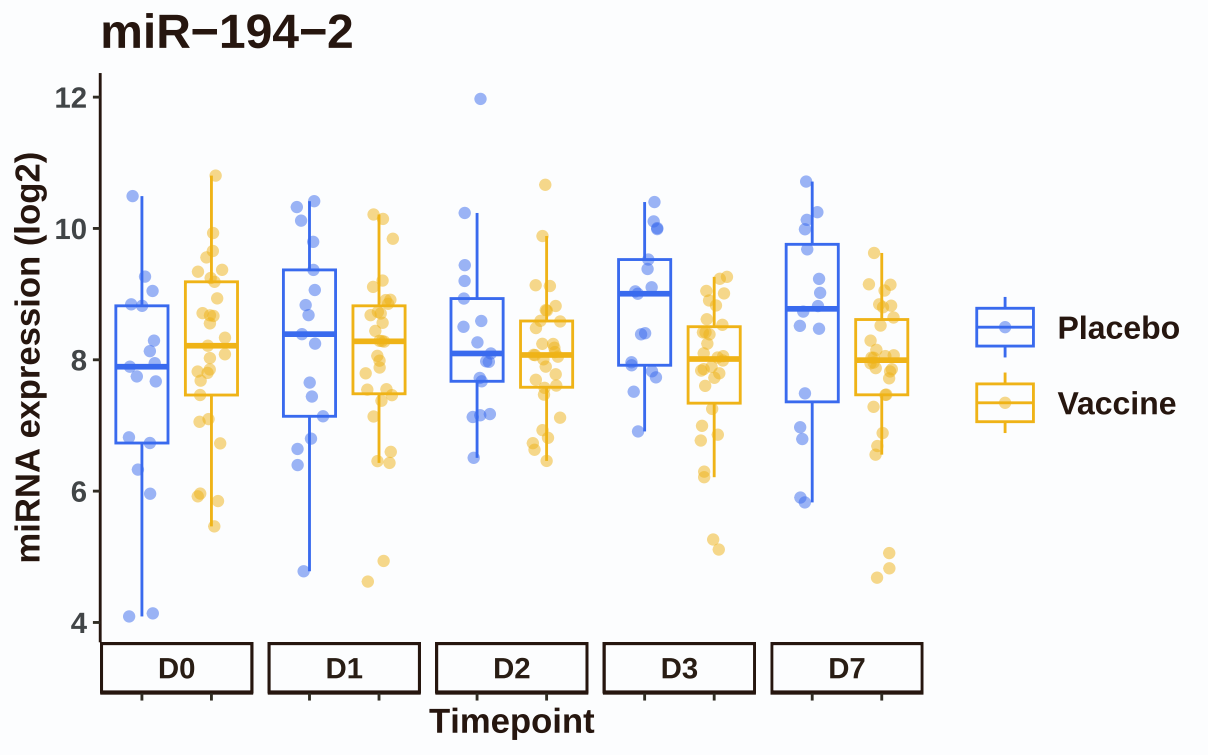
miR−194−2
miRNA expression (log2)
12
10
8
6
4
D0
D1
D2
D3
D7
Timepoint
Placebo
Vaccine

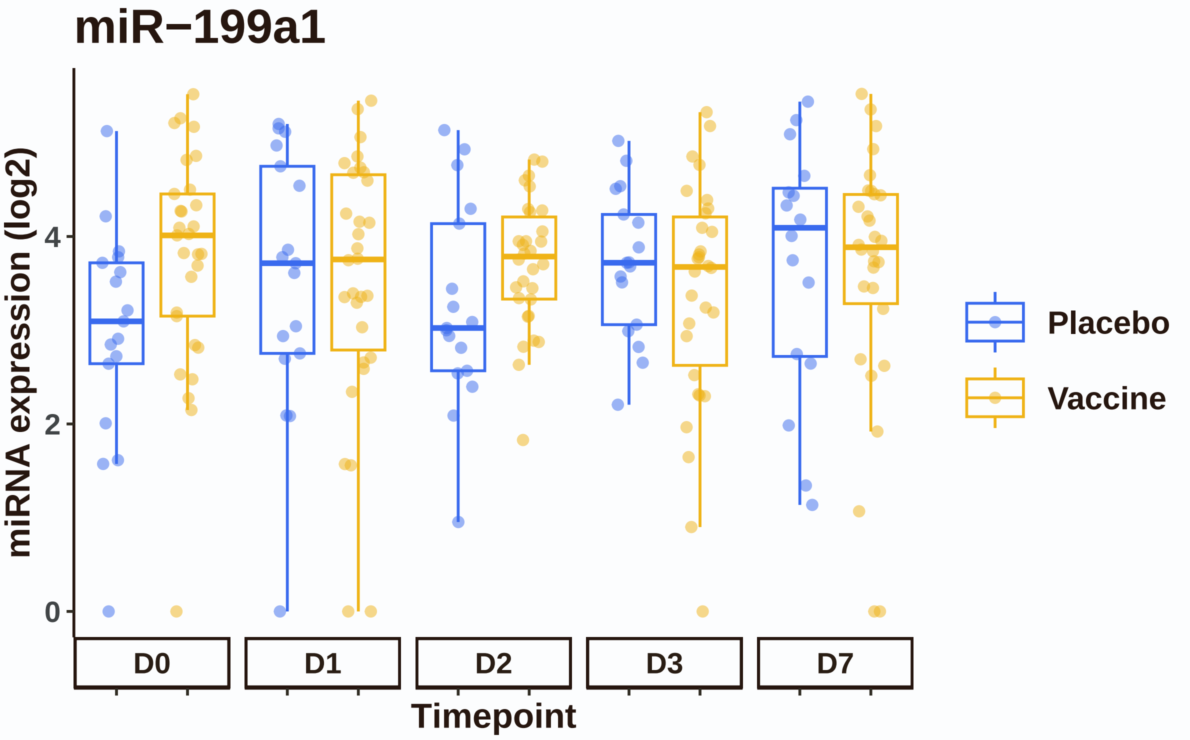
miR−199a1
miRNA expression (log2)
0
2
4
D0
D1
D2
D3
D7
Timepoint
Placebo
Vaccine

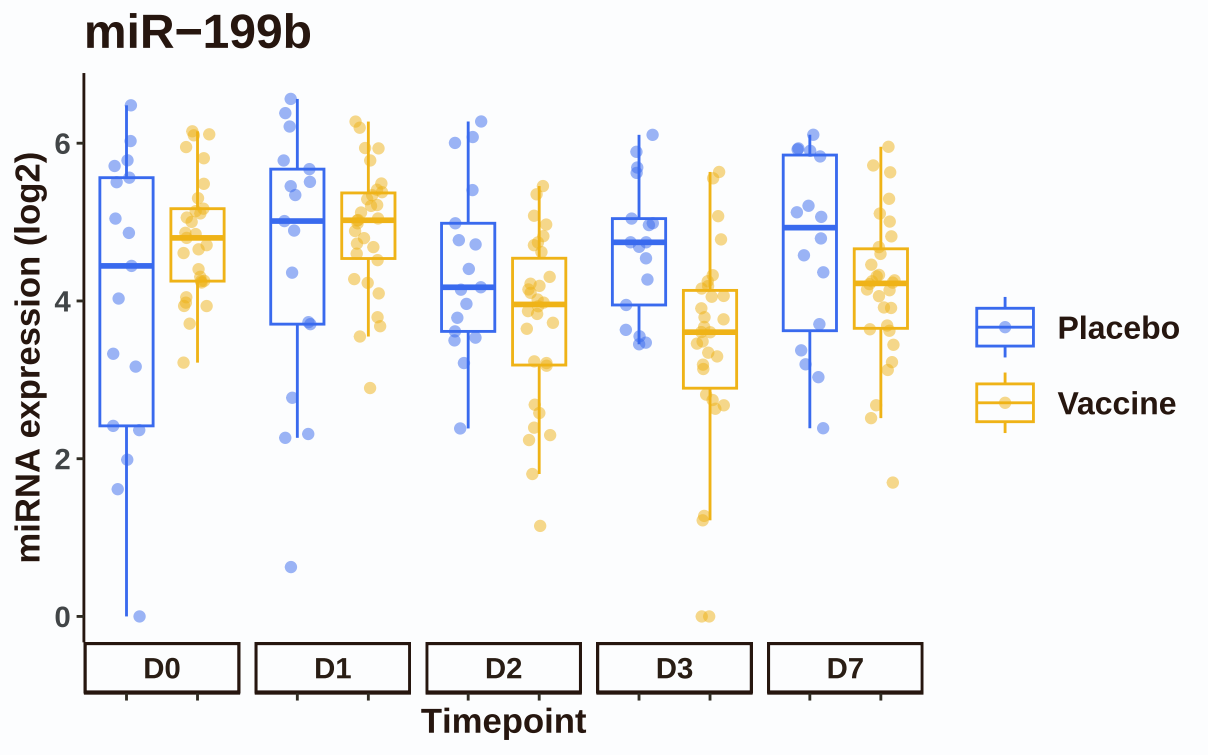
miR−199b
miRNA expression (log2)
0
2
4
6
D0
D1
D2
D3
D7
Timepoint
Placebo
Vaccine

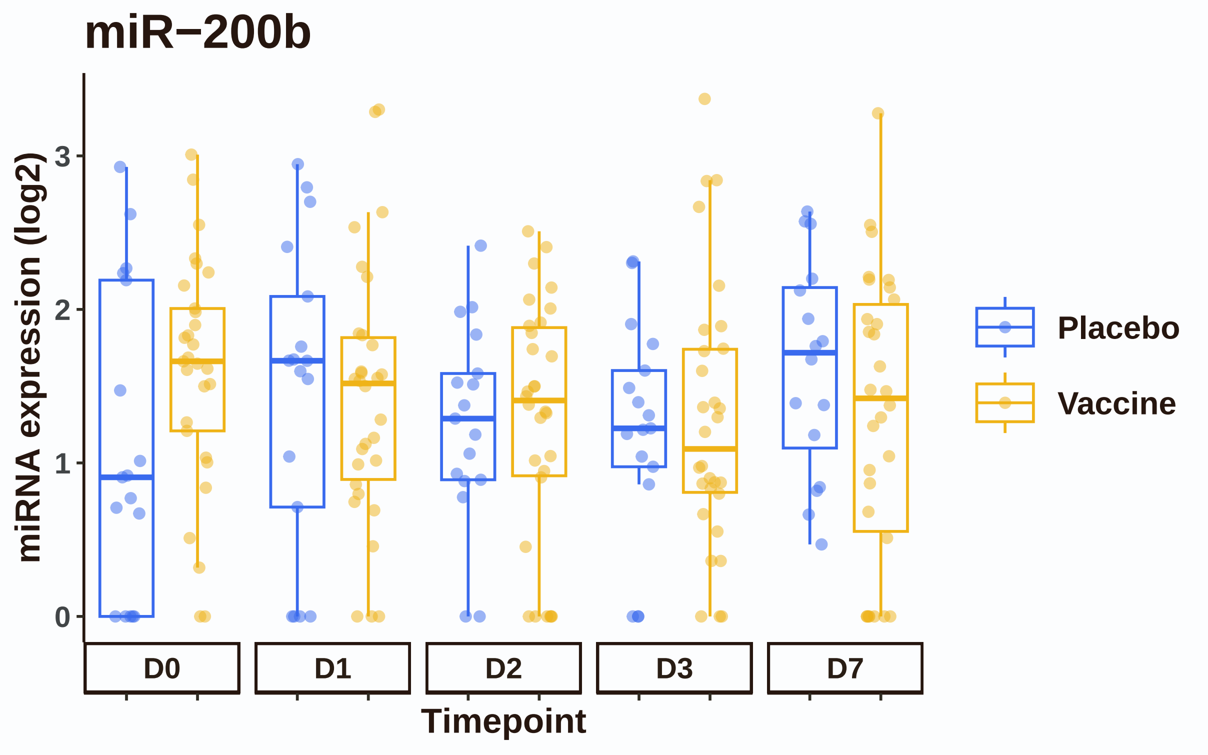
miR−200b
miRNA expression (log2)
0
1
2
3
D0
D1
D2
D3
D7
Timepoint
Placebo
Vaccine

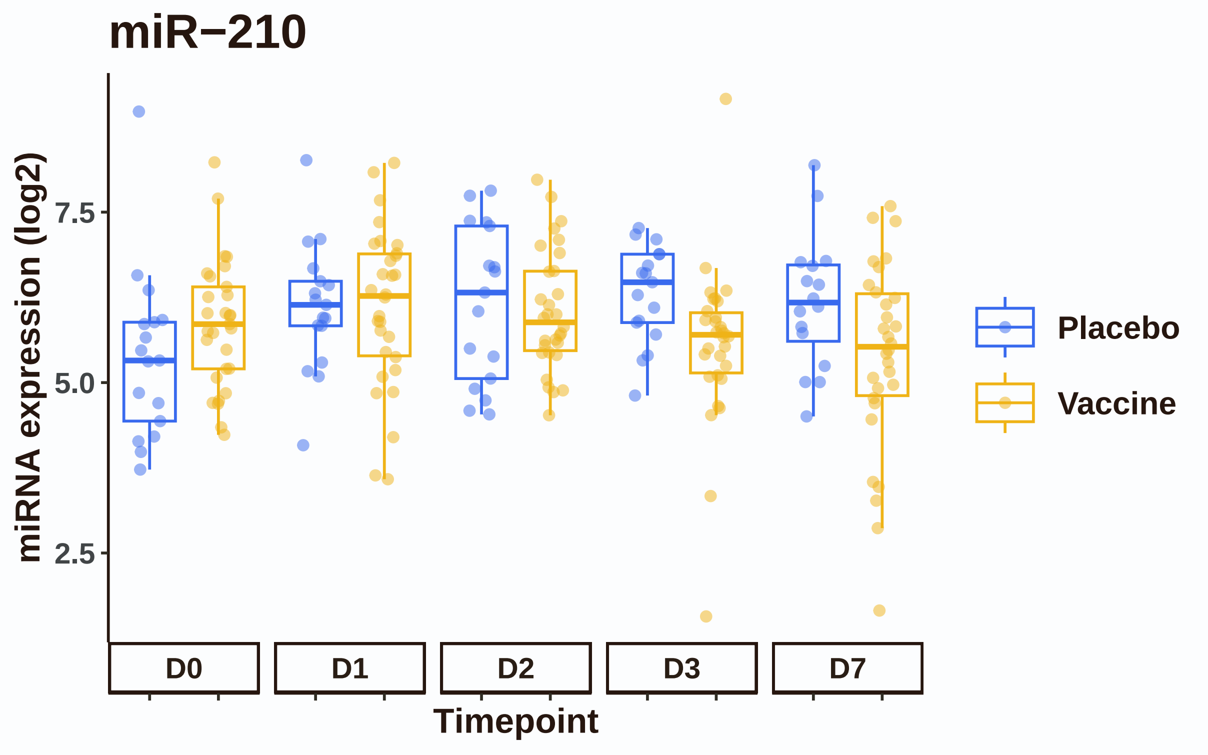

miR−210
miRNA expression (log2)
7.5
5.0
2.5
D0
D1
D2
D3
D7
Timepoint
Placebo
Vaccine

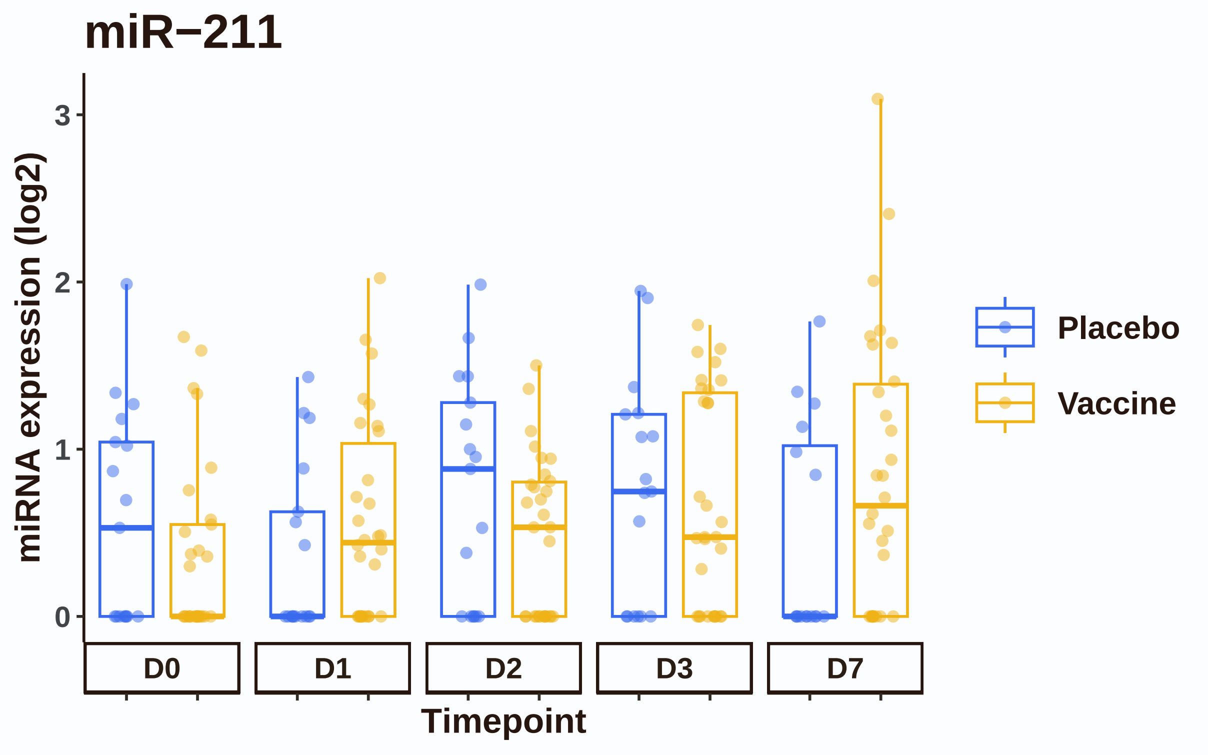
miR−211
miRNA expression (log2)
3
2
1
0
D0
D1
D2
D3
D7
Timepoint
Placebo
Vaccine

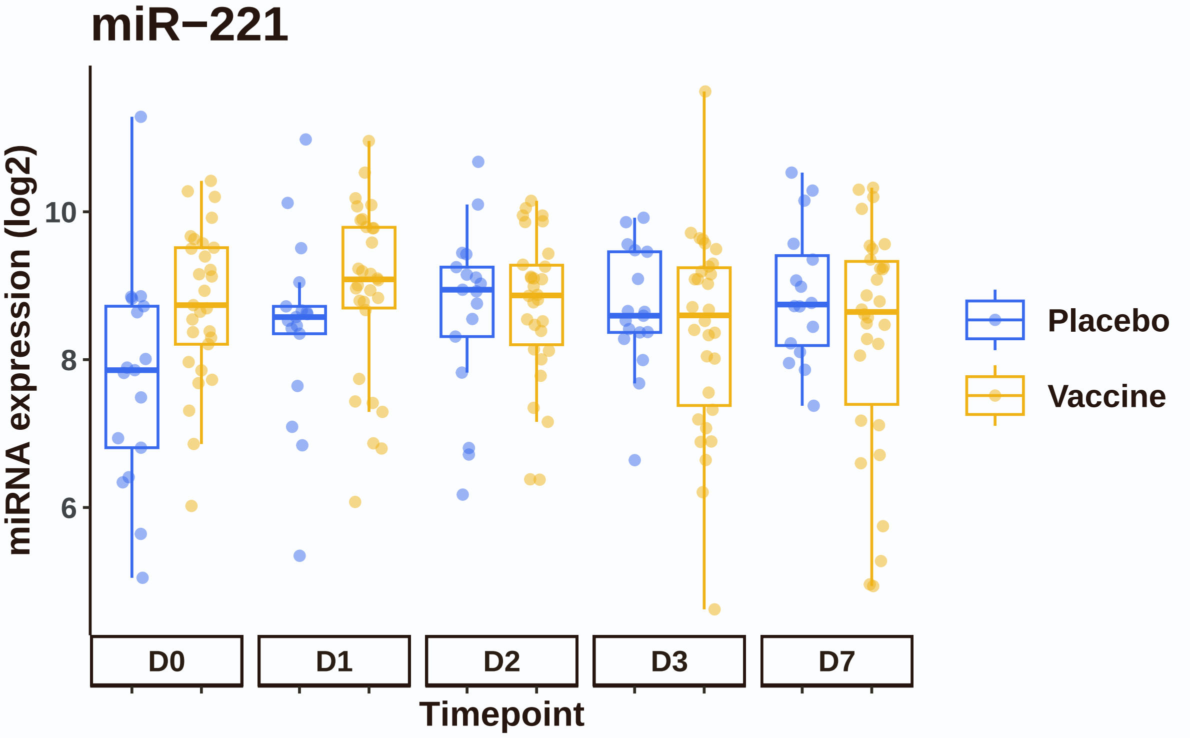
miR−221
miRNA expression (log2)
10
8
6
D0
D1
D2
D3
D7
Timepoint
Placebo
Vaccine

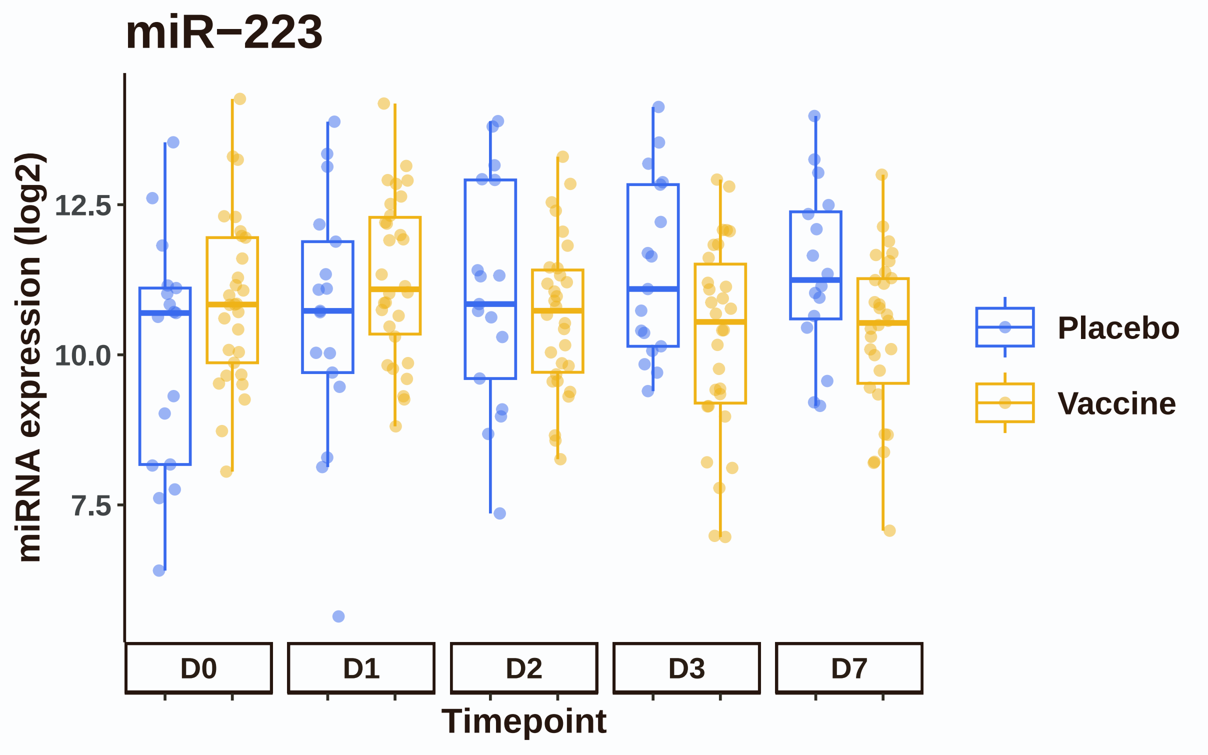

miR−223
miRNA expression (log2)
12.5
10.0
7.5
D0
D1
D2
D3
D7
Timepoint
Placebo
Vaccine

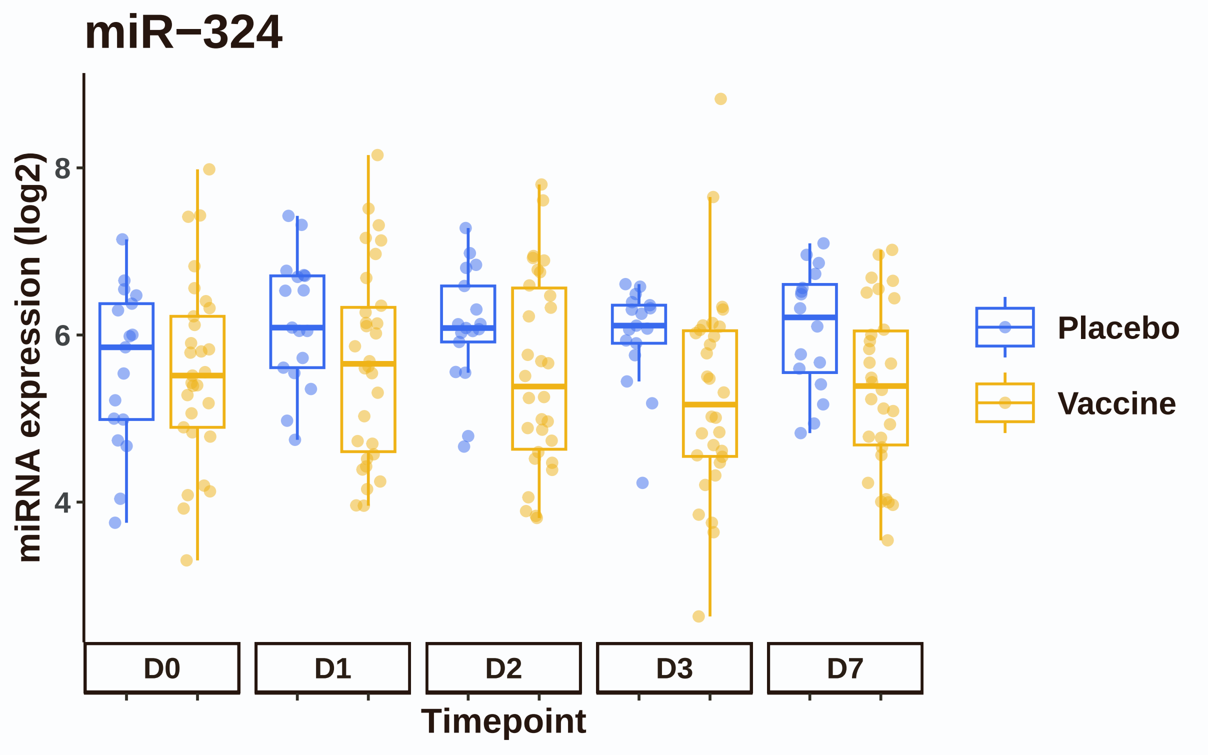

miR−324
miRNA expression (log2)
8
6
4
D0
D1
D2
D3
D7
Timepoint
Placebo
Vaccine

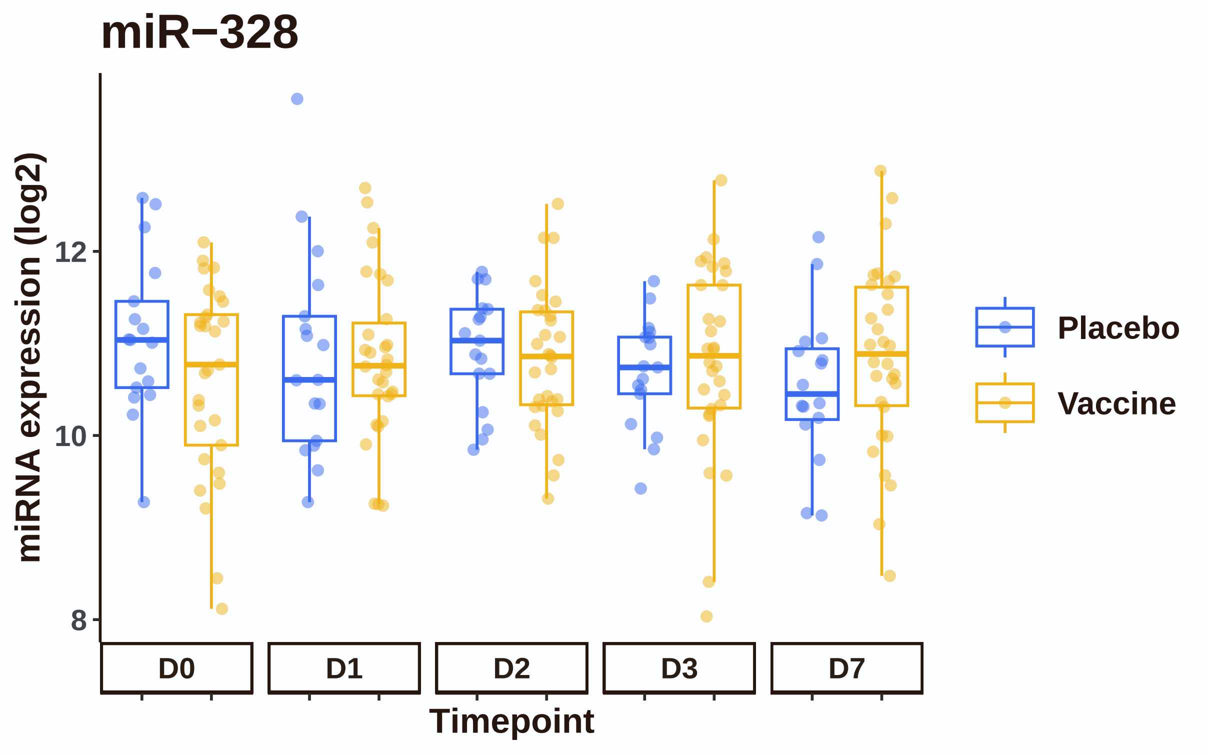
miR−328
miRNA expression (log2)
12
10
8
D0
D1
D2
D3
D7
Timepoint
Placebo
Vaccine

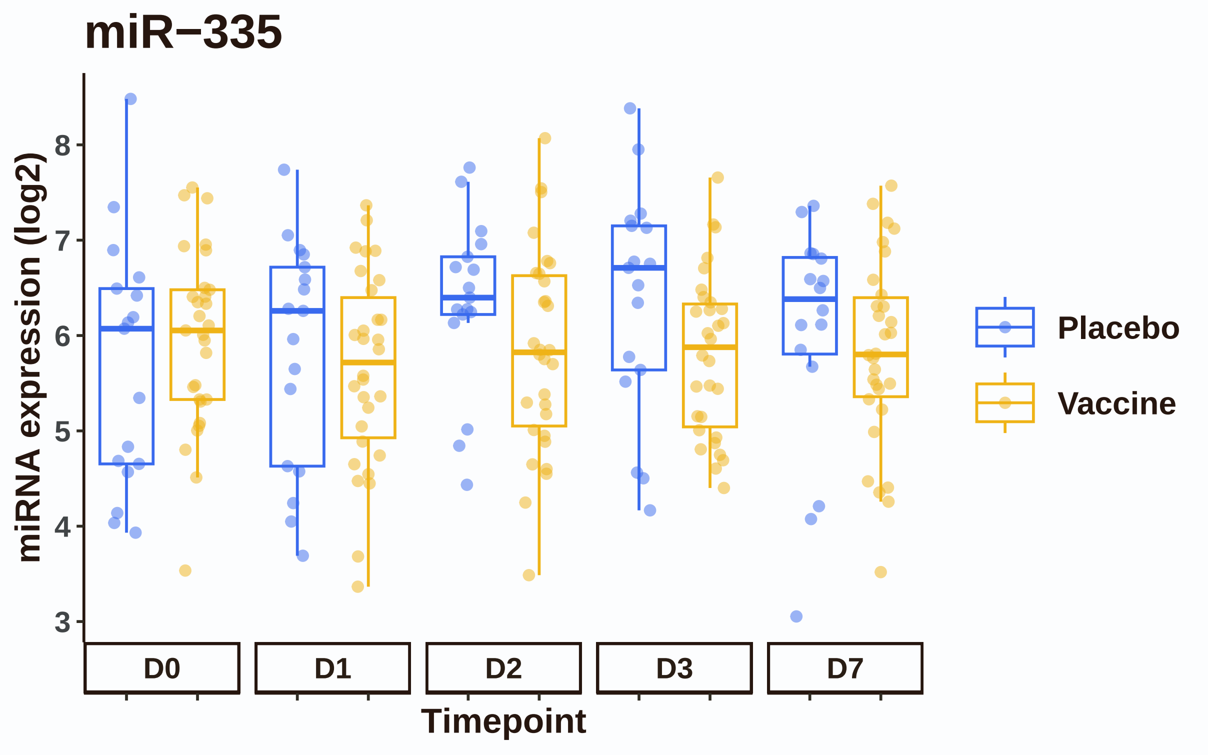
miR−335
miRNA expression (log2)
8
7
6
5
4
3
D0
D1
D2
D3
D7
Timepoint
Placebo
Vaccine

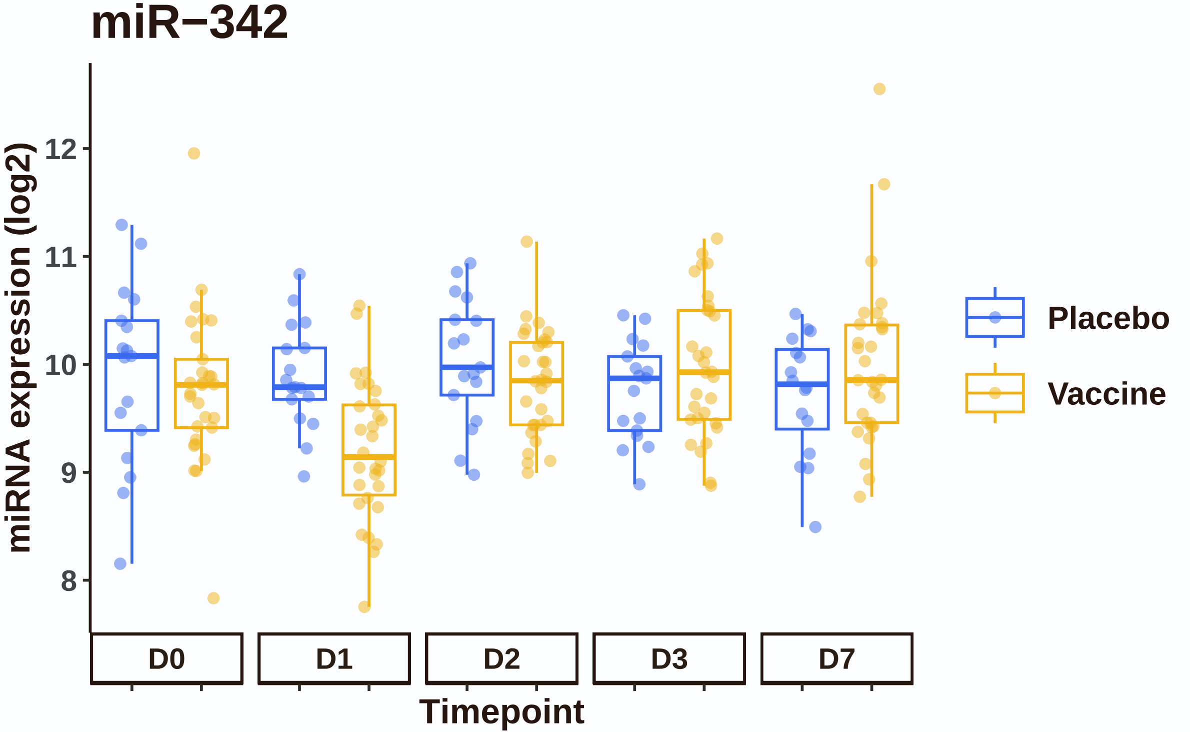

miR−342
miRNA expression (log2)
12
11
10
9
8
D0
D1
D2
D3
D7
Timepoint
Placebo
Vaccine

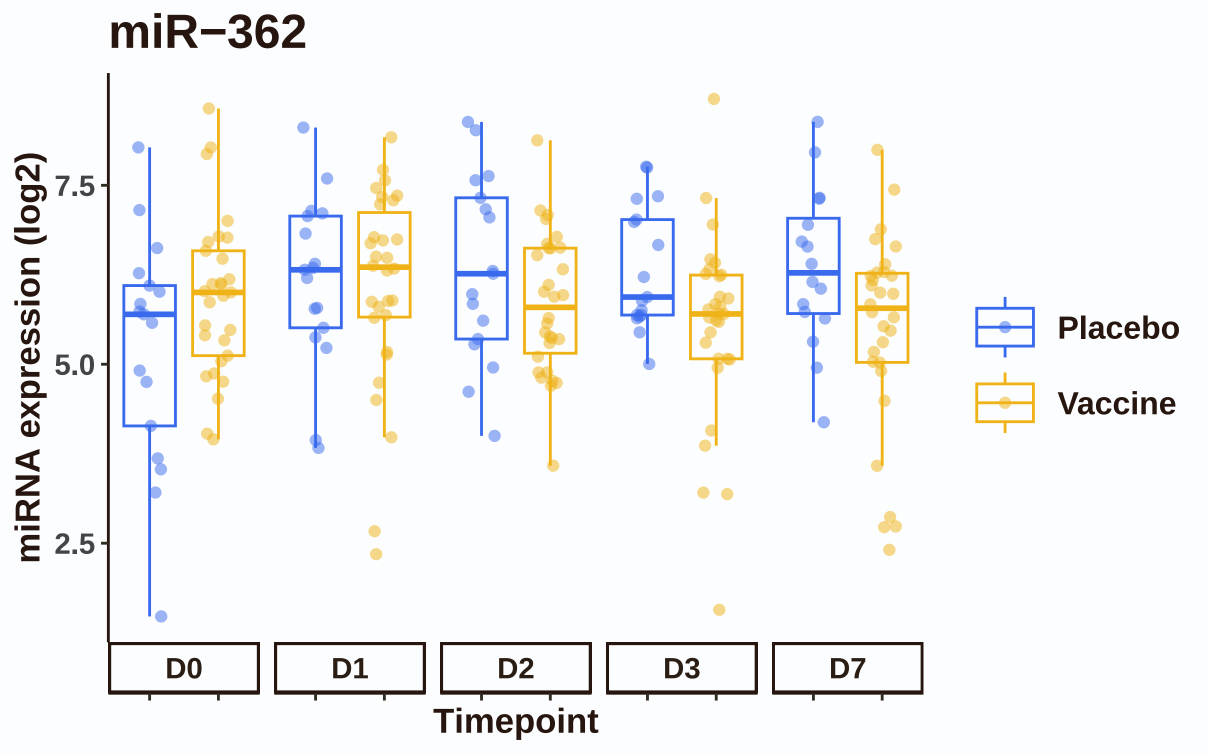
miR−362
miRNA expression (log2)
7.5
5.0
2.5
D0
D1
D2
D3
D7
Timepoint
Placebo
Vaccine

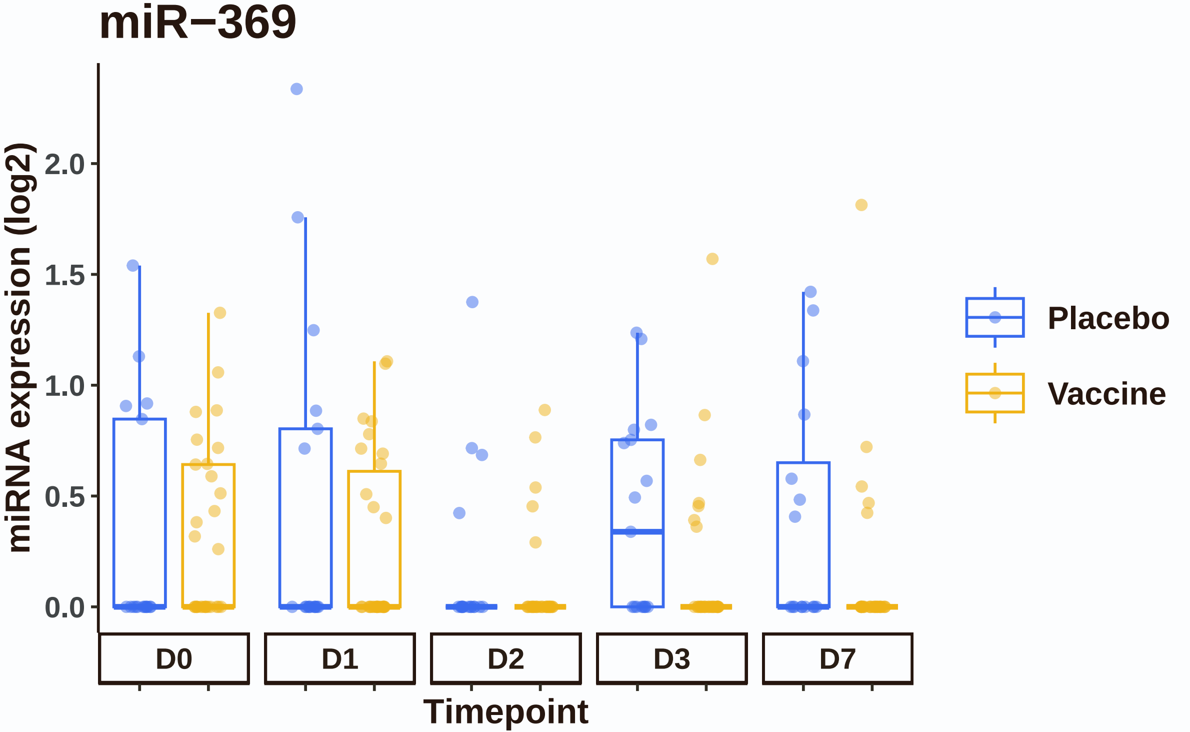
miR−369
miRNA expression (log2)
2.0
1.5
1.0
0.5
0.0
D0
D1
D2
D3
D7
Timepoint
Placebo
Vaccine

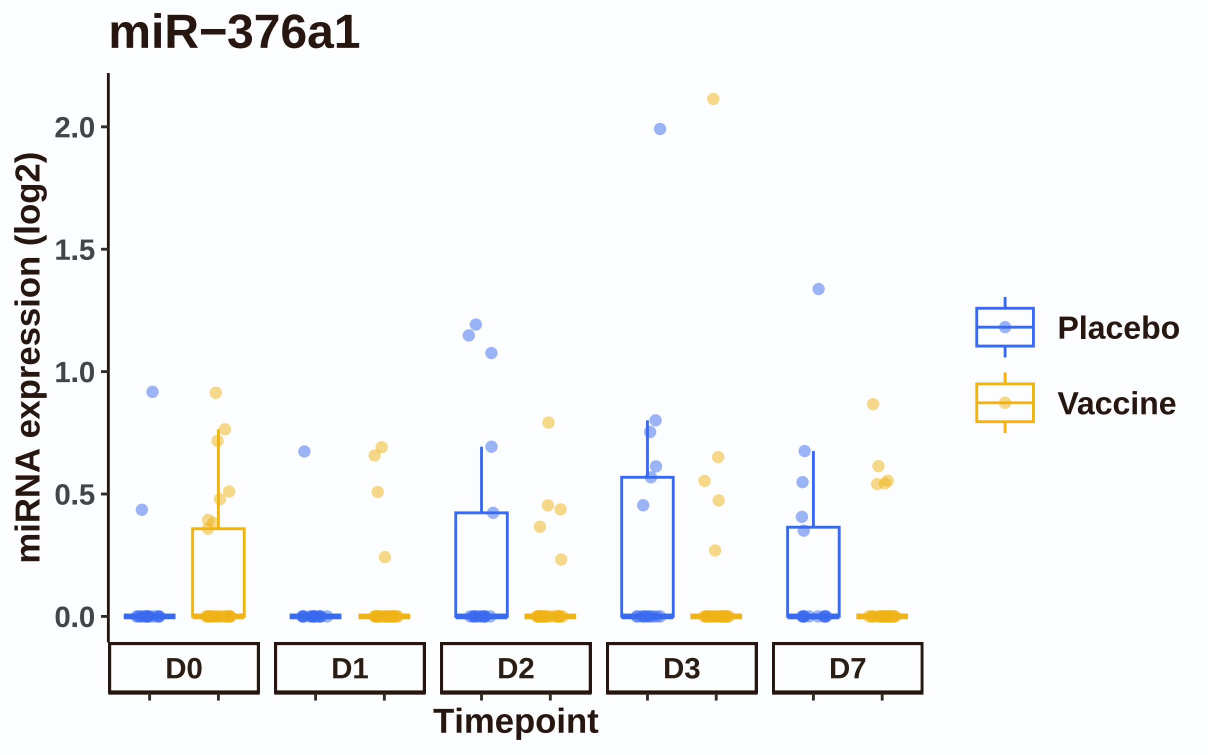

miR−376a1
miRNA expression (log2)
2.0
1.5
1.0
0.5
0.0
D0
D1
D2
D3
D7
Timepoint
Placebo
Vaccine

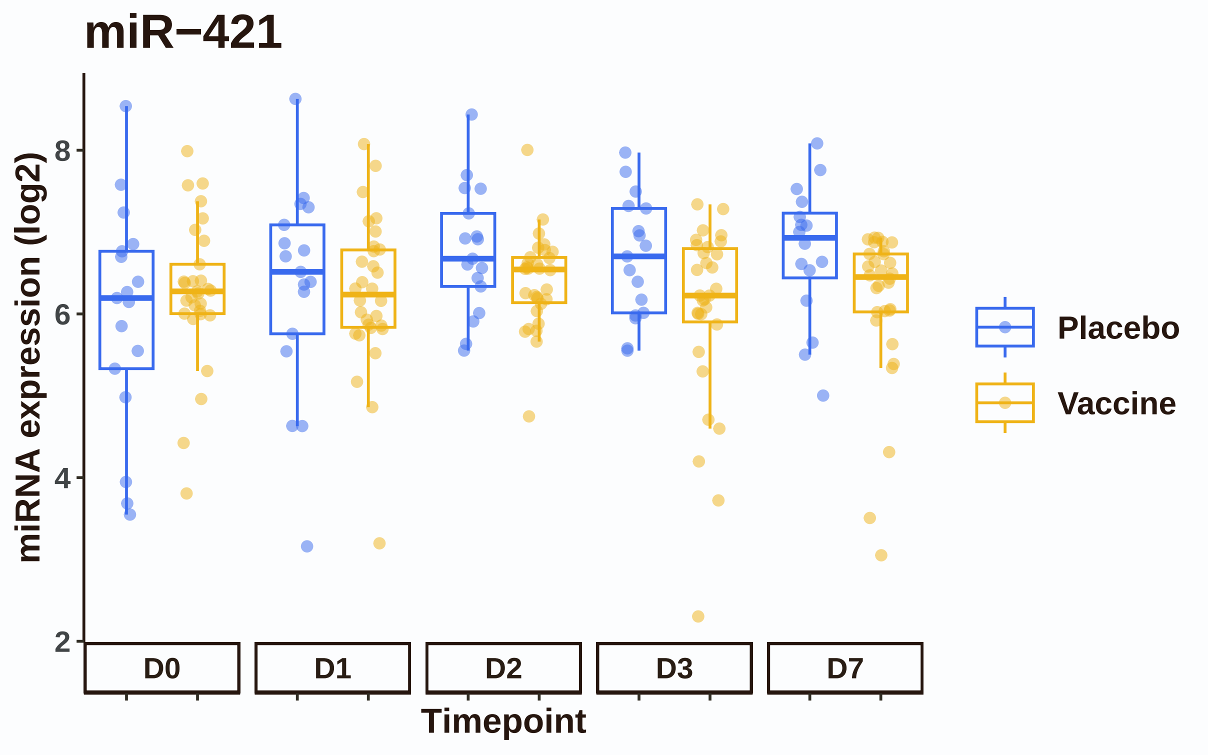
miR−421
miRNA expression (log2)
8
6
4
2
D0
D1
D2
D3
D7
Timepoint
Placebo
Vaccine

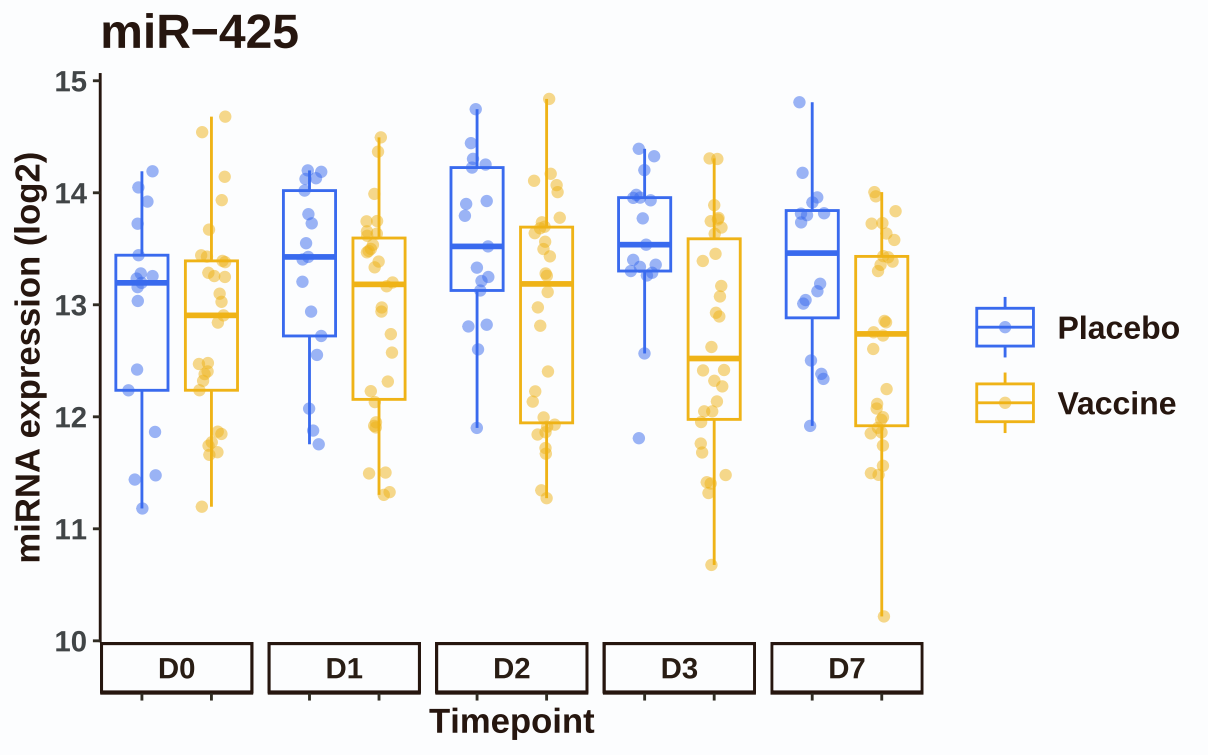

miR−425
miRNA expression (log2)
15
14
13
12
11
10
D0
D1
D2
D3
D7
Timepoint
Placebo
Vaccine

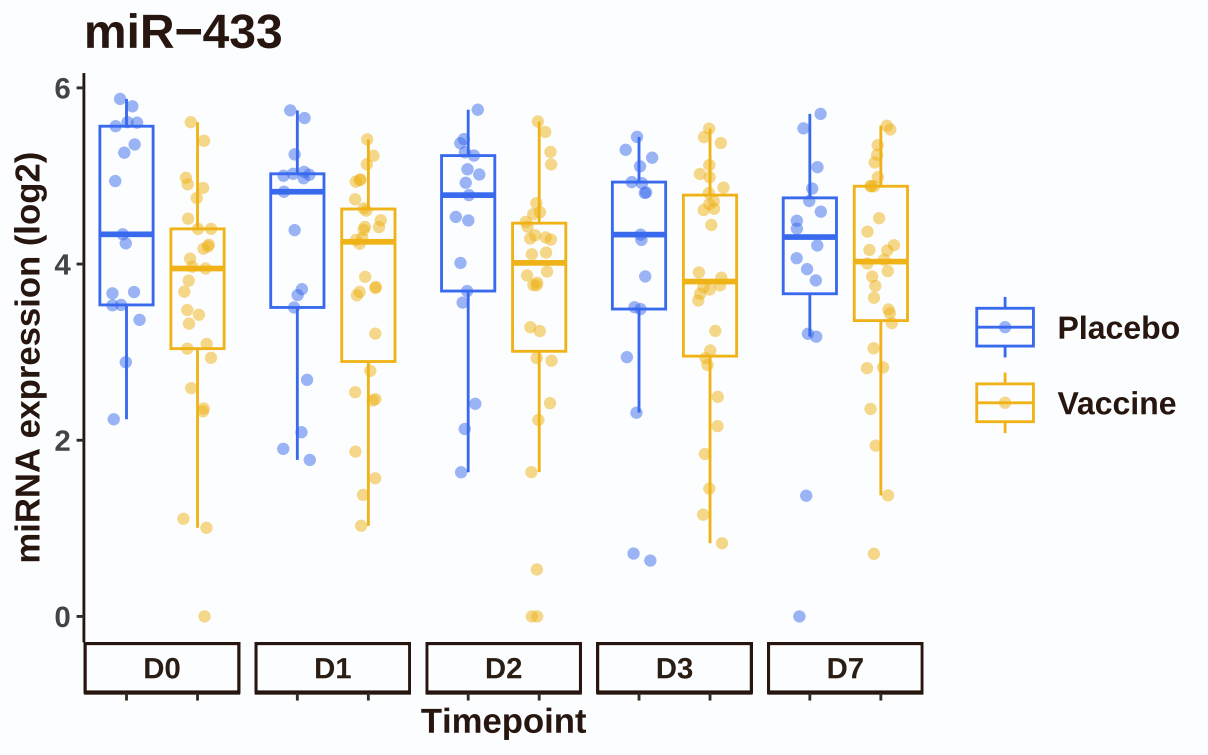

miR−433
miRNA expression (log2)
6
4
2
0
D0
D1
D2
D3
D7
Timepoint
Placebo
Vaccine

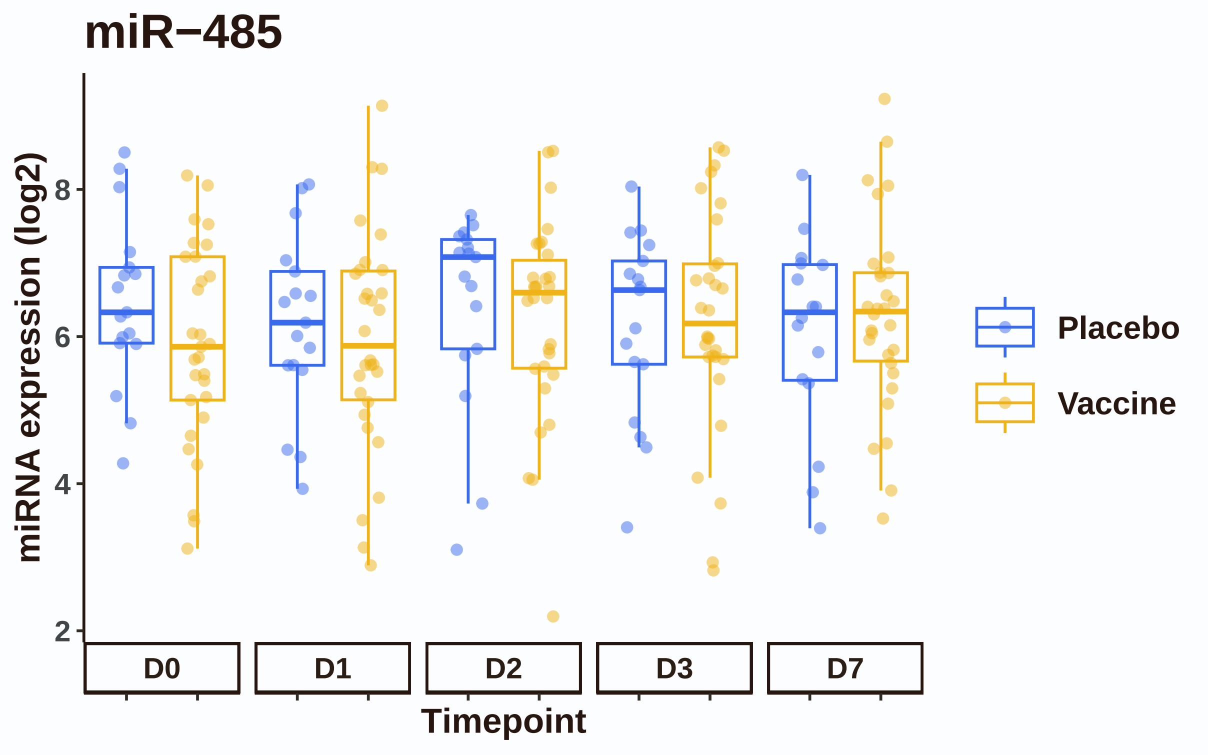
miR−485
miRNA expression (log2)
8
6
4
2
D0
D1
D2
D3
D7
Timepoint
Placebo
Vaccine

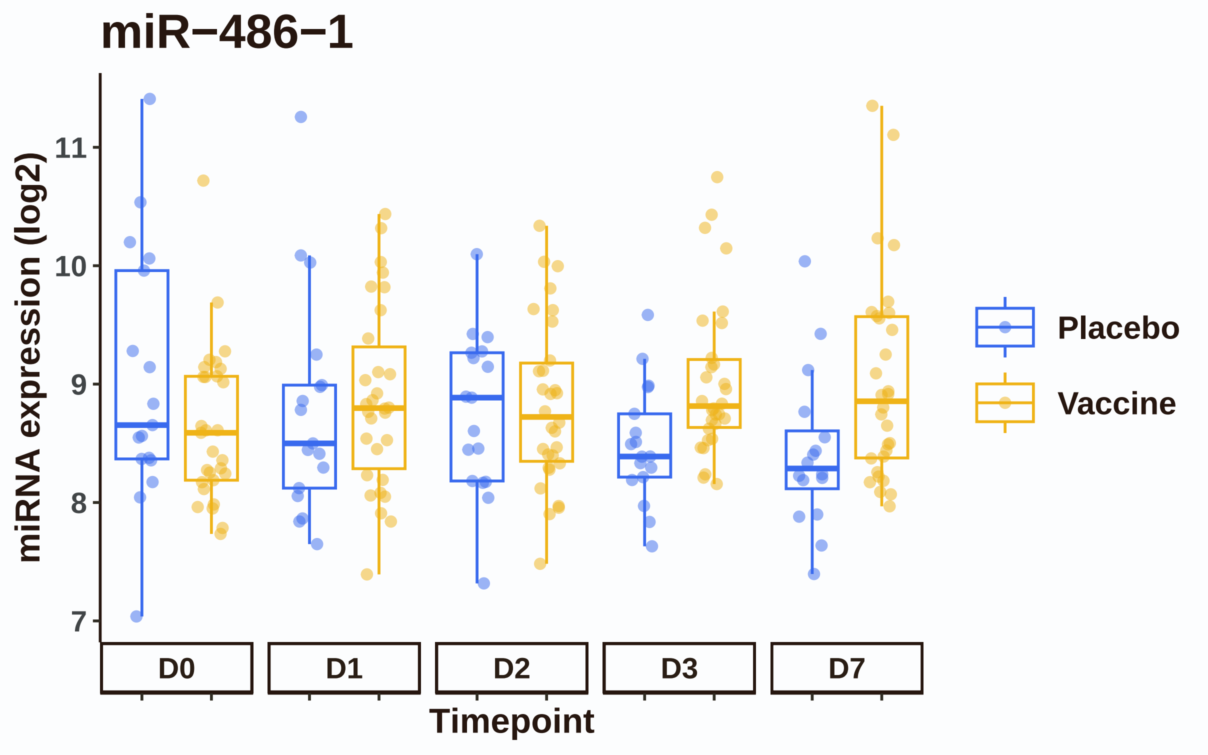
miR−486−1
miRNA expression (log2)
11
10
9
8
7
D0
D1
D2
D3
D7
Timepoint
Placebo
Vaccine

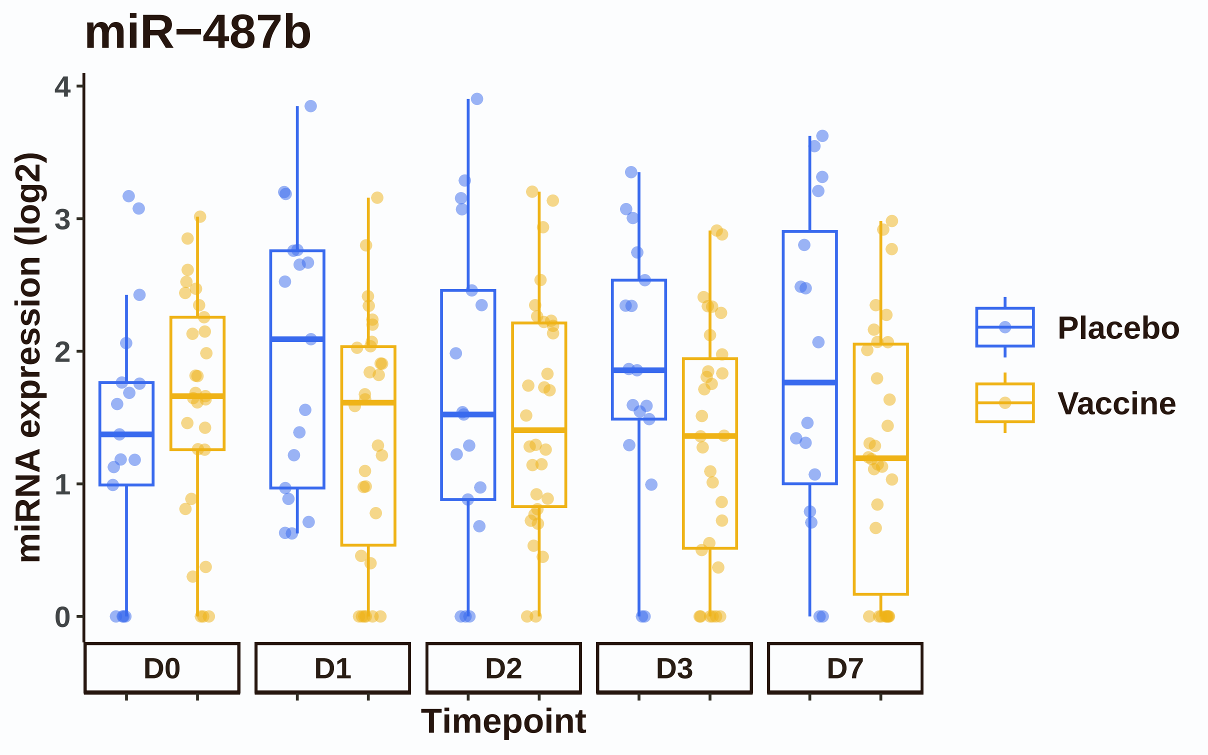
miR−487b
miRNA expression (log2)
4
3
2
1
0
D0
D1
D2
D3
D7
Timepoint
Placebo
Vaccine

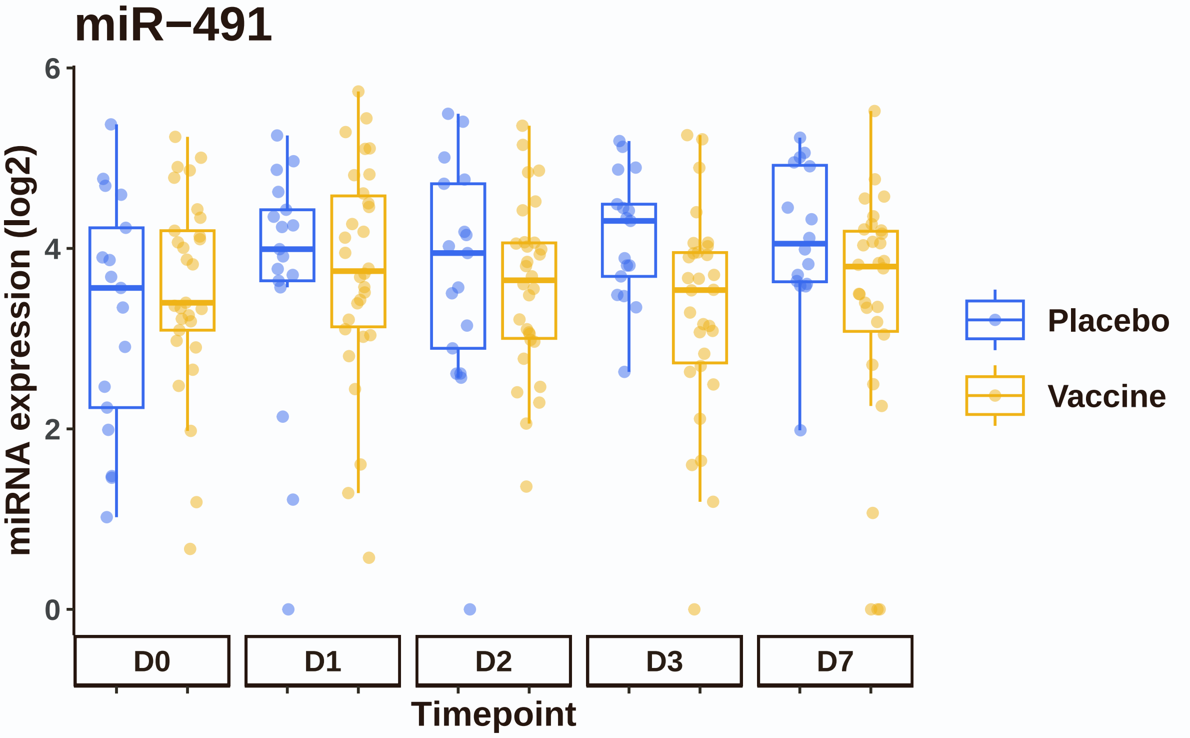
miR−491
miRNA expression (log2)
6
4
2
0
D0
D1
D2
D3
D7
Timepoint
Placebo
Vaccine

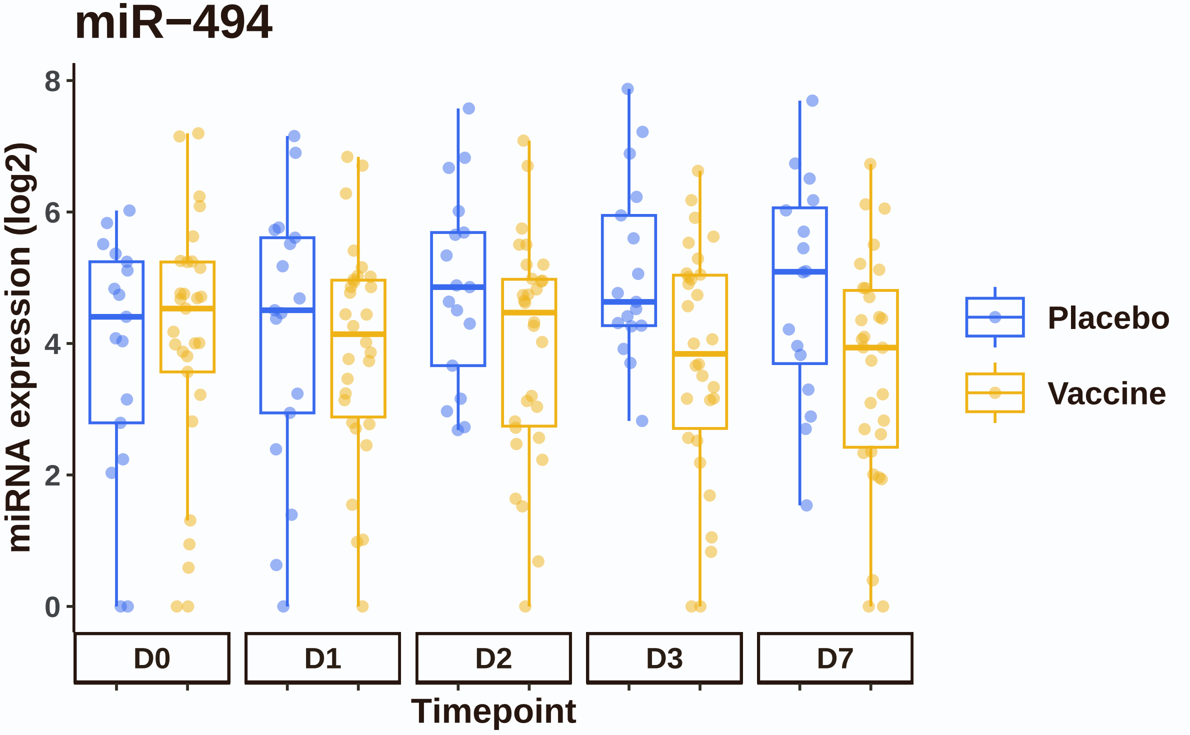

miR−494
miRNA expression (log2)
8
6
4
2
0
D0
D1
D2
D3
D7
Timepoint
Placebo
Vaccine

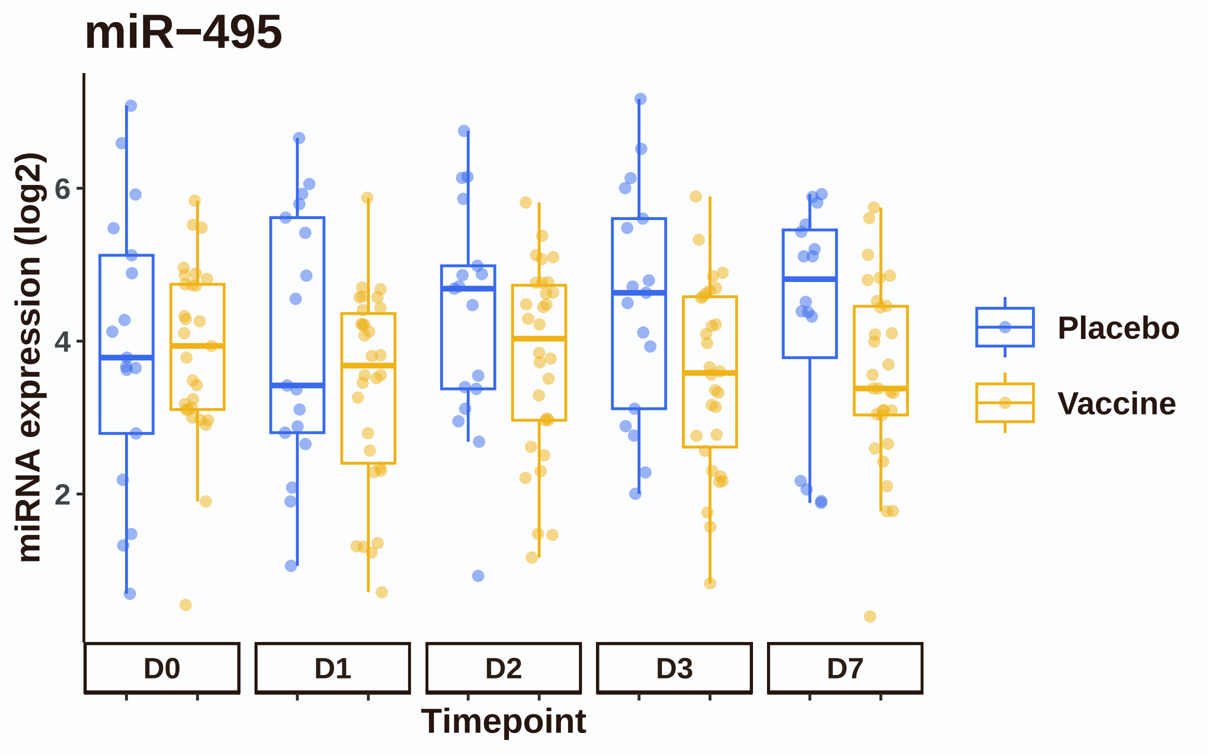
miR−495
miRNA expression (log2)
6
4
2
D0
D1
D2
D3
D7
Timepoint
Placebo
Vaccine

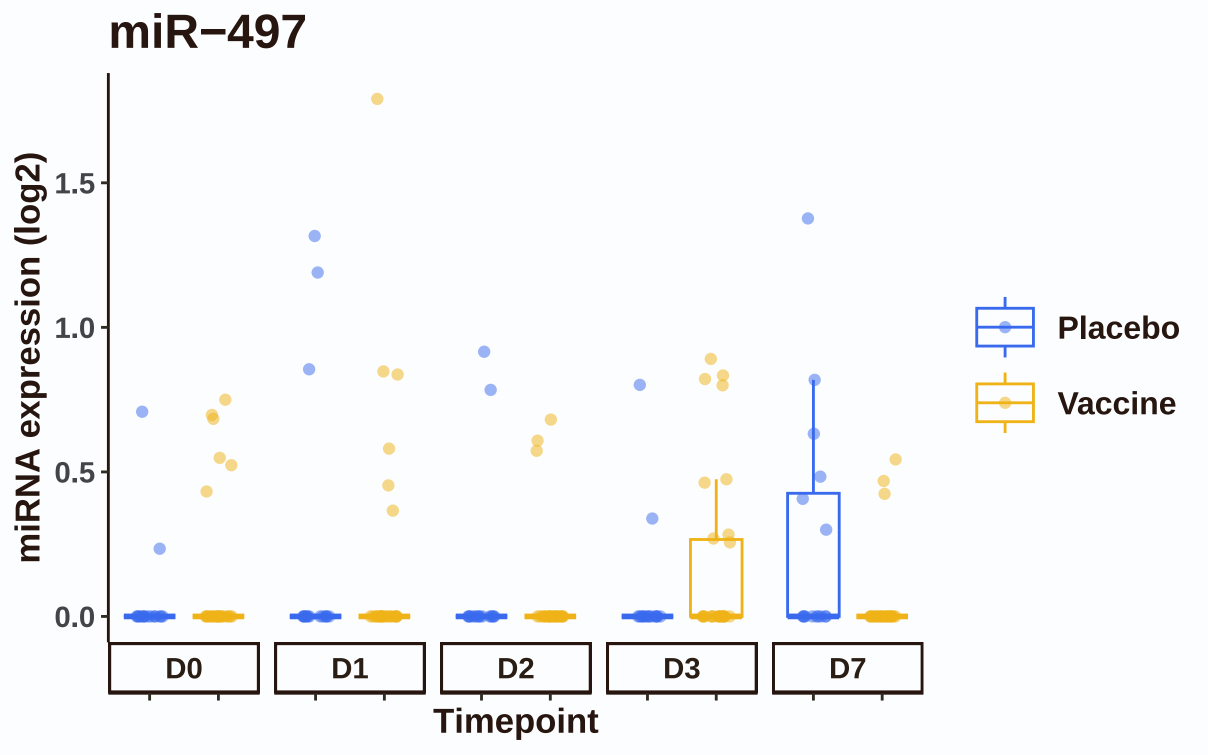
miR−497
miRNA expression (log2)
1.5
1.0
0.5
0.0
D0
D1
D2
D3
D7
Timepoint
Placebo
Vaccine

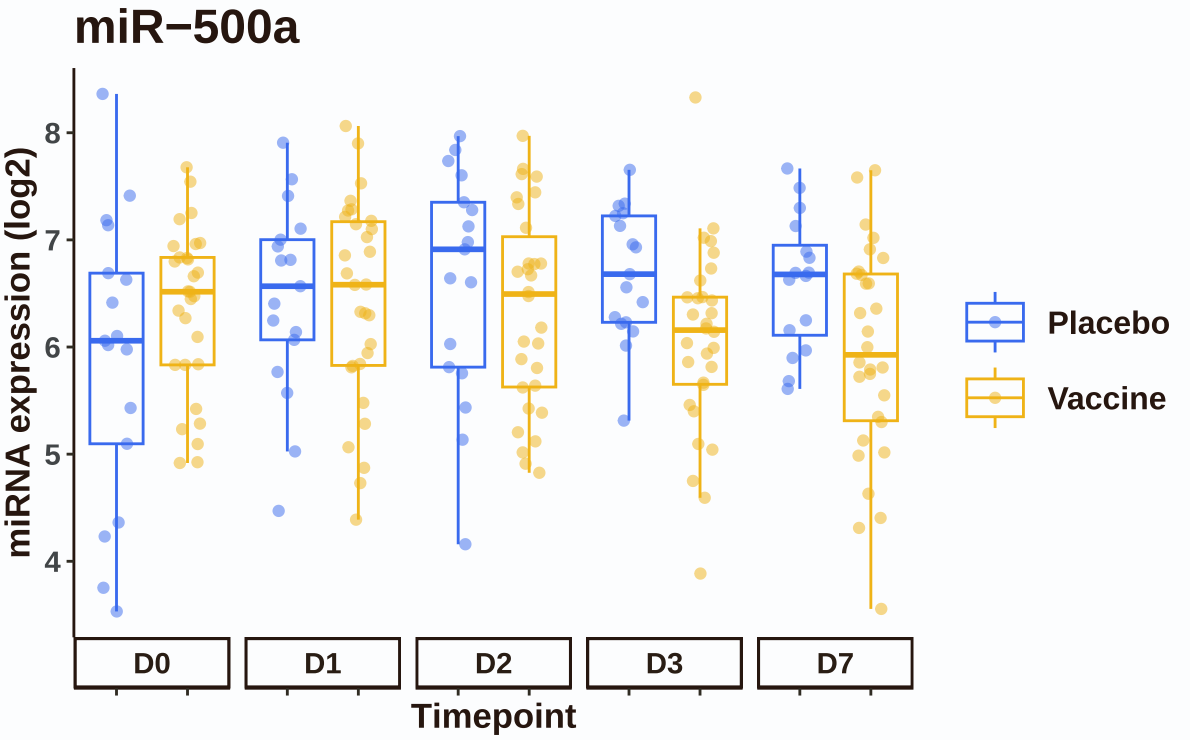
miR−500a
miRNA expression (log2)
8
7
6
5
4
D0
D1
D2
D3
D7
Timepoint
Placebo
Vaccine

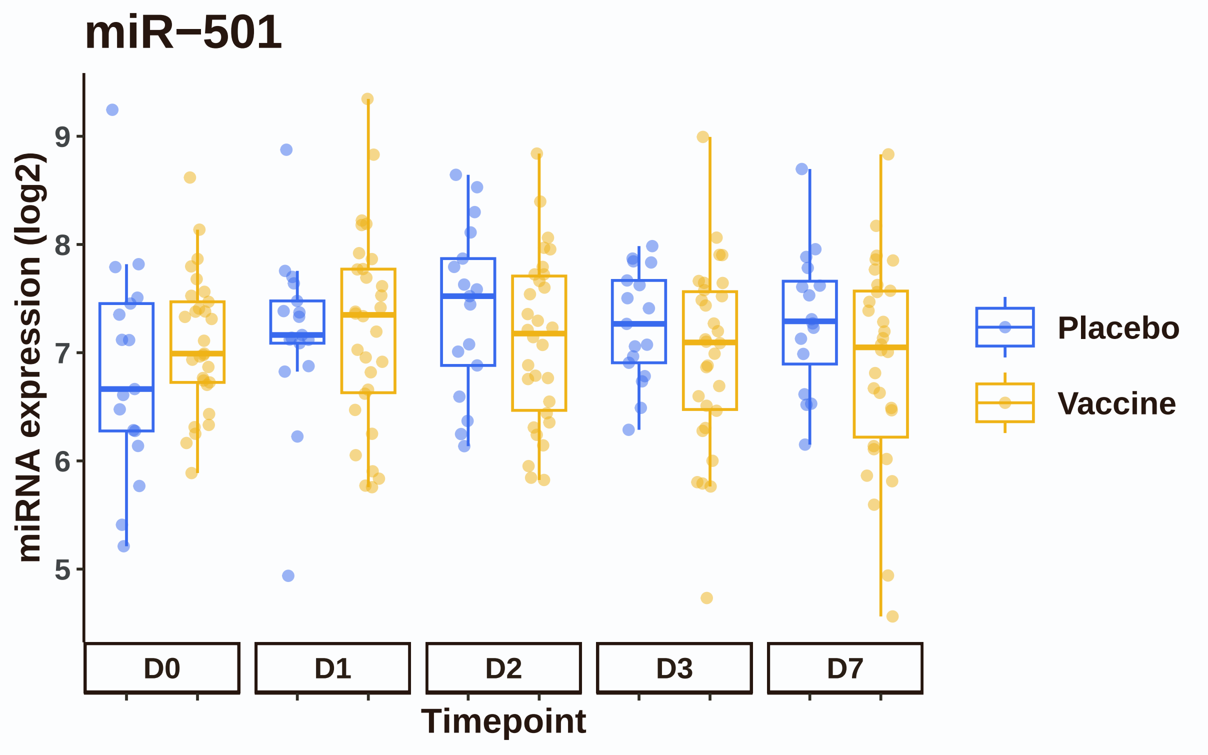

miR−501
miRNA expression (log2)
9
8
7
6
5
D0
D1
D2
D3
D7
Timepoint
Placebo
Vaccine

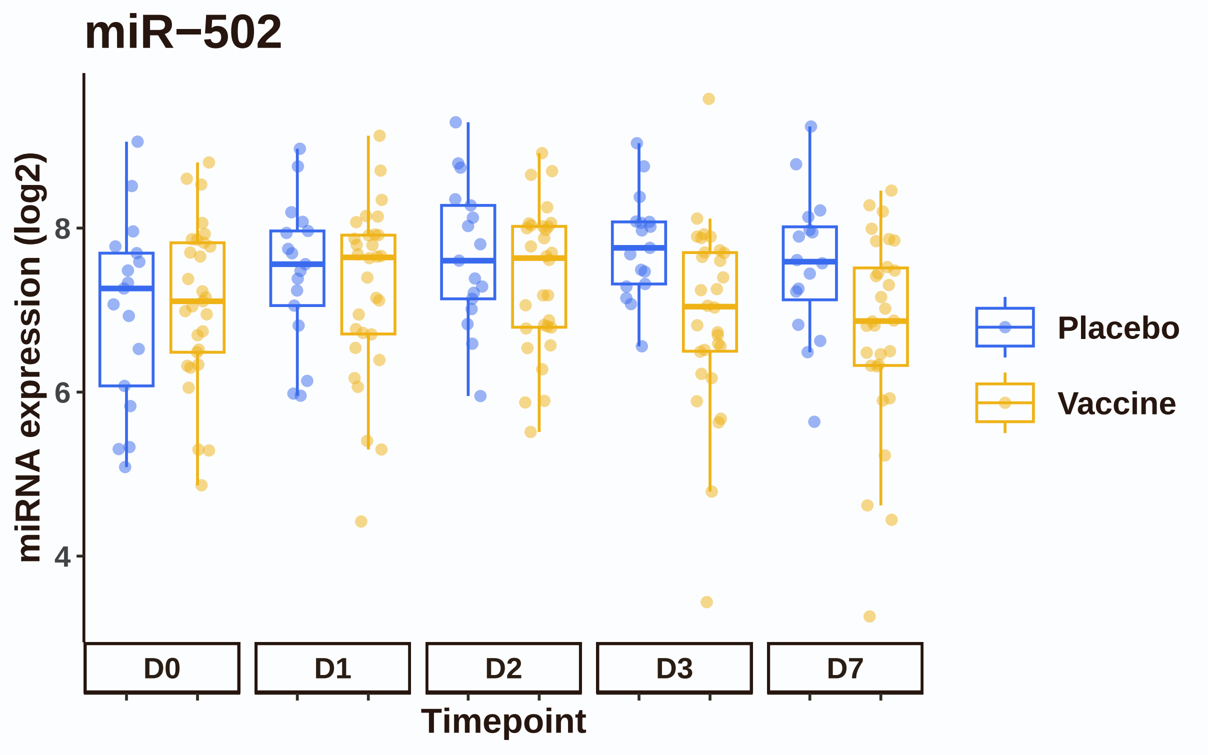
miR−502
miRNA expression (log2)
8
6
4
D0
D1
D2
D3
D7
Timepoint
Placebo
Vaccine

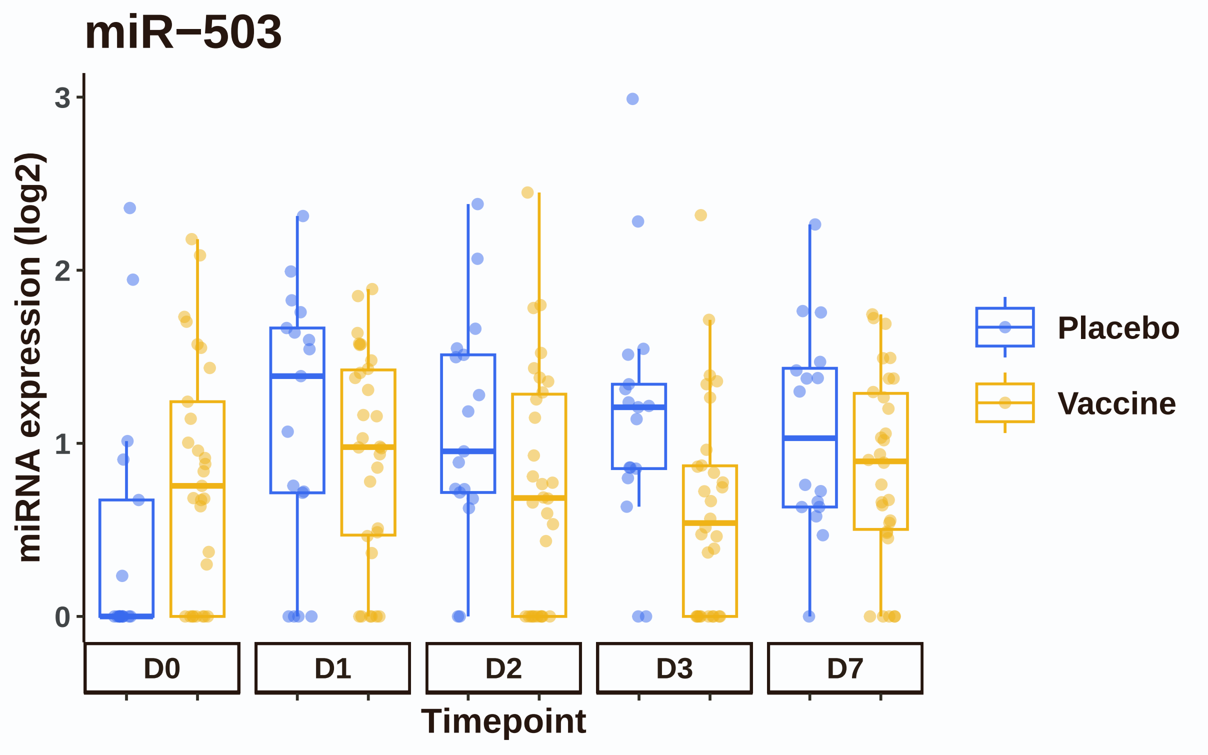

miR−503
miRNA expression (log2)
3
2
1
0
D0
D1
D2
D3
D7
Timepoint
Placebo
Vaccine

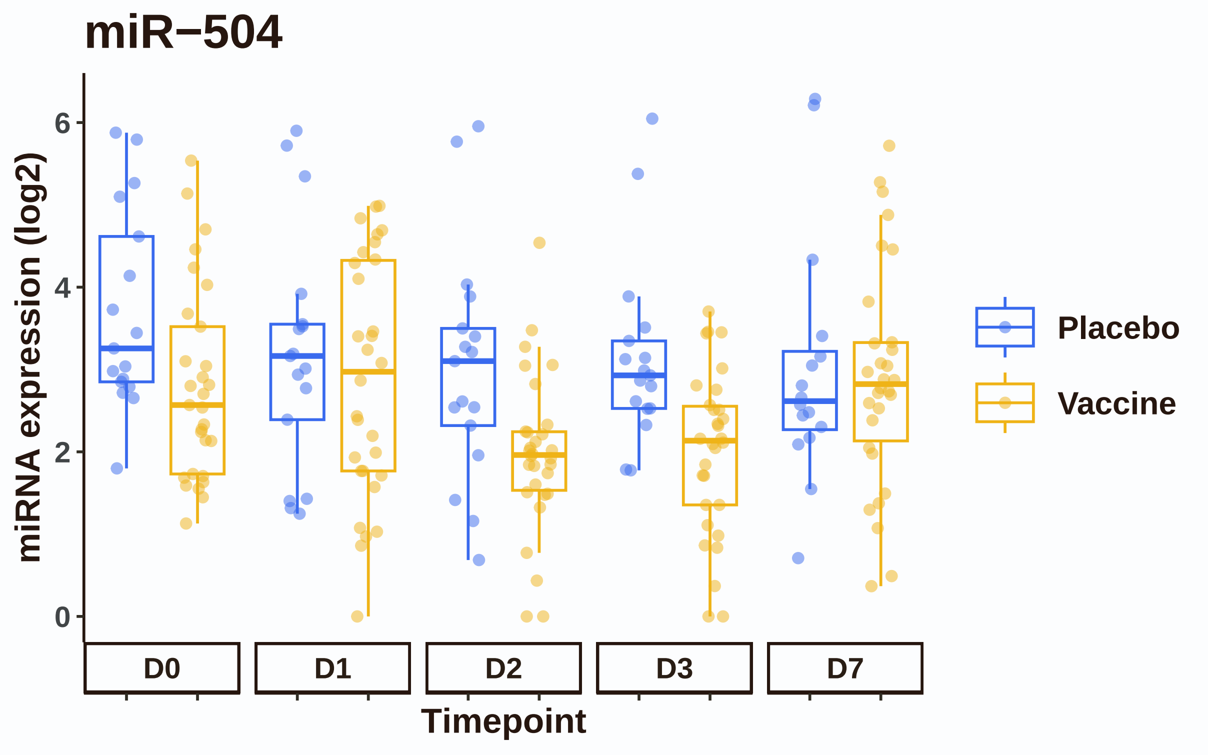
miR−504
miRNA expression (log2)
6
4
2
0
D0
D1
D2
D3
D7
Timepoint
Placebo
Vaccine

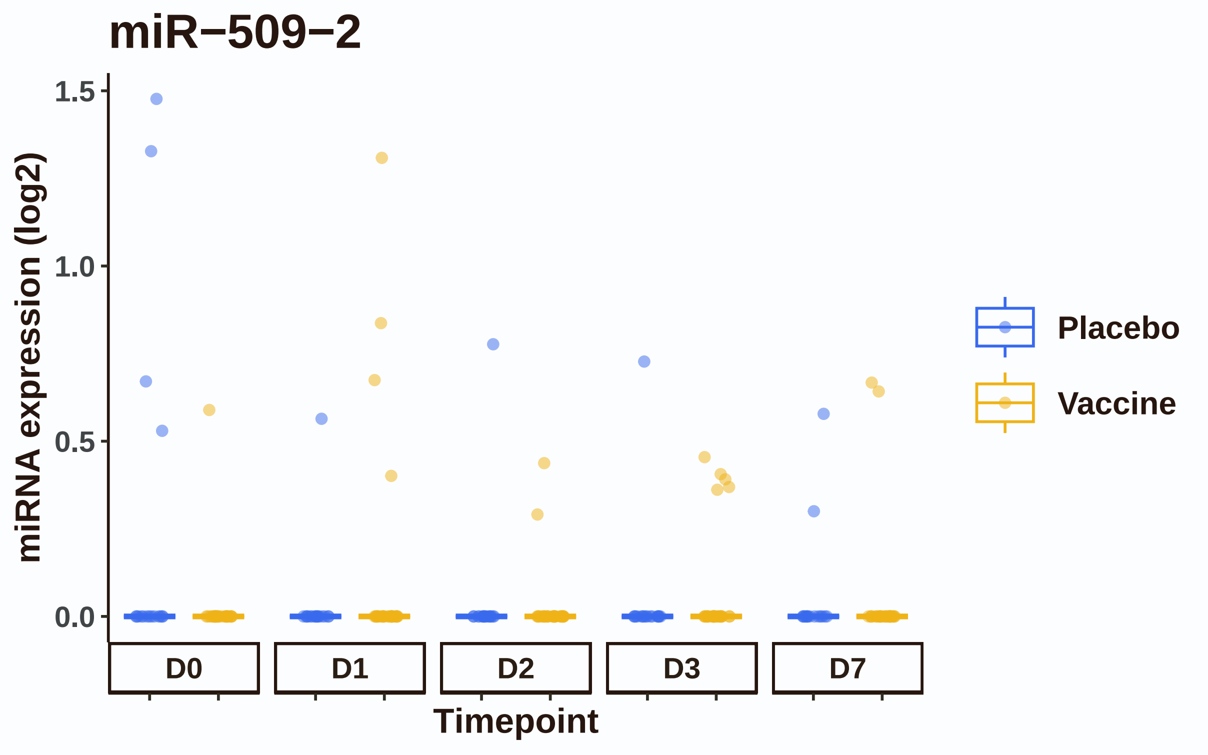
miR−509−2
miRNA expression (log2)
1.5
1.0
0.5
0.0
D0
D1
D2
D3
D7
Timepoint
Placebo
Vaccine

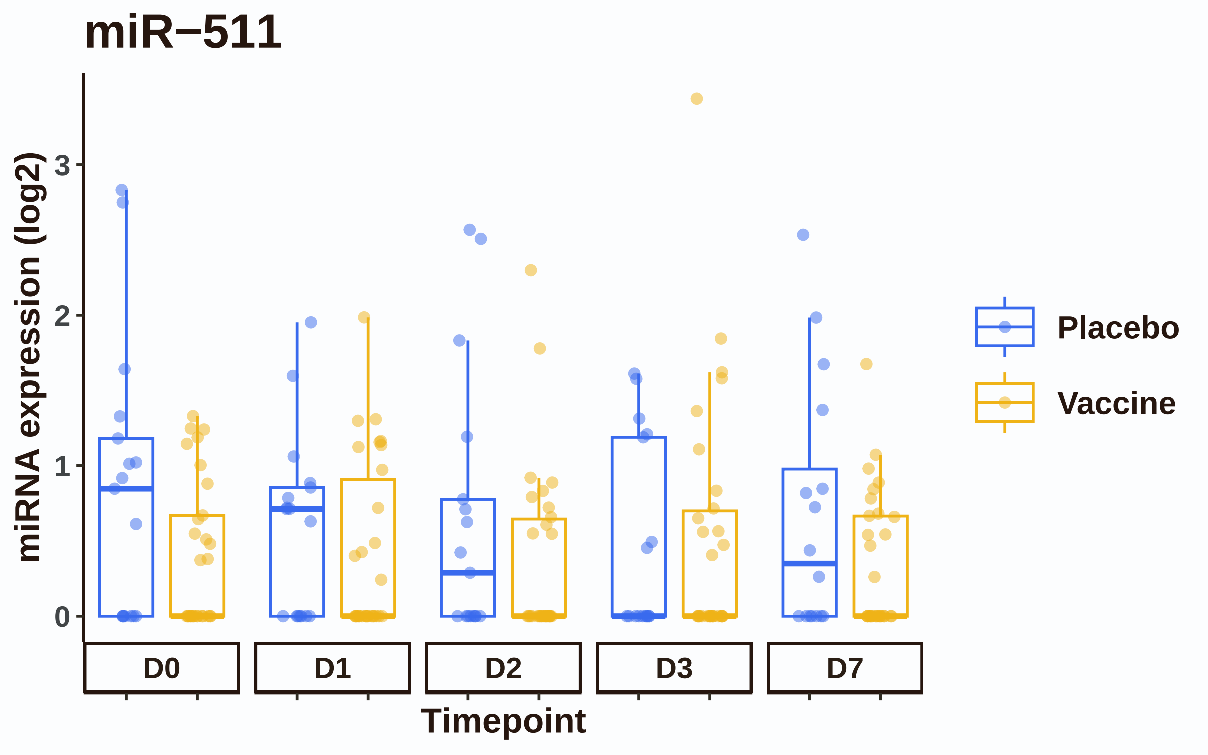
miR−511
miRNA expression (log2)
0
1
2
3
D0
D1
D2
D3
D7
Timepoint
Placebo
Vaccine

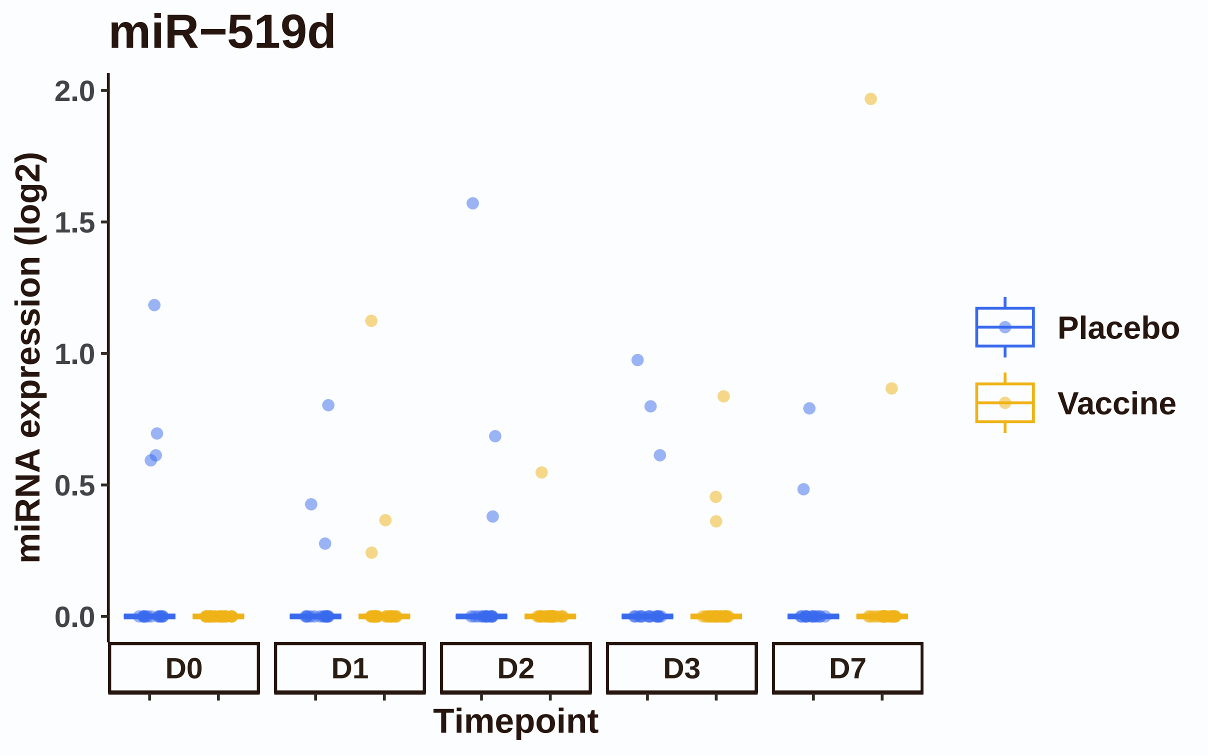
miR−519d
miRNA expression (log2)
2.0
1.5
1.0
0.5
0.0
D0
D1
D2
D3
D7
Timepoint
Placebo
Vaccine

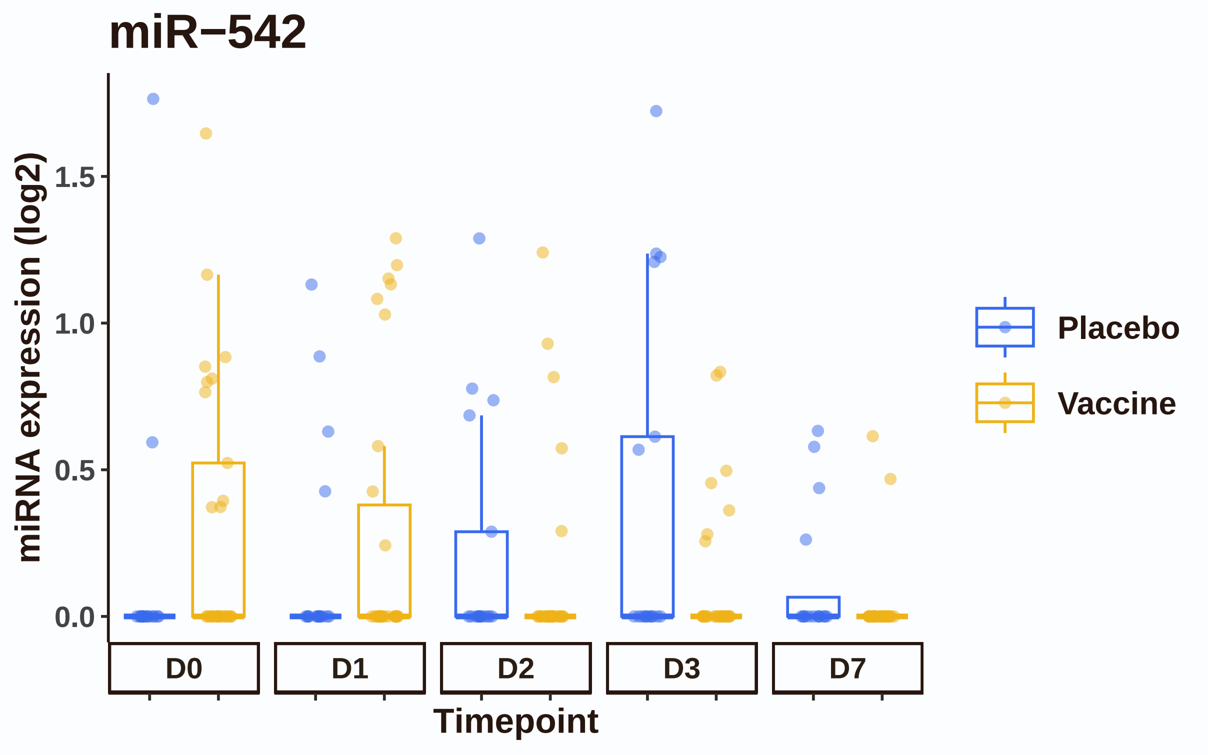

miR−542
miRNA expression (log2)
0.0
0.5
1.0
1.5
D0
D1
D2
D3
D7
Timepoint
Placebo
Vaccine

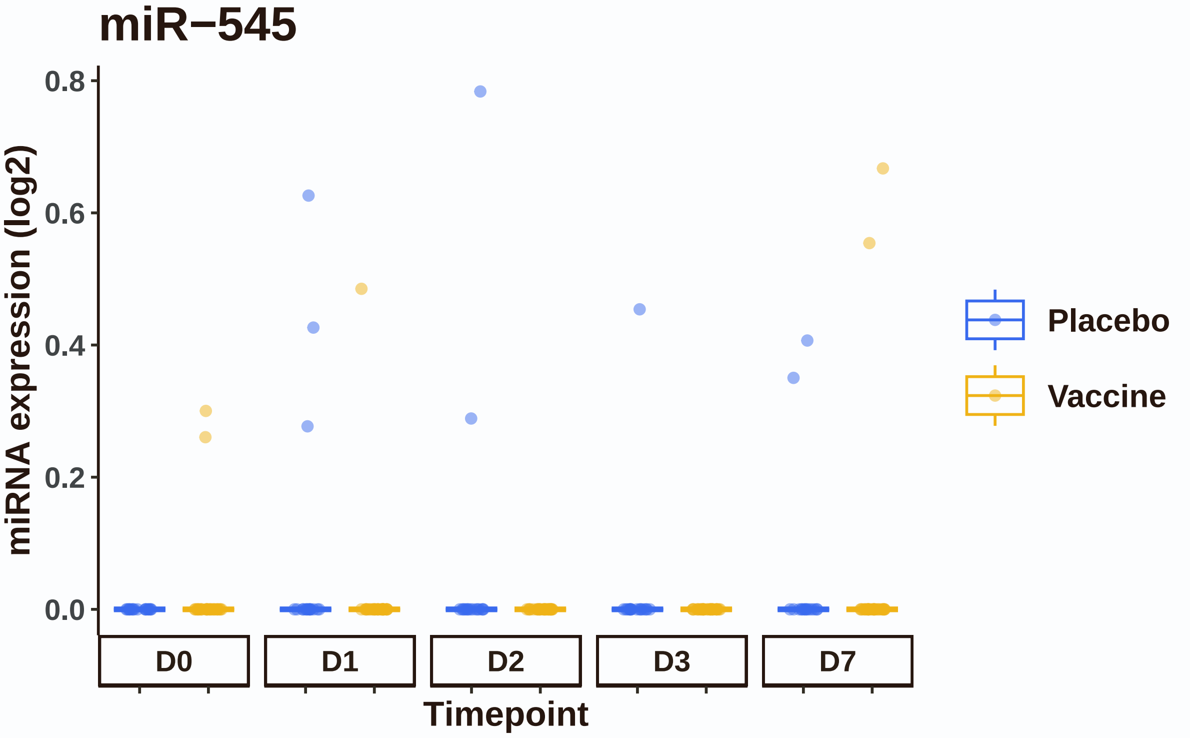
miR−545
miRNA expression (log2)
0.8
0.6
0.4
0.2
0.0
D0
D1
D2
D3
D7
Timepoint
Placebo
Vaccine

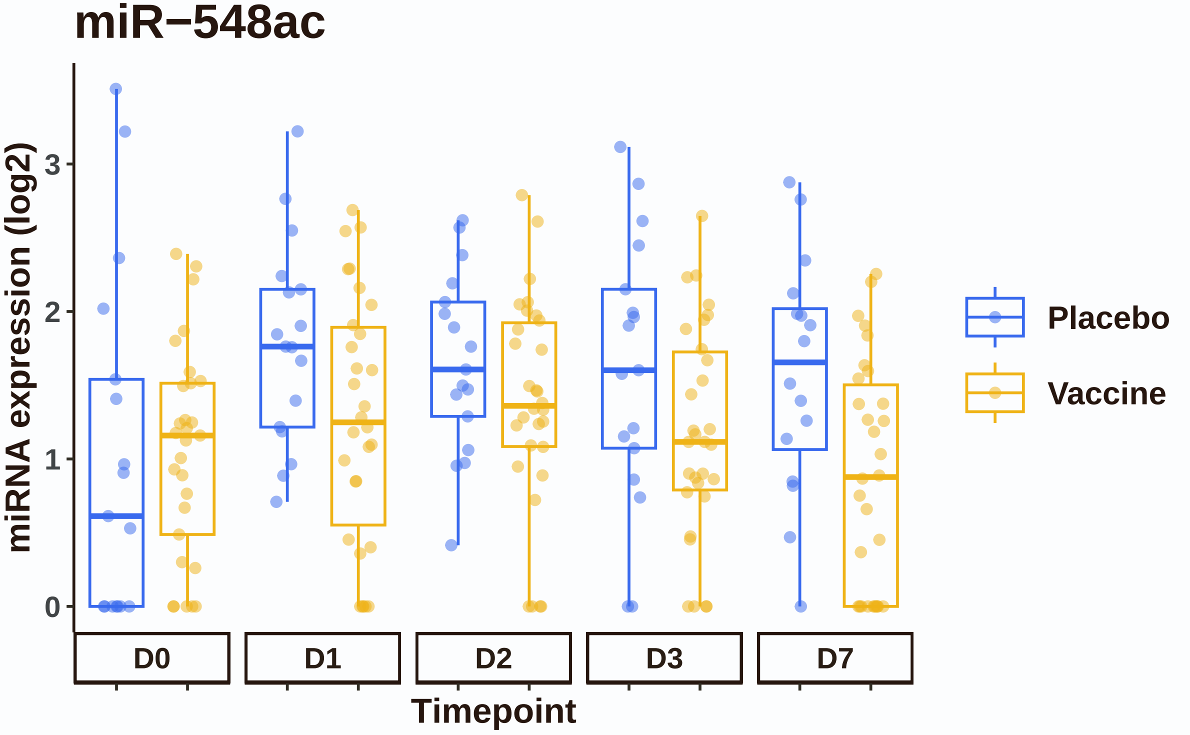
miR−548ac
miRNA expression (log2)
0
1
2
3
D0
D1
D2
D3
D7
Timepoint
Placebo
Vaccine

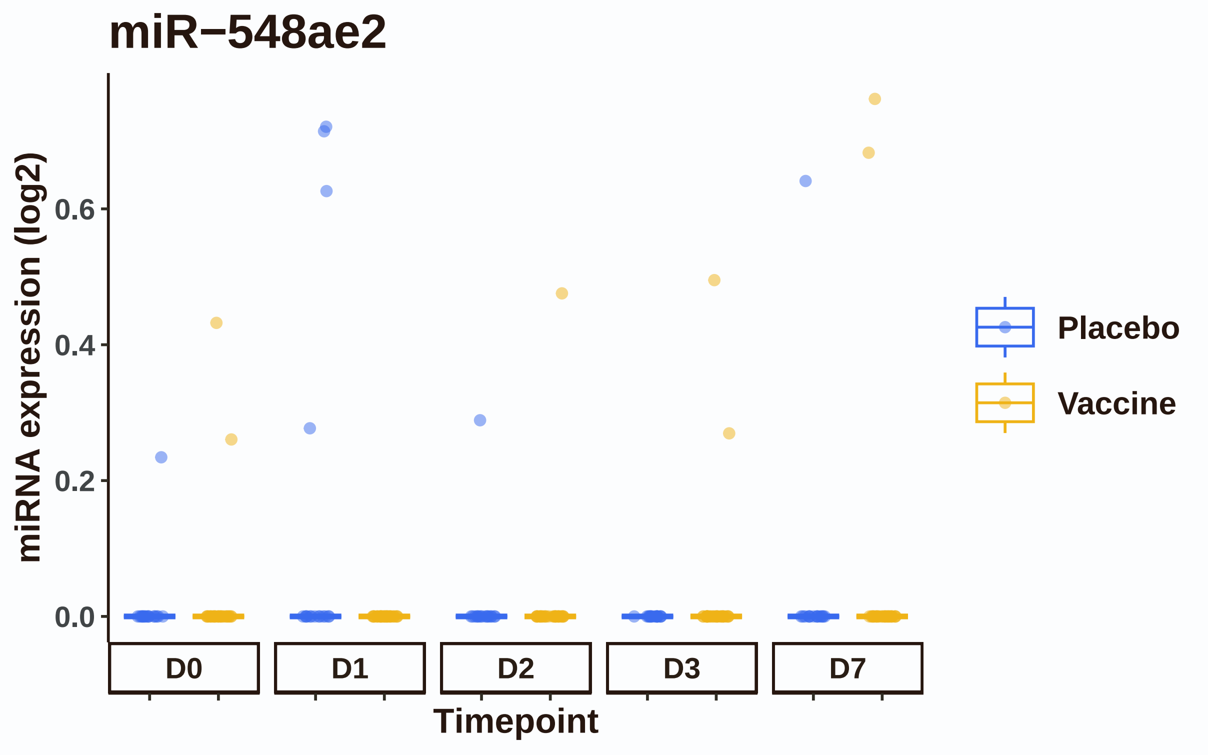

miR−548ae2
miRNA expression (log2)
0.6
0.4
0.2
0.0
D0
D1
D2
D3
D7
Timepoint
Placebo
Vaccine

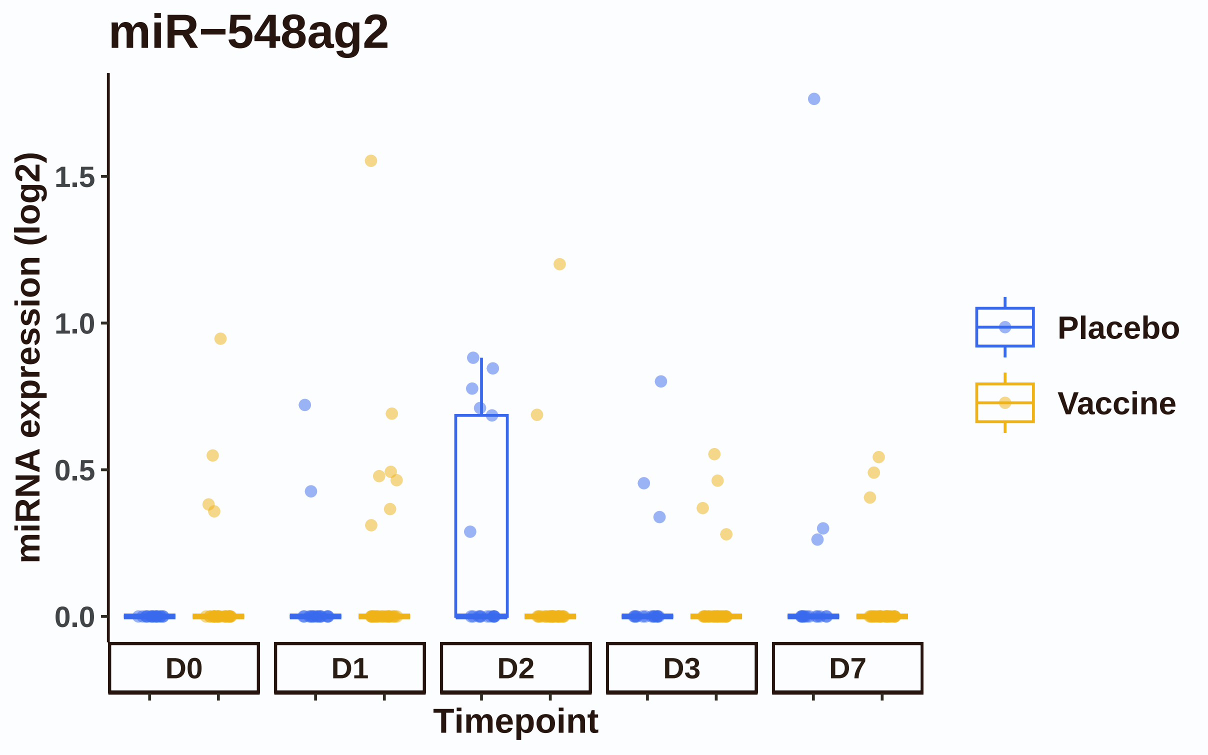
miR−548ag2
miRNA expression (log2)
1.5
1.0
0.5
0.0
D0
D1
D2
D3
D7
Timepoint
Placebo
Vaccine

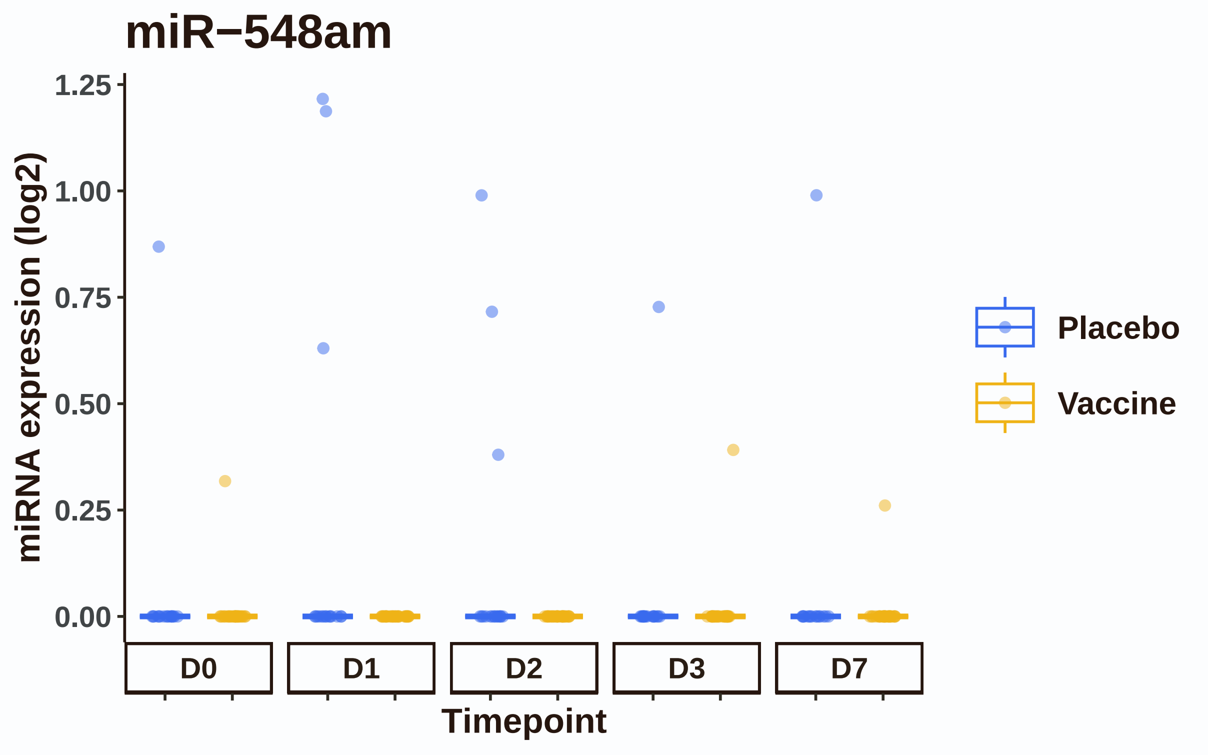
miR−548am
miRNA expression (log2)
1.25
1.00
0.75
0.50
0.25
0.00
D0
D1
D2
D3
D7
Timepoint
Placebo
Vaccine

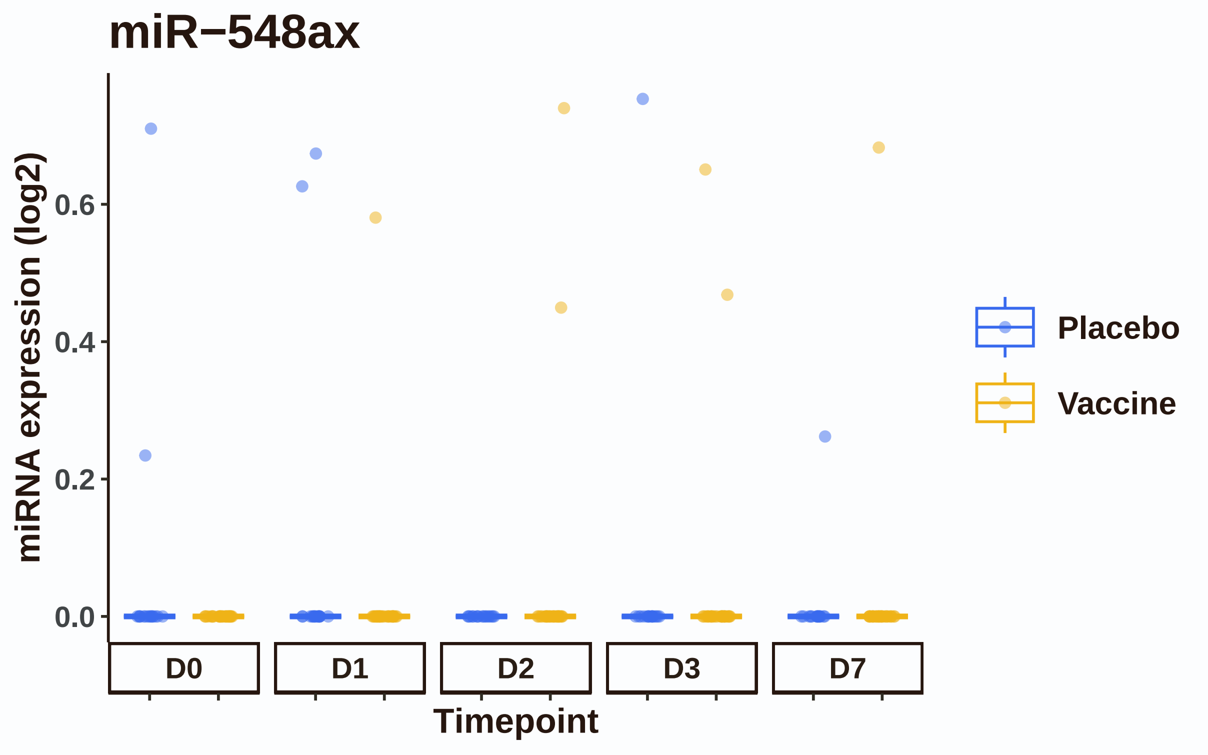
miR−548ax
miRNA expression (log2)
0.6
0.4
0.2
0.0
D0
D1
D2
D3
D7
Timepoint
Placebo
Vaccine

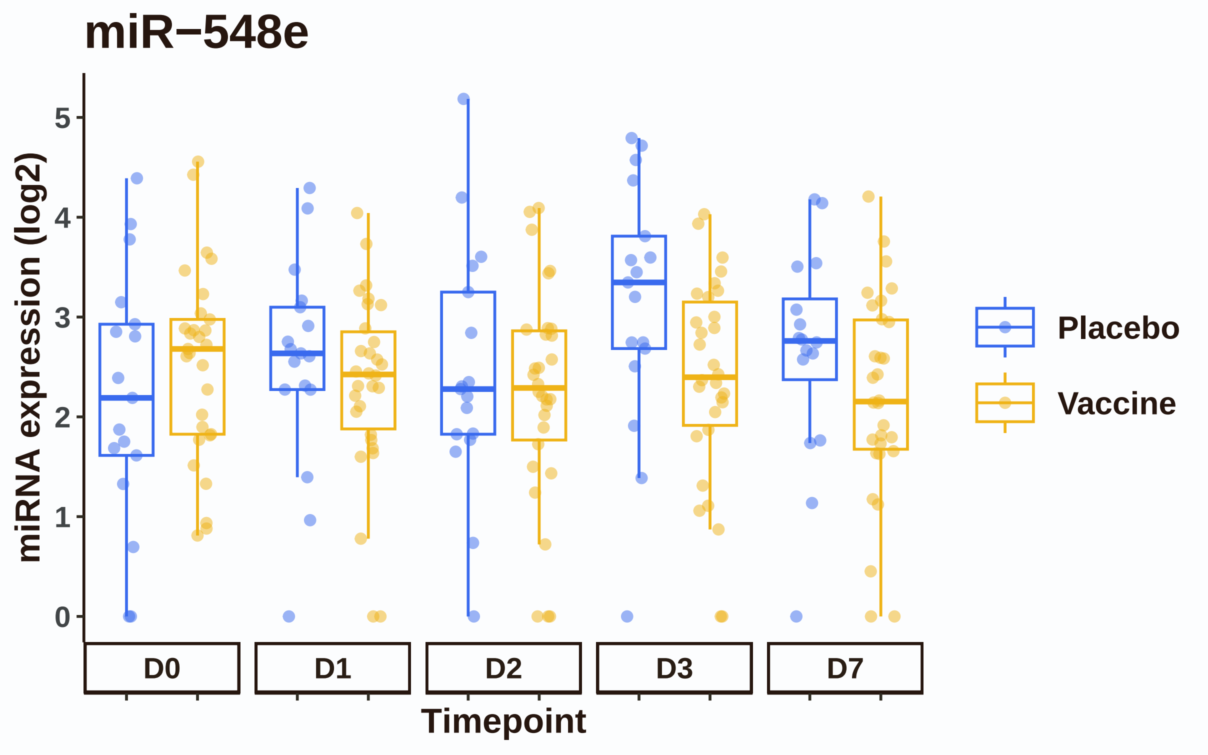

miR−548e
miRNA expression (log2)
0
1
2
3
4
5
D0
D1
D2
D3
D7
Timepoint
Placebo
Vaccine

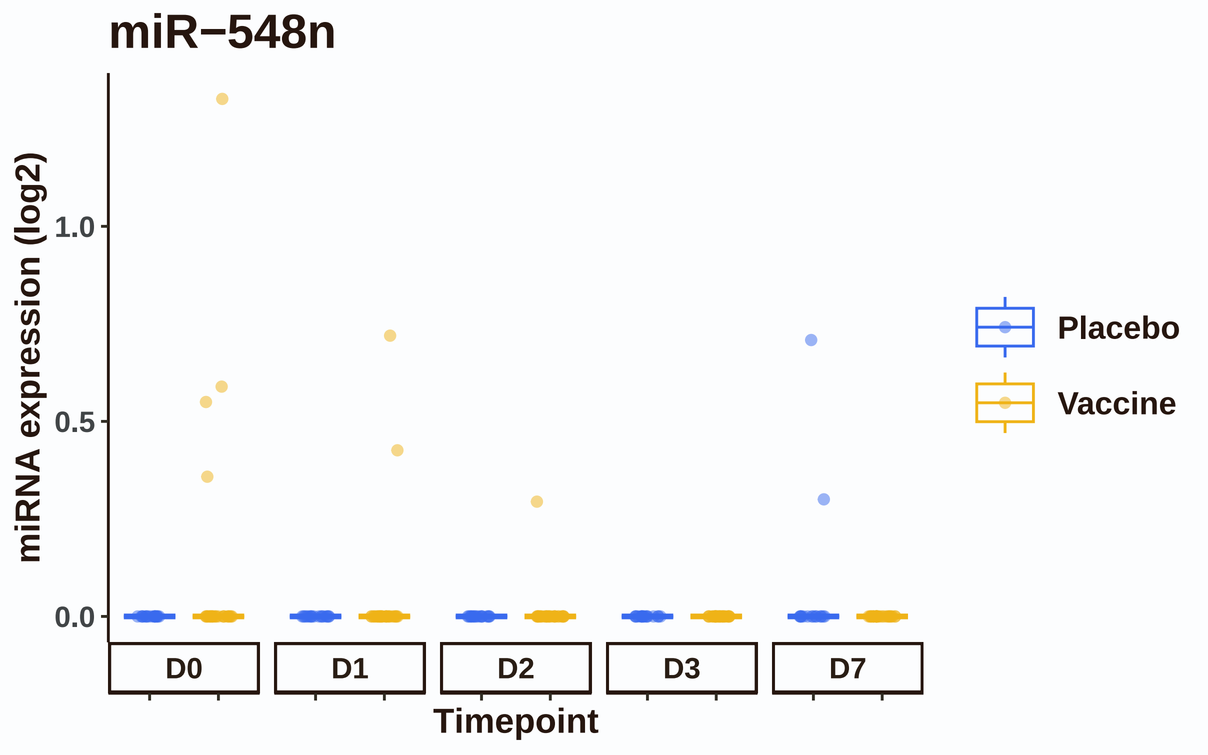

miR−548n
miRNA expression (log2)
1.0
0.5
0.0
D0
D1
D2
D3
D7
Timepoint
Placebo
Vaccine

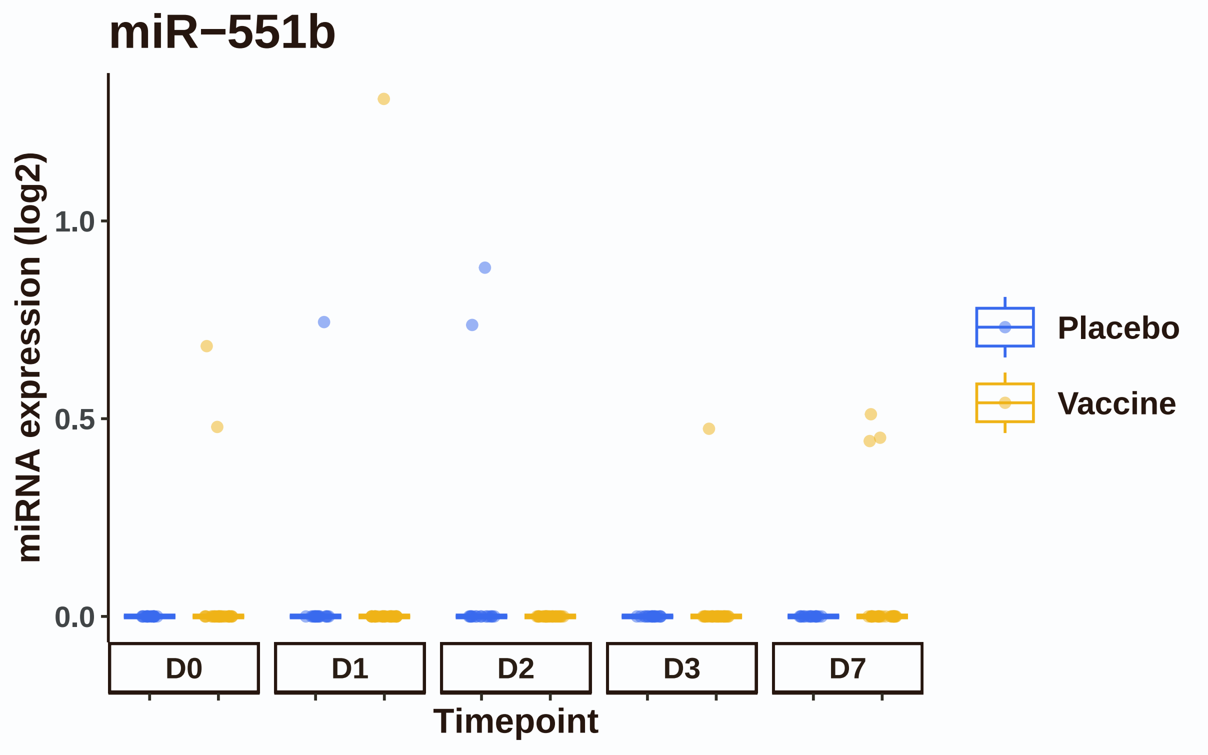
miR−551b
miRNA expression (log2)
1.0
0.5
0.0
D0
D1
D2
D3
D7
Timepoint
Placebo
Vaccine

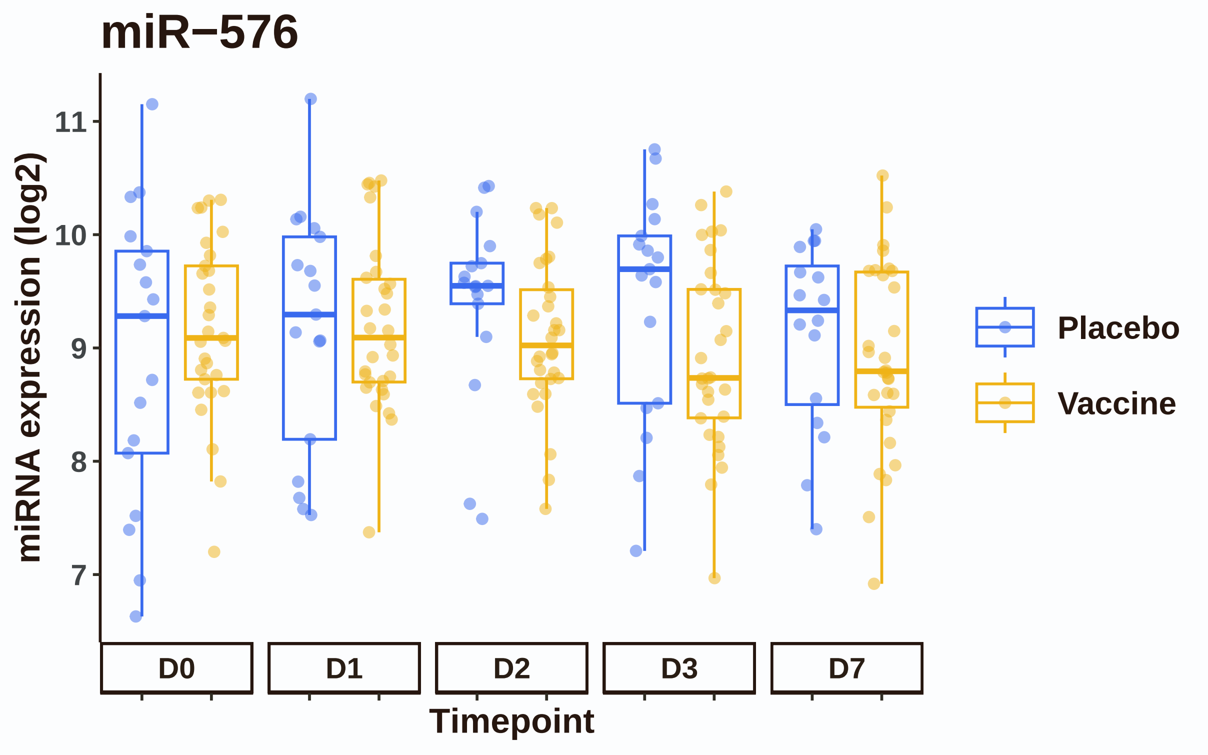

miR−576
miRNA expression (log2)
11
10
9
8
7
D0
D1
D2
D3
D7
Timepoint
Placebo
Vaccine

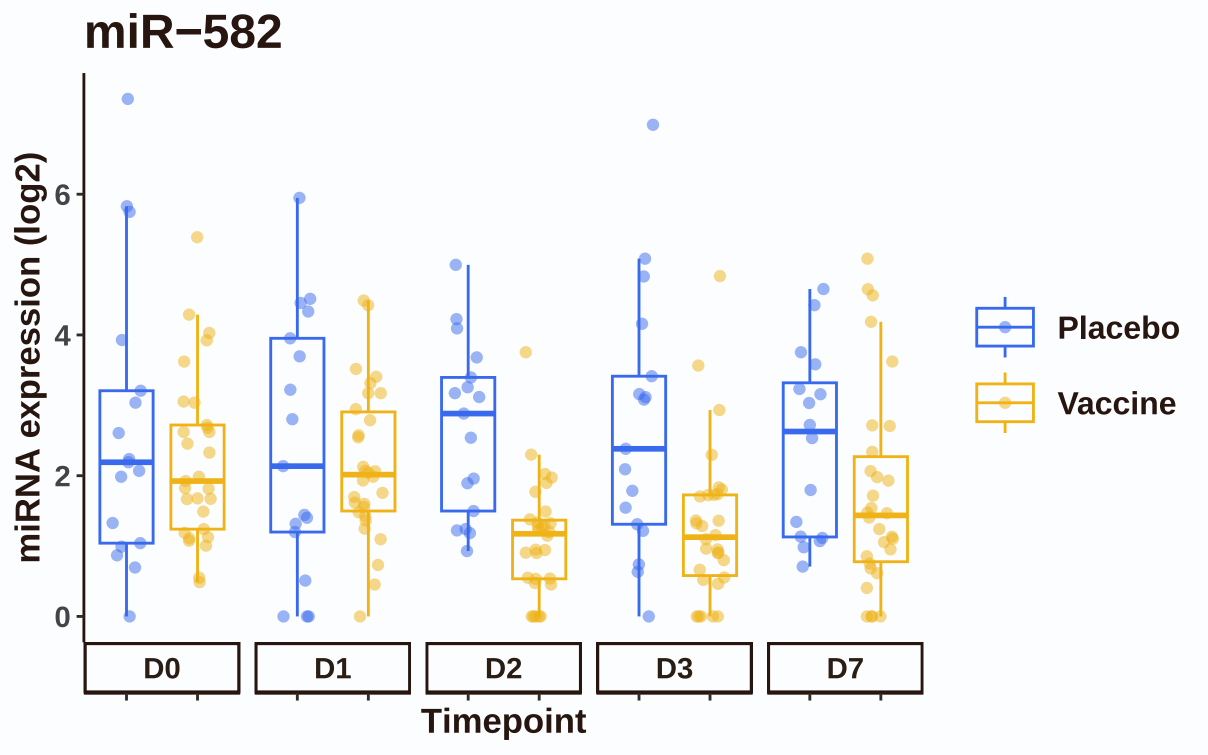

miR−582
miRNA expression (log2)
0
2
4
6
D0
D1
D2
D3
D7
Timepoint
Placebo
Vaccine

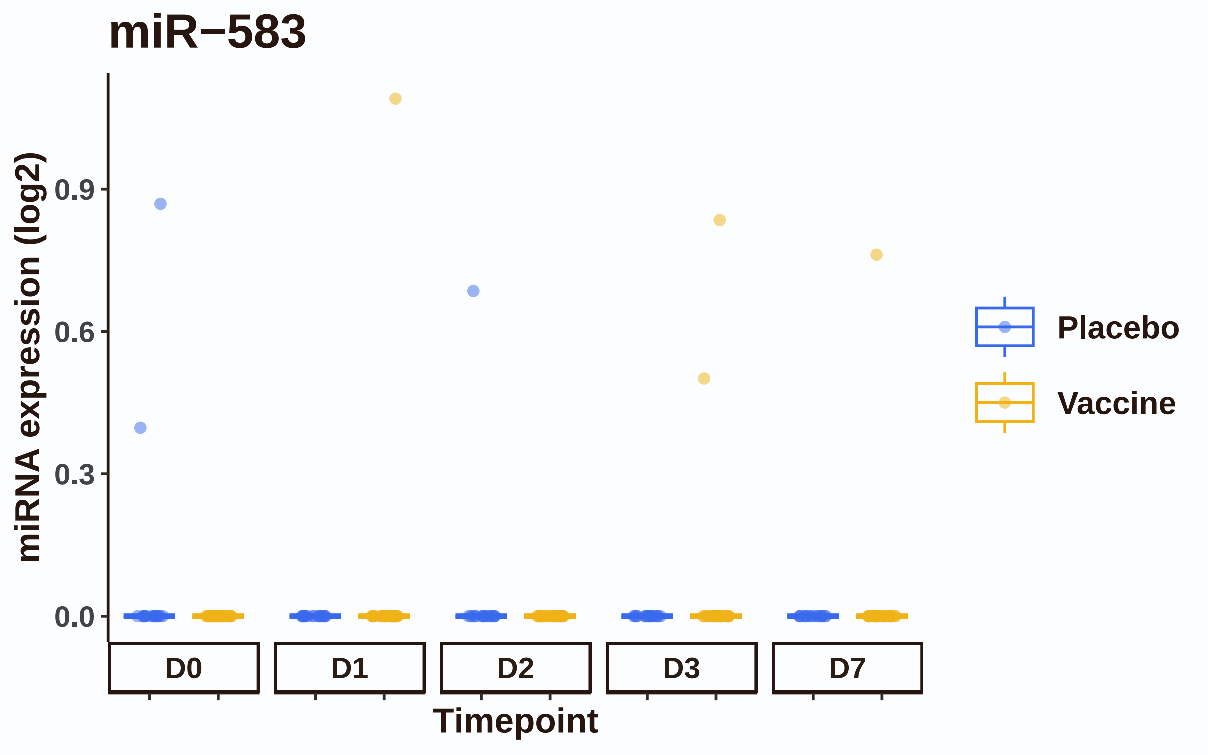

miR−583
miRNA expression (log2)
0.0
0.3
0.6
0.9
D0
D1
D2
D3
D7
Timepoint
Placebo
Vaccine

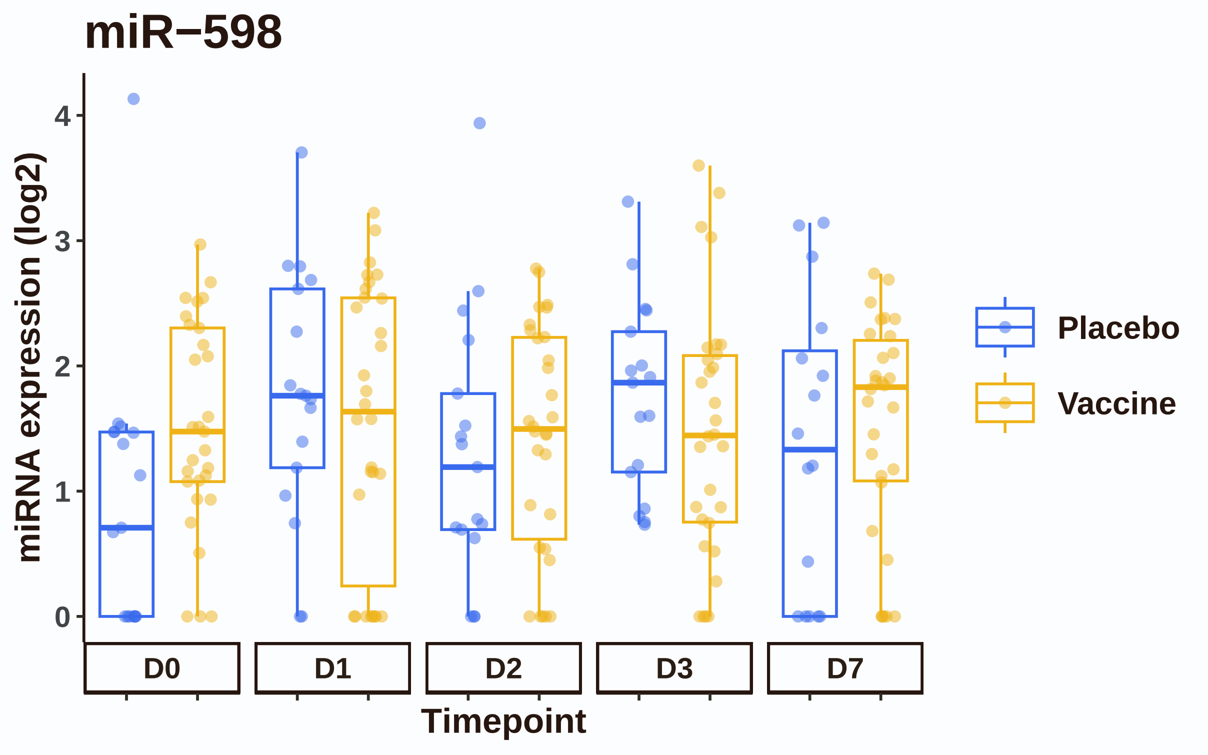
miR−598
miRNA expression (log2)
0
1
2
3
4
D0
D1
D2
D3
D7
Timepoint
Placebo
Vaccine

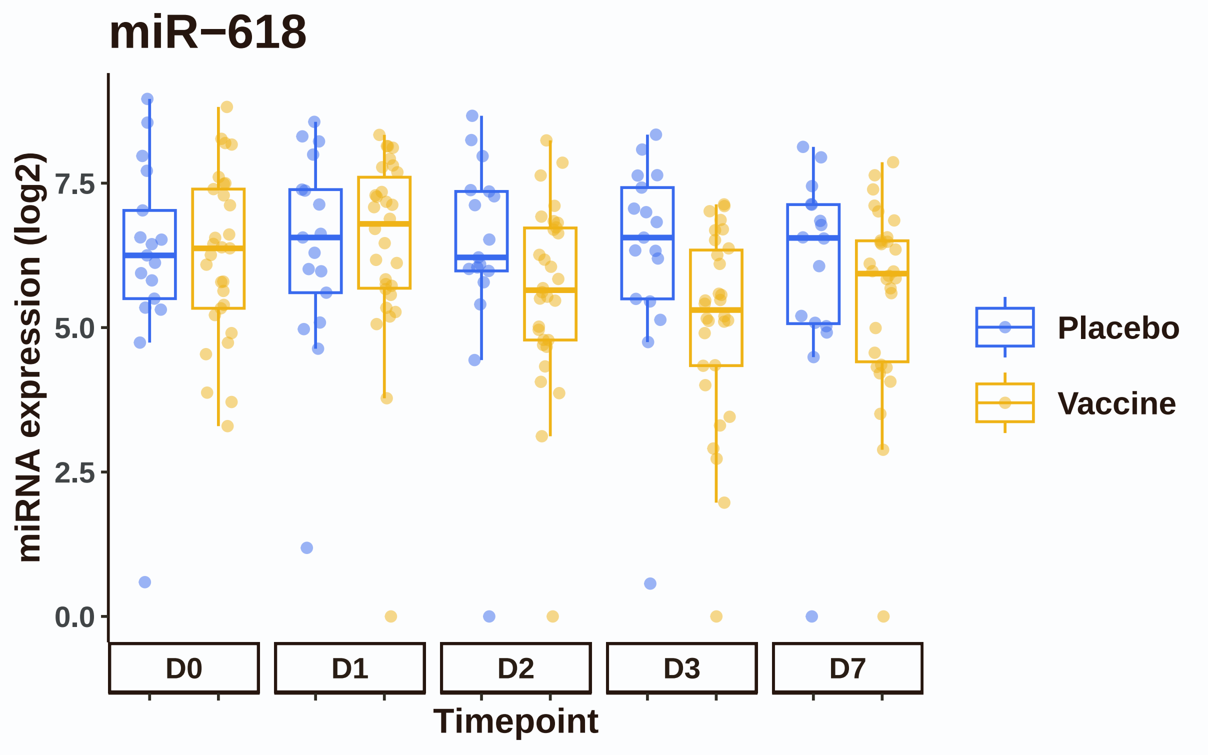

miR−618
miRNA expression (log2)
7.5
5.0
2.5
0.0
D0
D1
D2
D3
D7
Timepoint
Placebo
Vaccine

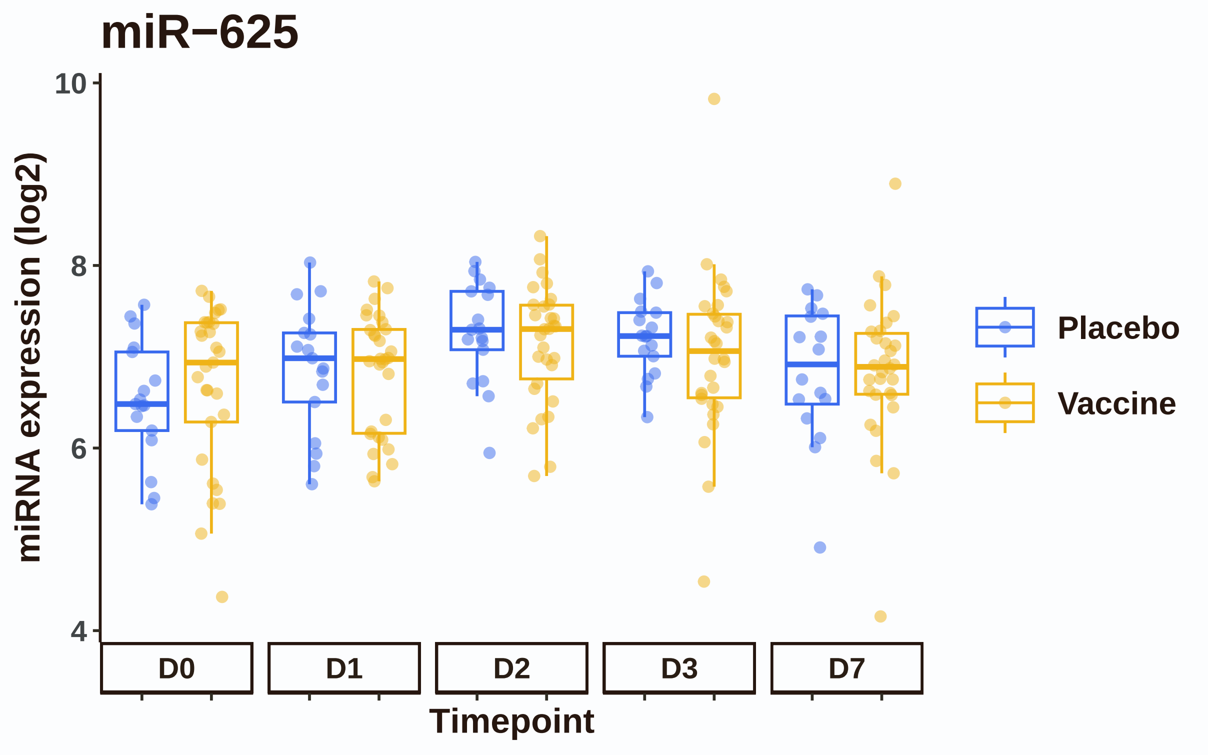
miR−625
miRNA expression (log2)
10
8
6
4
D0
D1
D2
D3
D7
Timepoint
Placebo
Vaccine

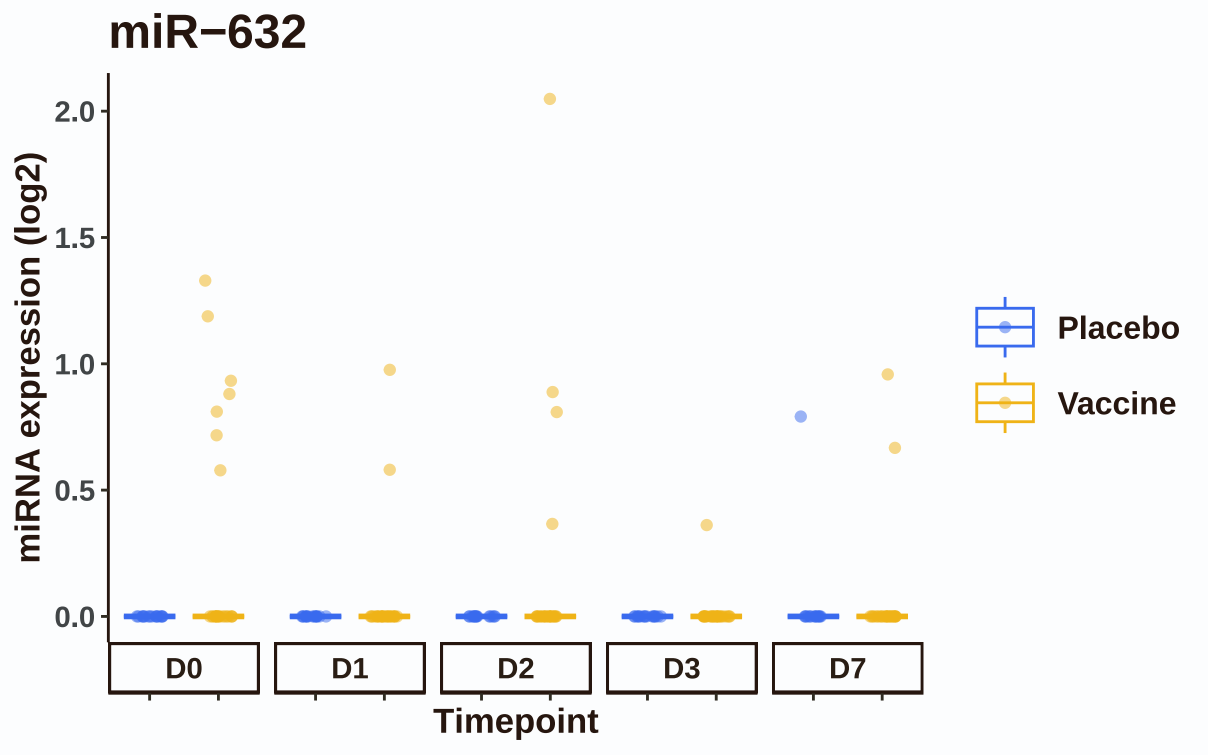

miR−632
miRNA expression (log2)
2.0
1.5
1.0
0.5
0.0
D0
D1
D2
D3
D7
Timepoint
Placebo
Vaccine

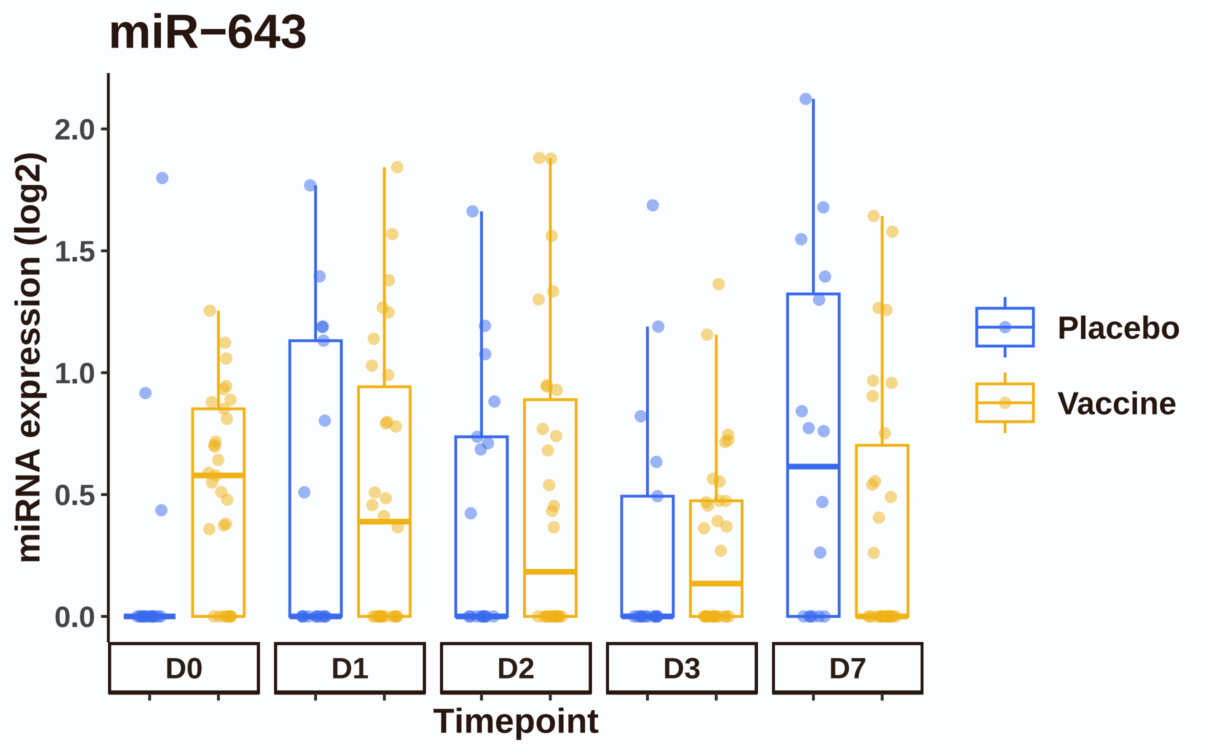

miR−643
miRNA expression (log2)
2.0
1.5
1.0
0.5
0.0
D0
D1
D2
D3
D7
Timepoint
Placebo
Vaccine

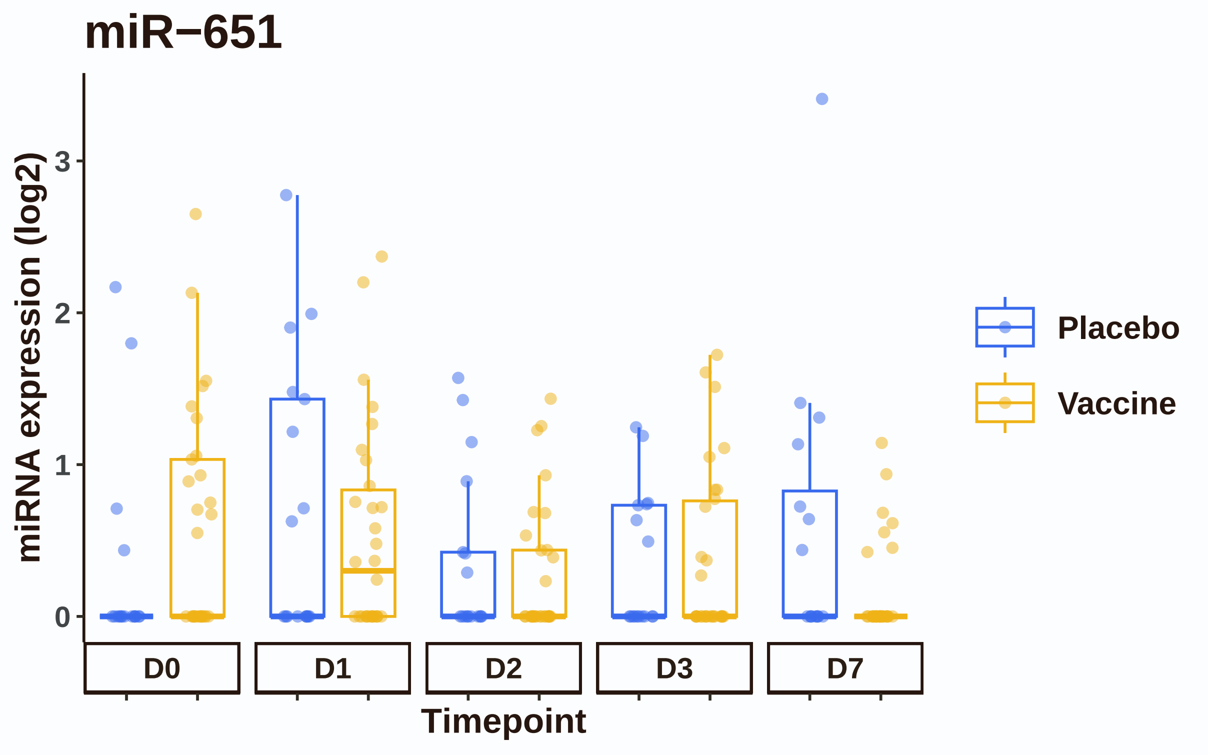

miR−651
miRNA expression (log2)
0
1
2
3
D0
D1
D2
D3
D7
Timepoint
Placebo
Vaccine

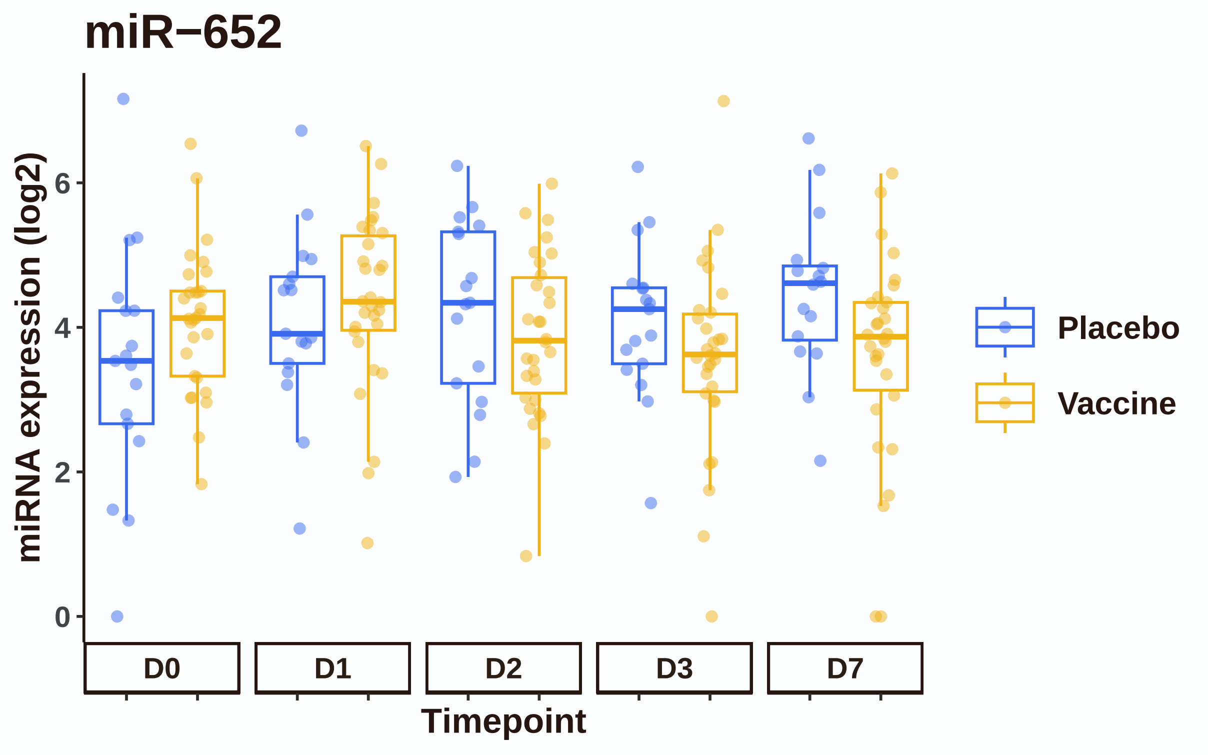

miR−652
miRNA expression (log2)
0
2
4
6
D0
D1
D2
D3
D7
Timepoint
Placebo
Vaccine

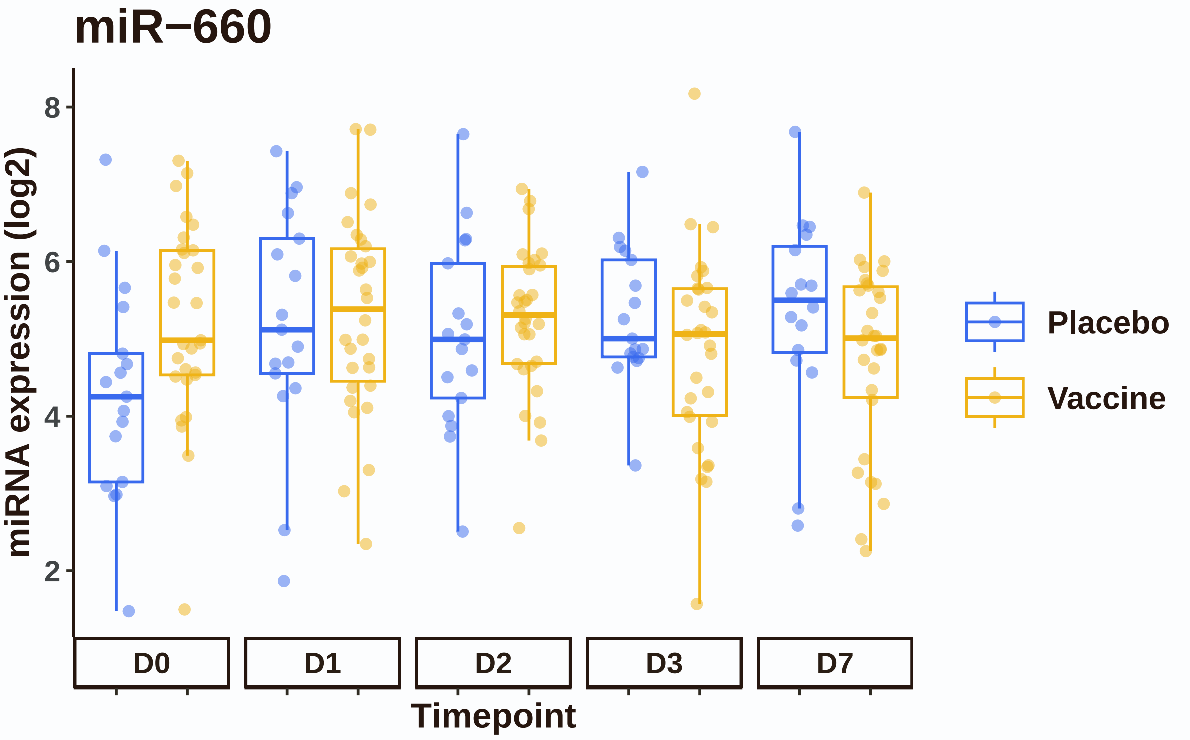
miR−660
miRNA expression (log2)
8
6
4
2
D0
D1
D2
D3
D7
Timepoint
Placebo
Vaccine

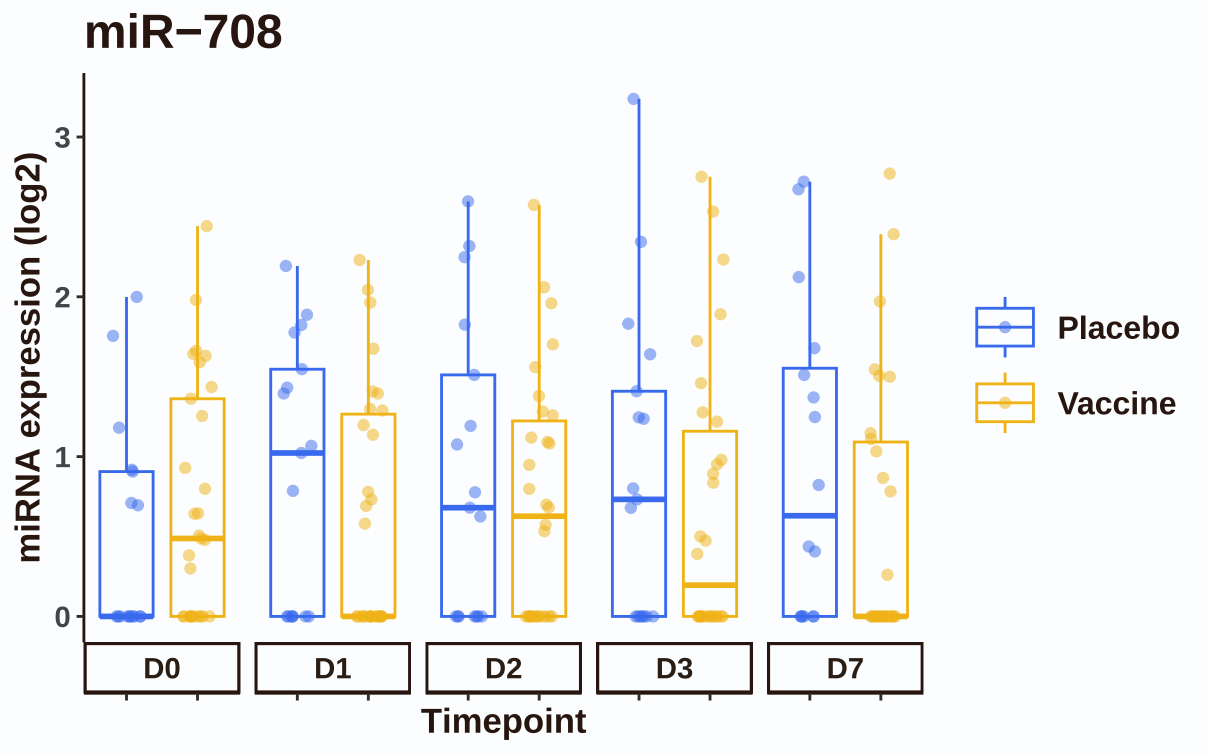
miR−708
miRNA expression (log2)
0
1
2
3
D0
D1
D2
D3
D7
Timepoint
Placebo
Vaccine

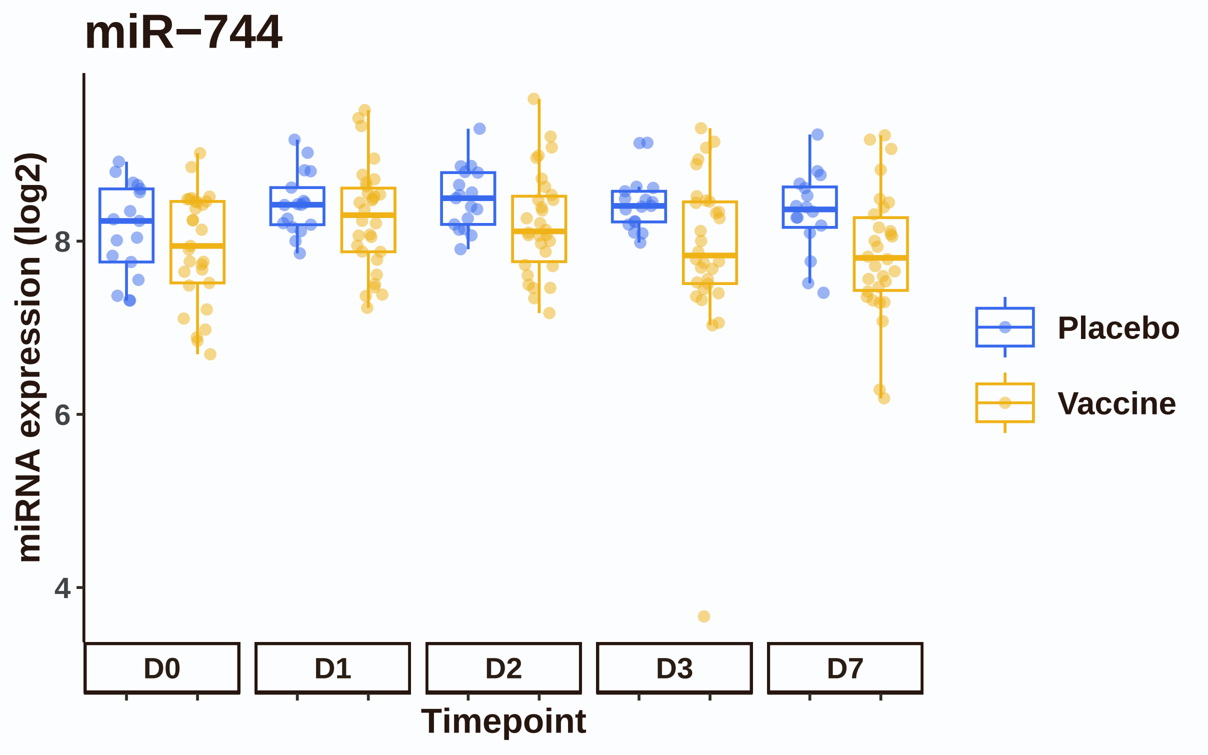
miR−744
miRNA expression (log2)
8
6
4
D0
D1
D2
D3
D7
Timepoint
Placebo
Vaccine

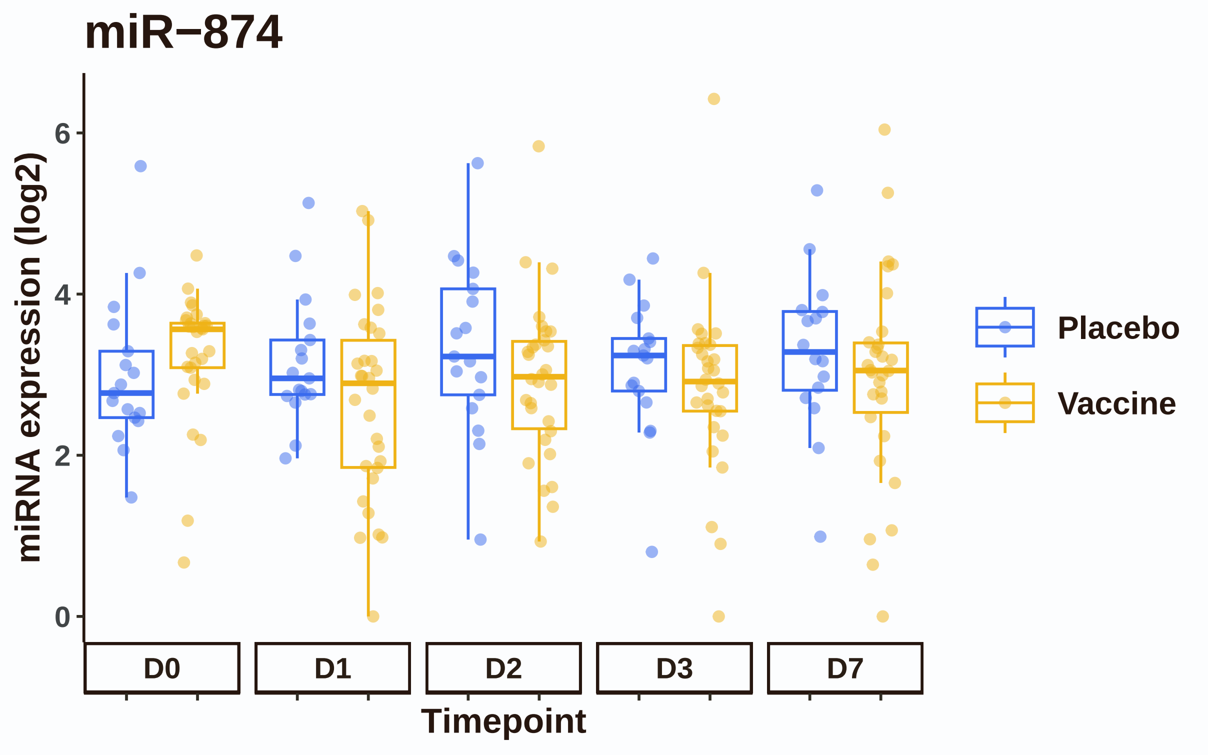
miR−874
miRNA expression (log2)
6
4
2
0
D0
D1
D2
D3
D7
Timepoint
Placebo
Vaccine

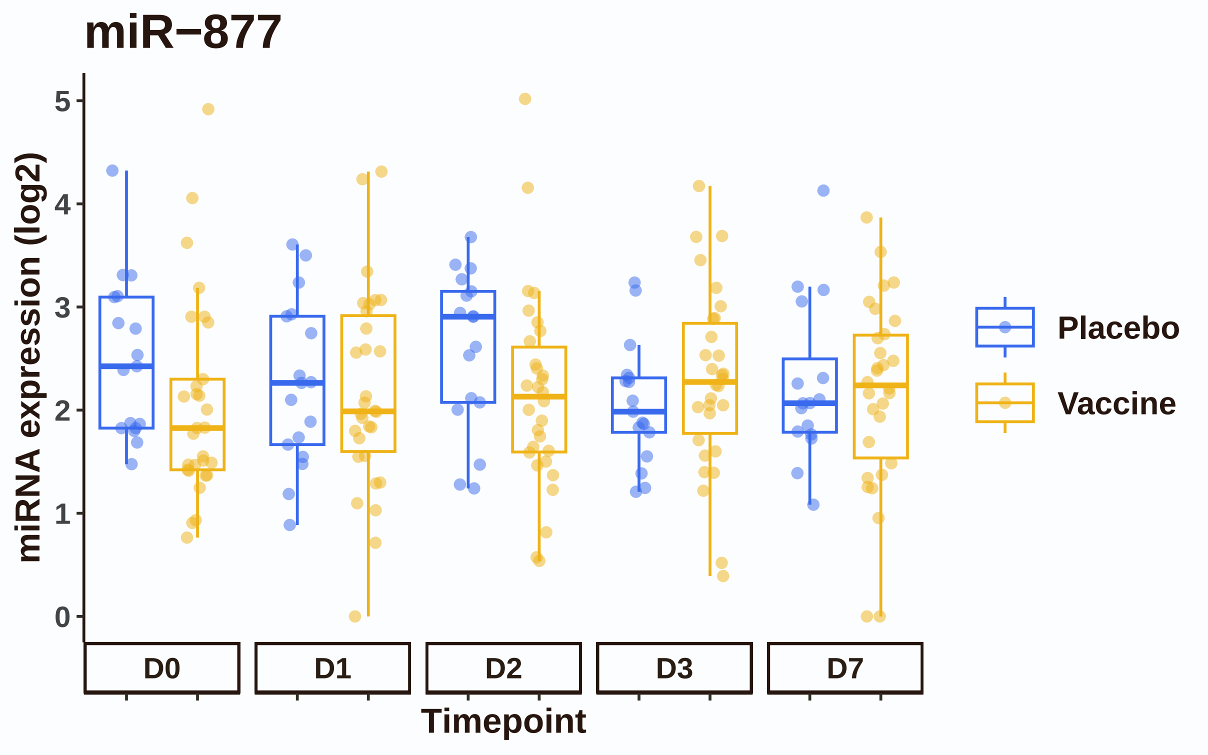
miR−877
miRNA expression (log2)
0
1
2
3
4
5
D0
D1
D2
D3
D7
Timepoint
Placebo
Vaccine

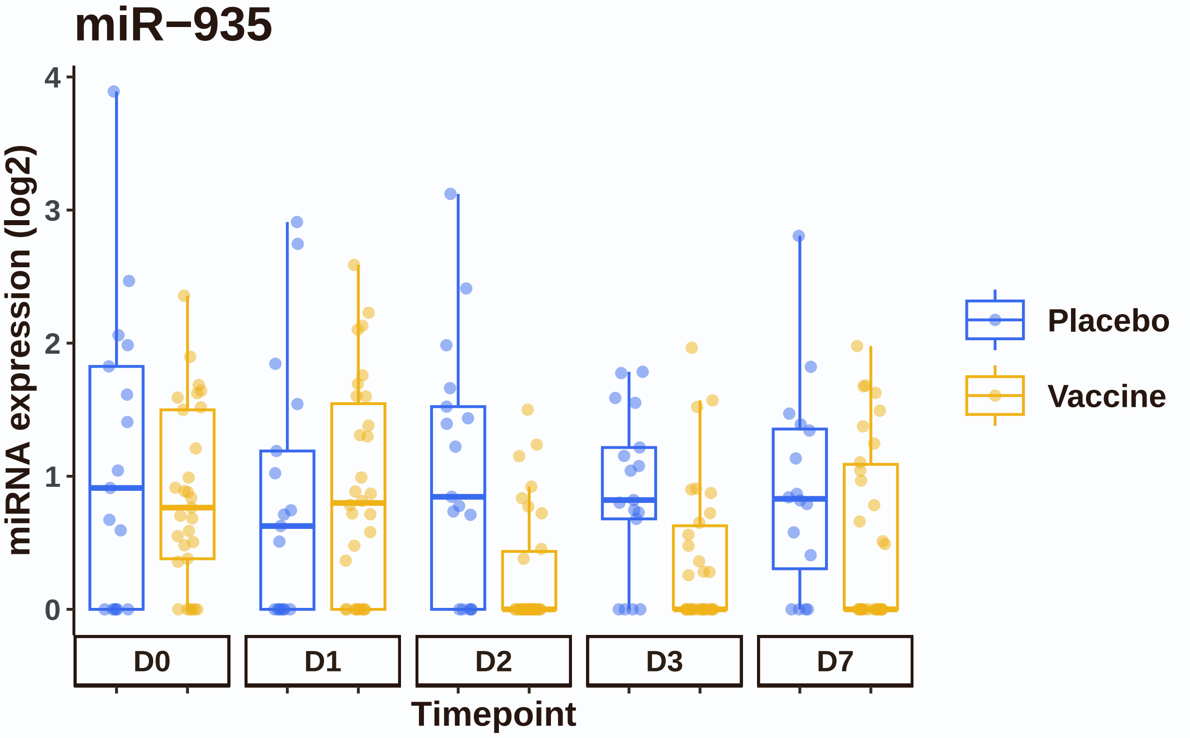
miR−935
miRNA expression (log2)
4
3
2
1
0
D0
D1
D2
D3
D7
Timepoint
Placebo
Vaccine

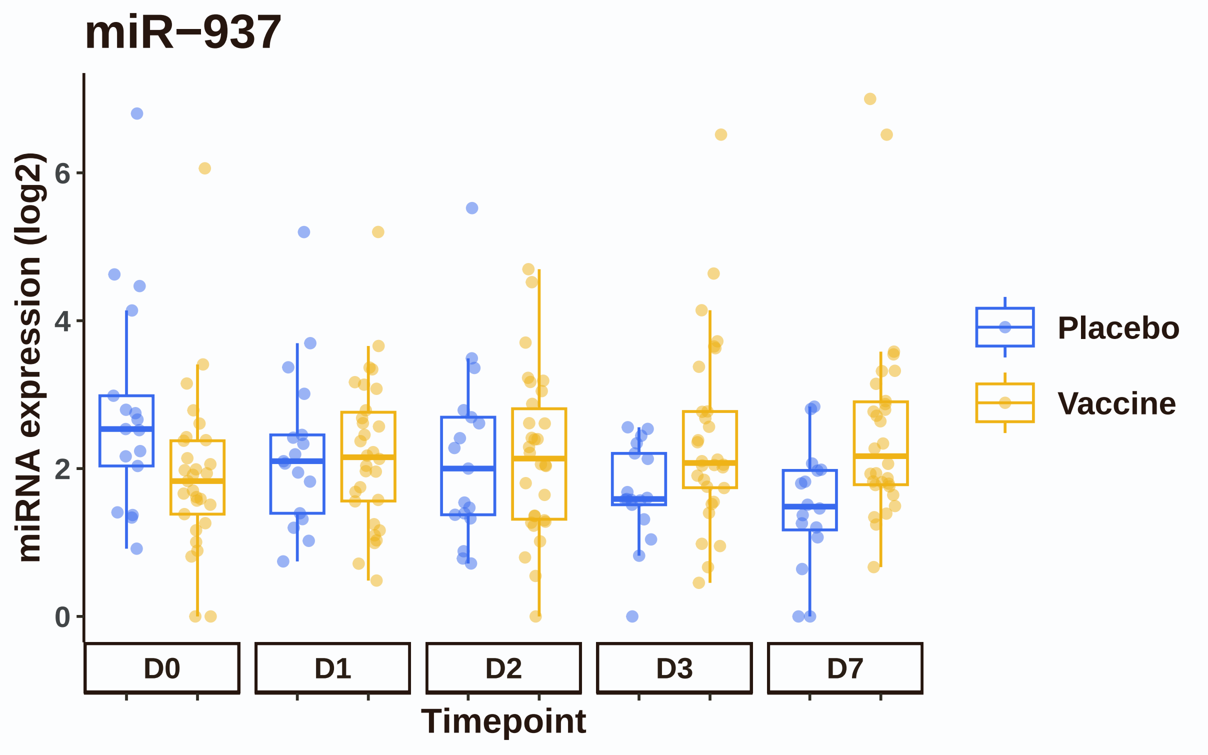
miR−937
miRNA expression (log2)
0
2
4
6
D0
D1
D2
D3
D7
Timepoint
Placebo
Vaccine

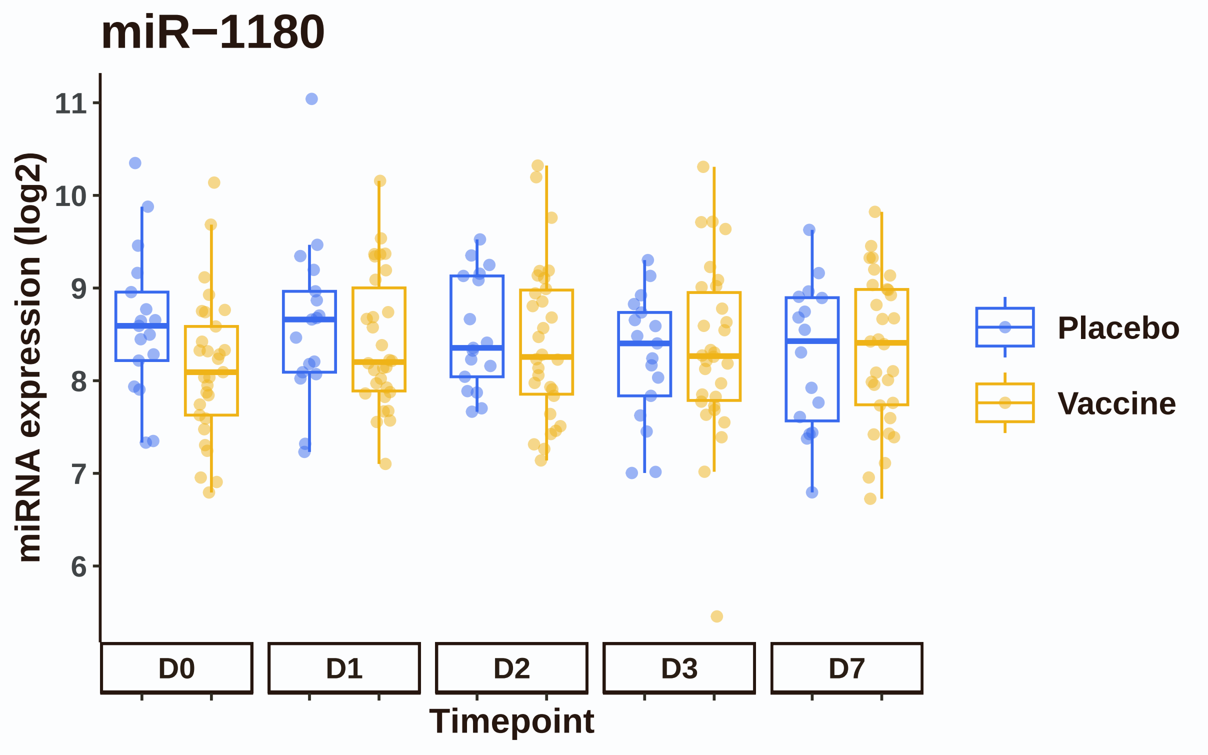
miR−1180
miRNA expression (log2)
11
10
9
8
7
6
D0
D1
D2
D3
D7
Timepoint
Placebo
Vaccine

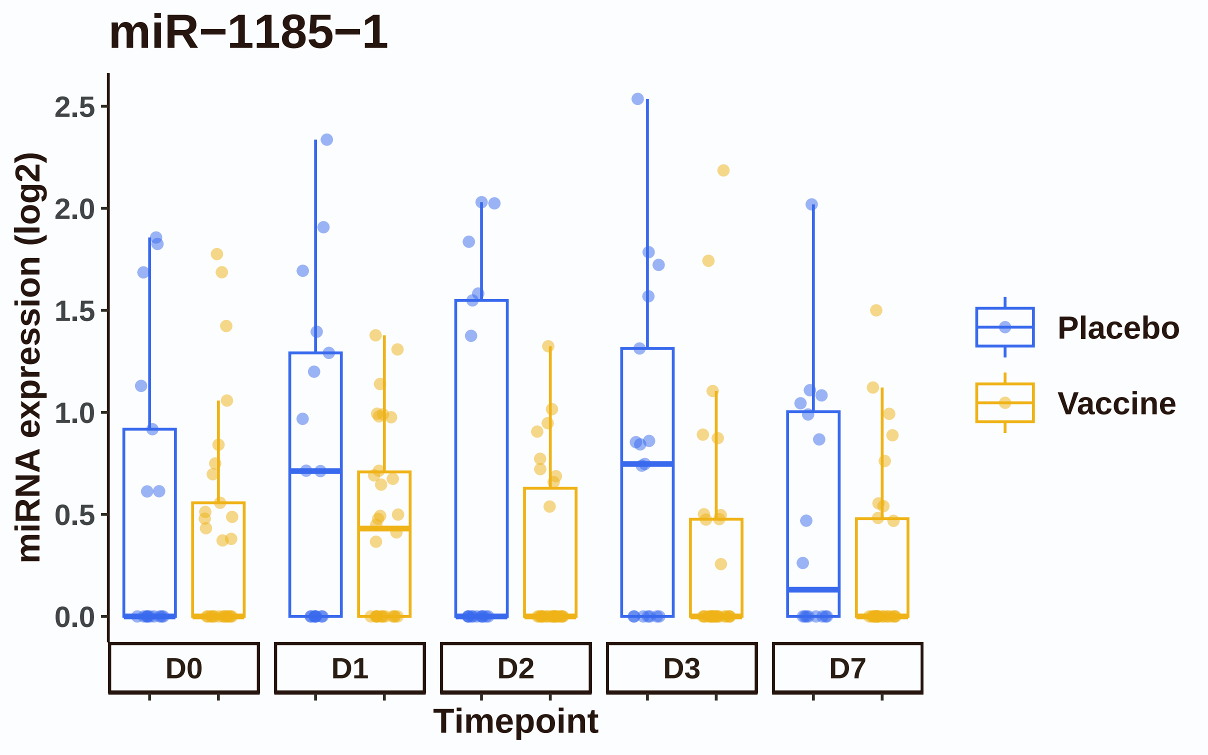

miR−1185−1
miRNA expression (log2)
2.5
2.0
1.5
1.0
0.5
0.0
D0
D1
D2
D3
D7
Timepoint
Placebo
Vaccine

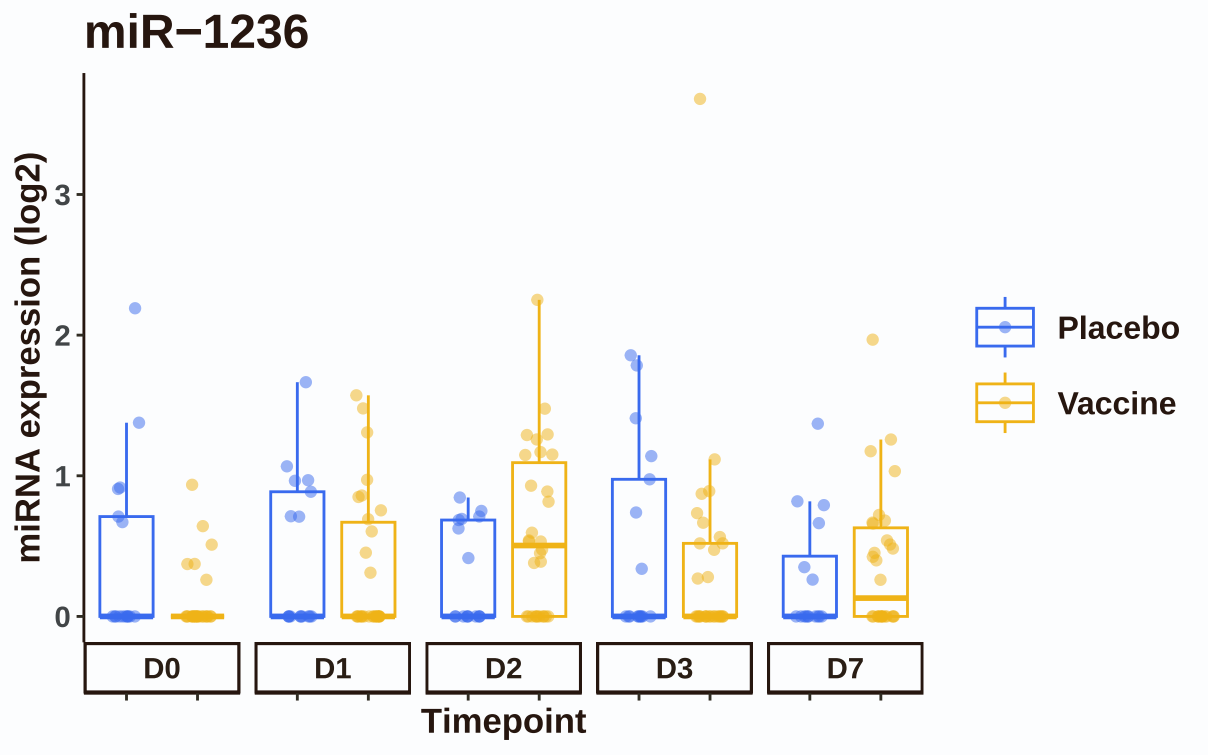
miR−1236
miRNA expression (log2)
3
2
1
0
D0
D1
D2
D3
D7
Timepoint
Placebo
Vaccine

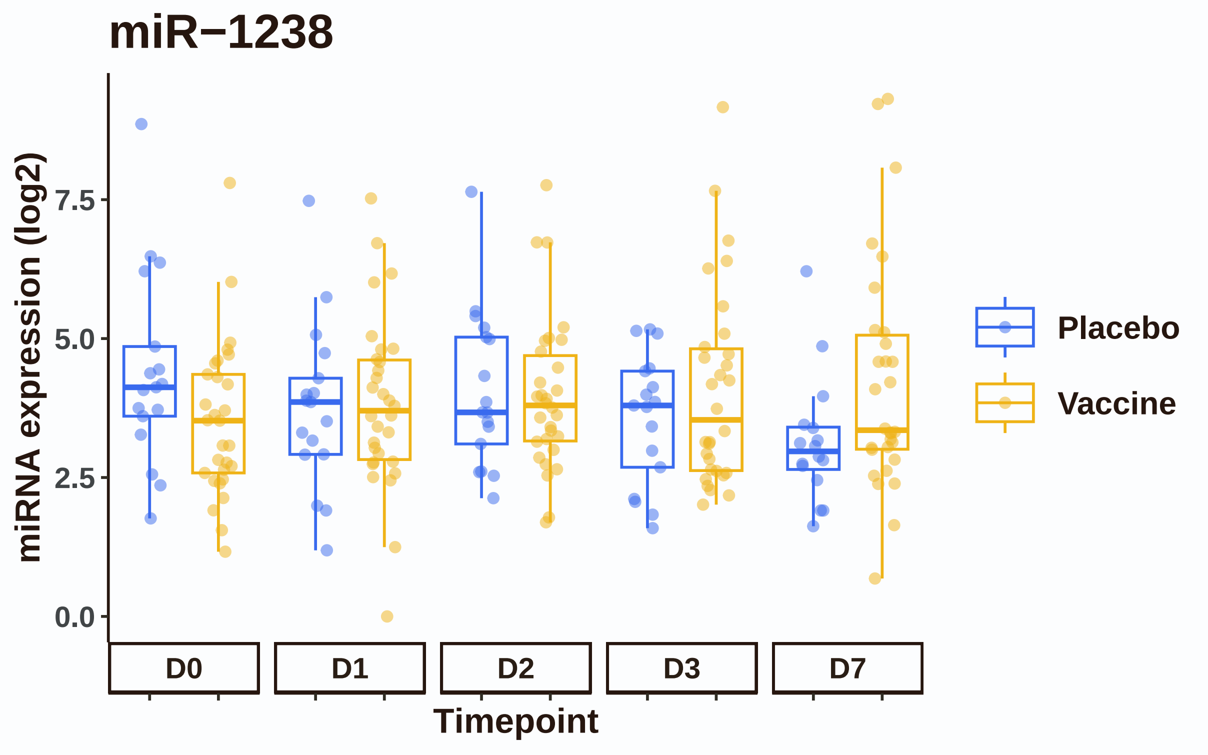
miR−1238
miRNA expression (log2)
7.5
5.0
2.5
0.0
D0
D1
D2
D3
D7
Timepoint
Placebo
Vaccine

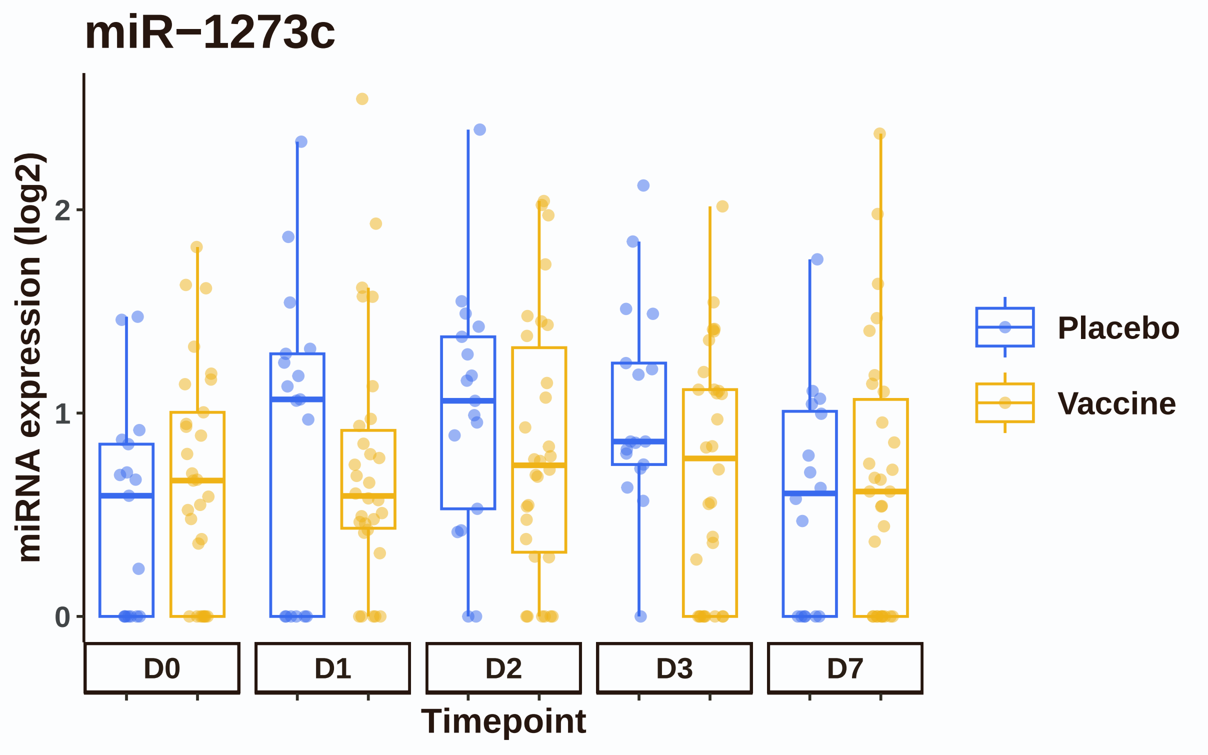

miR−1273c
miRNA expression (log2)
0
1
2
D0
D1
D2
D3
D7
Timepoint
Placebo
Vaccine

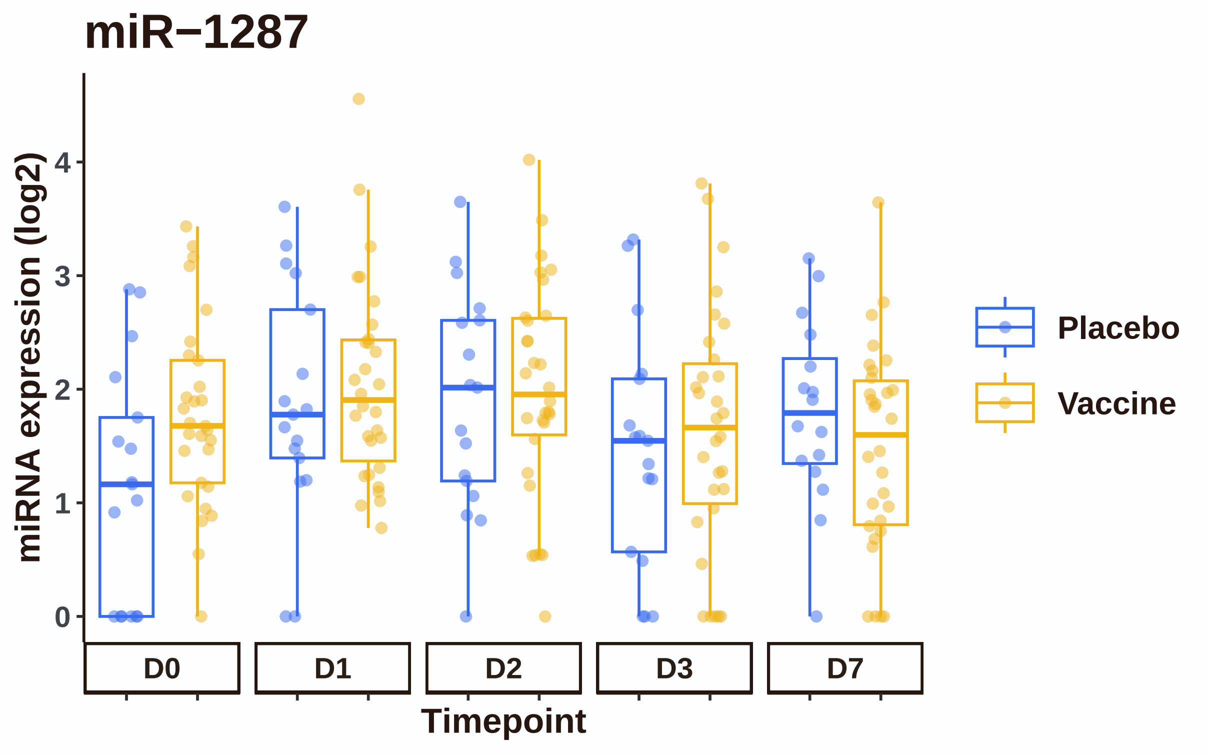

miR−1287
miRNA expression (log2)
0
1
2
3
4
D0
D1
D2
D3
D7
Timepoint
Placebo
Vaccine

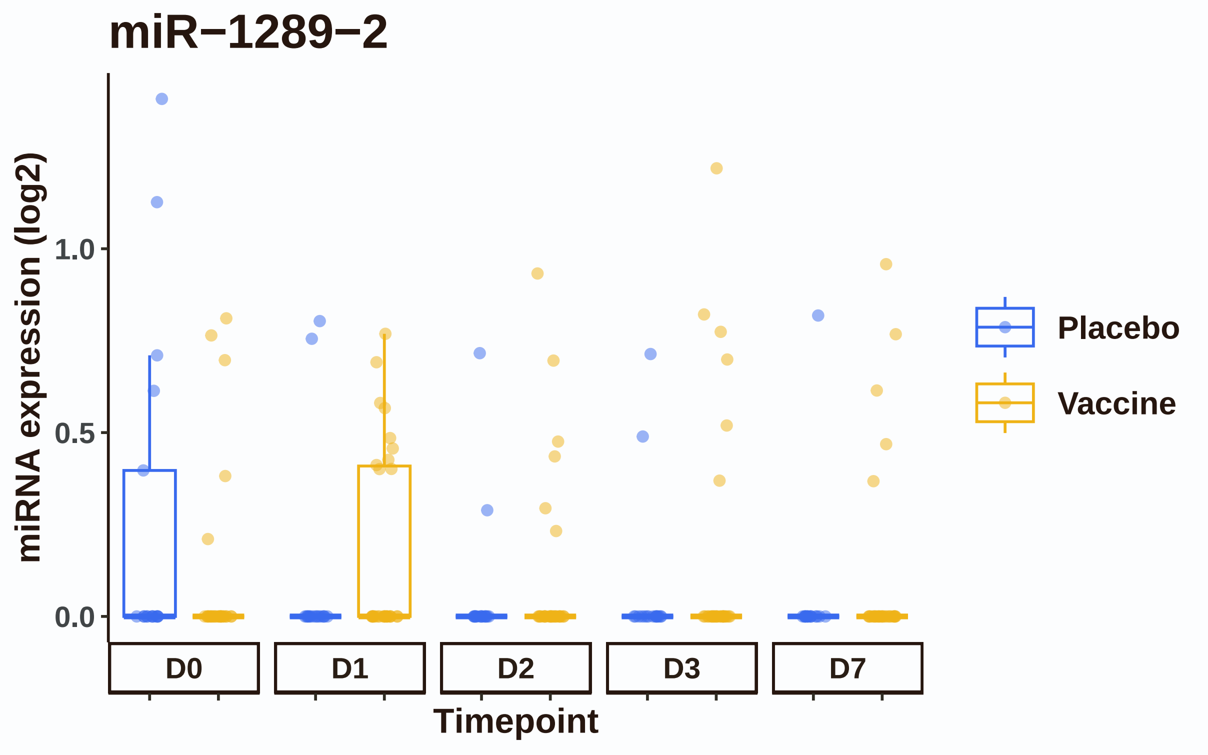

miR−1289−2
miRNA expression (log2)
1.0
0.5
0.0
D0
D1
D2
D3
D7
Timepoint
Placebo
Vaccine

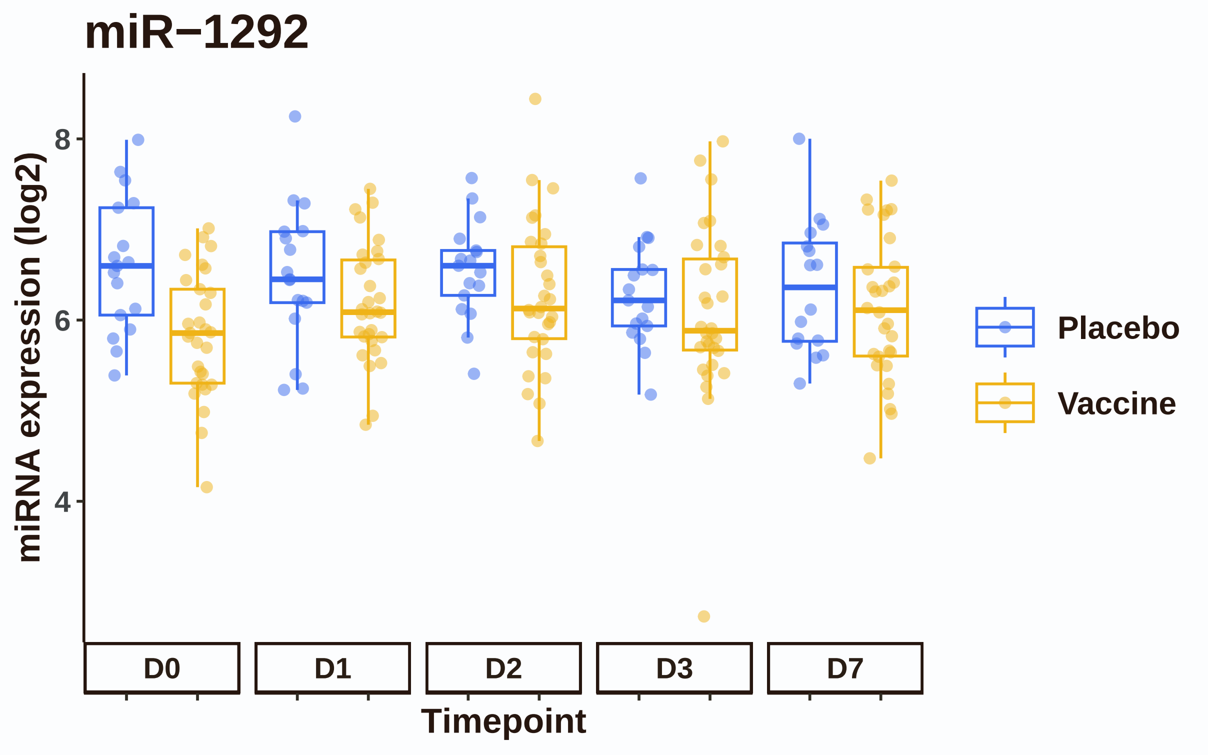
miR−1292
miRNA expression (log2)
8
6
4
D0
D1
D2
D3
D7
Timepoint
Placebo
Vaccine

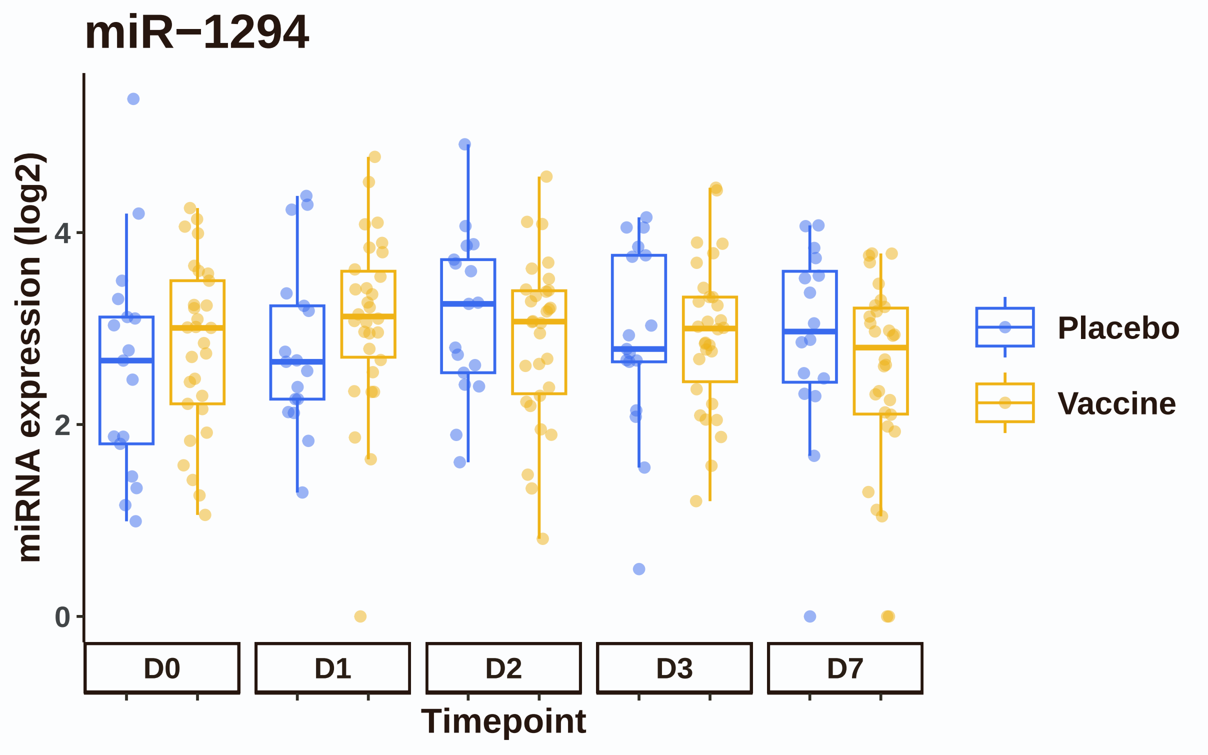

miR−1294
miRNA expression (log2)
0
2
4
D0
D1
D2
D3
D7
Timepoint
Placebo
Vaccine

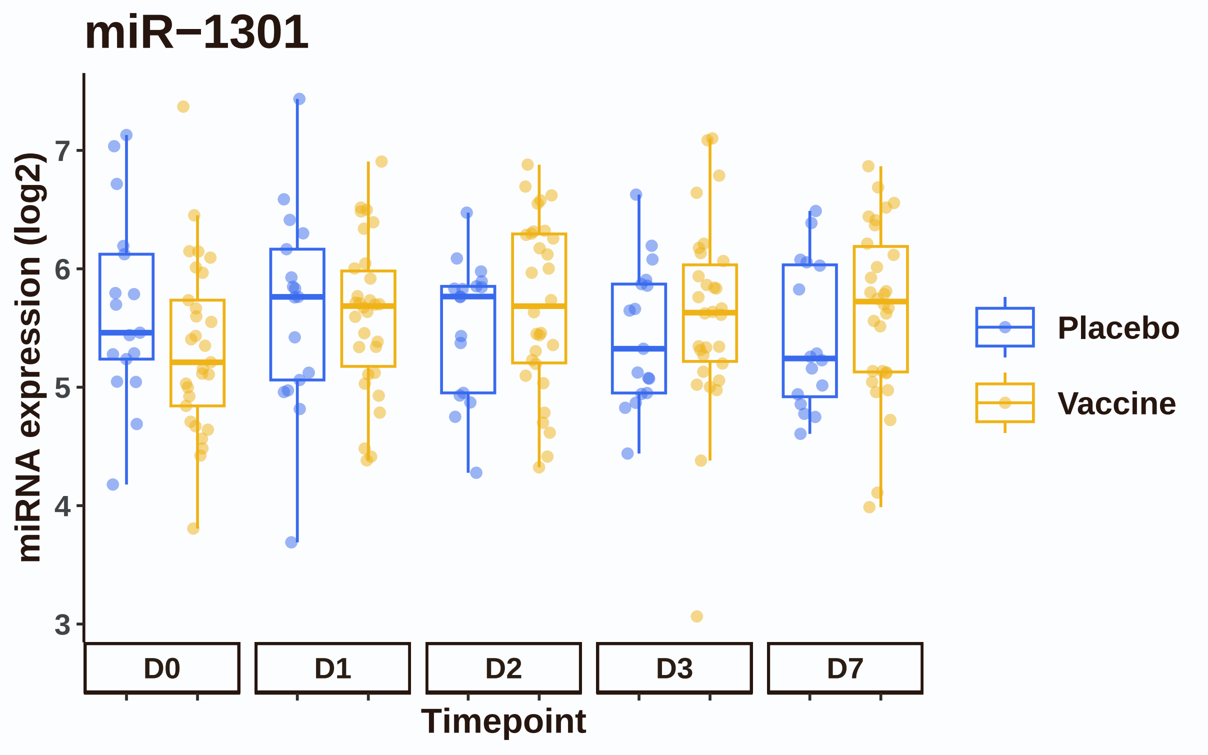
miR−1301
miRNA expression (log2)
7
6
5
4
3
D0
D1
D2
D3
D7
Timepoint
Placebo
Vaccine

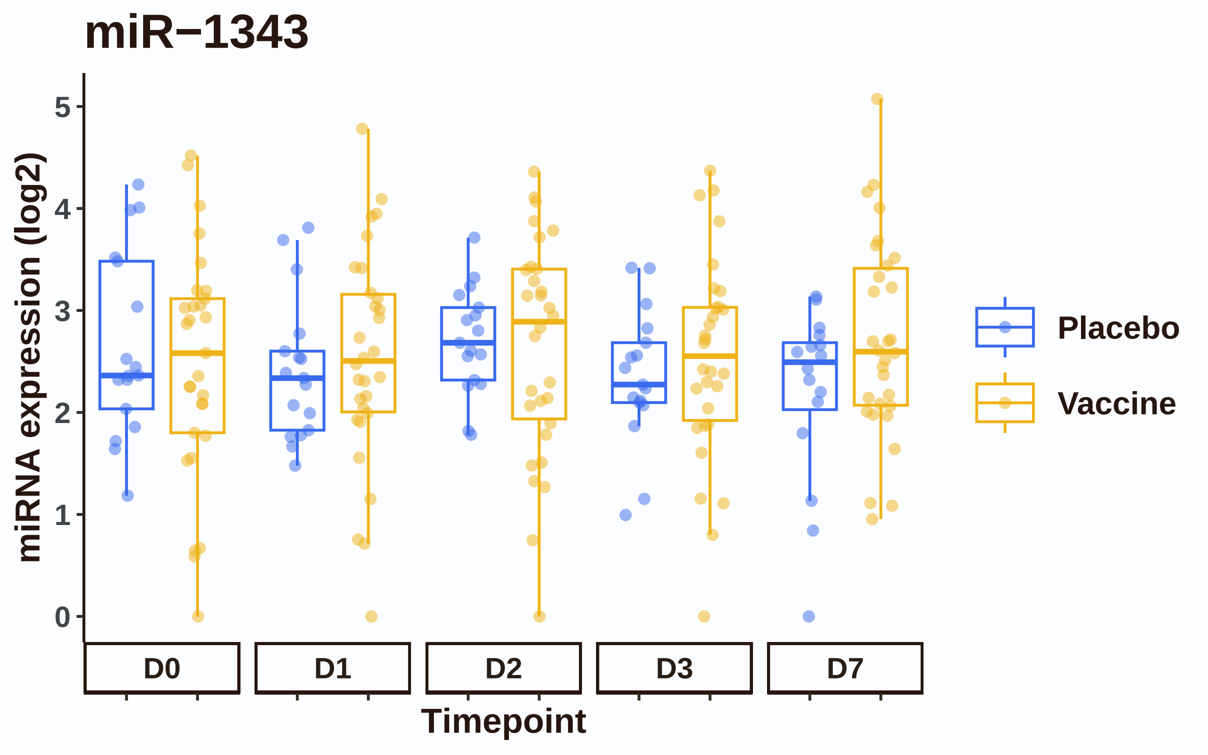
miR−1343
miRNA expression (log2)
0
1
2
3
4
5
D0
D1
D2
D3
D7
Timepoint
Placebo
Vaccine

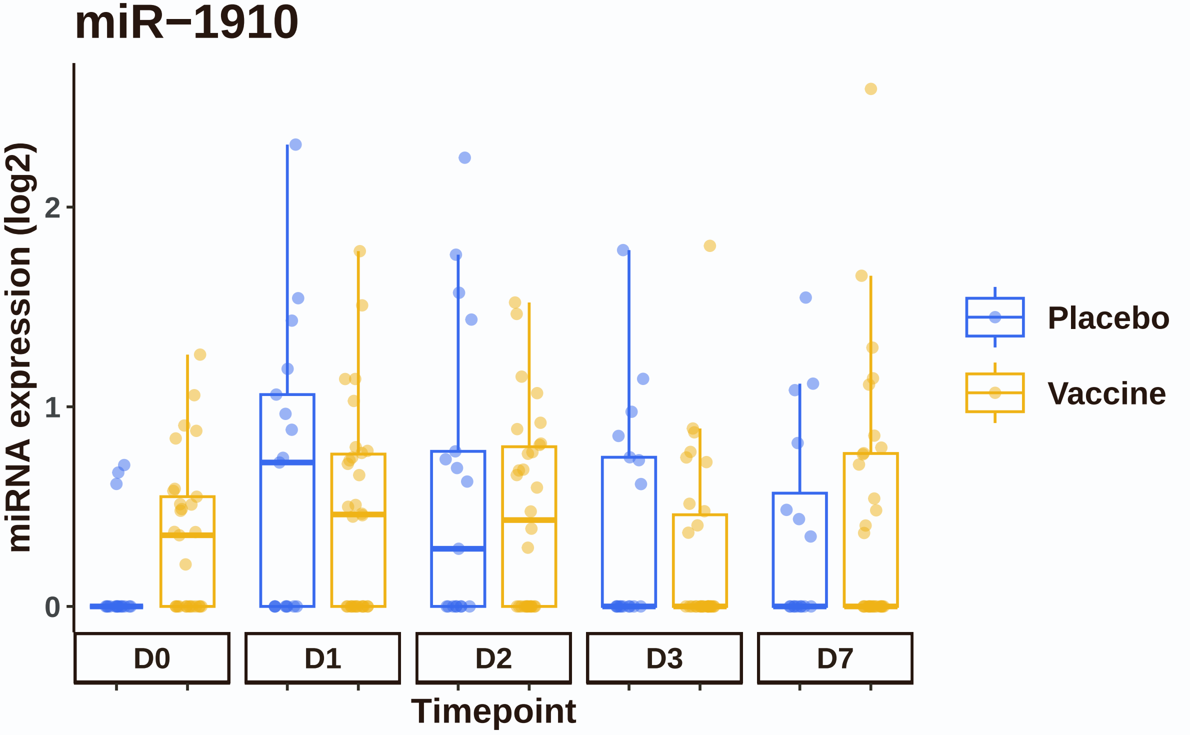
miR−1910
miRNA expression (log2)
0
1
2
D0
D1
D2
D3
D7
Timepoint
Placebo
Vaccine

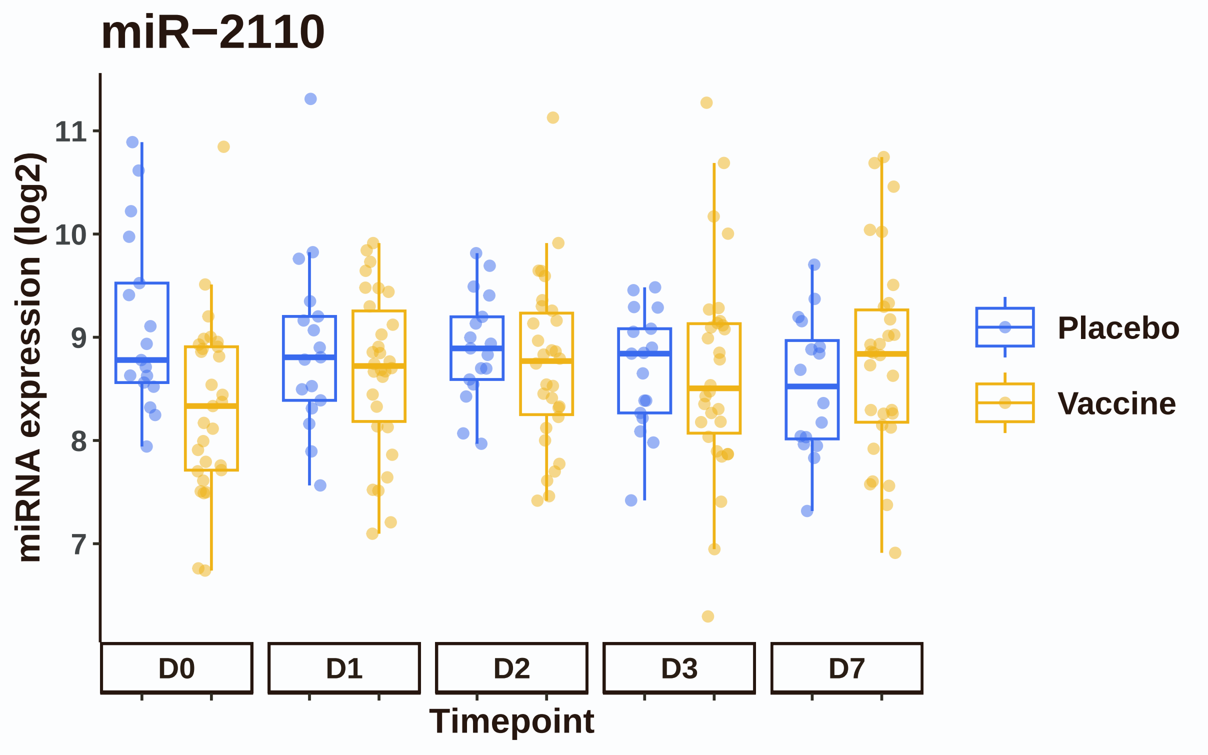

miR−2110
miRNA expression (log2)
11
10
9
8
7
D0
D1
D2
D3
D7
Timepoint
Placebo
Vaccine

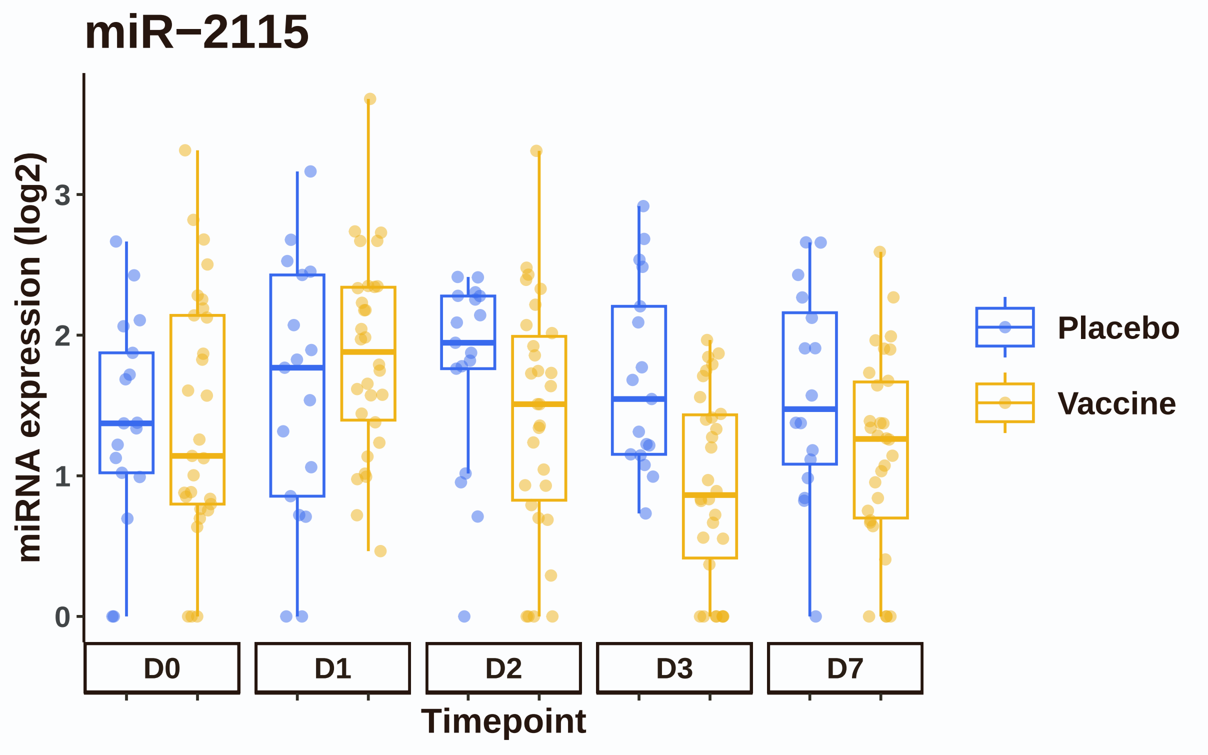
miR−2115
miRNA expression (log2)
0
1
2
3
D0
D1
D2
D3
D7
Timepoint
Placebo
Vaccine

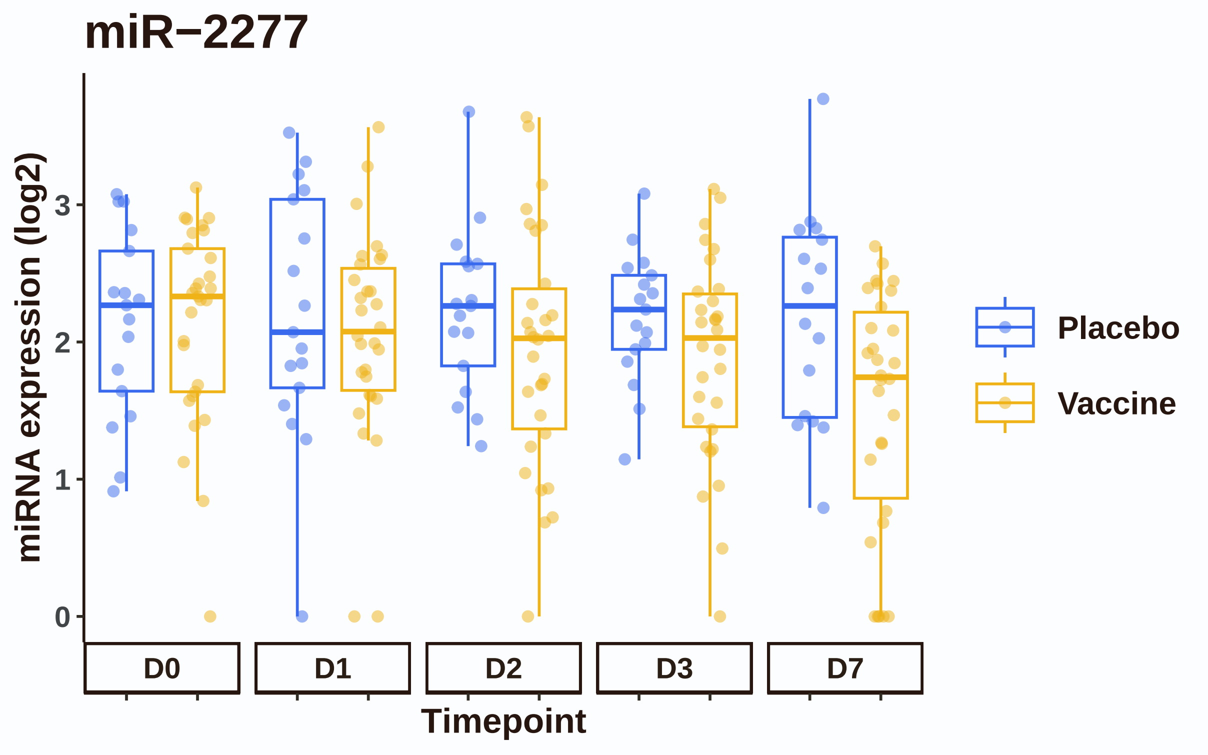

miR−2277
miRNA expression (log2)
0
1
2
3
D0
D1
D2
D3
D7
Timepoint
Placebo
Vaccine

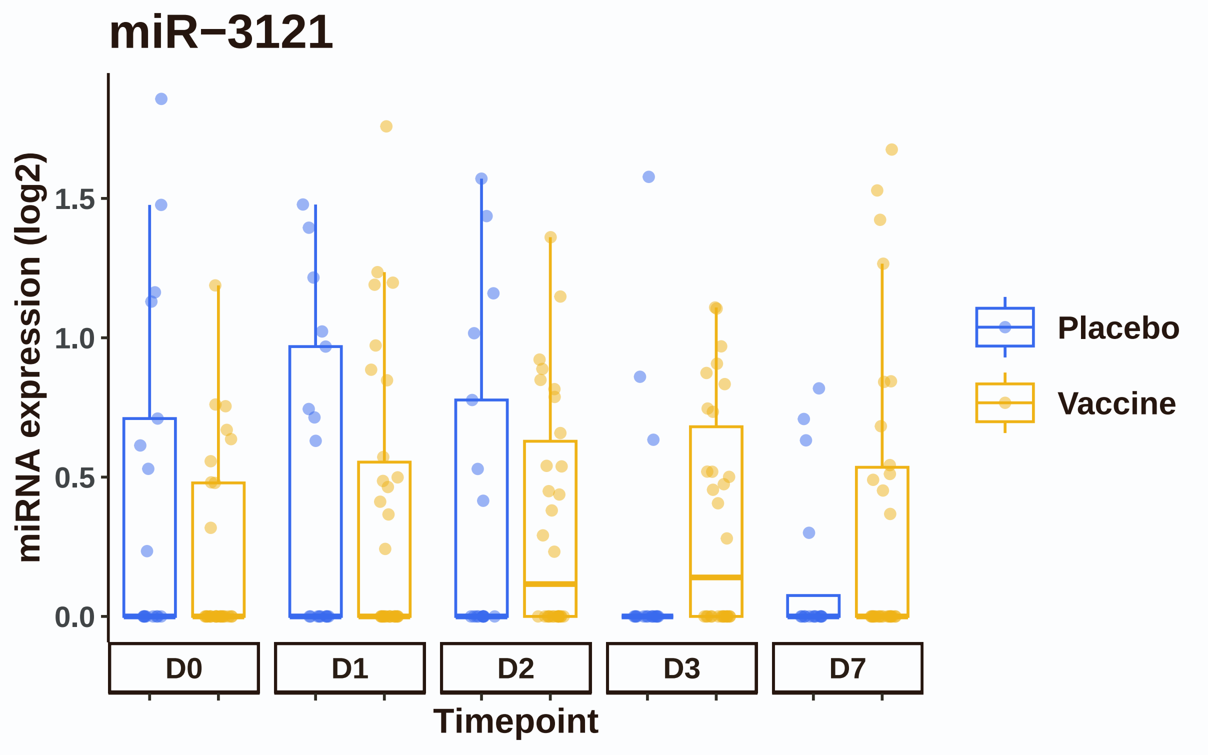

miR−3121
miRNA expression (log2)
0.0
0.5
1.0
1.5
D0
D1
D2
D3
D7
Timepoint
Placebo
Vaccine

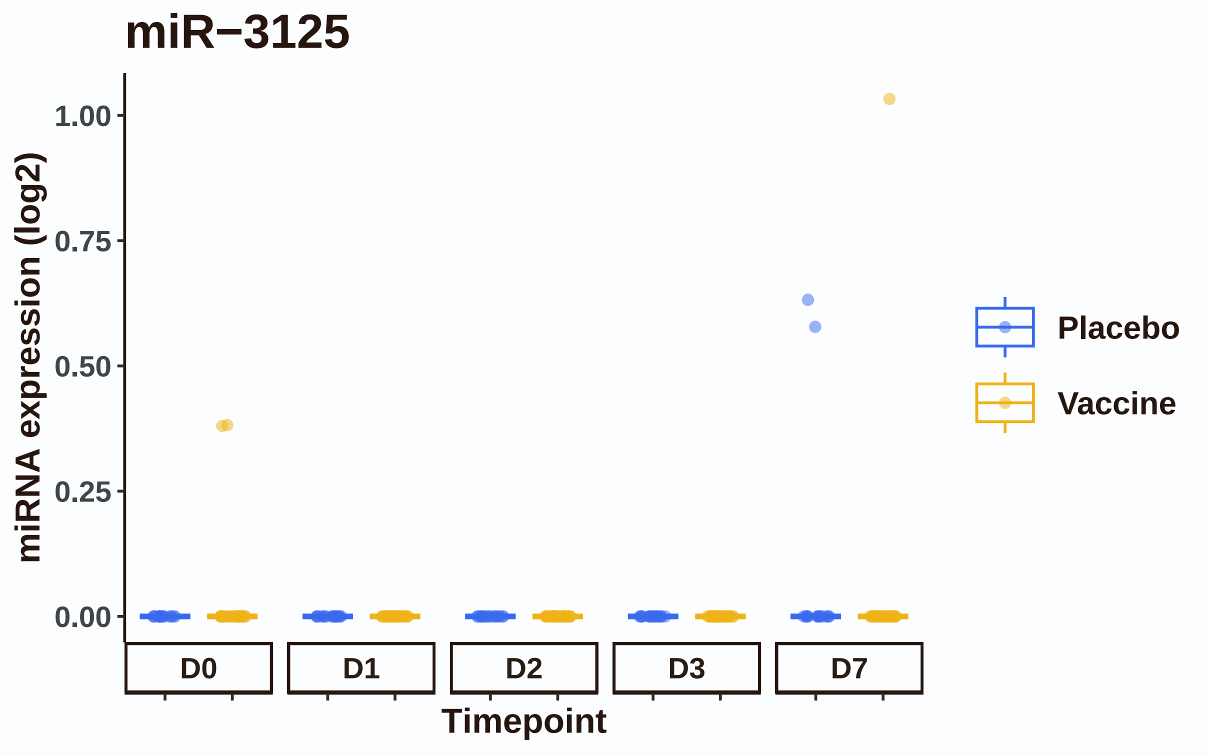
miR−3125
miRNA expression (log2)
1.00
0.75
0.50
0.25
0.00
D0
D1
D2
D3
D7
Timepoint
Placebo
Vaccine

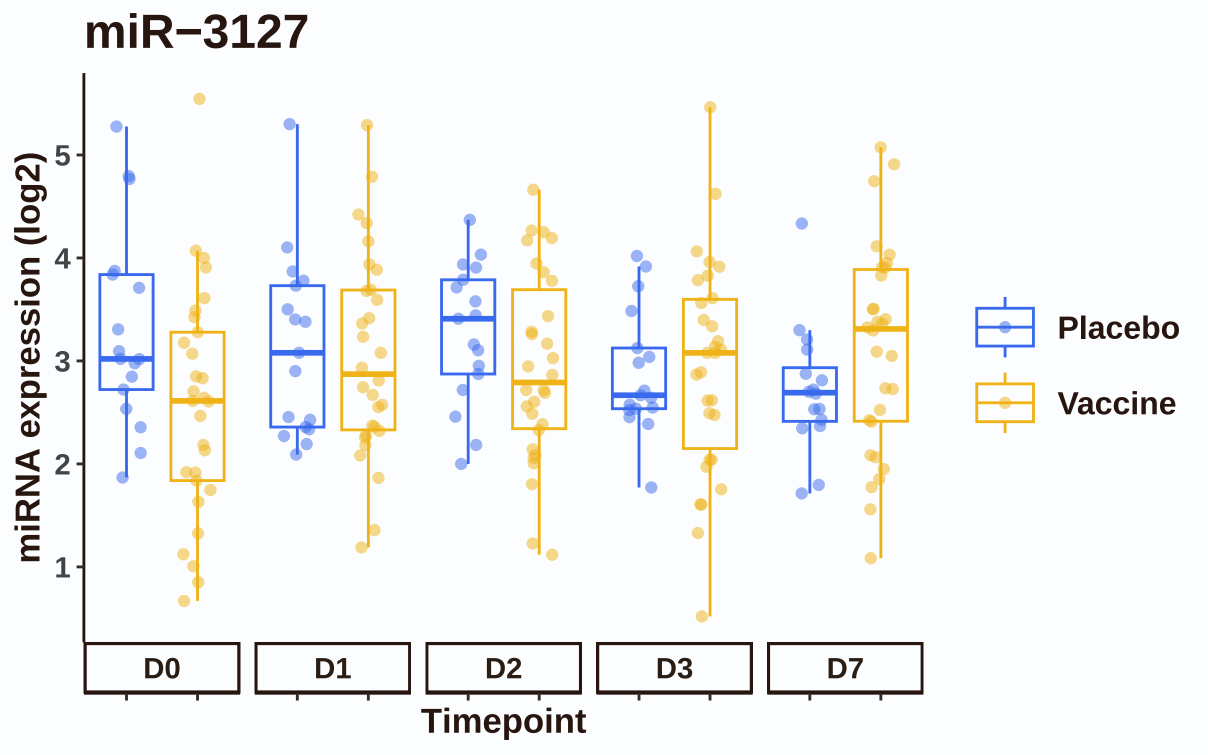
miR−3127
miRNA expression (log2)
5
4
3
2
1
D0
D1
D2
D3
D7
Timepoint
Placebo
Vaccine

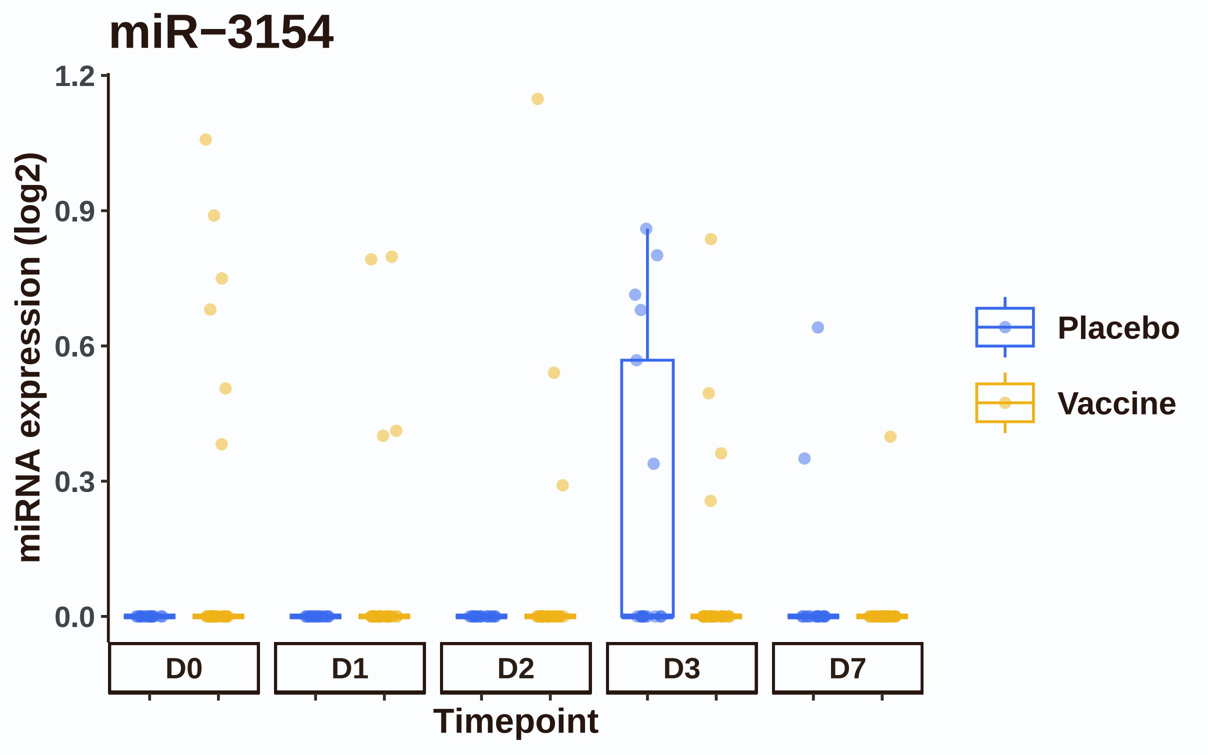
miR−3154
miRNA expression (log2)
1.2
0.9
0.6
0.3
0.0
D0
D1
D2
D3
D7
Timepoint
Placebo
Vaccine

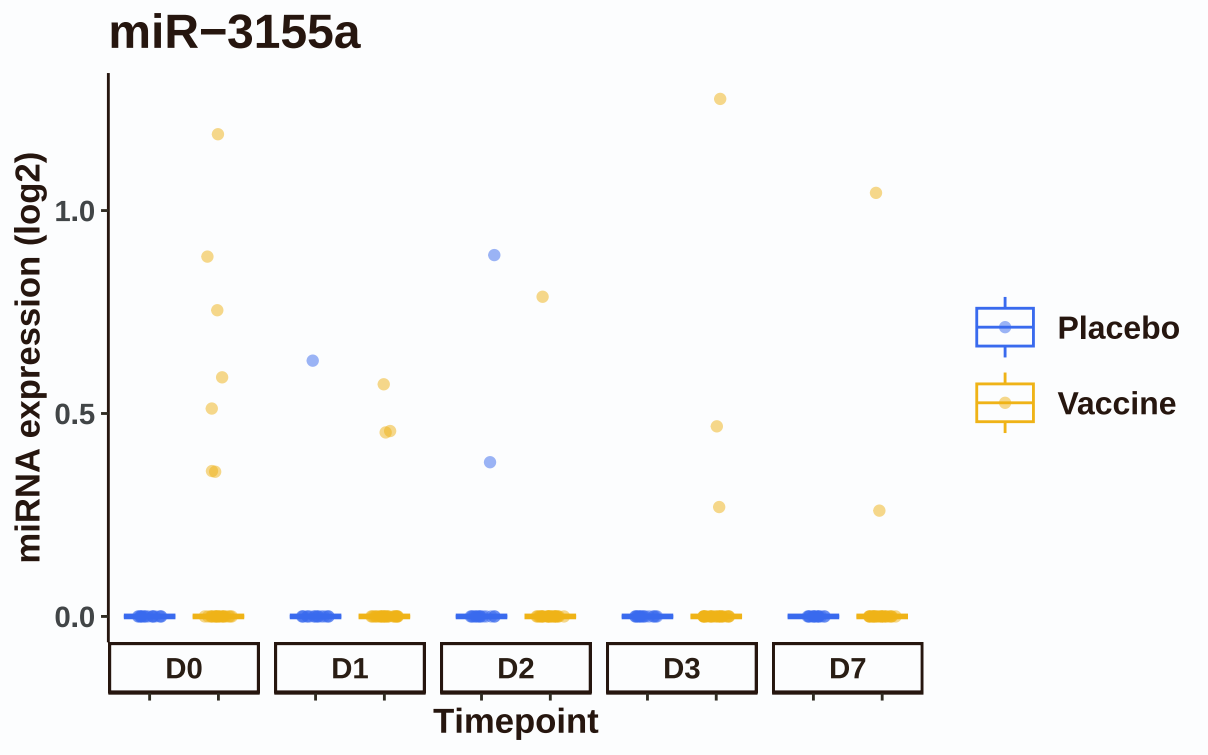

miR−3155a
miRNA expression (log2)
1.0
0.5
0.0
D0
D1
D2
D3
D7
Timepoint
Placebo
Vaccine

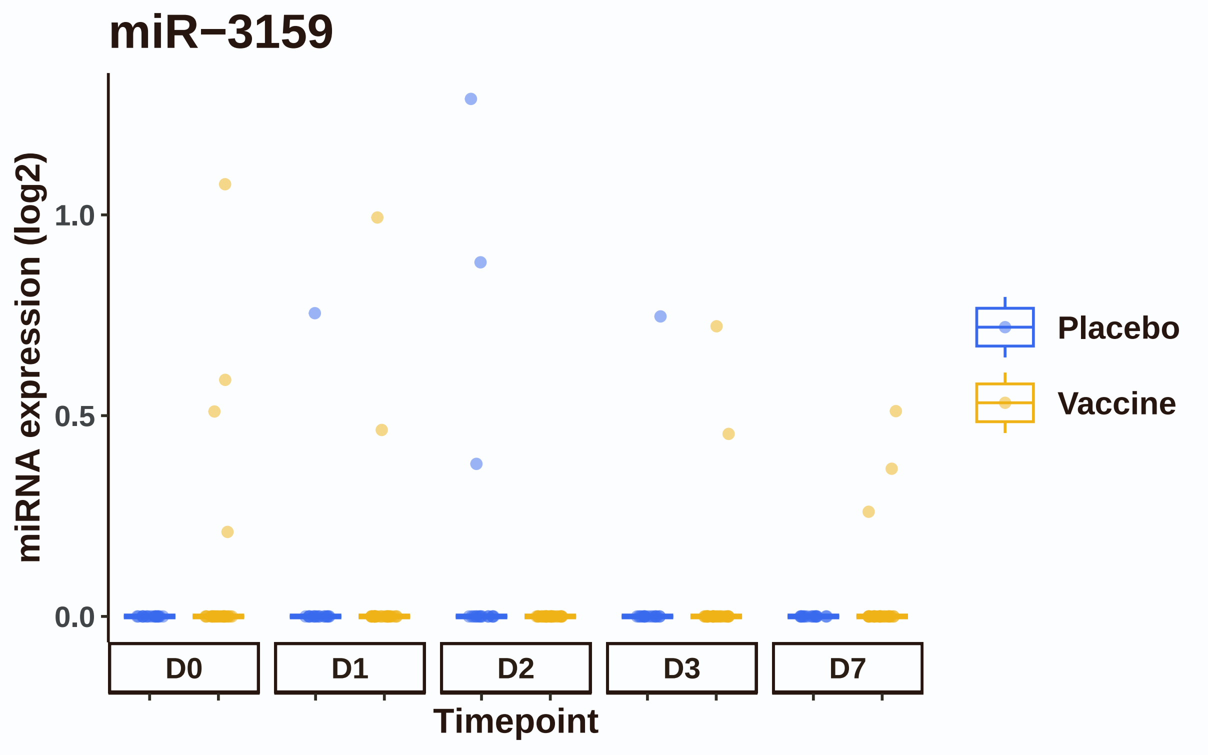
miR−3159
miRNA expression (log2)
1.0
0.5
0.0
D0
D1
D2
D3
D7
Timepoint
Placebo
Vaccine

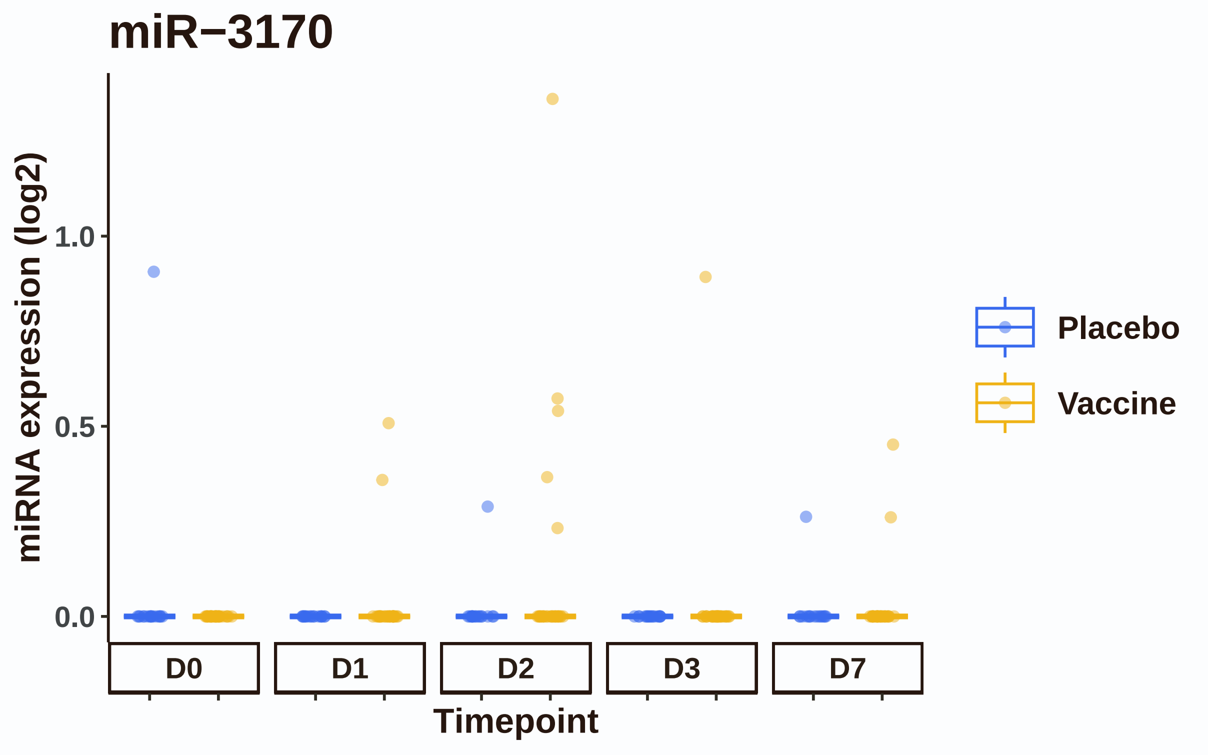
miR−3170
miRNA expression (log2)
1.0
0.5
0.0
D0
D1
D2
D3
D7
Timepoint
Placebo
Vaccine

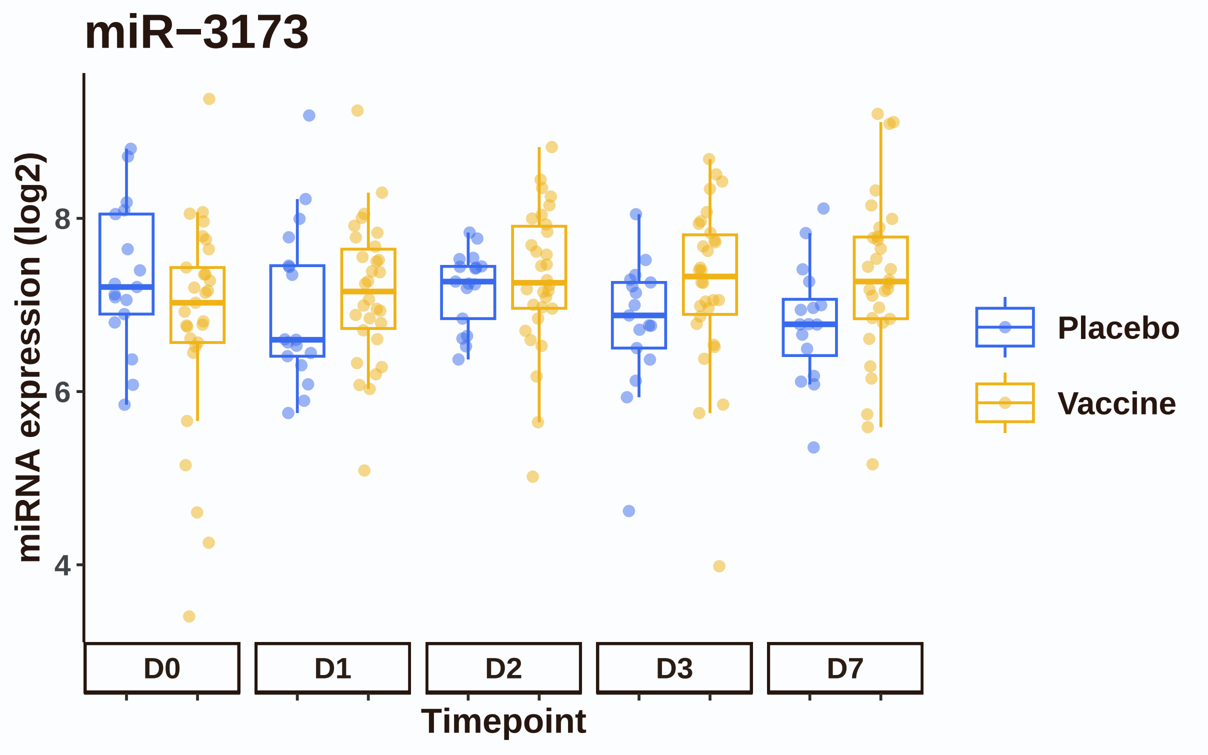
miR−3173
miRNA expression (log2)
8
6
4
D0
D1
D2
D3
D7
Timepoint
Placebo
Vaccine

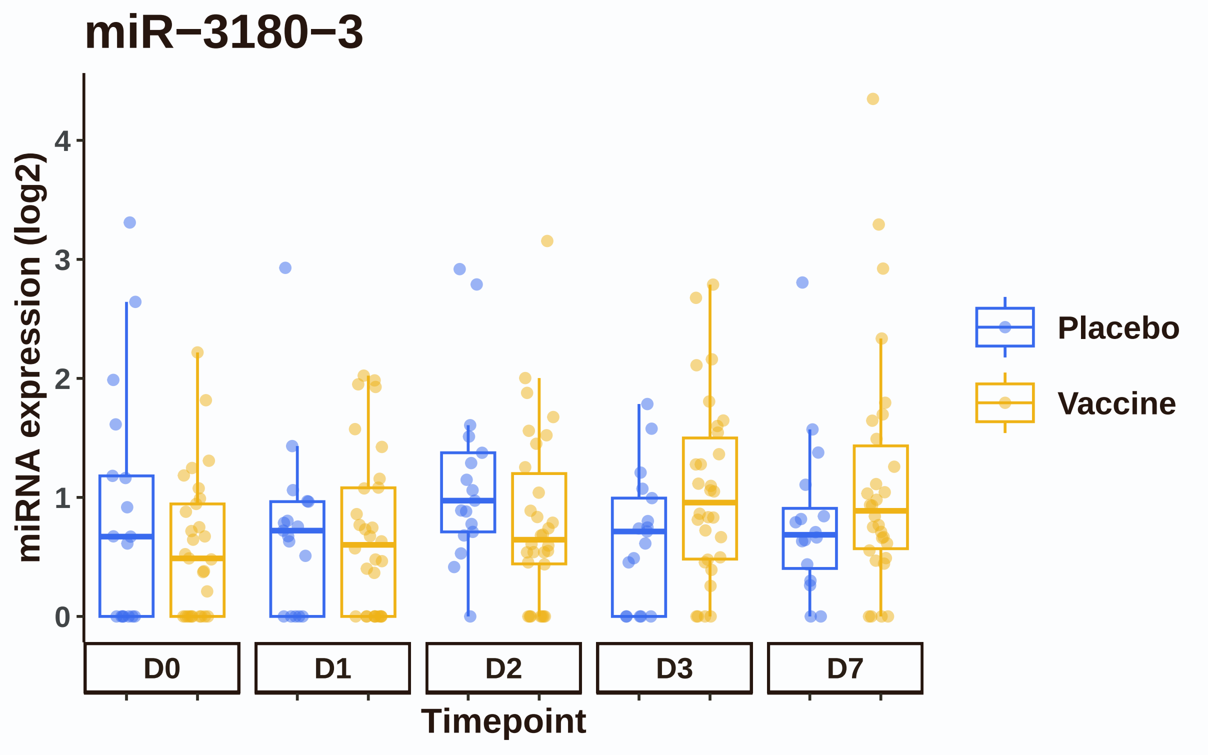
miR−3180−3
miRNA expression (log2)
0
1
2
3
4
D0
D1
D2
D3
D7
Timepoint
Placebo
Vaccine

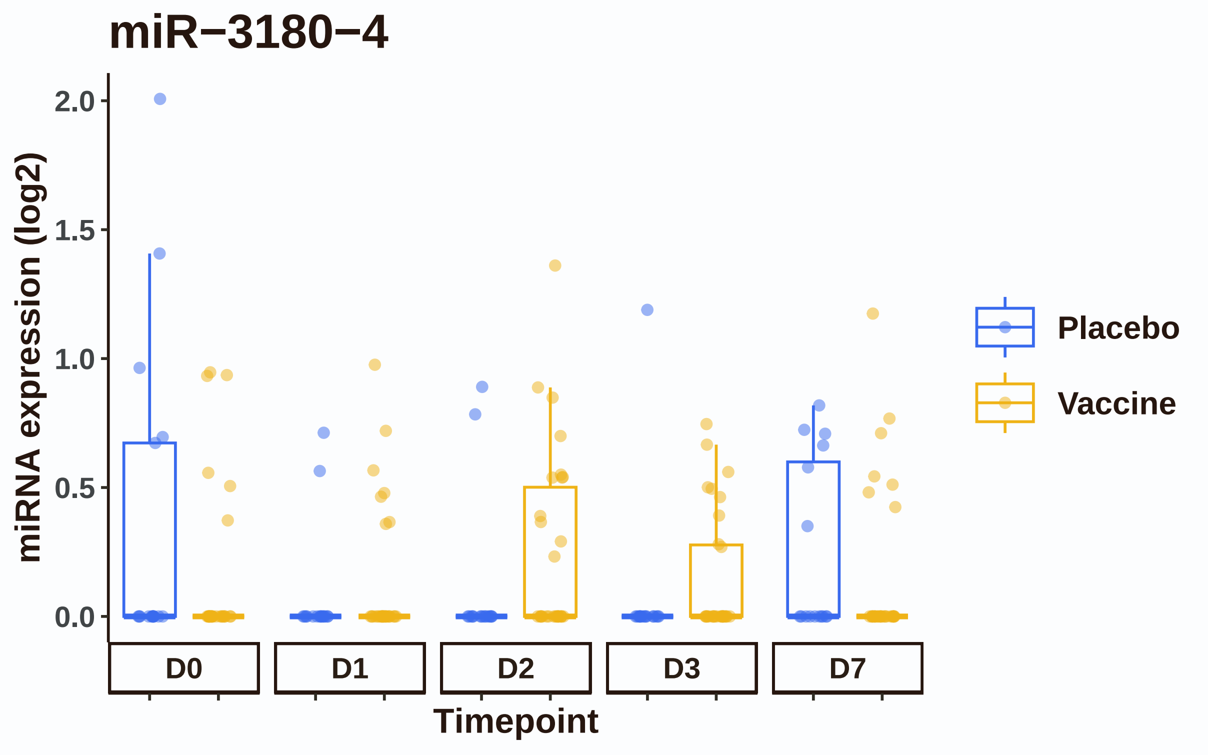
miR−3180−4
miRNA expression (log2)
2.0
1.5
1.0
0.5
0.0
D0
D1
D2
D3
D7
Timepoint
Placebo
Vaccine

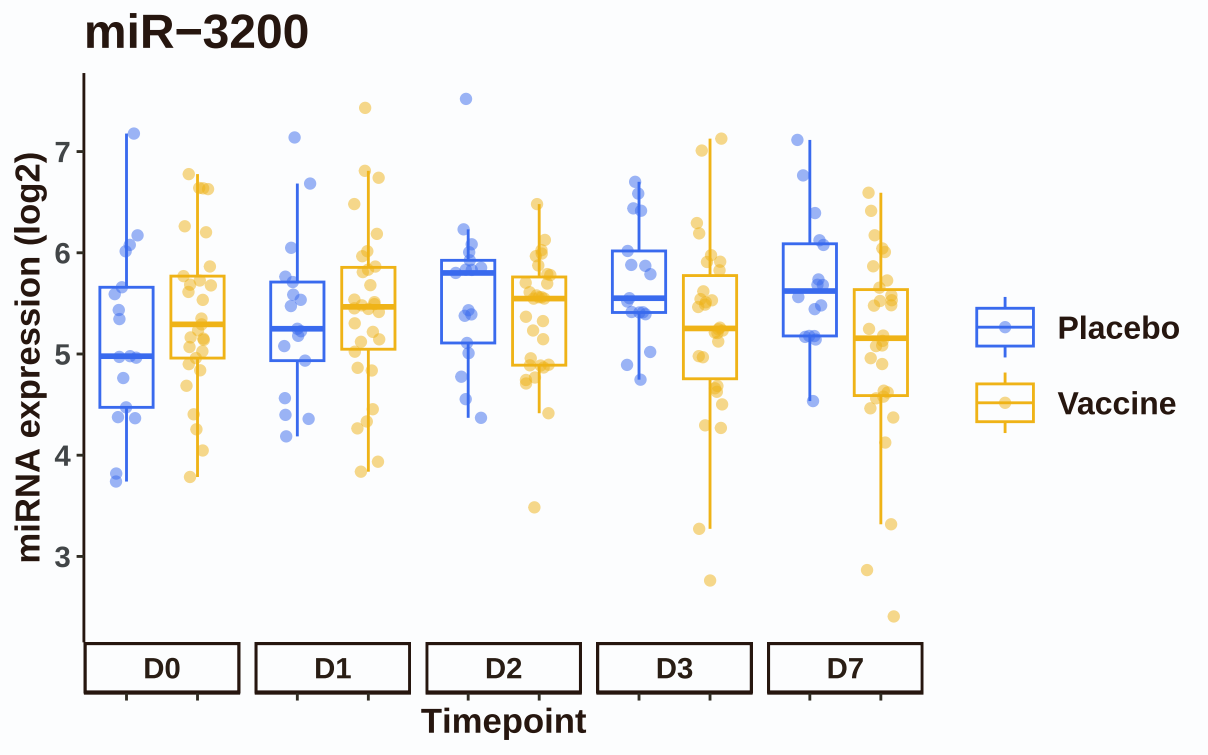

miR−3200
miRNA expression (log2)
7
6
5
4
3
D0
D1
D2
D3
D7
Timepoint
Placebo
Vaccine

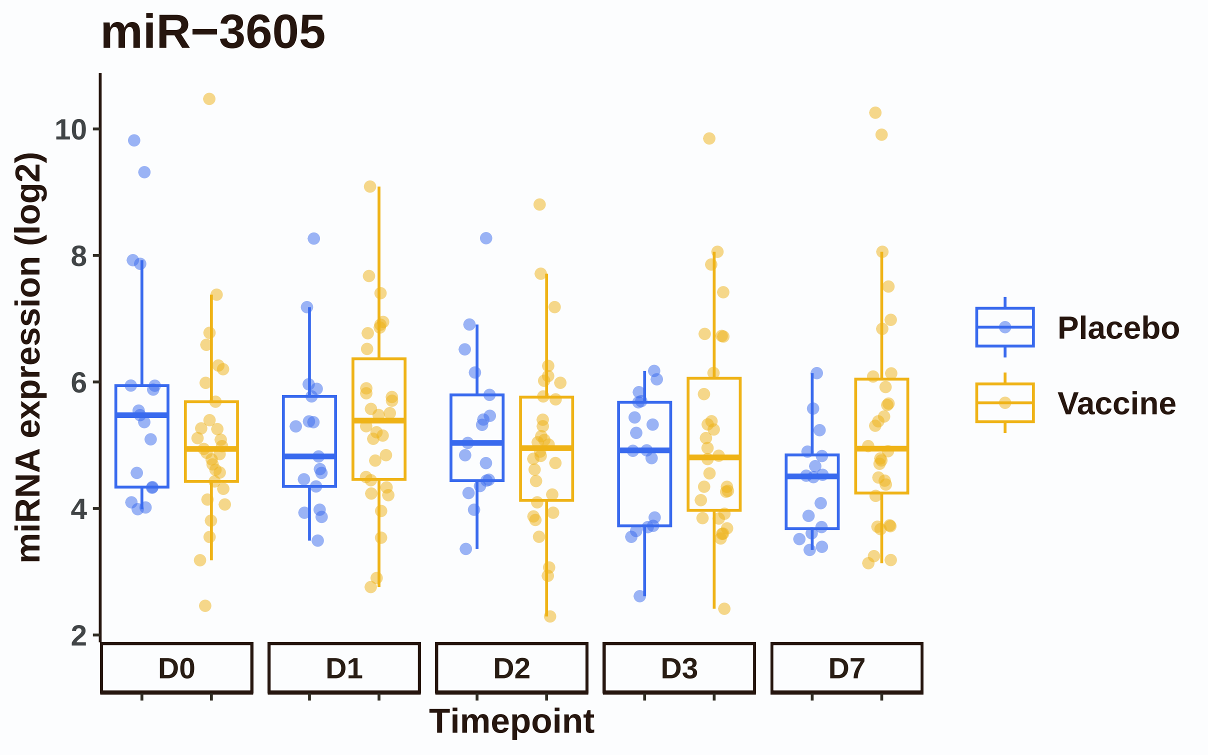
miR−3605
miRNA expression (log2)
10
8
6
4
2
D0
D1
D2
D3
D7
Timepoint
Placebo
Vaccine

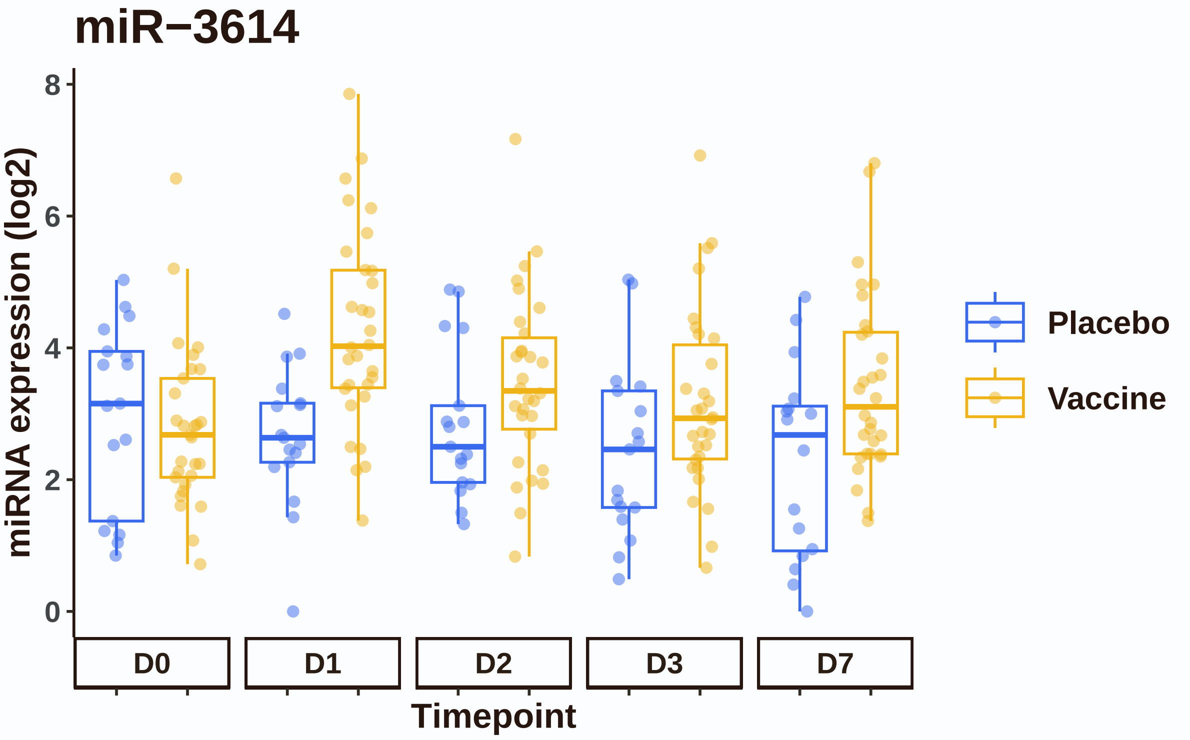

miR−3614
miRNA expression (log2)
8
6
4
2
0
D0
D1
D2
D3
D7
Timepoint
Placebo
Vaccine

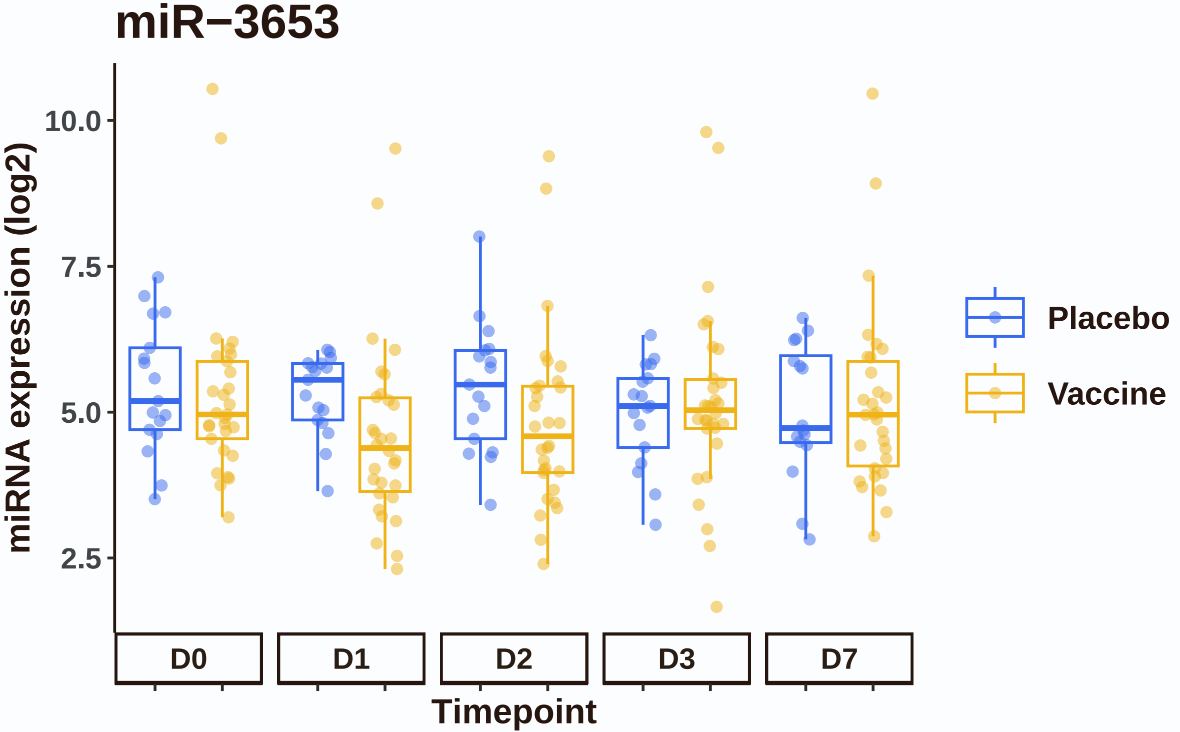

miR−3653
miRNA expression (log2)
10.0
7.5
5.0
2.5
D0
D1
D2
D3
D7
Timepoint
Placebo
Vaccine

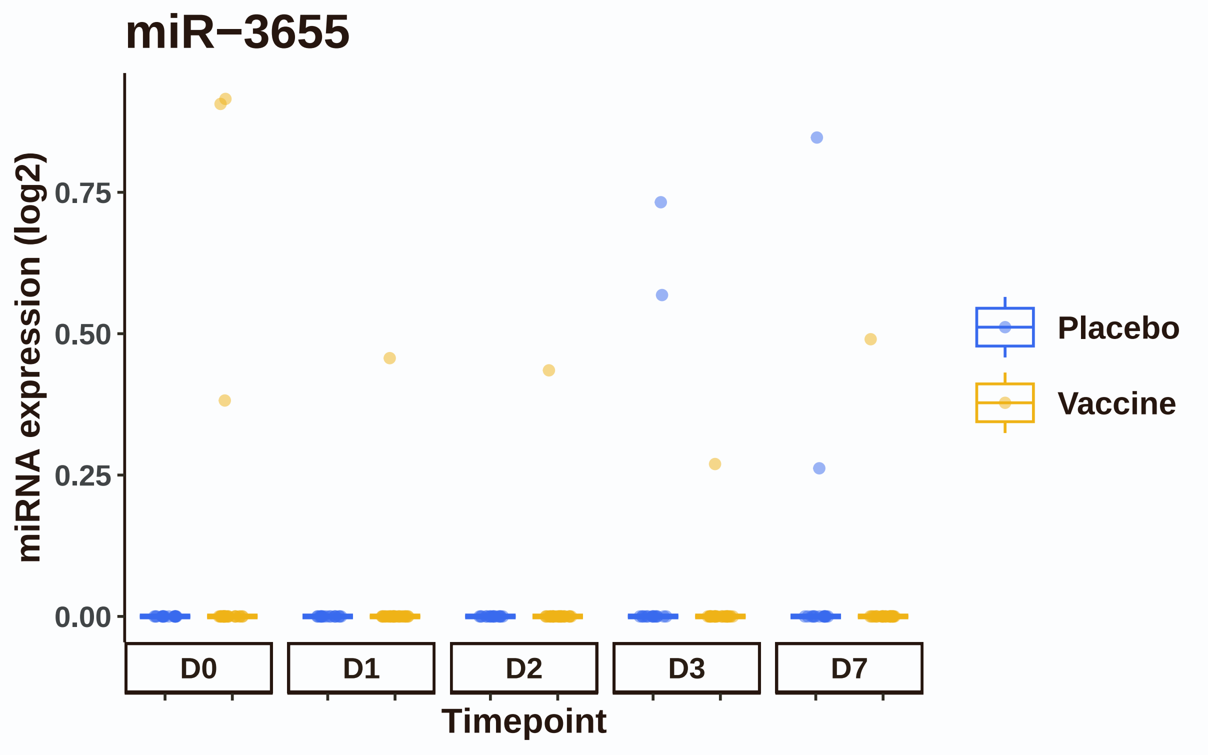
miR−3655
miRNA expression (log2)
0.75
0.50
0.25
0.00
D0
D1
D2
D3
D7
Timepoint
Placebo
Vaccine

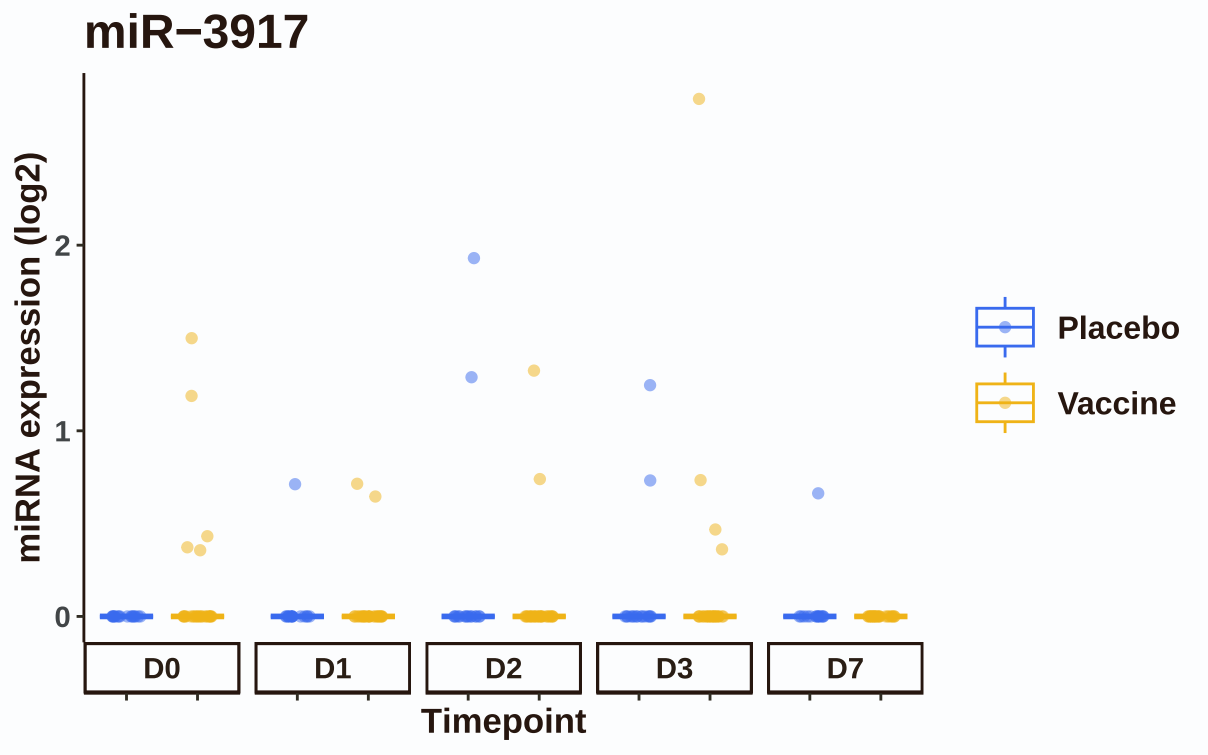

miR−3917
miRNA expression (log2)
0
1
2
D0
D1
D2
D3
D7
Timepoint
Placebo
Vaccine

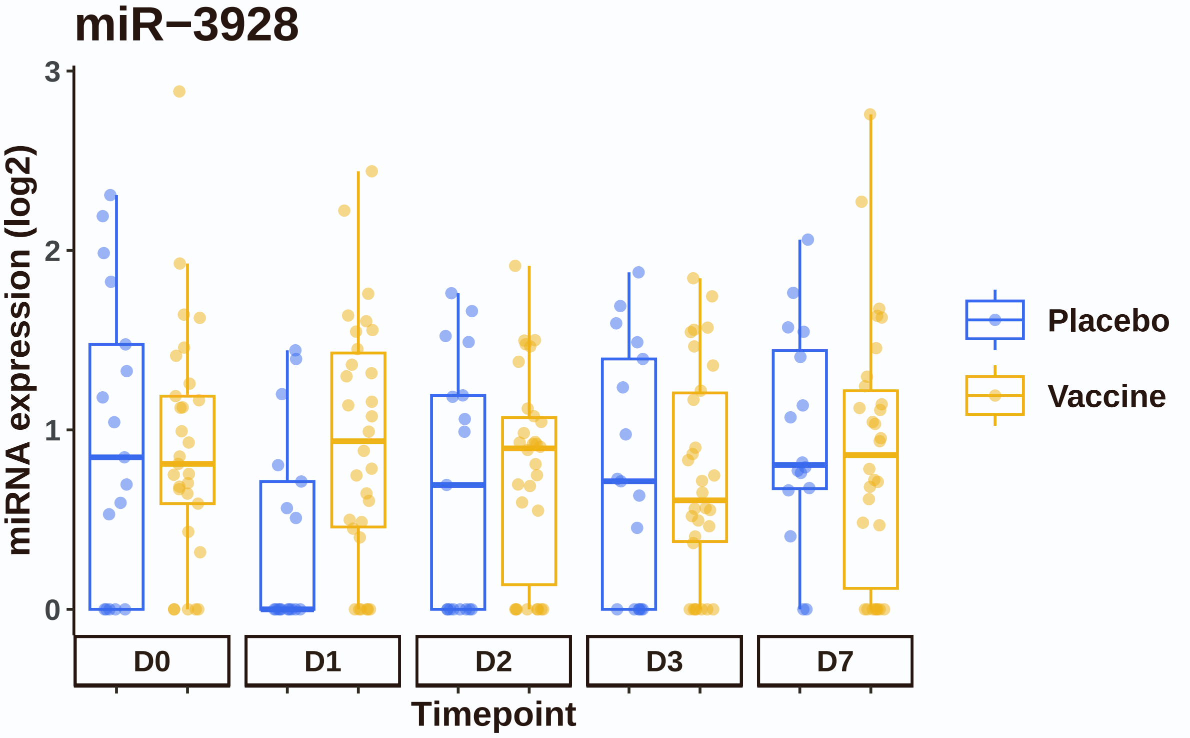

miR−3928
miRNA expression (log2)
3
2
1
0
D0
D1
D2
D3
D7
Timepoint
Placebo
Vaccine

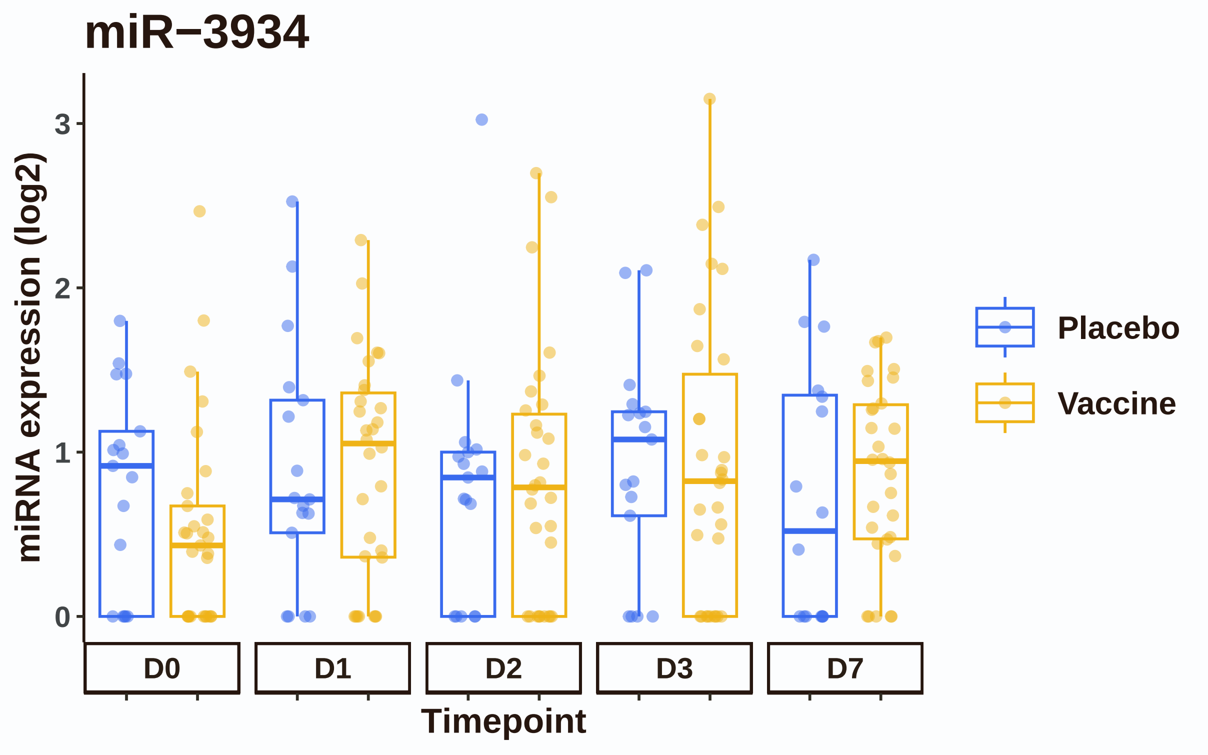
miR−3934
miRNA expression (log2)
3
2
1
0
D0
D1
D2
D3
D7
Timepoint
Placebo
Vaccine

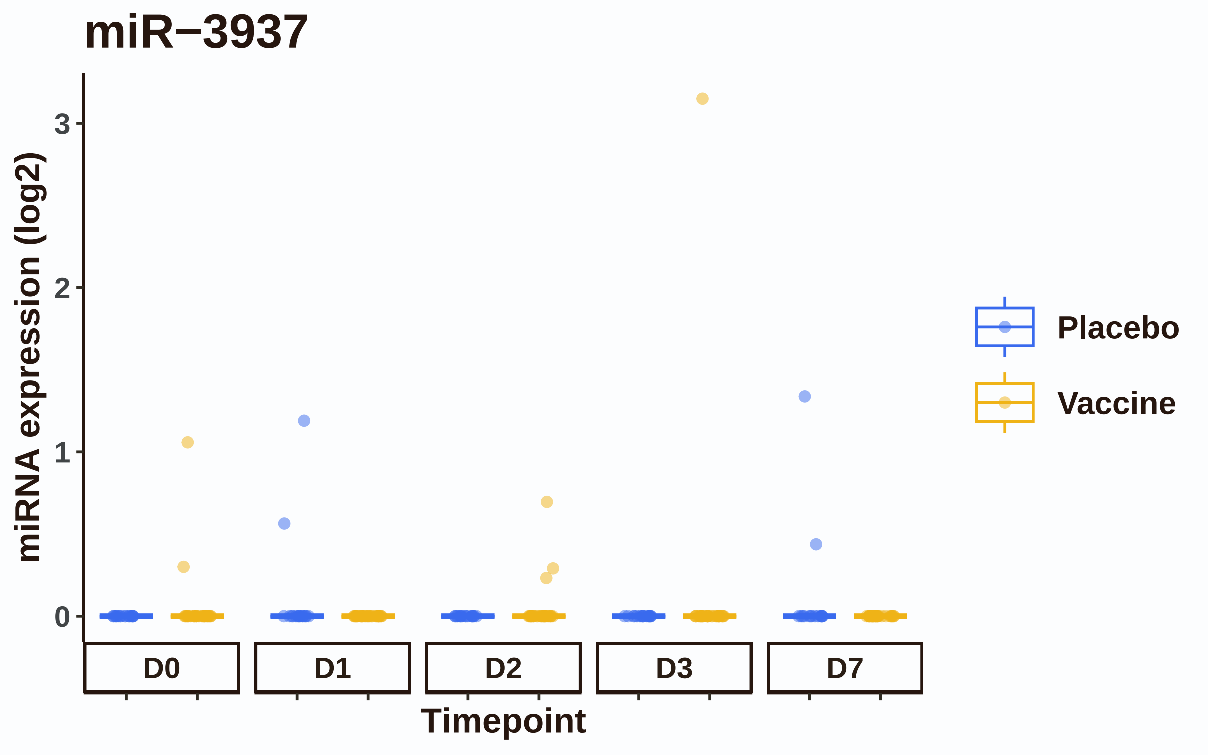
miR−3937
miRNA expression (log2)
3
2
1
0
D0
D1
D2
D3
D7
Timepoint
Placebo
Vaccine

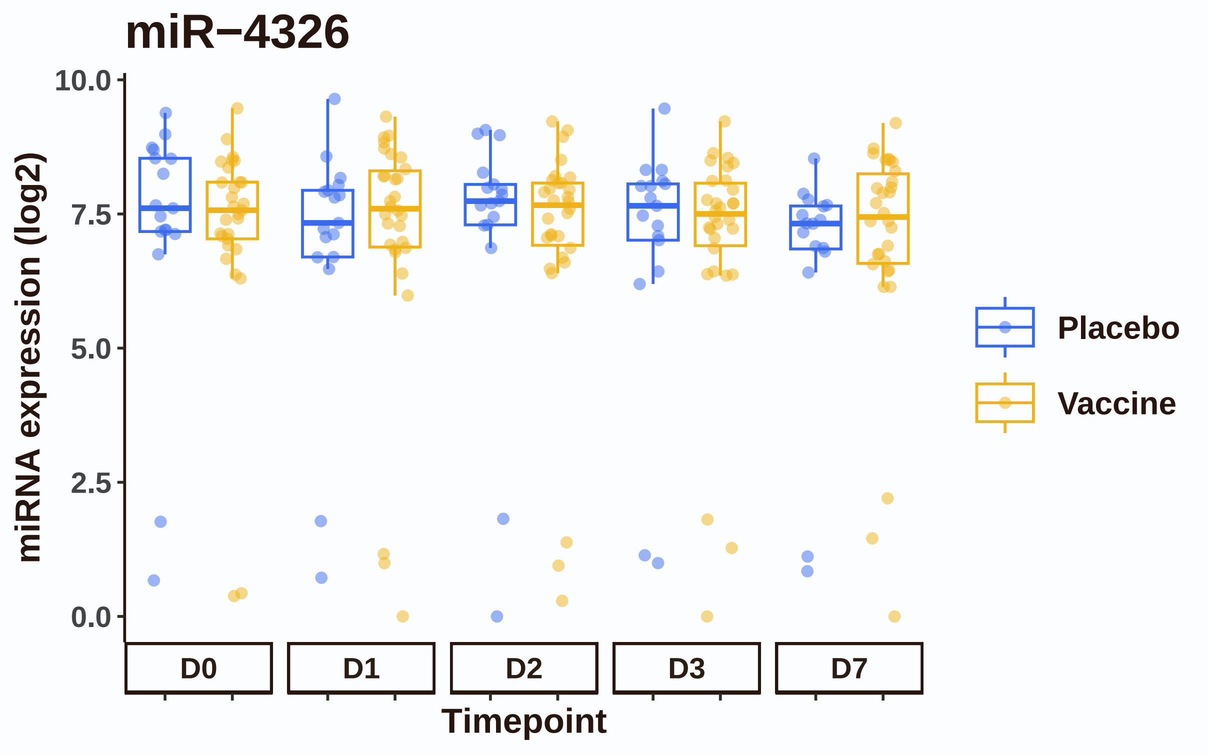

miR−4326
miRNA expression (log2)
10.0
7.5
5.0
2.5
0.0
D0
D1
D2
D3
D7
Timepoint
Placebo
Vaccine

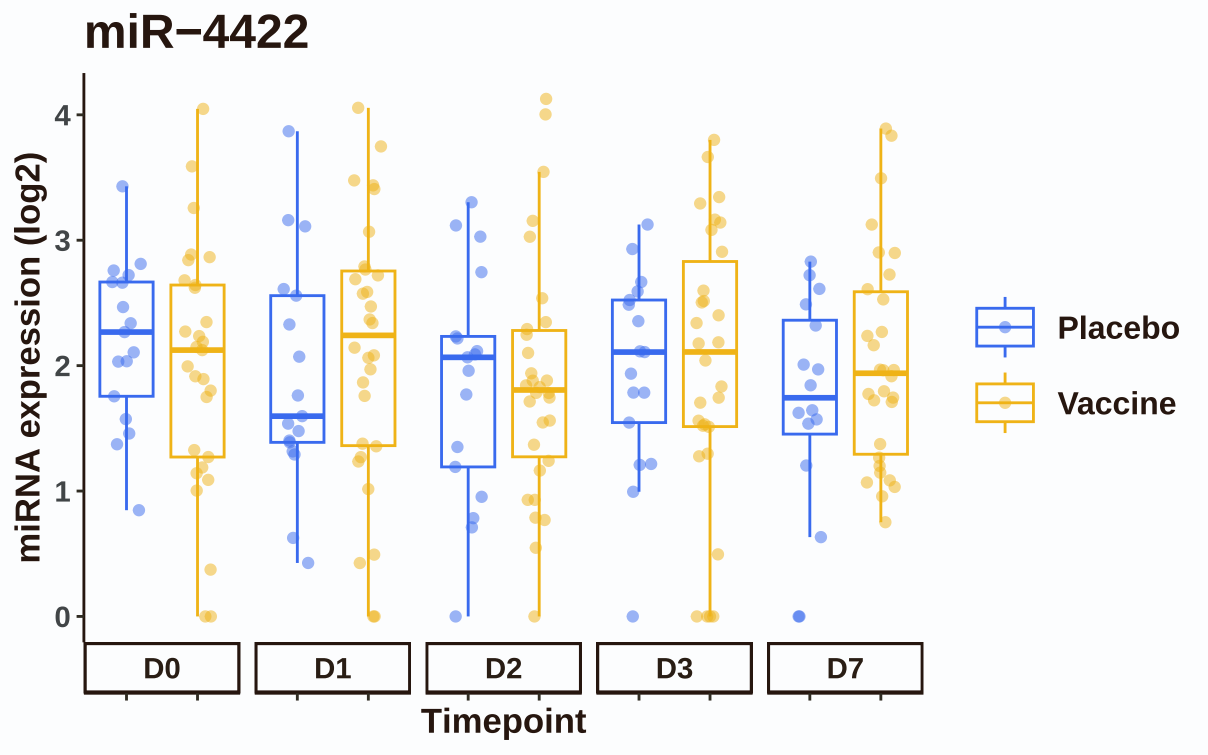

miR−4422
miRNA expression (log2)
0
1
2
3
4
D0
D1
D2
D3
D7
Timepoint
Placebo
Vaccine

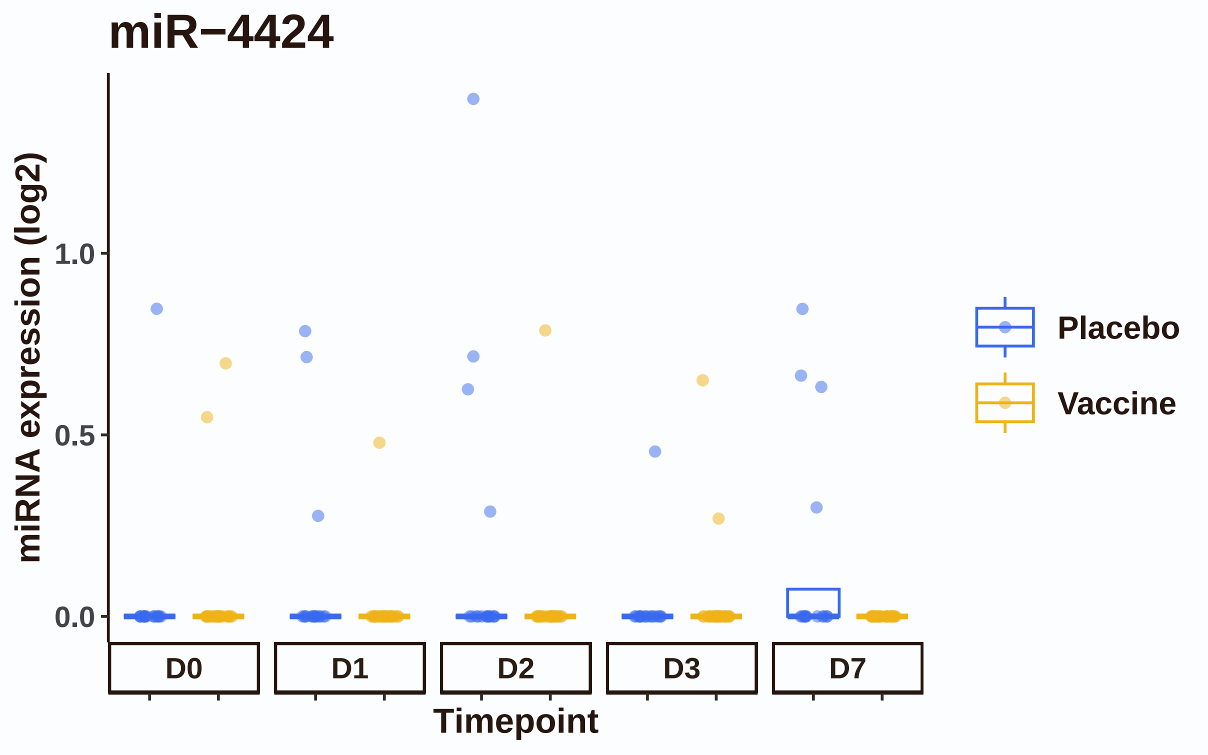

miR−4424
miRNA expression (log2)
1.0
0.5
0.0
D0
D1
D2
D3
D7
Timepoint
Placebo
Vaccine

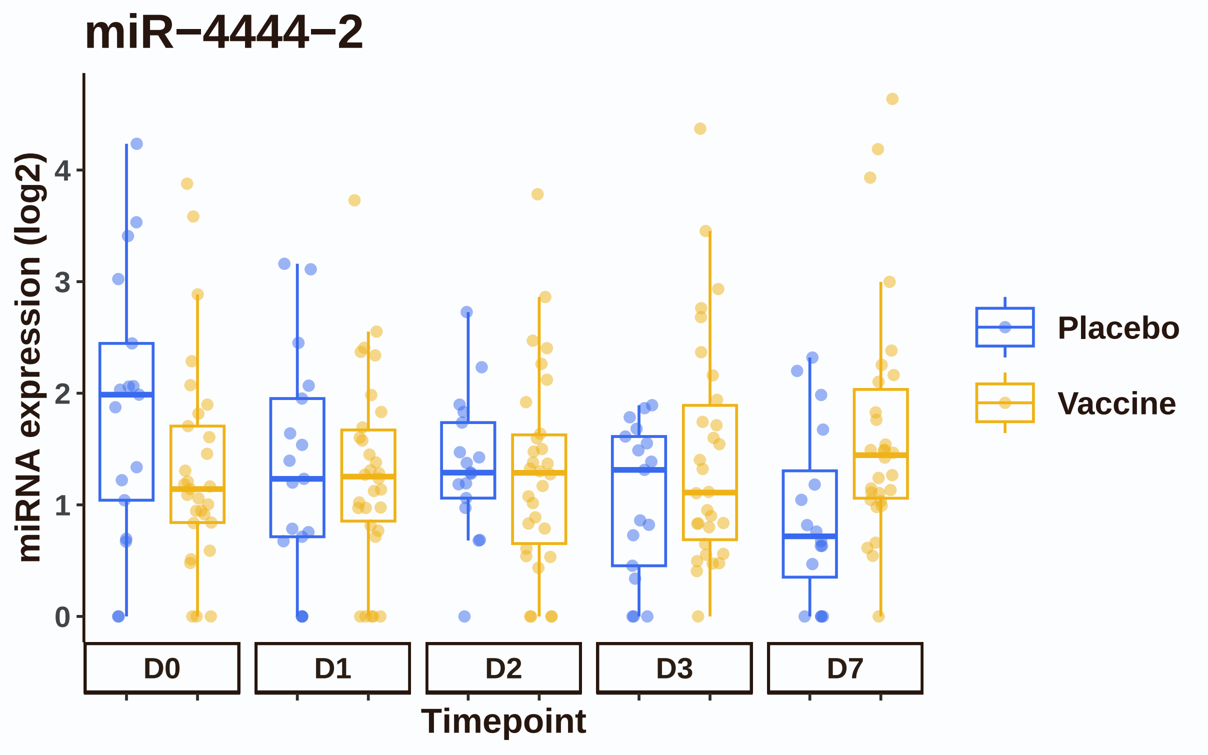

miR−4444−2
miRNA expression (log2)
0
1
2
3
4
D0
D1
D2
D3
D7
Timepoint
Placebo
Vaccine

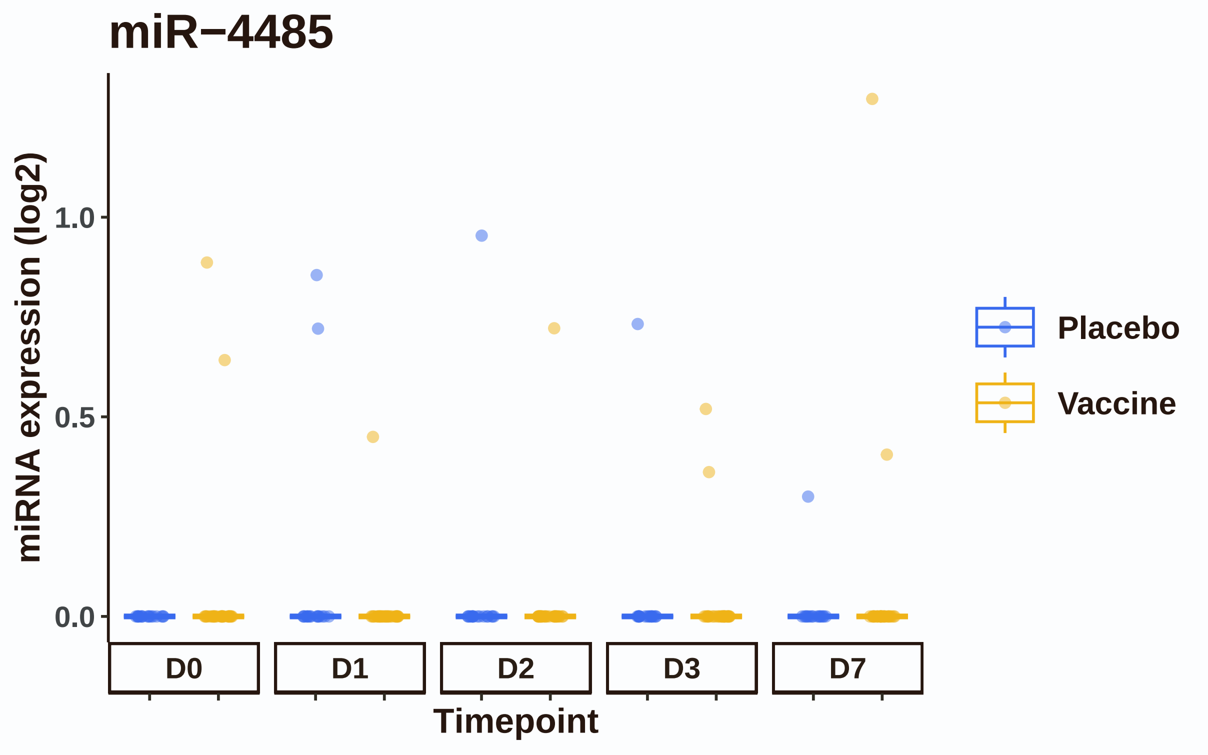
miR−4485
miRNA expression (log2)
1.0
0.5
0.0
D0
D1
D2
D3
D7
Timepoint
Placebo
Vaccine

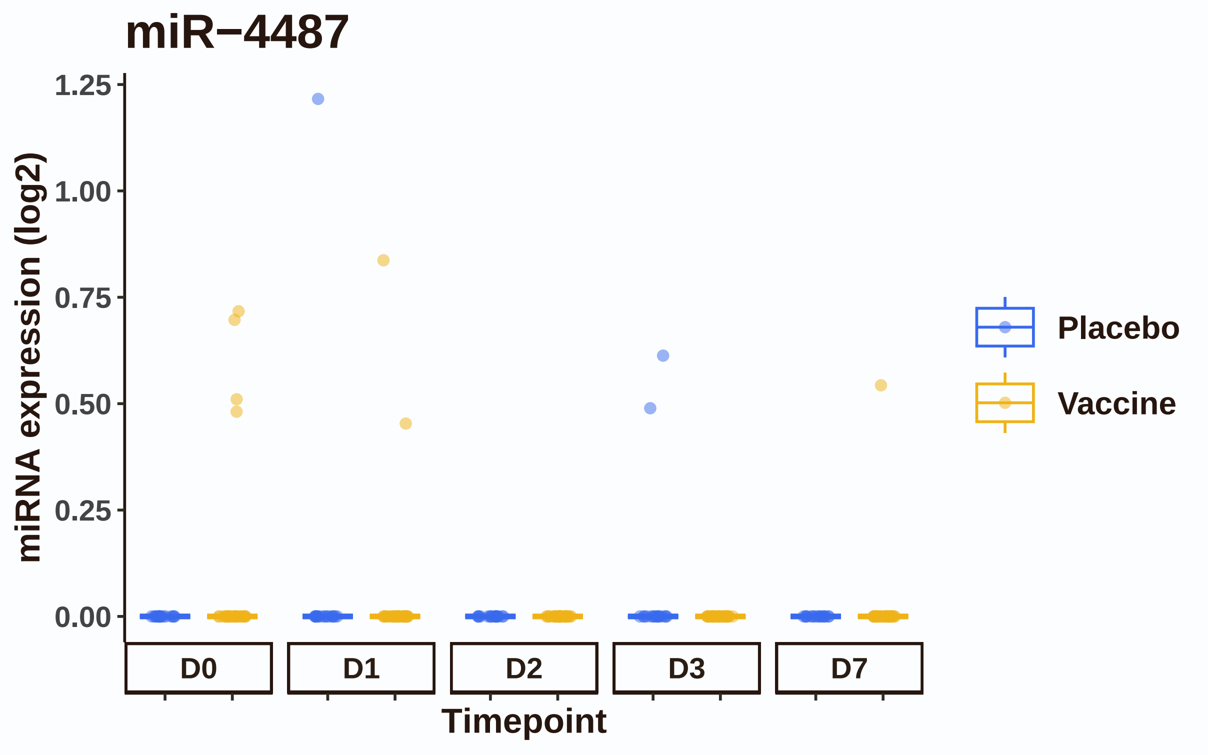

miR−4487
miRNA expression (log2)
1.25
1.00
0.75
0.50
0.25
0.00
D0
D1
D2
D3
D7
Timepoint
Placebo
Vaccine

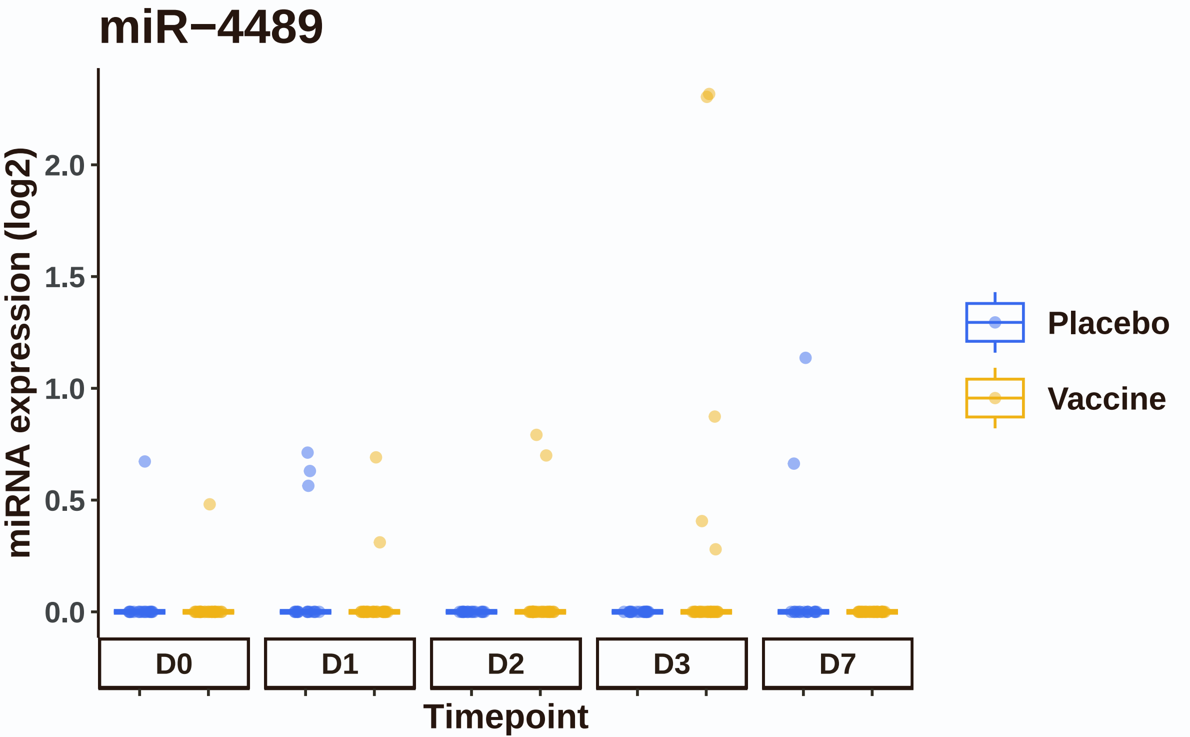

miR−4489
miRNA expression (log2)
2.0
1.5
1.0
0.5
0.0
D0
D1
D2
D3
D7
Timepoint
Placebo
Vaccine

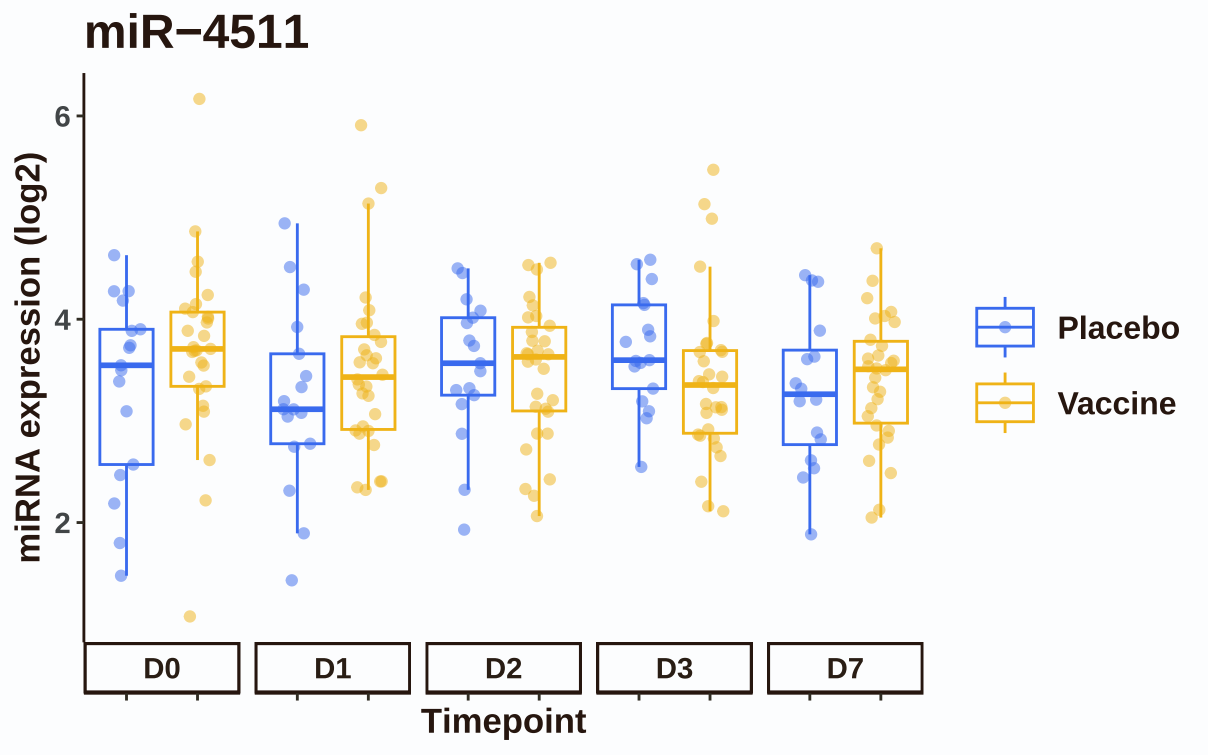

miR−4511
miRNA expression (log2)
6
4
2
D0
D1
D2
D3
D7
Timepoint
Placebo
Vaccine

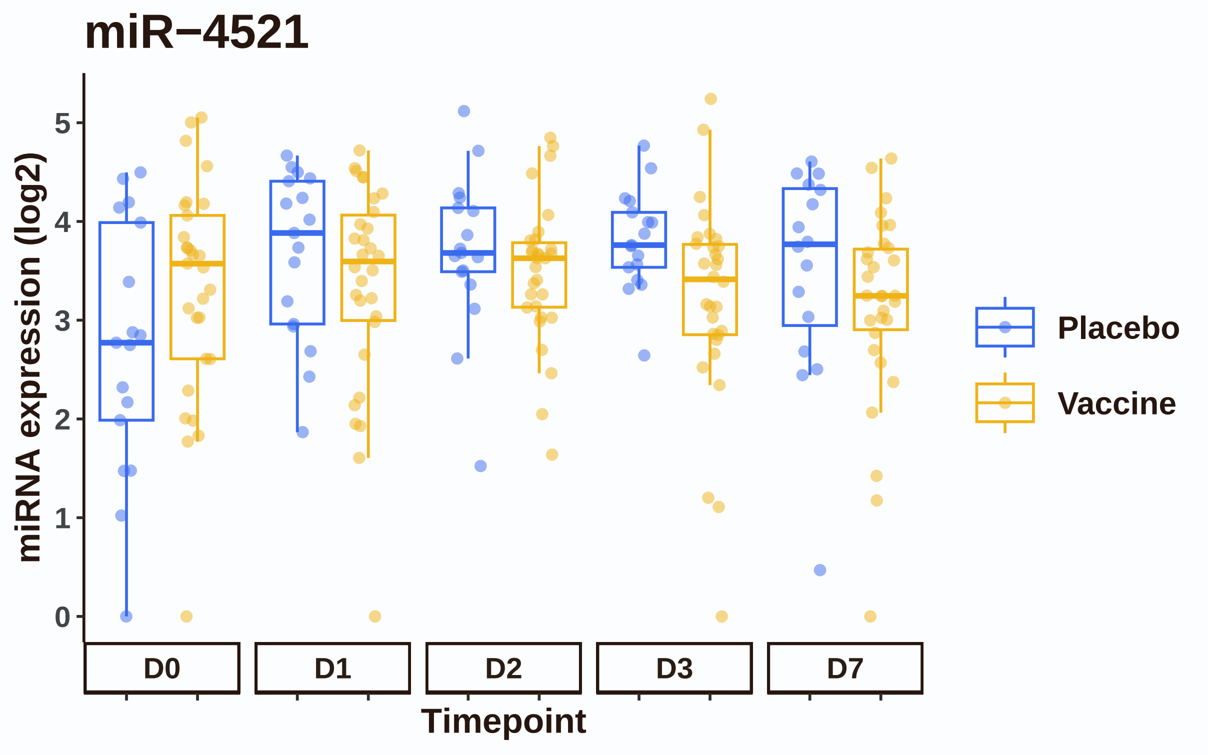
miR−4521
miRNA expression (log2)
0
1
2
3
4
5
D0
D1
D2
D3
D7
Timepoint
Placebo
Vaccine

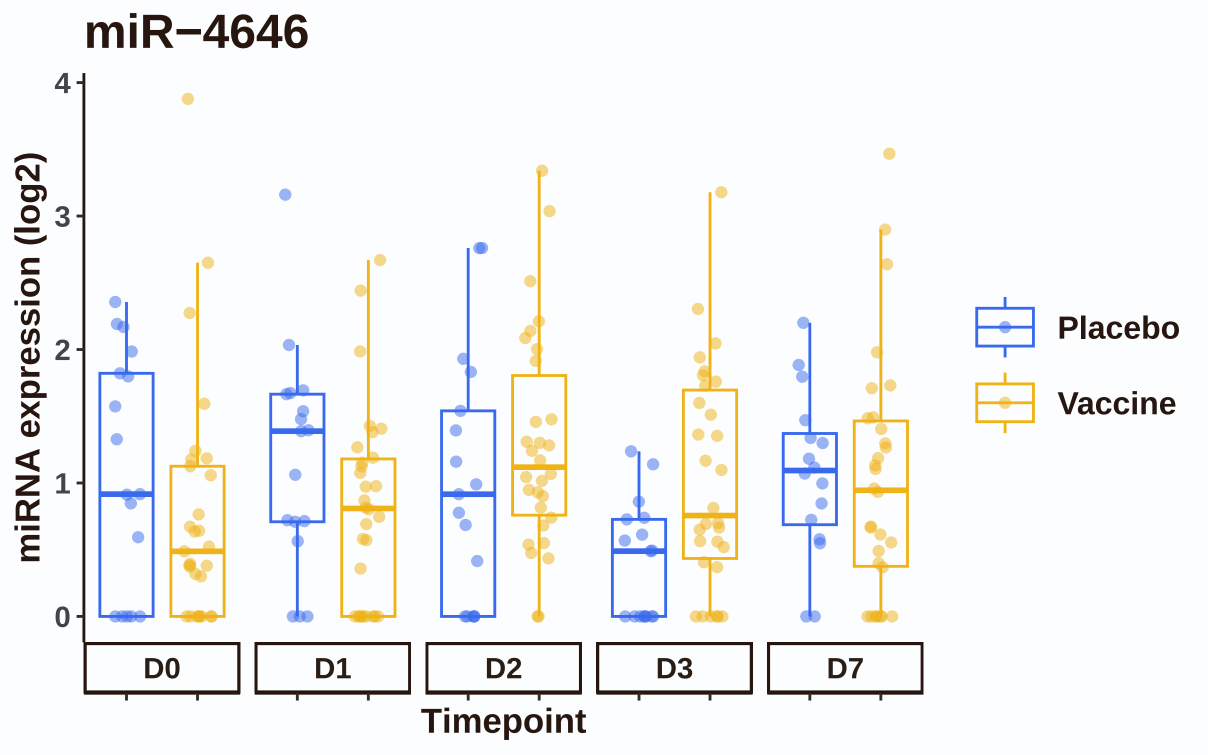

miR−4646
miRNA expression (log2)
4
3
2
1
0
D0
D1
D2
D3
D7
Timepoint
Placebo
Vaccine

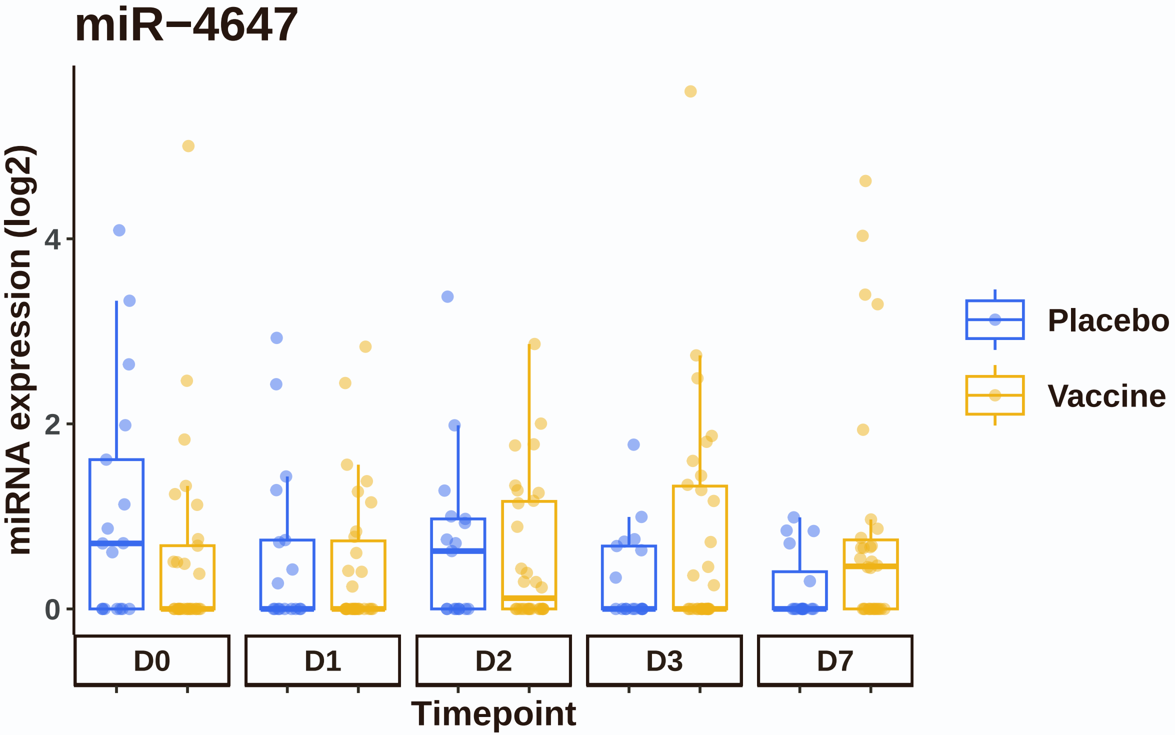

miR−4647
miRNA expression (log2)
0
2
4
D0
D1
D2
D3
D7
Timepoint
Placebo
Vaccine

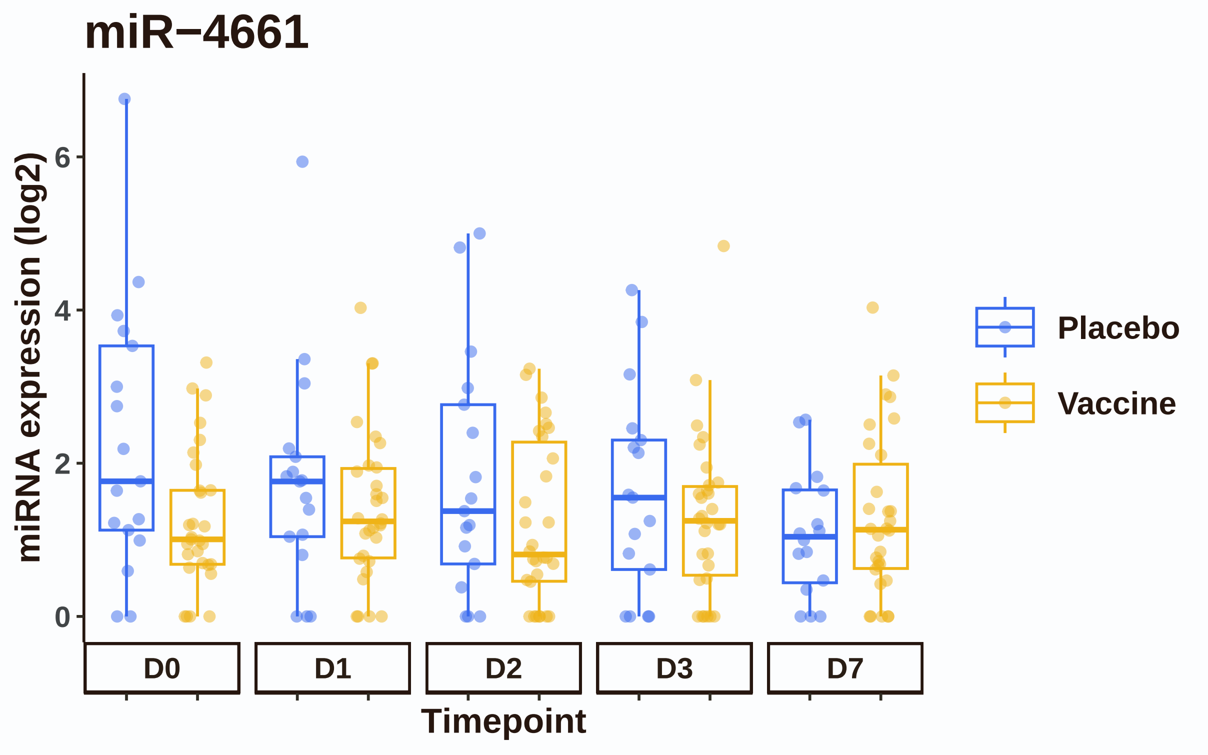
miR−4661
miRNA expression (log2)
0
2
4
6
D0
D1
D2
D3
D7
Timepoint
Placebo
Vaccine

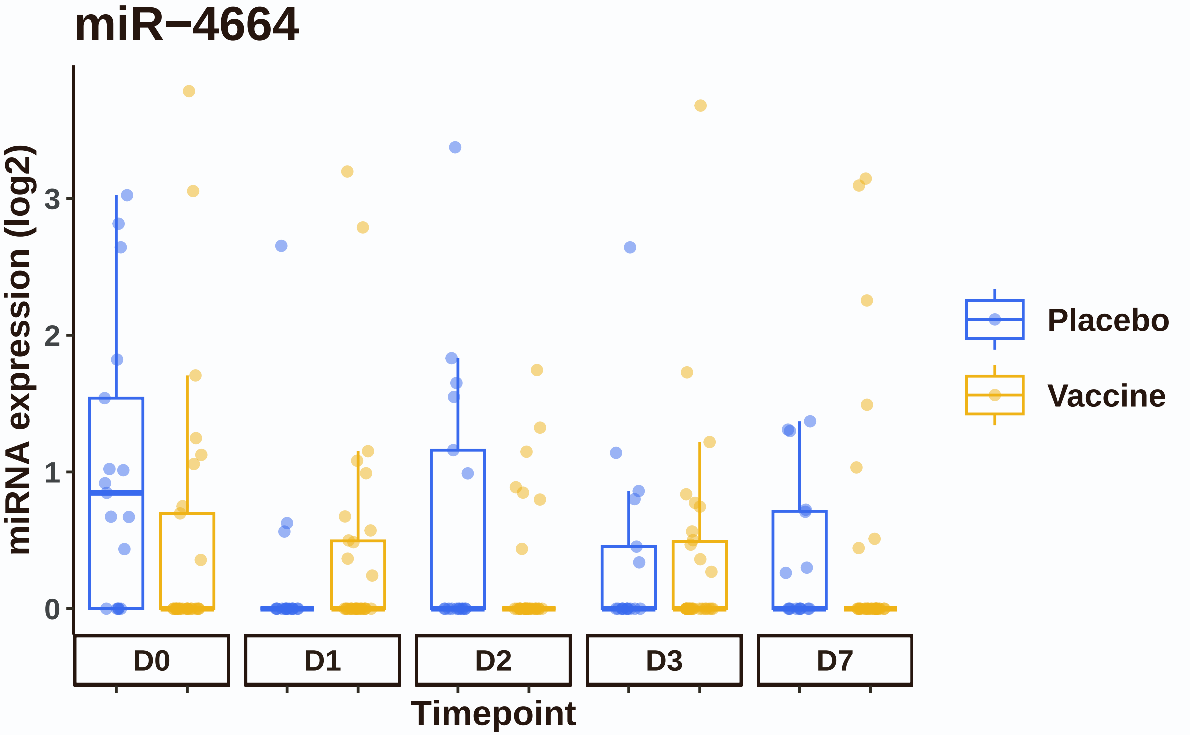

miR−4664
miRNA expression (log2)
0
1
2
3
D0
D1
D2
D3
D7
Timepoint
Placebo
Vaccine

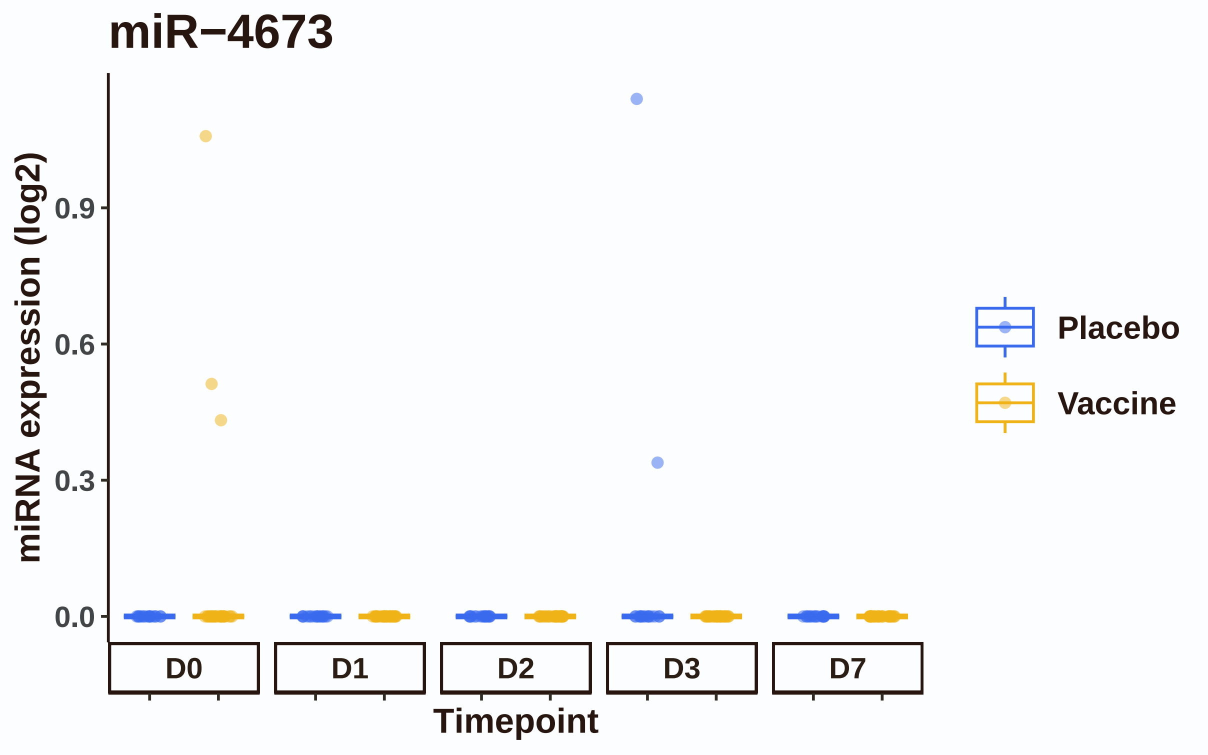
miR−4673
miRNA expression (log2)
0.9
0.6
0.3
0.0
D0
D1
D2
D3
D7
Timepoint
Placebo
Vaccine

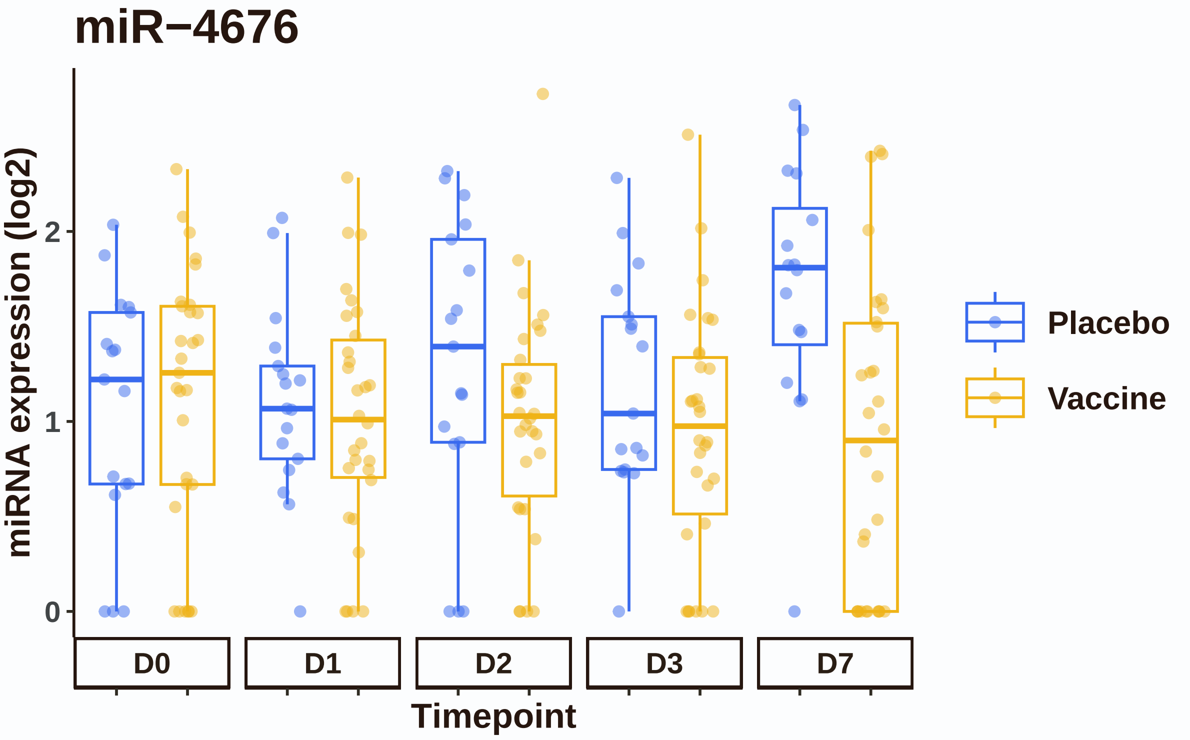
miR−4676
miRNA expression (log2)
0
1
2
D0
D1
D2
D3
D7
Timepoint
Placebo
Vaccine

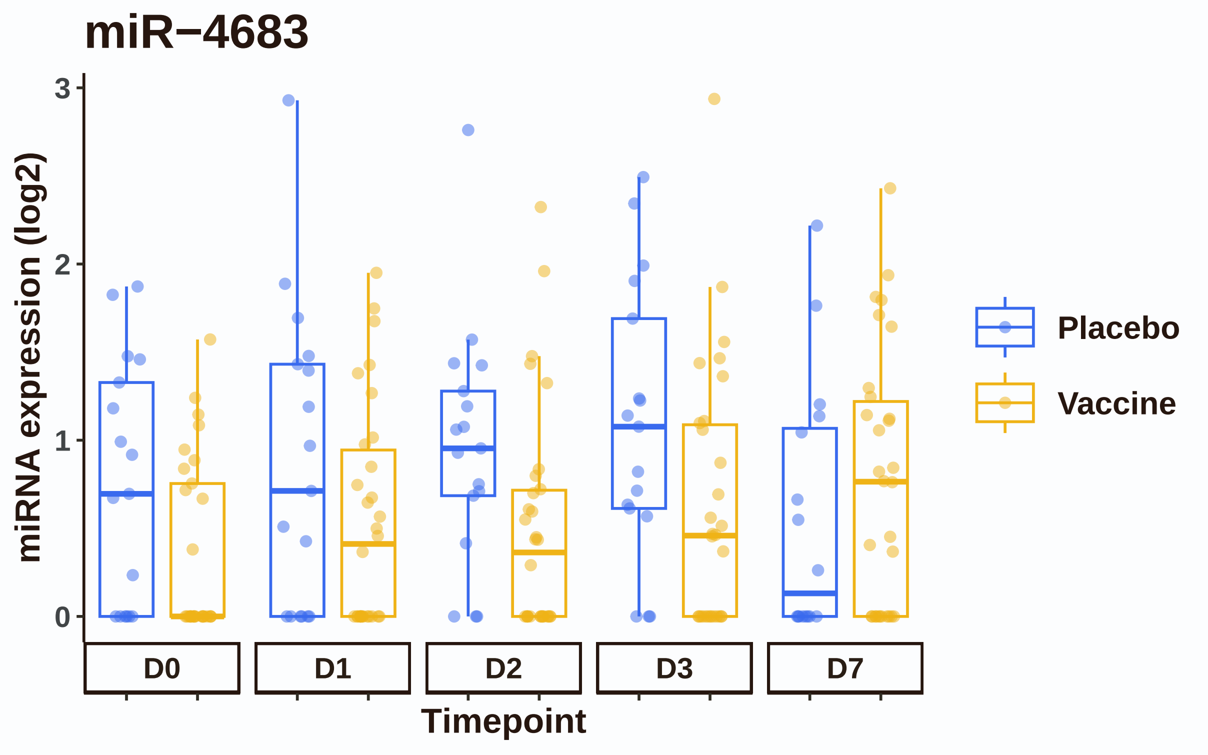
miR−4683
miRNA expression (log2)
3
2
1
0
D0
D1
D2
D3
D7
Timepoint
Placebo
Vaccine

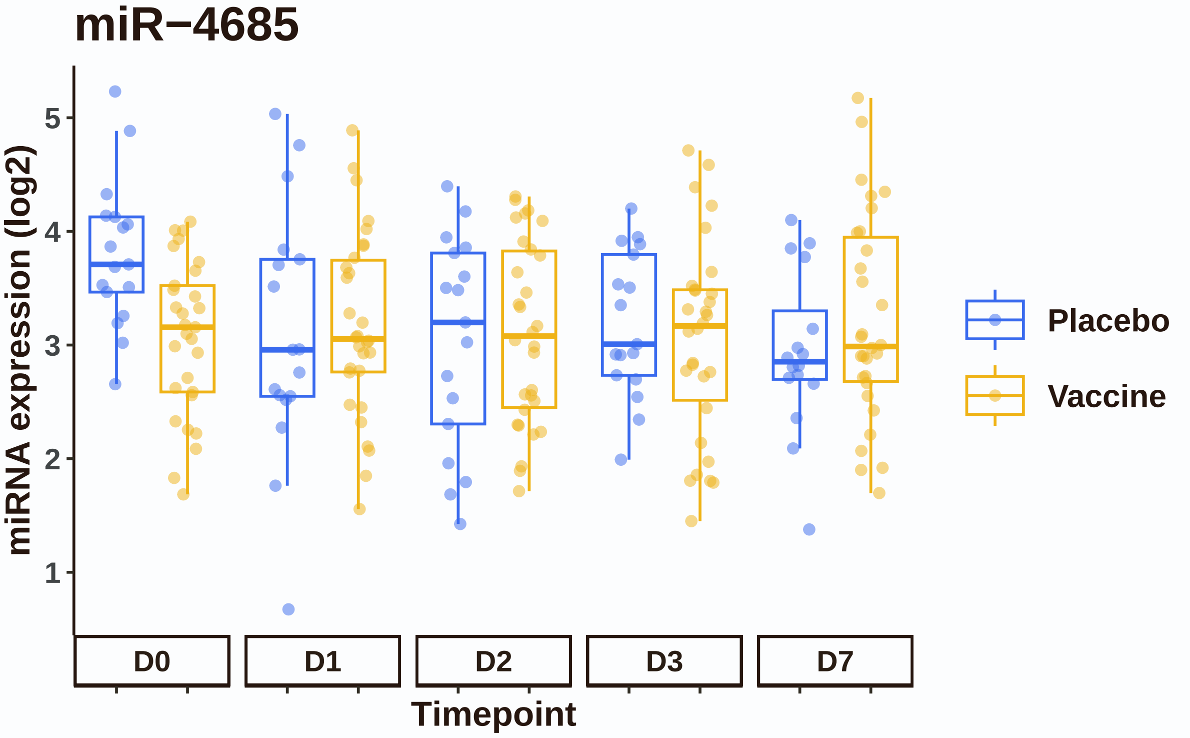
miR−4685
miRNA expression (log2)
5
4
3
2
1
D0
D1
D2
D3
D7
Timepoint
Placebo
Vaccine

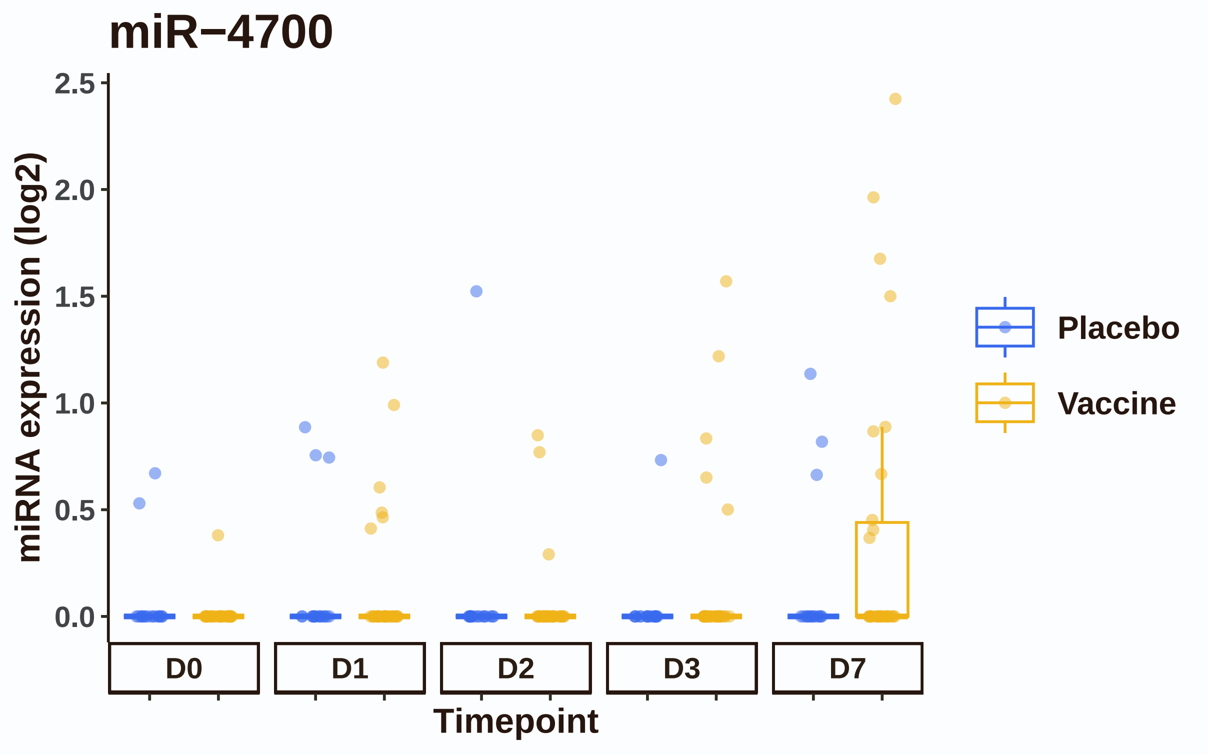
miR−4700
miRNA expression (log2)
2.5
2.0
1.5
1.0
0.5
0.0
D0
D1
D2
D3
D7
Timepoint
Placebo
Vaccine

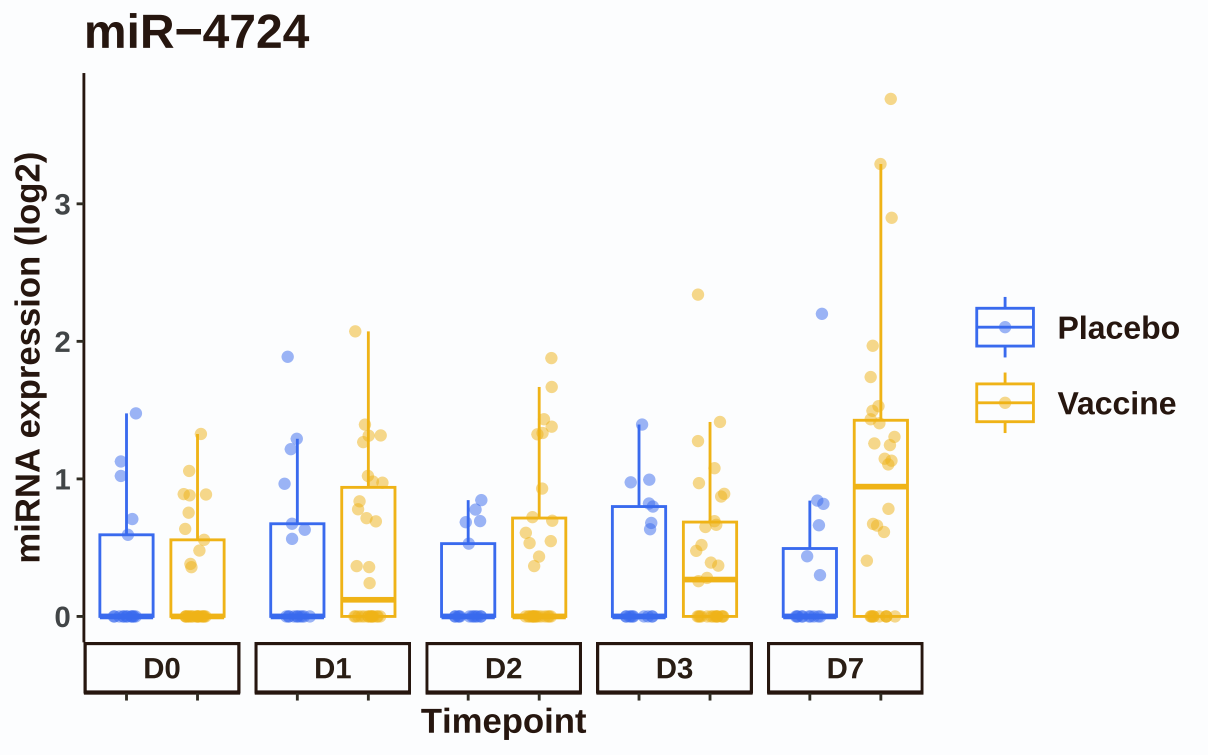

miR−4724
miRNA expression (log2)
0
1
2
3
D0
D1
D2
D3
D7
Timepoint
Placebo
Vaccine

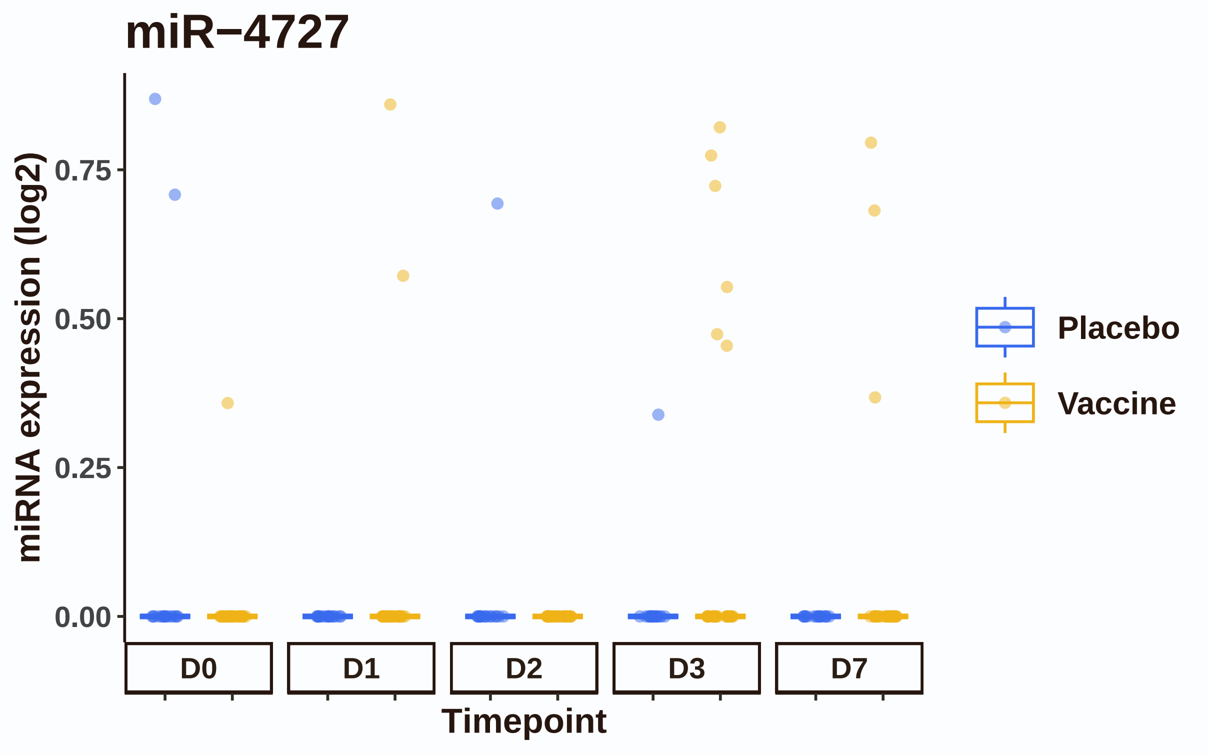
miR−4727
miRNA expression (log2)
0.75
0.50
0.25
0.00
D0
D1
D2
D3
D7
Timepoint
Placebo
Vaccine

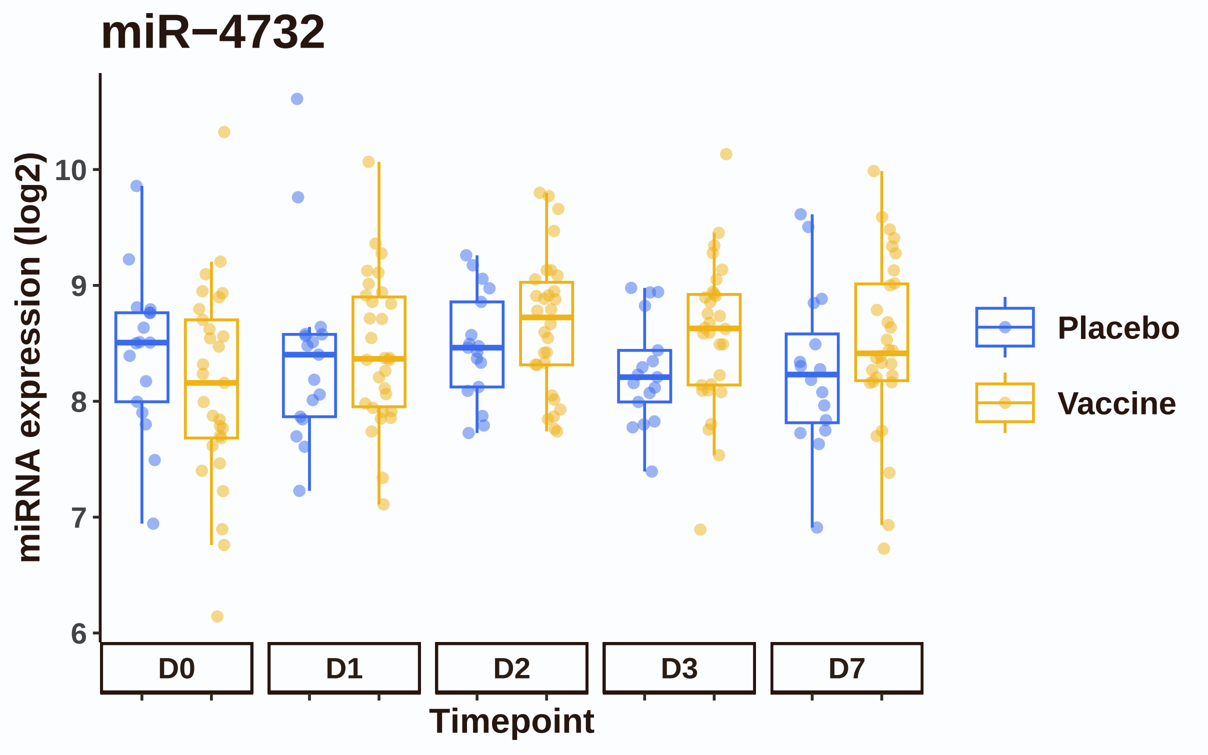
miR−4732
miRNA expression (log2)
10
9
8
7
6
D0
D1
D2
D3
D7
Timepoint
Placebo
Vaccine

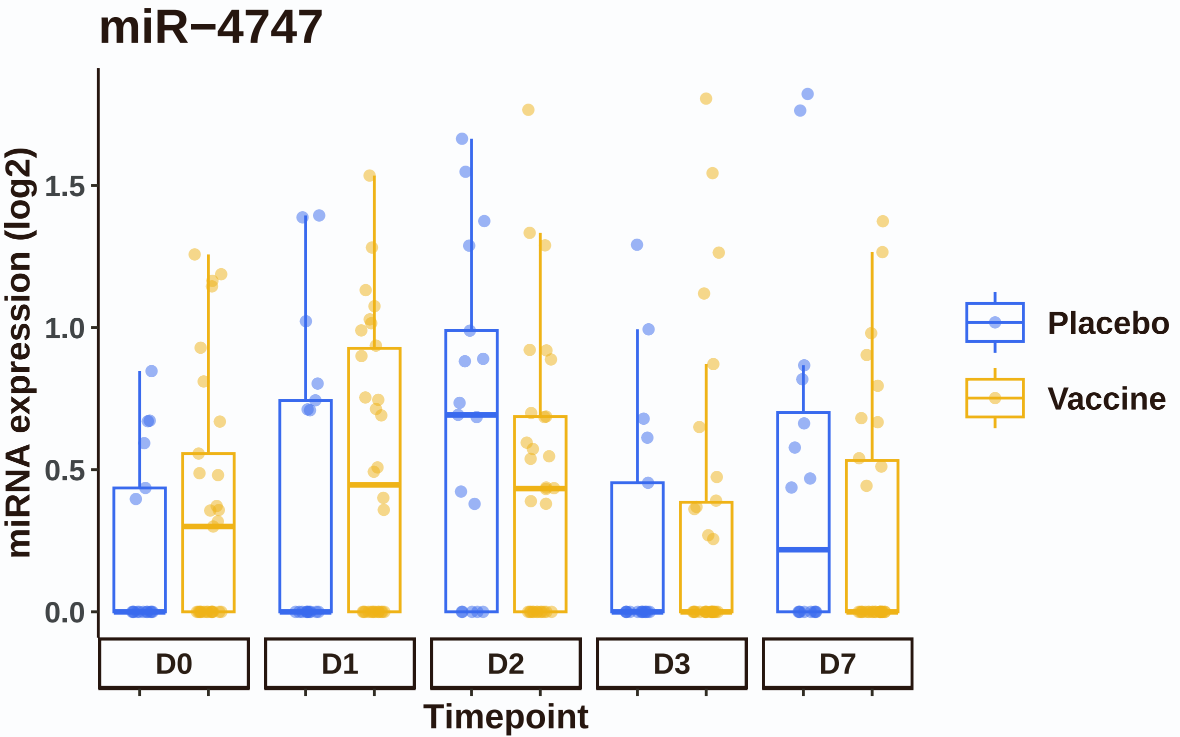
miR−4747
miRNA expression (log2)
1.5
1.0
0.5
0.0
D0
D1
D2
D3
D7
Timepoint
Placebo
Vaccine

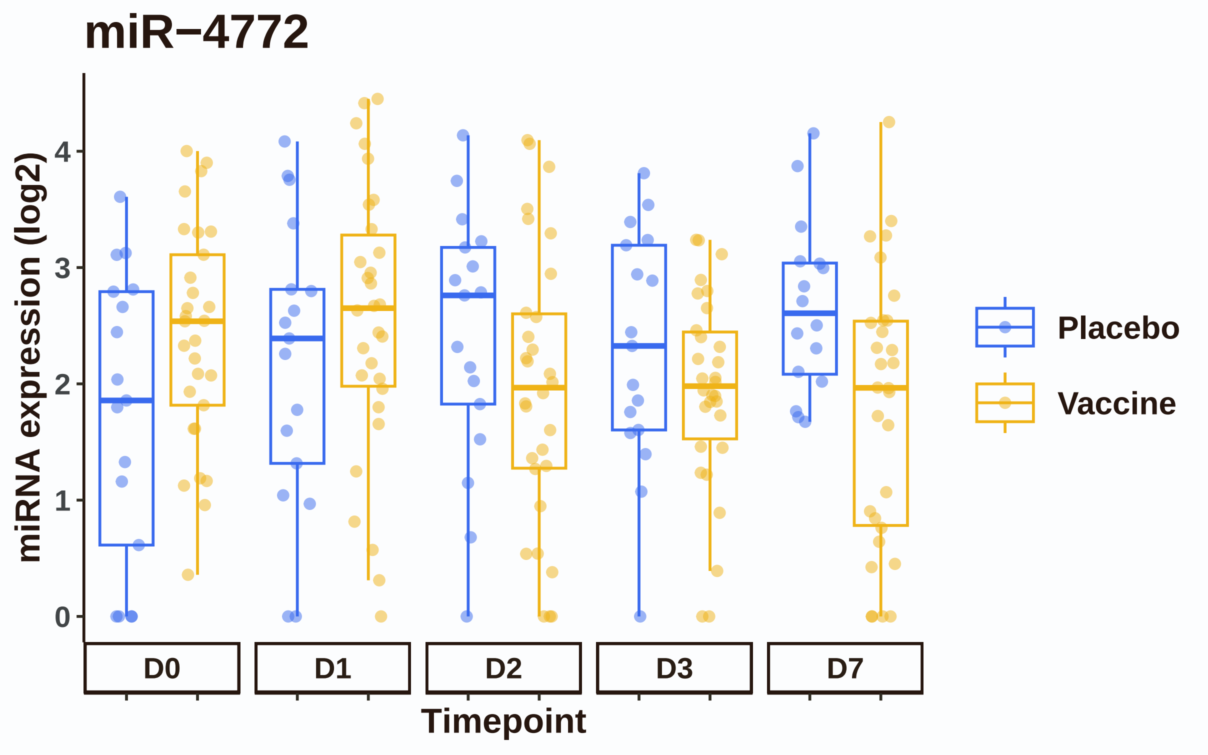
miR−4772
miRNA expression (log2)
0
1
2
3
4
D0
D1
D2
D3
D7
Timepoint
Placebo
Vaccine

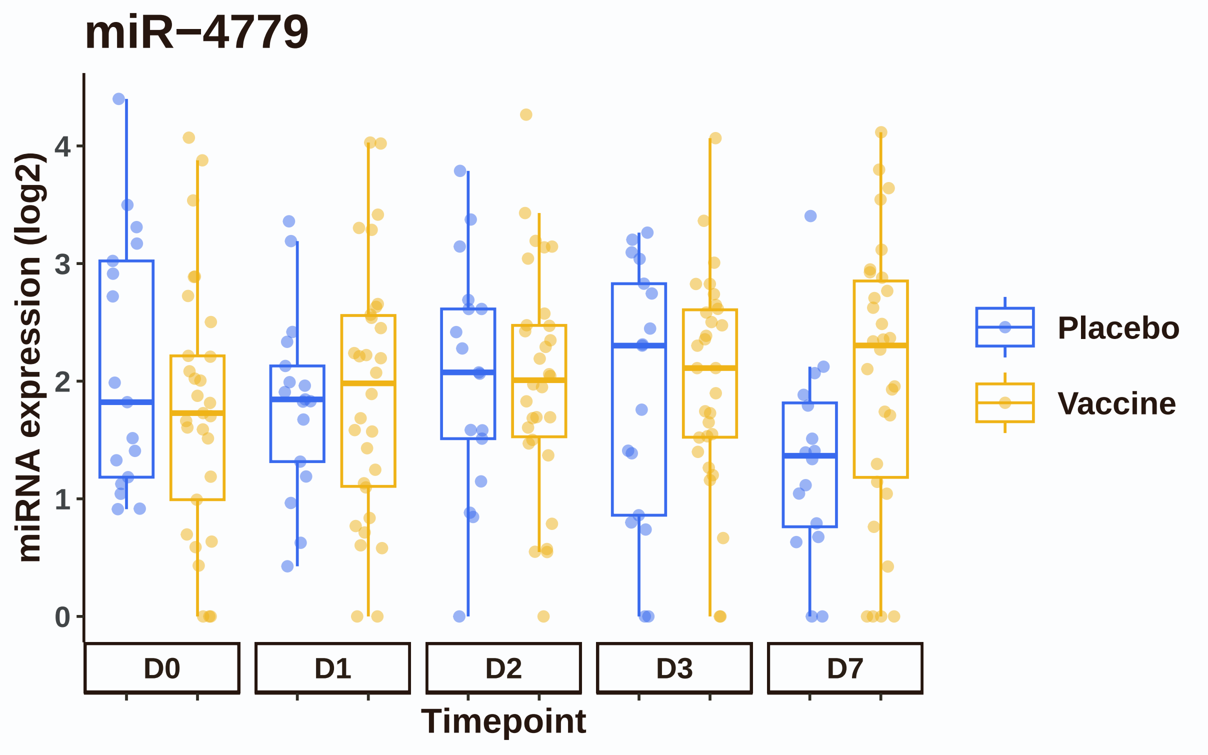

miR−4779
miRNA expression (log2)
0
1
2
3
4
D0
D1
D2
D3
D7
Timepoint
Placebo
Vaccine

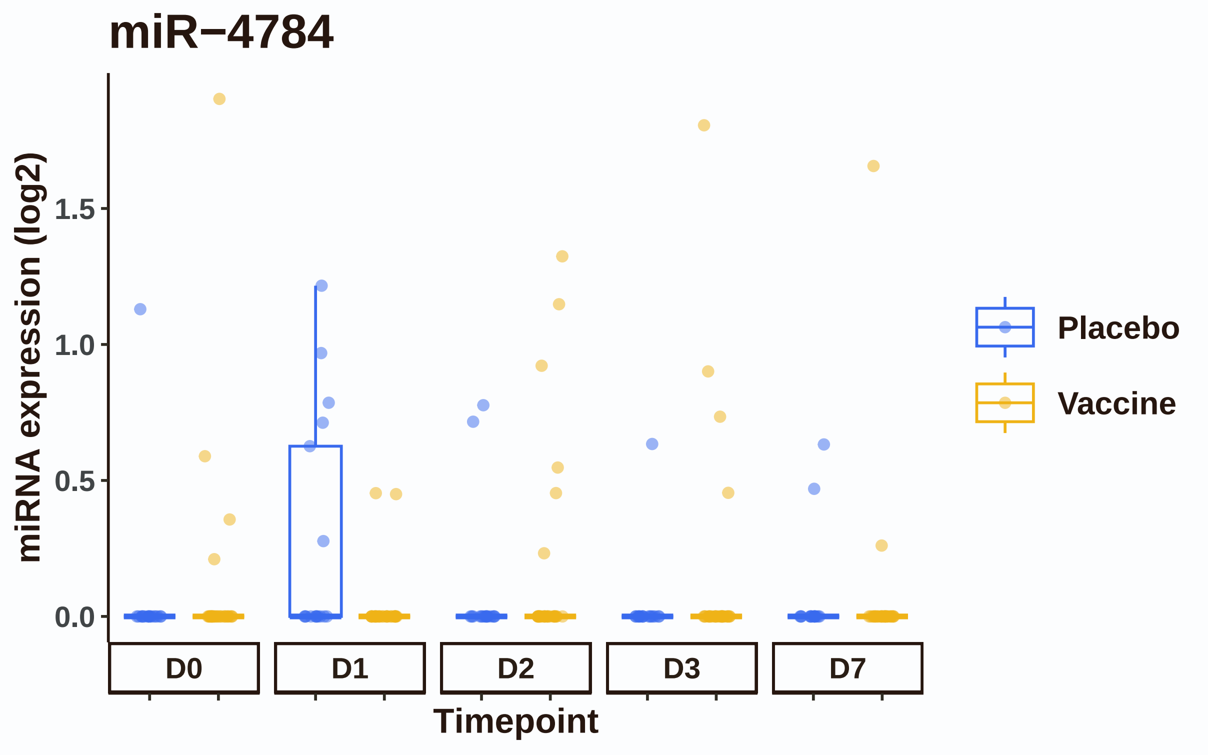

miR−4784
miRNA expression (log2)
1.5
1.0
0.5
0.0
D0
D1
D2
D3
D7
Timepoint
Placebo
Vaccine

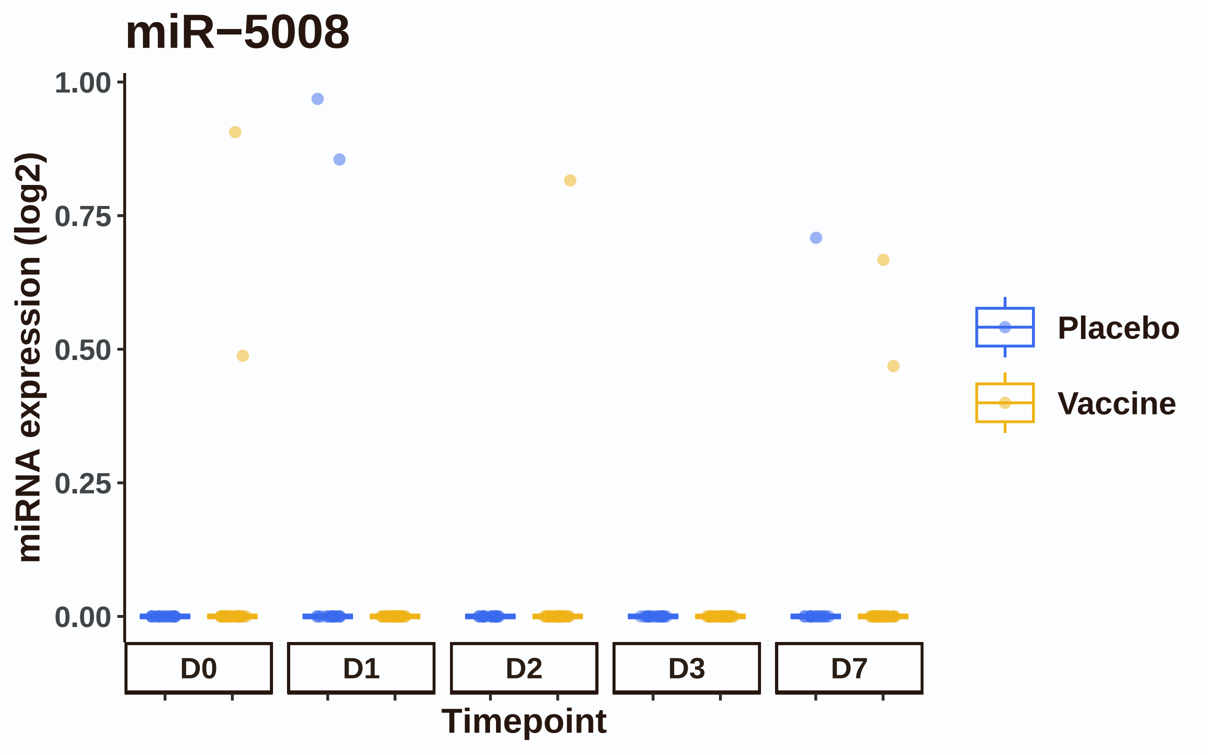

miR−5008
miRNA expression (log2)
1.00
0.75
0.50
0.25
0.00
D0
D1
D2
D3
D7
Timepoint
Placebo
Vaccine

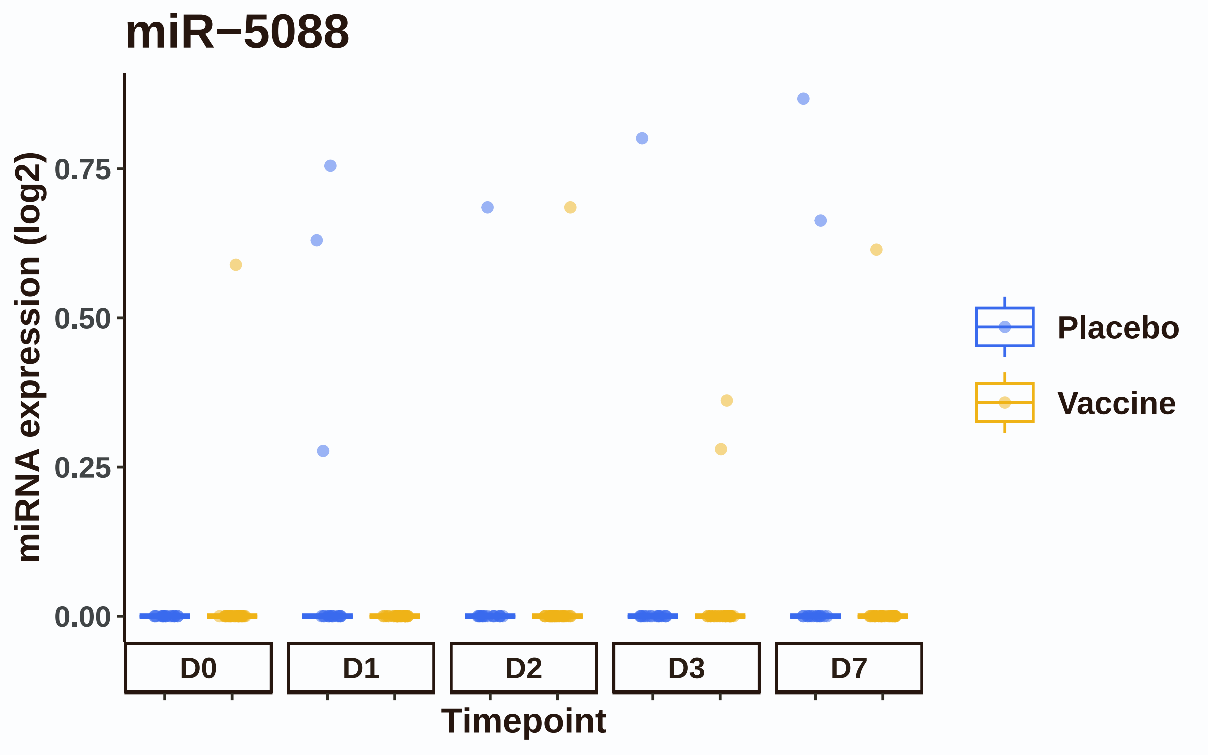

miR−5088
miRNA expression (log2)
0.00
0.25
0.50
0.75
D0
D1
D2
D3
D7
Timepoint
Placebo
Vaccine

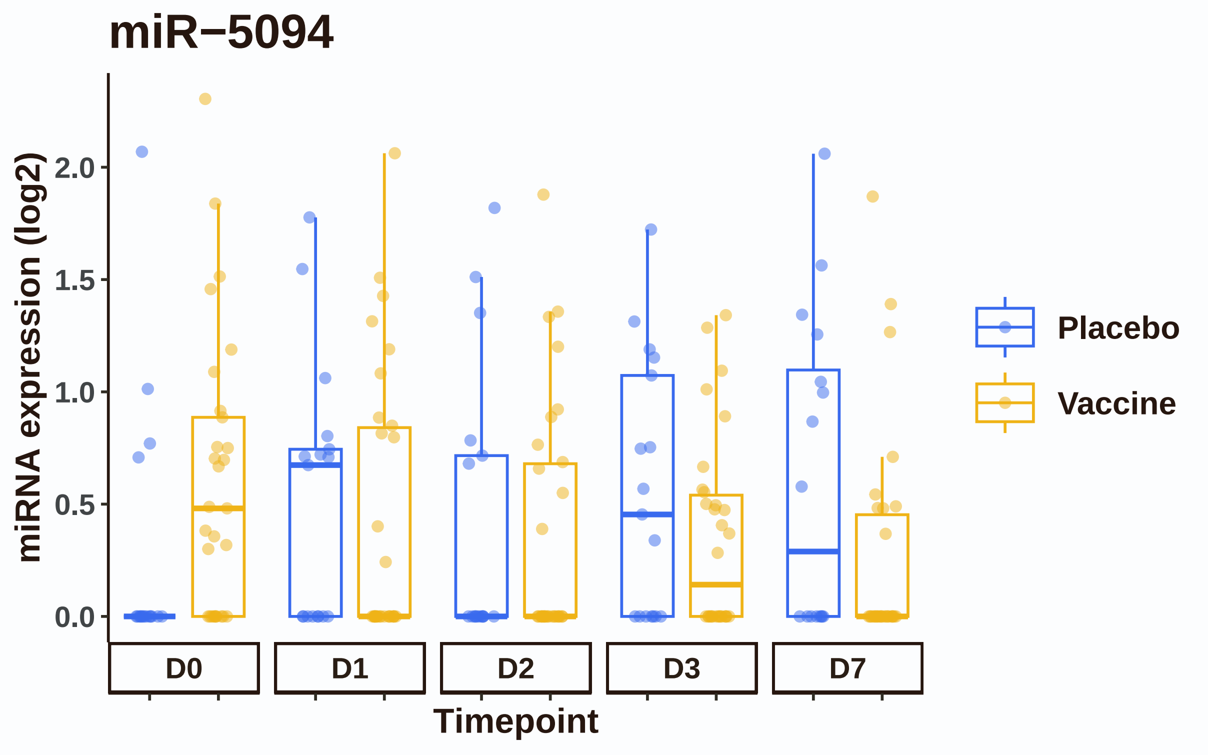
miR−5094
miRNA expression (log2)
2.0
1.5
1.0
0.5
0.0
D0
D1
D2
D3
D7
Timepoint
Placebo
Vaccine

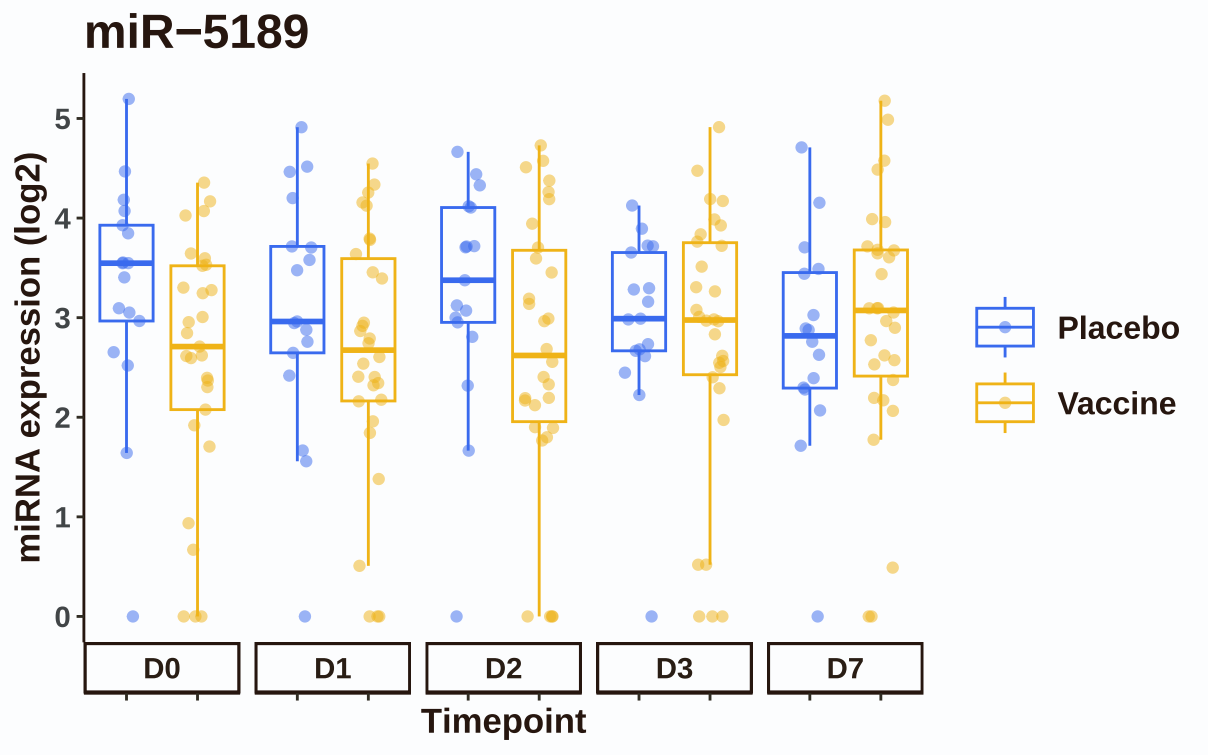

miR−5189
miRNA expression (log2)
0
1
2
3
4
5
D0
D1
D2
D3
D7
Timepoint
Placebo
Vaccine

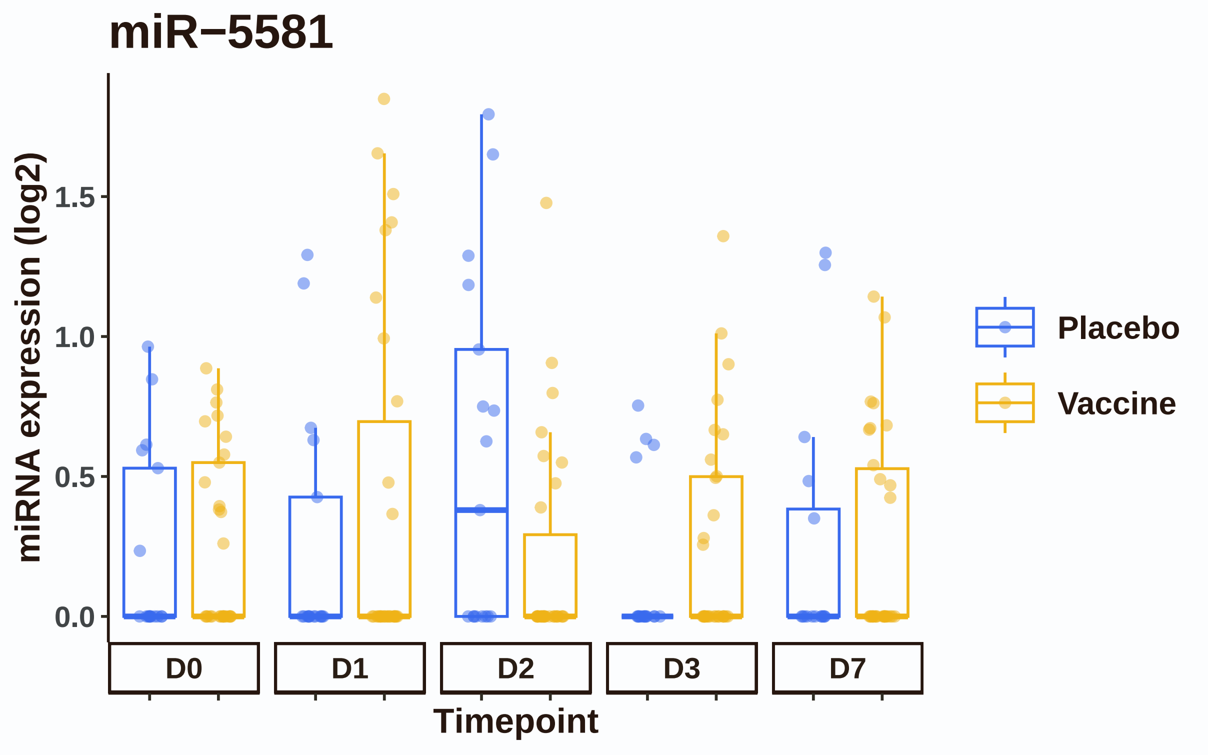

miR−5581
miRNA expression (log2)
0.0
0.5
1.0
1.5
D0
D1
D2
D3
D7
Timepoint
Placebo
Vaccine

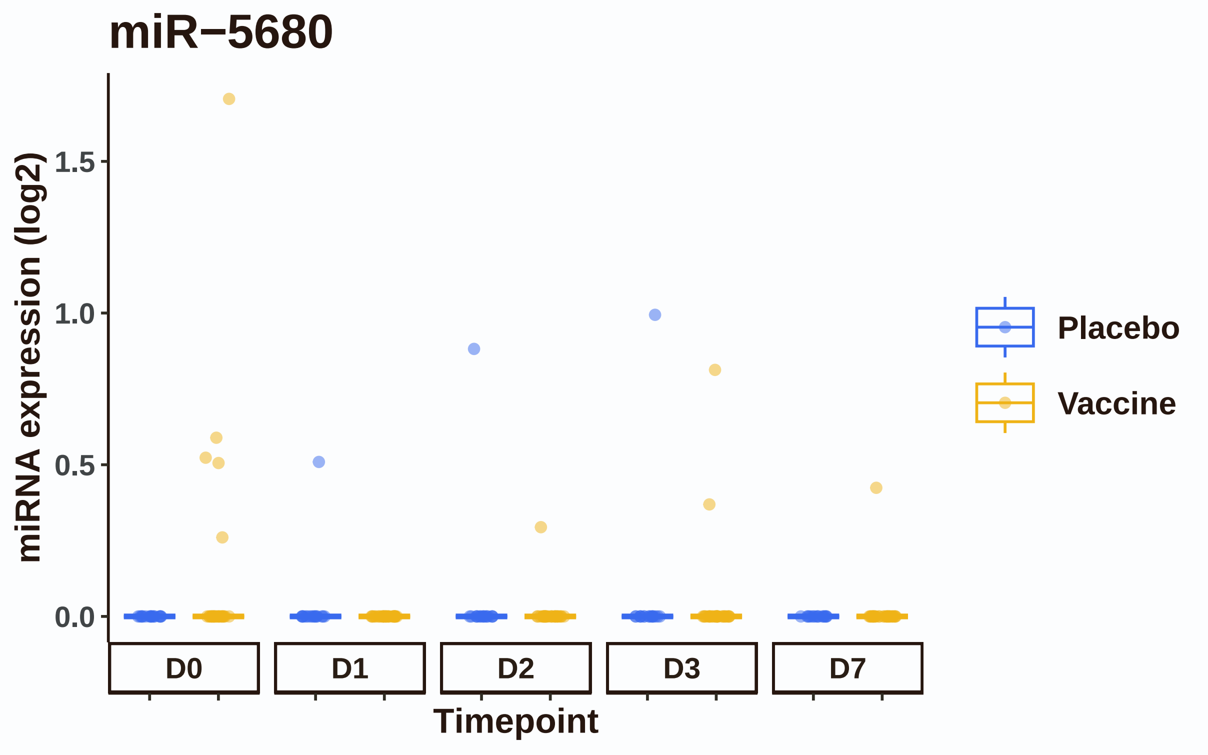
miR−5680
miRNA expression (log2)
1.5
1.0
0.5
0.0
D0
D1
D2
D3
D7
Timepoint
Placebo
Vaccine

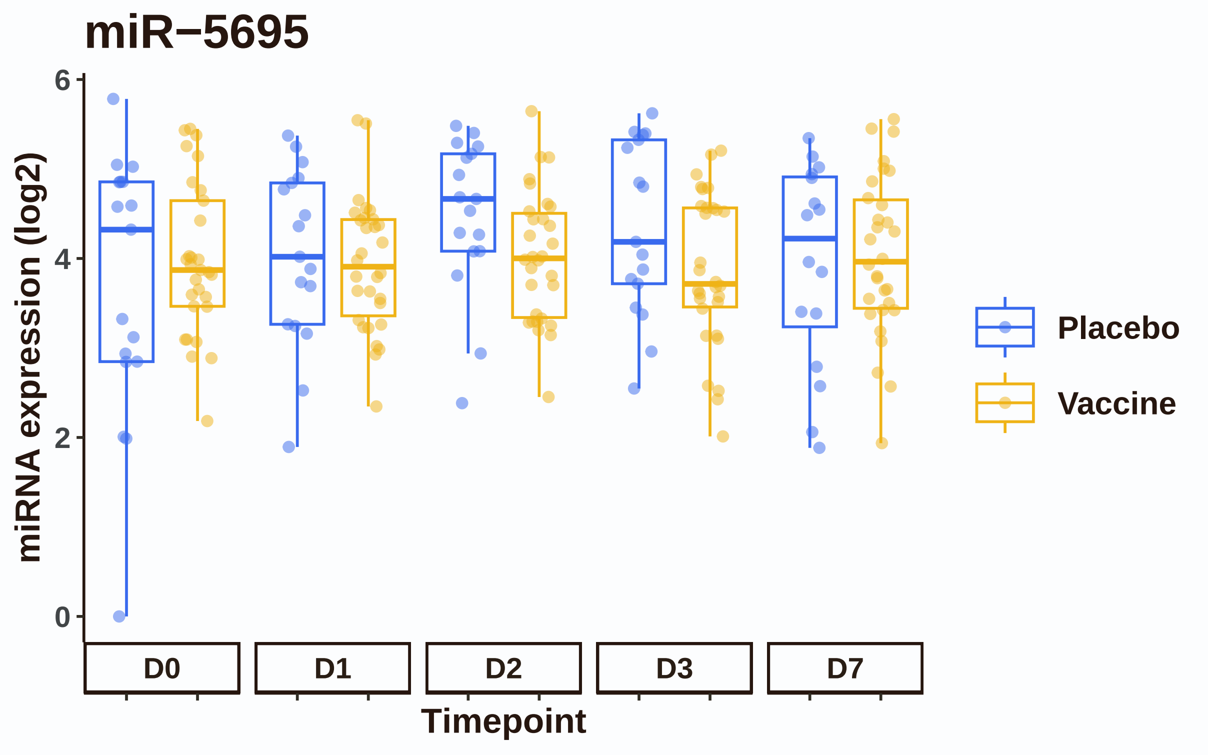
miR−5695
miRNA expression (log2)
0
2
4
6
D0
D1
D2
D3
D7
Timepoint
Placebo
Vaccine

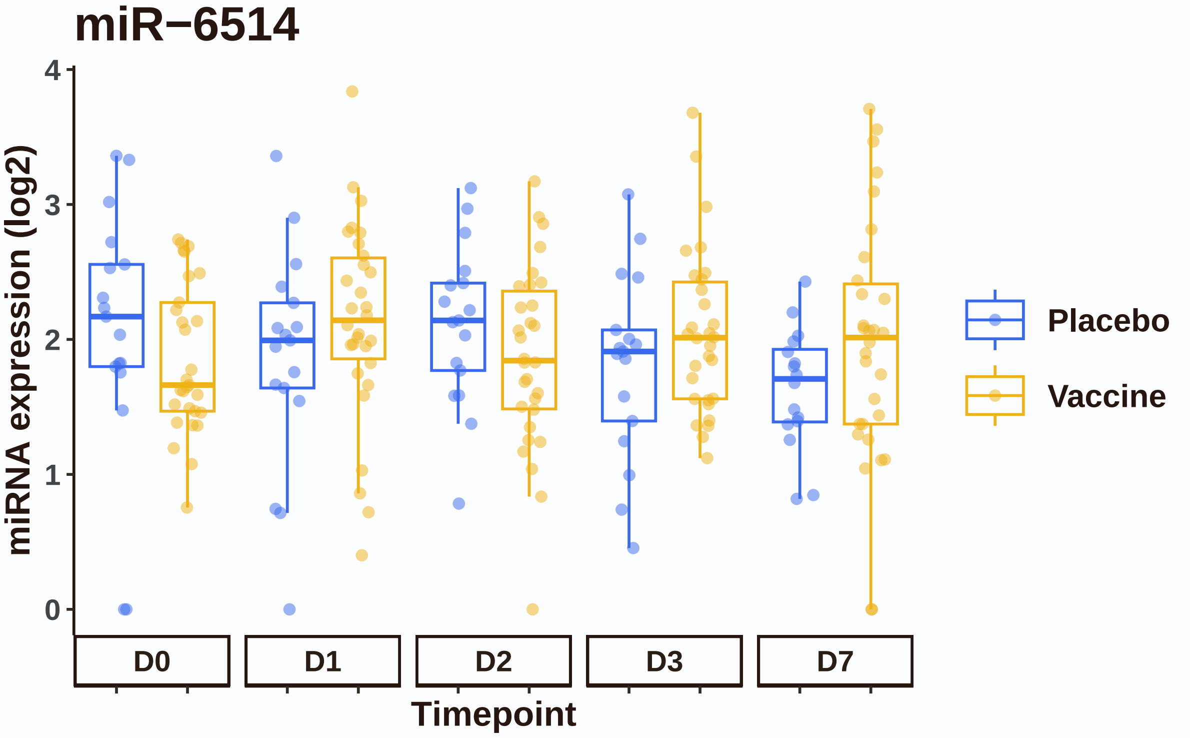
miR−6514
miRNA expression (log2)
4
3
2
1
0
D0
D1
D2
D3
D7
Timepoint
Placebo
Vaccine

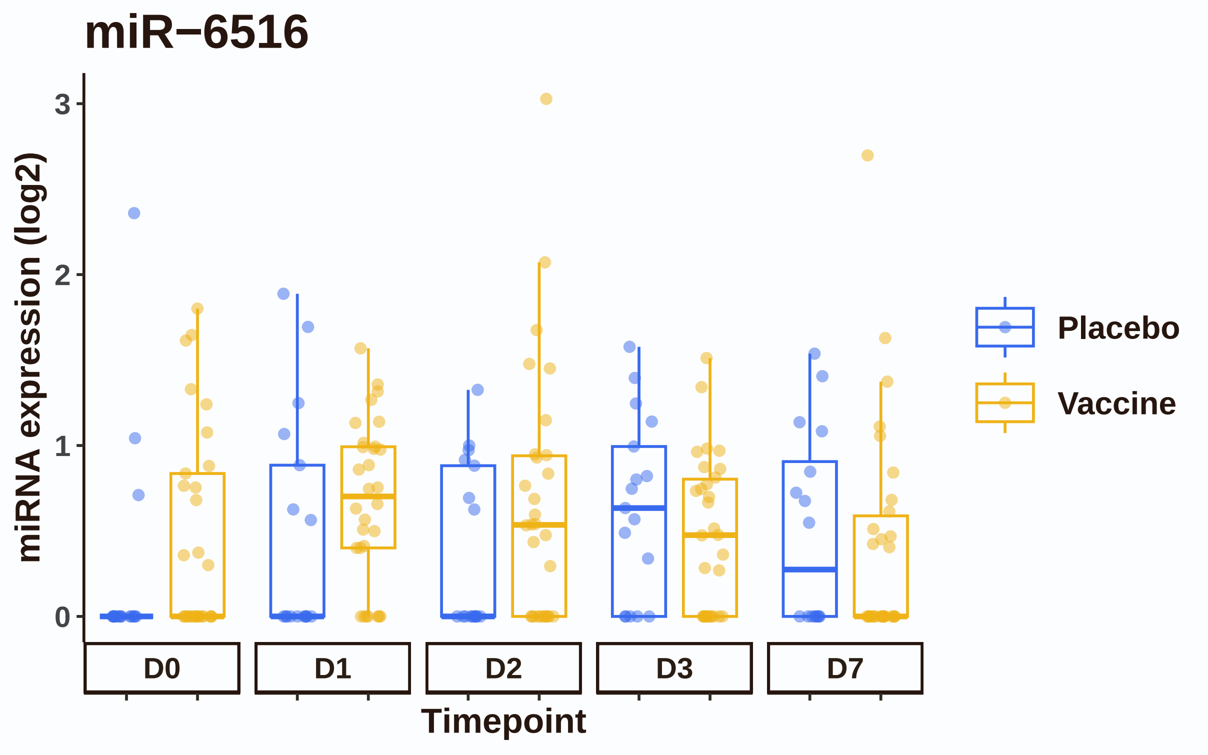

miR−6516
miRNA expression (log2)
3
2
1
0
D0
D1
D2
D3
D7
Timepoint
Placebo
Vaccine

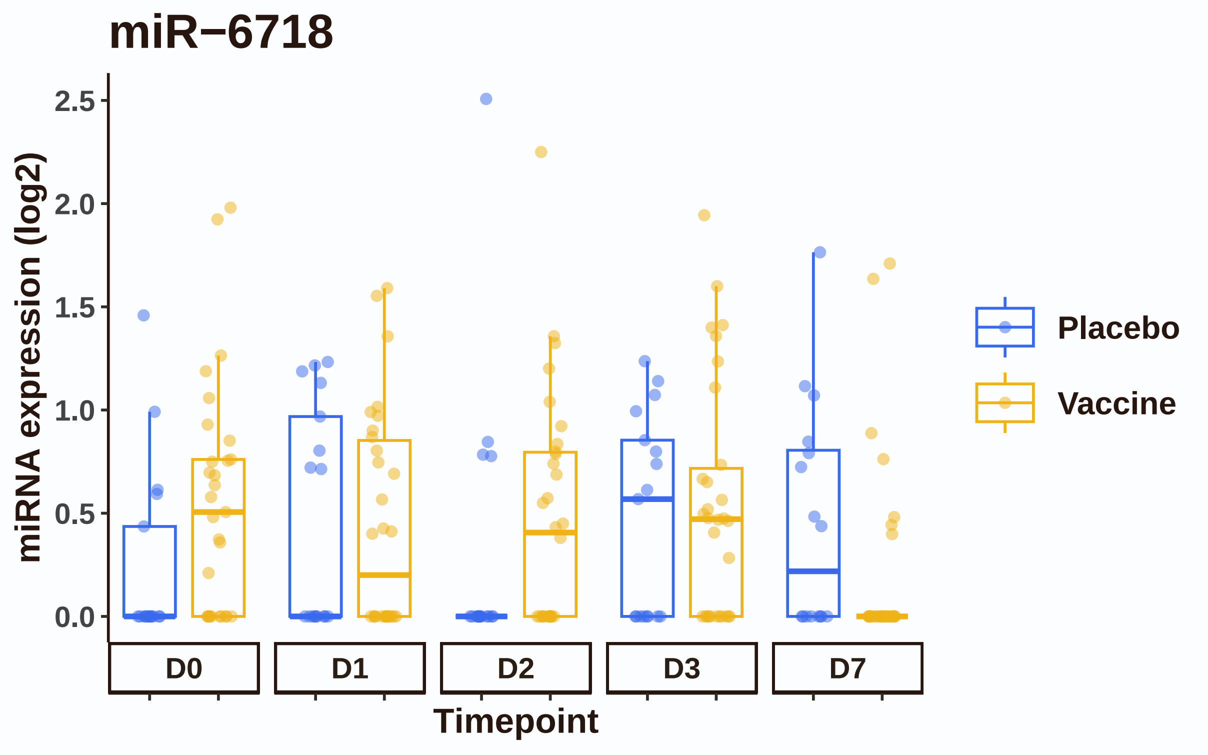

miR−6718
miRNA expression (log2)
2.5
2.0
1.5
1.0
0.5
0.0
D0
D1
D2
D3
D7
Timepoint
Placebo
Vaccine

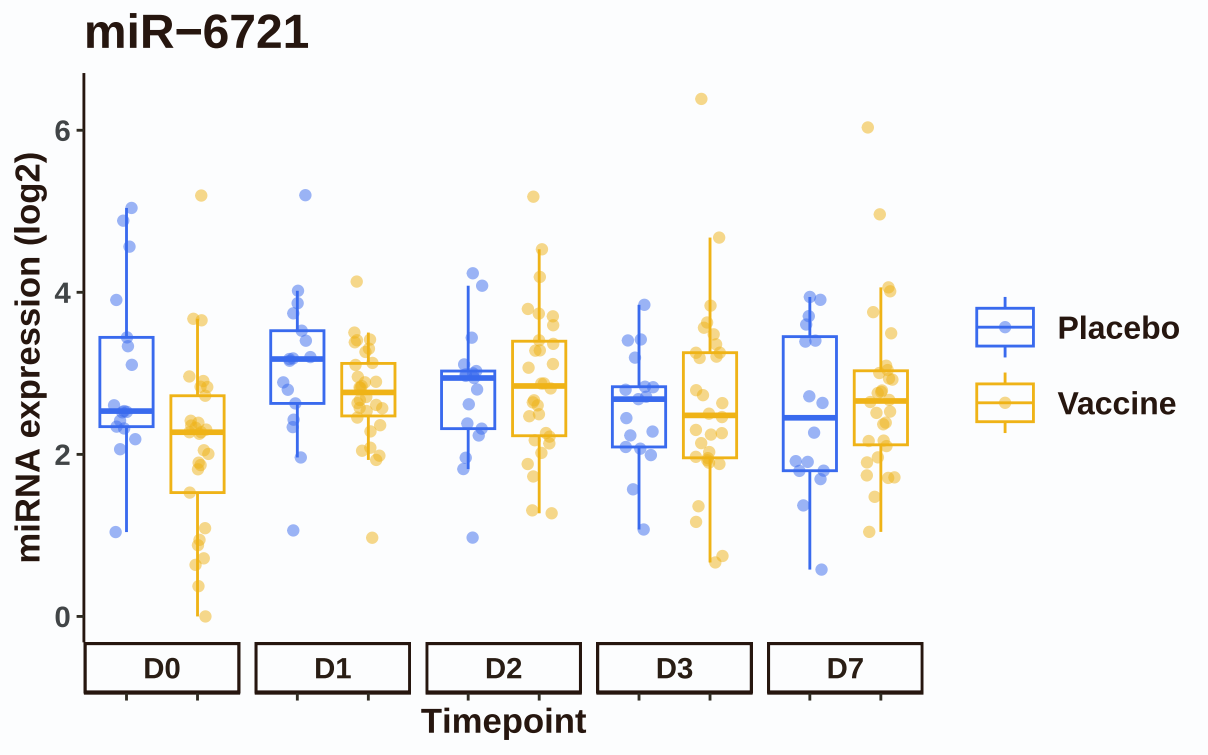

miR−6721
miRNA expression (log2)
0
2
4
6
D0
D1
D2
D3
D7
Timepoint
Placebo
Vaccine

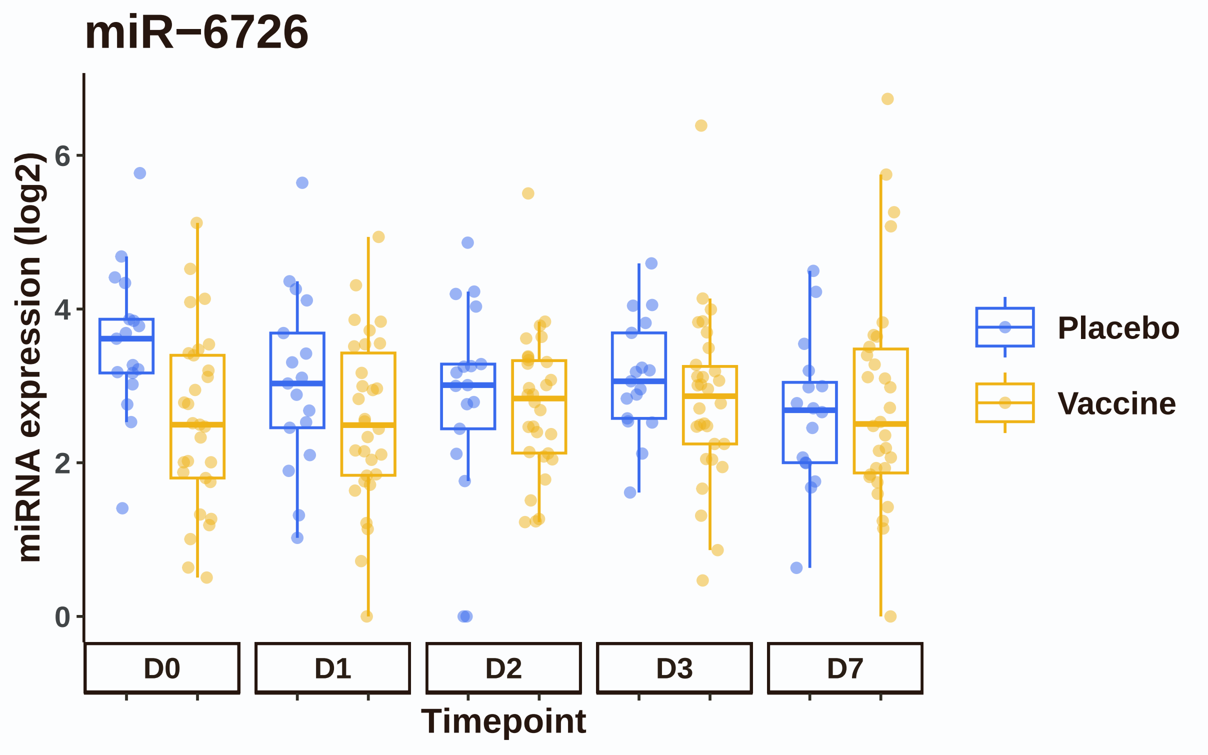

miR−6726
miRNA expression (log2)
6
4
2
0
D0
D1
D2
D3
D7
Timepoint
Placebo
Vaccine

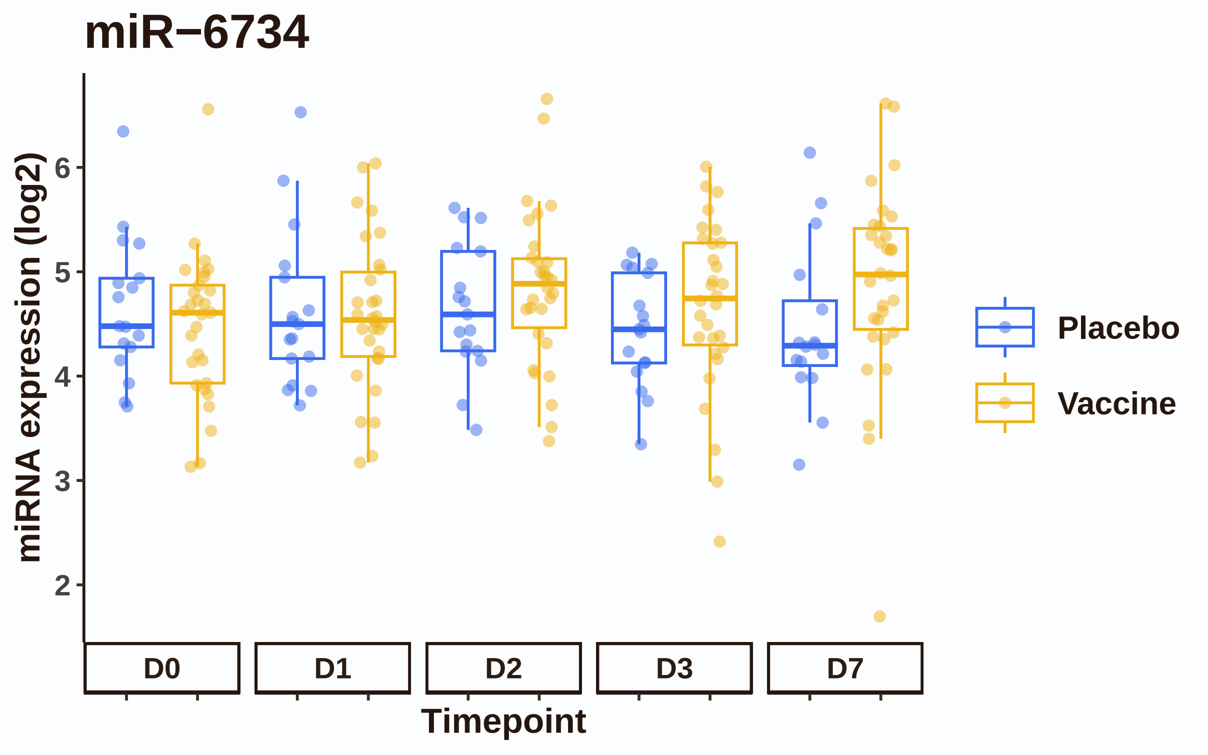
miR−6734
miRNA expression (log2)
2
3
4
5
6
D0
D1
D2
D3
D7
Timepoint
Placebo
Vaccine

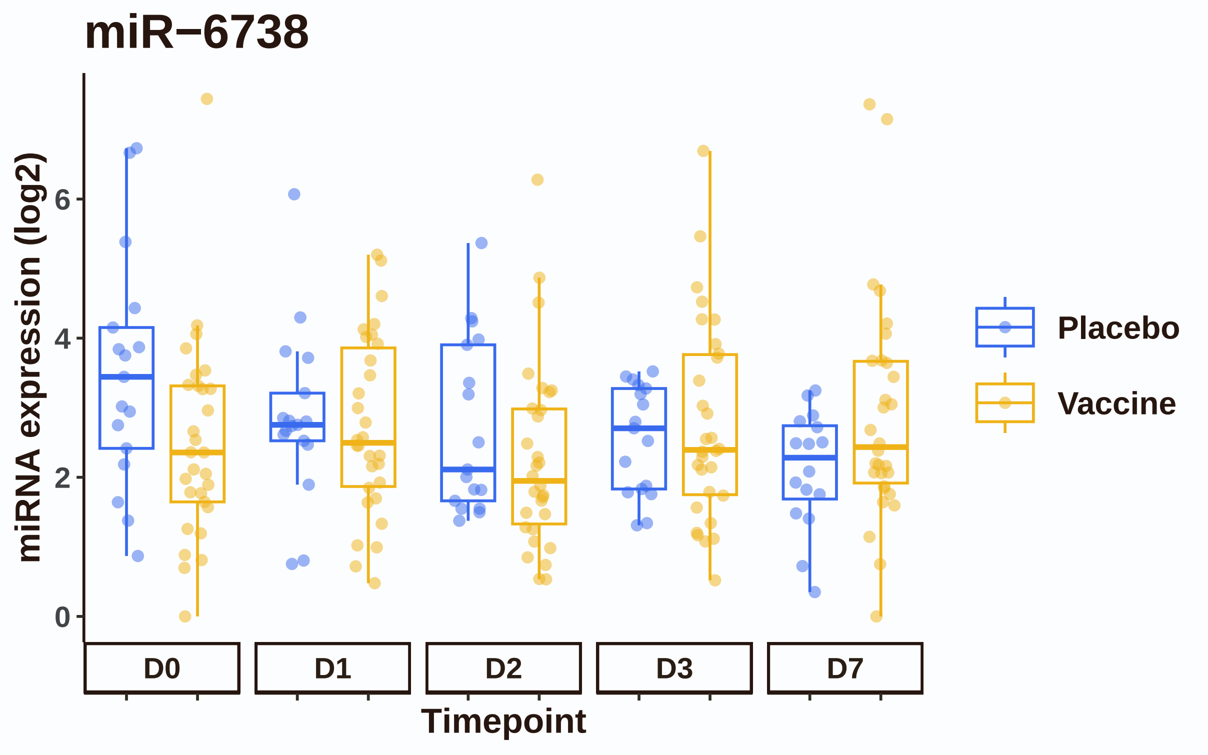
miR−6738
miRNA expression (log2)
0
2
4
6
D0
D1
D2
D3
D7
Timepoint
Placebo
Vaccine

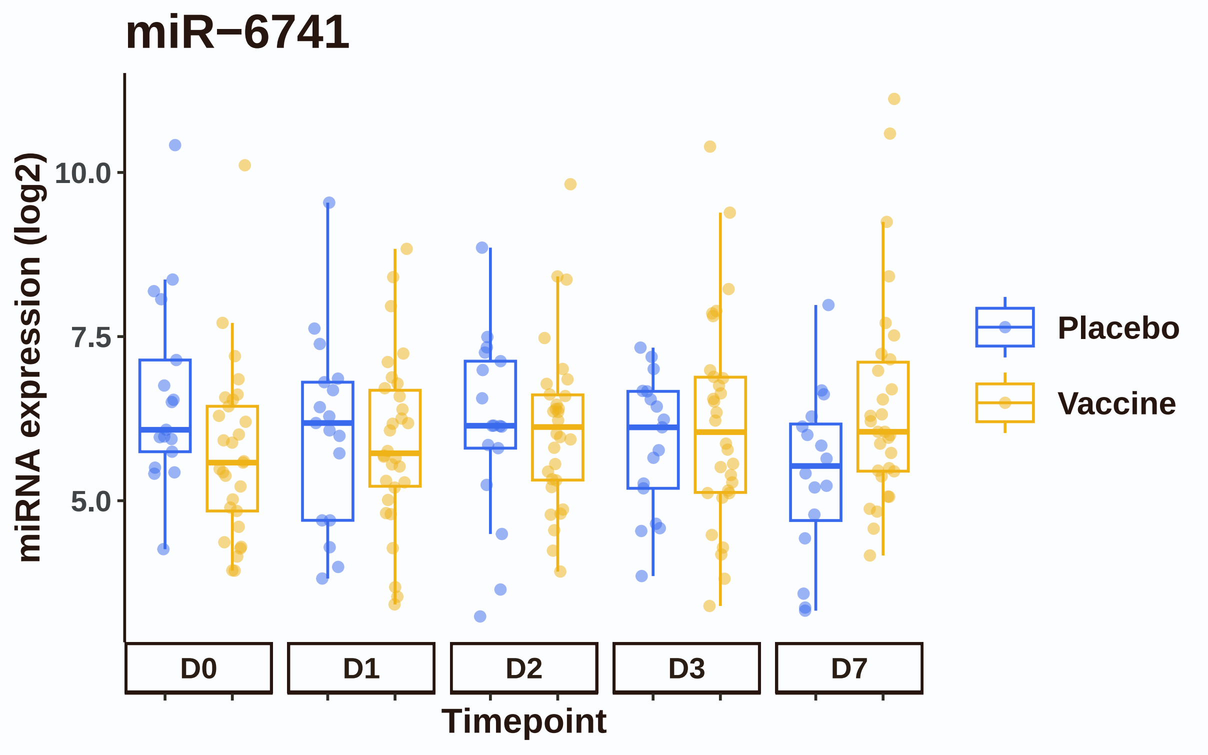

miR−6741
miRNA expression (log2)
10.0
7.5
5.0
D0
D1
D2
D3
D7
Timepoint
Placebo
Vaccine

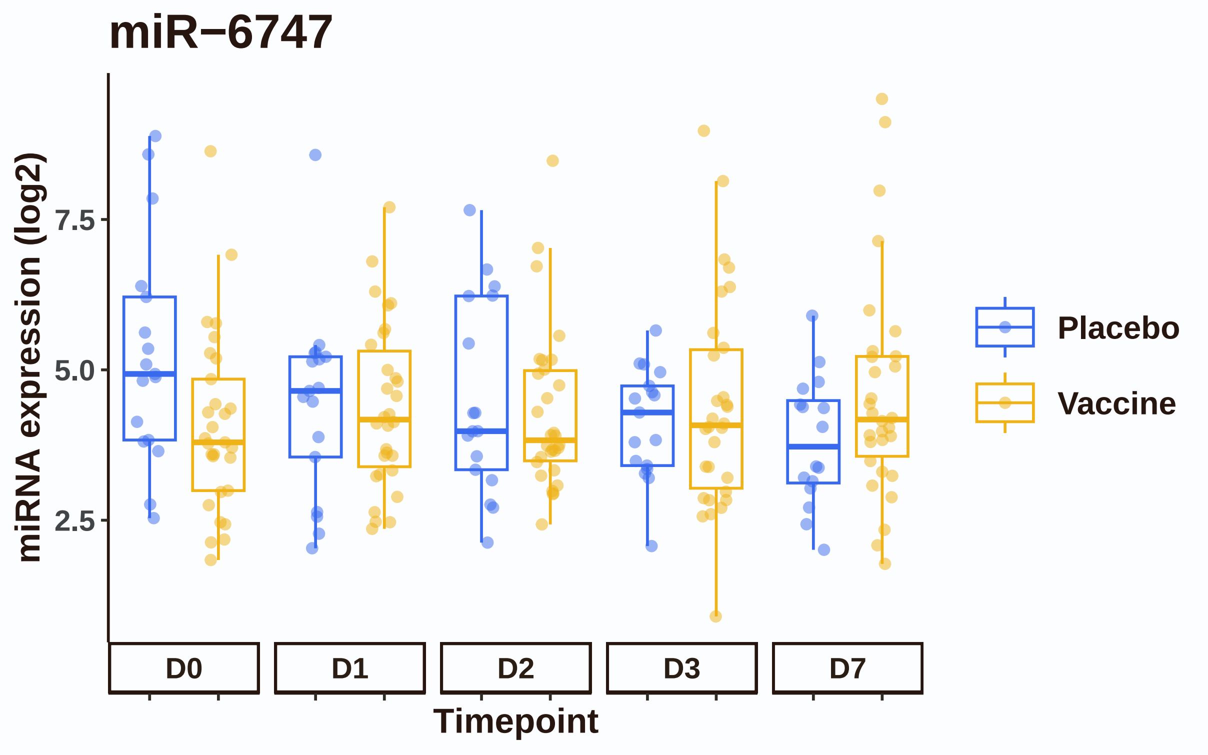
miR−6747
miRNA expression (log2)
7.5
5.0
2.5
D0
D1
D2
D3
D7
Timepoint
Placebo
Vaccine

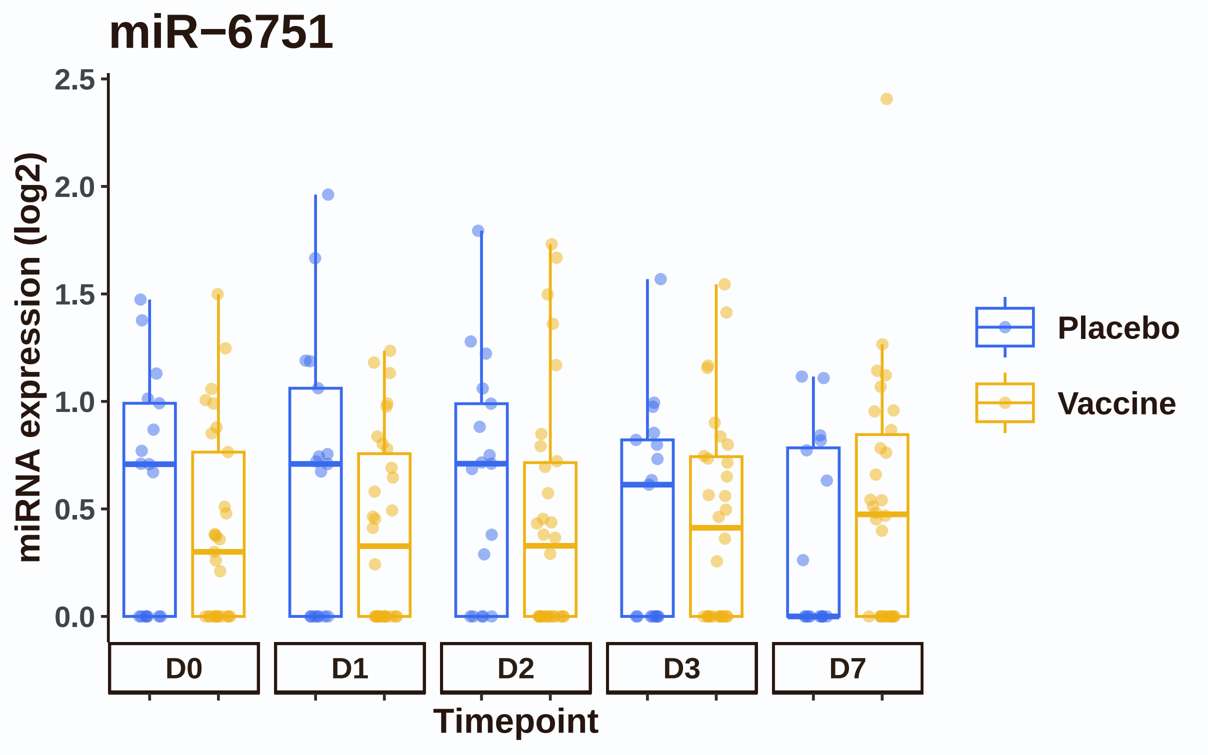

miR−6751
miRNA expression (log2)
2.5
2.0
1.5
1.0
0.5
0.0
D0
D1
D2
D3
D7
Timepoint
Placebo
Vaccine

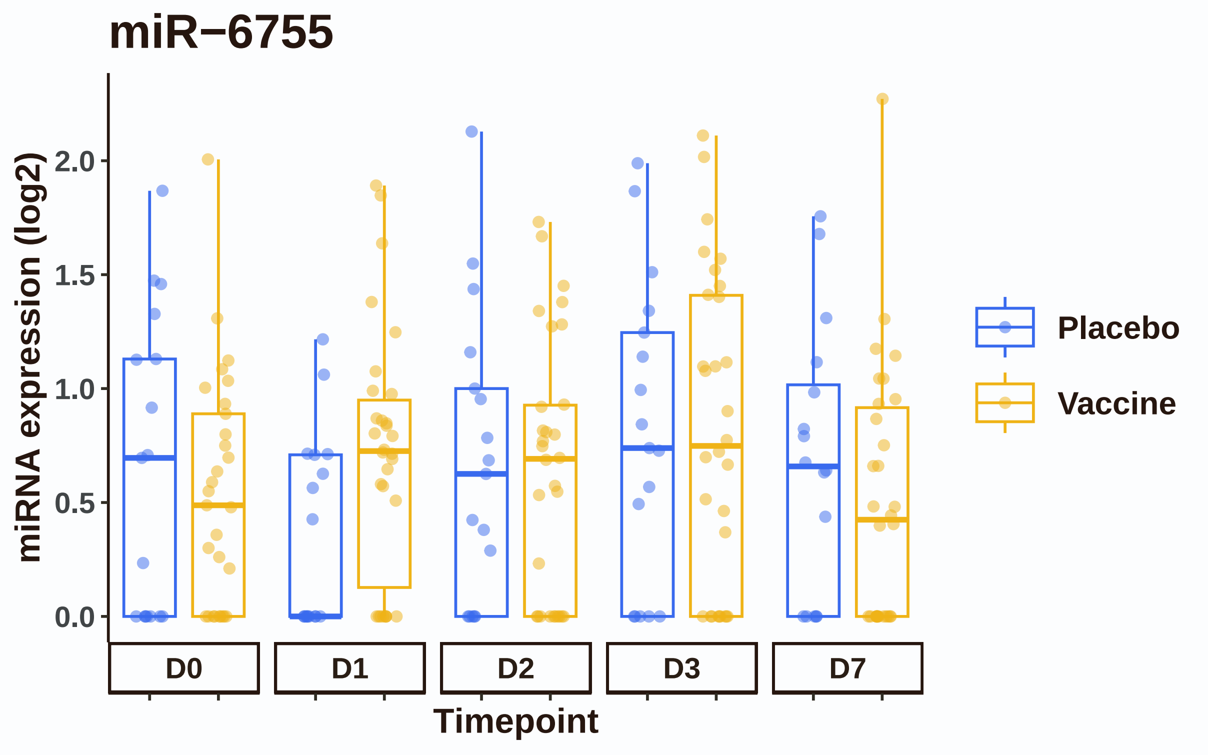
miR−6755
miRNA expression (log2)
2.0
1.5
1.0
0.5
0.0
D0
D1
D2
D3
D7
Timepoint
Placebo
Vaccine

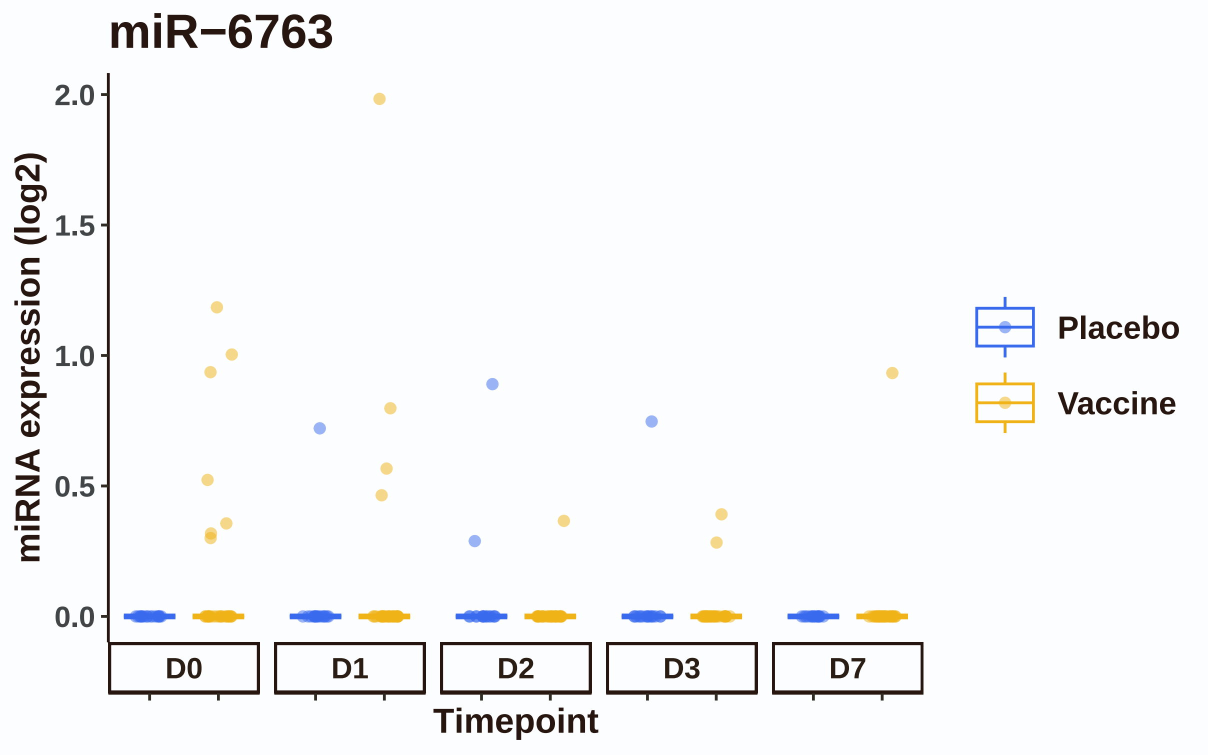
miR−6763
miRNA expression (log2)
2.0
1.5
1.0
0.5
0.0
D0
D1
D2
D3
D7
Timepoint
Placebo
Vaccine

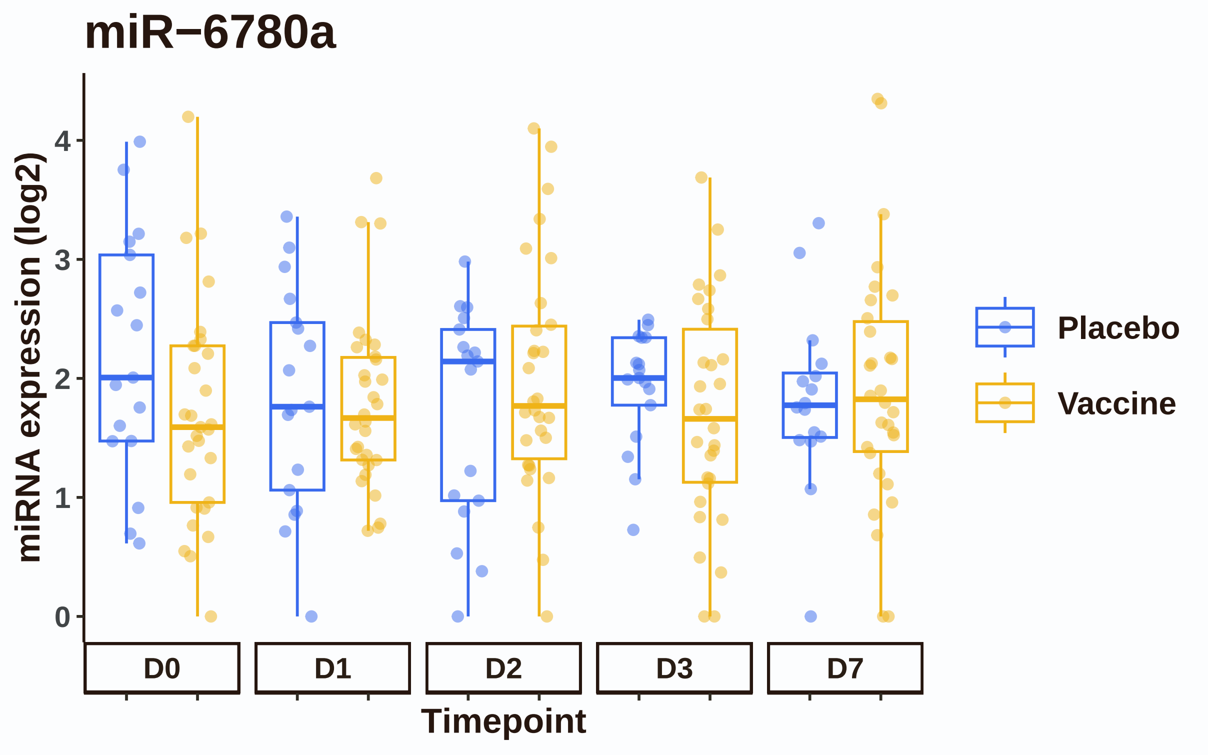

miR−6780a
miRNA expression (log2)
0
1
2
3
4
D0
D1
D2
D3
D7
Timepoint
Placebo
Vaccine

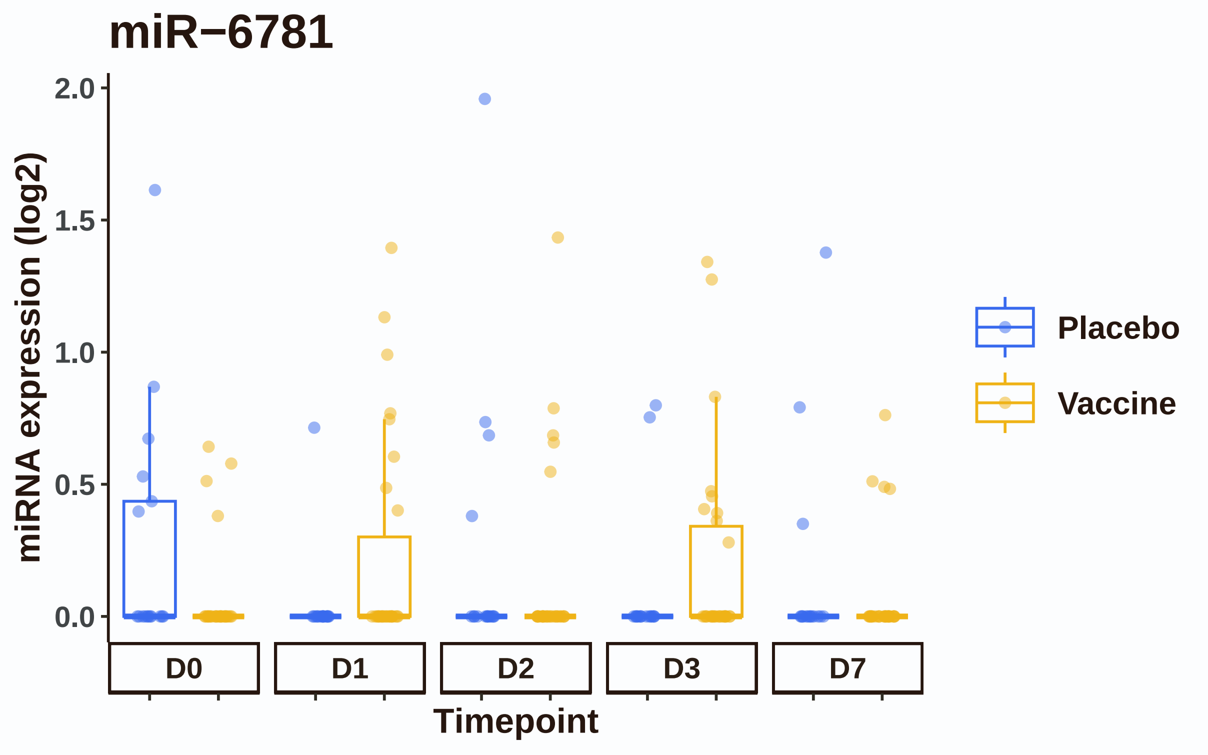
miR−6781
miRNA expression (log2)
2.0
1.5
1.0
0.5
0.0
D0
D1
D2
D3
D7
Timepoint
Placebo
Vaccine

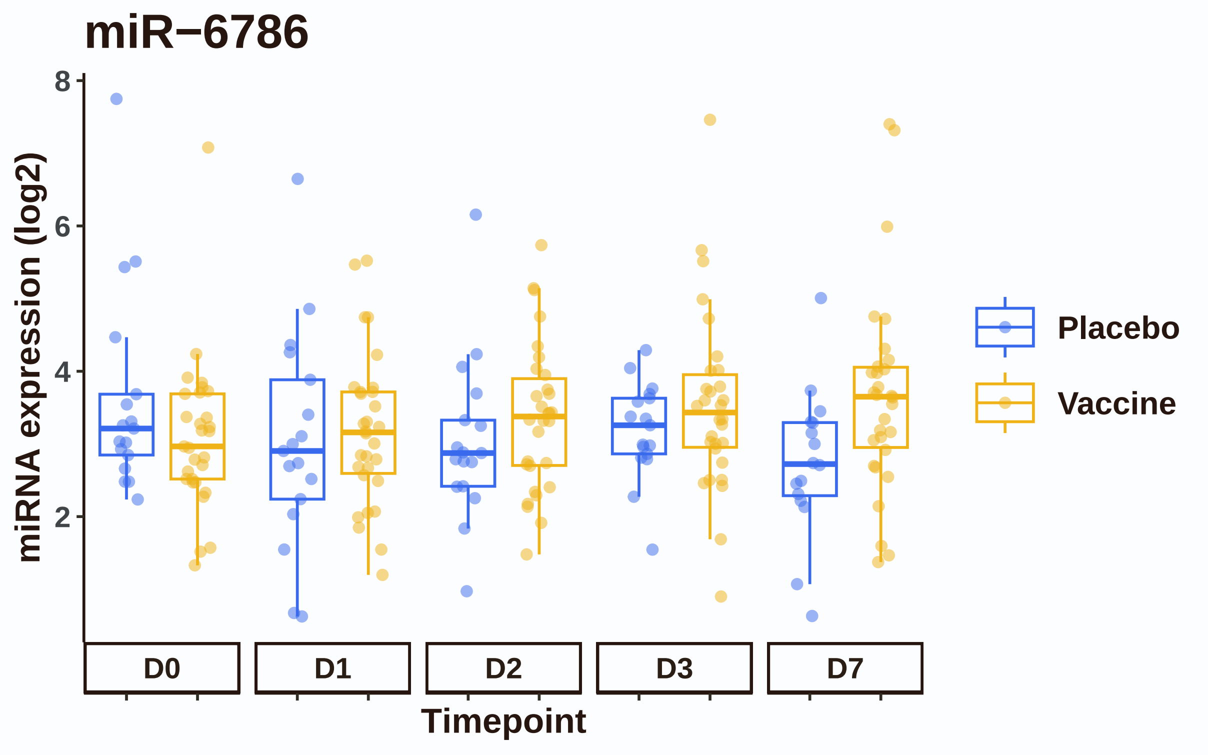
miR−6786
miRNA expression (log2)
8
6
4
2
D0
D1
D2
D3
D7
Timepoint
Placebo
Vaccine

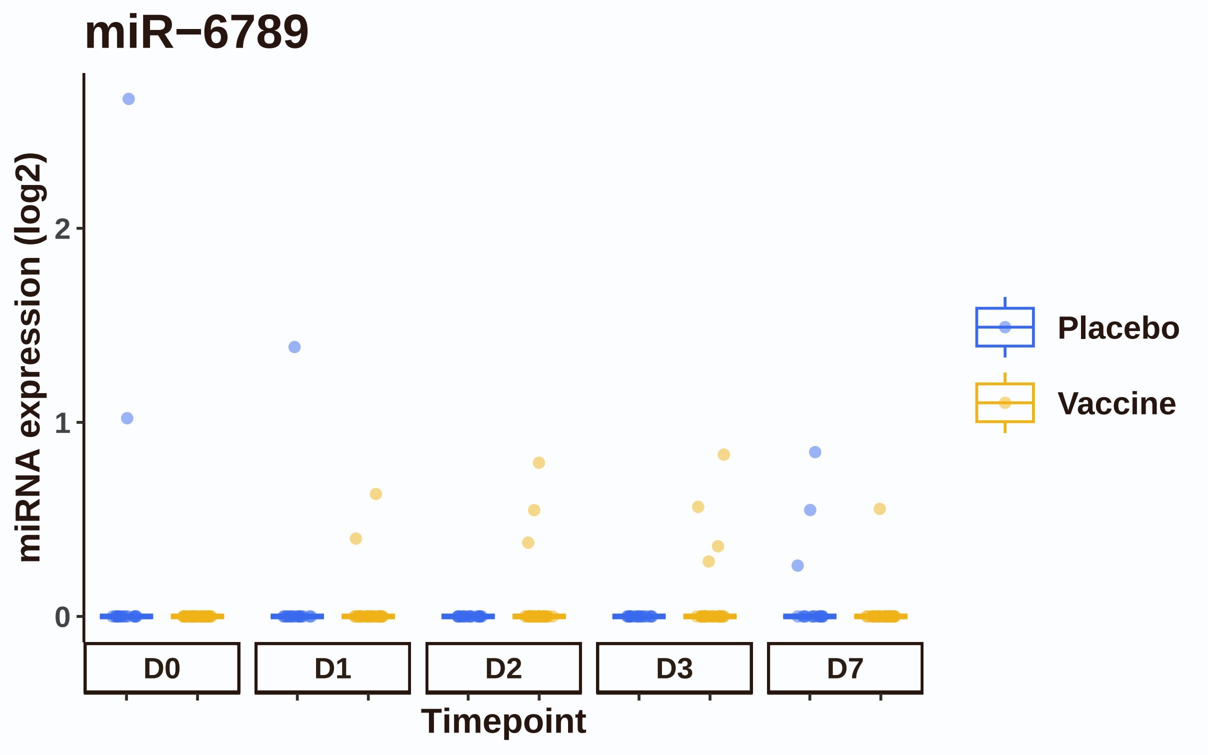
miR−6789
miRNA expression (log2)
0
1
2
D0
D1
D2
D3
D7
Timepoint
Placebo
Vaccine

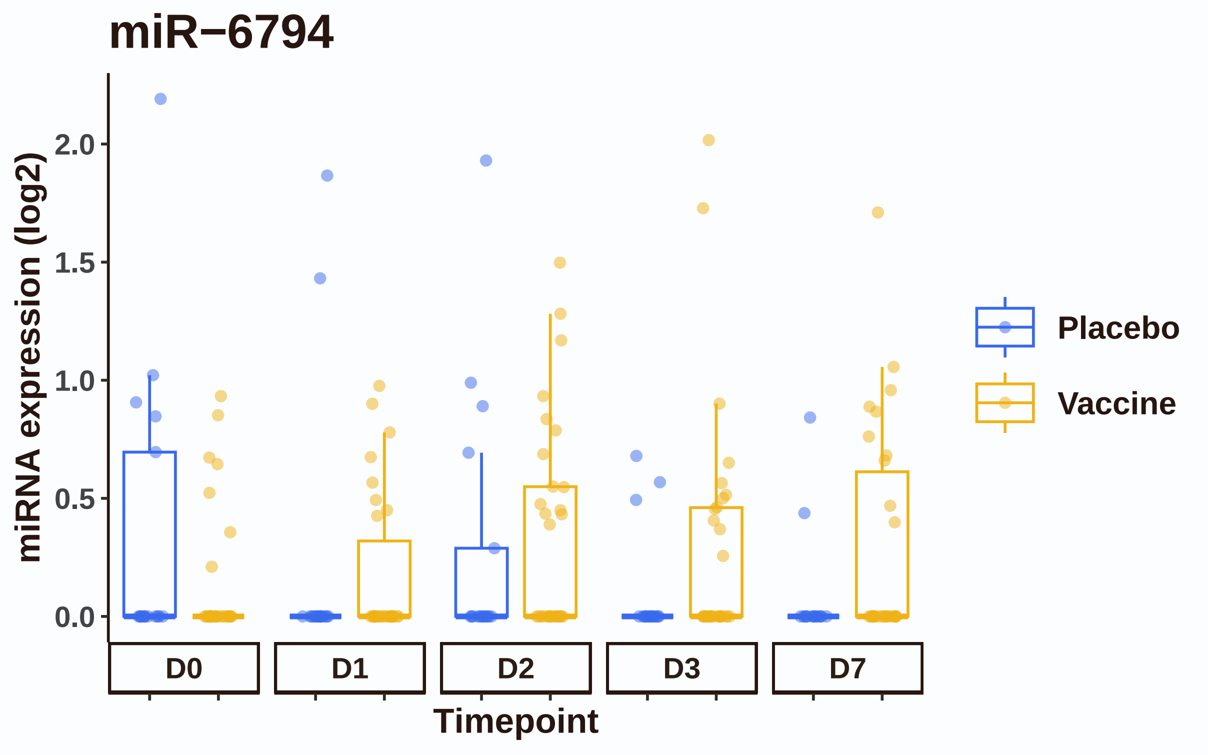

miR−6794
miRNA expression (log2)
2.0
1.5
1.0
0.5
0.0
D0
D1
D2
D3
D7
Timepoint
Placebo
Vaccine

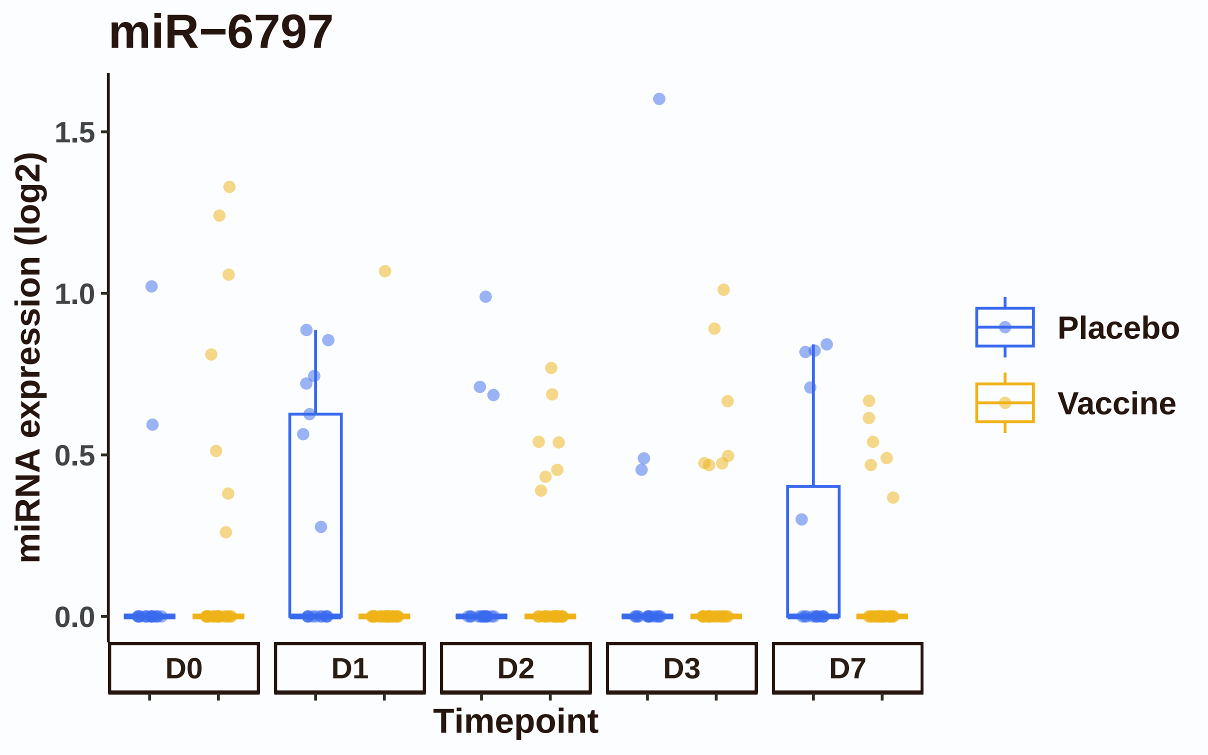

miR−6797
miRNA expression (log2)
1.5
1.0
0.5
0.0
D0
D1
D2
D3
D7
Timepoint
Placebo
Vaccine

miR−6802

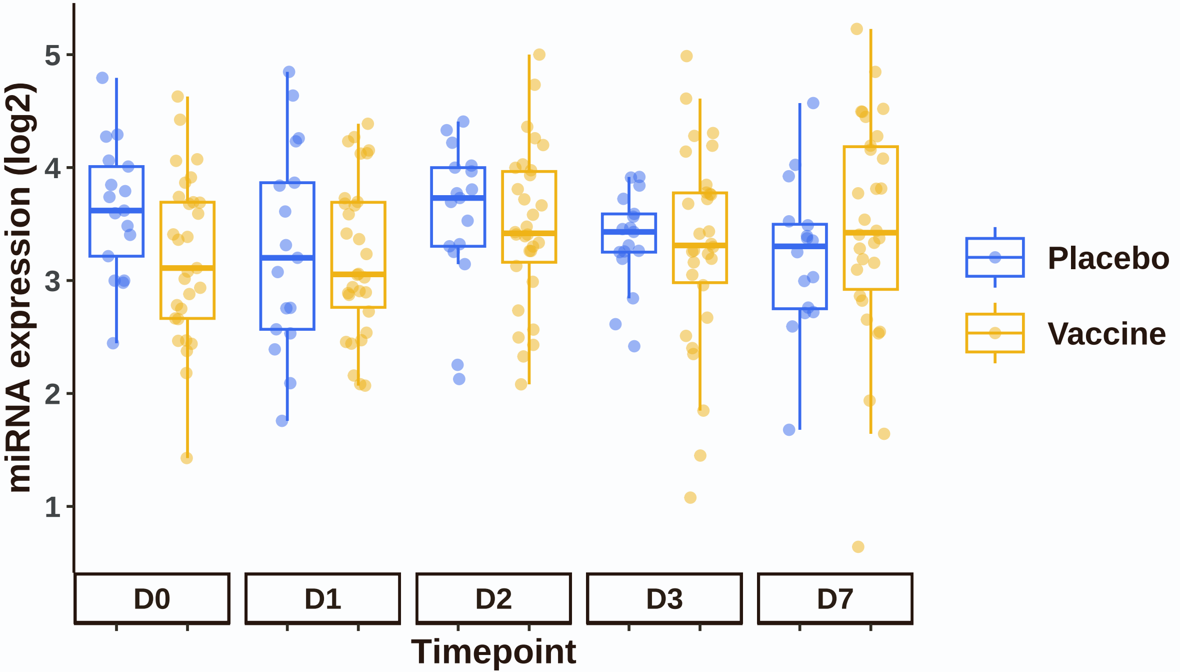

miRNA expression (log2)
5
4
3
2
1
D0
D1
D2
D3
D7
Timepoint
Placebo
Vaccine

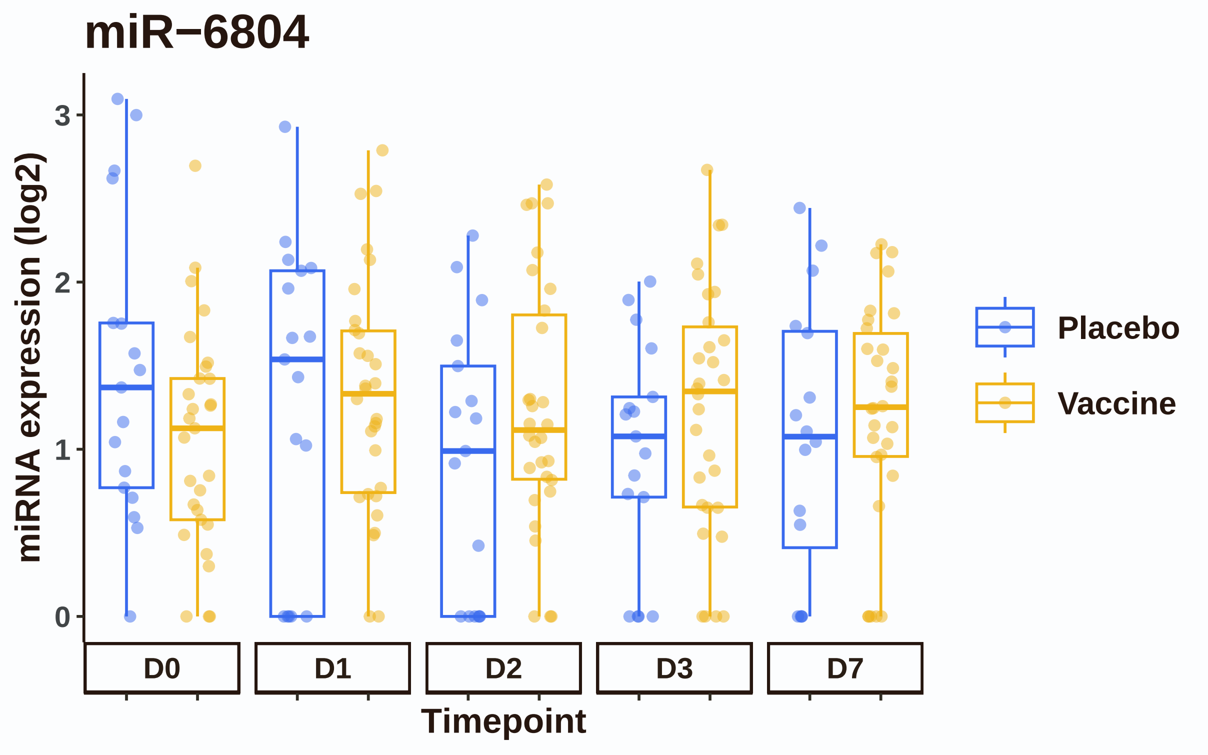
miR−6804
miRNA expression (log2)
3
2
1
0
D0
D1
D2
D3
D7
Timepoint
Placebo
Vaccine

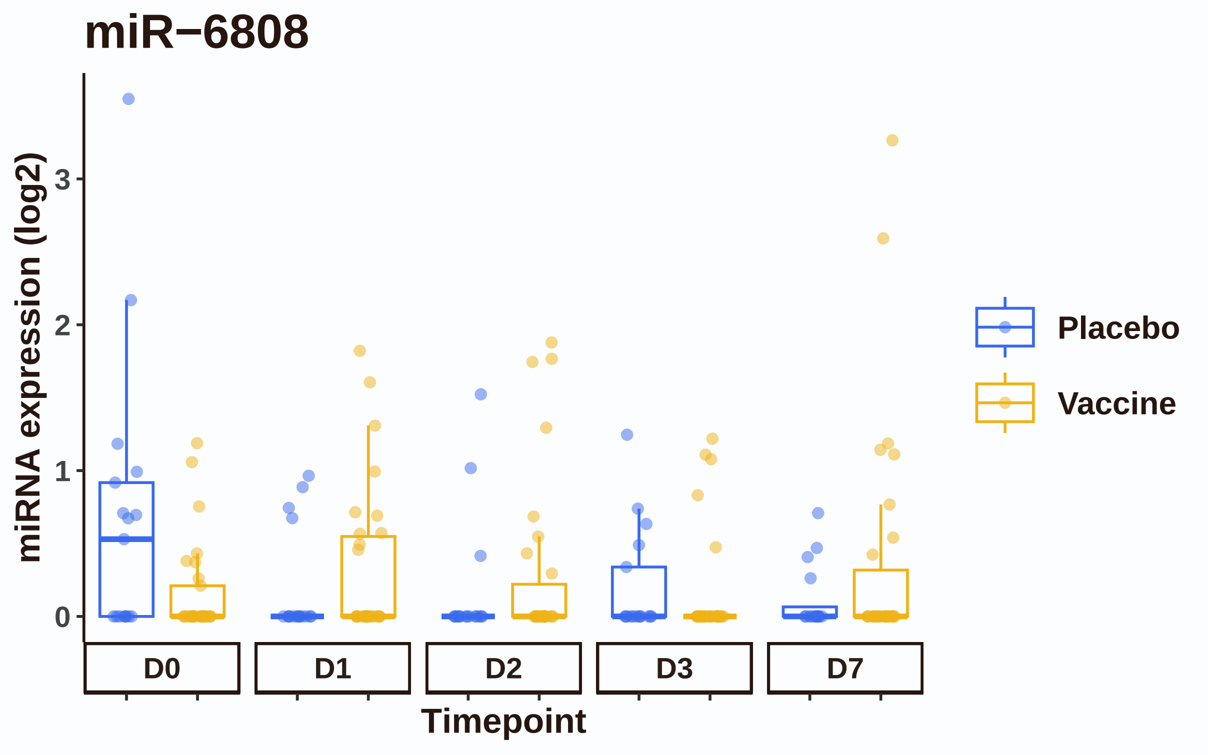

miR−6808
miRNA expression (log2)
3
2
1
0
D0
D1
D2
D3
D7
Timepoint
Placebo
Vaccine

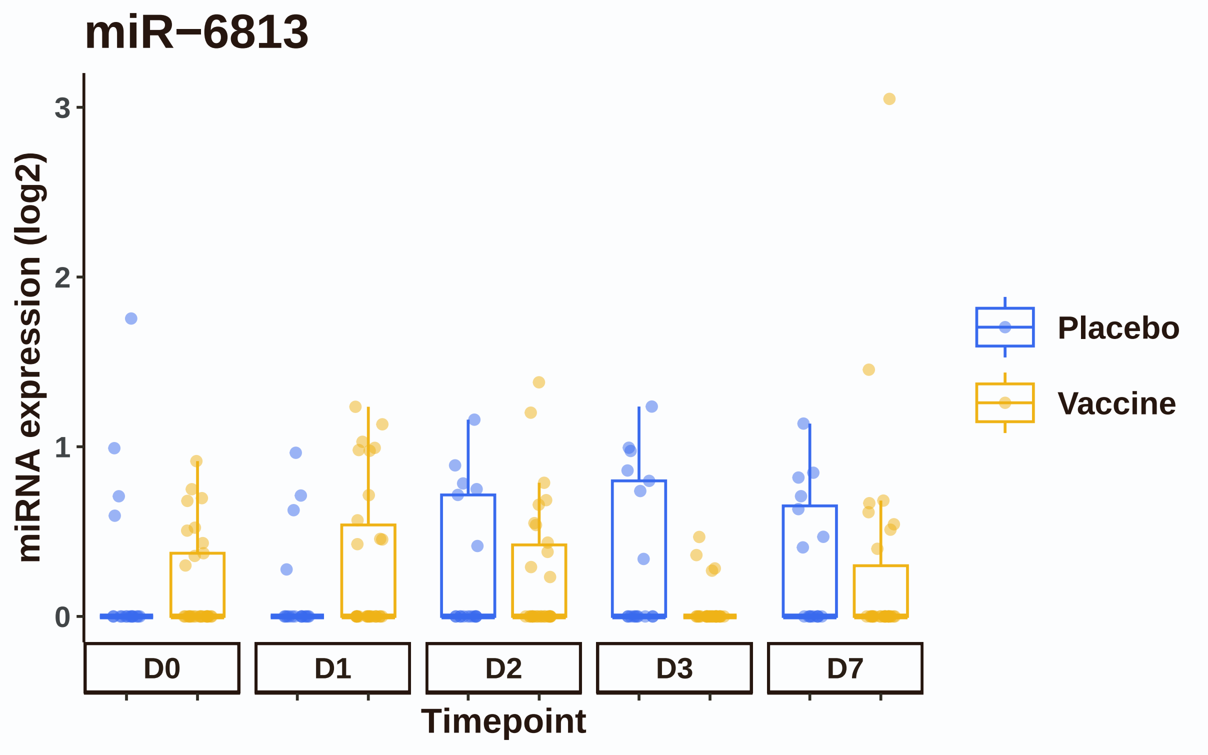
miR−6813
miRNA expression (log2)
3
2
1
0
D0
D1
D2
D3
D7
Timepoint
Placebo
Vaccine

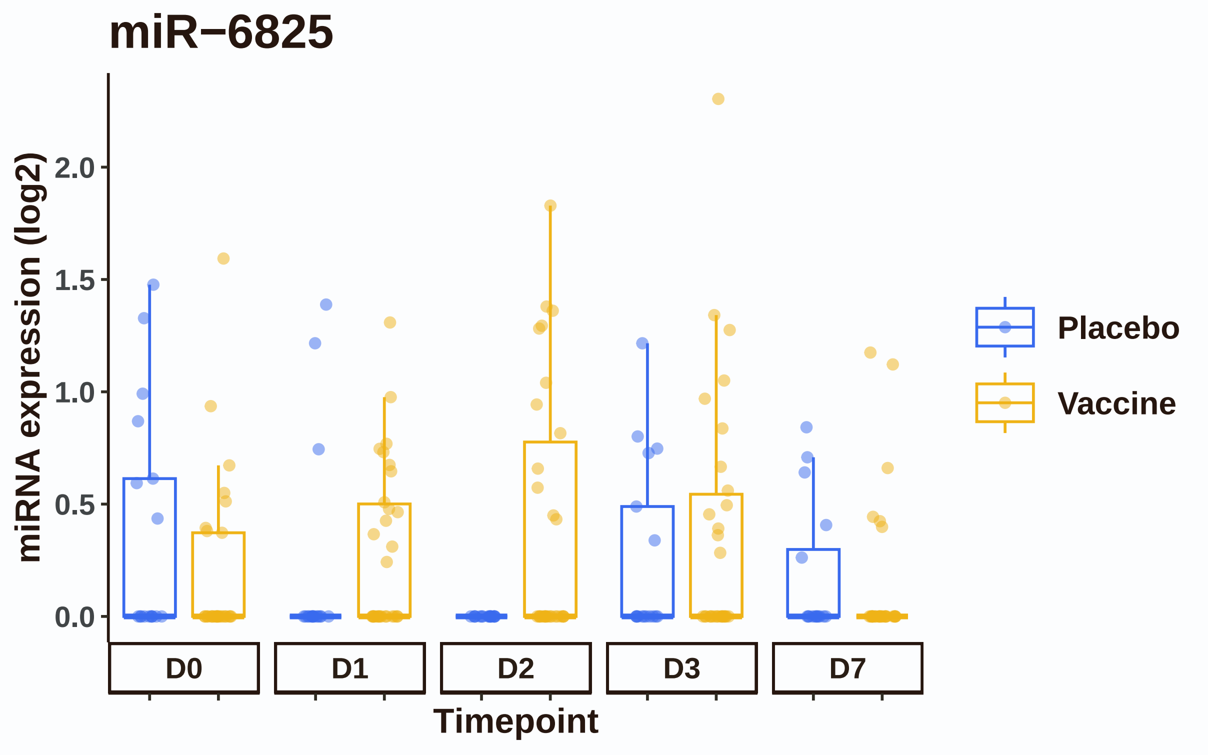
miR−6825
miRNA expression (log2)
2.0
1.5
1.0
0.5
0.0
D0
D1
D2
D3
D7
Timepoint
Placebo
Vaccine

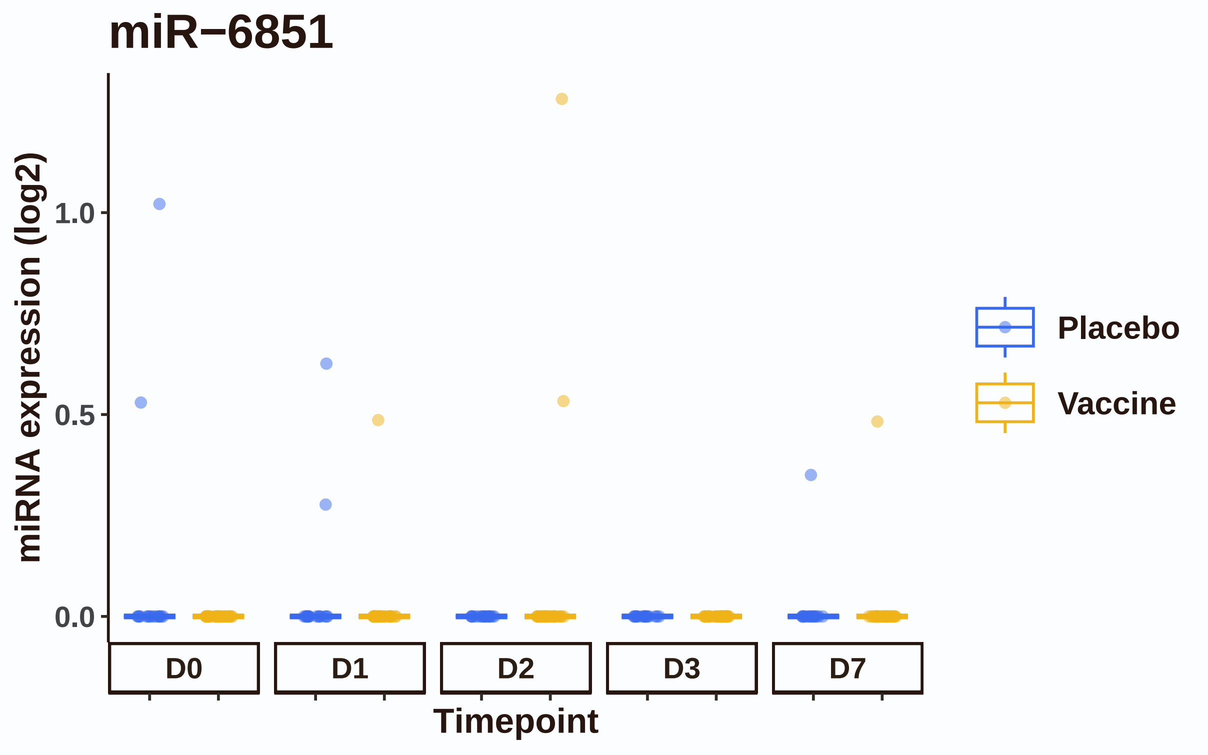
miR−6851
miRNA expression (log2)
1.0
0.5
0.0
D0
D1
D2
D3
D7
Timepoint
Placebo
Vaccine

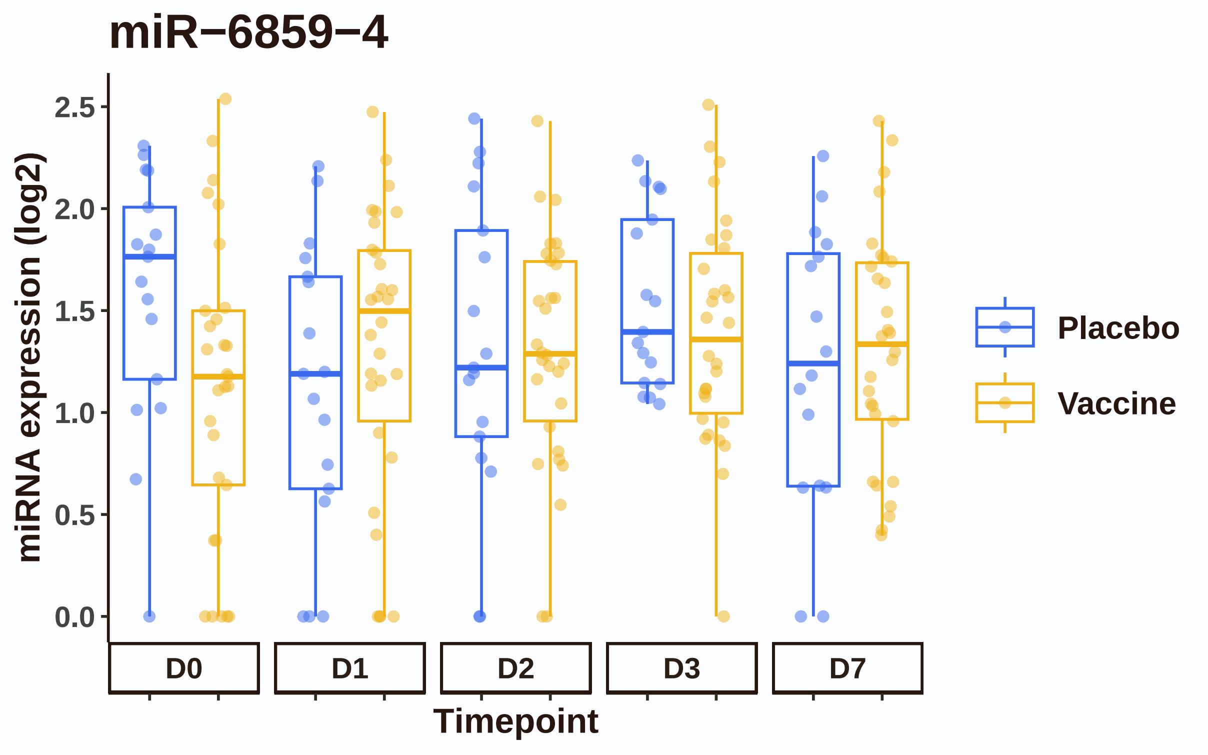
miR−6859−4
miRNA expression (log2)
2.5
2.0
1.5
1.0
0.5
0.0
D0
D1
D2
D3
D7
Timepoint
Placebo
Vaccine

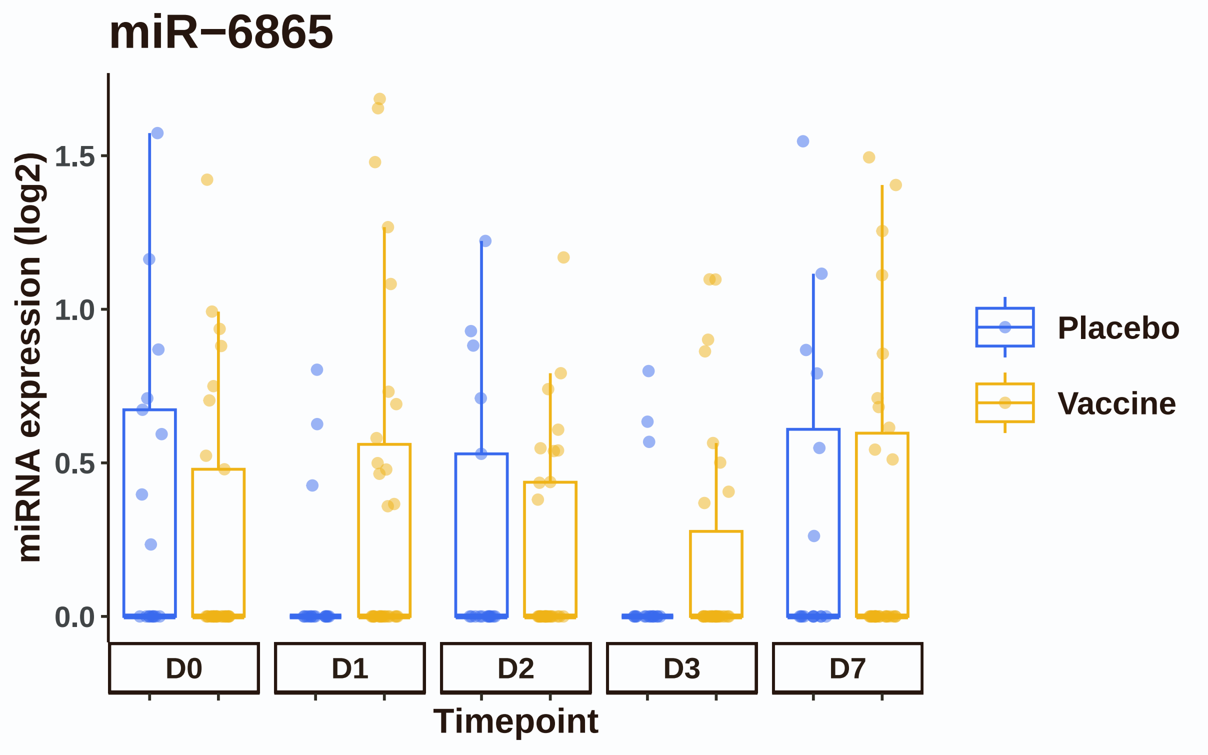

miR−6865
miRNA expression (log2)
1.5
1.0
0.5
0.0
D0
D1
D2
D3
D7
Timepoint
Placebo
Vaccine

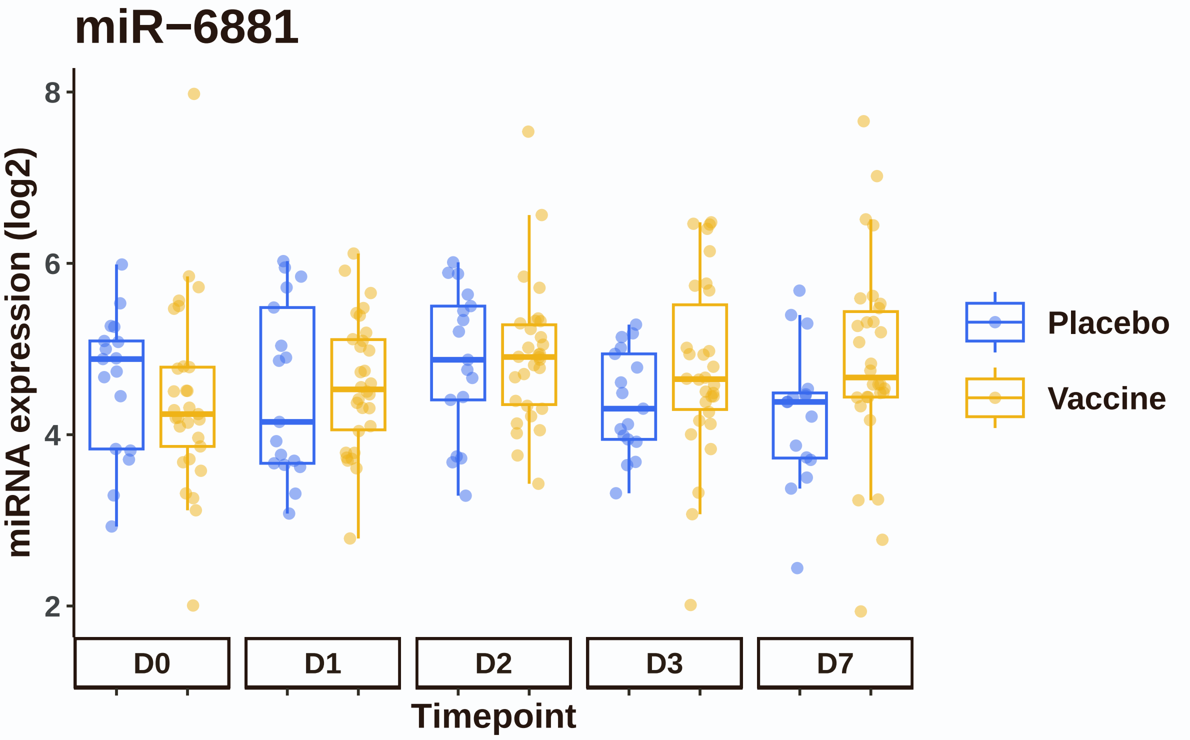

miR−6881
miRNA expression (log2)
8
6
4
2
D0
D1
D2
D3
D7
Timepoint
Placebo
Vaccine

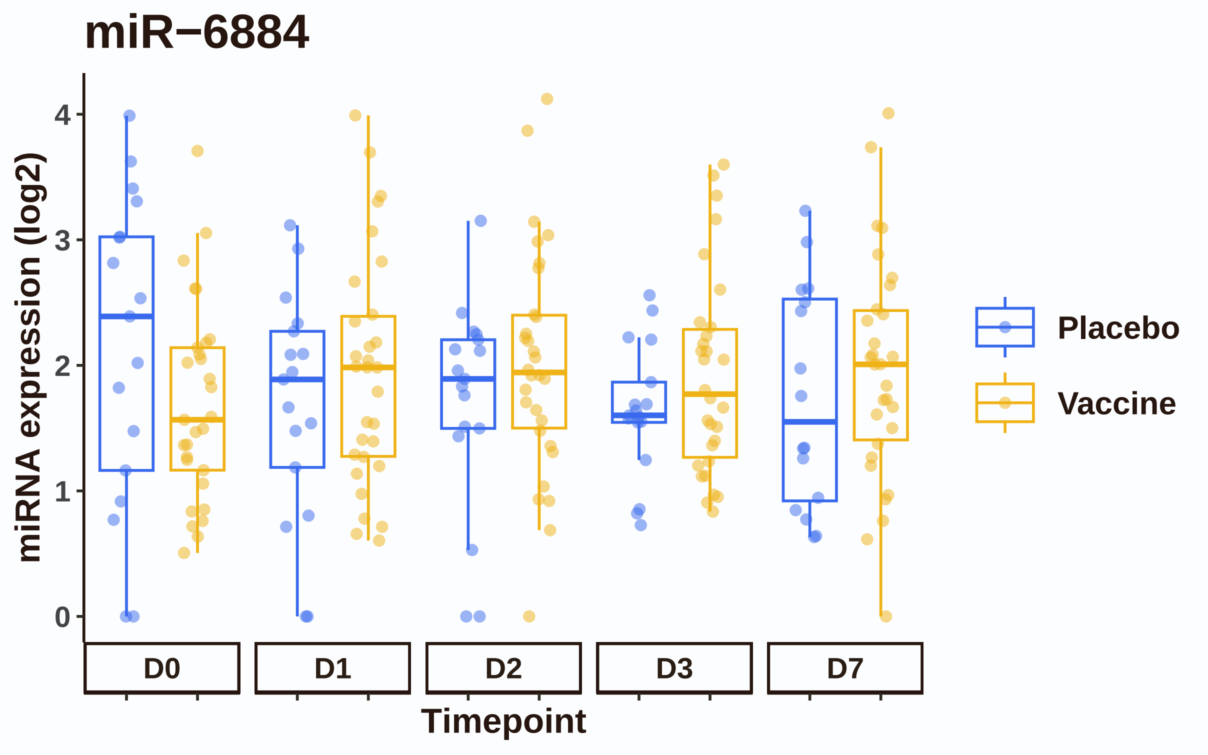

miR−6884
miRNA expression (log2)
0
1
2
3
4
D0
D1
D2
D3
D7
Timepoint
Placebo
Vaccine

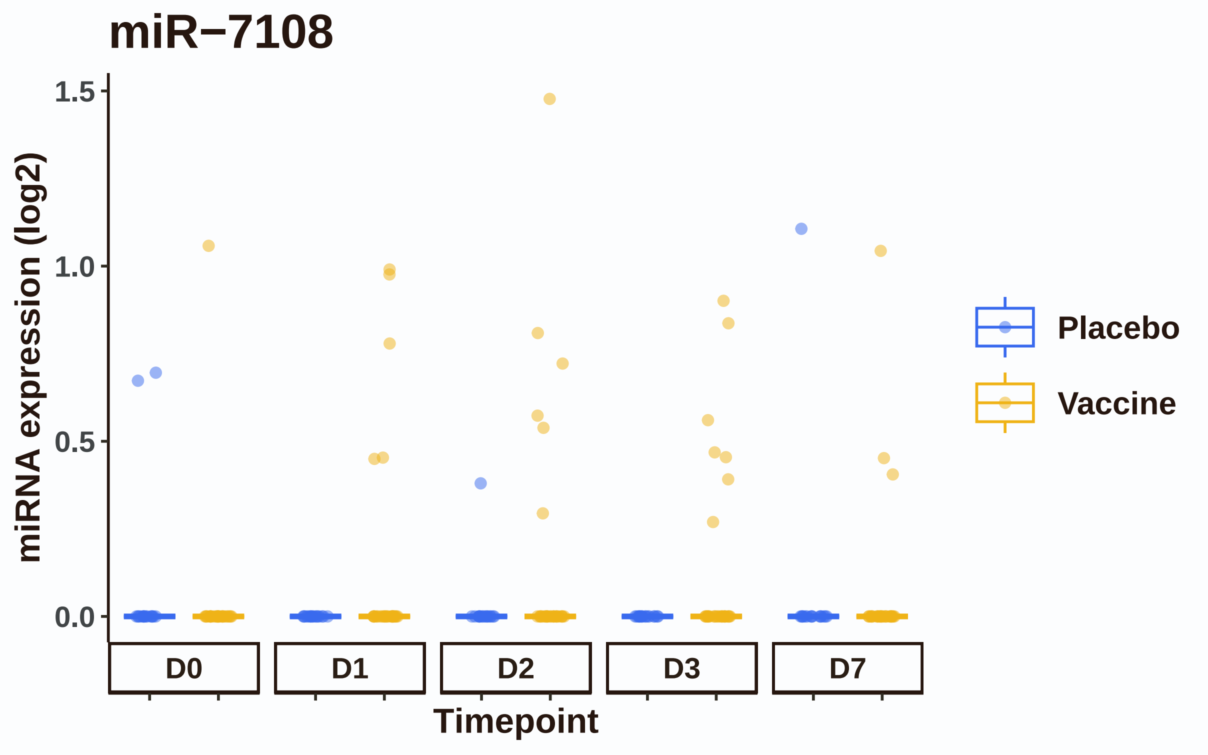
miR−7108
miRNA expression (log2)
1.5
1.0
0.5
0.0
D0
D1
D2
D3
D7
Timepoint
Placebo
Vaccine

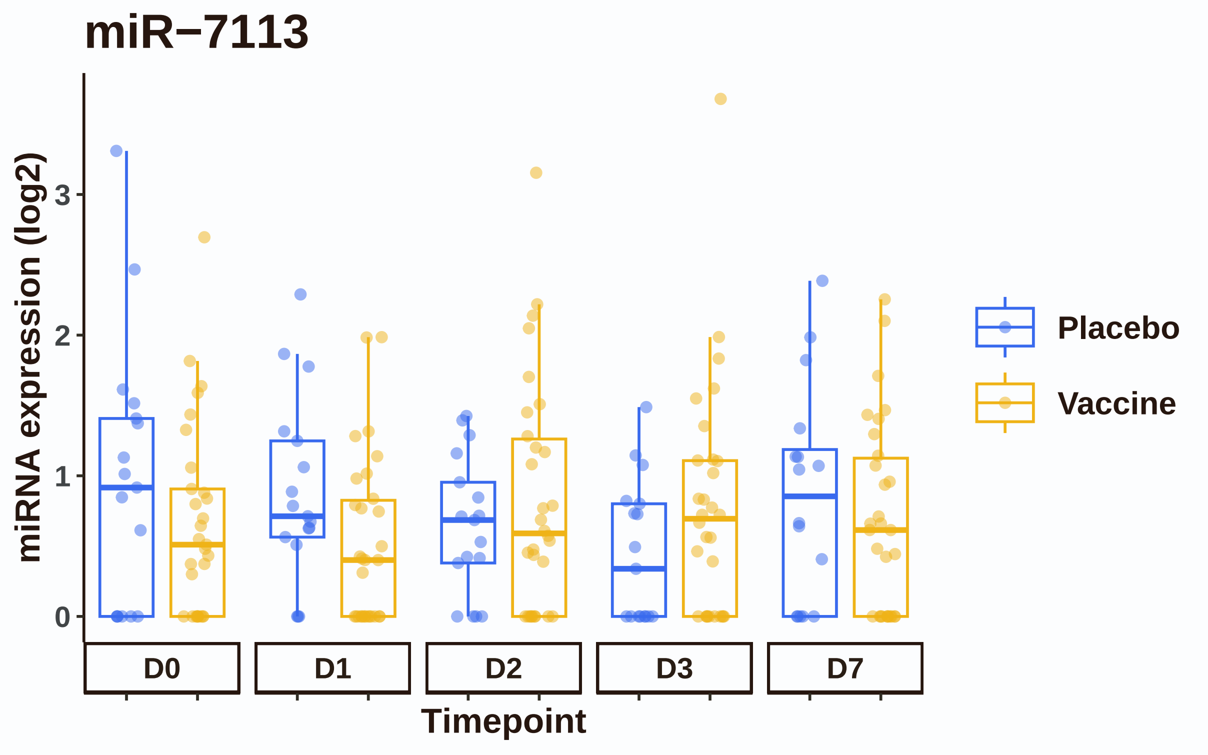
miR−7113
miRNA expression (log2)
0
1
2
3
D0
D1
D2
D3
D7
Timepoint
Placebo
Vaccine

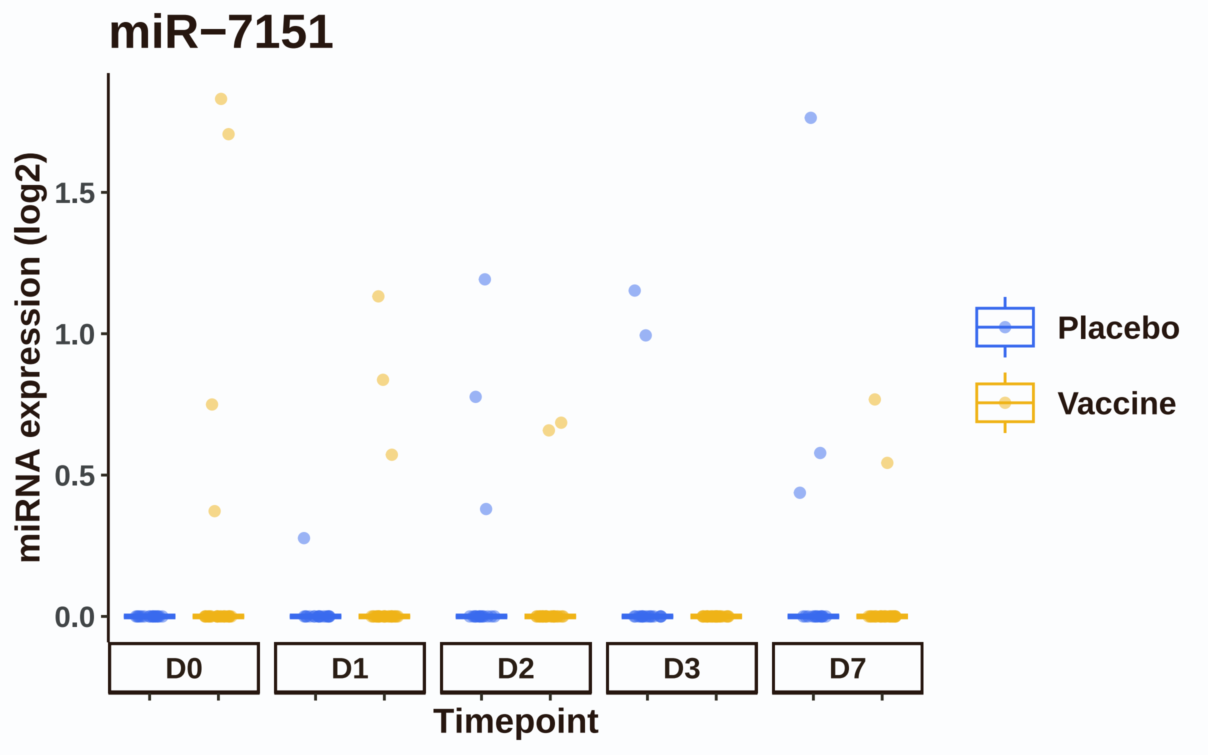
miR−7151
miRNA expression (log2)
1.5
1.0
0.5
0.0
D0
D1
D2
D3
D7
Timepoint
Placebo
Vaccine

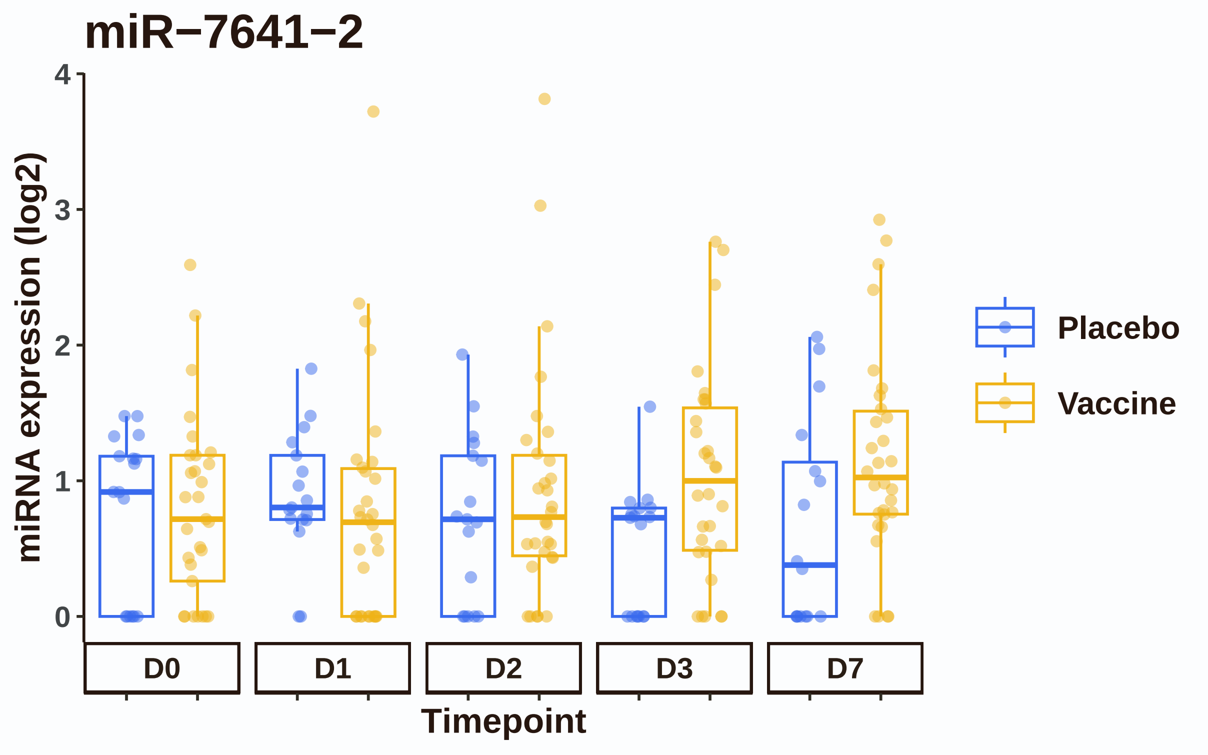

miR−7641−2
miRNA expression (log2)
0
1
2
3
4
D0
D1
D2
D3
D7
Timepoint
Placebo
Vaccine

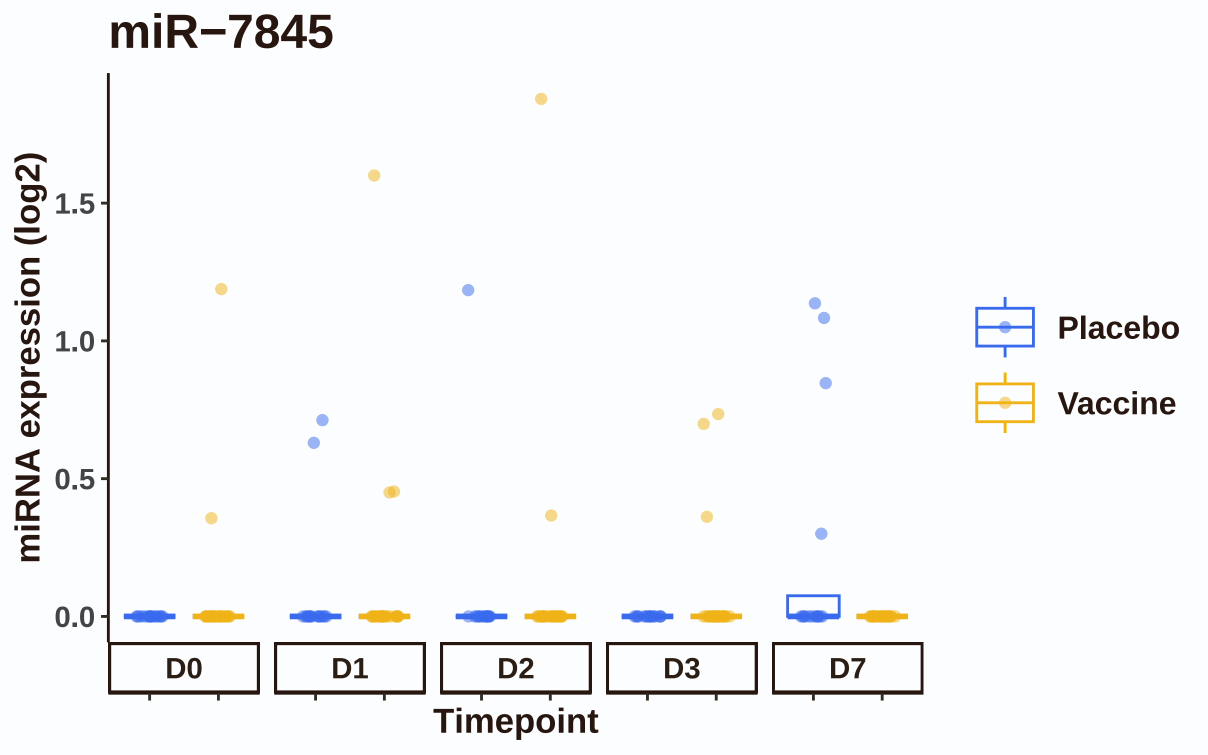
miR−7845
miRNA expression (log2)
0.0
0.5
1.0
1.5
D0
D1
D2
D3
D7
Timepoint
Placebo
Vaccine

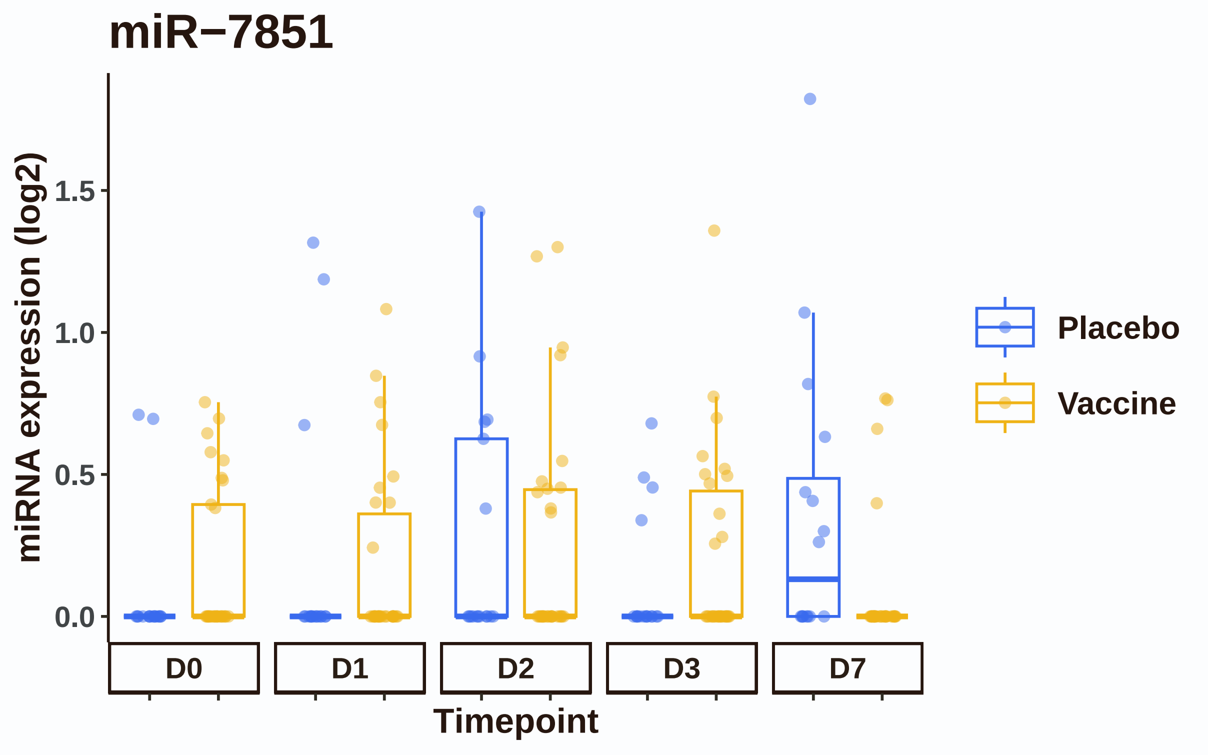

miR−7851
miRNA expression (log2)
1.5
1.0
0.5
0.0
D0
D1
D2
D3
D7
Timepoint
Placebo
Vaccine

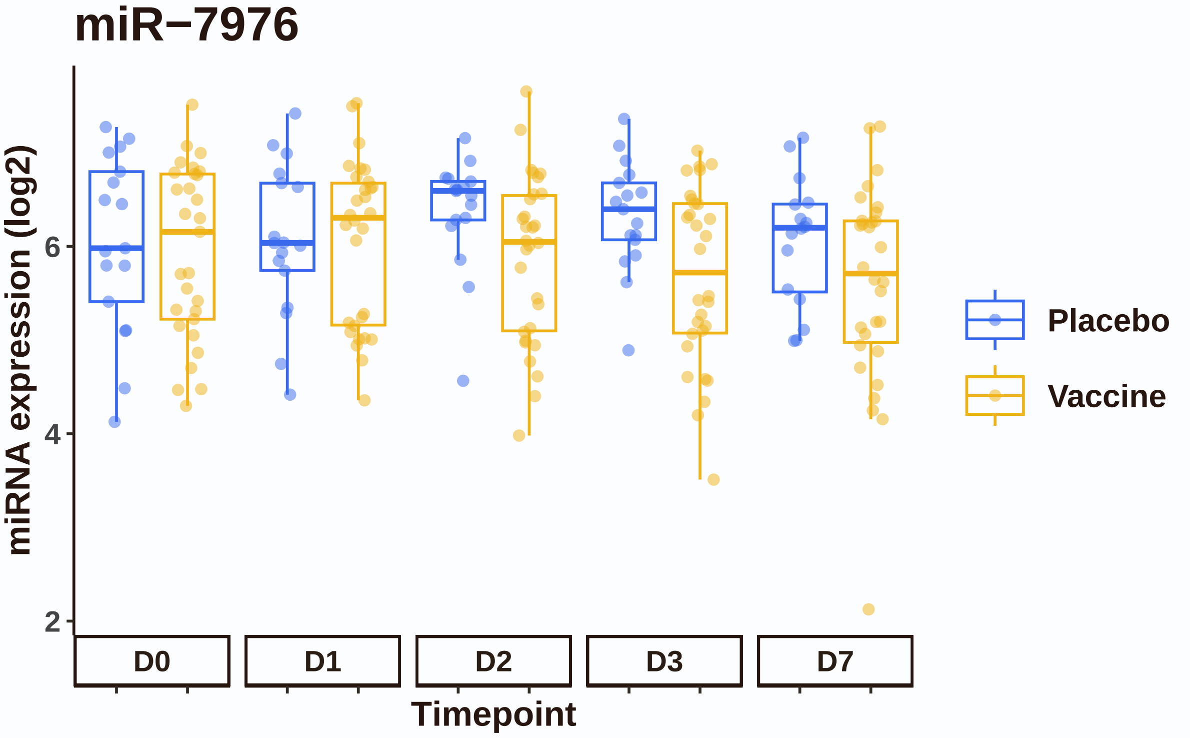
miR−7976
miRNA expression (log2)
2
4
6
D0
D1
D2
D3
D7
Timepoint
Placebo
Vaccine

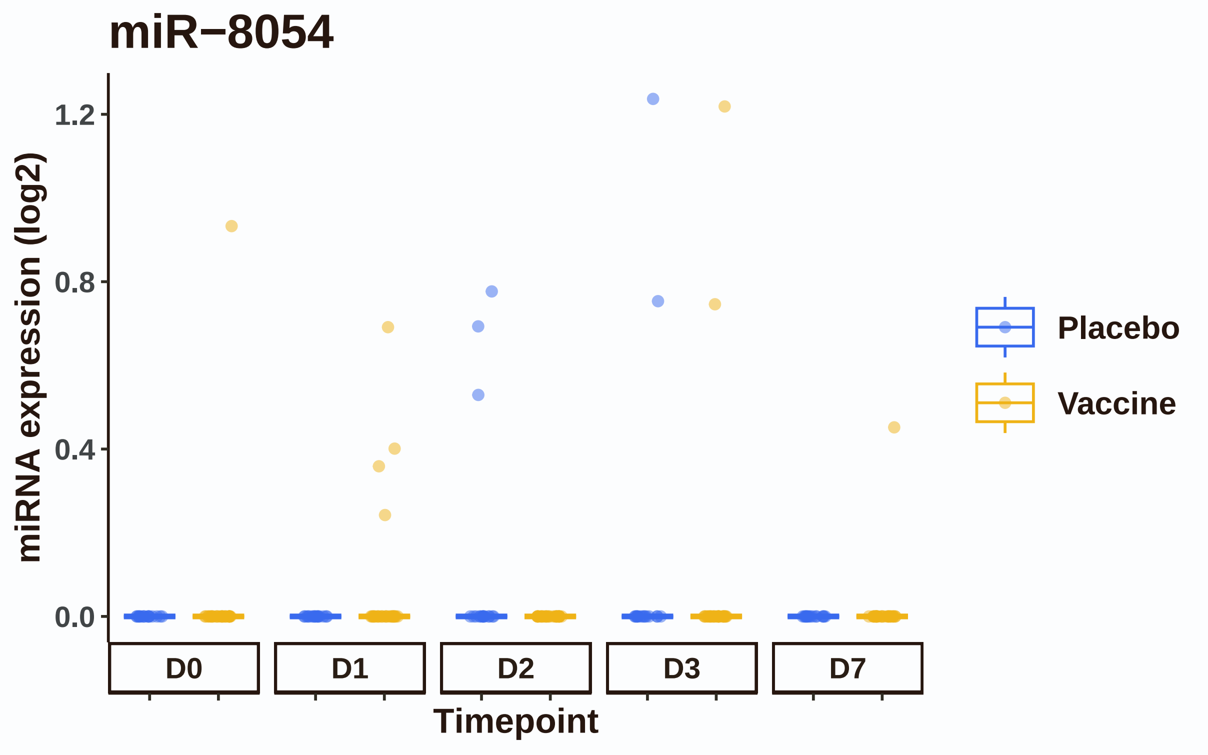

miR−8054
miRNA expression (log2)
1.2
0.8
0.4
0.0
D0
D1
D2
D3
D7
Timepoint
Placebo
Vaccine

Data S2

Data S2. Expression kinetics of the full list of the differentially expressed EV-miRNAs, Related to Figure 2 and 3.

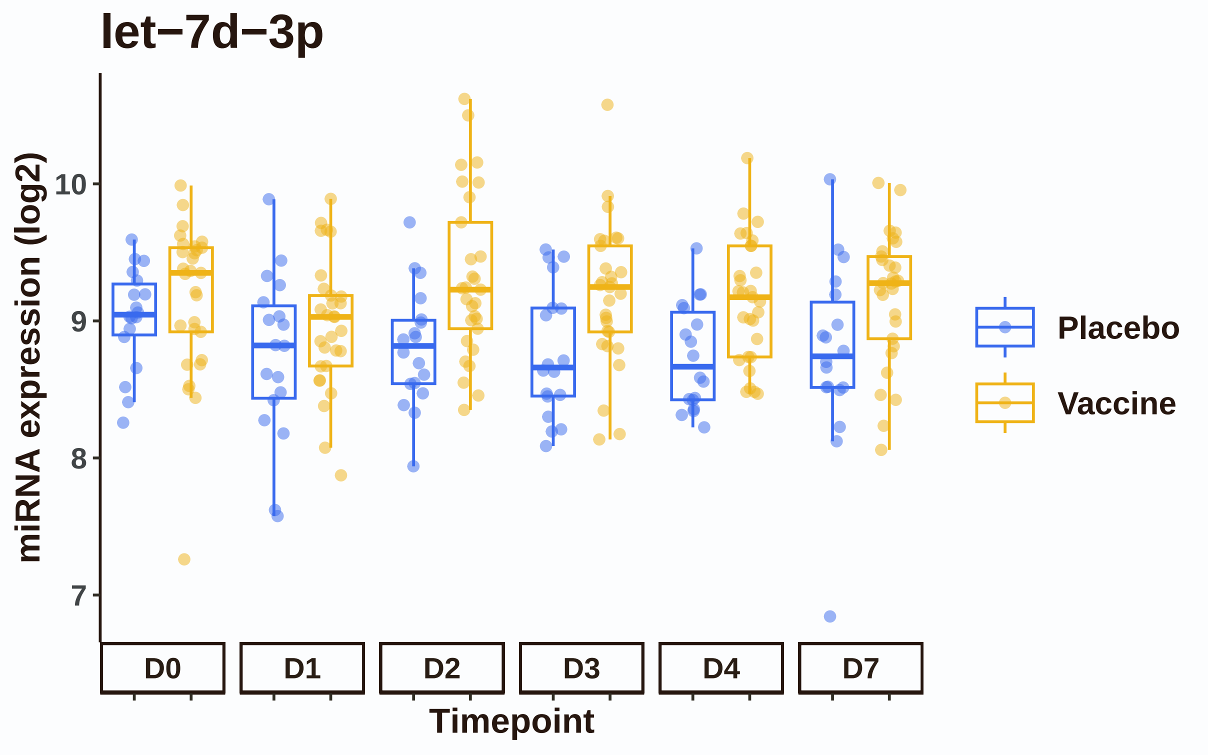
let−7d−3p
miRNA expression (log2)
10
9
8
7
D0
D1
D2
D3
D4
D7
Timepoint
Placebo
Vaccine

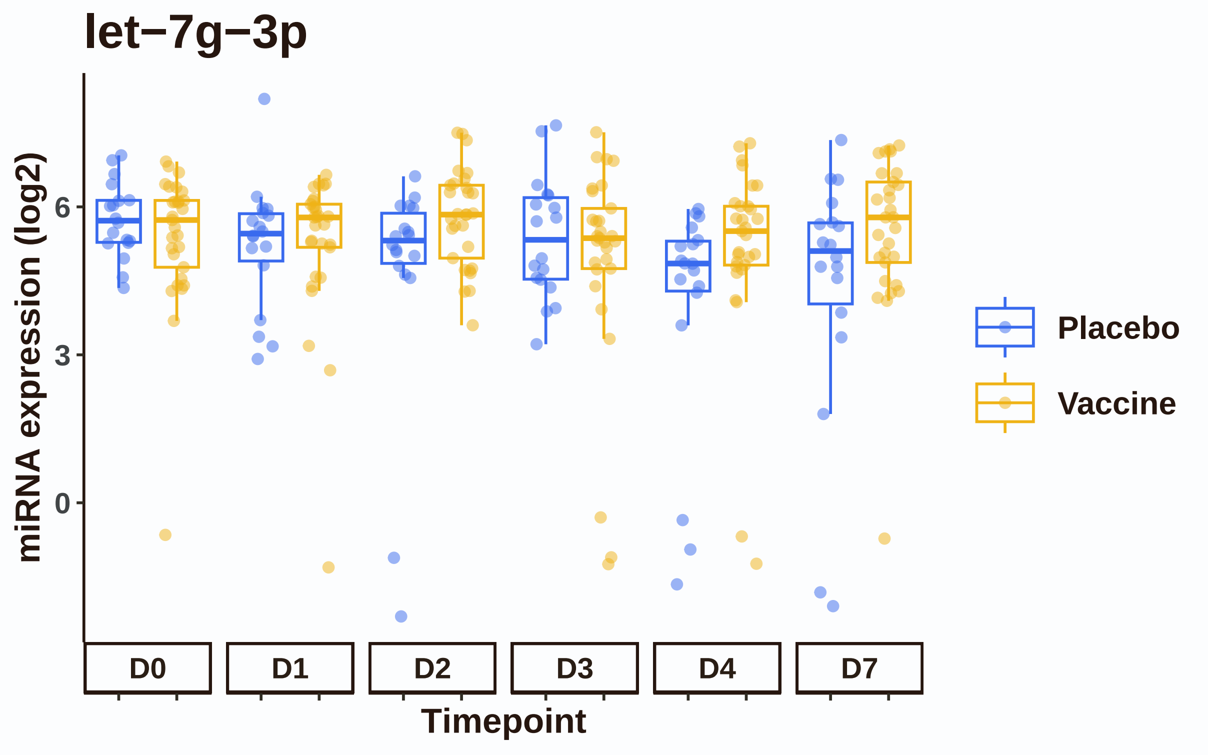

let−7g−3p
miRNA expression (log2)
6
3
0
D0
D1
D2
D3
D4
D7
Timepoint
Placebo
Vaccine

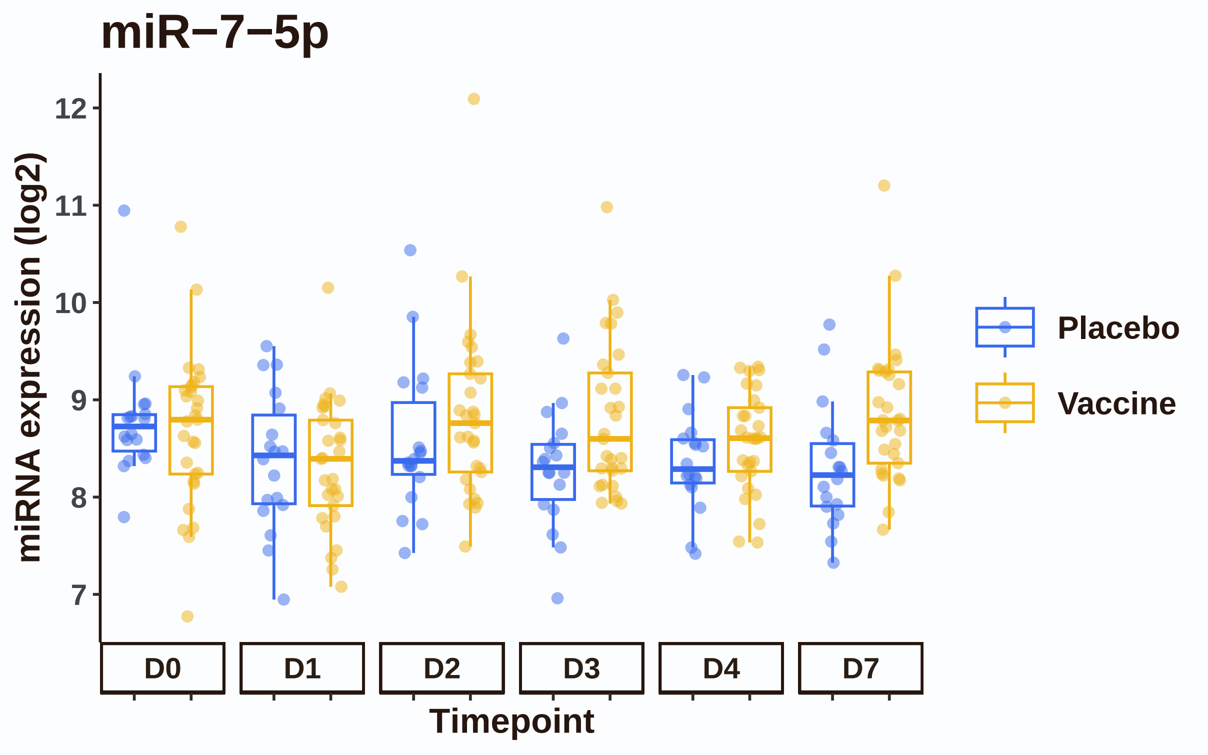
miR−7−5p
miRNA expression (log2)
12
11
10
9
8
7
D0
D1
D2
D3
D4
D7
Timepoint
Placebo
Vaccine

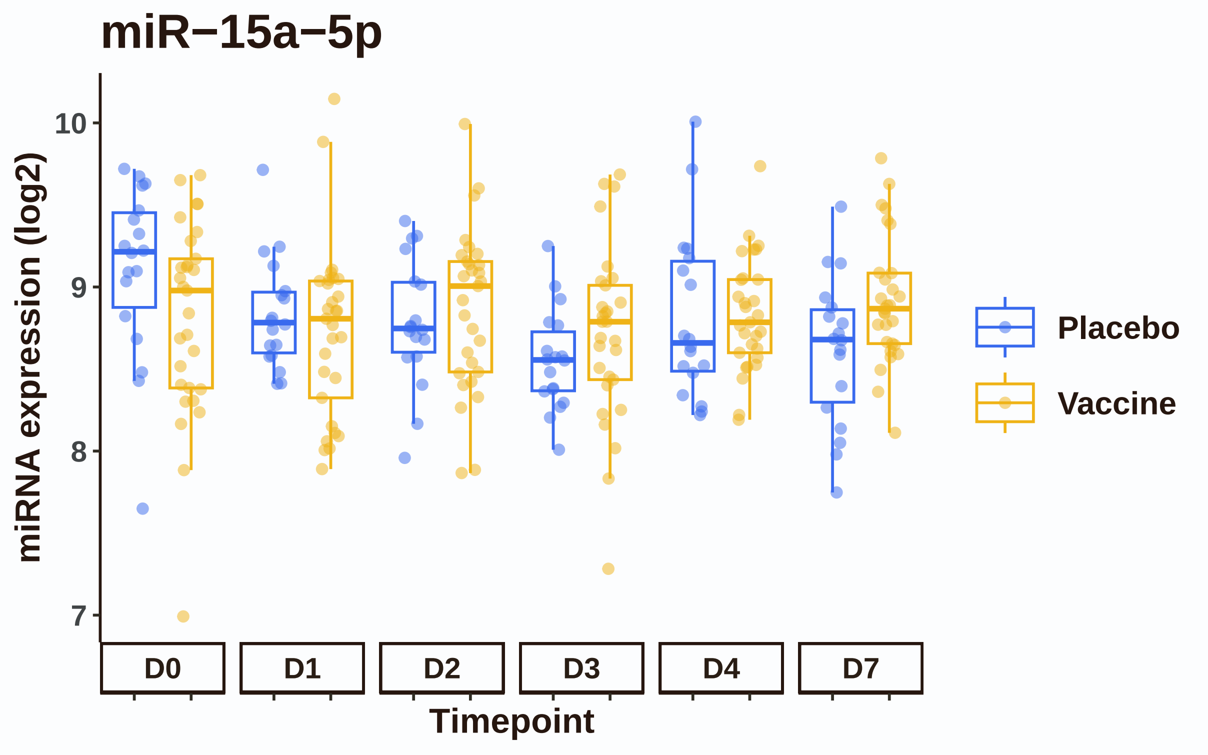

miR−15a−5p
miRNA expression (log2)
10
9
8
7
D0
D1
D2
D3
D4
D7
Timepoint
Placebo
Vaccine

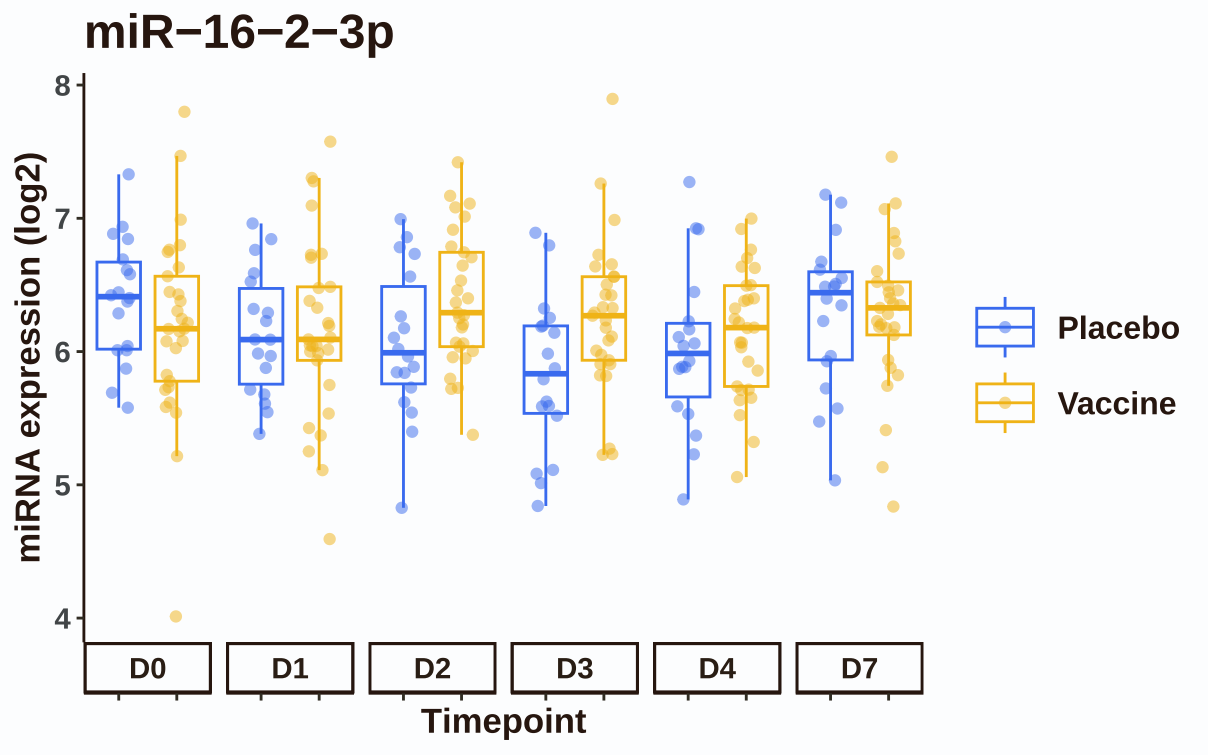

miR−16−2−3p
miRNA expression (log2)
8
7
6
5
4
D0
D1
D2
D3
D4
D7
Timepoint
Placebo
Vaccine

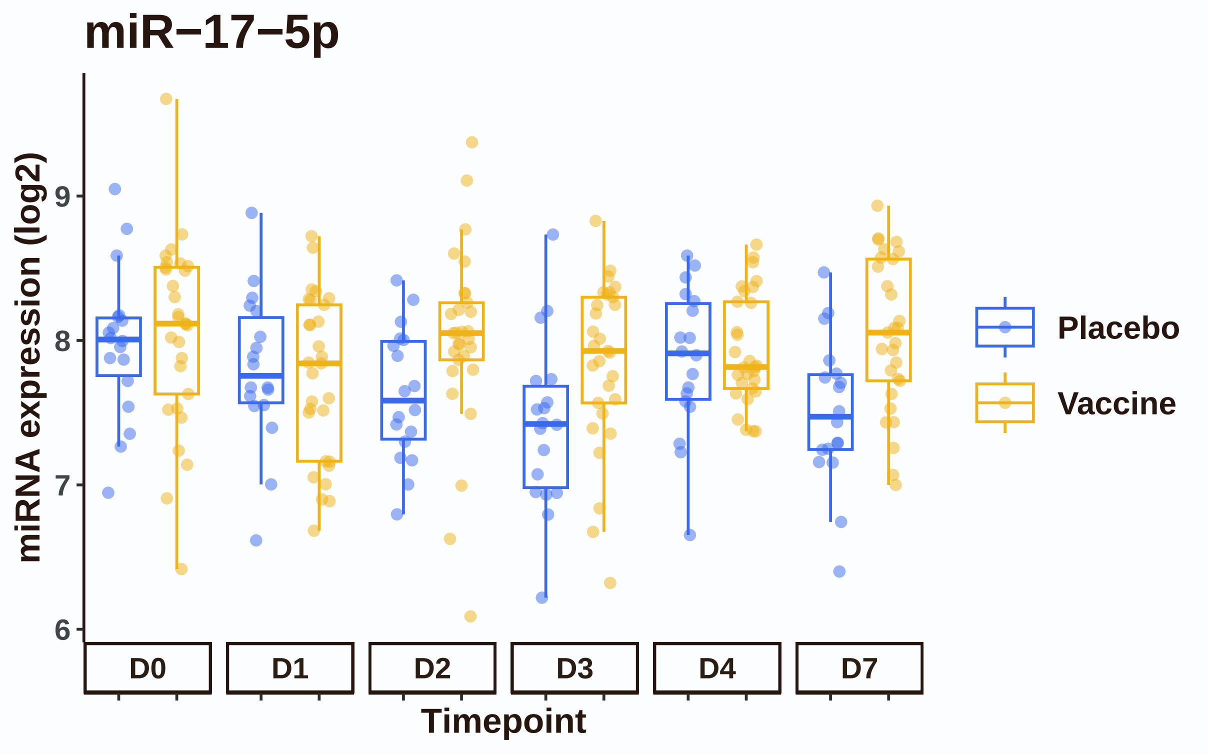

miR−17−5p
miRNA expression (log2)
9
8
7
6
D0
D1
D2
D3
D4
D7
Timepoint
Placebo
Vaccine

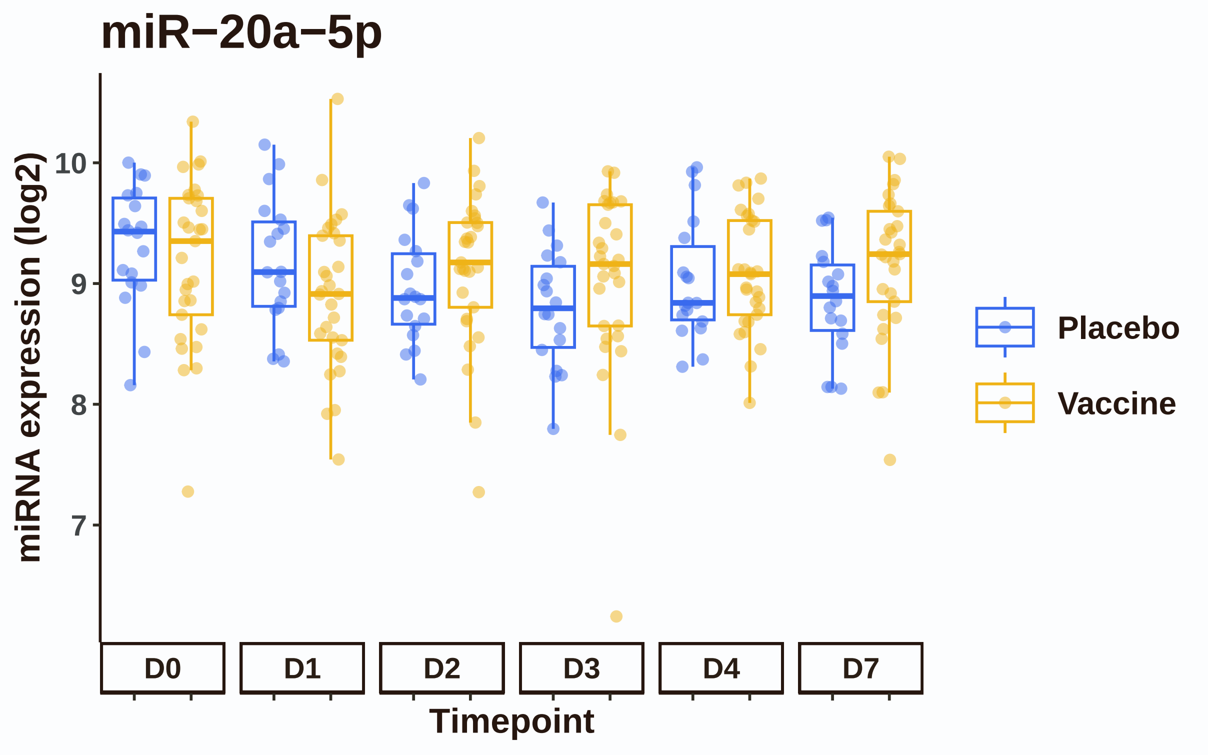
miR−20a−5p
miRNA expression (log2)
10
9
8
7
D0
D1
D2
D3
D4
D7
Timepoint
Placebo
Vaccine

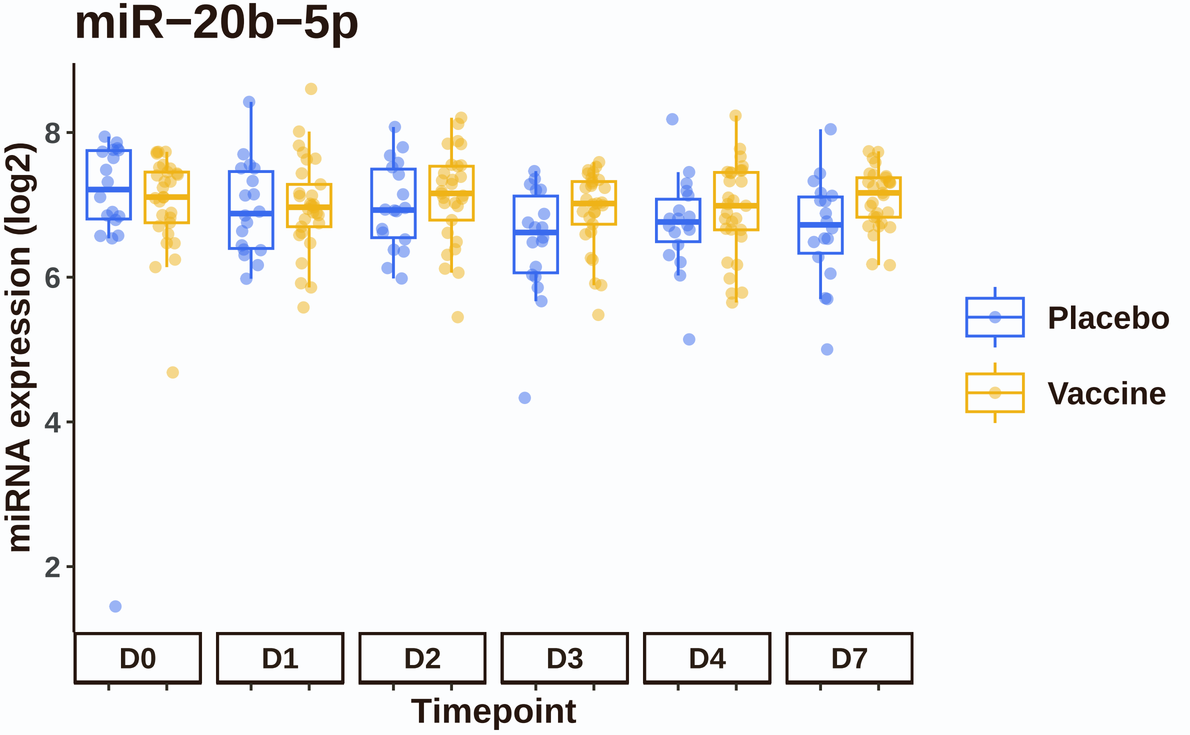

miR−20b−5p
miRNA expression (log2)
2
4
6
8
D0
D1
D2
D3
D4
D7
Timepoint
Placebo
Vaccine

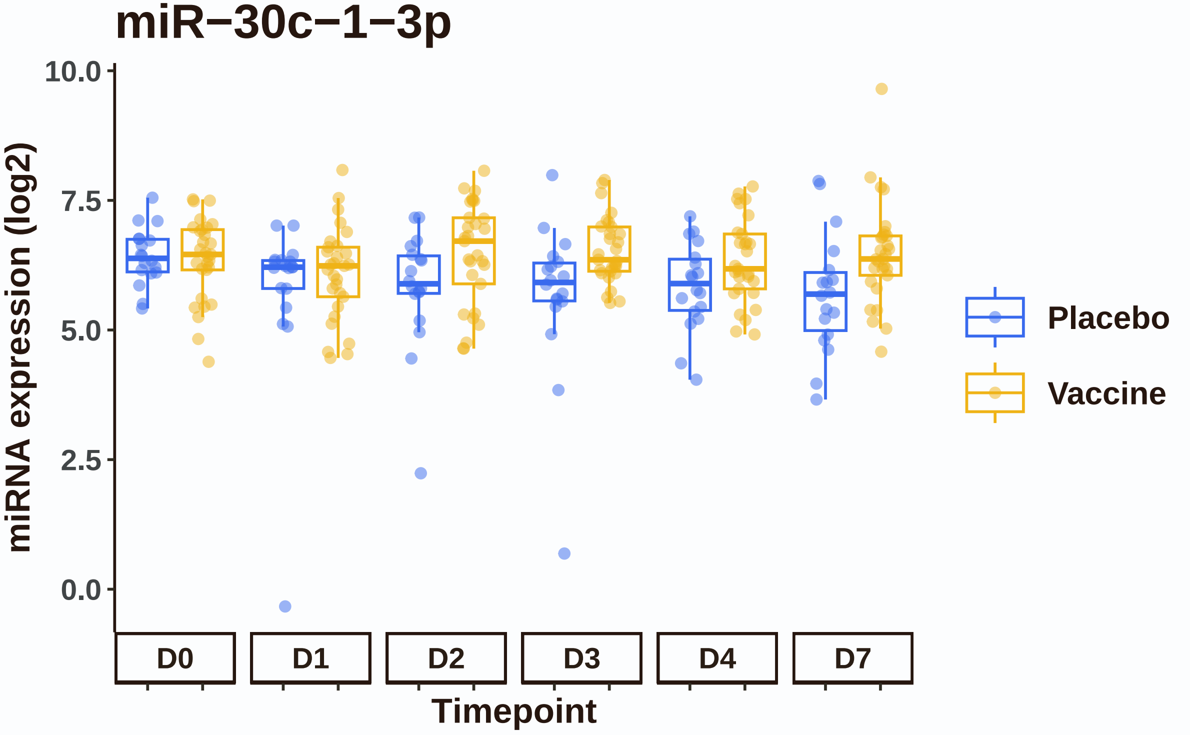

miR−30c−1−3p
miRNA expression (log2)
10.0
7.5
5.0
2.5
0.0
D0
D1
D2
D3
D4
D7
Timepoint
Placebo
Vaccine

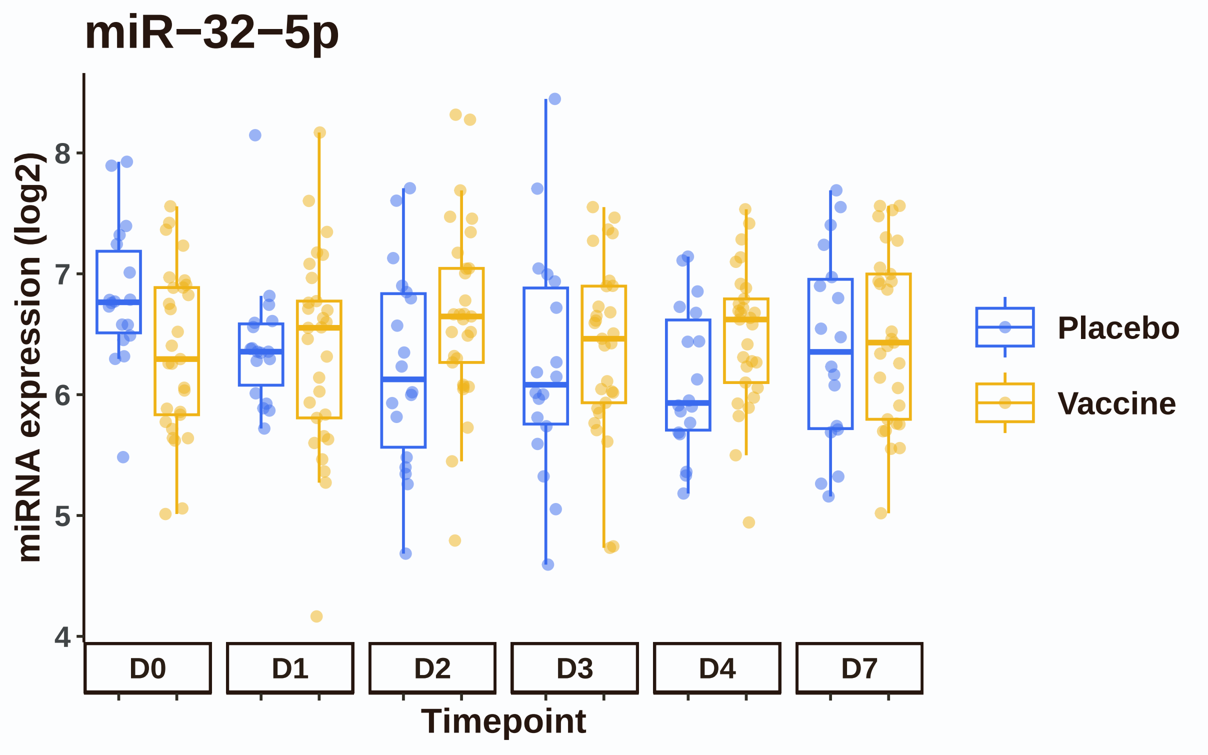

miR−32−5p
miRNA expression (log2)
8
7
6
5
4
D0
D1
D2
D3
D4
D7
Timepoint
Placebo
Vaccine

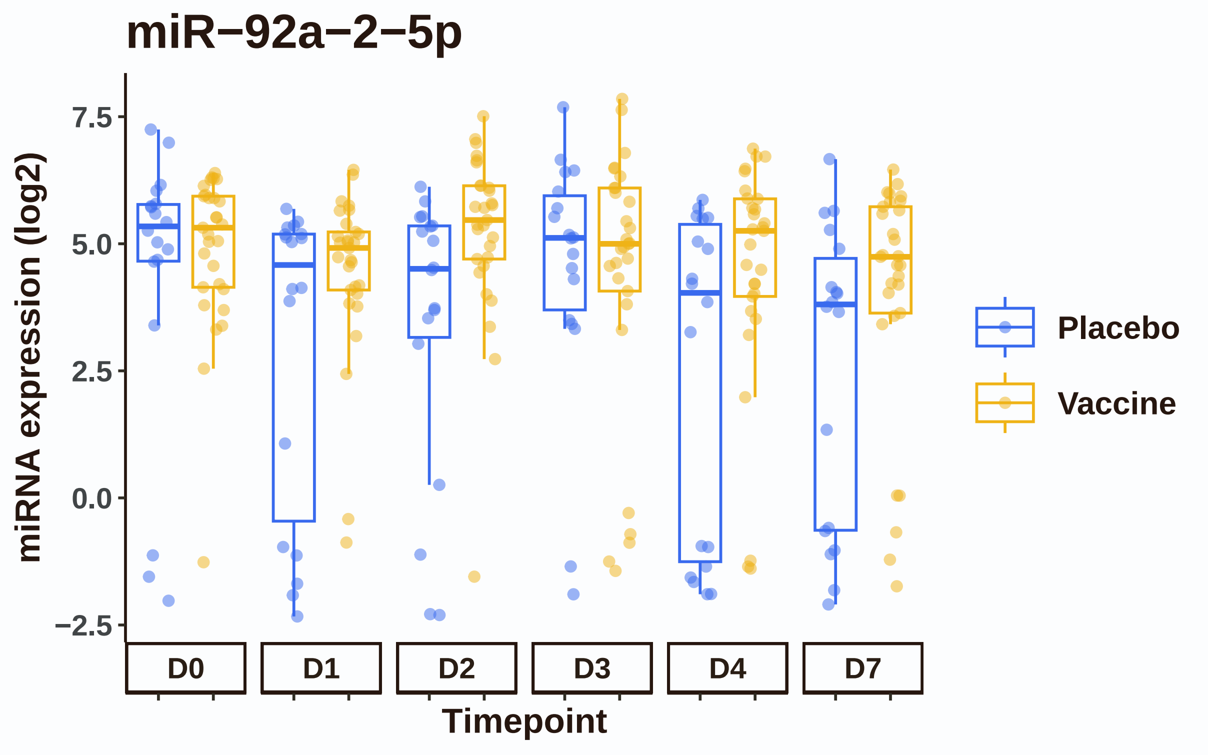
miR−92a−2−5p
miRNA expression (log2)
7.5
5.0
2.5
0.0
−2.5
D0
D1
D2
D3
D4
D7
Timepoint
Placebo
Vaccine

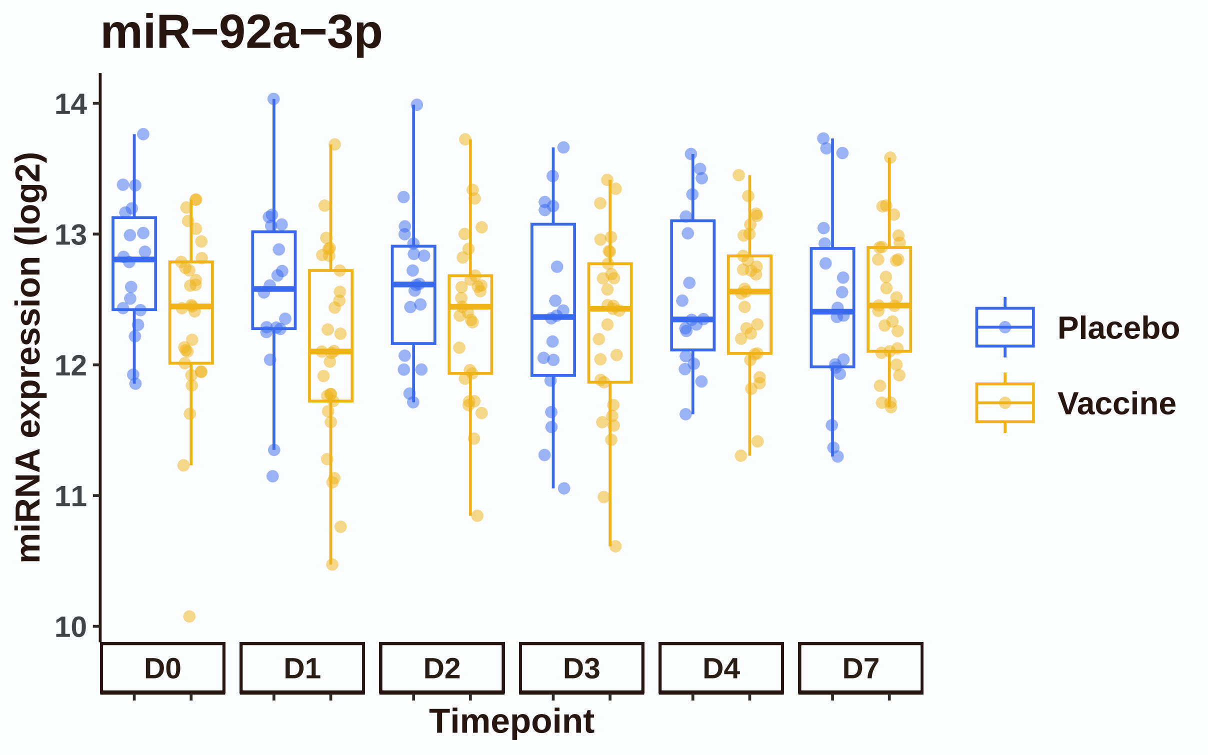

miR−92a−3p
miRNA expression (log2)
14
13
12
11
10
D0
D1
D2
D3
D4
D7
Timepoint
Placebo
Vaccine

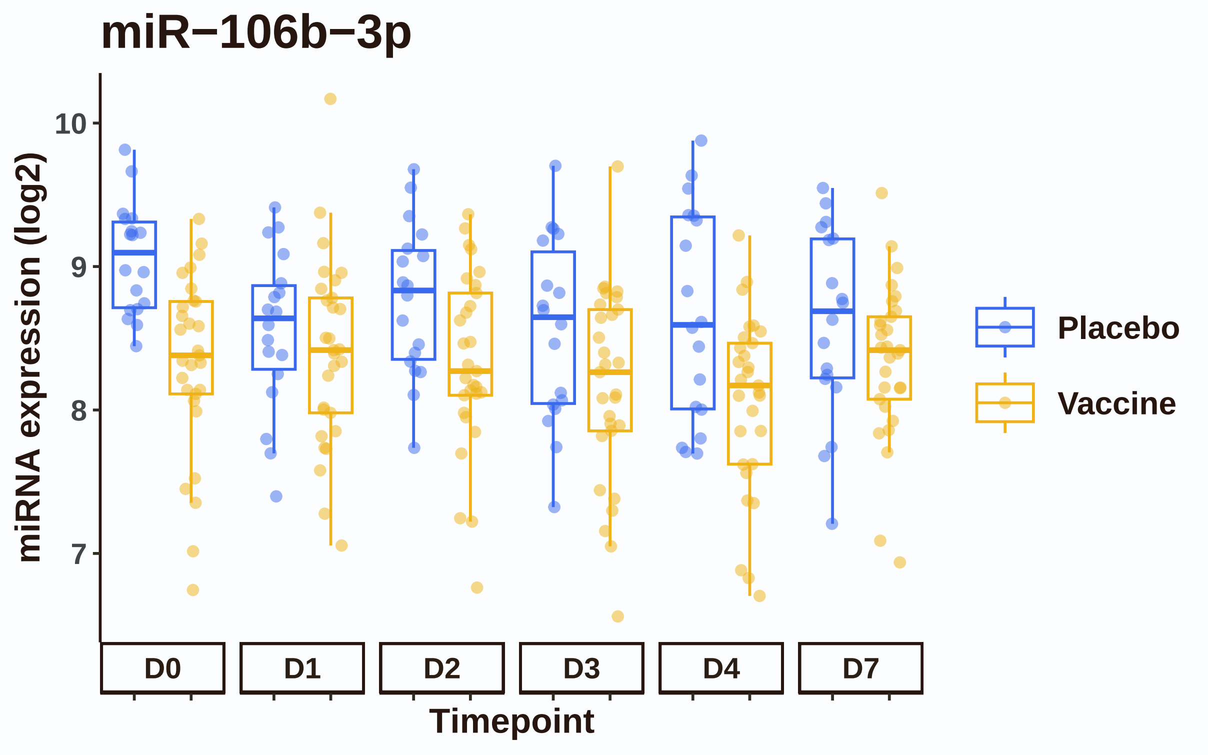
miR−106b−3p
miRNA expression (log2)
10
9
8
7
D0
D1
D2
D3
D4
D7
Timepoint
Placebo
Vaccine

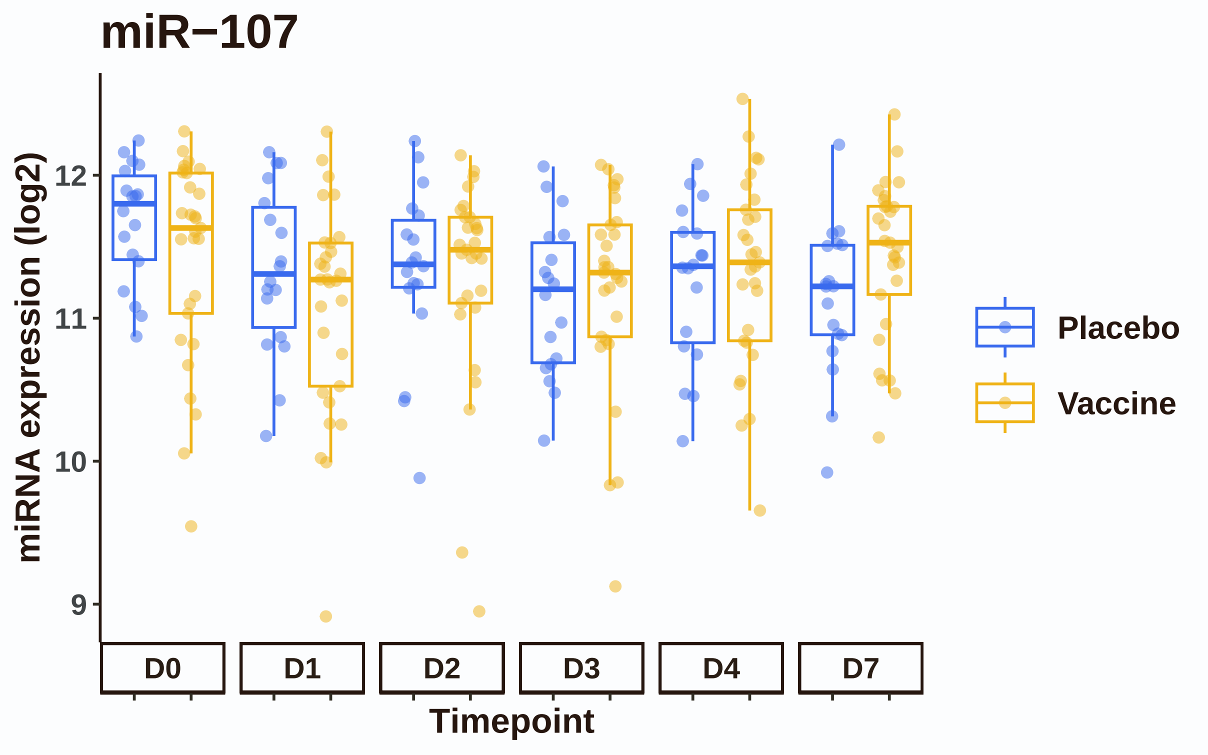

miR−107
miRNA expression (log2)
12
11
10
9
D0
D1
D2
D3
D4
D7
Timepoint
Placebo
Vaccine

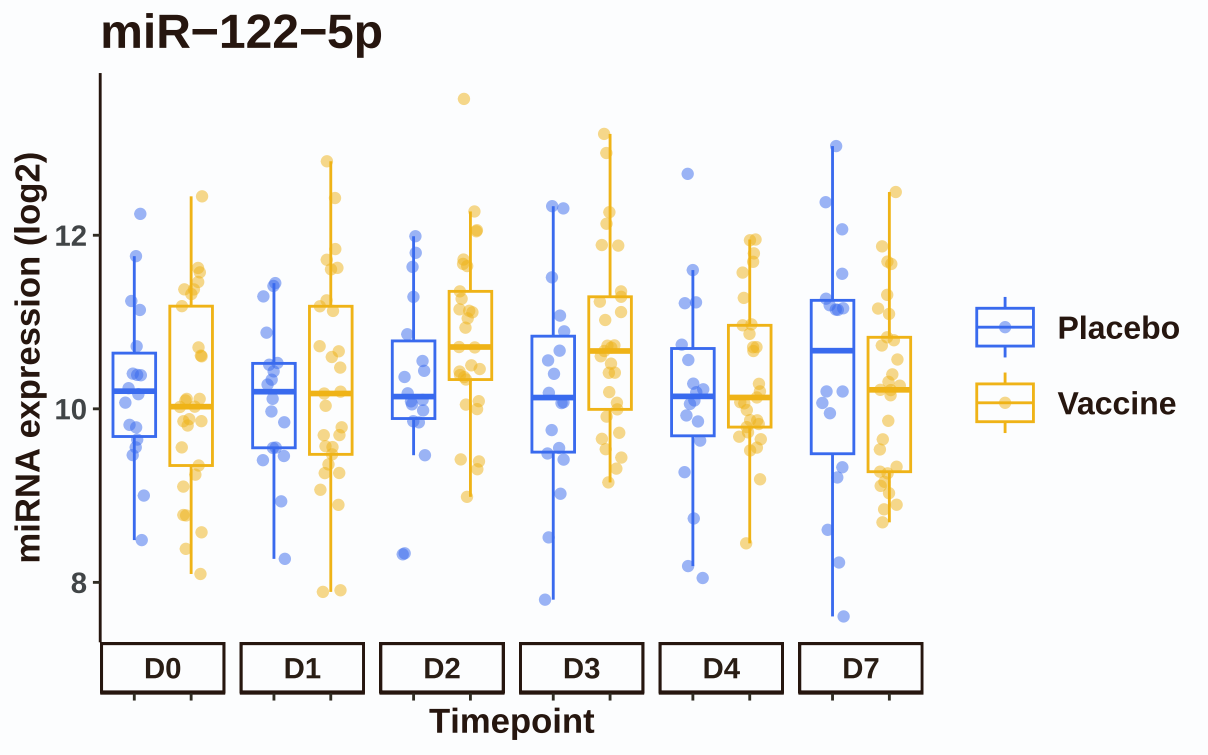

miR−122−5p
miRNA expression (log2)
12
10
8
D0
D1
D2
D3
D4
D7
Timepoint
Placebo
Vaccine

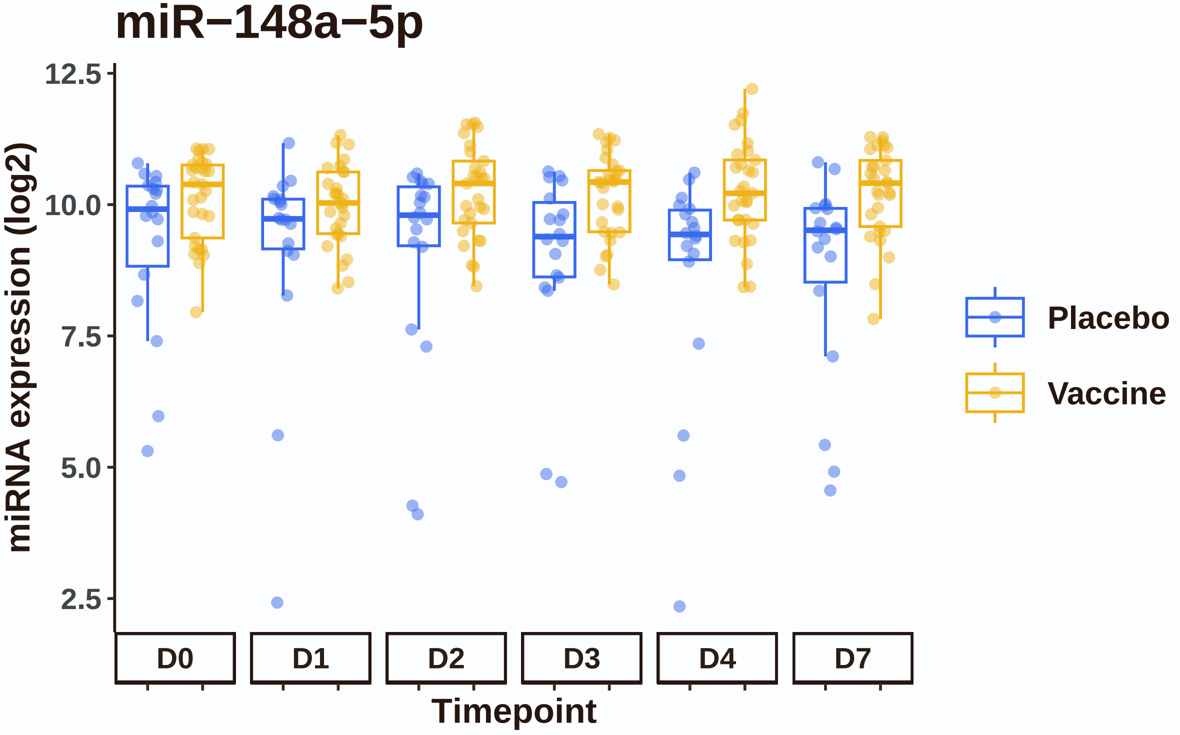
miR−148a−5p
miRNA expression (log2)
12.5
10.0
7.5
5.0
2.5
D0
D1
D2
D3
D4
D7
Timepoint
Placebo
Vaccine

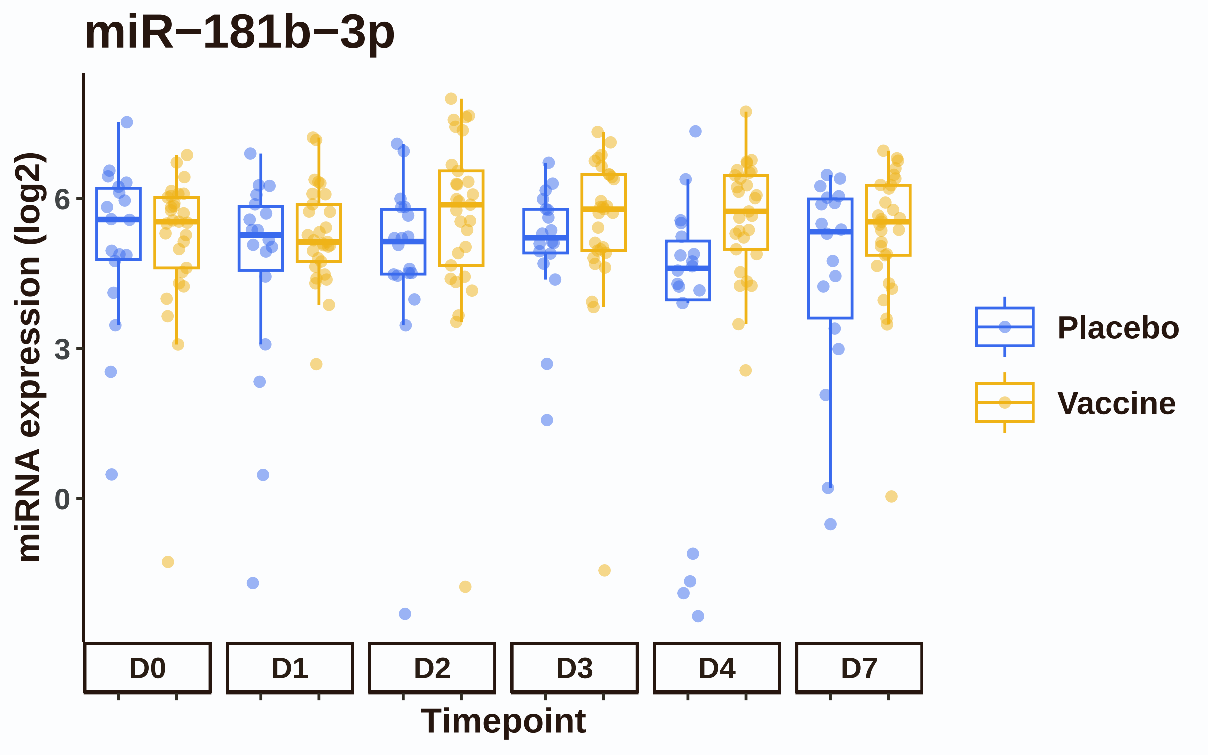

miR−181b−3p
miRNA expression (log2)
6
3
0
D0
D1
D2
D3
D4
D7
Timepoint
Placebo
Vaccine

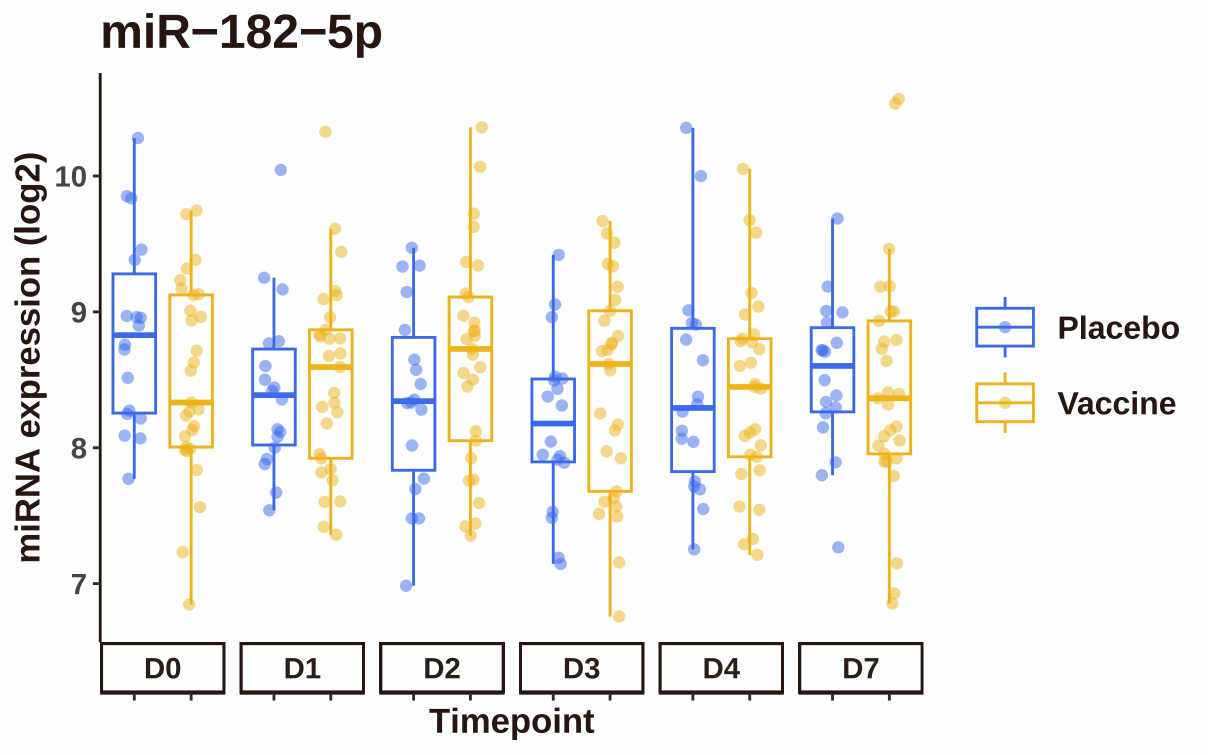

miR−182−5p
miRNA expression (log2)
10
9
8
7
D0
D1
D2
D3
D4
D7
Timepoint
Placebo
Vaccine

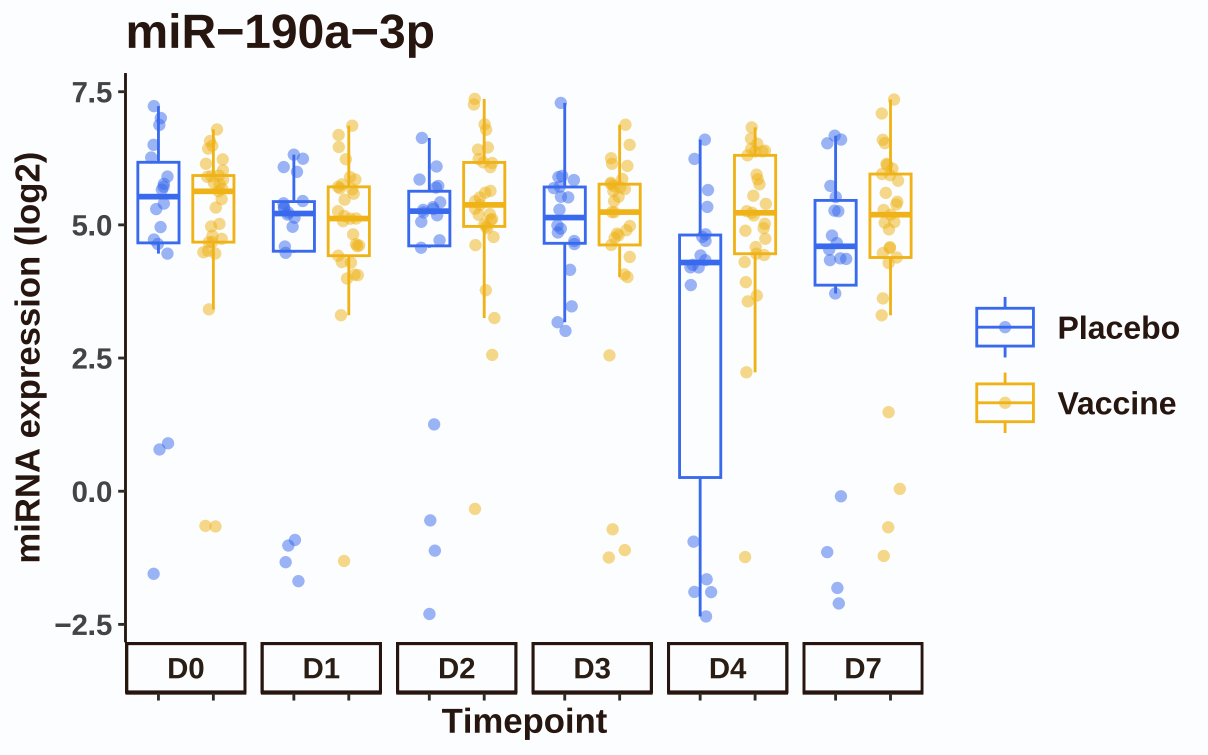

miR−190a−3p
miRNA expression (log2)
7.5
5.0
2.5
0.0
−2.5
D0
D1
D2
D3
D4
D7
Timepoint
Placebo
Vaccine

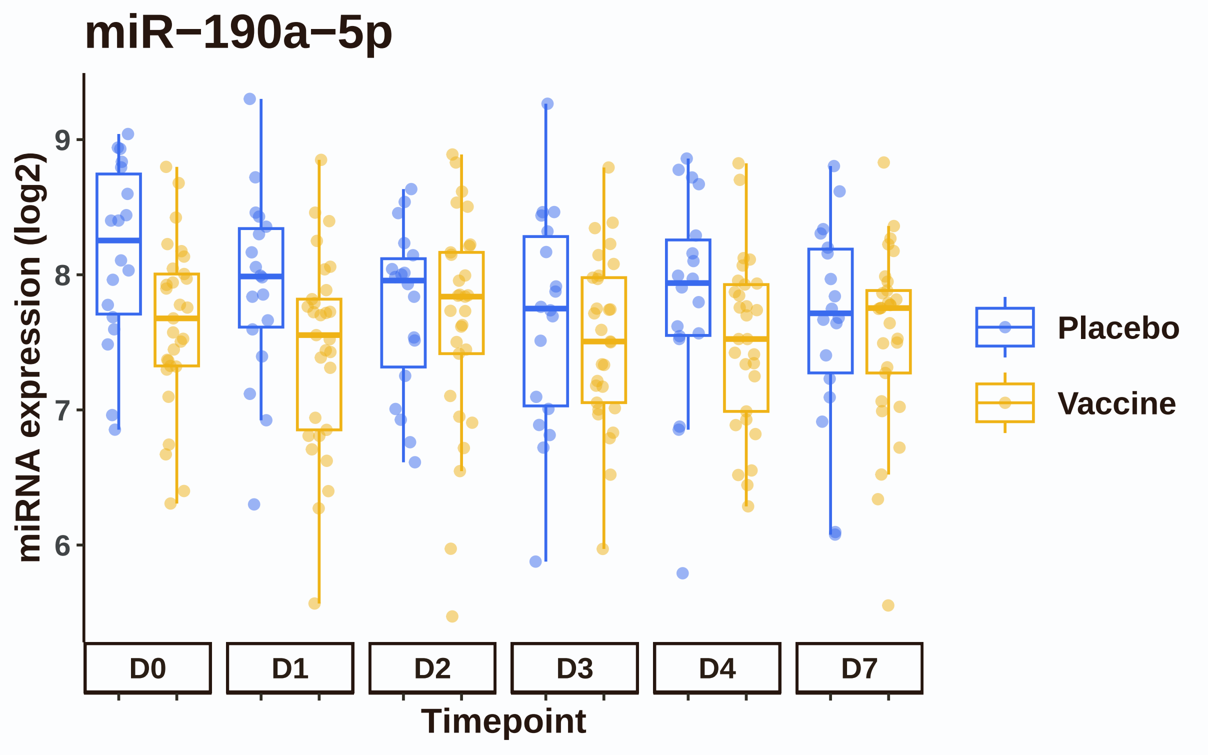

miR−190a−5p
miRNA expression (log2)
9
8
7
6
D0
D1
D2
D3
D4
D7
Timepoint
Placebo
Vaccine

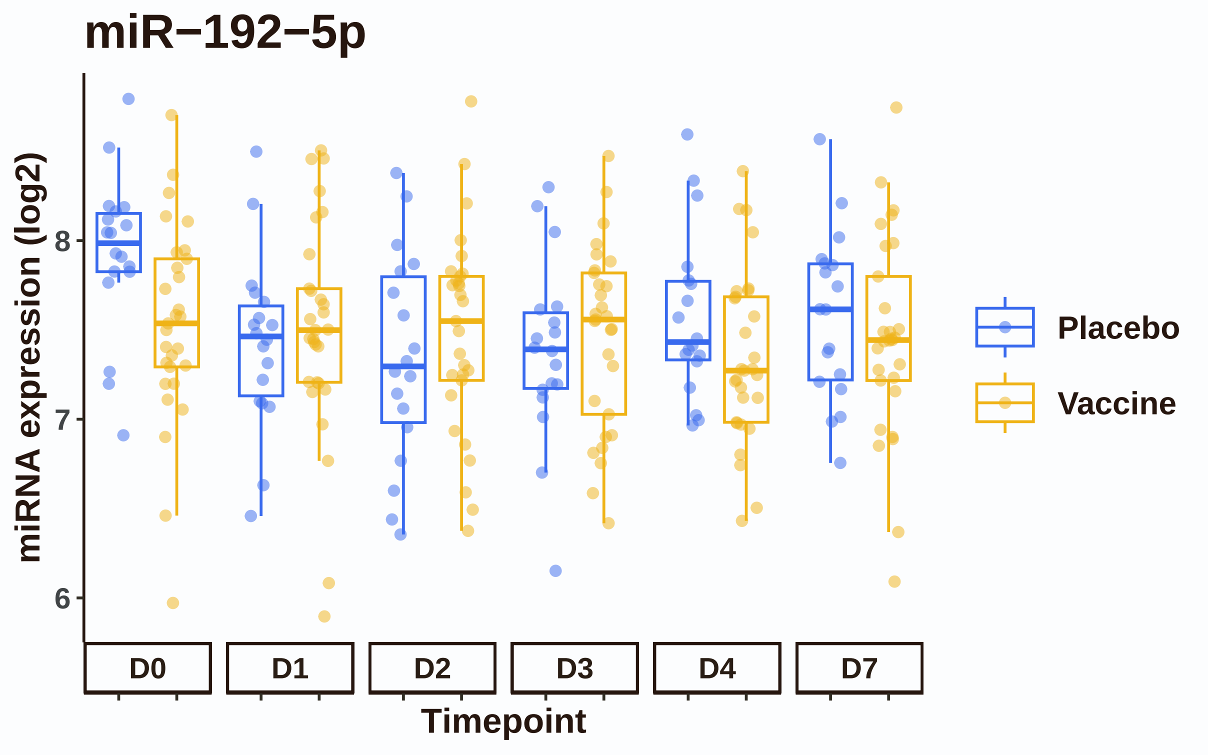

miR−192−5p
miRNA expression (log2)
8
7
6
D0
D1
D2
D3
D4
D7
Timepoint
Placebo
Vaccine

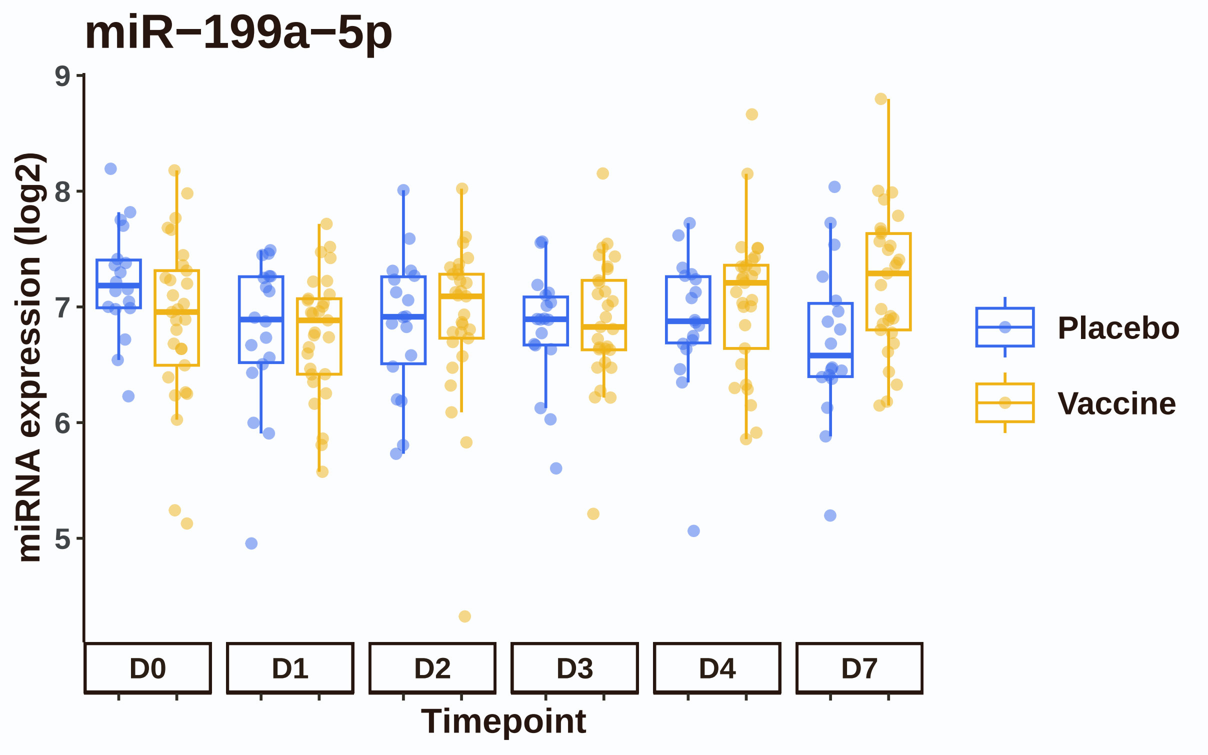
miR−199a−5p
miRNA expression (log2)
9
8
7
6
5
D0
D1
D2
D3
D4
D7
Timepoint
Placebo
Vaccine

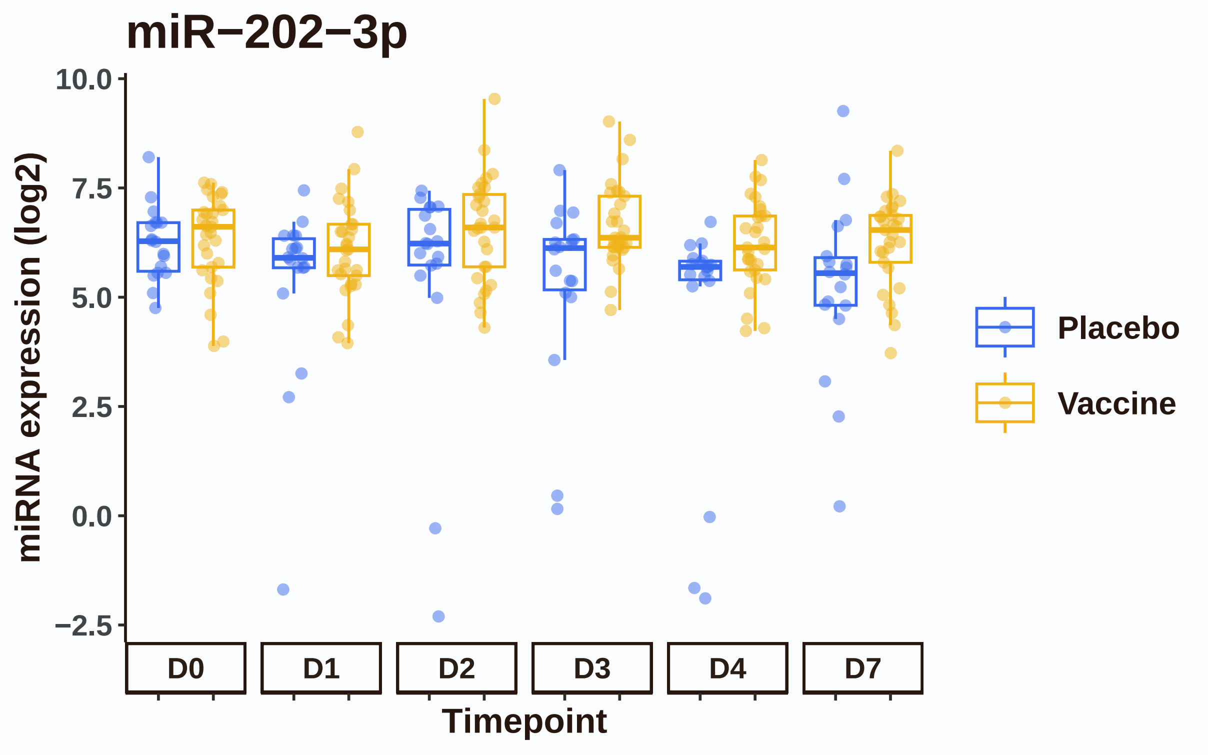
miR−202−3p
miRNA expression (log2)
10.0
7.5
5.0
2.5
0.0
−2.5
D0
D1
D2
D3
D4
D7
Timepoint
Placebo
Vaccine

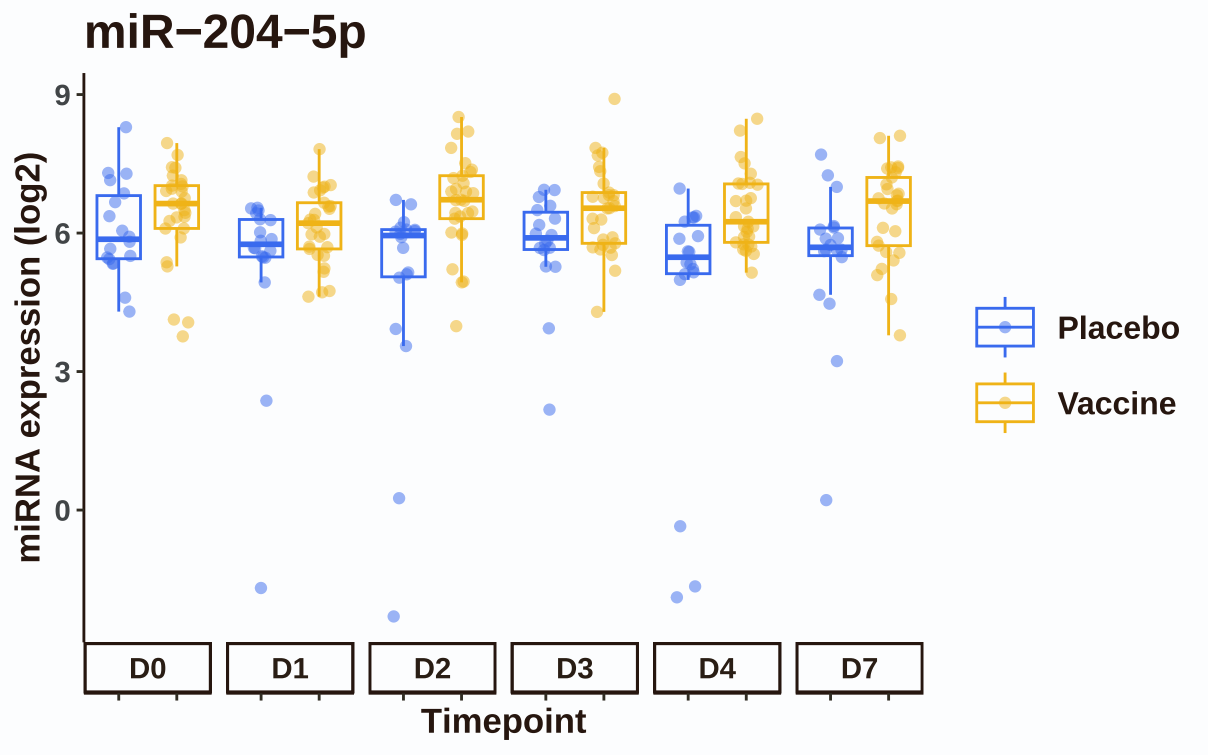
miR−204−5p
miRNA expression (log2)
9
6
3
0
D0
D1
D2
D3
D4
D7
Timepoint
Placebo
Vaccine

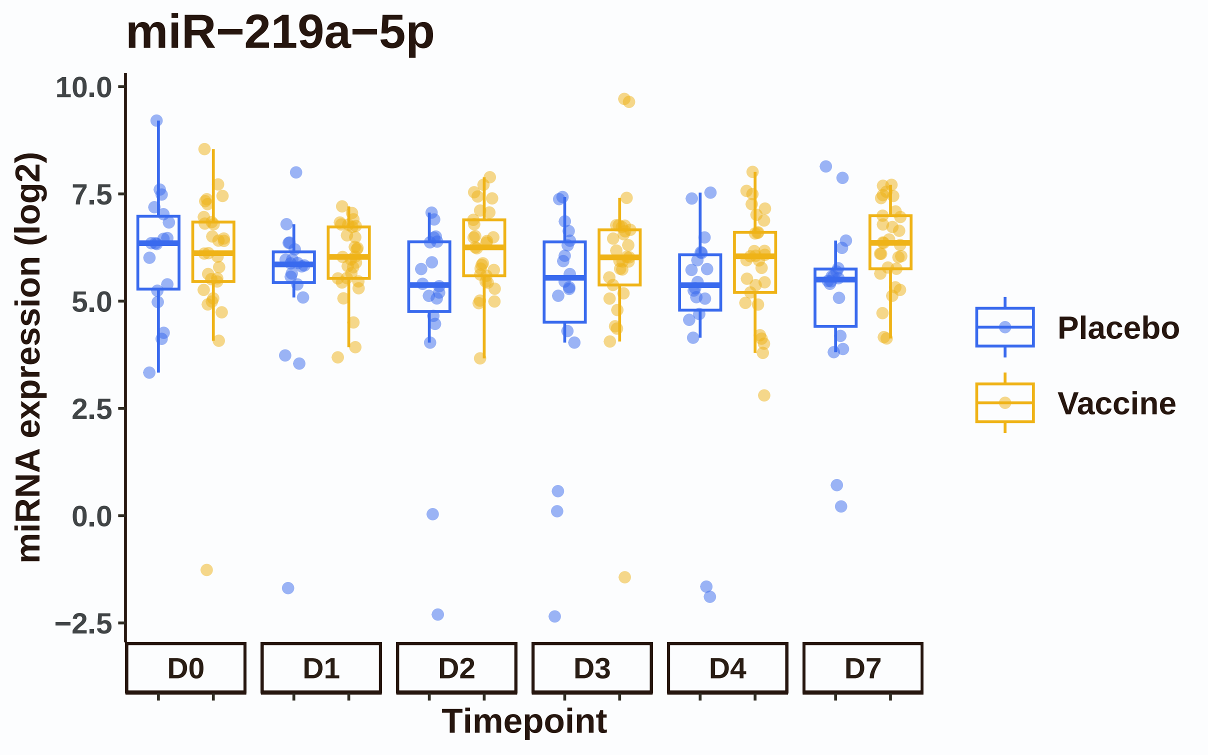
miR−219a−5p
miRNA expression (log2)
10.0
7.5
5.0
2.5
0.0
−2.5
D0
D1
D2
D3
D4
D7
Timepoint
Placebo
Vaccine

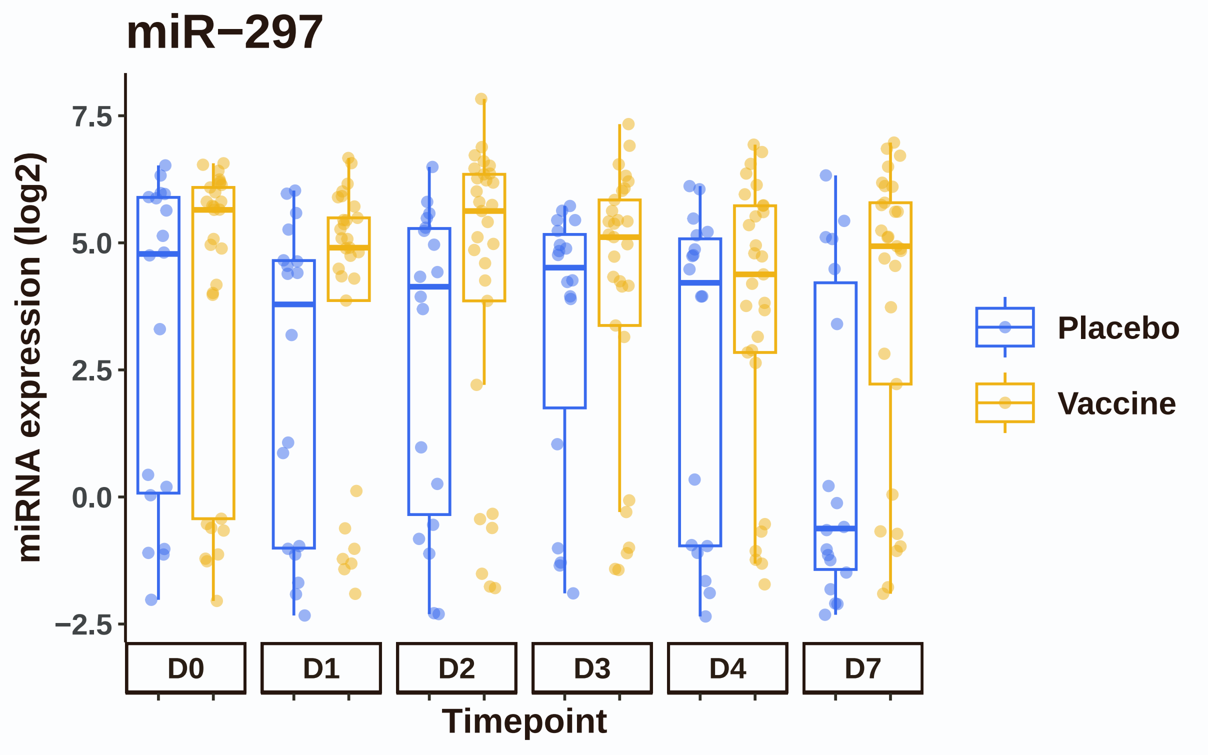

miR−297
miRNA expression (log2)
7.5
5.0
2.5
0.0
−2.5
D0
D1
D2
D3
D4
D7
Timepoint
Placebo
Vaccine

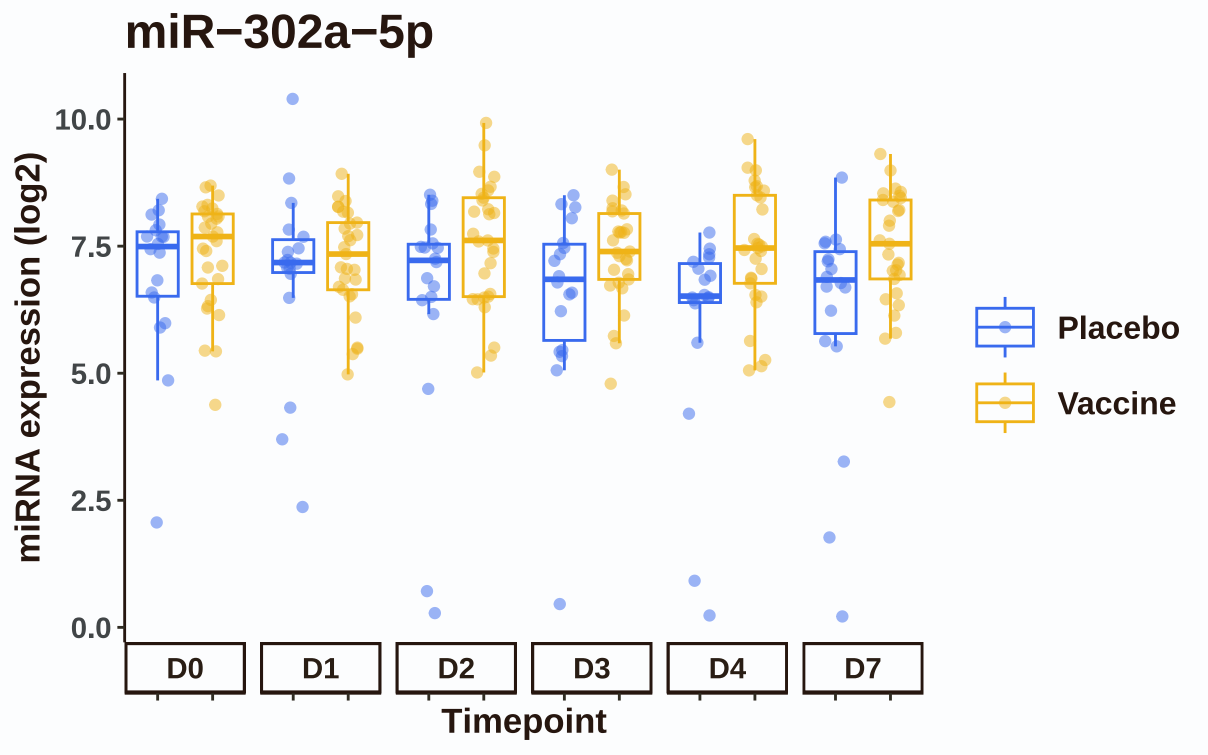

miR−302a−5p
miRNA expression (log2)
10.0
7.5
5.0
2.5
0.0
D0
D1
D2
D3
D4
D7
Timepoint
Placebo
Vaccine

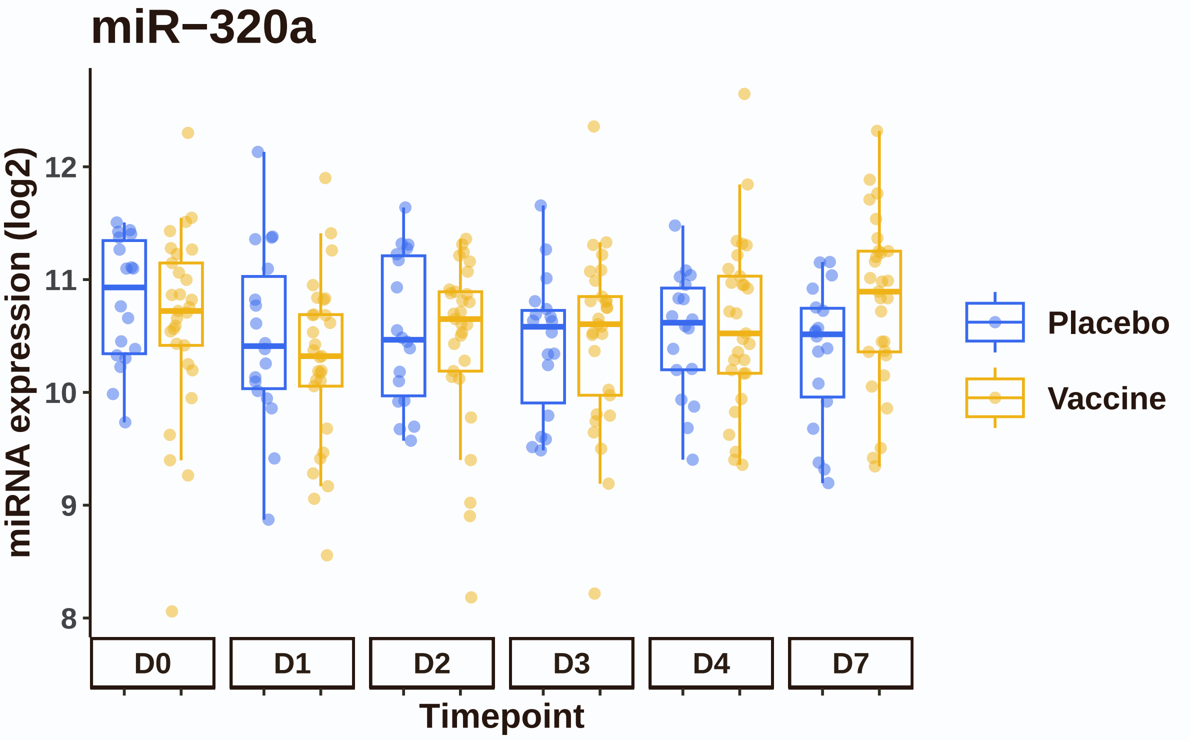

miR−320a
miRNA expression (log2)
12
11
10
9
8
D0
D1
D2
D3
D4
D7
Timepoint
Placebo
Vaccine

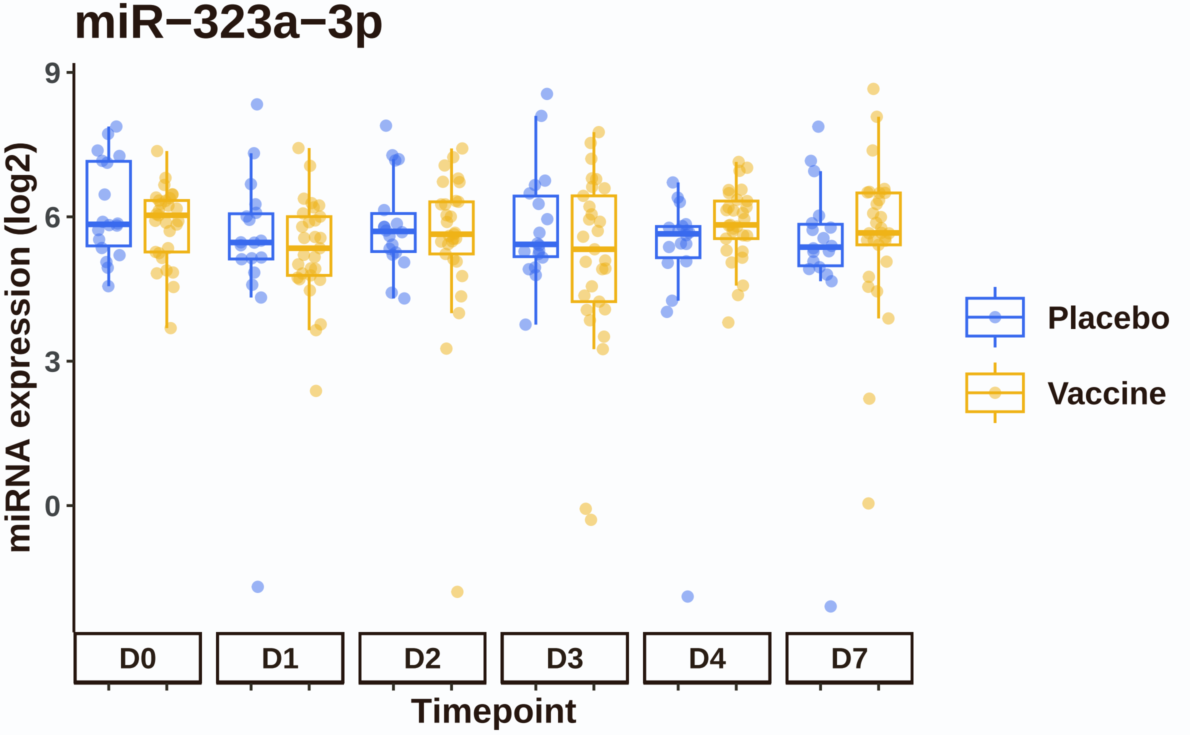
miR−323a−3p
miRNA expression (log2)
9
6
3
0
D0
D1
D2
D3
D4
D7
Timepoint
Placebo
Vaccine

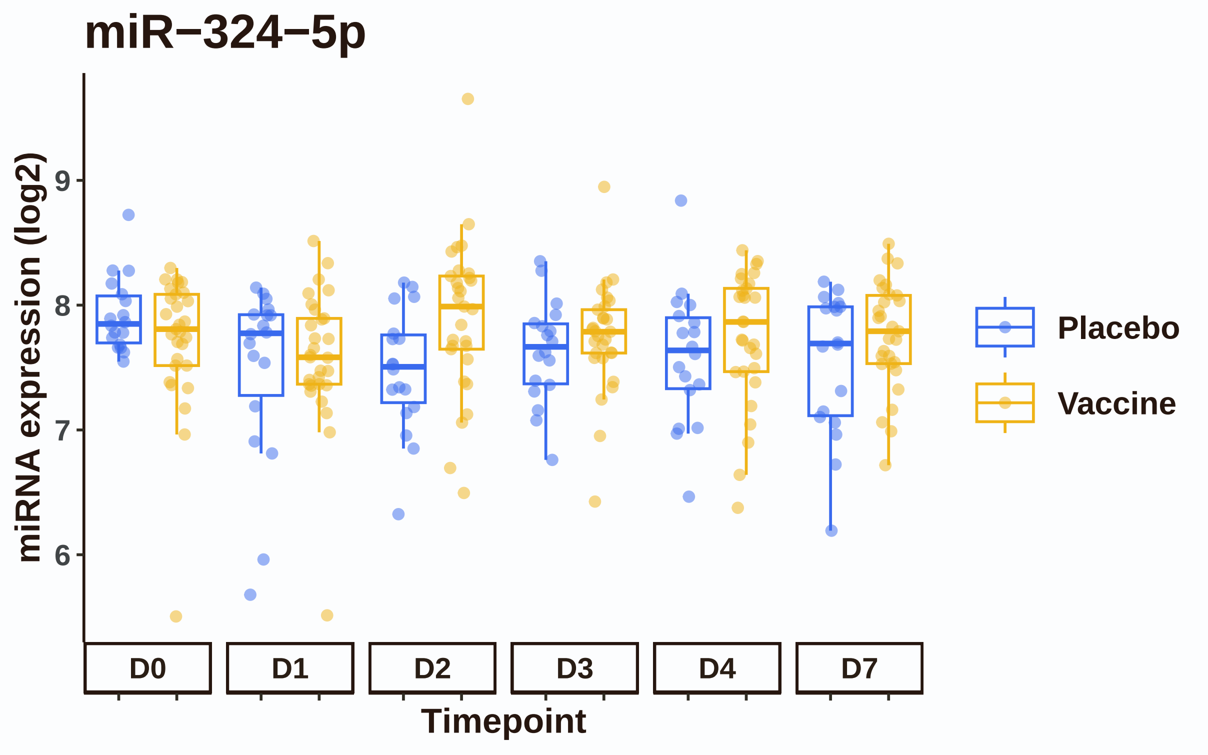
miR−324−5p
miRNA expression (log2)
9
8
7
6
D0
D1
D2
D3
D4
D7
Timepoint
Placebo
Vaccine

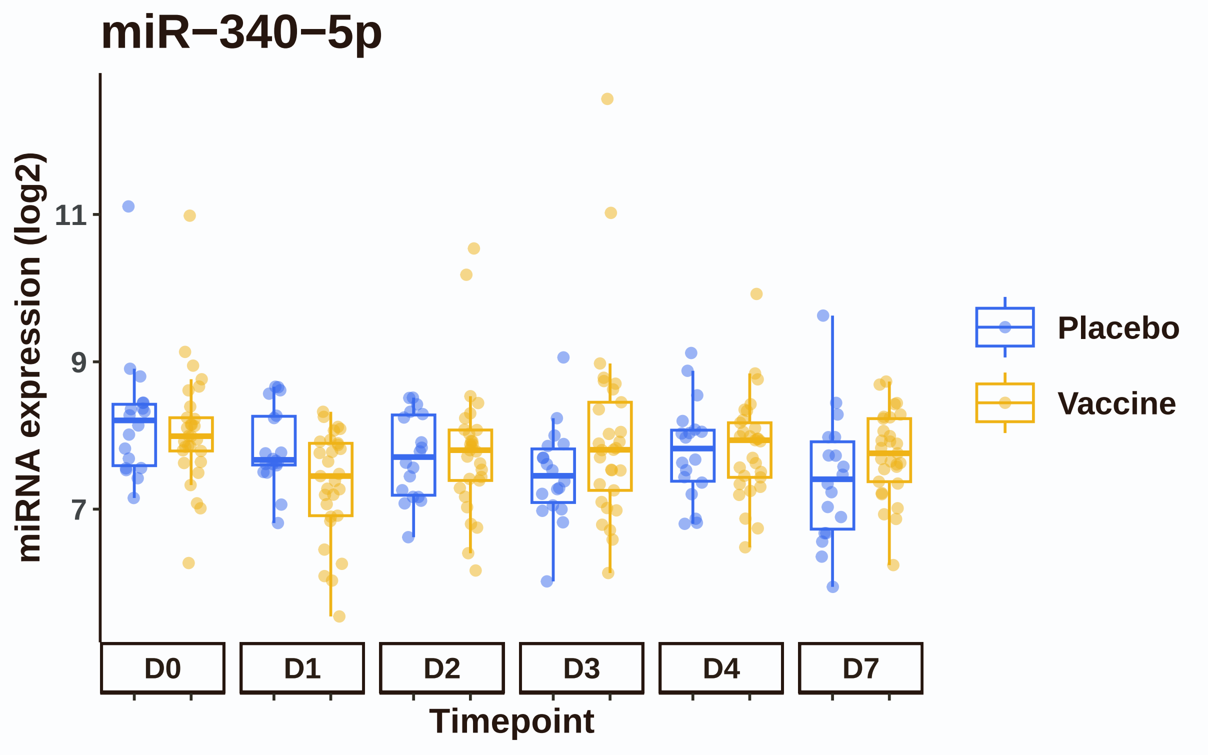

miR−340−5p
miRNA expression (log2)
11
9
7
D0
D1
D2
D3
D4
D7
Timepoint
Placebo
Vaccine

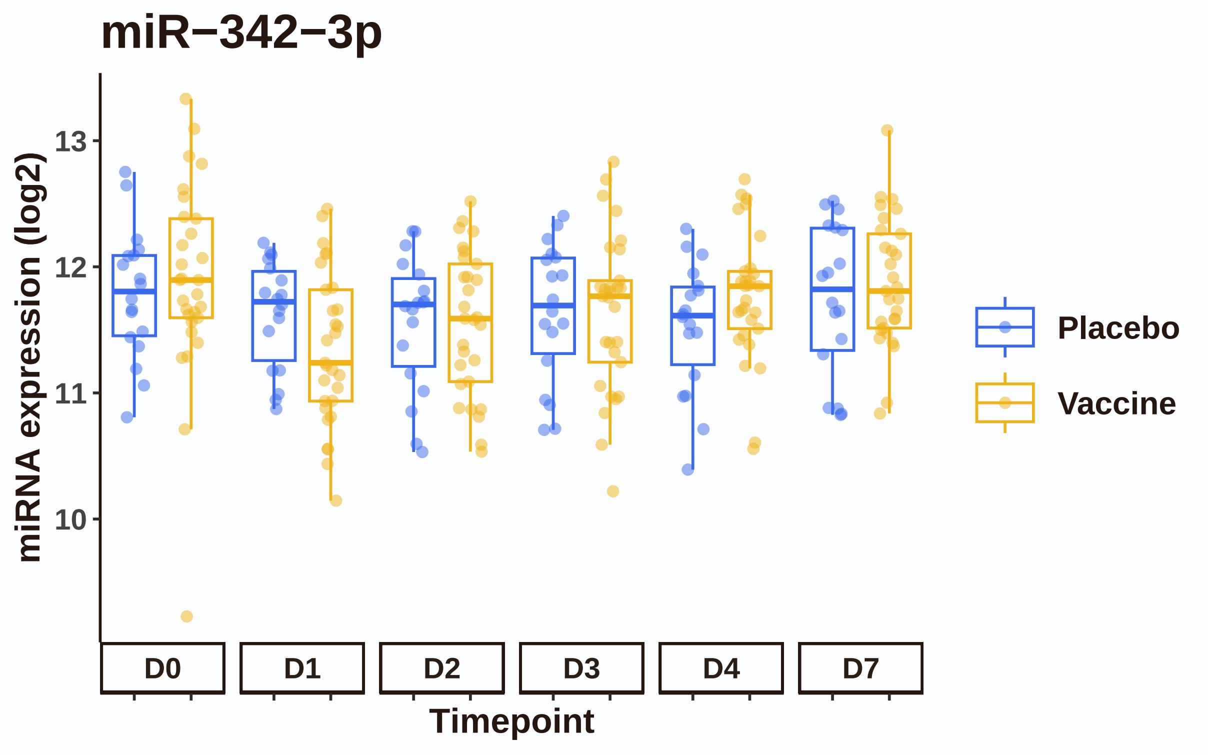

miR−342−3p
miRNA expression (log2)
13
12
11
10
D0
D1
D2
D3
D4
D7
Timepoint
Placebo
Vaccine

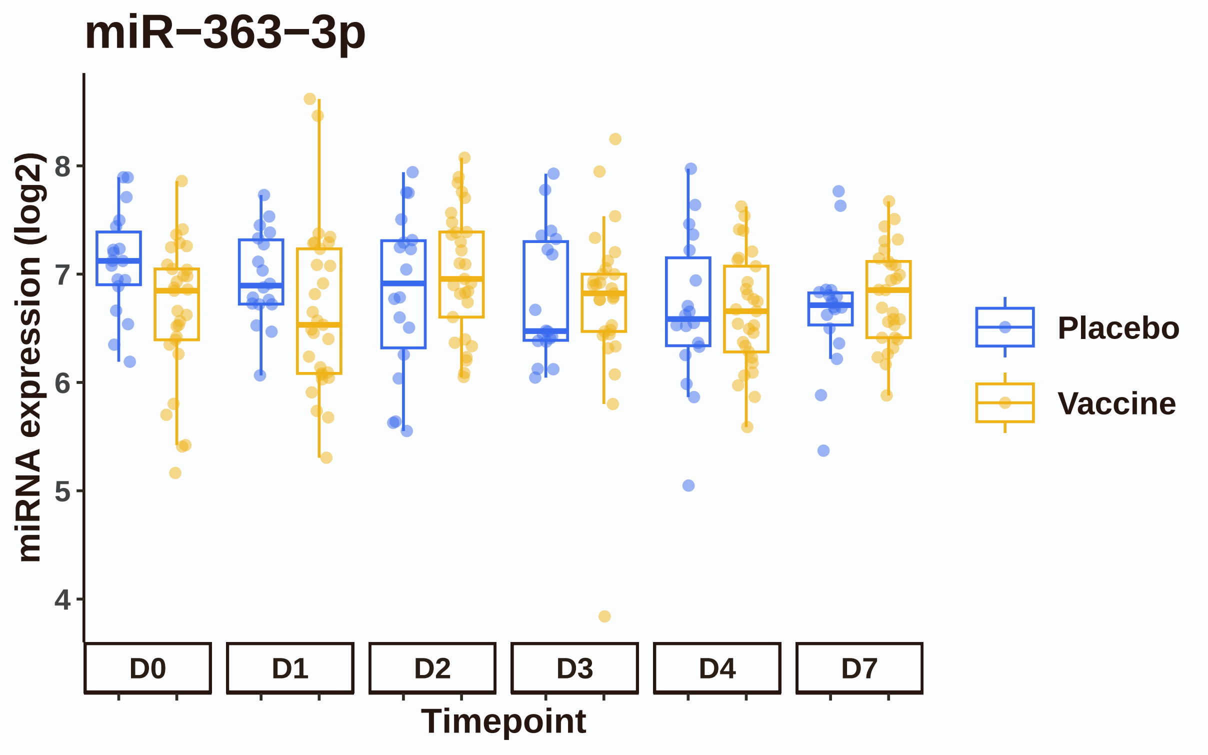
miR−363−3p
miRNA expression (log2)
8
7
6
5
4
D0
D1
D2
D3
D4
D7
Timepoint
Placebo
Vaccine

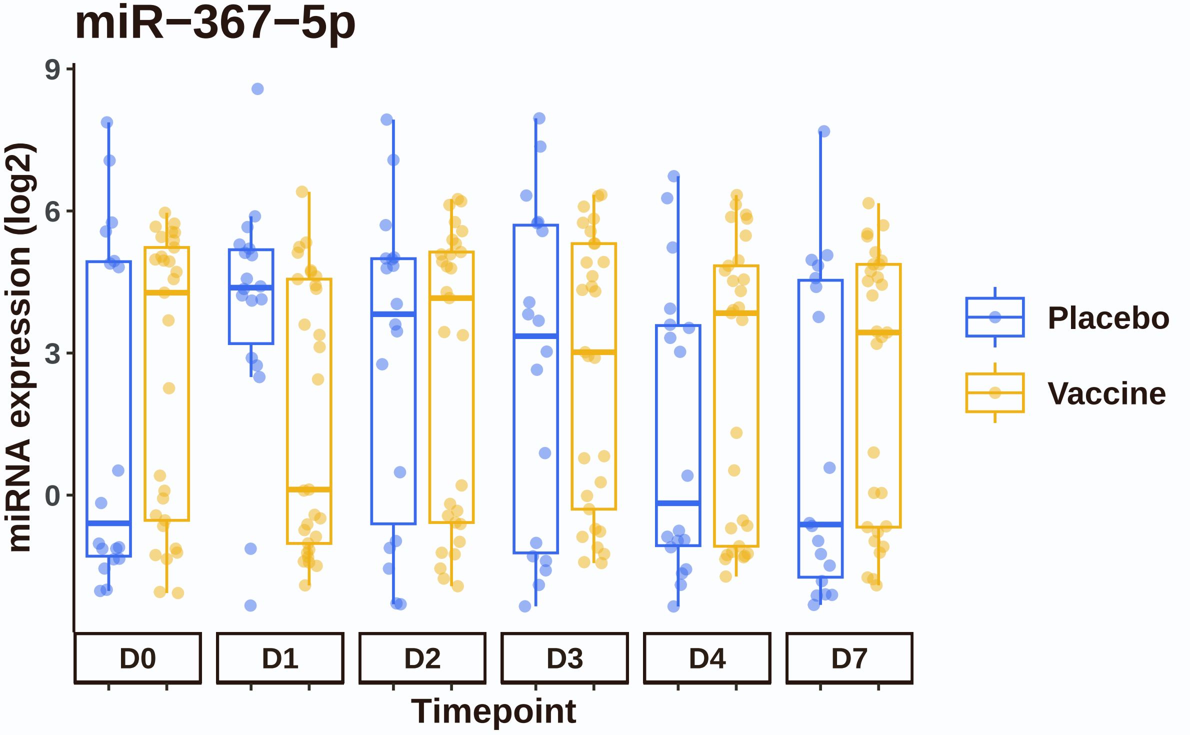
miR−367−5p
miRNA expression (log2)
9
6
3
0
D0
D1
D2
D3
D4
D7
Timepoint
Placebo
Vaccine

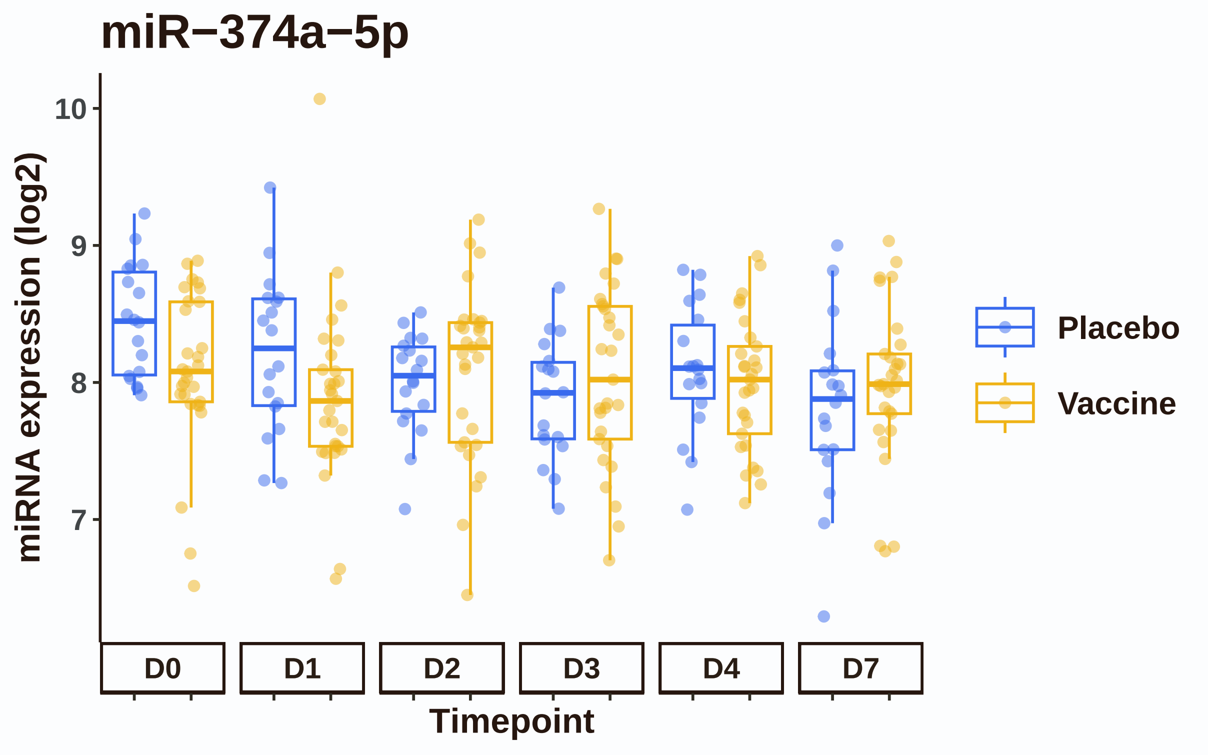

miR−374a−5p
miRNA expression (log2)
10
9
8
7
D0
D1
D2
D3
D4
D7
Timepoint
Placebo
Vaccine

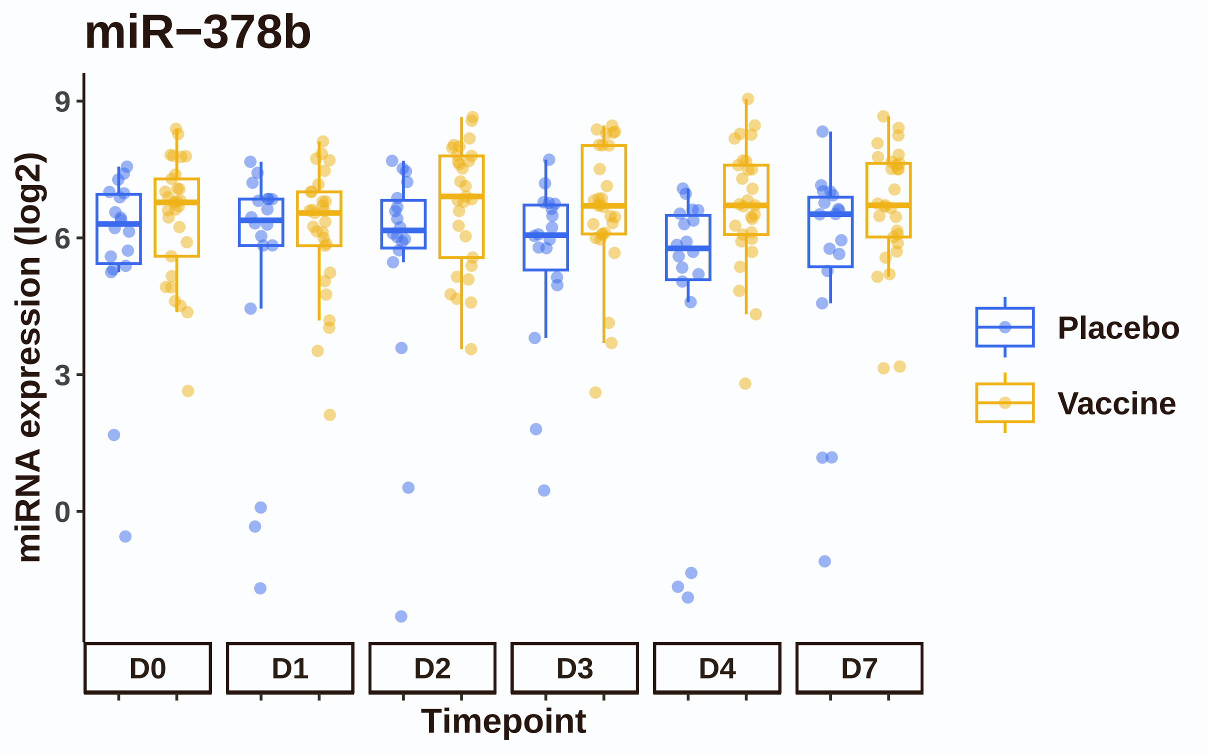

miR−378b
9
6
3
0
miRNA expression (log2)
D0
D1
D2
D3
D4
D7
Timepoint
Placebo
Vaccine

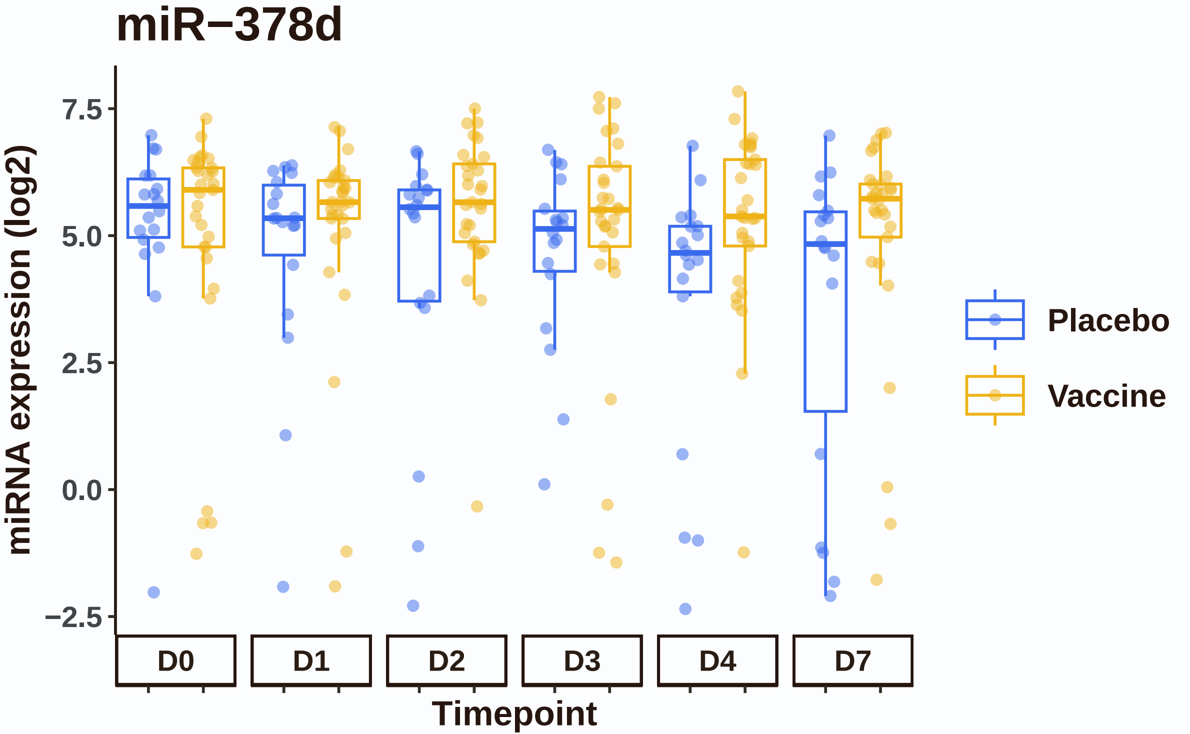
miR−378d
miRNA expression (log2)
7.5
5.0
2.5
0.0
−2.5
D0
D1
D2
D3
D4
D7
Timepoint
Placebo
Vaccine

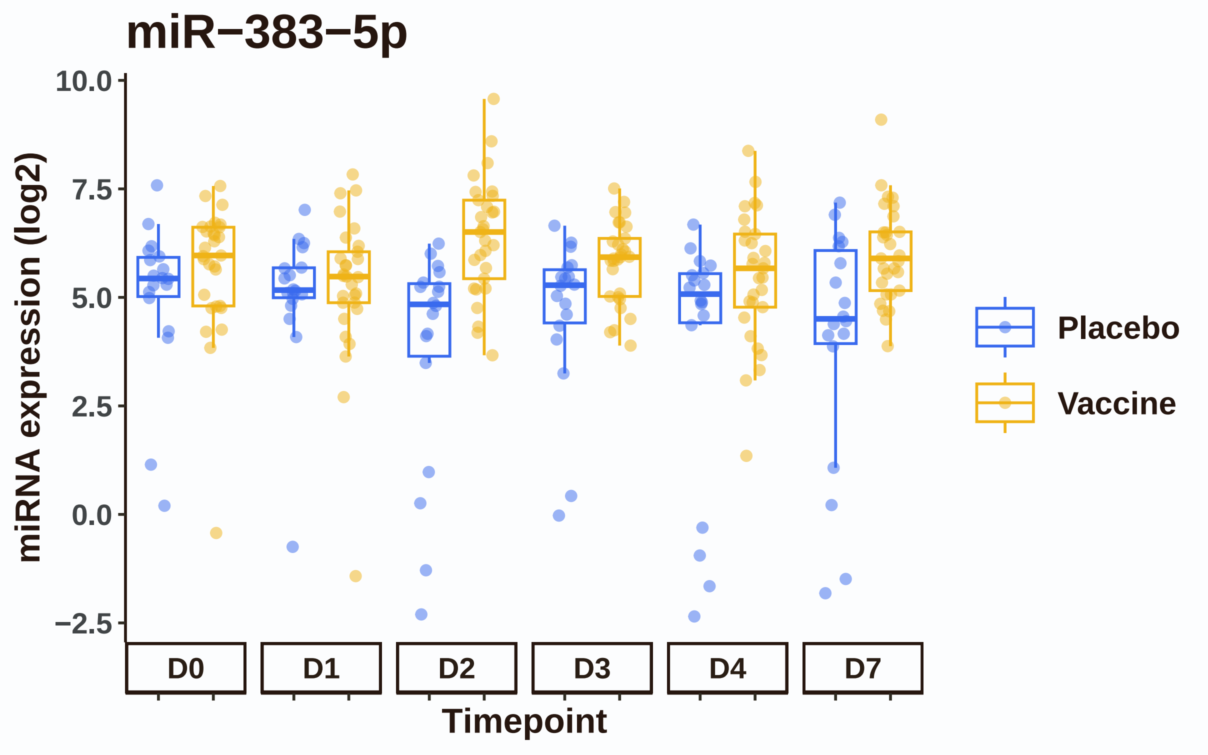

miR−383−5p
10.0
7.5
5.0
2.5
0.0
−2.5
miRNA expression (log2)
D0
D1
D2
D3
D4
D7
Timepoint
Placebo
Vaccine

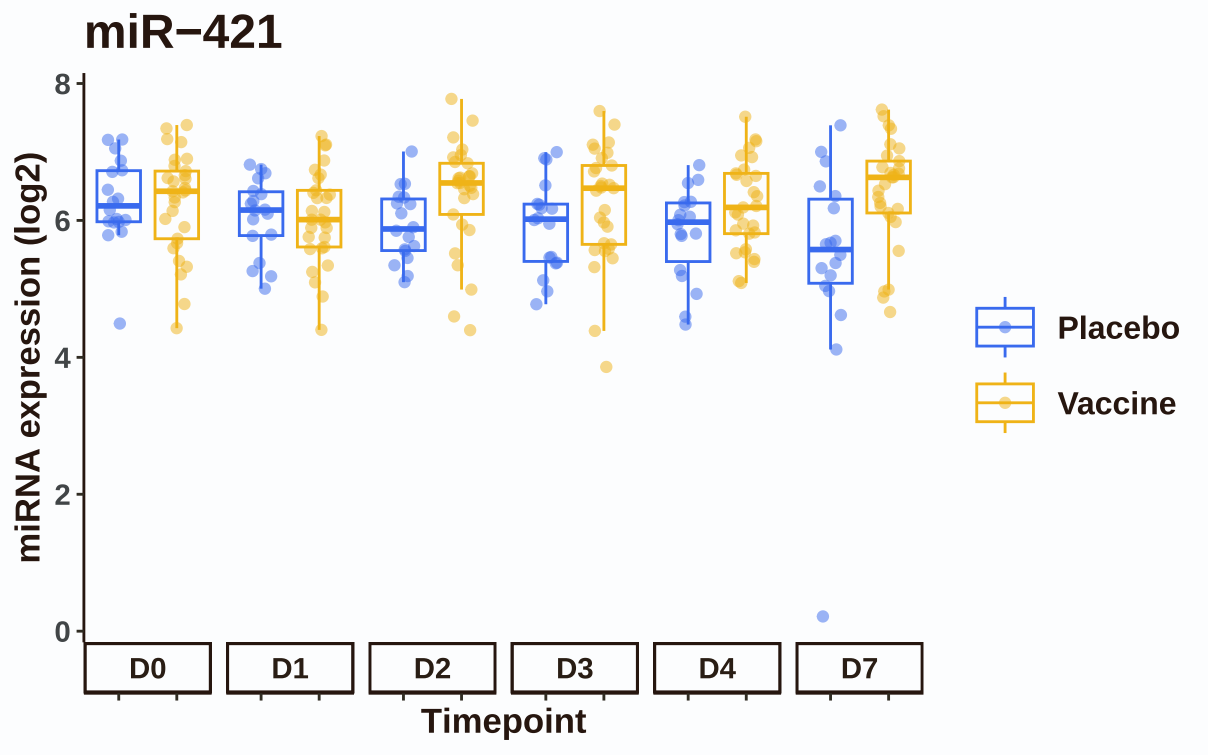

miR−421
miRNA expression (log2)
8
6
4
2
0
D0
D1
D2
D3
D4
D7
Timepoint
Placebo
Vaccine

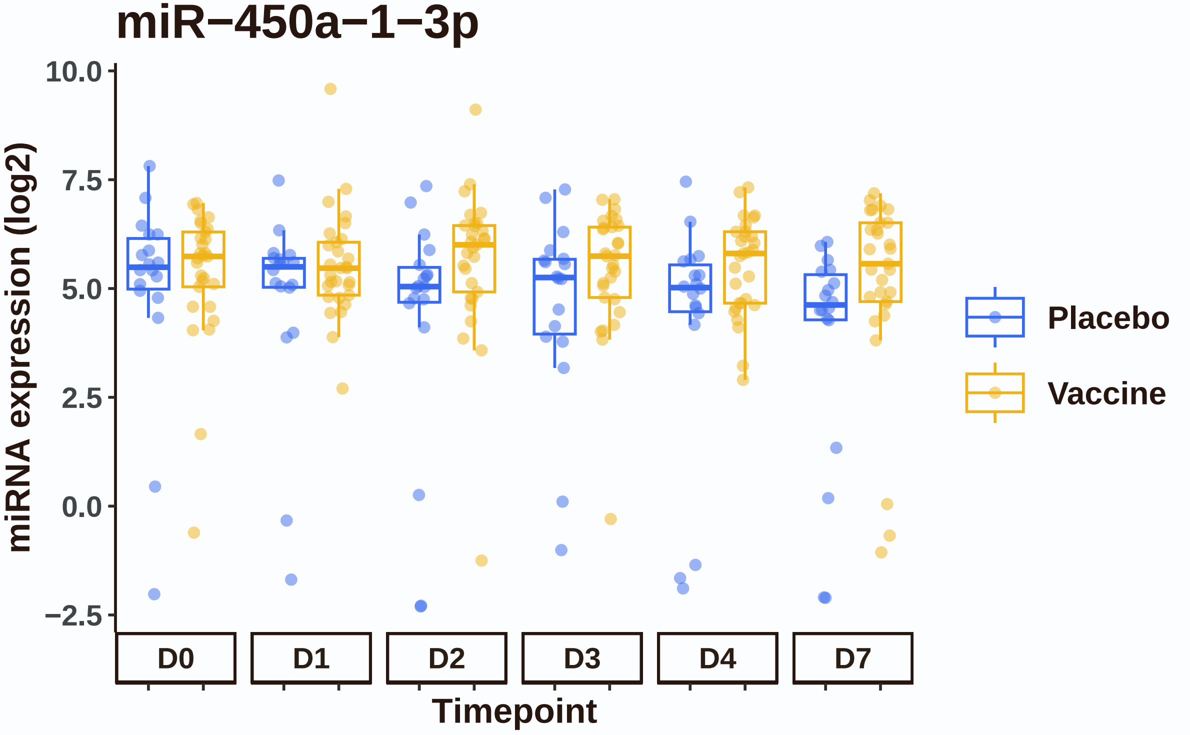

miR−450a−1−3p
miRNA expression (log2)
10.0
7.5
5.0
2.5
0.0
−2.5
D0
D1
D2
D3
D4
D7
Timepoint
Placebo
Vaccine

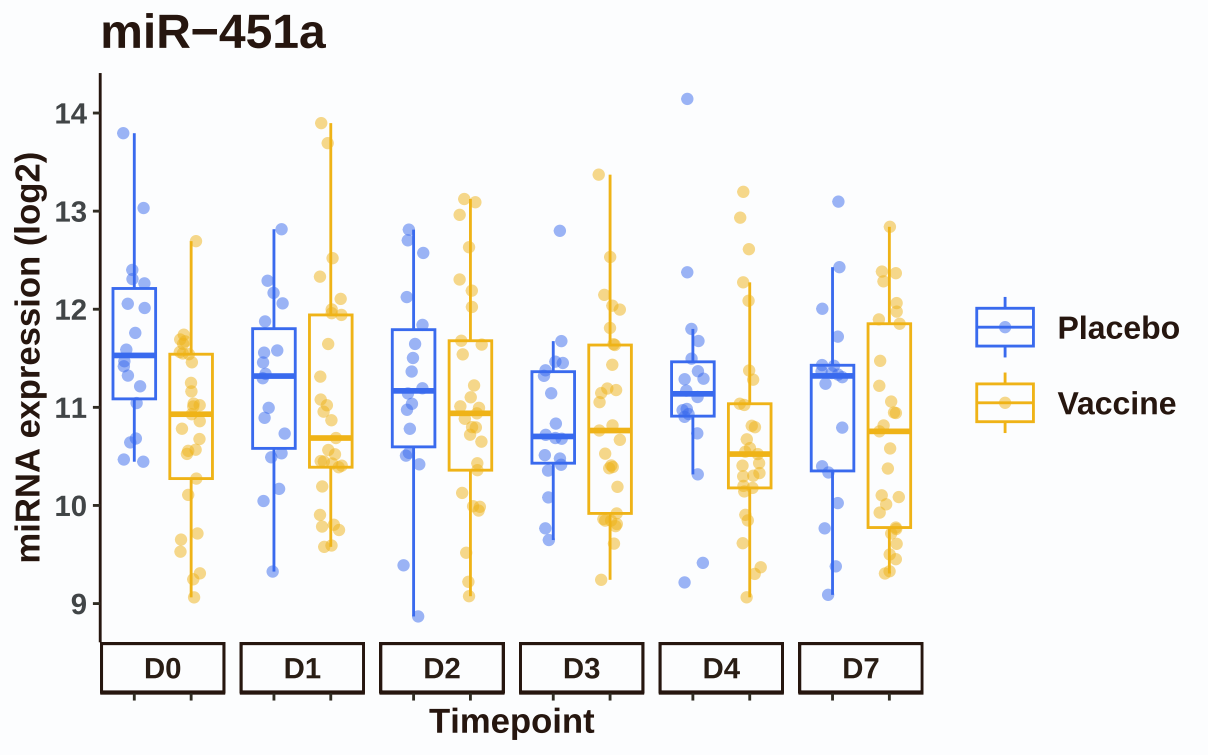
miR−451a
miRNA expression (log2)
14
13
12
11
10
9
D0
D1
D2
D3
D4
D7
Timepoint
Placebo
Vaccine

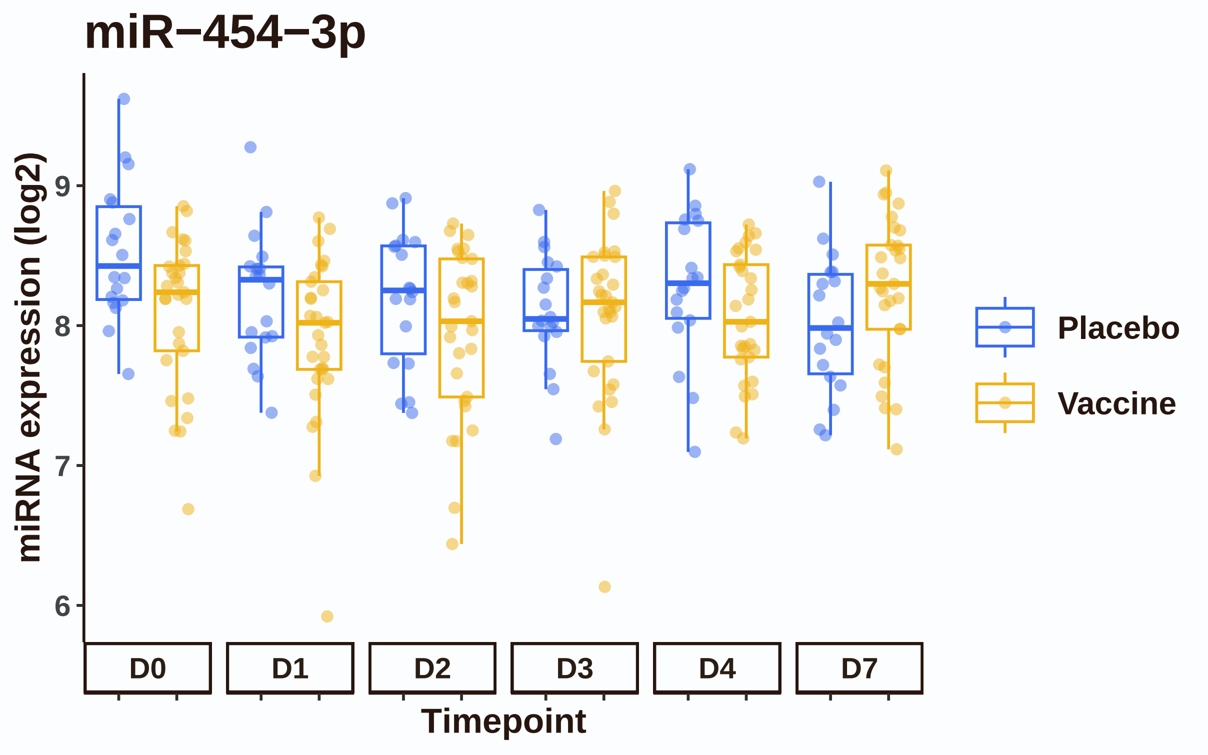
miR−454−3p
miRNA expression (log2)
9
8
7
6
D0
D1
D2
D3
D4
D7
Timepoint
Placebo
Vaccine

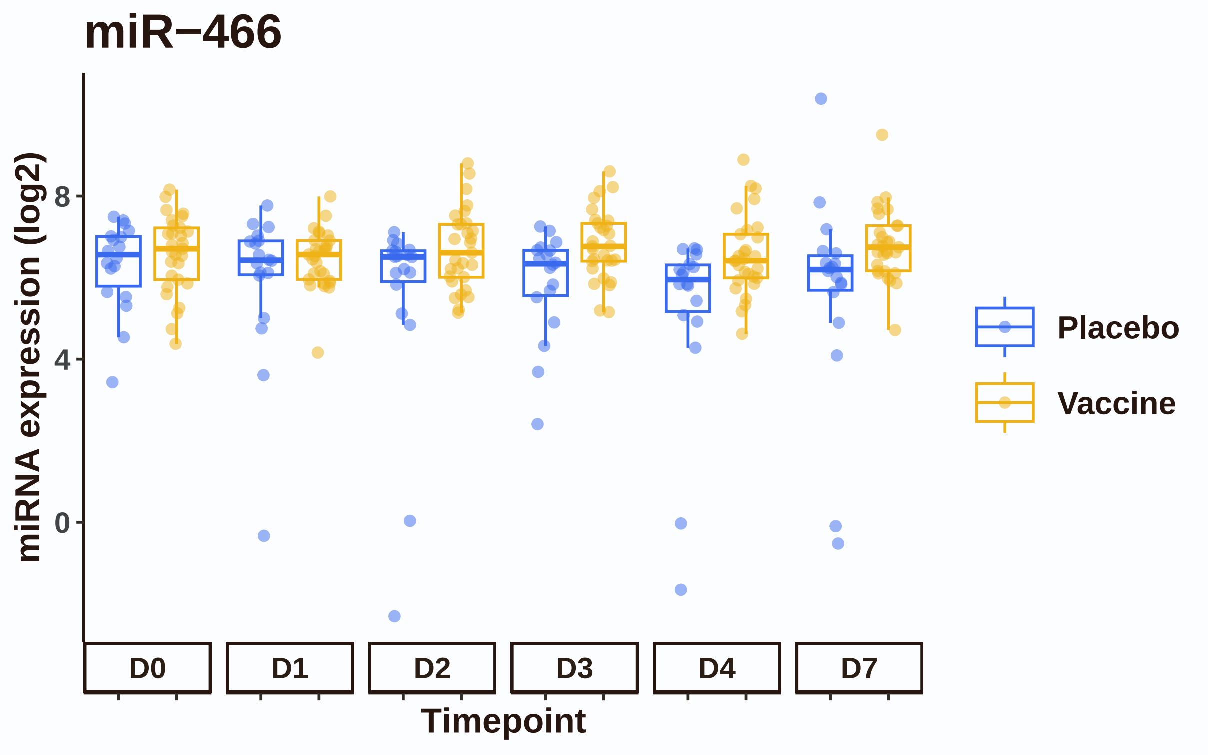
miR−466
miRNA expression (log2)
8
4
0
D0
D1
D2
D3
D4
D7
Timepoint
Placebo
Vaccine

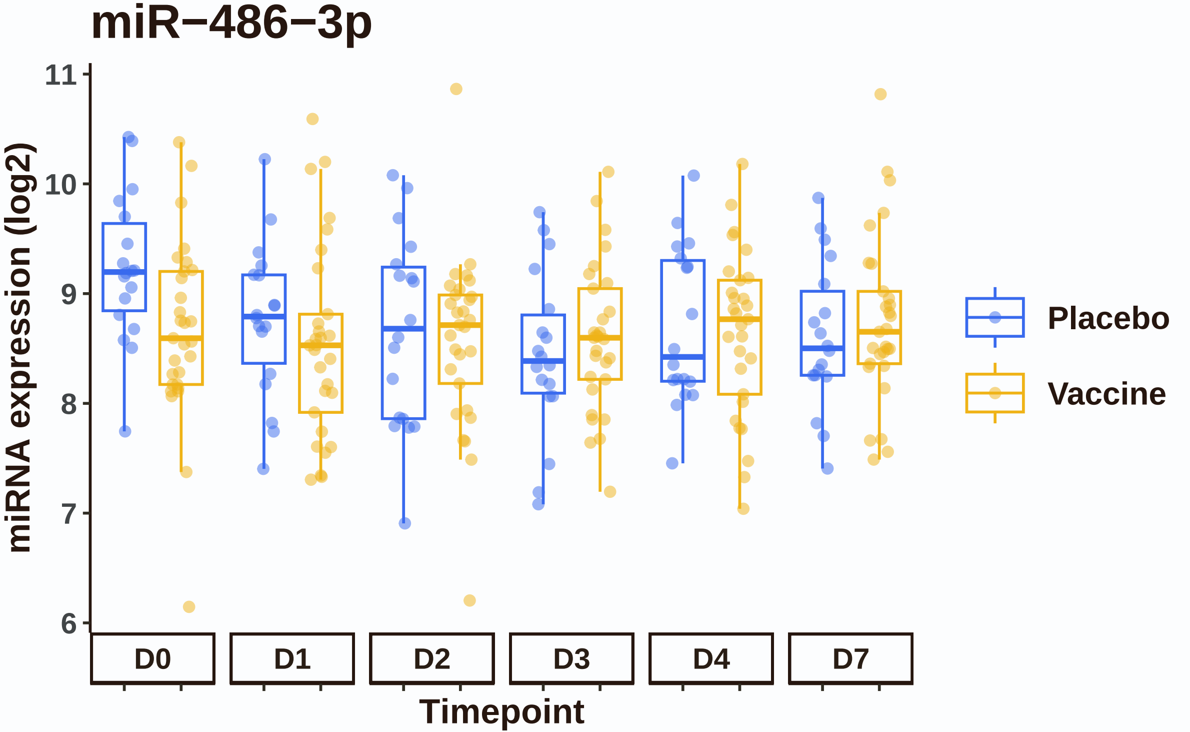

miR−486−3p
11
10
9
8
7
6
miRNA expression (log2)
D0
D1
D2
D3
D4
D7
Timepoint
Placebo
Vaccine

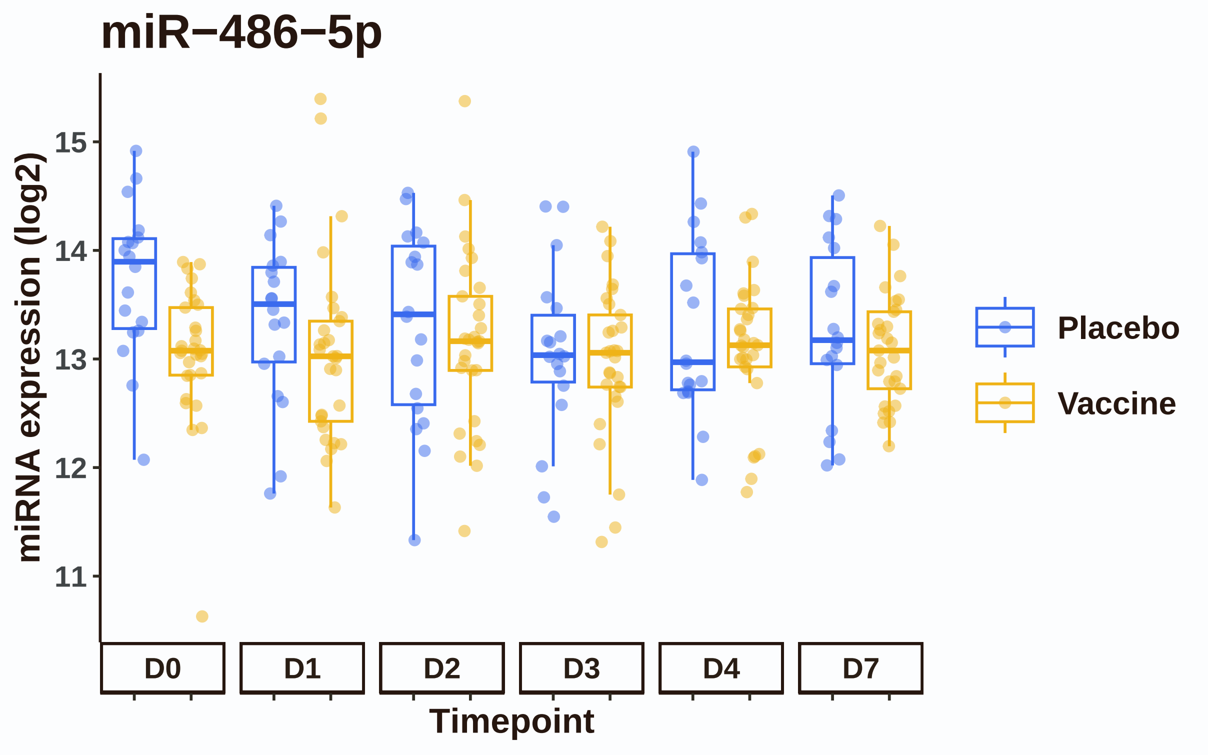
miR−486−5p
miRNA expression (log2)
15
14
13
12
11
D0
D1
D2
D3
D4
D7
Timepoint
Placebo
Vaccine

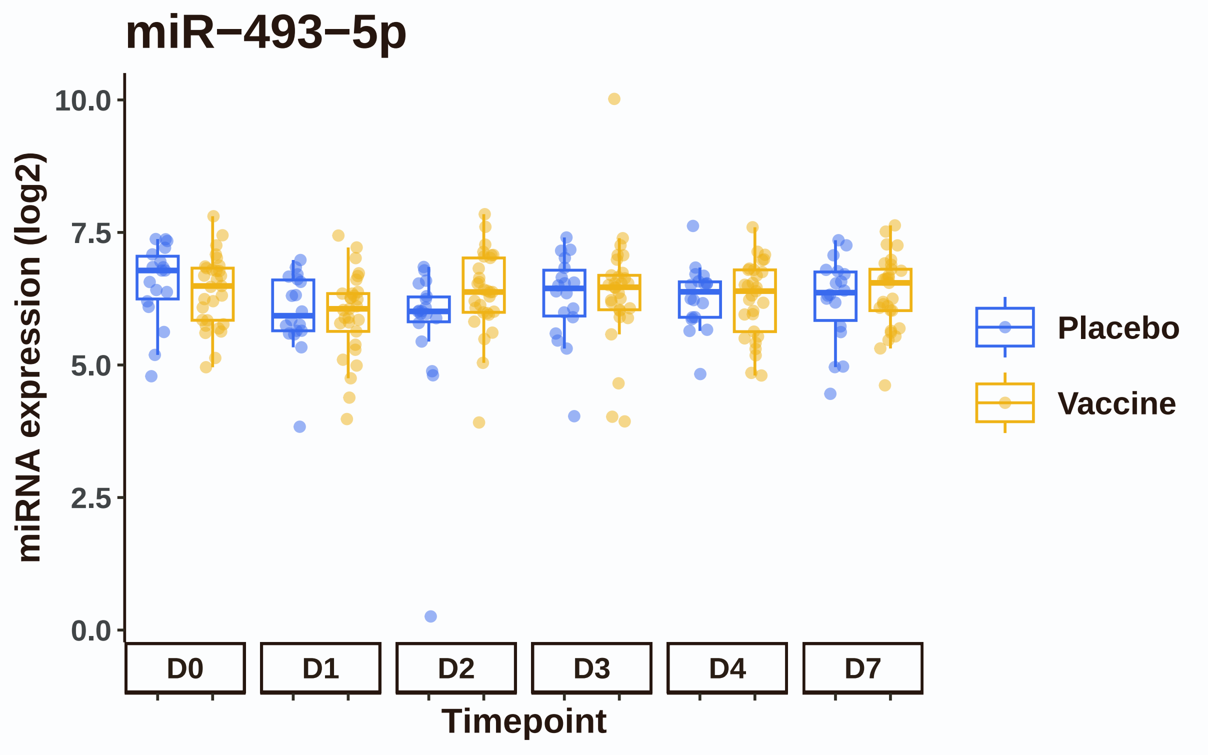

miR−493−5p
miRNA expression (log2)
10.0
7.5
5.0
2.5
0.0
D0
D1
D2
D3
D4
D7
Timepoint
Placebo
Vaccine

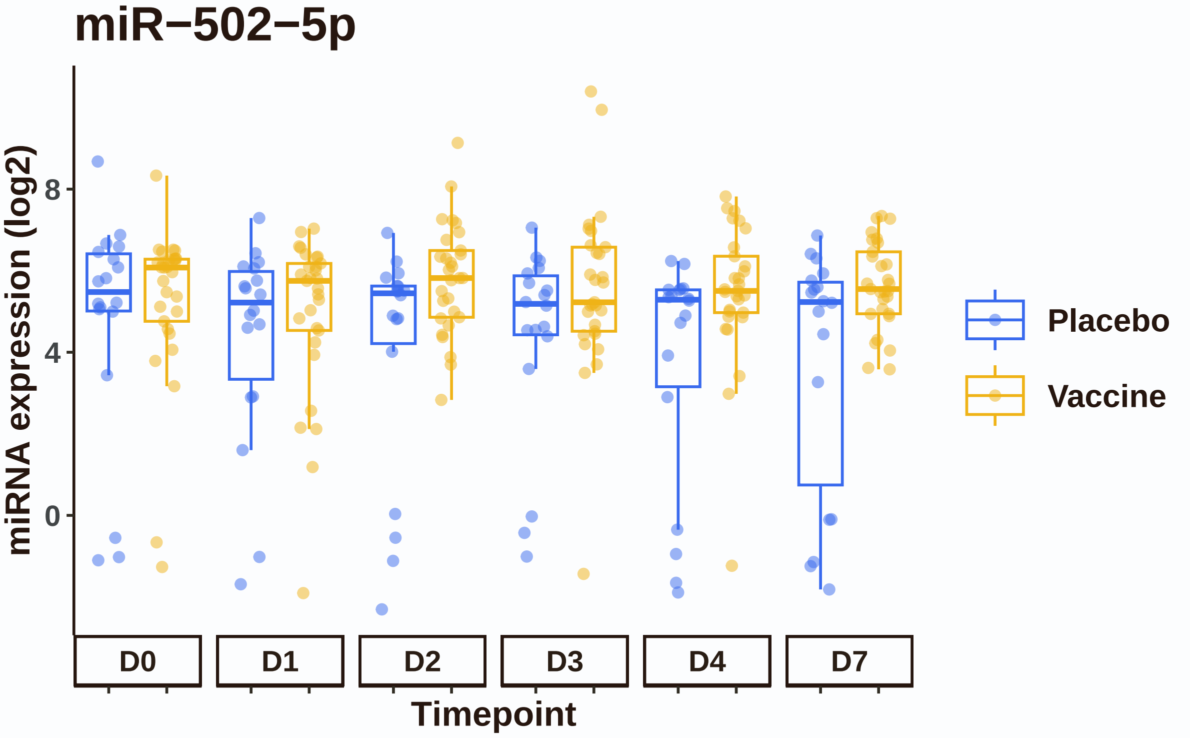

miR−502−5p
miRNA expression (log2)
8
4
0
D0
D1
D2
D3
D4
D7
Timepoint
Placebo
Vaccine

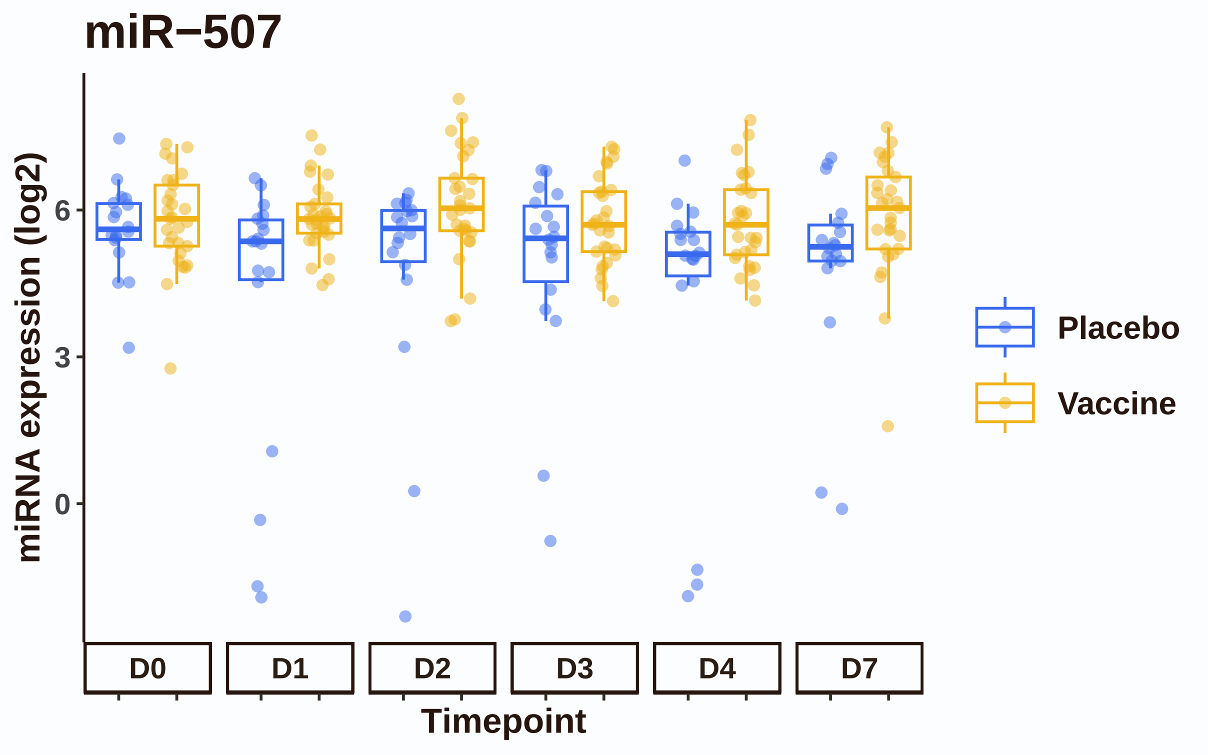
miR−507
miRNA expression (log2)
6
3
0
D0
D1
D2
D3
D4
D7
Timepoint
Placebo
Vaccine

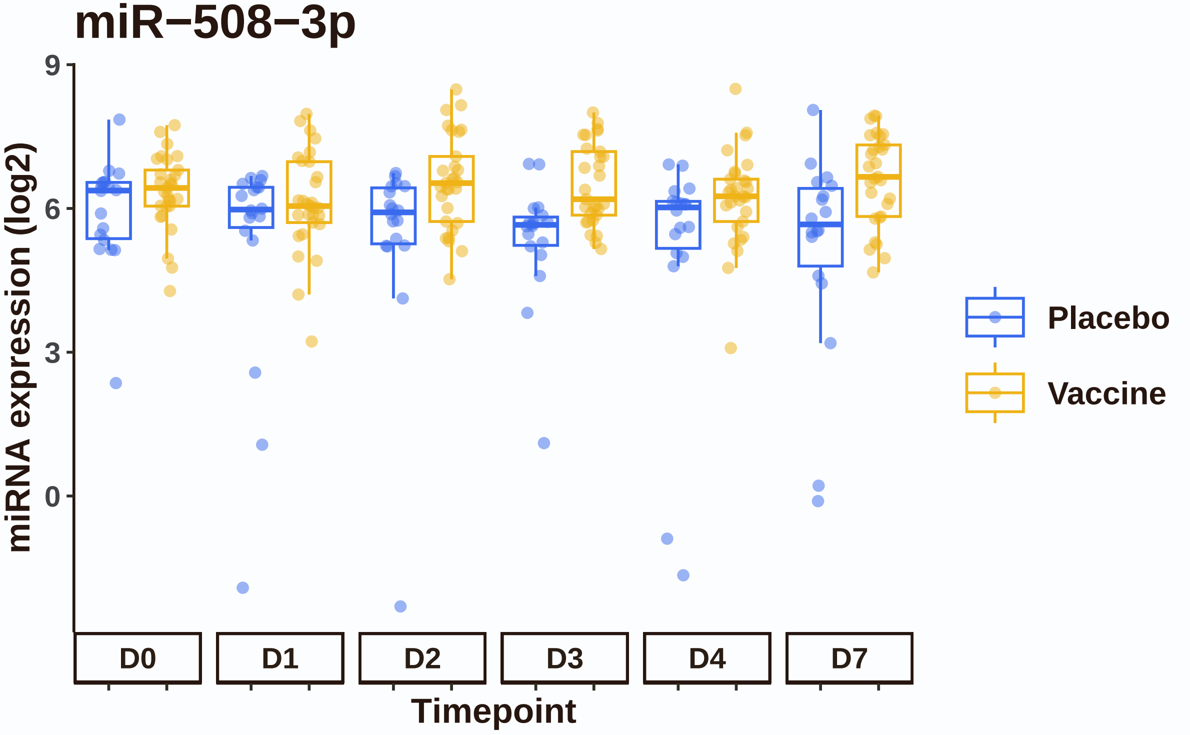
miR−508−3p
miRNA expression (log2)
9
6
3
0
D0
D1
D2
D3
D4
D7
Timepoint
Placebo
Vaccine

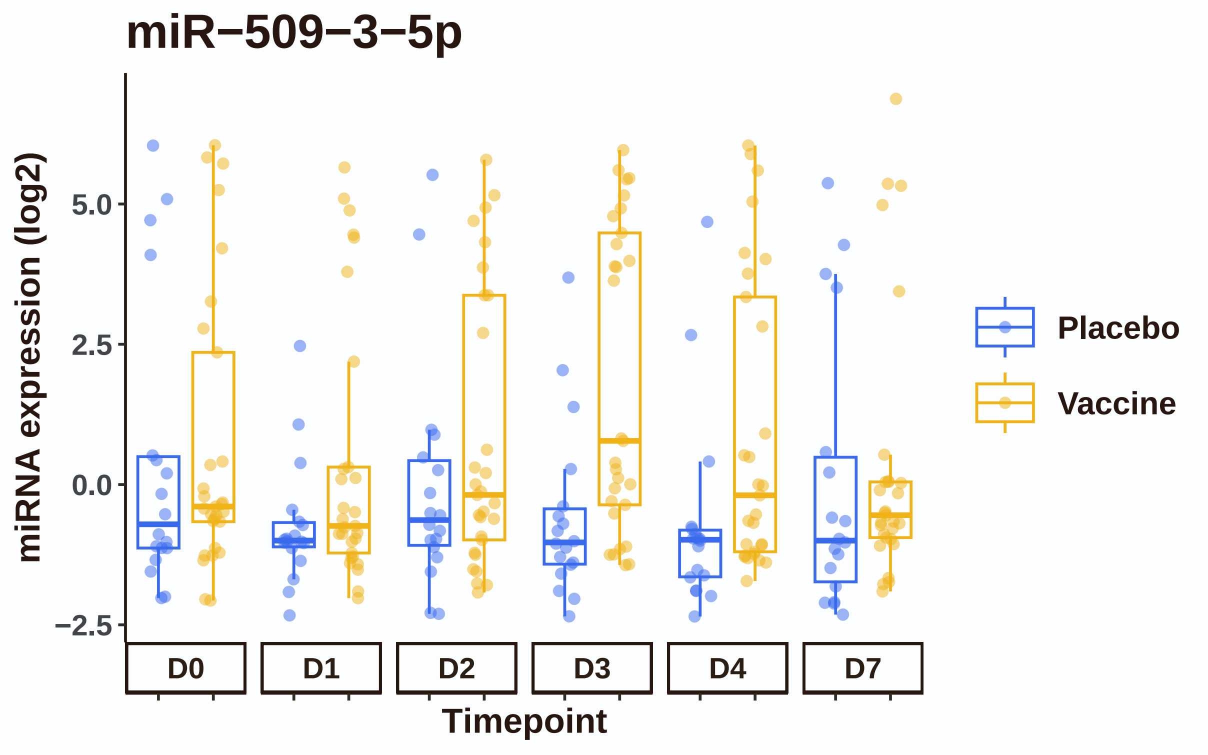
miR−509−3−5p
miRNA expression (log2)
5.0
2.5
0.0
−2.5
D0
D1
D2
D3
D4
D7
Timepoint
Placebo
Vaccine

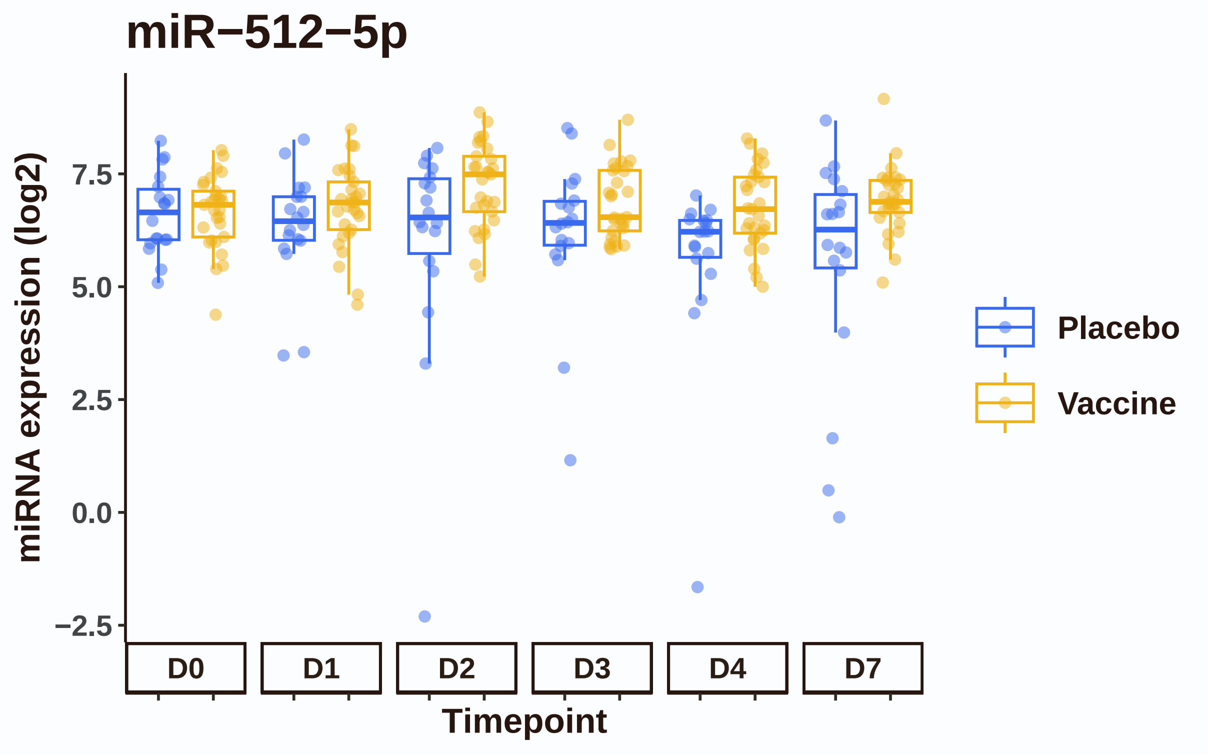

miR−512−5p
miRNA expression (log2)
7.5
5.0
2.5
0.0
−2.5
D0
D1
D2
D3
D4
D7
Timepoint
Placebo
Vaccine

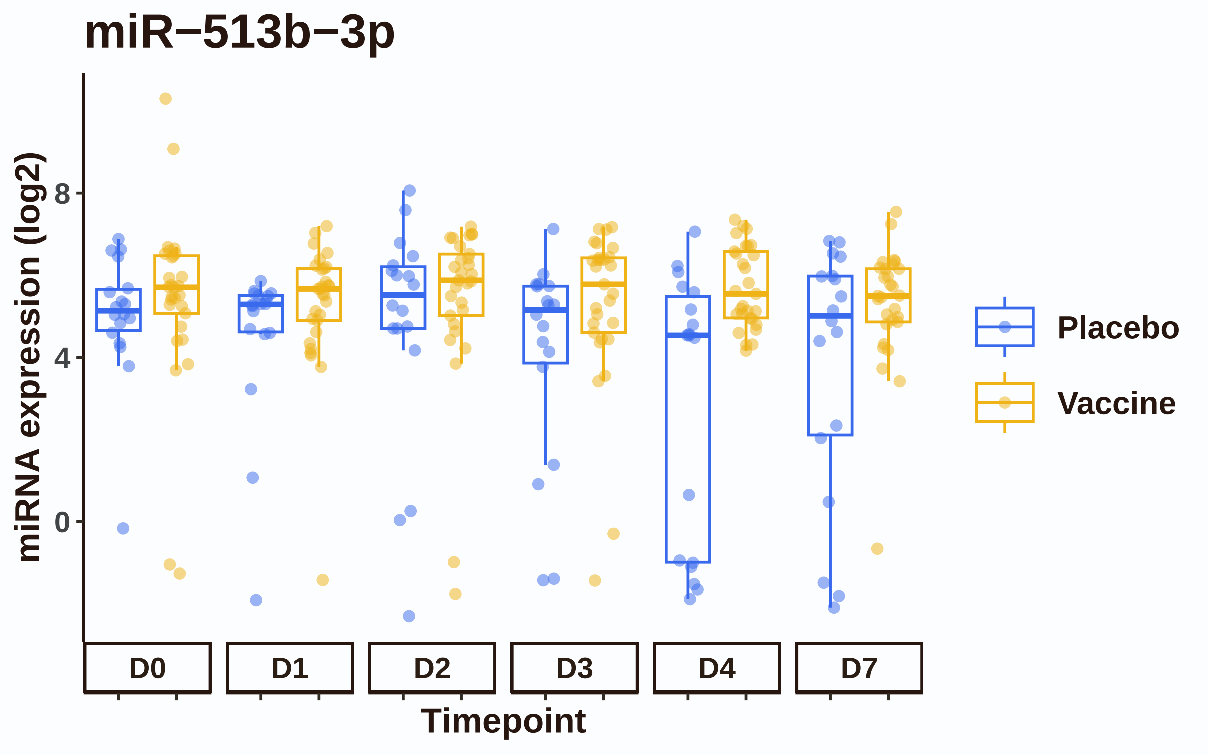

miR−513b−3p
miRNA expression (log2)
8
4
0
D0
D1
D2
D3
D4
D7
Timepoint
Placebo
Vaccine

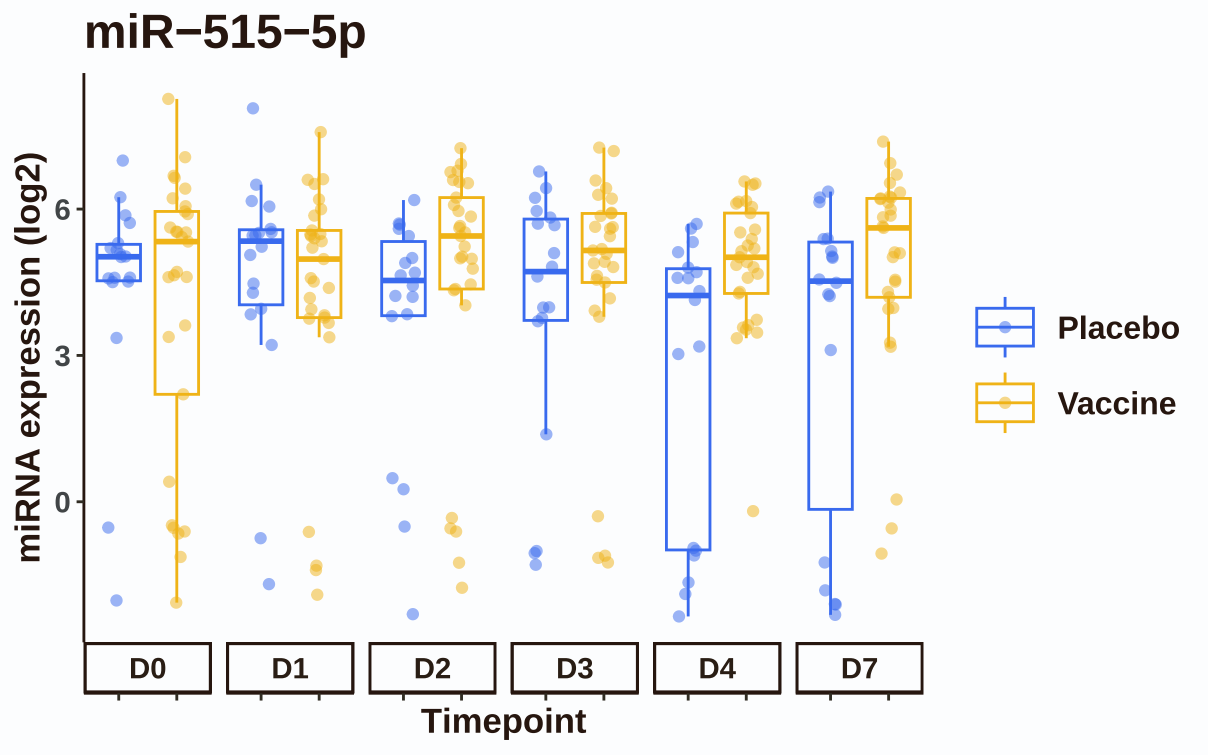

miR−515−5p
miRNA expression (log2)
6
3
0
D0
D1
D2
D3
D4
D7
Timepoint
Placebo
Vaccine

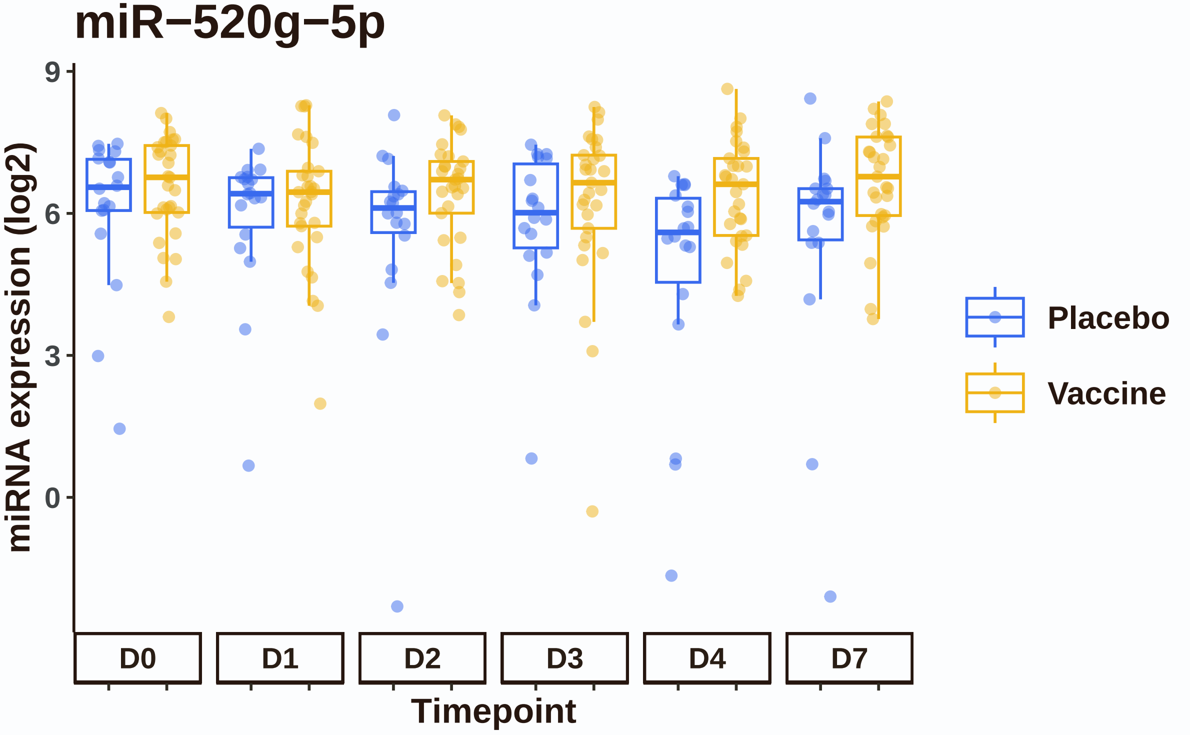

miR−520g−5p
9
6
3
0
miRNA expression (log2)
D0
D1
D2
D3
D4
D7
Timepoint
Placebo
Vaccine

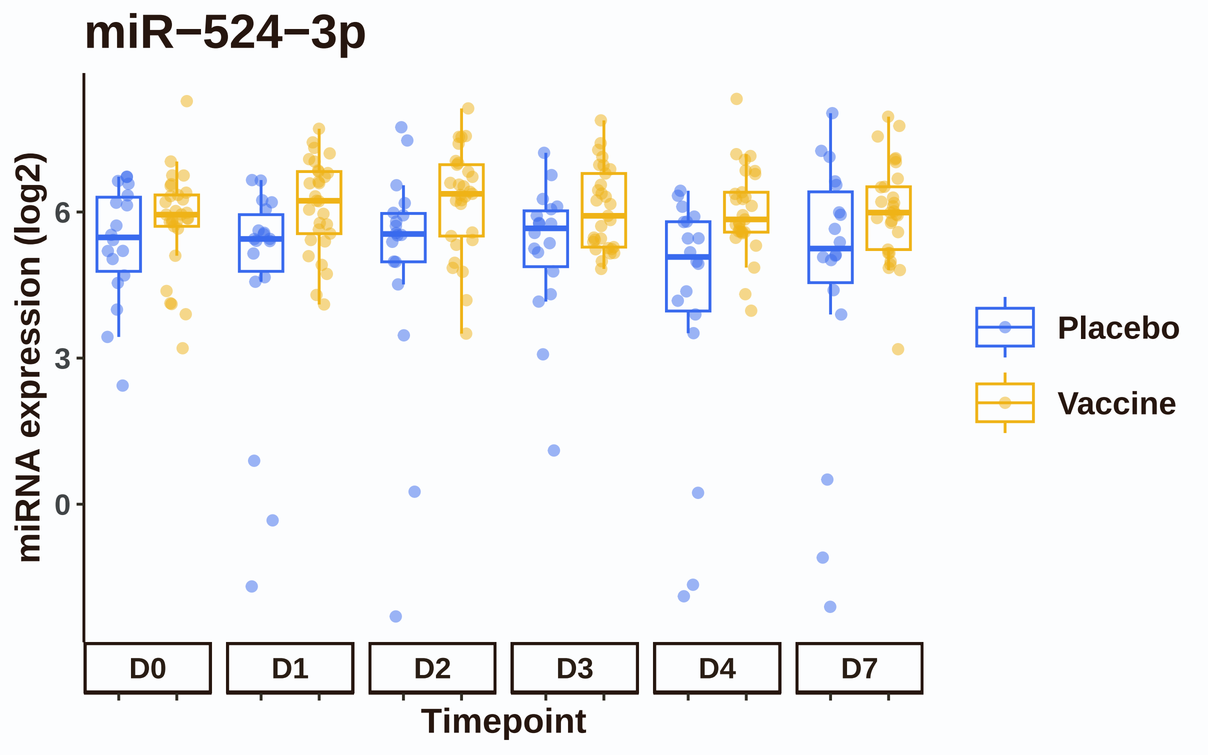

miR−524−3p
miRNA expression (log2)
0
3
6
D0
D1
D2
D3
D4
D7
Timepoint
Placebo
Vaccine

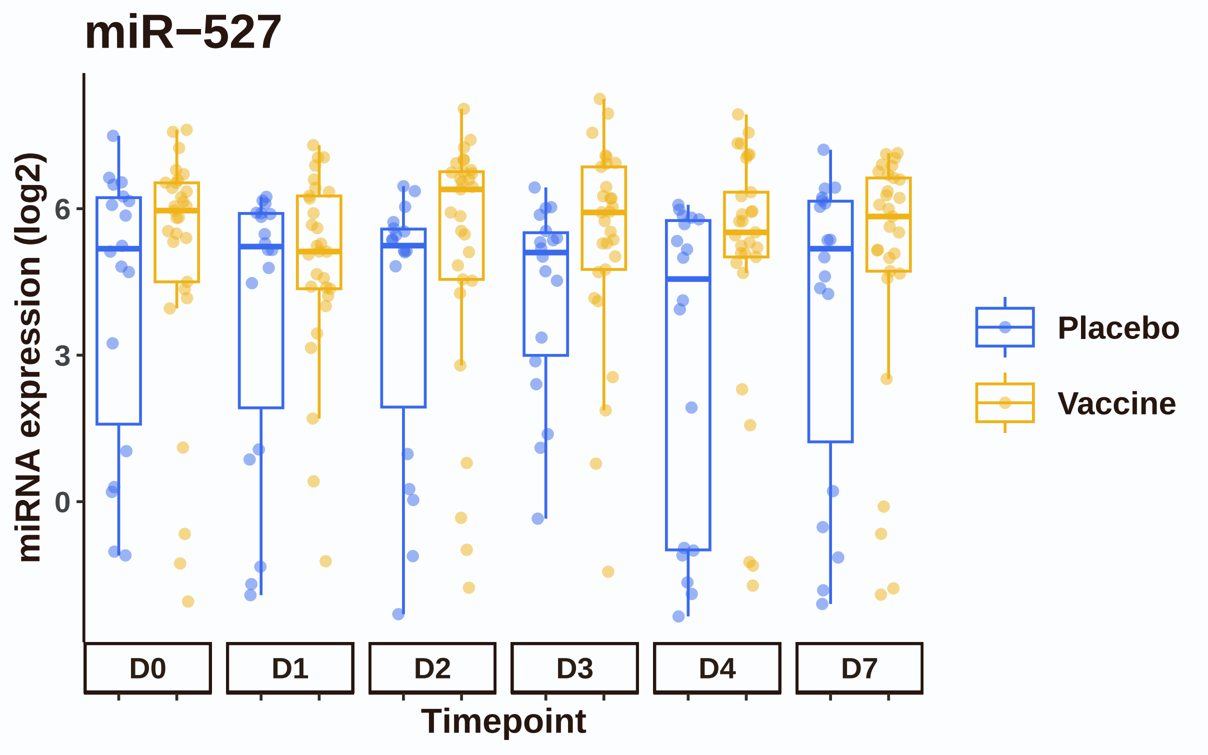
miR−527
miRNA expression (log2)
6
3
0
D0
D1
D2
D3
D4
D7
Timepoint
Placebo
Vaccine

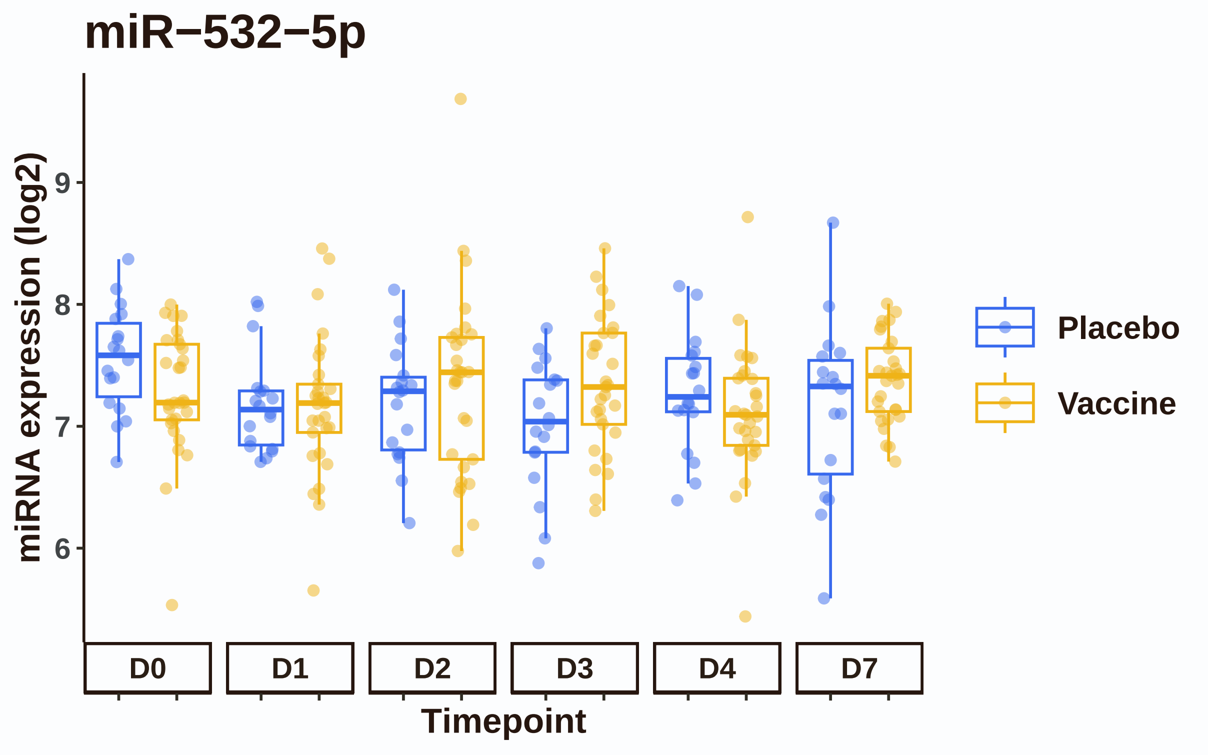
miR−532−5p
miRNA expression (log2)
9
8
7
6
D0
D1
D2
D3
D4
D7
Timepoint
Placebo
Vaccine

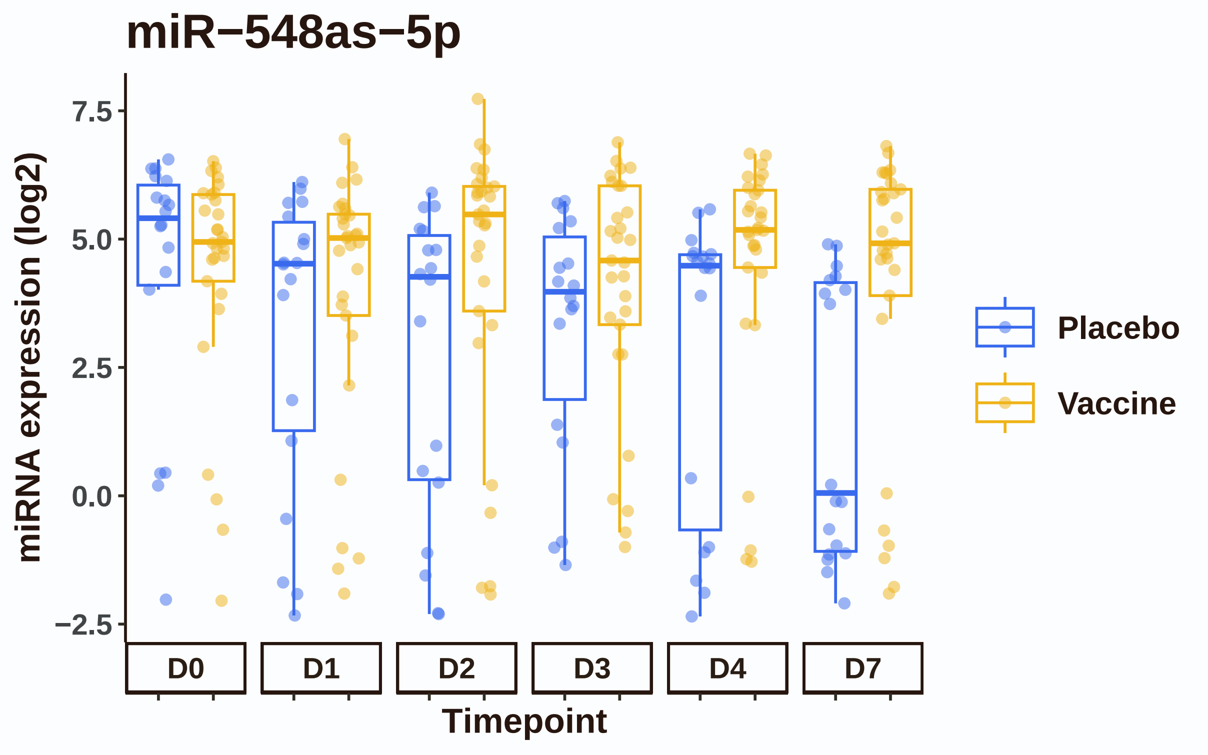
miR−548as−5p
miRNA expression (log2)
7.5
5.0
2.5
0.0
−2.5
D0
D1
D2
D3
D4
D7
Timepoint
Placebo
Vaccine

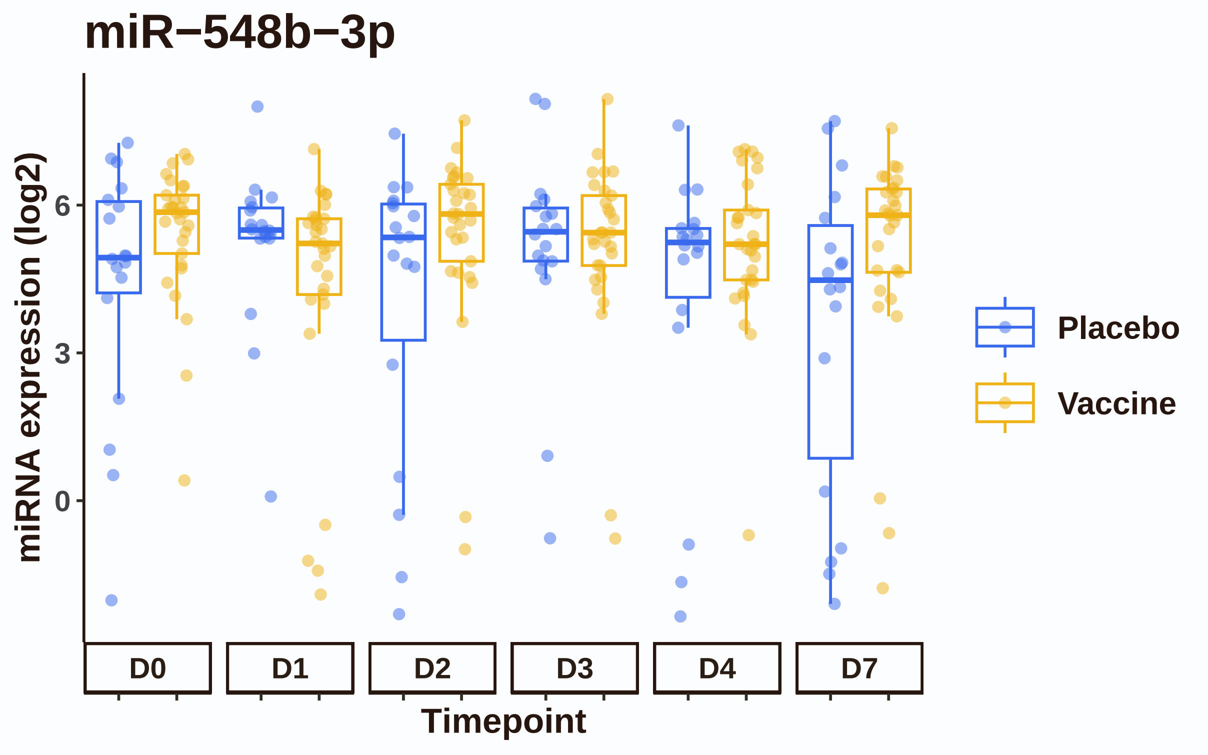
miR−548b−3p
miRNA expression (log2)
6
3
0
D0
D1
D2
D3
D4
D7
Timepoint
Placebo
Vaccine

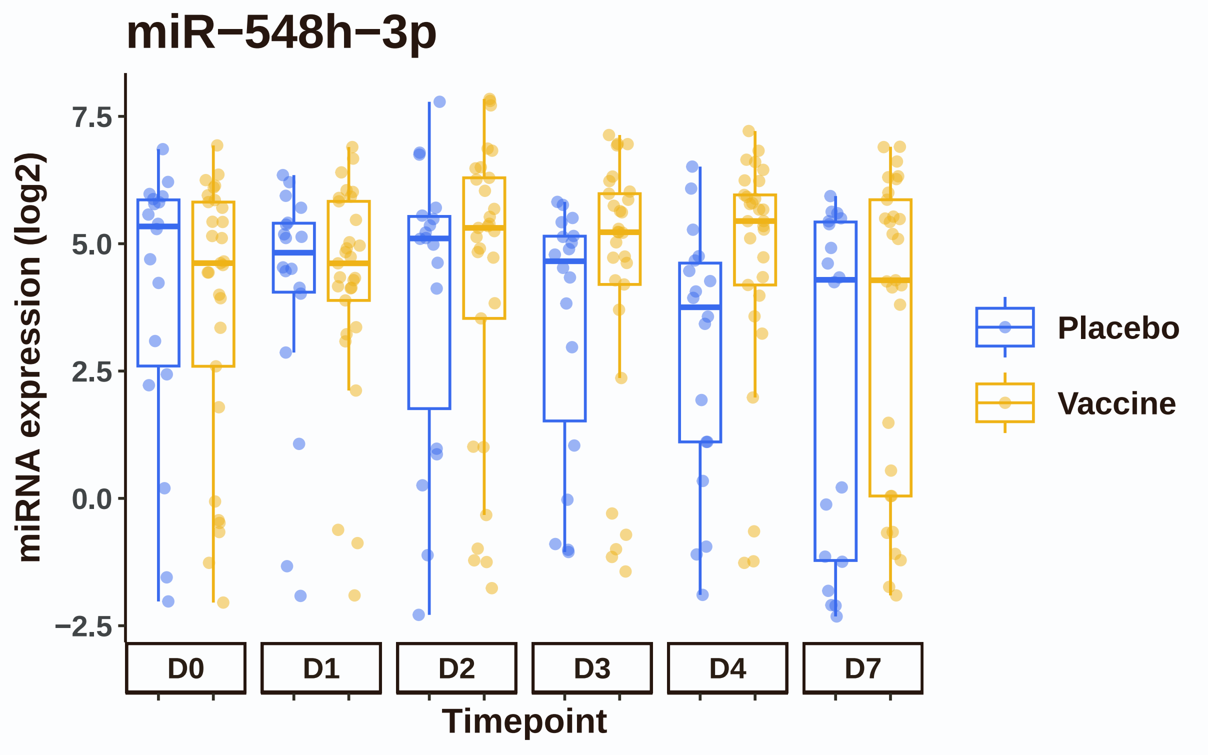

miR−548h−3p
miRNA expression (log2)
7.5
5.0
2.5
0.0
−2.5
D0
D1
D2
D3
D4
D7
Timepoint
Placebo
Vaccine

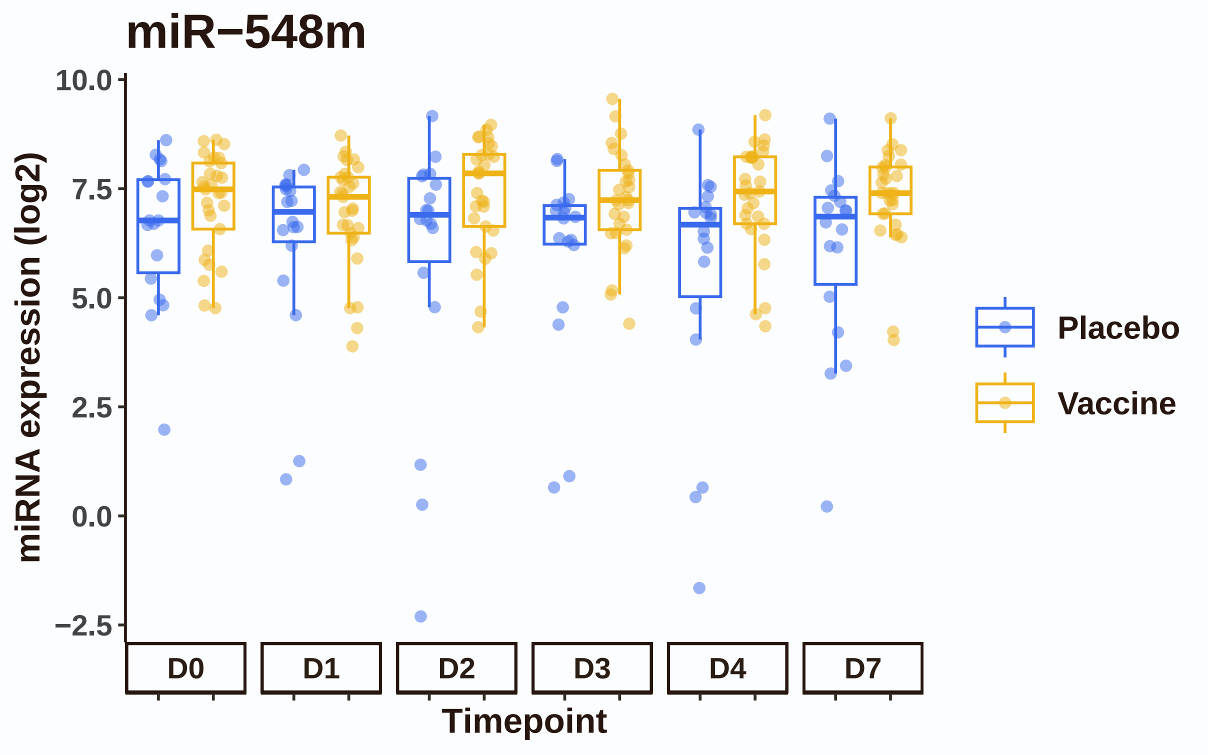

miR−548m
10.0
7.5
5.0
2.5
0.0
−2.5
miRNA expression (log2)
D0
D1
D2
D3
D4
D7
Timepoint
Placebo
Vaccine

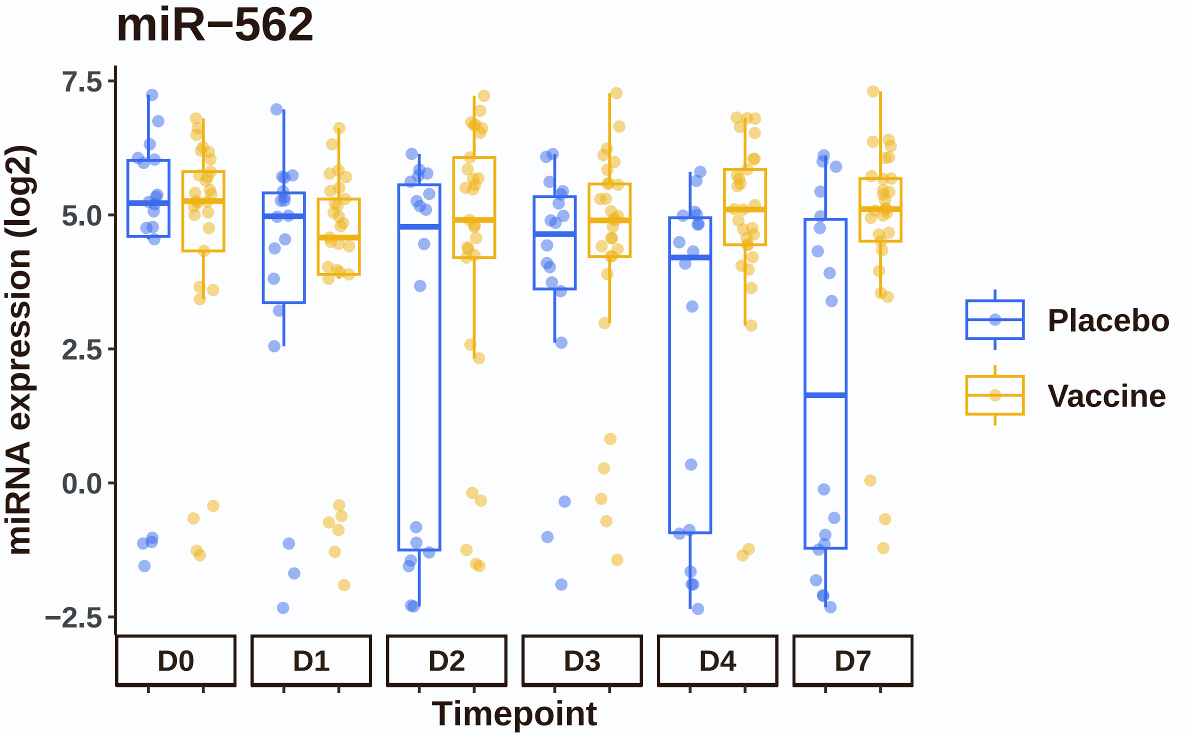
miR−562
miRNA expression (log2)
7.5
5.0
2.5
0.0
−2.5
D0
D1
D2
D3
D4
D7
Timepoint
Placebo
Vaccine

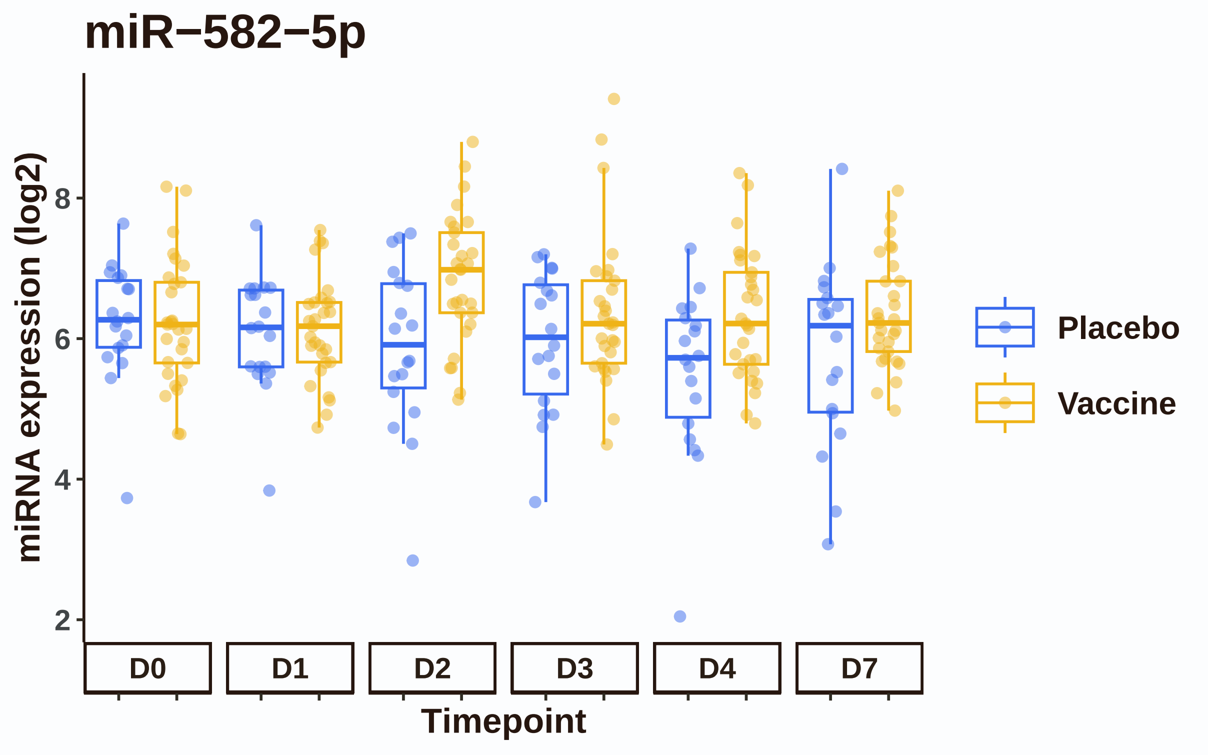

miR−582−5p
miRNA expression (log2)
2
4
6
8
D0
D1
D2
D3
D4
D7
Timepoint
Placebo
Vaccine

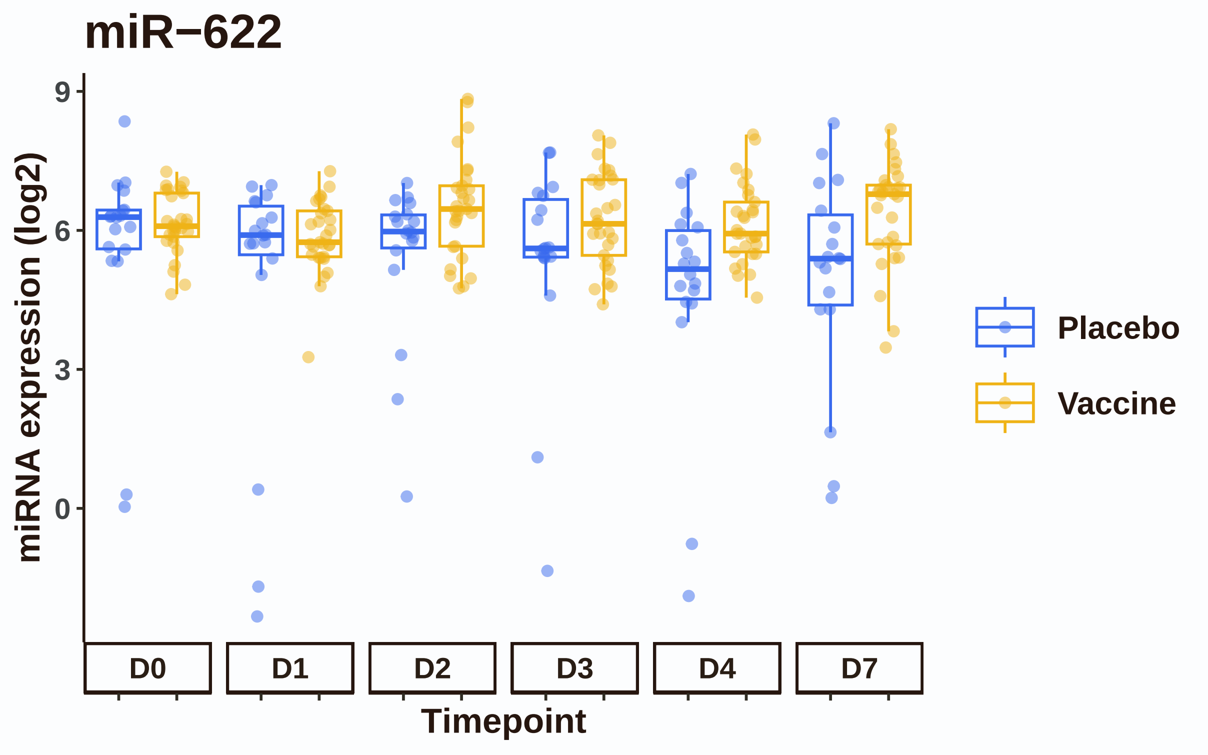
miR−622
miRNA expression (log2)
9
6
3
0
D0
D1
D2
D3
D4
D7
Timepoint
Placebo
Vaccine

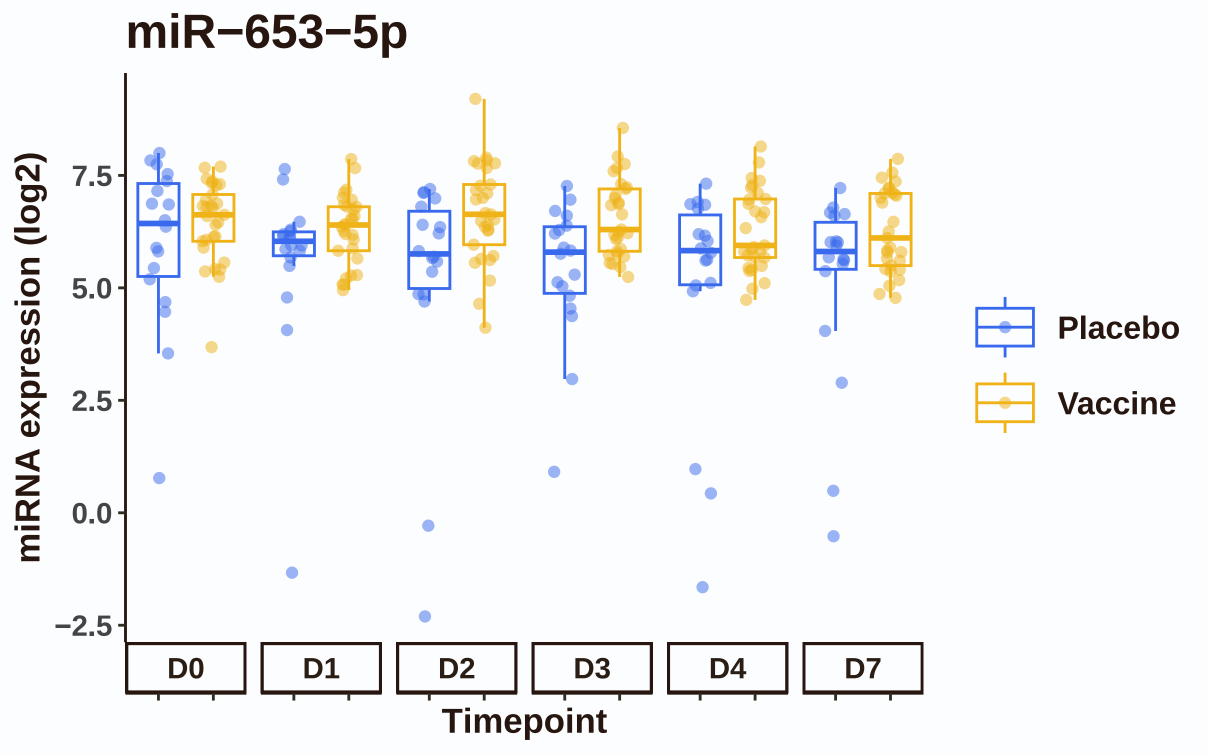
miR−653−5p
miRNA expression (log2)
7.5
5.0
2.5
0.0
−2.5
D0
D1
D2
D3
D4
D7
Timepoint
Placebo
Vaccine

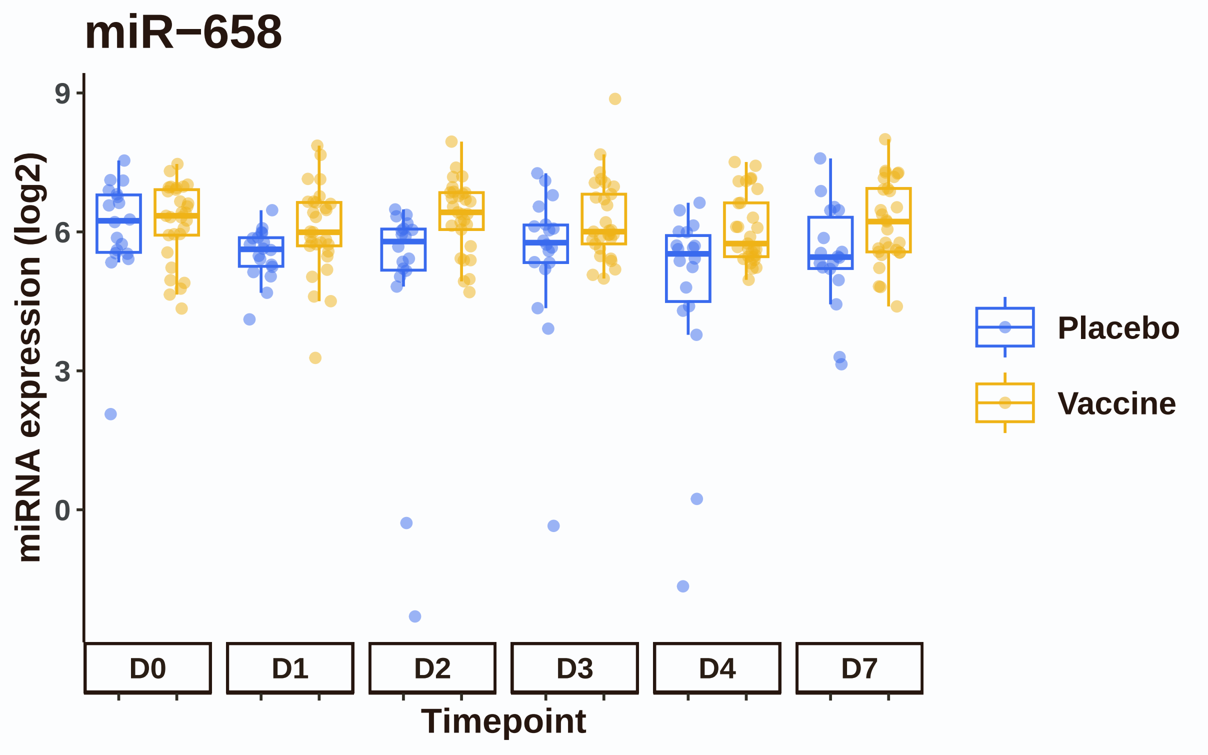

miR−658
miRNA expression (log2)
9
6
3
0
D0
D1
D2
D3
D4
D7
Timepoint
Placebo
Vaccine

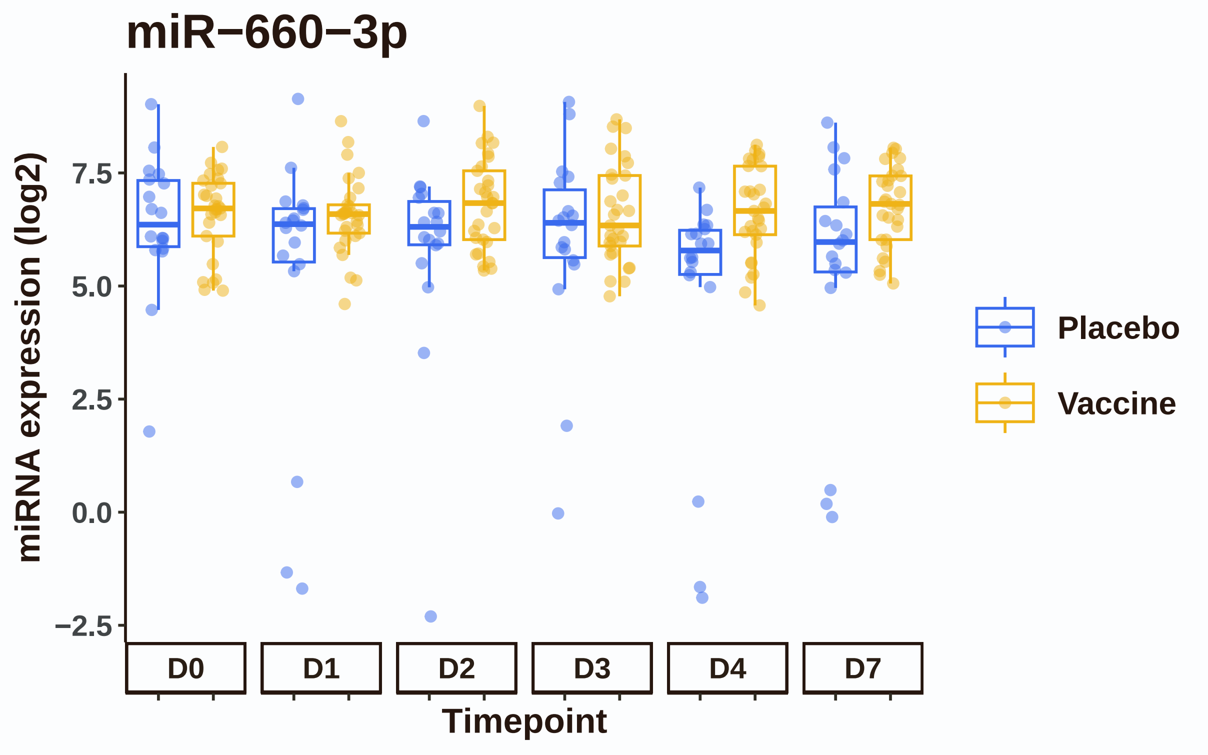

miR−660−3p
miRNA expression (log2)
7.5
5.0
2.5
0.0
−2.5
D0
D1
D2
D3
D4
D7
Timepoint
Placebo
Vaccine

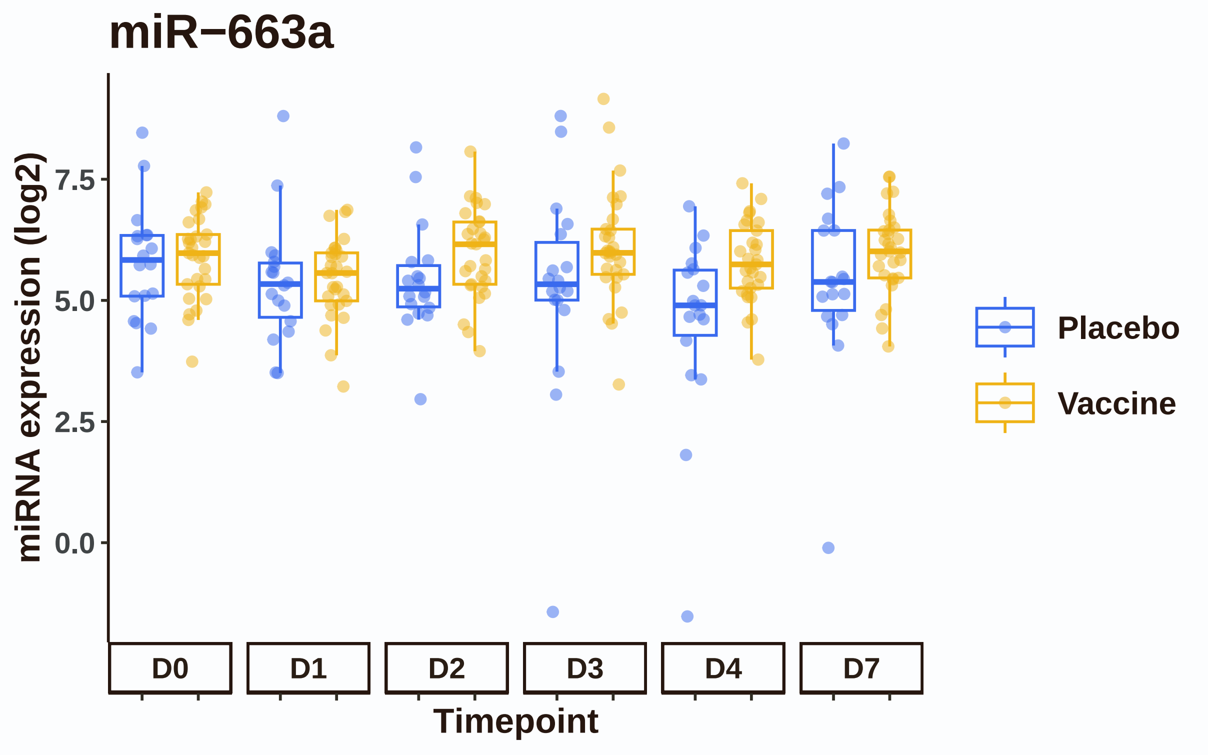

miR−663a
miRNA expression (log2)
7.5
5.0
2.5
0.0
D0
D1
D2
D3
D4
D7
Timepoint
Placebo
Vaccine

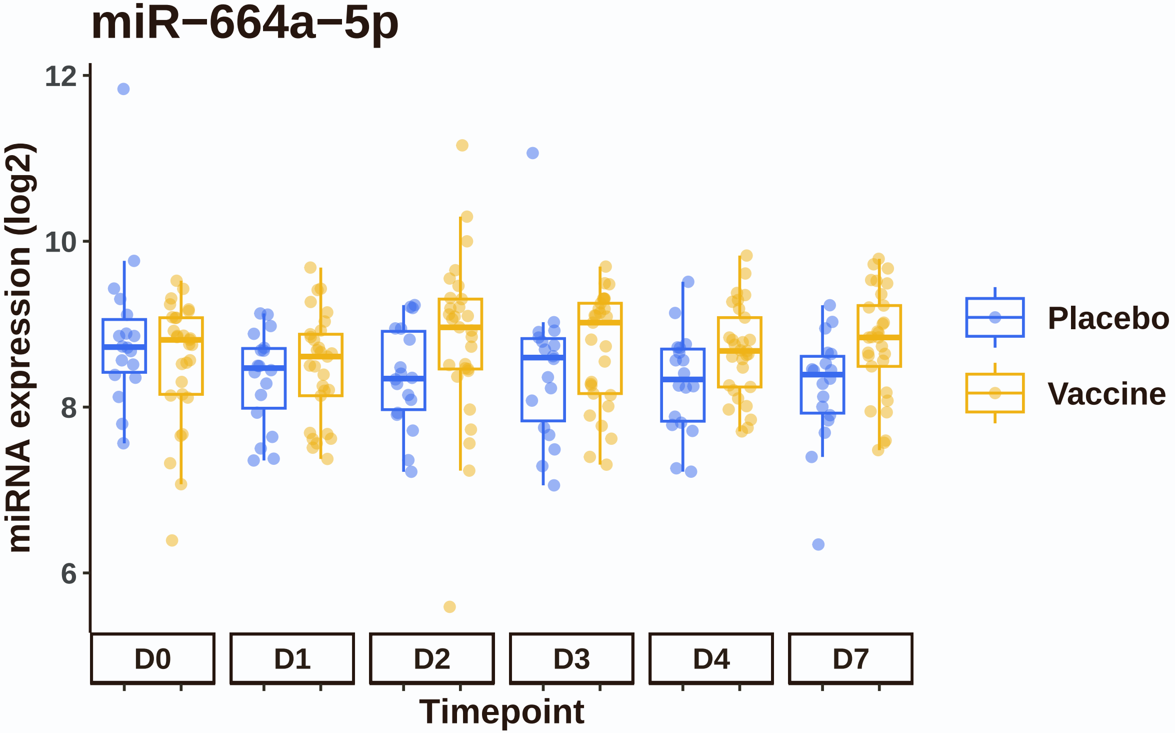
miR−664a−5p
miRNA expression (log2)
12
10
8
6
D0
D1
D2
D3
D4
D7
Timepoint
Placebo
Vaccine

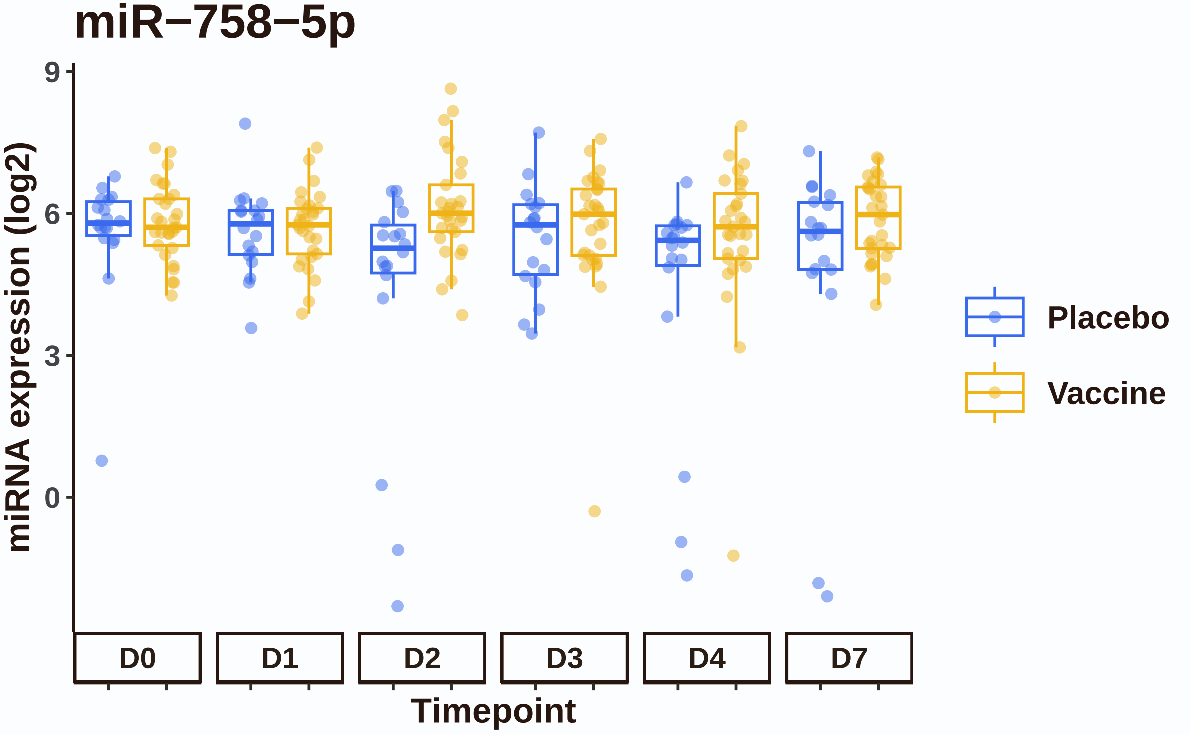

miR−758−5p
miRNA expression (log2)
9
6
3
0
D0
D1
D2
D3
D4
D7
Timepoint
Placebo
Vaccine

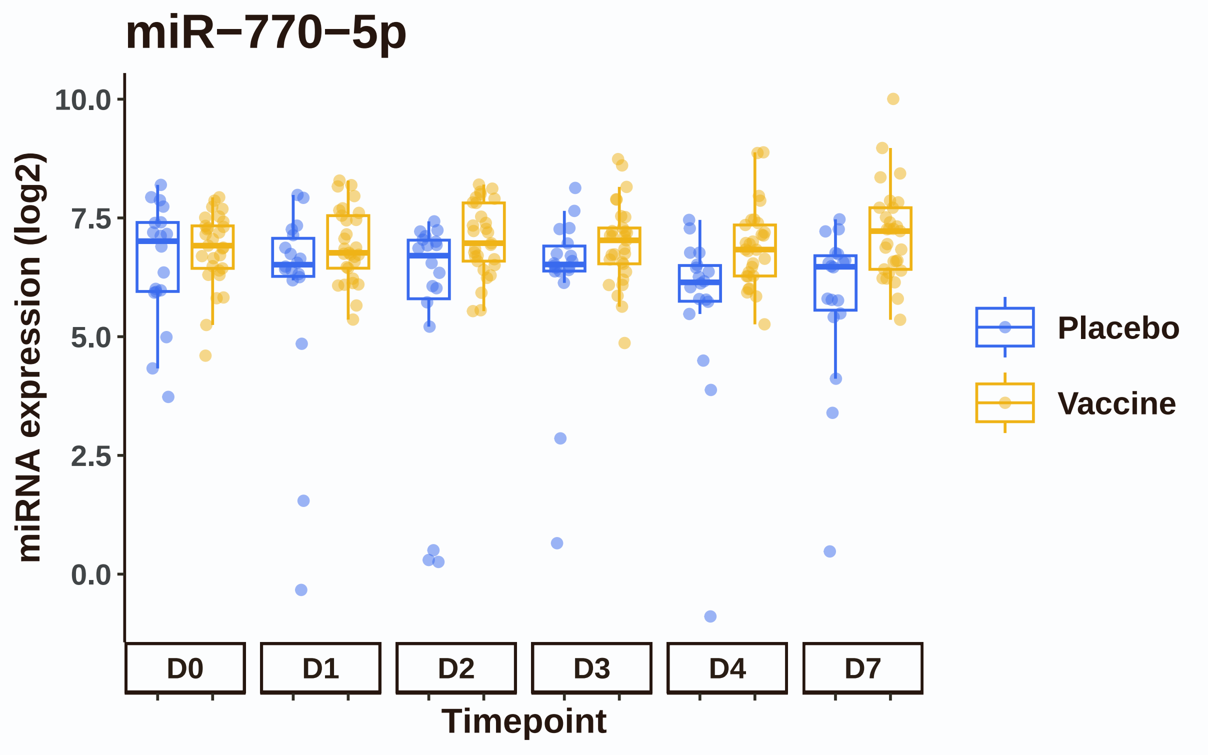
miR−770−5p
10.0
7.5
5.0
2.5
0.0
miRNA expression (log2)
D0
D1
D2
D3
D4
D7
Timepoint
Placebo
Vaccine

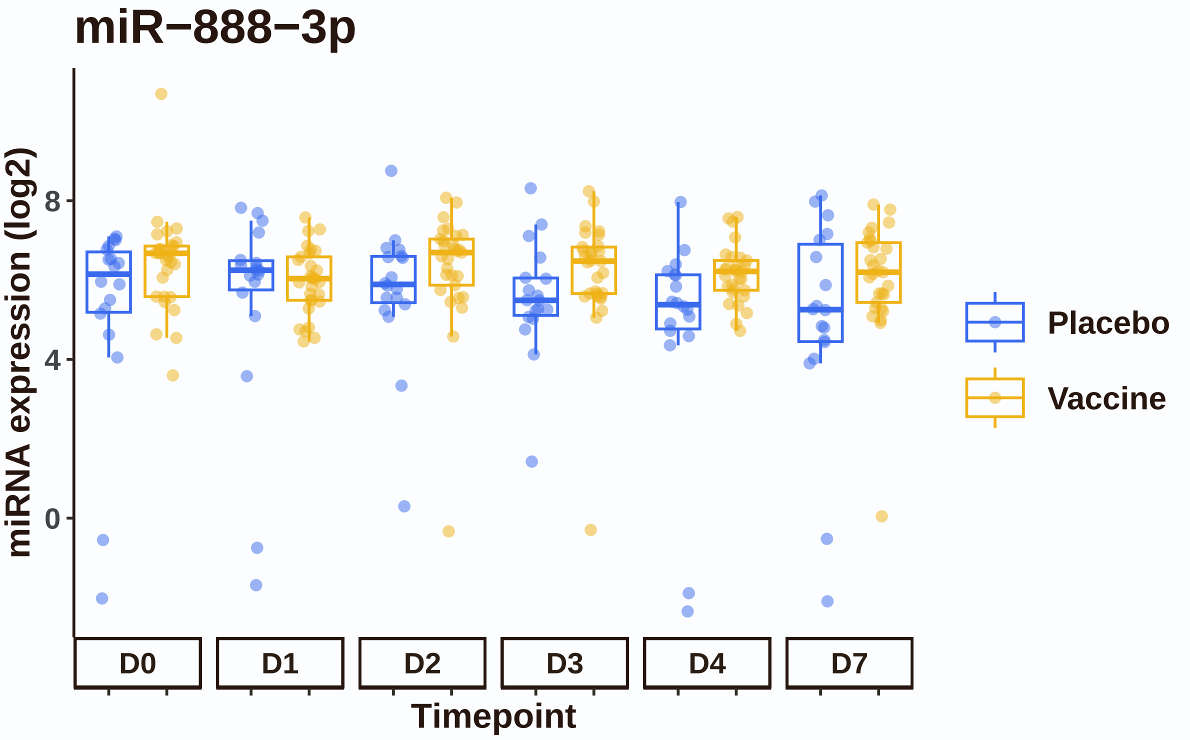
miR−888−3p
miRNA expression (log2)
8
4
0
D0
D1
D2
D3
D4
D7
Timepoint
Placebo
Vaccine

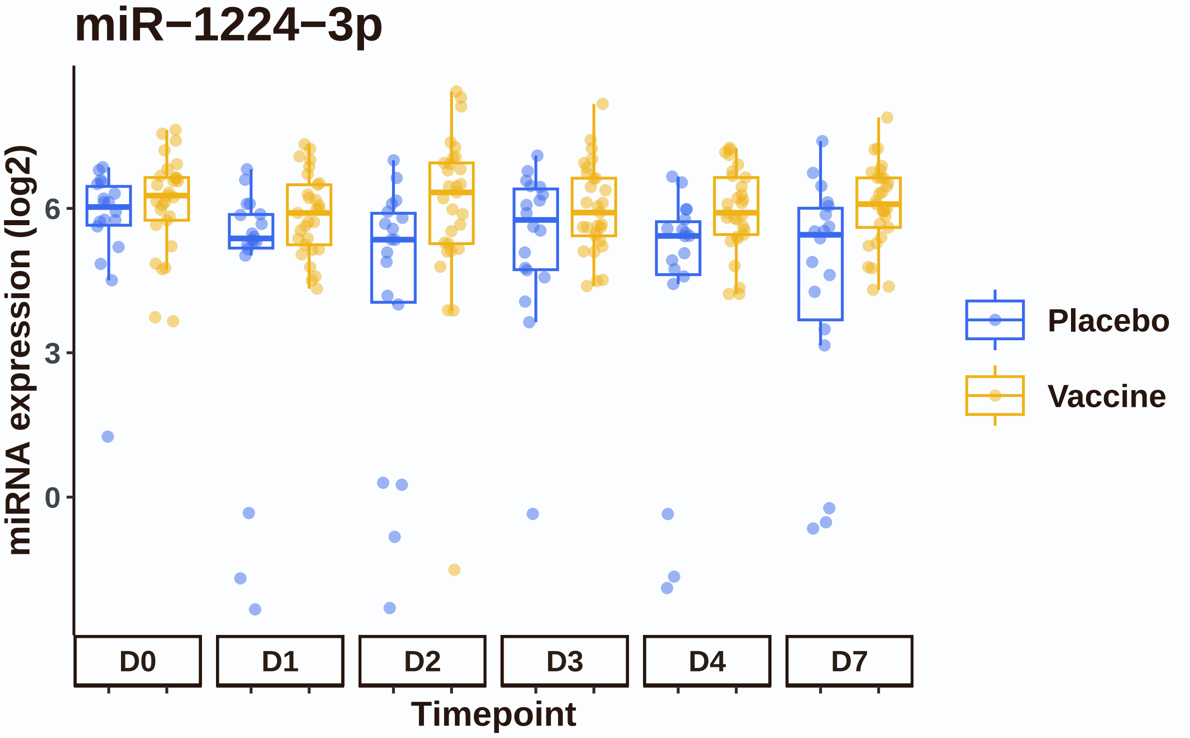

miR−1224−3p
miRNA expression (log2)
6
3
0
D0
D1
D2
D3
D4
D7
Timepoint
Placebo
Vaccine

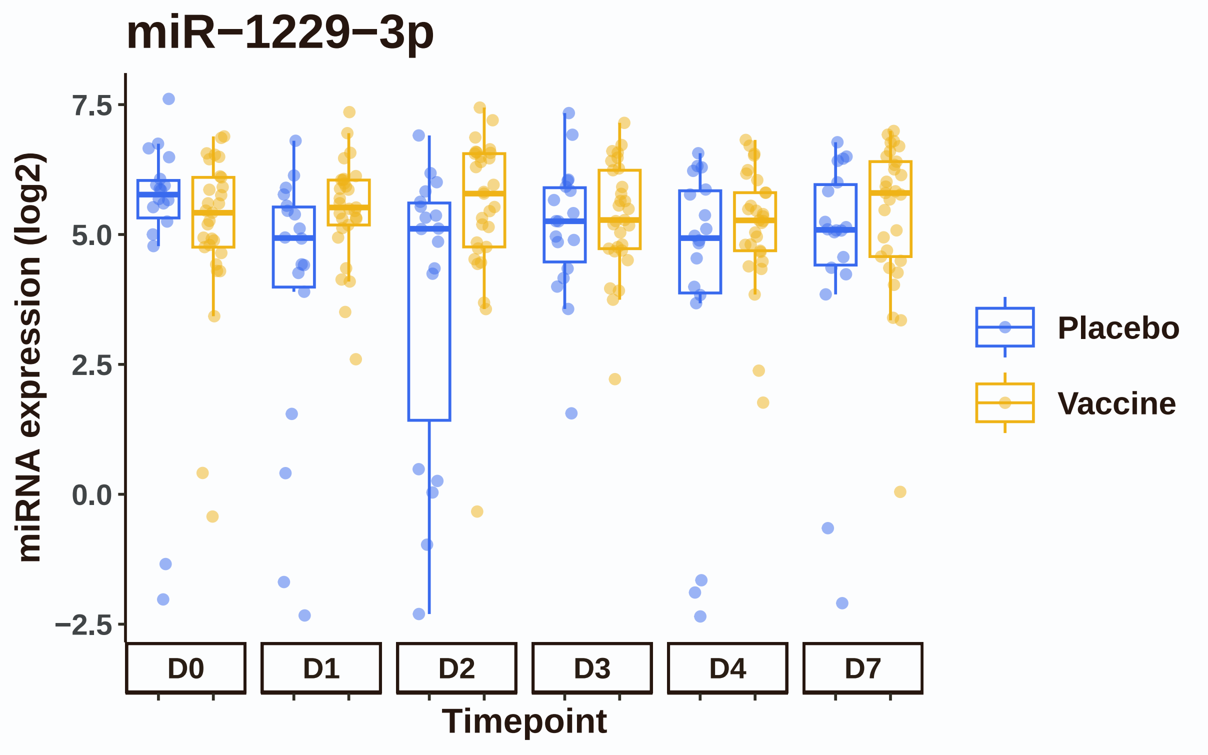
miR−1229−3p
7.5
5.0
2.5
0.0
−2.5
miRNA expression (log2)
D0
D1
D2
D3
D4
D7
Timepoint
Placebo
Vaccine

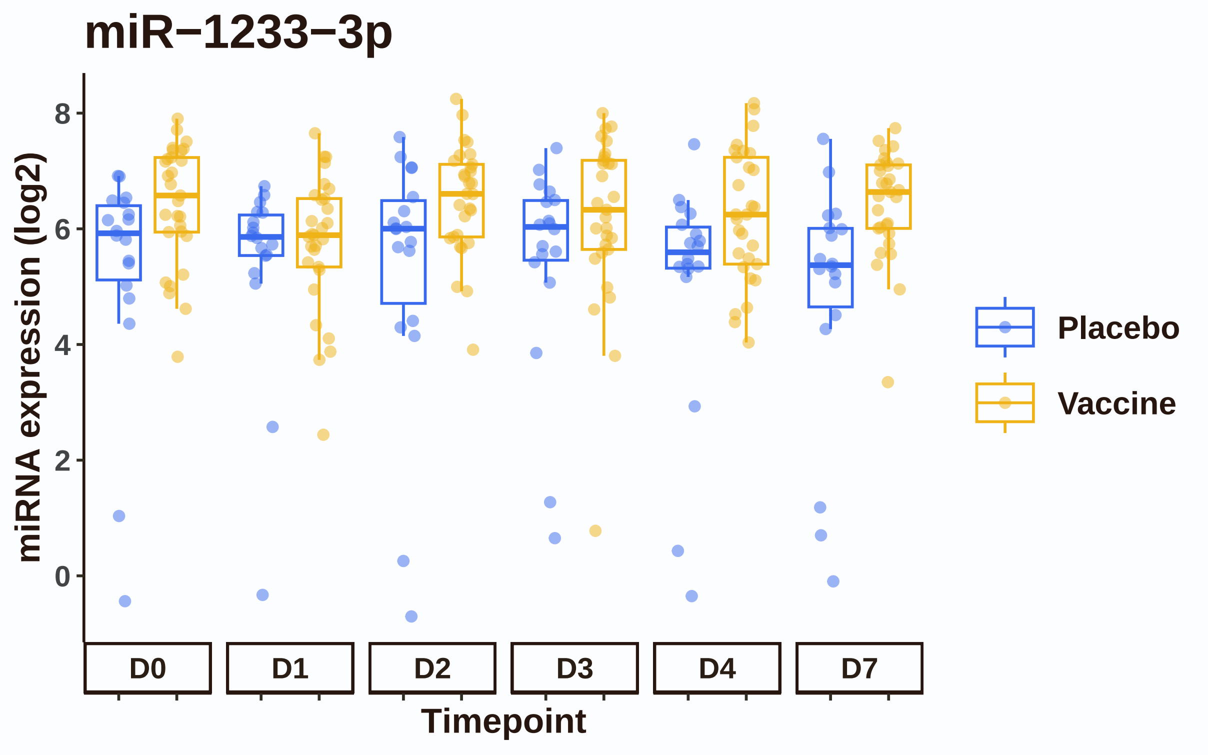
miR−1233−3p
miRNA expression (log2)
0
2
4
6
8
D0
D1
D2
D3
D4
D7
Timepoint
Placebo
Vaccine

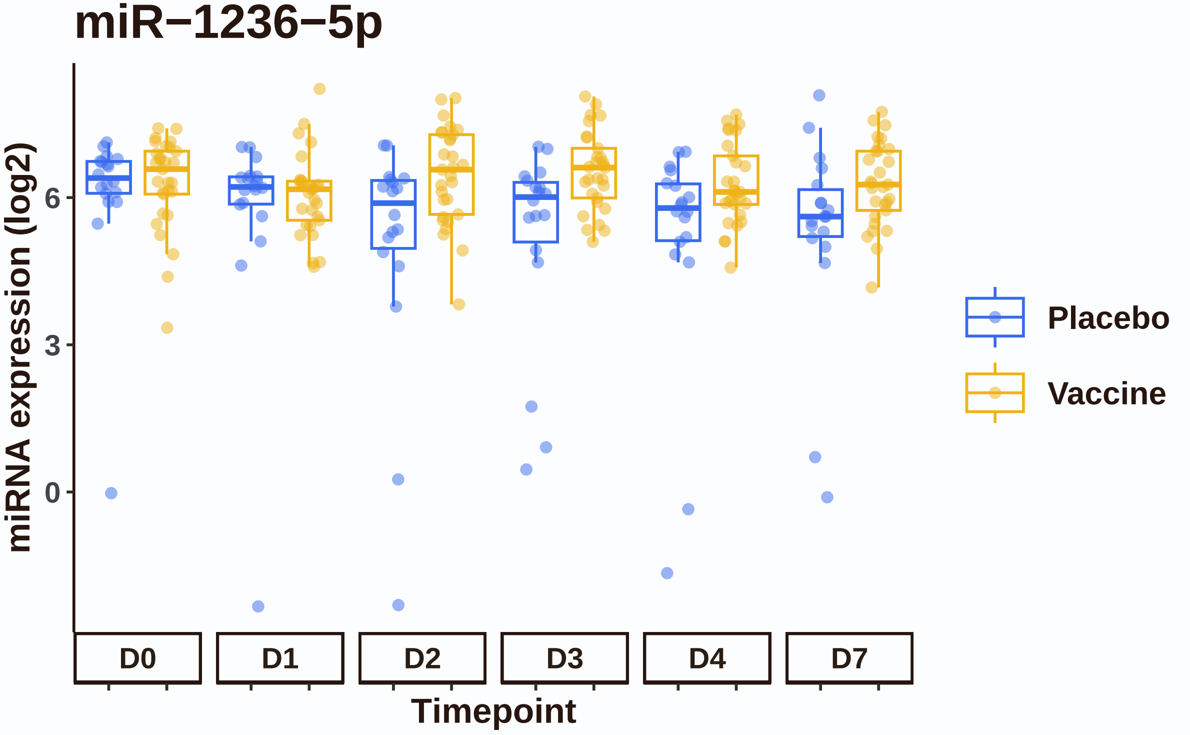
miR−1236−5p
miRNA expression (log2)
6
3
0
D0
D1
D2
D3
D4
D7
Timepoint
Placebo
Vaccine

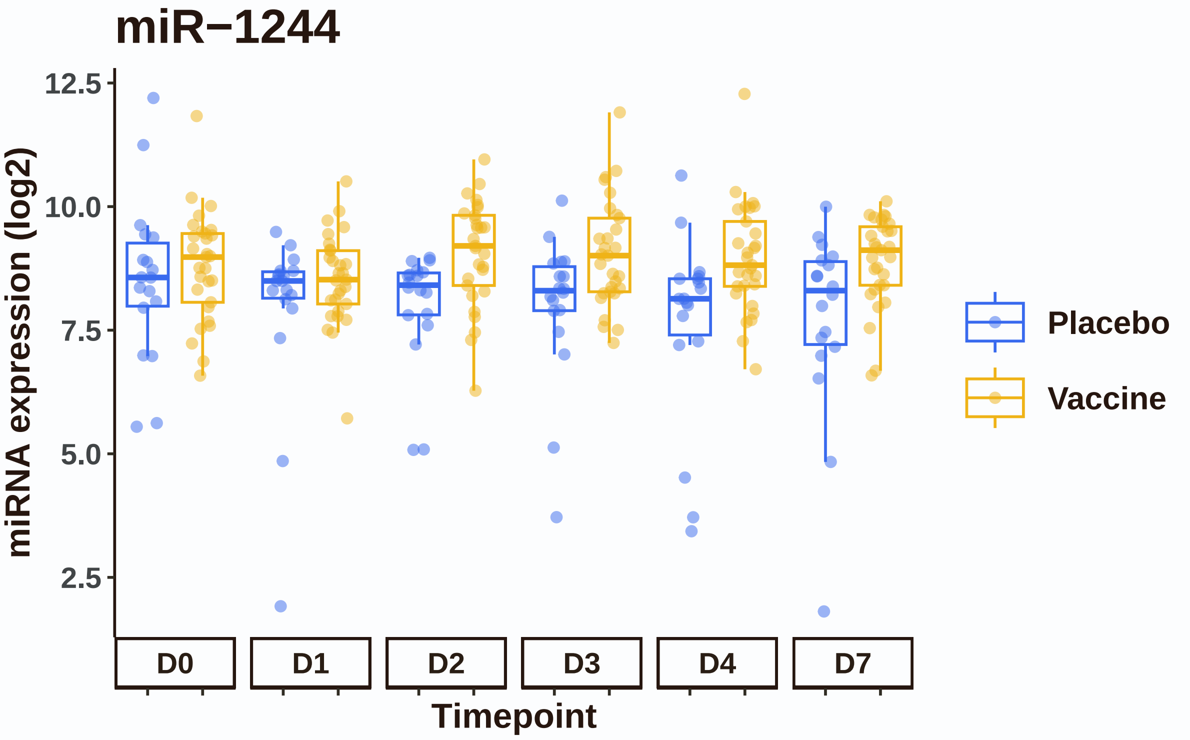

miR−1244
miRNA expression (log2)
12.5
10.0
7.5
5.0
2.5
D0
D1
D2
D3
D4
D7
Timepoint
Placebo
Vaccine

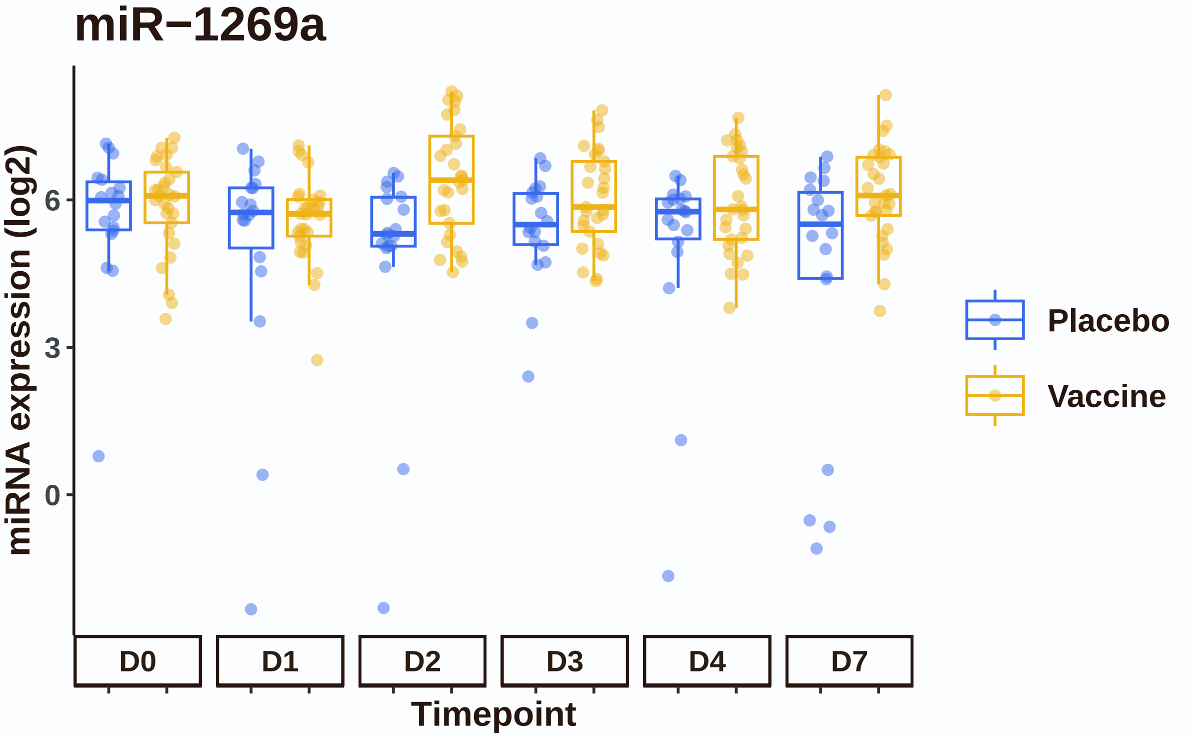

miR−1269a
miRNA expression (log2)
6
3
0
D0
D1
D2
D3
D4
D7
Timepoint
Placebo
Vaccine

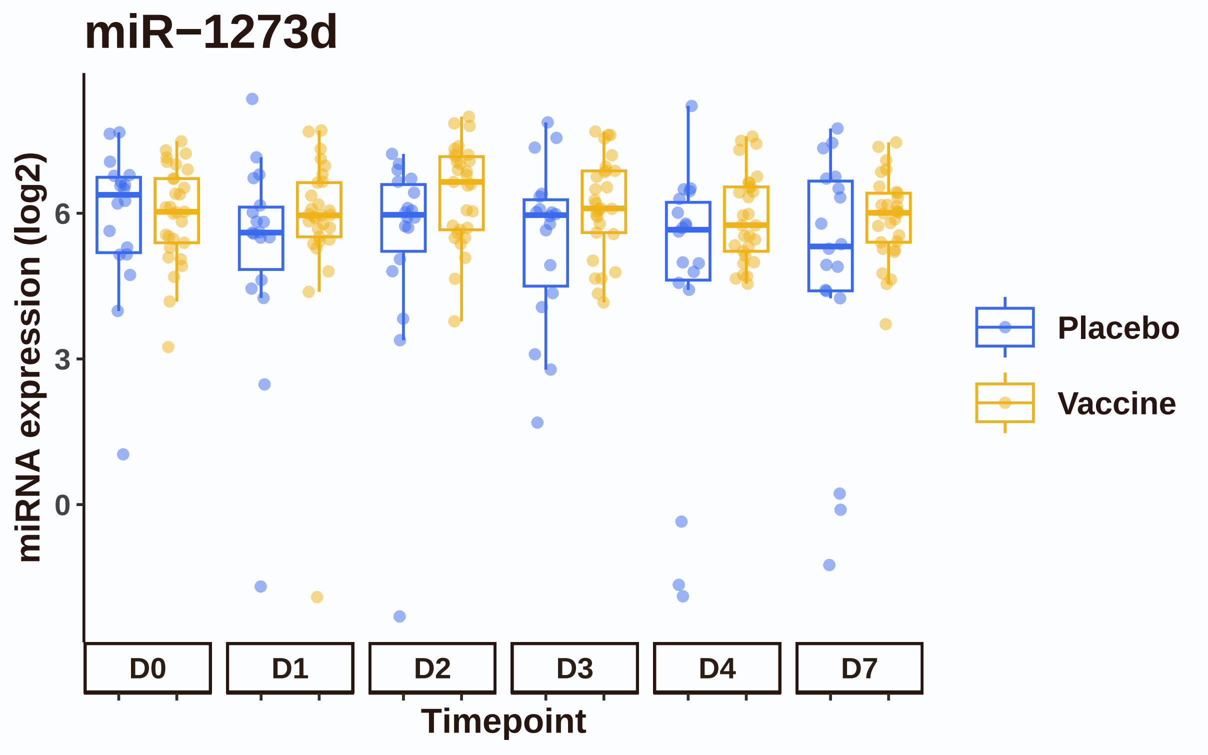
miR−1273d
miRNA expression (log2)
6
3
0
D0
D1
D2
D3
D4
D7
Timepoint
Placebo
Vaccine

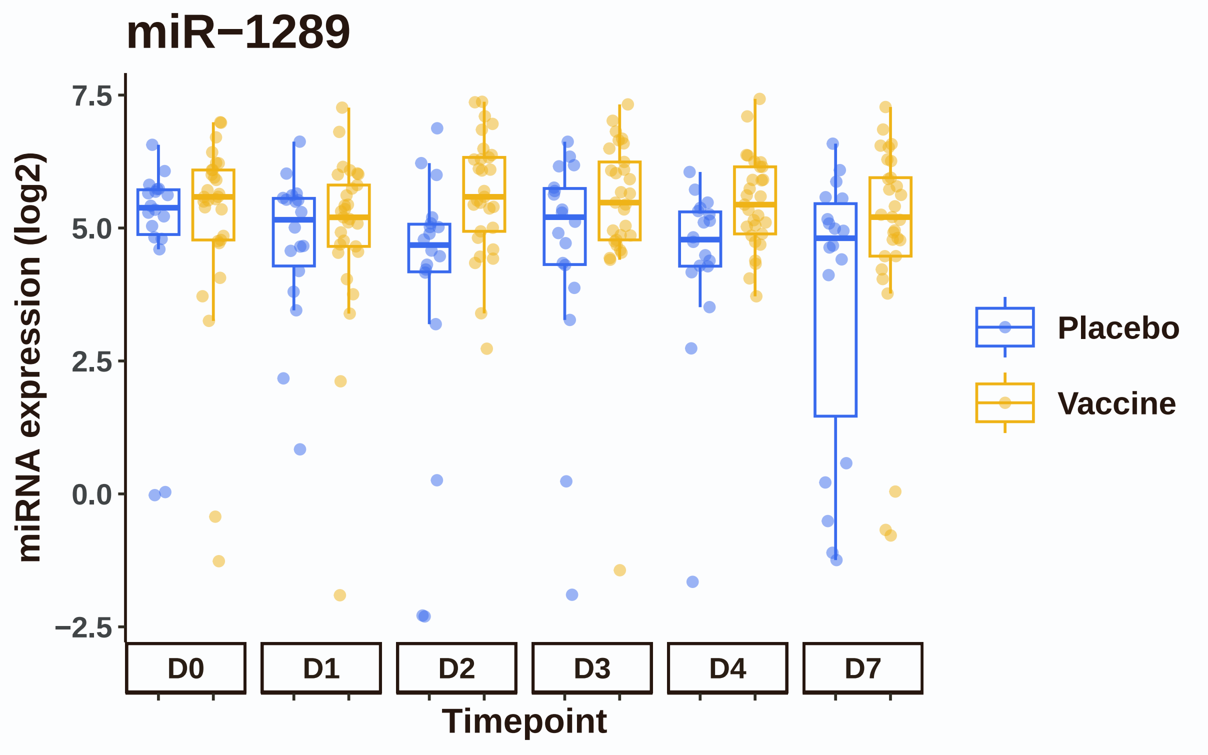

miR−1289
miRNA expression (log2)
7.5
5.0
2.5
0.0
−2.5
D0
D1
D2
D3
D4
D7
Timepoint
Placebo
Vaccine

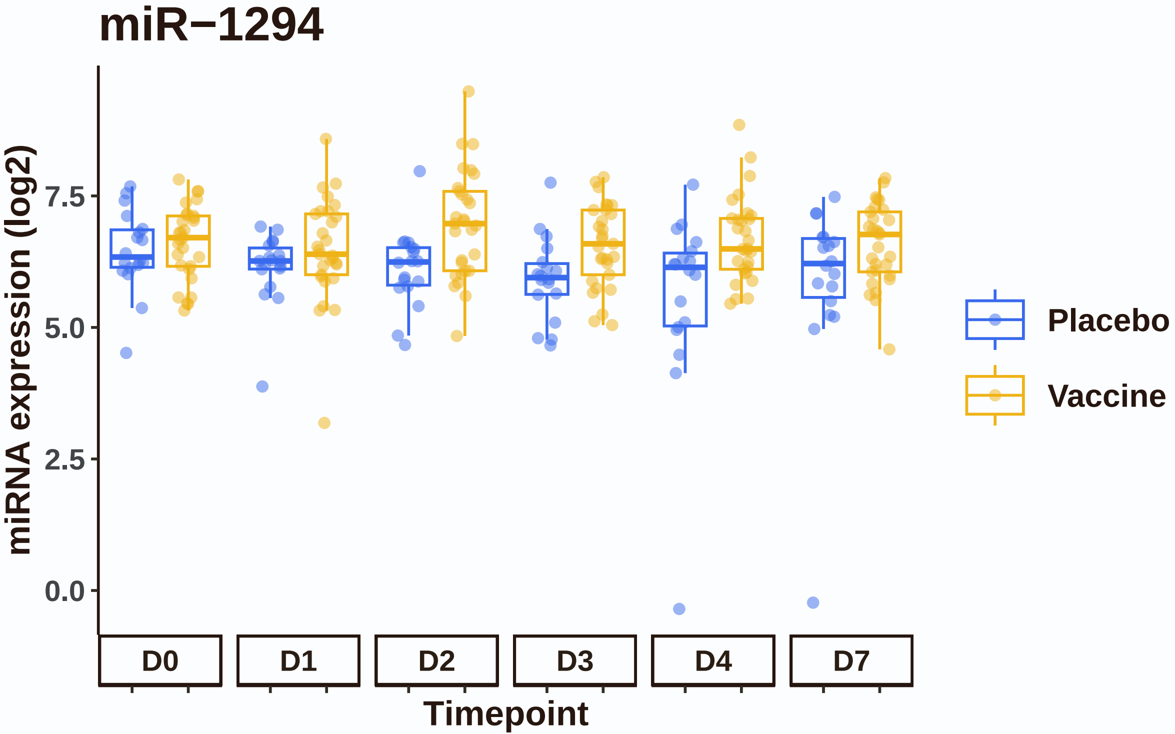
miR−1294
miRNA expression (log2)
7.5
5.0
2.5
0.0
D0
D1
D2
D3
D4
D7
Timepoint
Placebo
Vaccine

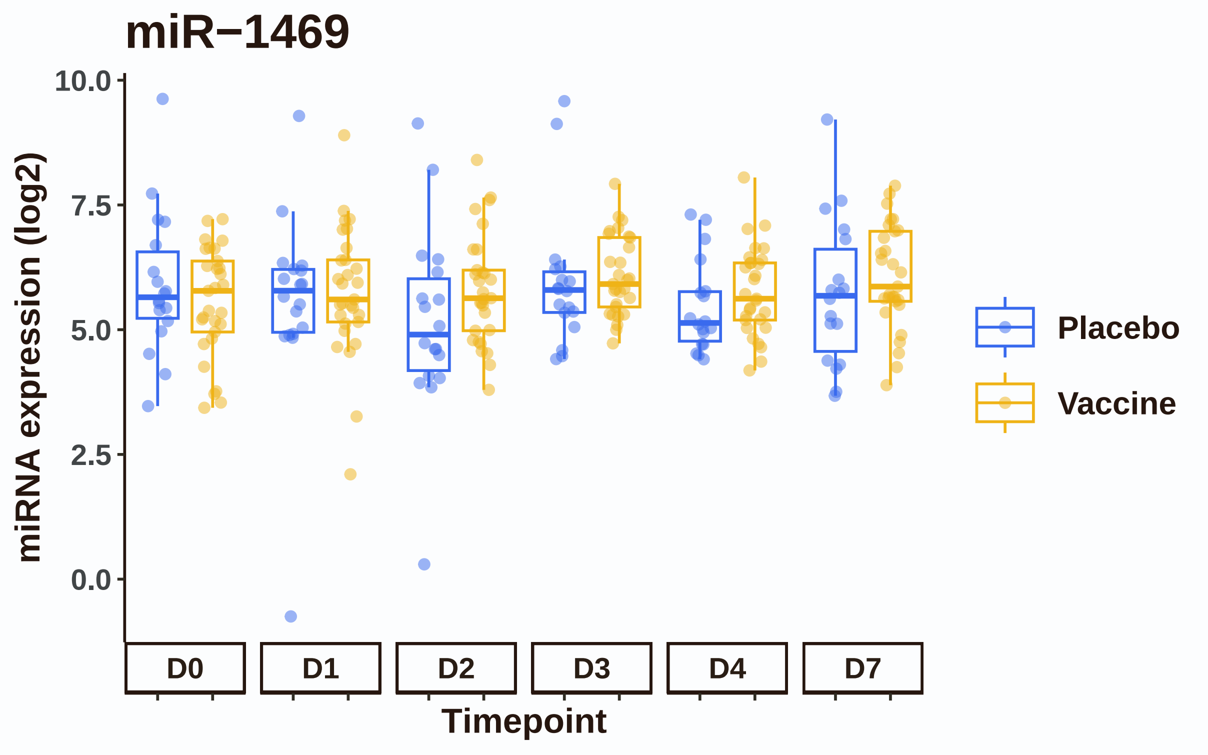
miR−1469
miRNA expression (log2)
10.0
7.5
5.0
2.5
0.0
D0
D1
D2
D3
D4
D7
Timepoint
Placebo
Vaccine

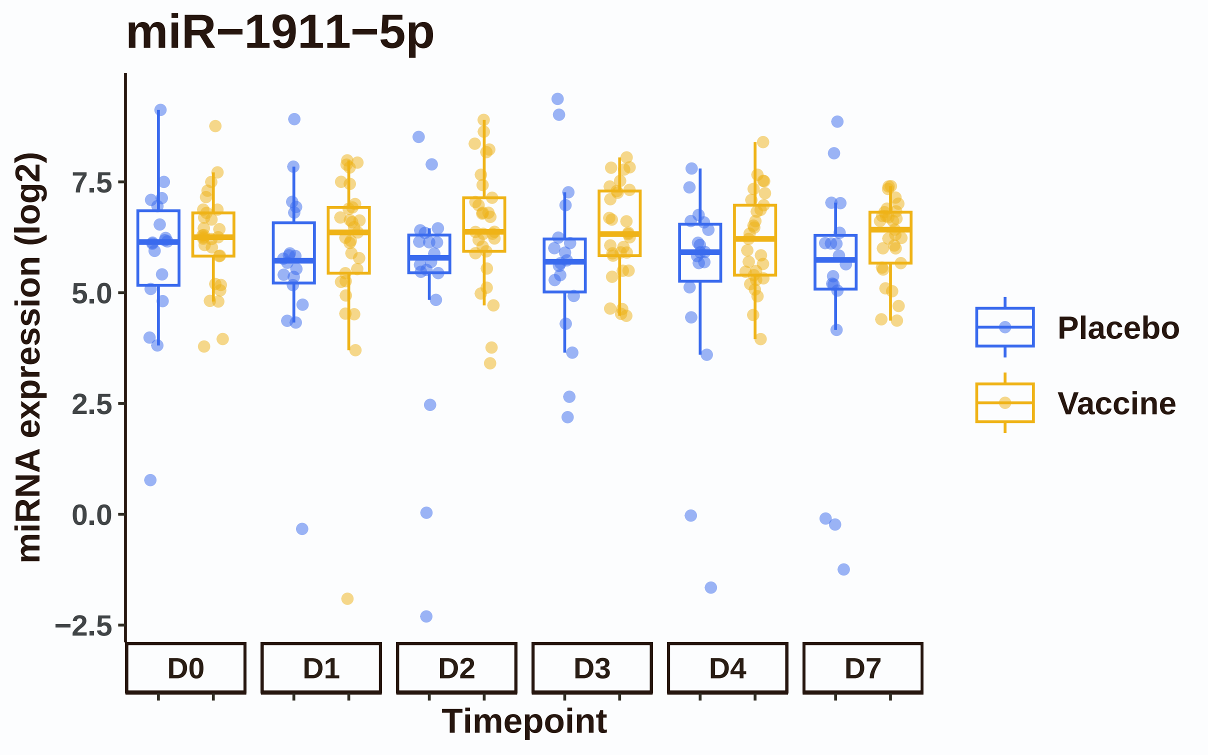
miR−1911−5p
miRNA expression (log2)
7.5
5.0
2.5
0.0
−2.5
D0
D1
D2
D3
D4
D7
Timepoint
Placebo
Vaccine

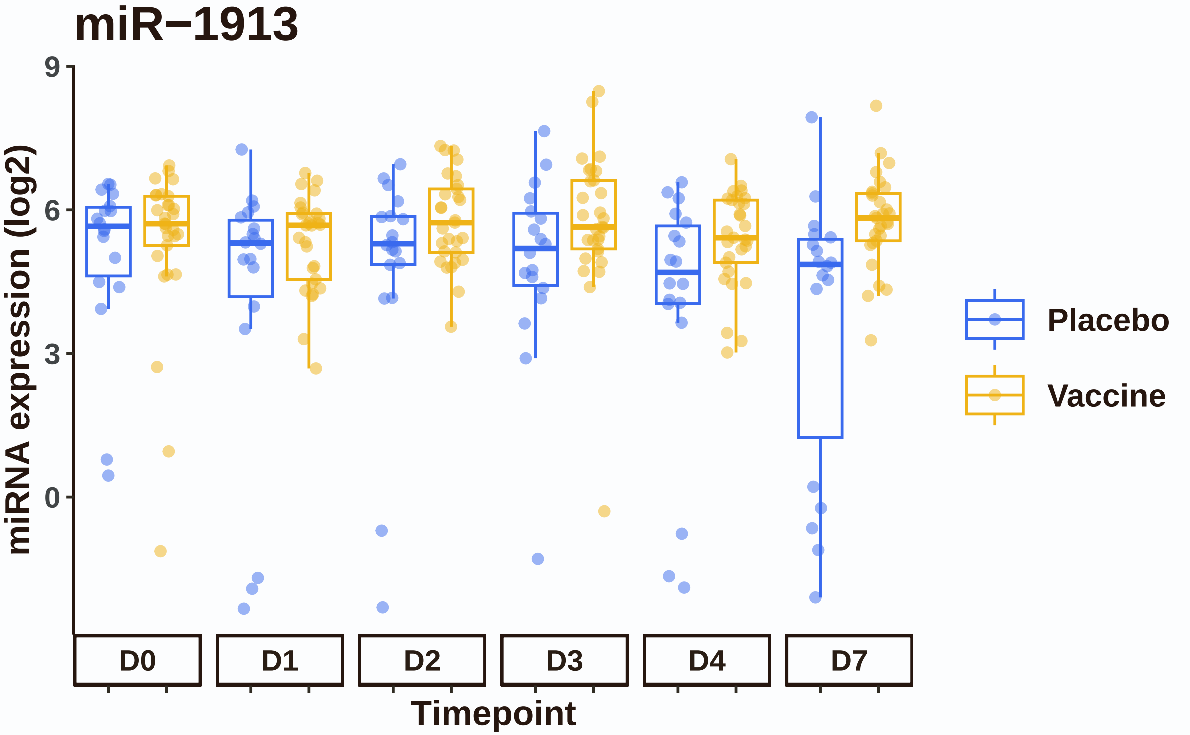

miR−1913
miRNA expression (log2)
9
6
3
0
D0
D1
D2
D3
D4
D7
Timepoint
Placebo
Vaccine

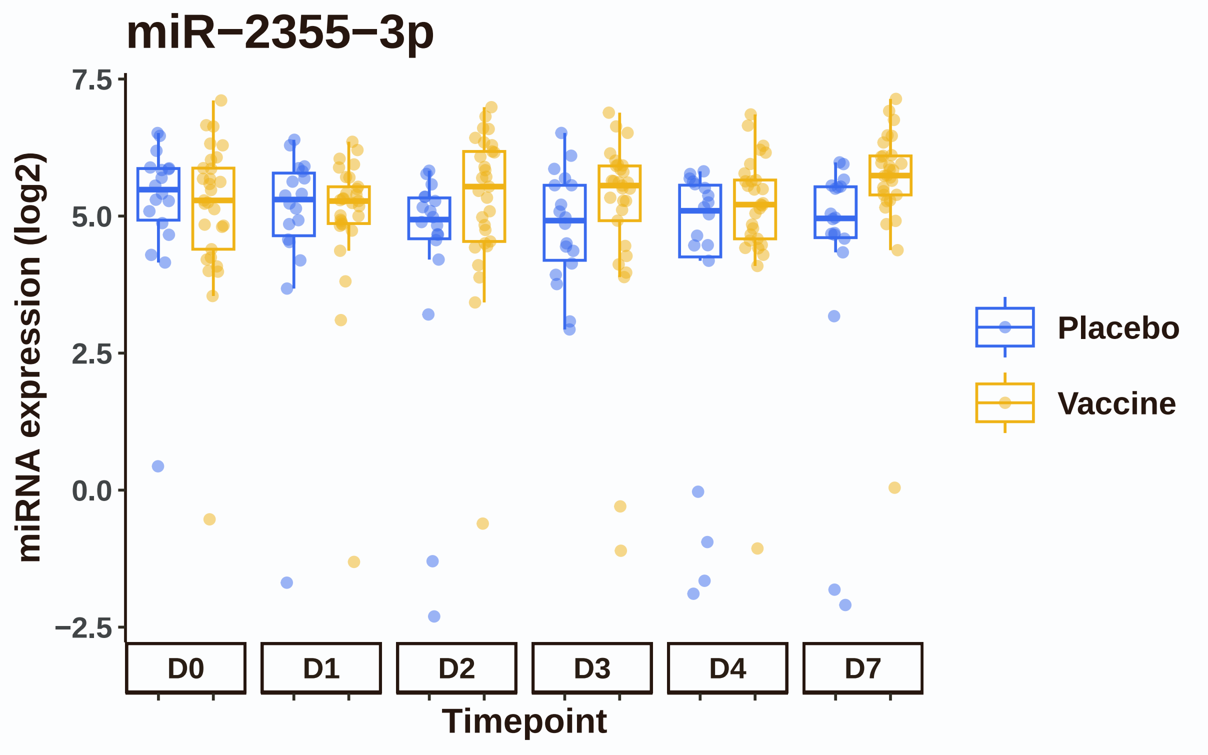
miR−2355−3p
7.5
5.0
2.5
0.0
−2.5
miRNA expression (log2)
D0
D1
D2
D3
D4
D7
Timepoint
Placebo
Vaccine

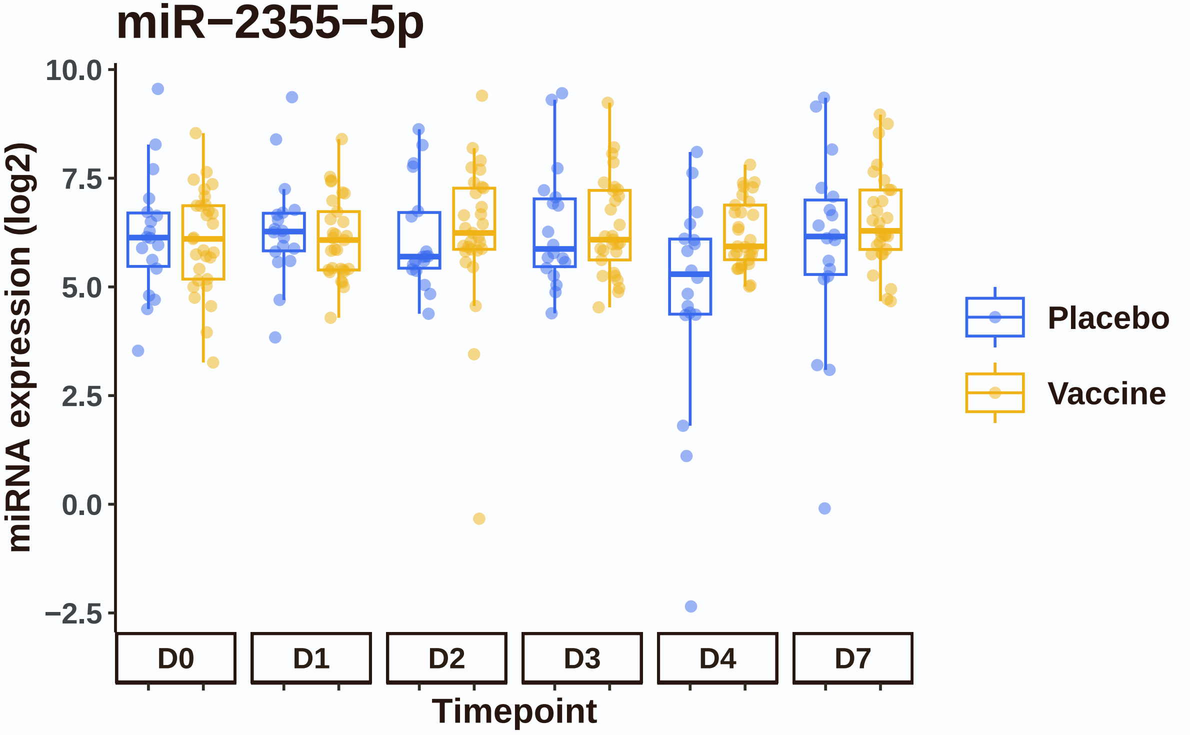
miR−2355−5p
miRNA expression (log2)
10.0
7.5
5.0
2.5
0.0
−2.5
D0
D1
D2
D3
D4
D7
Timepoint
Placebo
Vaccine

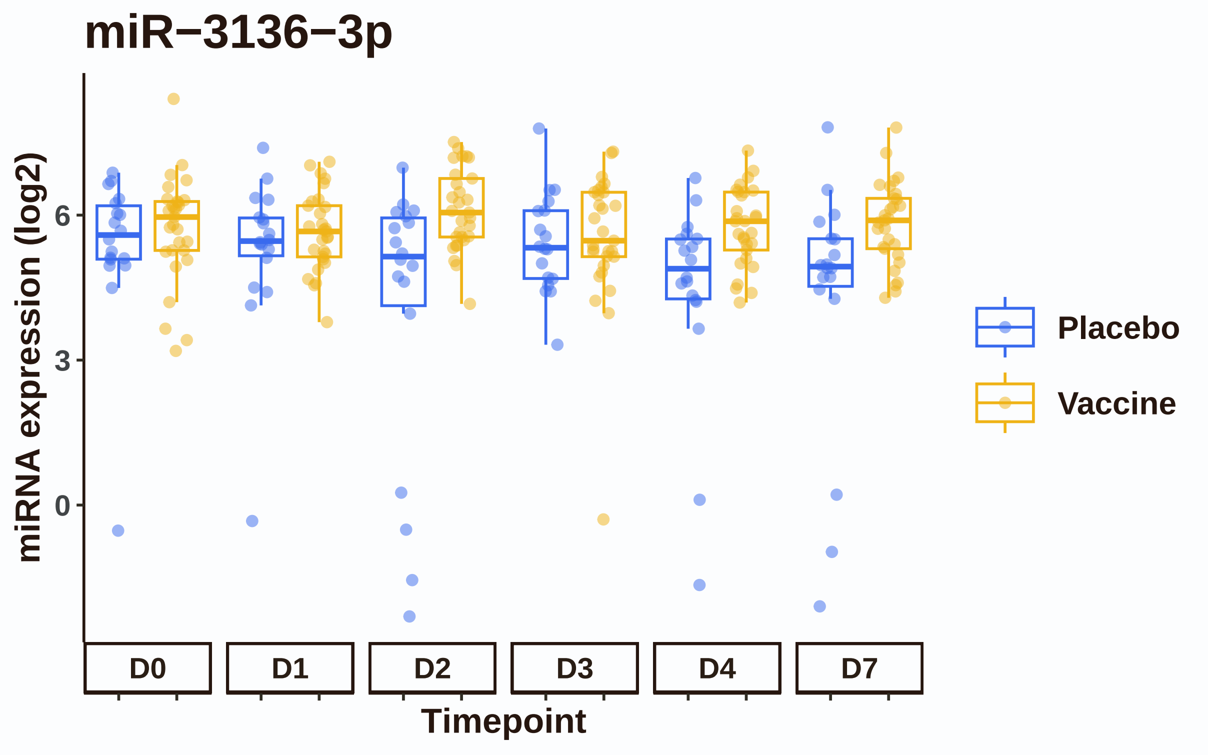
miR−3136−3p
miRNA expression (log2)
6
3
0
D0
D1
D2
D3
D4
D7
Timepoint
Placebo
Vaccine

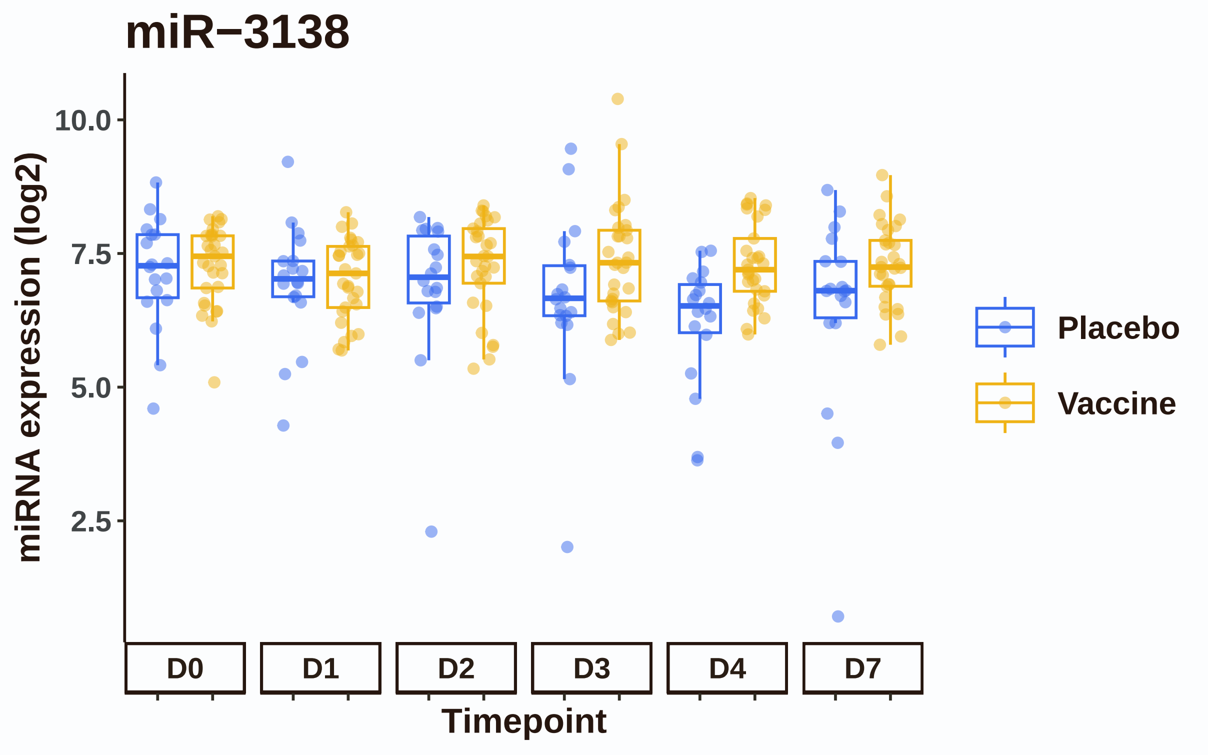
miR−3138
miRNA expression (log2)
10.0
7.5
5.0
2.5
D0
D1
D2
D3
D4
D7
Timepoint
Placebo
Vaccine

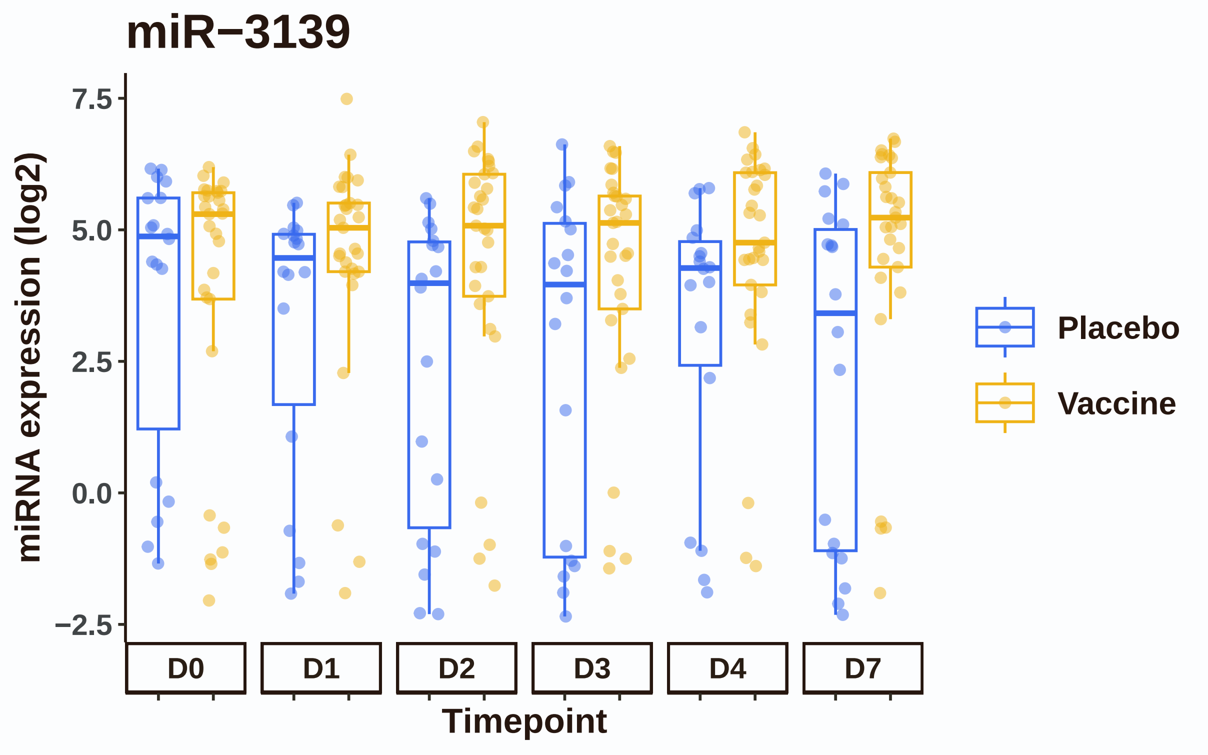
miR−3139
miRNA expression (log2)
7.5
5.0
2.5
0.0
−2.5
D0
D1
D2
D3
D4
D7
Timepoint
Placebo
Vaccine

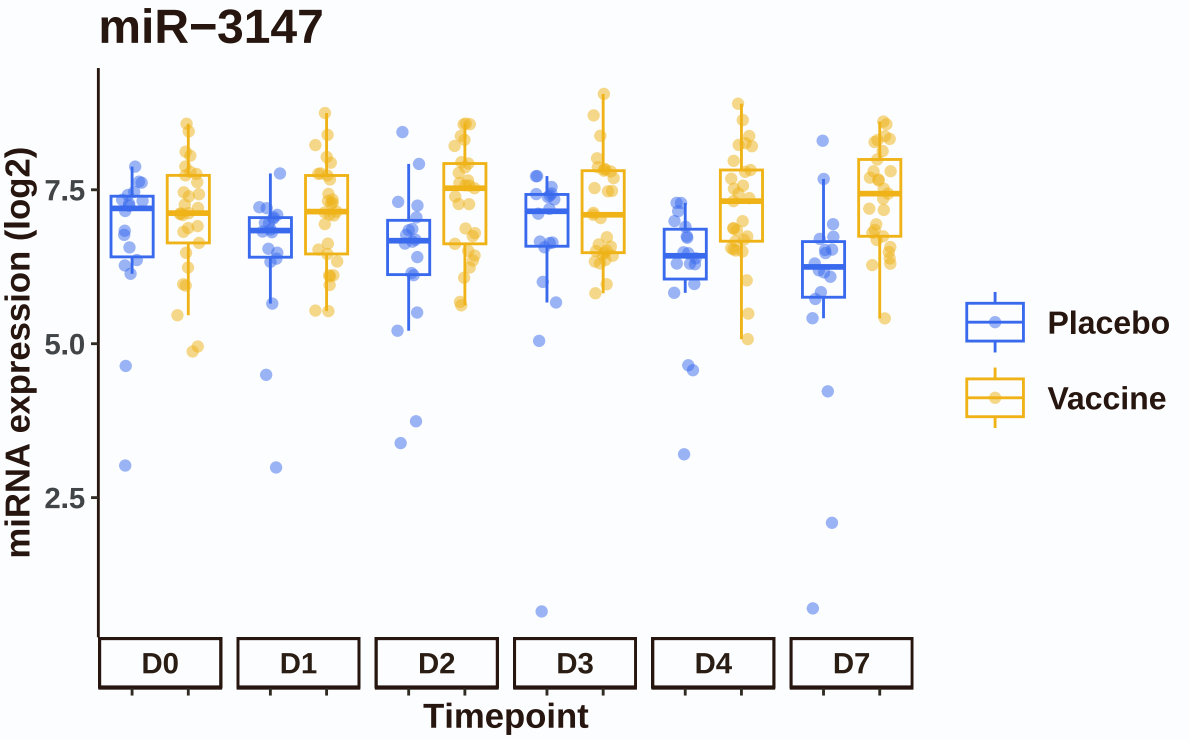
miR−3147
miRNA expression (log2)
7.5
5.0
2.5
D0
D1
D2
D3
D4
D7
Timepoint
Placebo
Vaccine

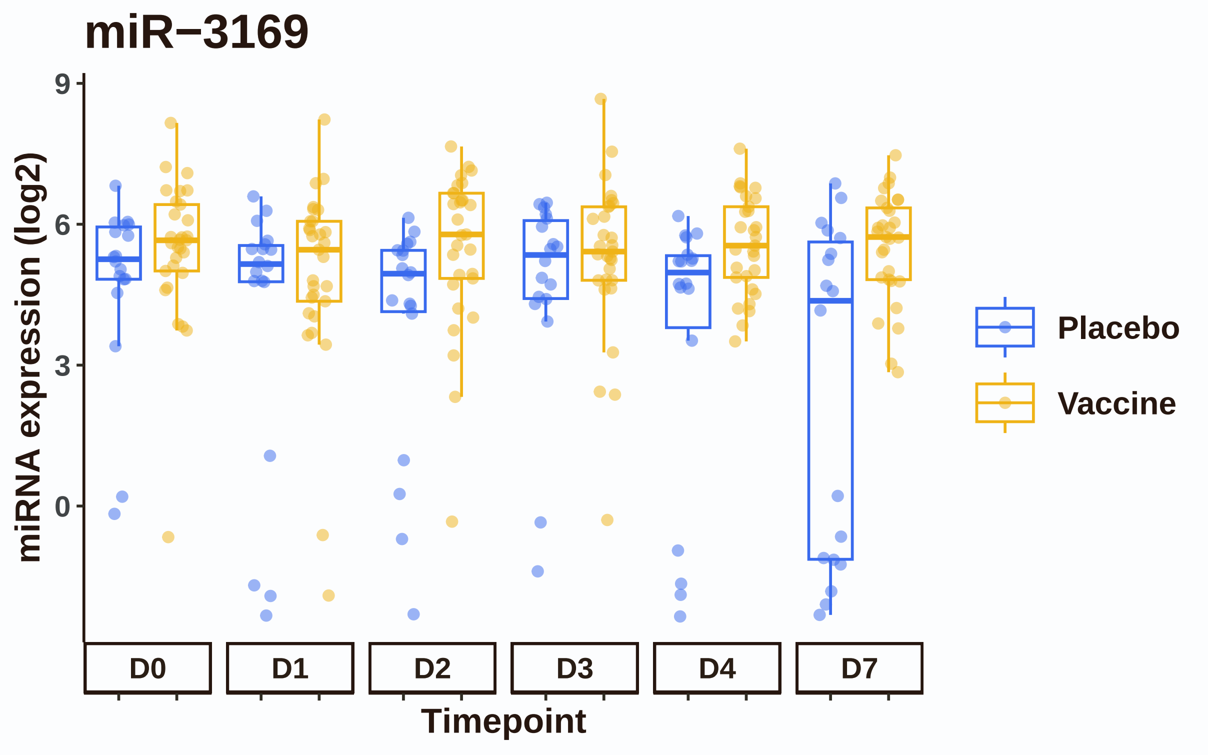
miR−3169
miRNA expression (log2)
9
6
3
0
D0
D1
D2
D3
D4
D7
Timepoint
Placebo
Vaccine

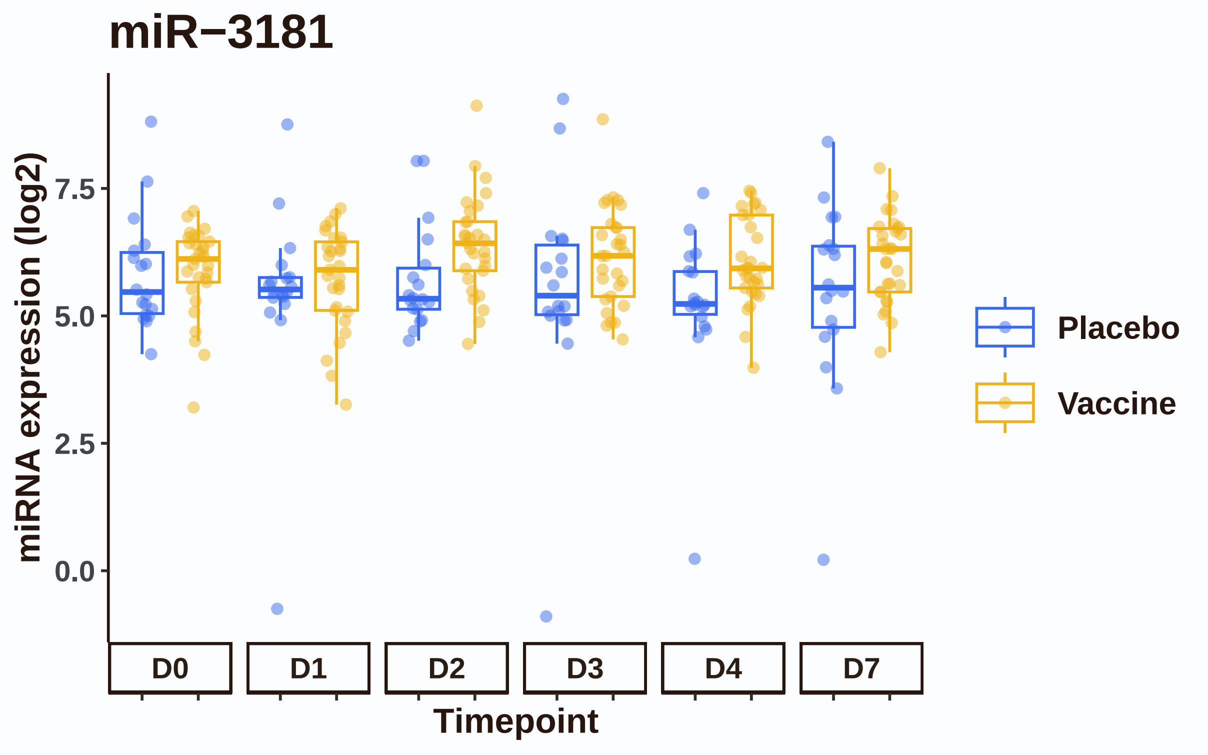

miR−3181
miRNA expression (log2)
7.5
5.0
2.5
0.0
D0
D1
D2
D3
D4
D7
Timepoint
Placebo
Vaccine

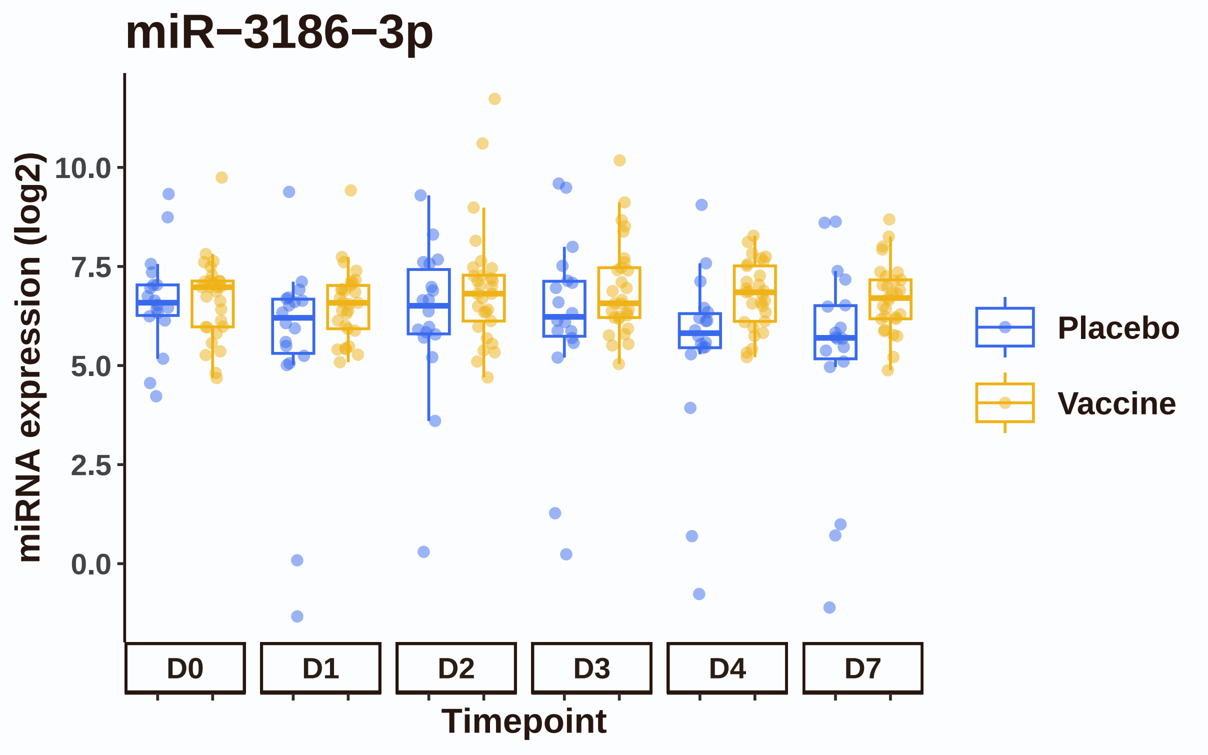
miR−3186−3p
miRNA expression (log2)
10.0
7.5
5.0
2.5
0.0
D0
D1
D2
D3
D4
D7
Timepoint
Placebo
Vaccine

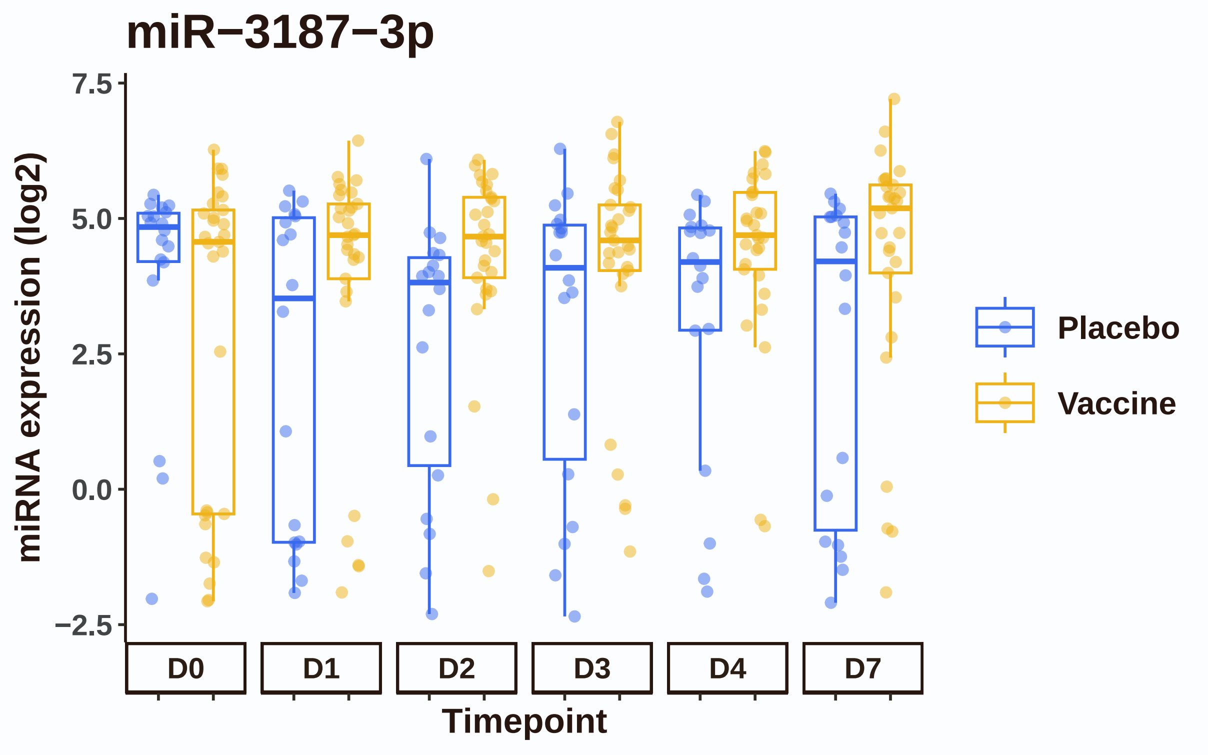

miR−3187−3p
miRNA expression (log2)
7.5
5.0
2.5
0.0
−2.5
D0
D1
D2
D3
D4
D7
Timepoint
Placebo
Vaccine

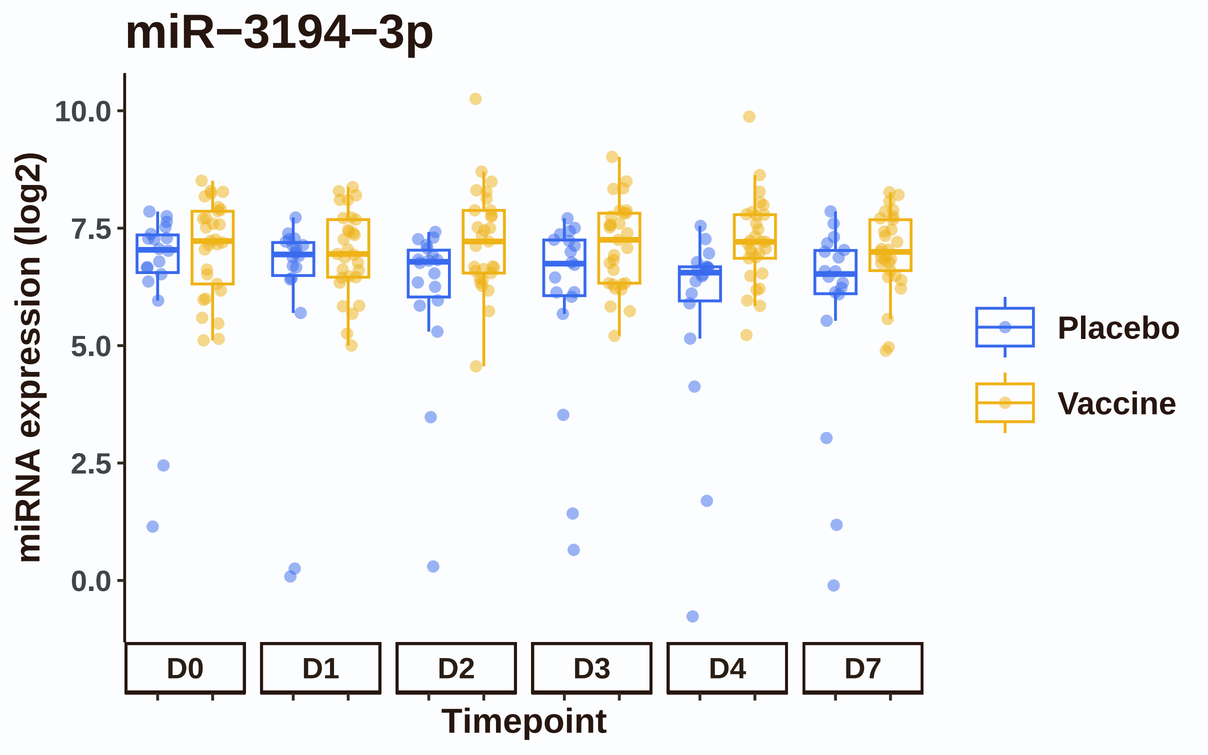
miR−3194−3p
miRNA expression (log2)
10.0
7.5
5.0
2.5
0.0
D0
D1
D2
D3
D4
D7
Timepoint
Placebo
Vaccine

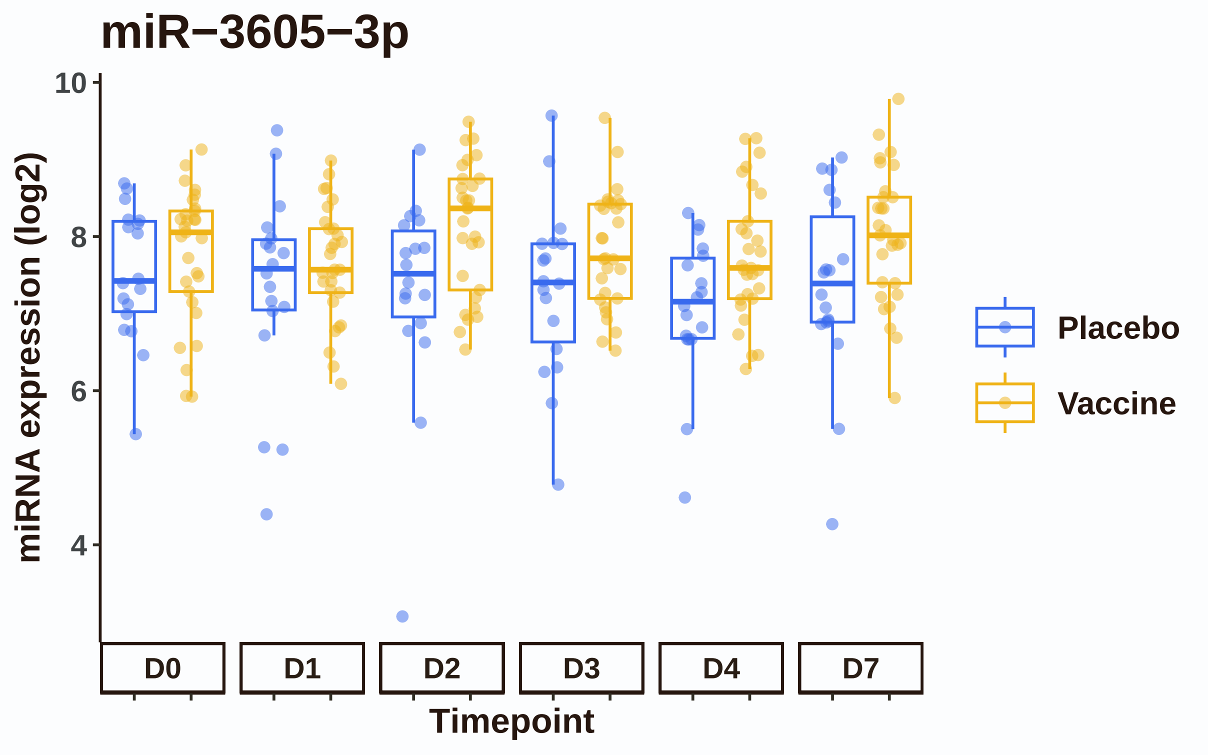
miR−3605−3p
miRNA expression (log2)
10
8
6
4
D0
D1
D2
D3
D4
D7
Timepoint
Placebo
Vaccine

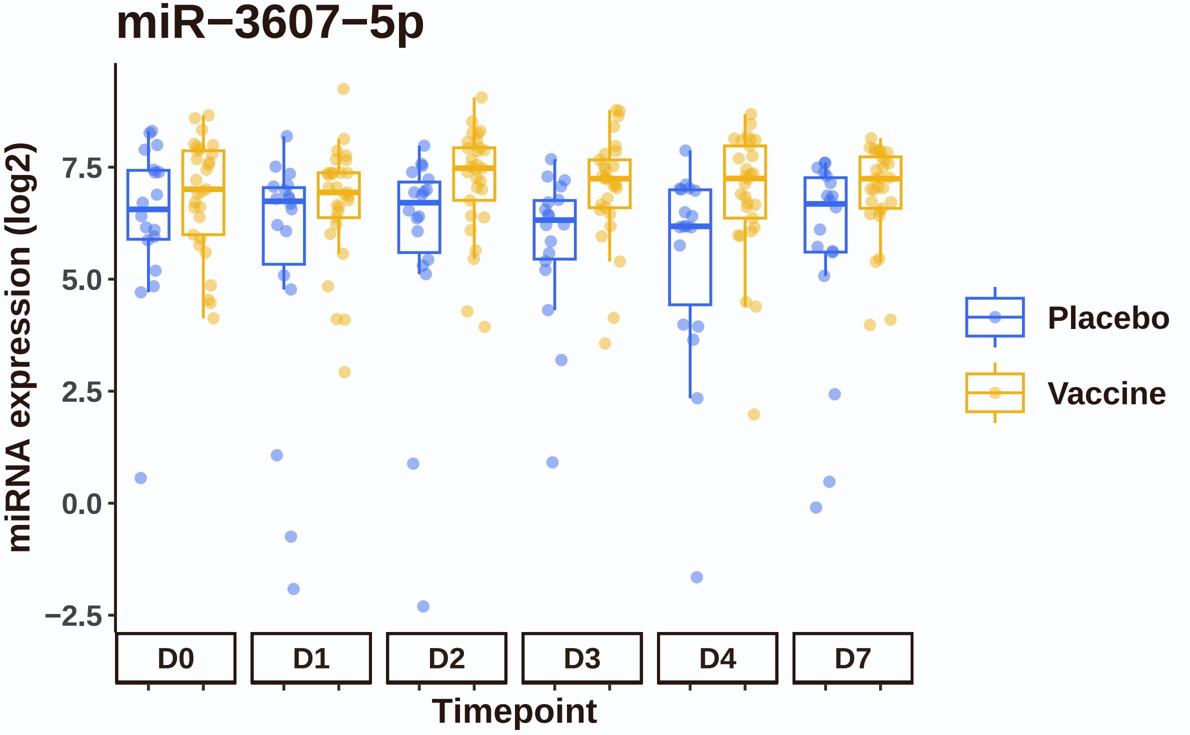
miR−3607−5p
miRNA expression (log2)
7.5
5.0
2.5
0.0
−2.5
D0
D1
D2
D3
D4
D7
Timepoint
Placebo
Vaccine

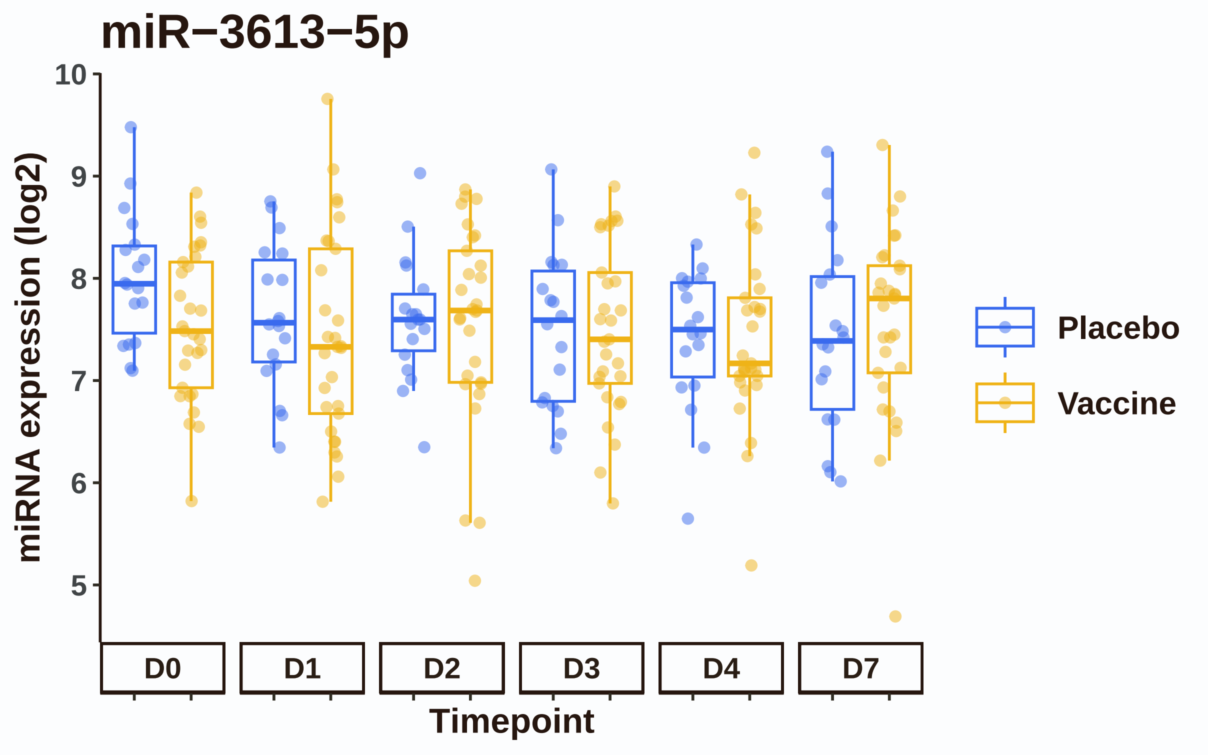

miR−3613−5p
miRNA expression (log2)
10
9
8
7
6
5
D0
D1
D2
D3
D4
D7
Timepoint
Placebo
Vaccine

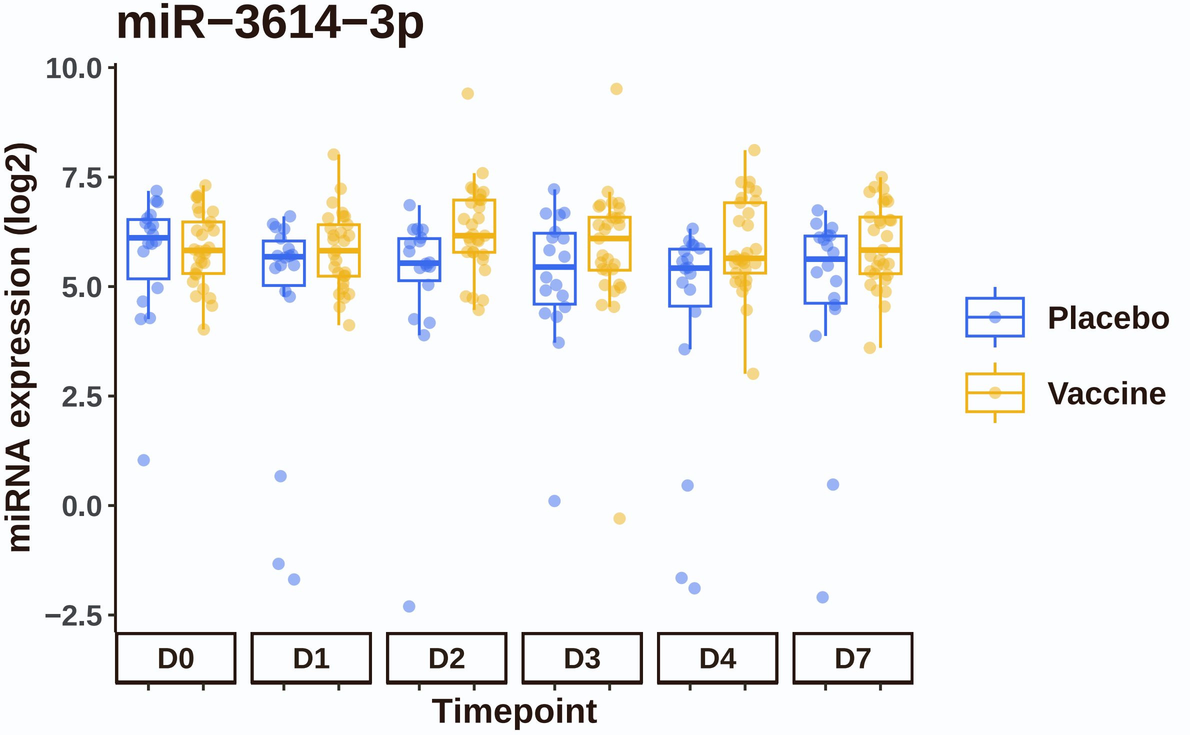
miR−3614−3p
miRNA expression (log2)
10.0
7.5
5.0
2.5
0.0
−2.5
D0
D1
D2
D3
D4
D7
Timepoint
Placebo
Vaccine

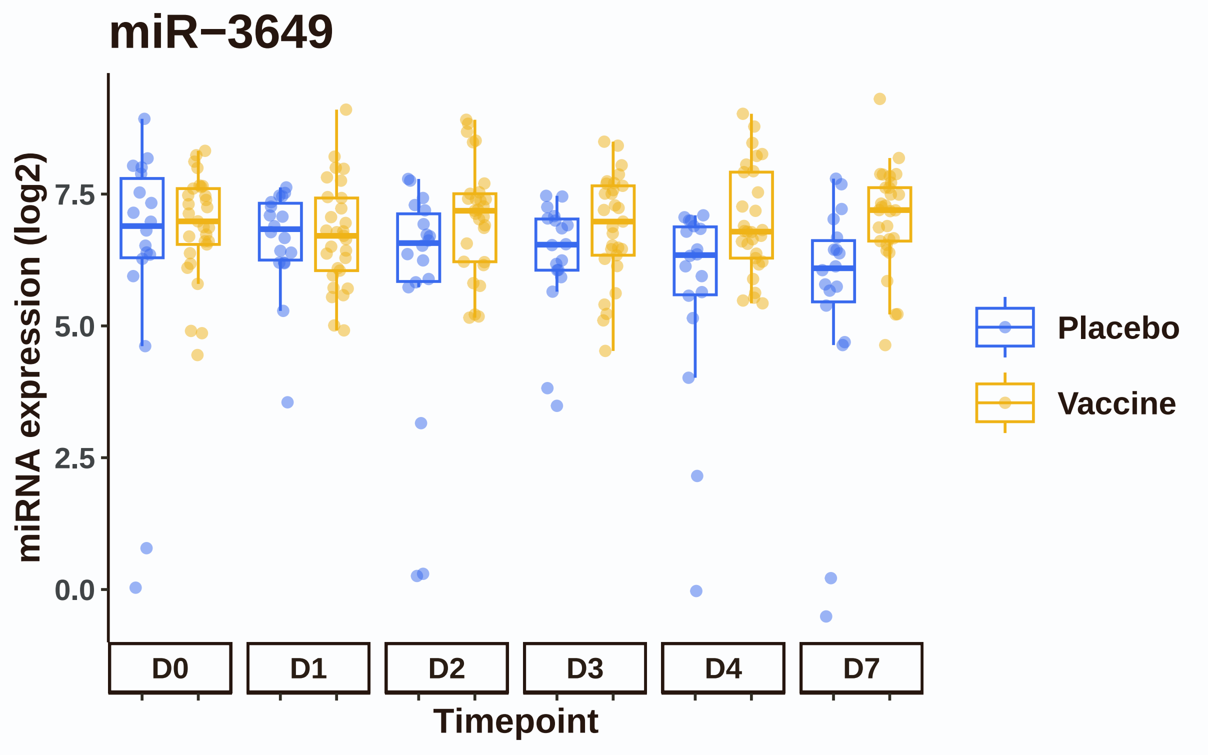
miR−3649
miRNA expression (log2)
7.5
5.0
2.5
0.0
D0
D1
D2
D3
D4
D7
Timepoint
Placebo
Vaccine

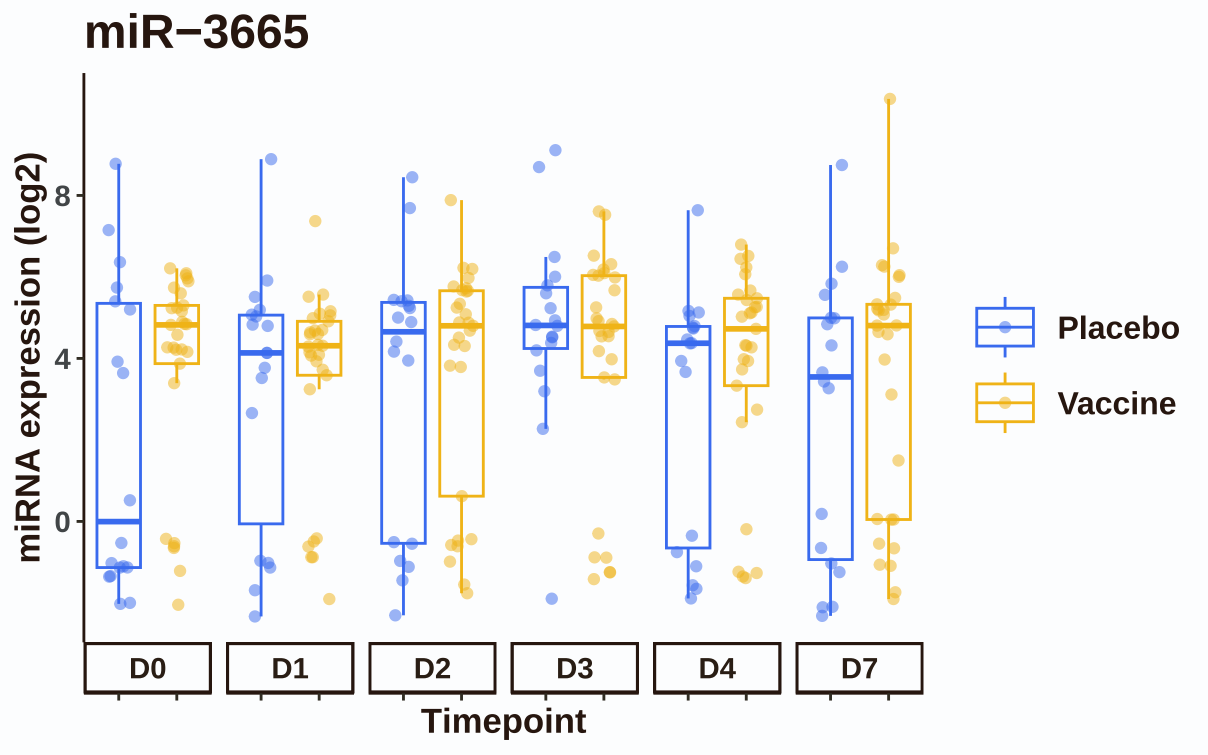

miR−3665
miRNA expression (log2)
8
4
0
D0
D1
D2
D3
D4
D7
Timepoint
Placebo
Vaccine

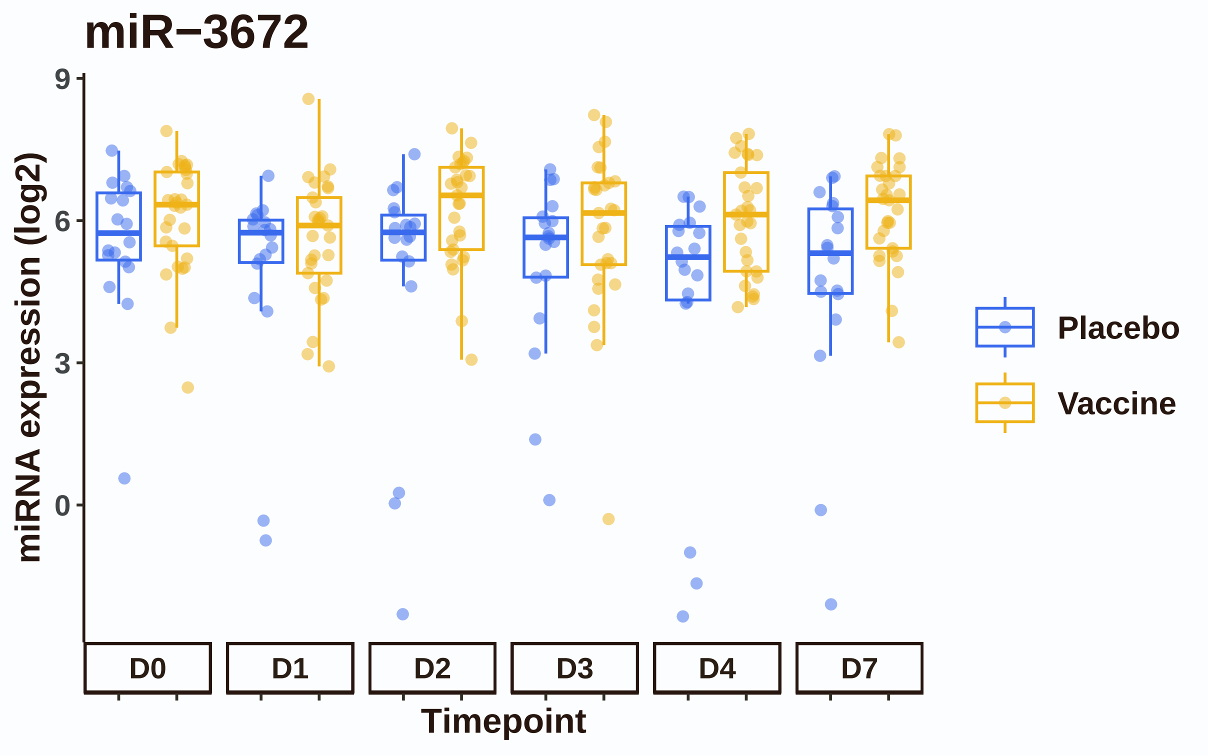

miR−3672
miRNA expression (log2)
9
6
3
0
D0
D1
D2
D3
D4
D7
Timepoint
Placebo
Vaccine

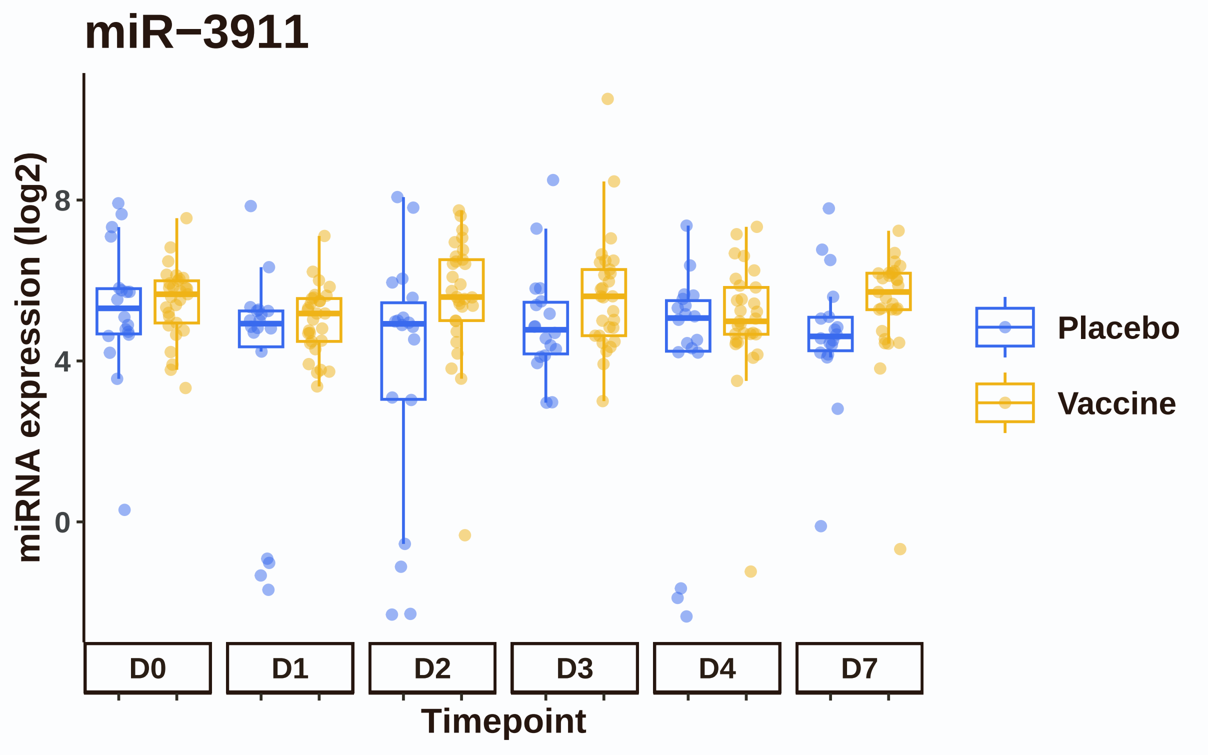
miR−3911
miRNA expression (log2)
8
4
0
D0
D1
D2
D3
D4
D7
Timepoint
Placebo
Vaccine

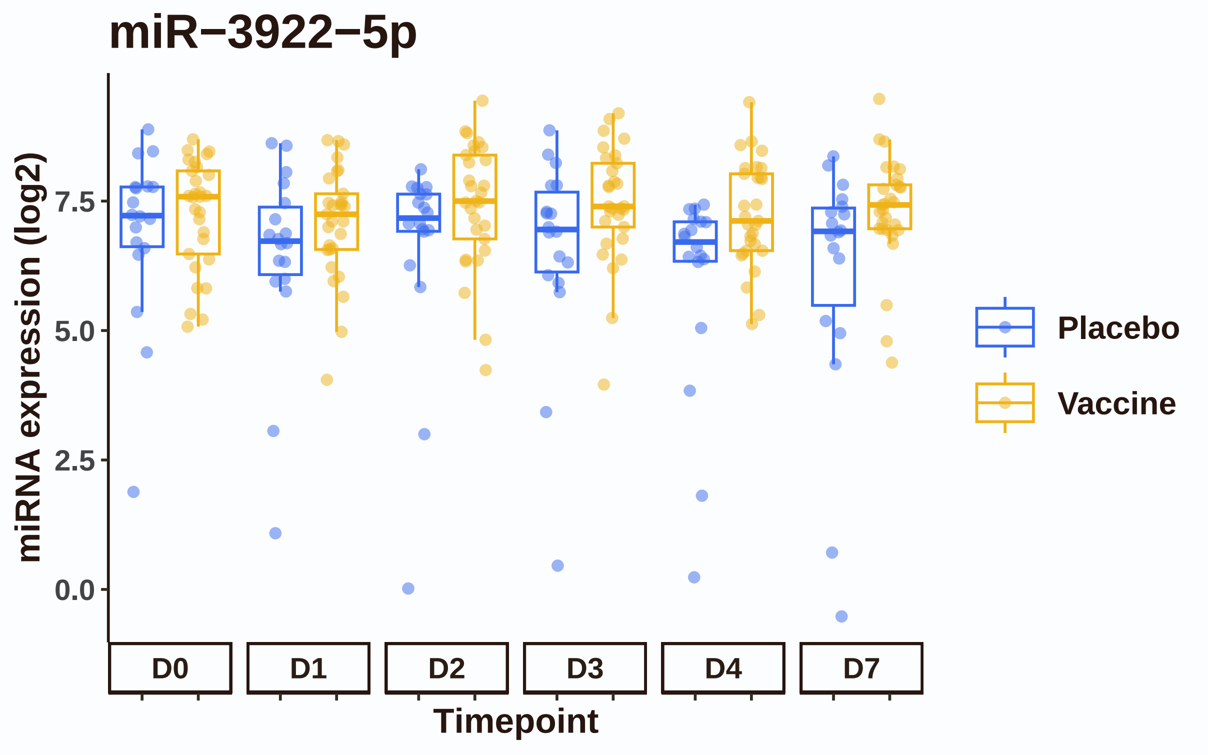
miR−3922−5p
miRNA expression (log2)
7.5
5.0
2.5
0.0
D0
D1
D2
D3
D4
D7
Timepoint
Placebo
Vaccine

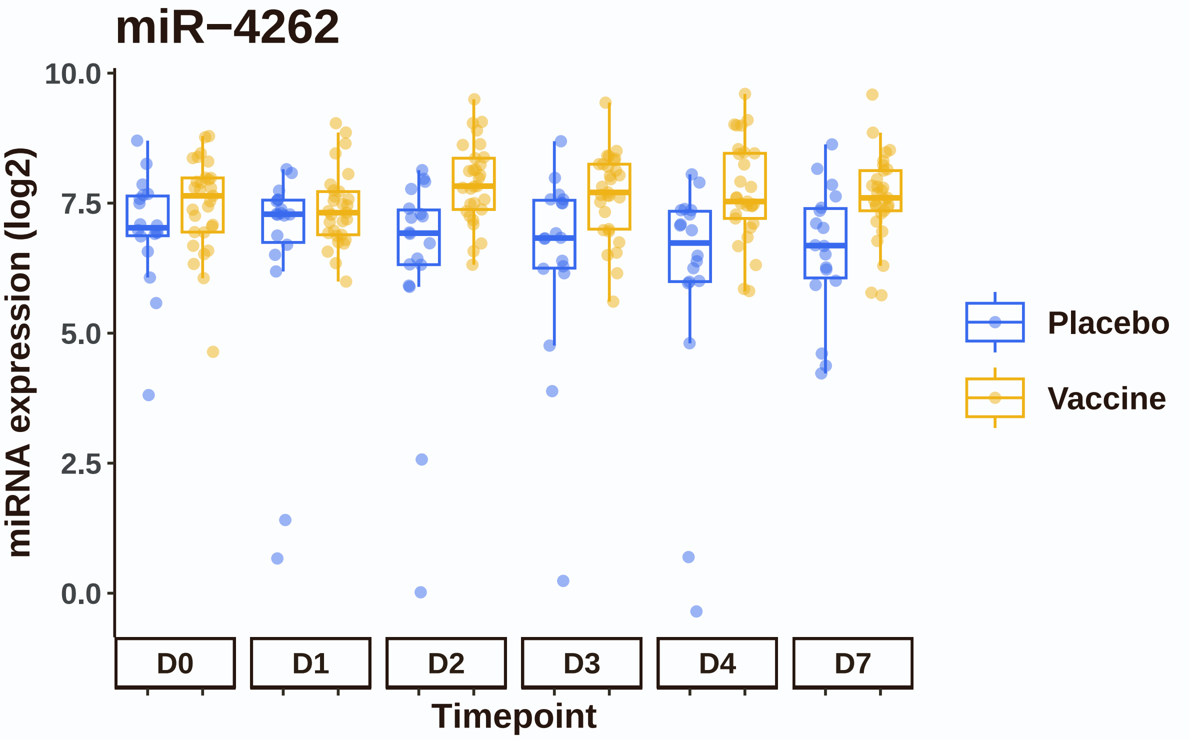

miR−4262
miRNA expression (log2)
10.0
7.5
5.0
2.5
0.0
D0
D1
D2
D3
D4
D7
Timepoint
Placebo
Vaccine

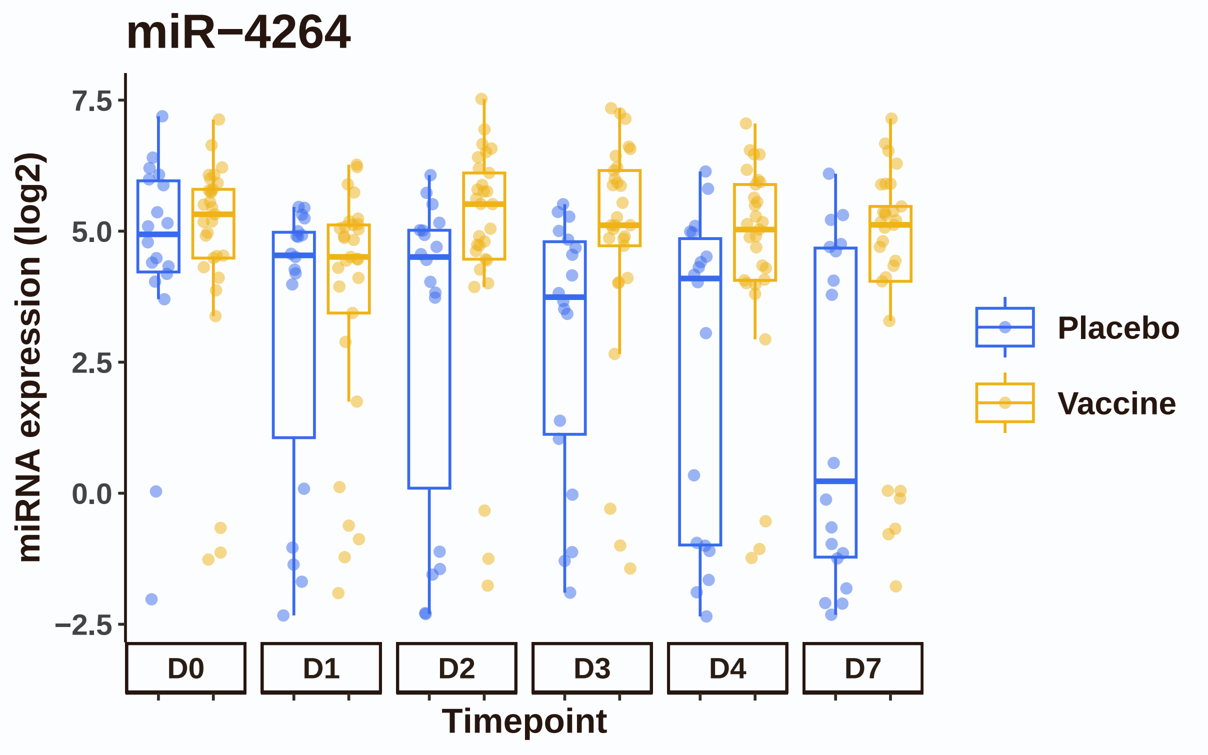

miR−4264
miRNA expression (log2)
7.5
5.0
2.5
0.0
−2.5
D0
D1
D2
D3
D4
D7
Timepoint
Placebo
Vaccine

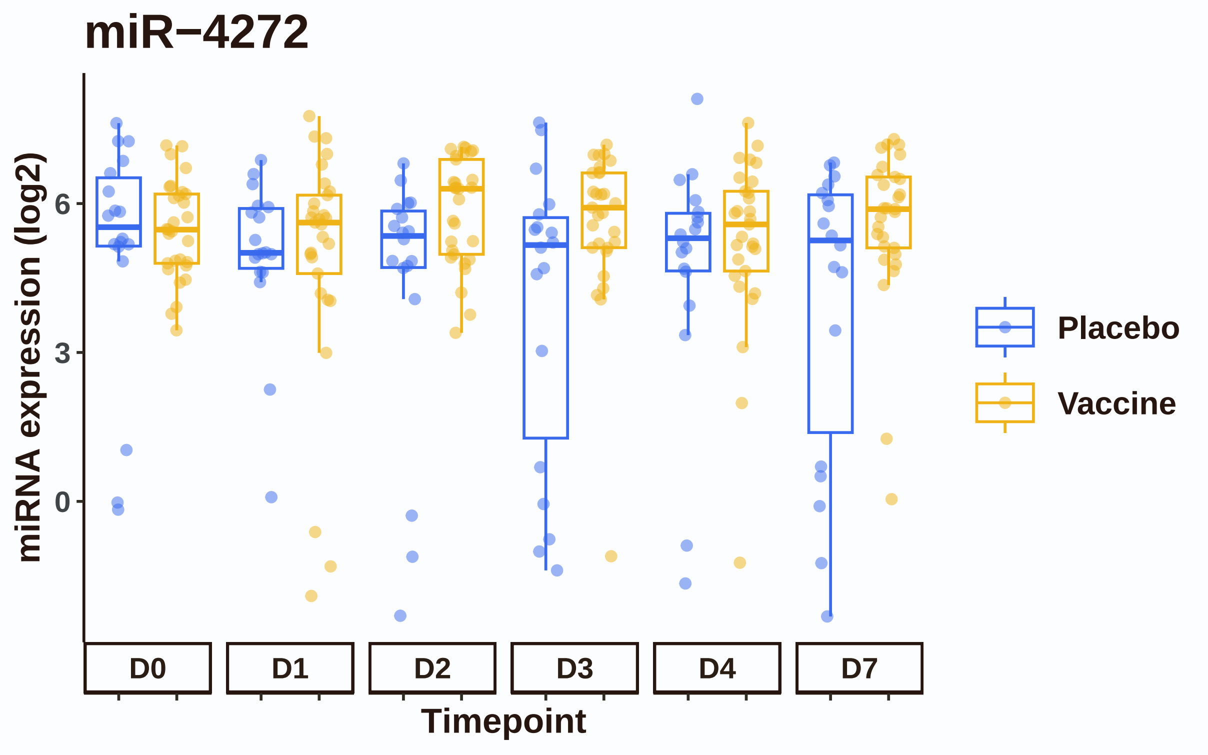
miR−4272
miRNA expression (log2)
0
3
6
D0
D1
D2
D3
D4
D7
Timepoint
Placebo
Vaccine

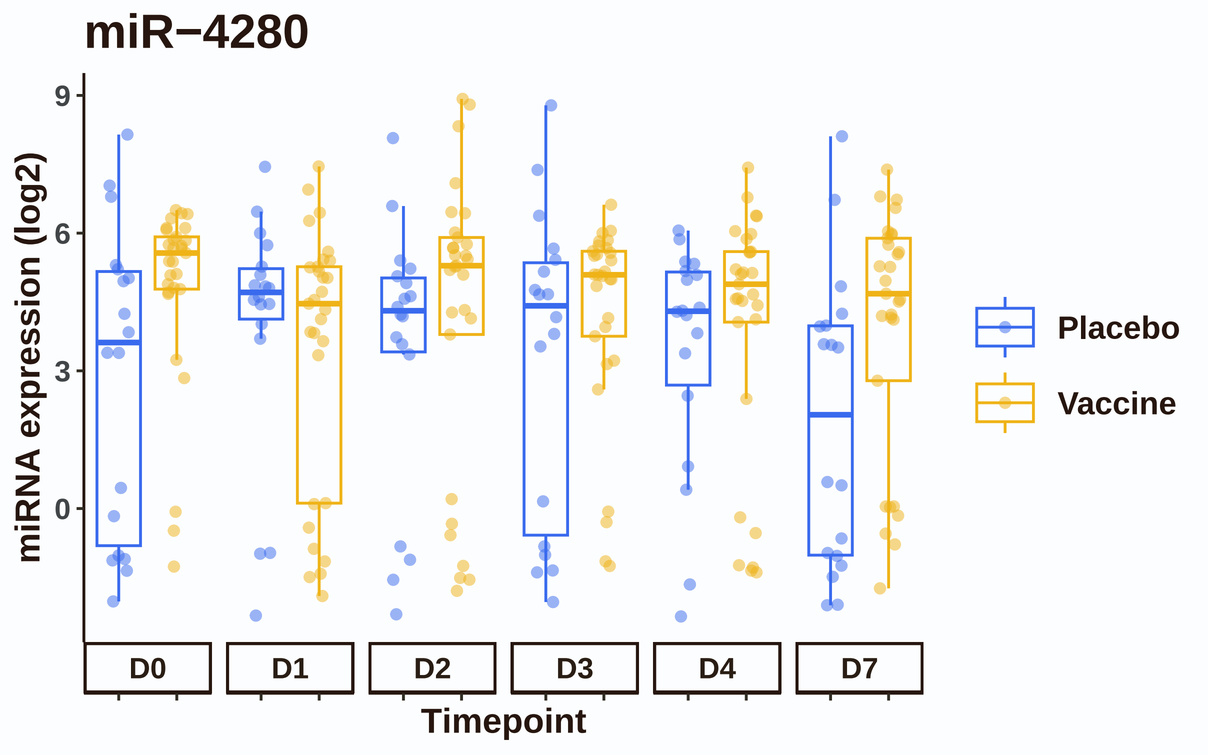

miR−4280
miRNA expression (log2)
9
6
3
0
D0
D1
D2
D3
D4
D7
Timepoint
Placebo
Vaccine

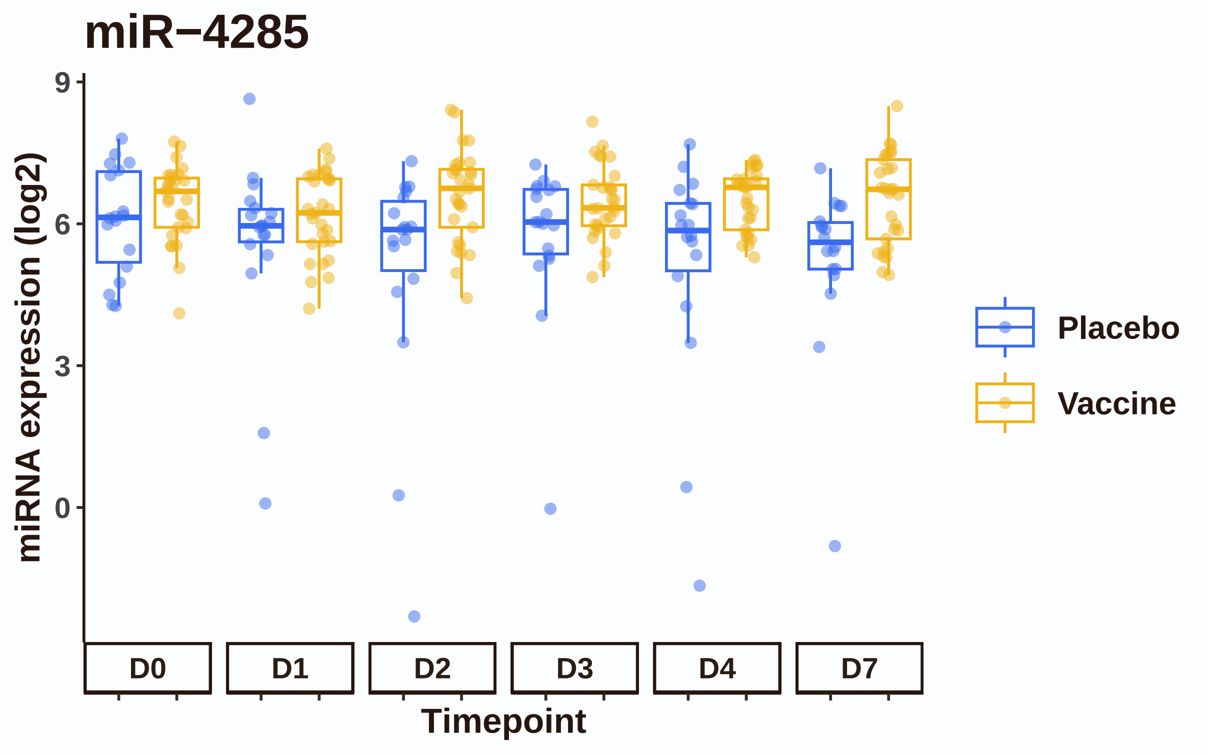
miR−4285
miRNA expression (log2)
9
6
3
0
D0
D1
D2
D3
D4
D7
Timepoint
Placebo
Vaccine

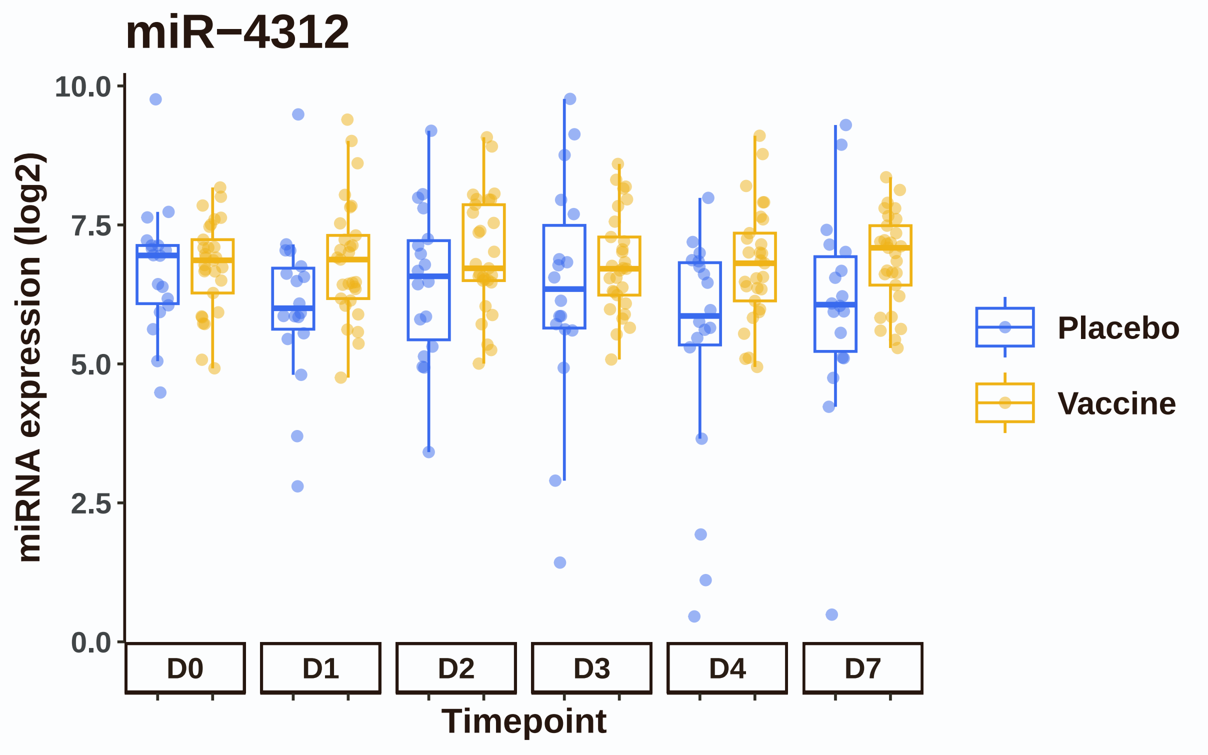
miR−4312
miRNA expression (log2)
10.0
7.5
5.0
2.5
0.0
D0
D1
D2
D3
D4
D7
Timepoint
Placebo
Vaccine

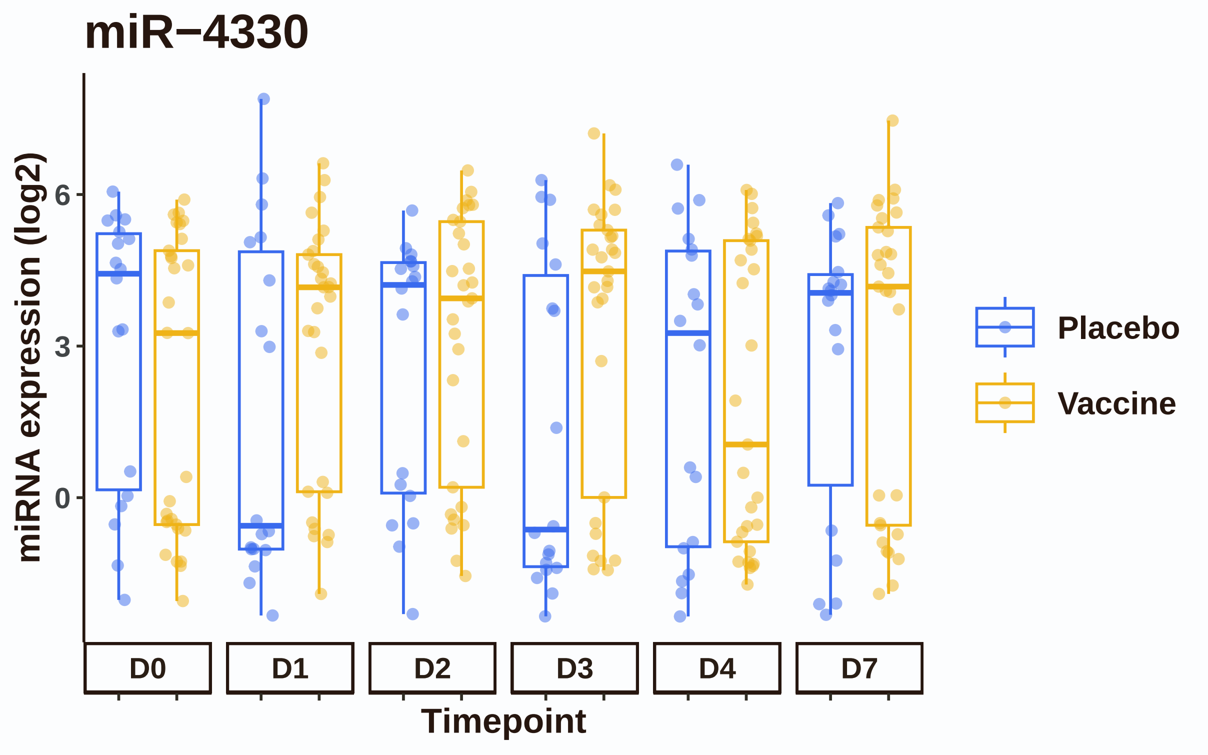
miR−4330
miRNA expression (log2)
6
3
0
D0
D1
D2
D3
D4
D7
Timepoint
Placebo
Vaccine

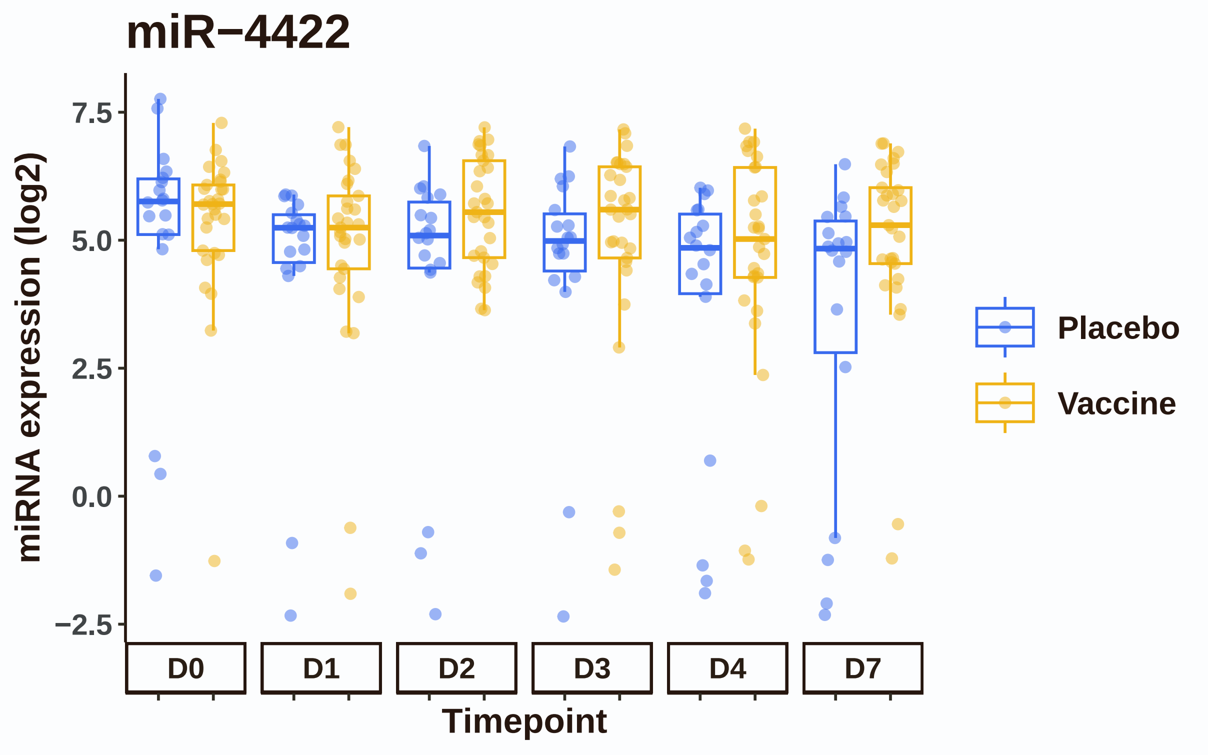
miR−4422
miRNA expression (log2)
7.5
5.0
2.5
0.0
−2.5
D0
D1
D2
D3
D4
D7
Timepoint
Placebo
Vaccine

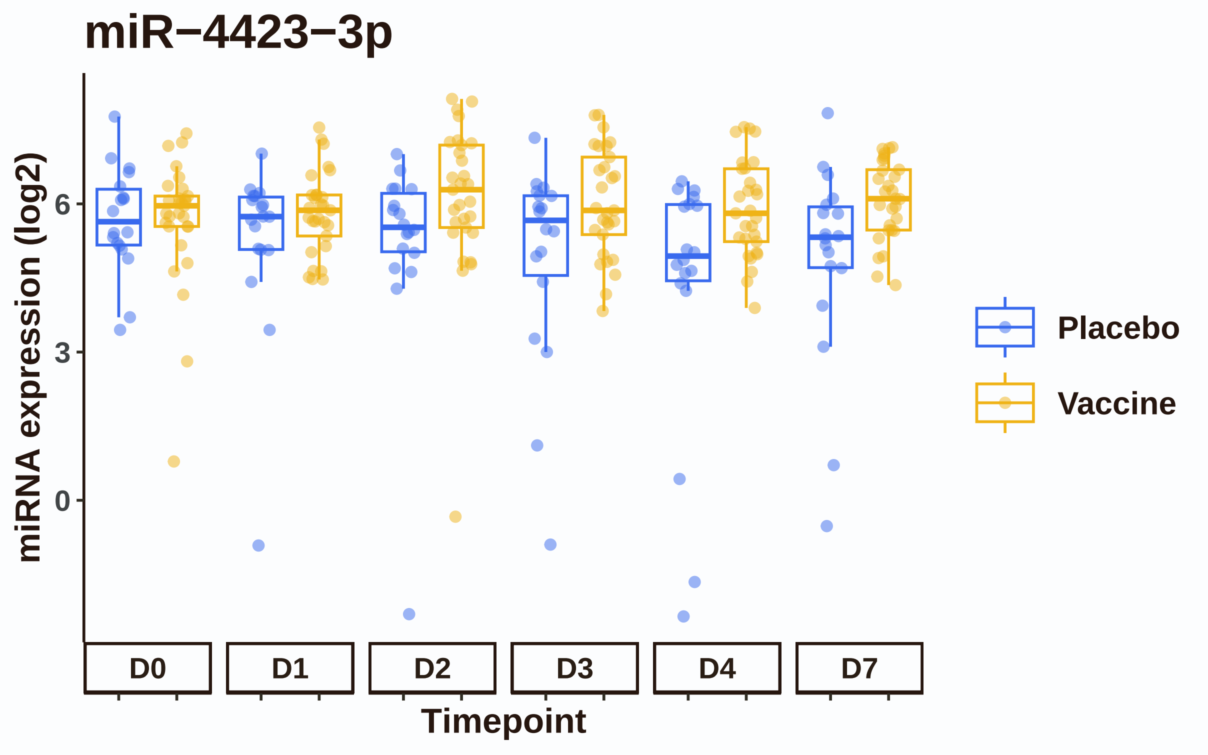

miR−4423−3p
miRNA expression (log2)
6
3
0
D0
D1
D2
D3
D4
D7
Timepoint
Placebo
Vaccine

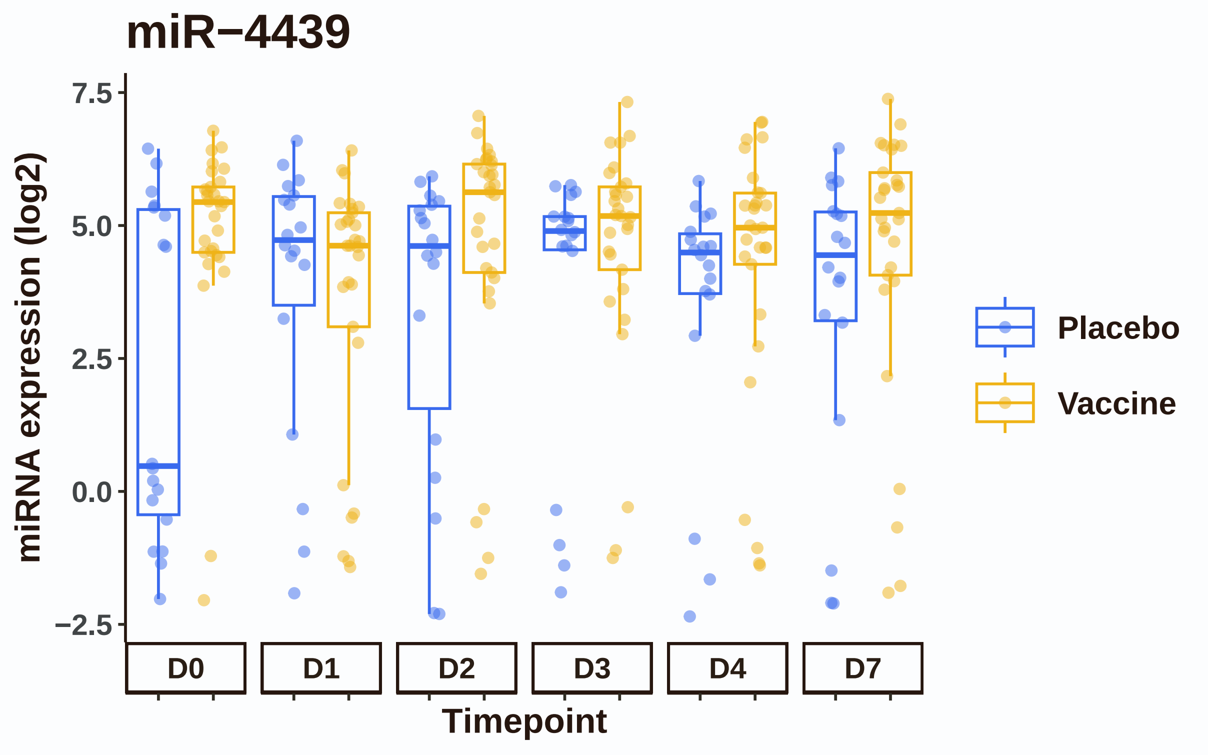

miR−4439
miRNA expression (log2)
7.5
5.0
2.5
0.0
−2.5
D0
D1
D2
D3
D4
D7
Timepoint
Placebo
Vaccine

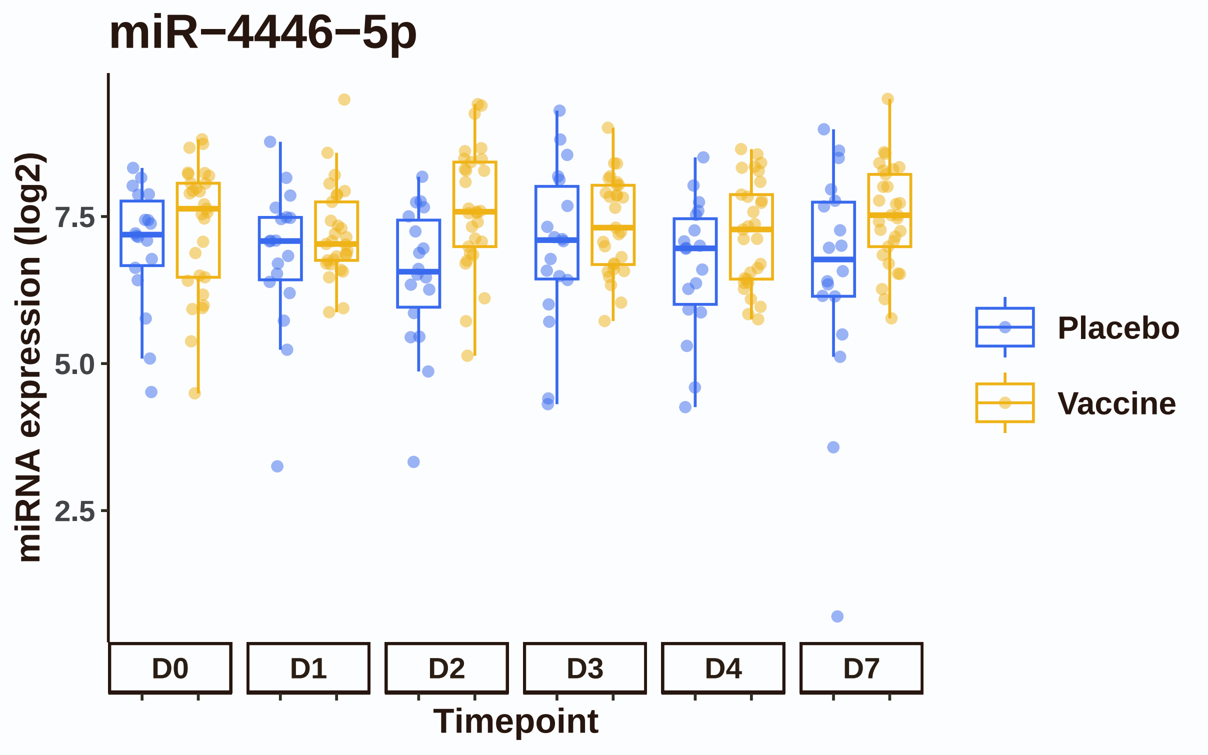
miR−4446−5p
miRNA expression (log2)
7.5
5.0
2.5
D0
D1
D2
D3
D4
D7
Timepoint
Placebo
Vaccine

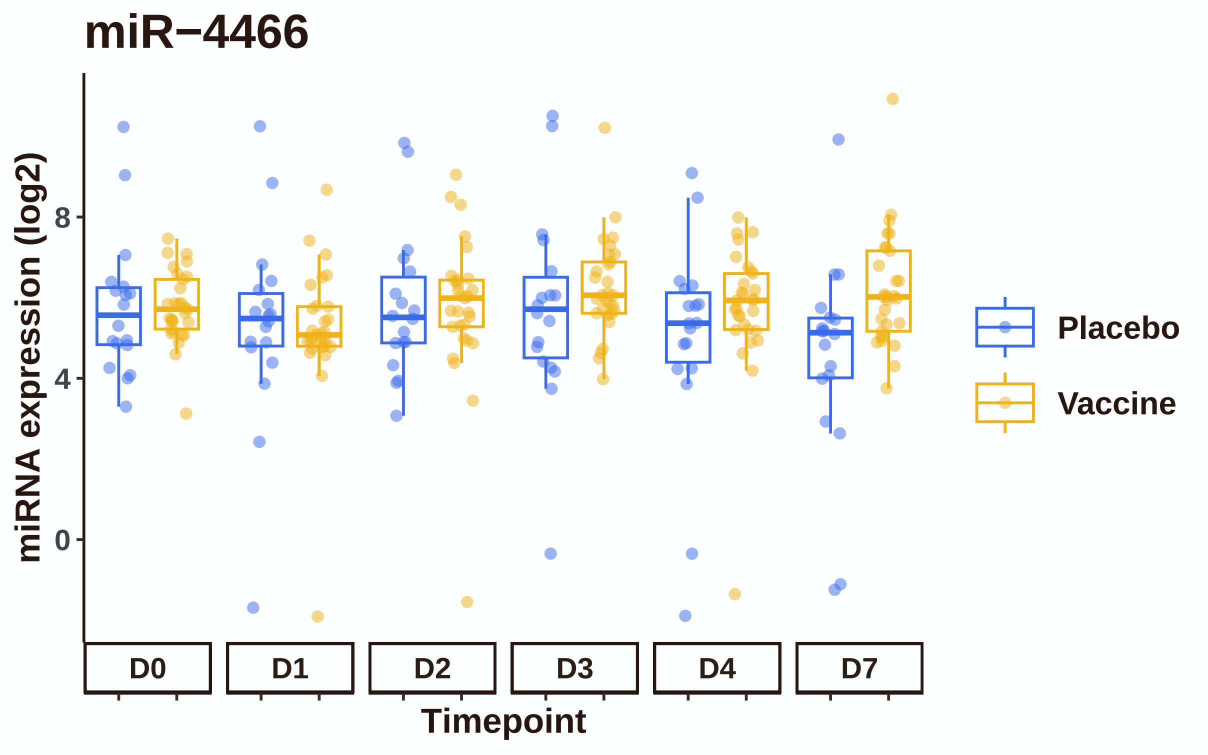

miR−4466
miRNA expression (log2)
8
4
0
D0
D1
D2
D3
D4
D7
Timepoint
Placebo
Vaccine

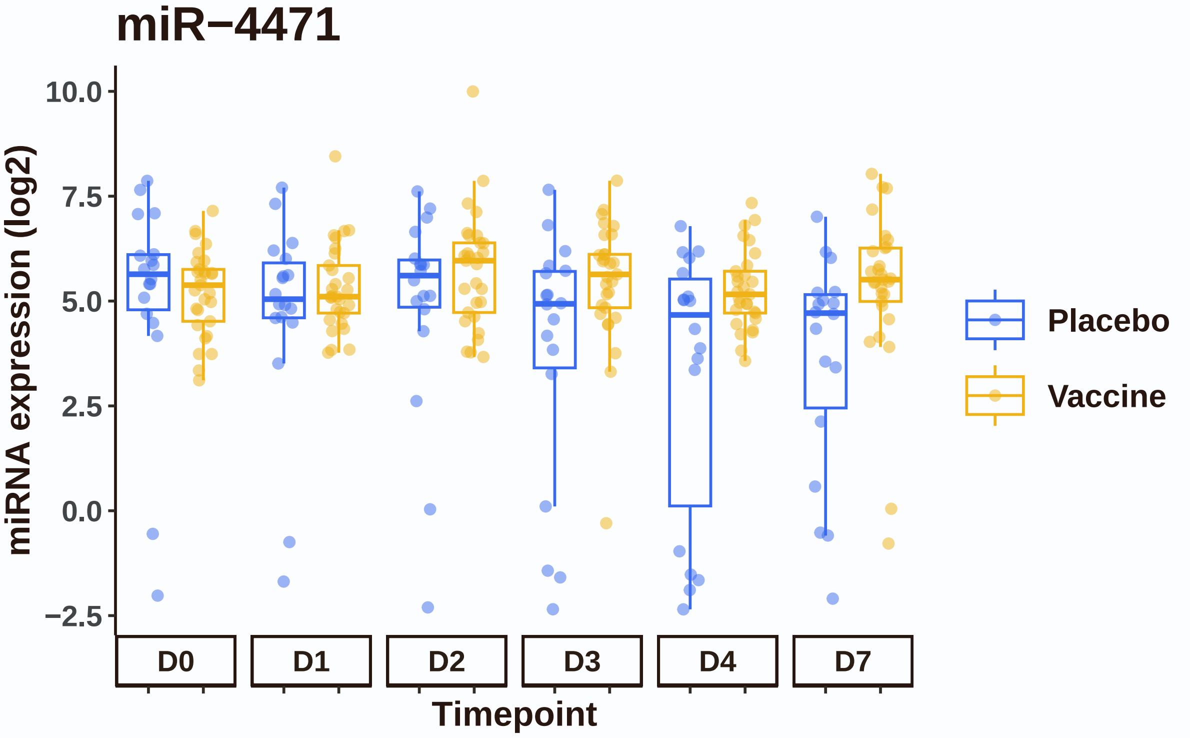

miR−4471
miRNA expression (log2)
10.0
7.5
5.0
2.5
0.0
−2.5
D0
D1
D2
D3
D4
D7
Timepoint
Placebo
Vaccine

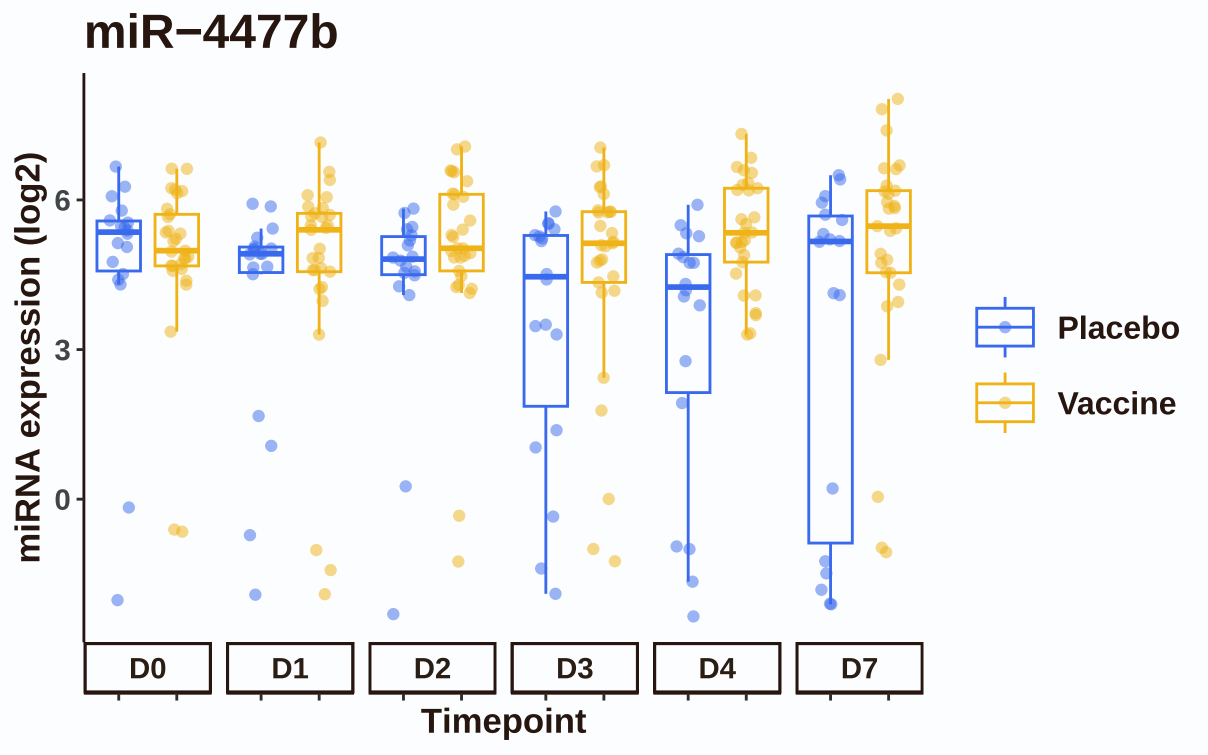

miR−4477b
miRNA expression (log2)
6
3
0
D0
D1
D2
D3
D4
D7
Timepoint
Placebo
Vaccine

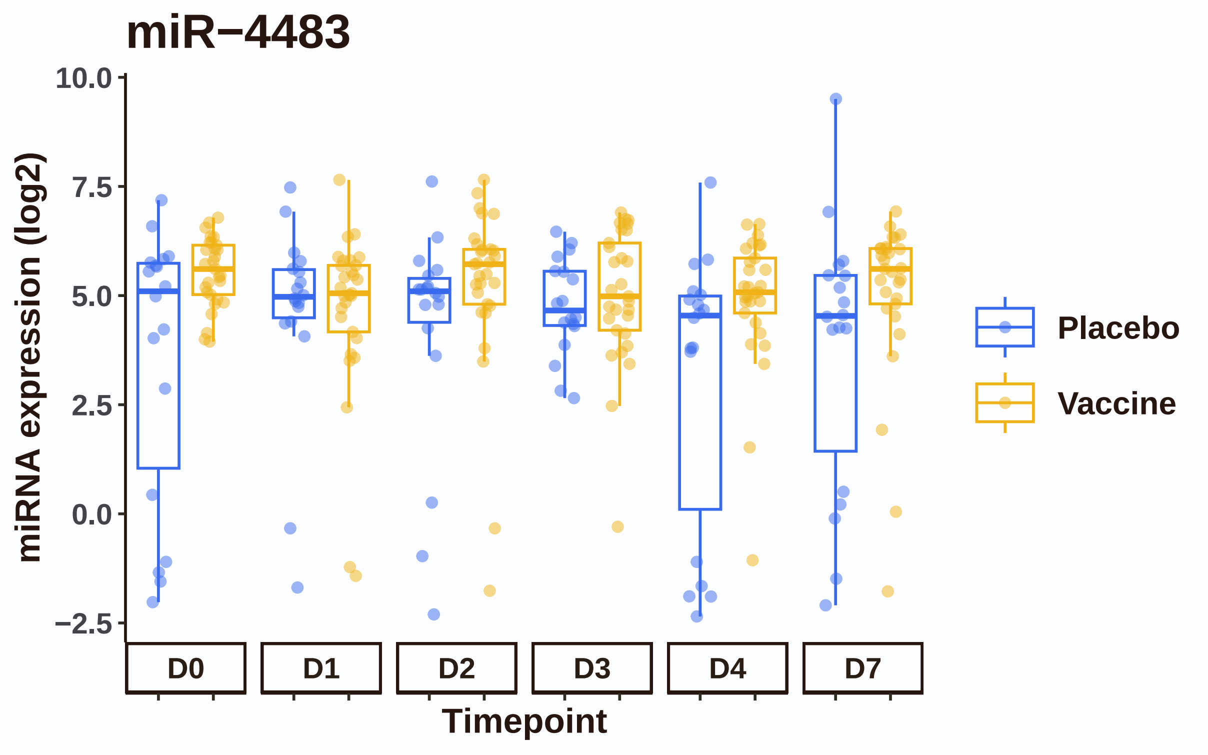
miR−4483
miRNA expression (log2)
10.0
7.5
5.0
2.5
0.0
−2.5
D0
D1
D2
D3
D4
D7
Timepoint
Placebo
Vaccine

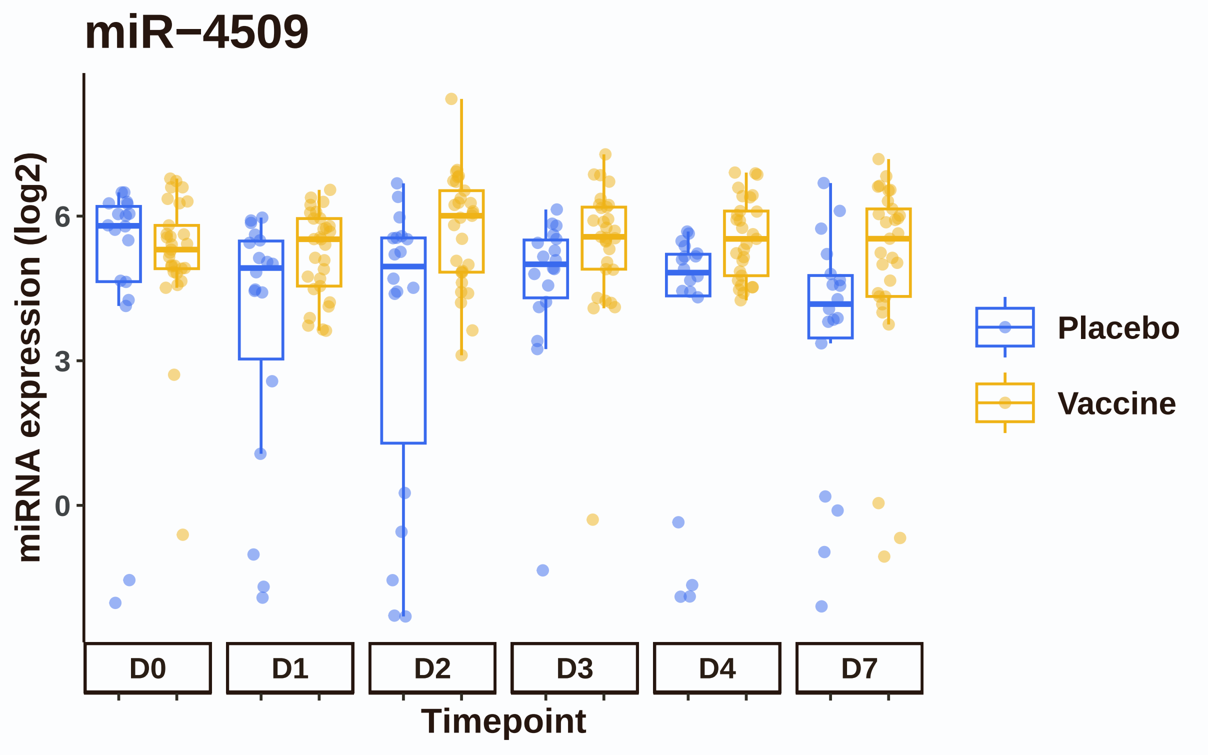
miR−4509
miRNA expression (log2)
6
3
0
D0
D1
D2
D3
D4
D7
Timepoint
Placebo
Vaccine

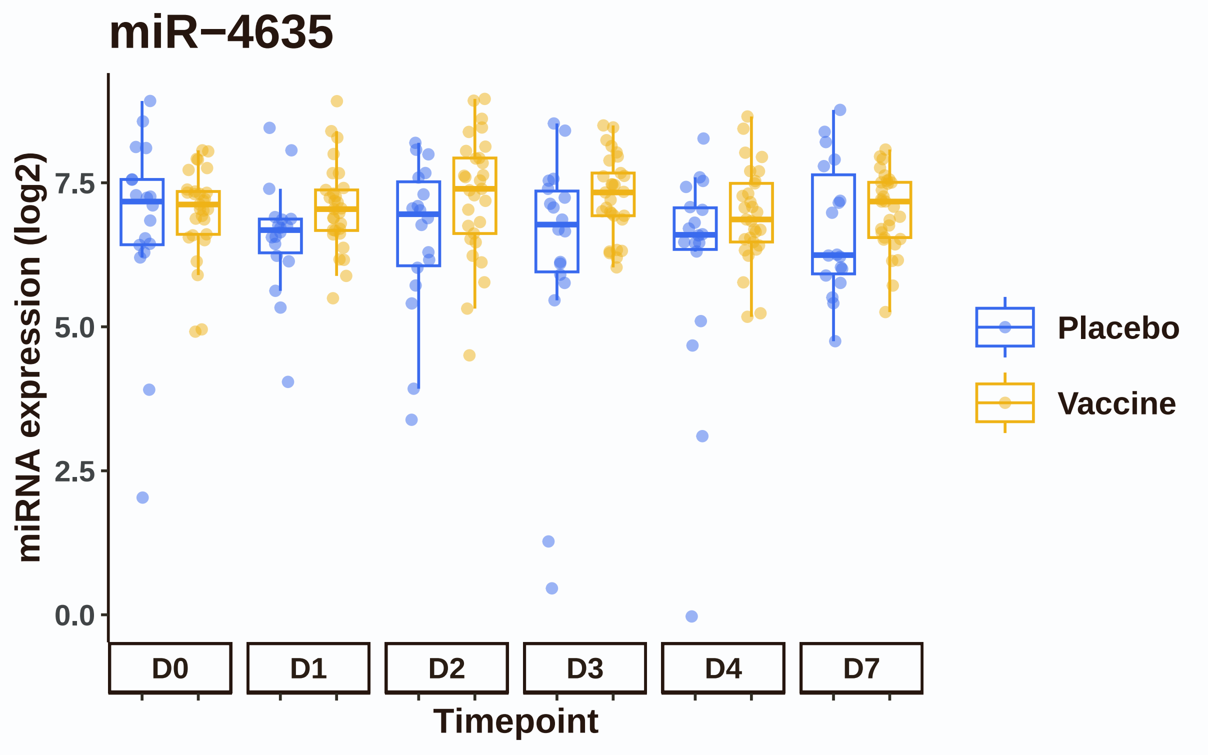
miR−4635
miRNA expression (log2)
7.5
5.0
2.5
0.0
D0
D1
D2
D3
D4
D7
Timepoint
Placebo
Vaccine

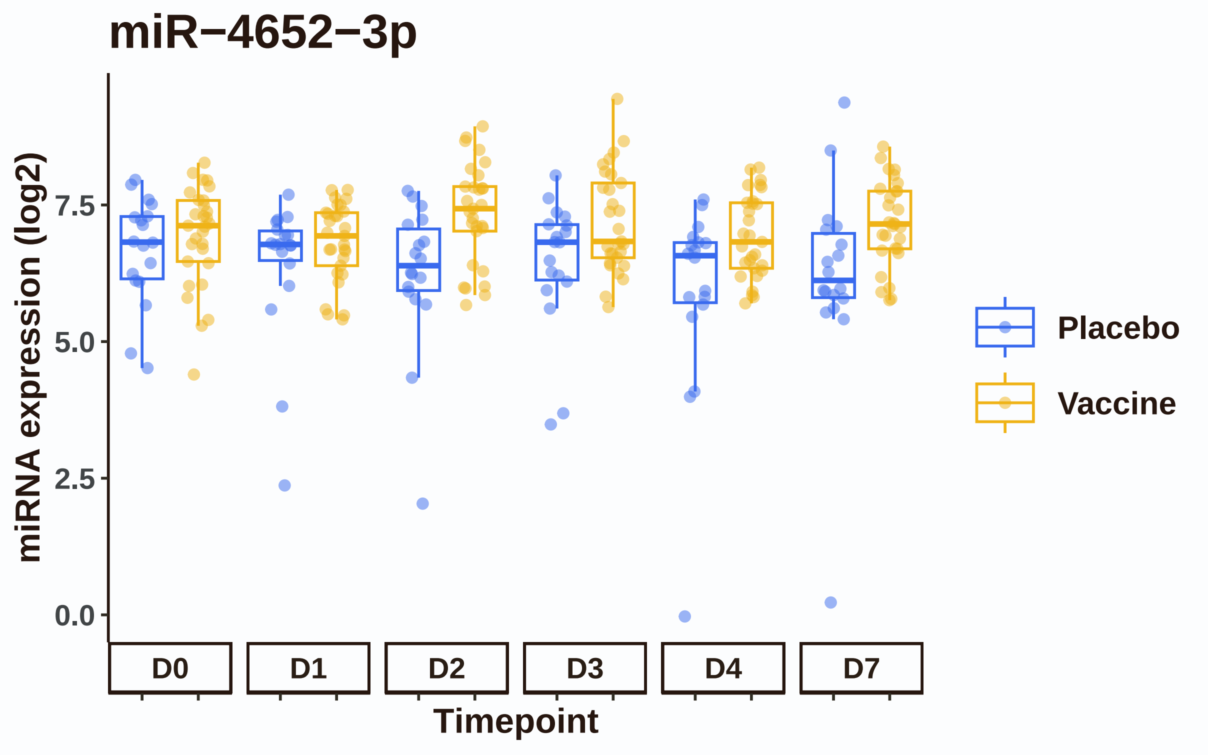

miR−4652−3p
miRNA expression (log2)
7.5
5.0
2.5
0.0
D0
D1
D2
D3
D4
D7
Timepoint
Placebo
Vaccine

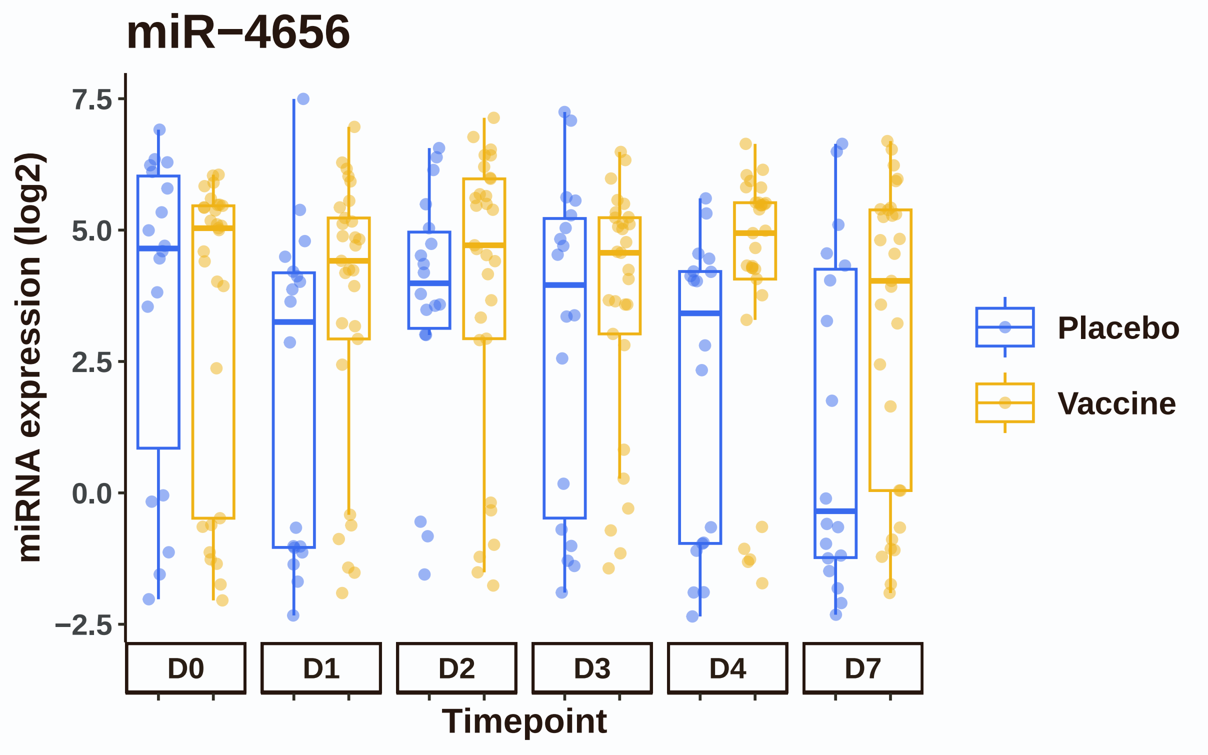

miR−4656
miRNA expression (log2)
7.5
5.0
2.5
0.0
−2.5
D0
D1
D2
D3
D4
D7
Timepoint
Placebo
Vaccine

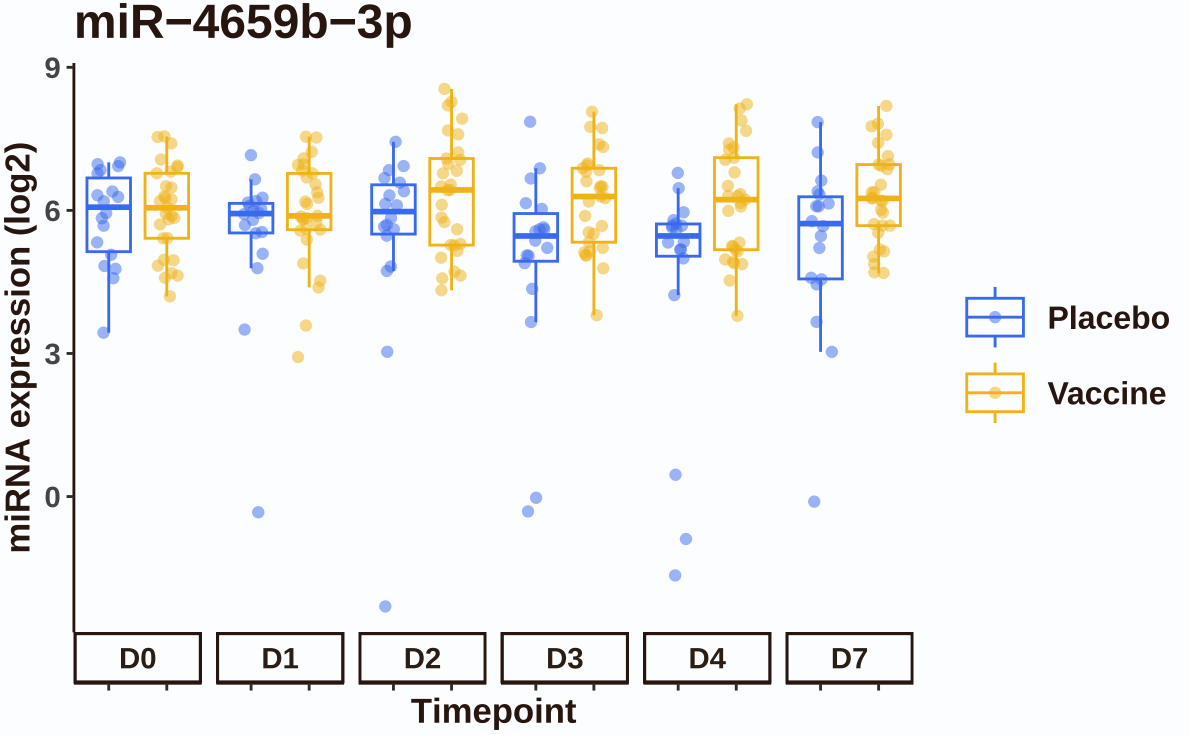
miR−4659b−3p
miRNA expression (log2)
9
6
3
0
D0
D1
D2
D3
D4
D7
Timepoint
Placebo
Vaccine

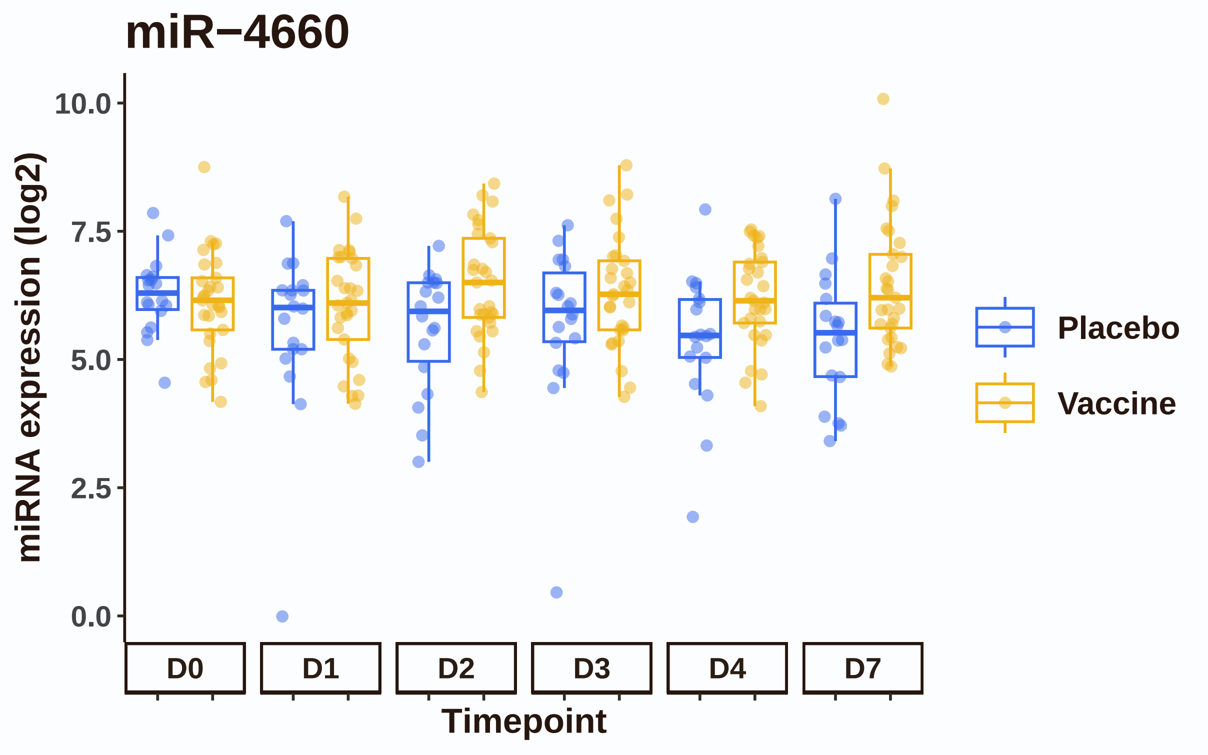

miR−4660
miRNA expression (log2)
10.0
7.5
5.0
2.5
0.0
D0
D1
D2
D3
D4
D7
Timepoint
Placebo
Vaccine

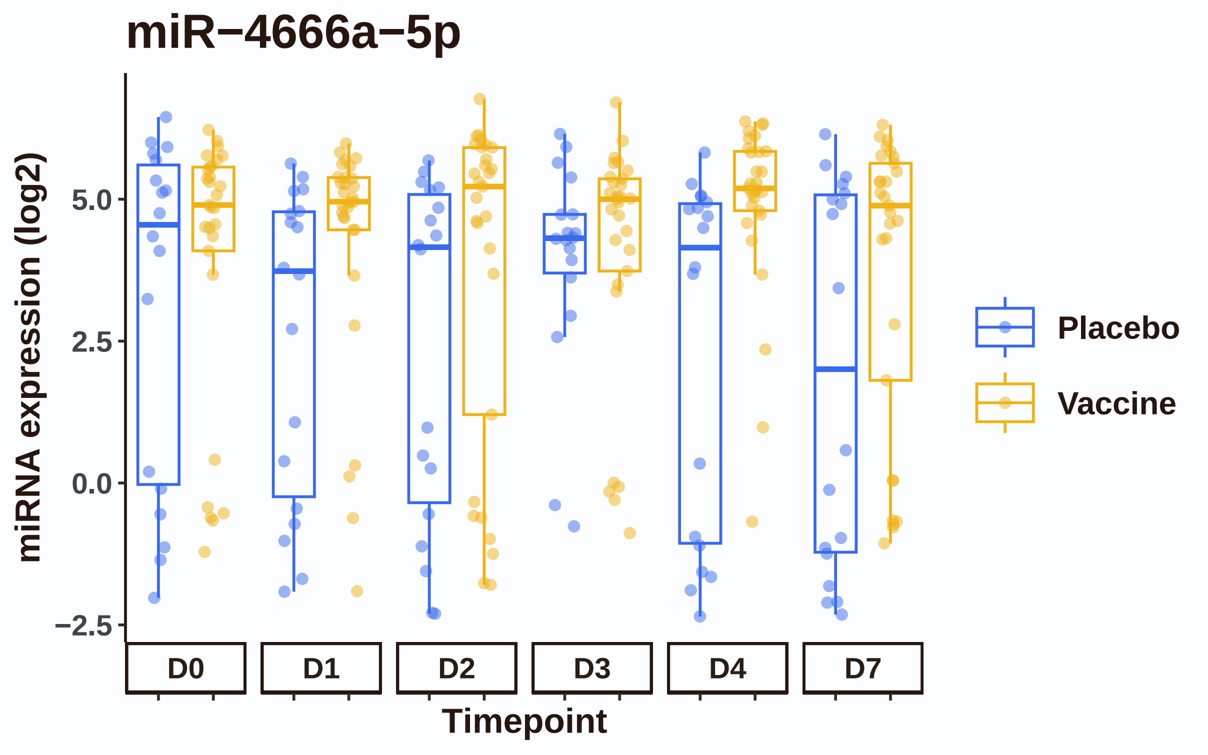

miR−4666a−5p
miRNA expression (log2)
5.0
2.5
0.0
−2.5
D0
D1
D2
D3
D4
D7
Timepoint
Placebo
Vaccine

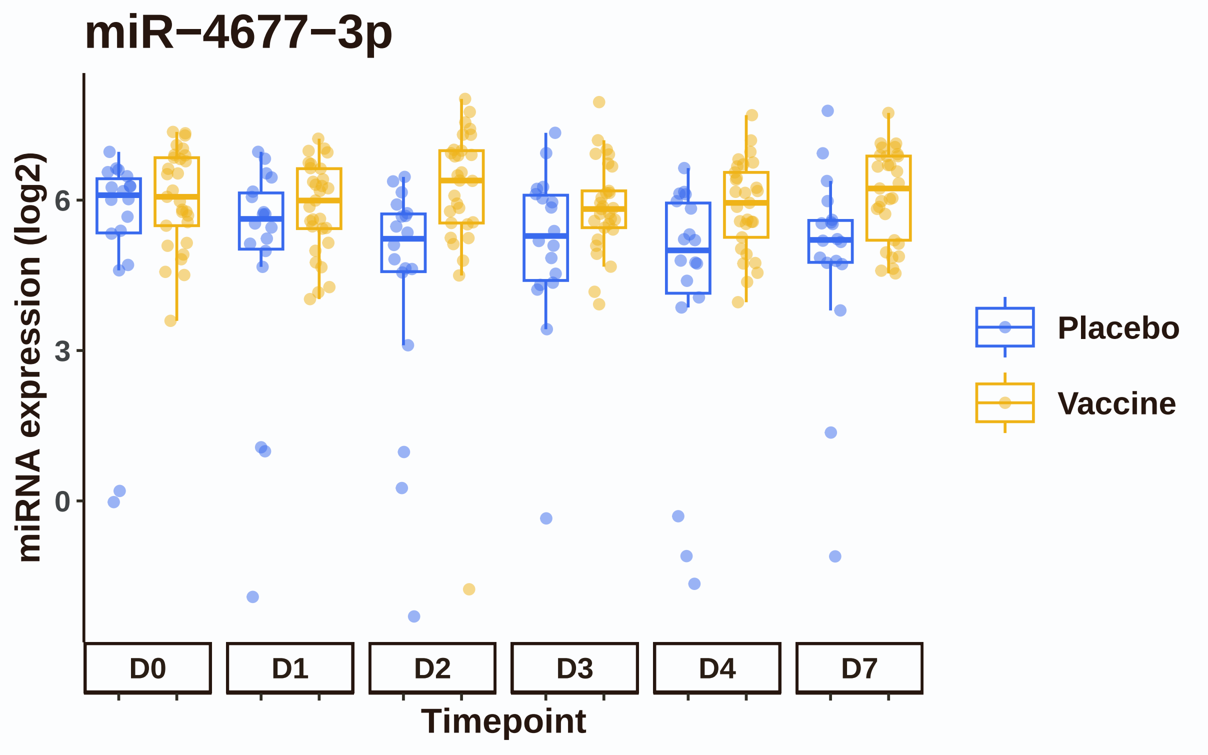
miR−4677−3p
miRNA expression (log2)
6
3
0
D0
D1
D2
D3
D4
D7
Timepoint
Placebo
Vaccine

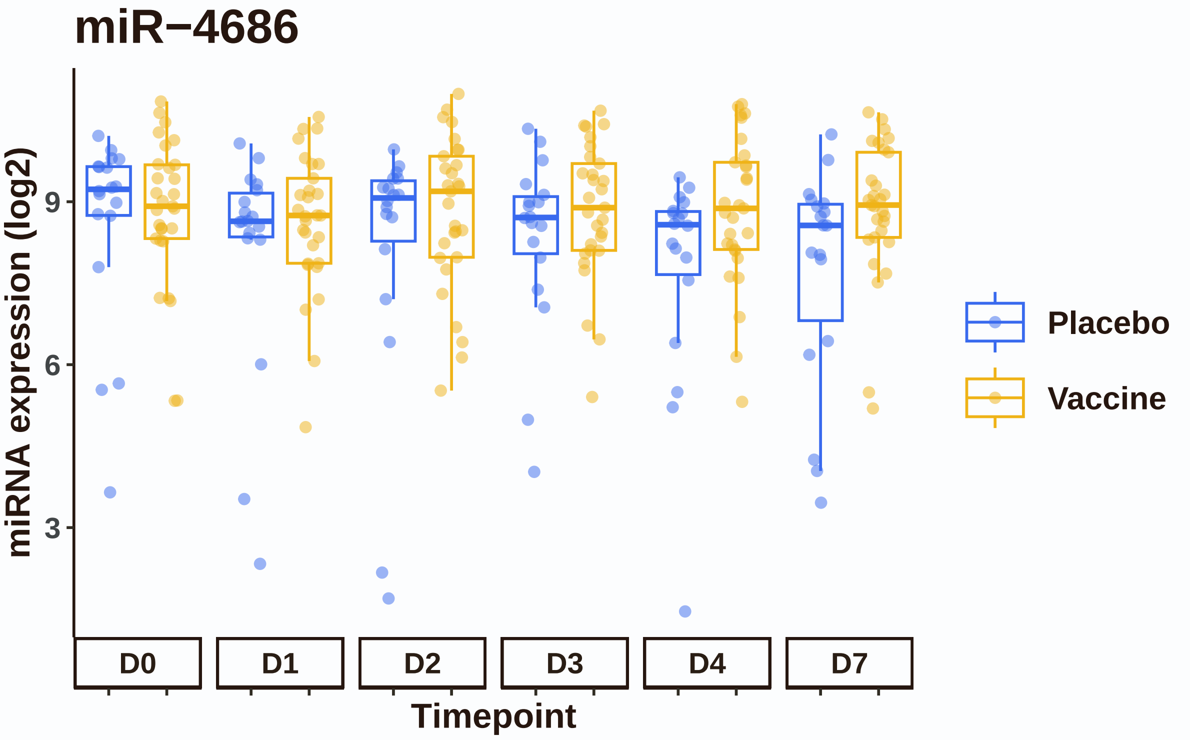
miR−4686
miRNA expression (log2)
9
6
3
D0
D1
D2
D3
D4
D7
Timepoint
Placebo
Vaccine

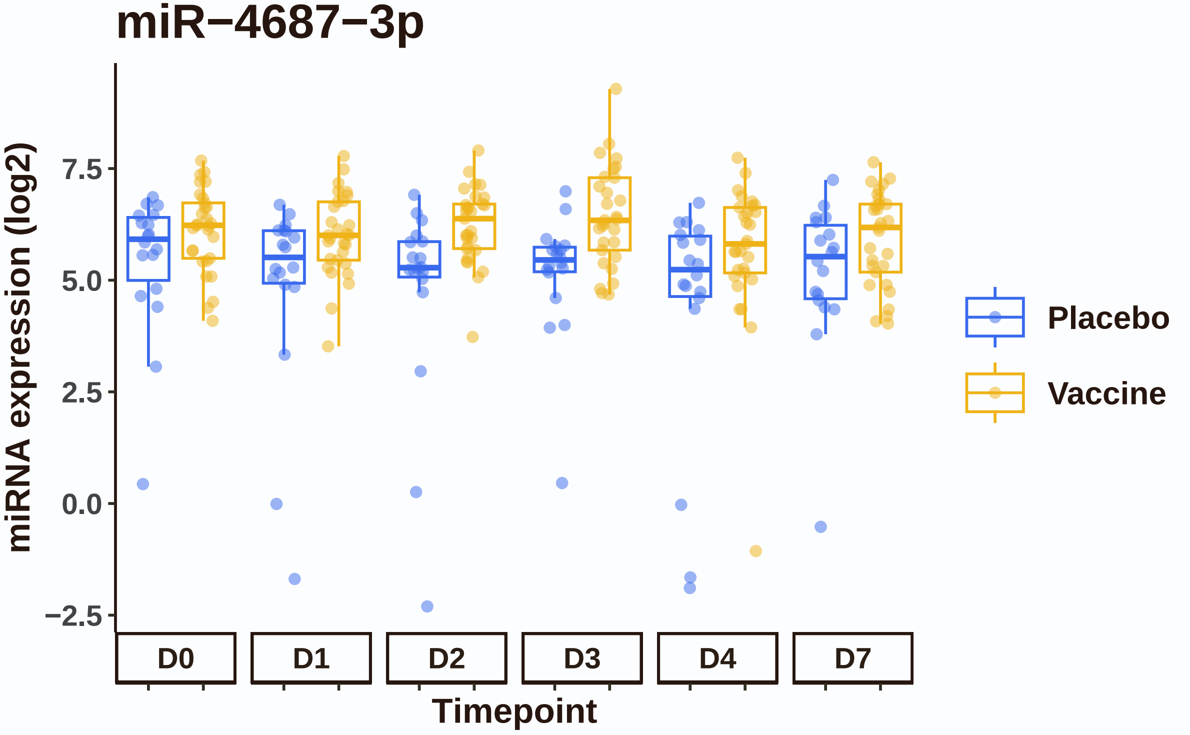
miR−4687−3p
miRNA expression (log2)
7.5
5.0
2.5
0.0
−2.5
D0
D1
D2
D3
D4
D7
Timepoint
Placebo
Vaccine

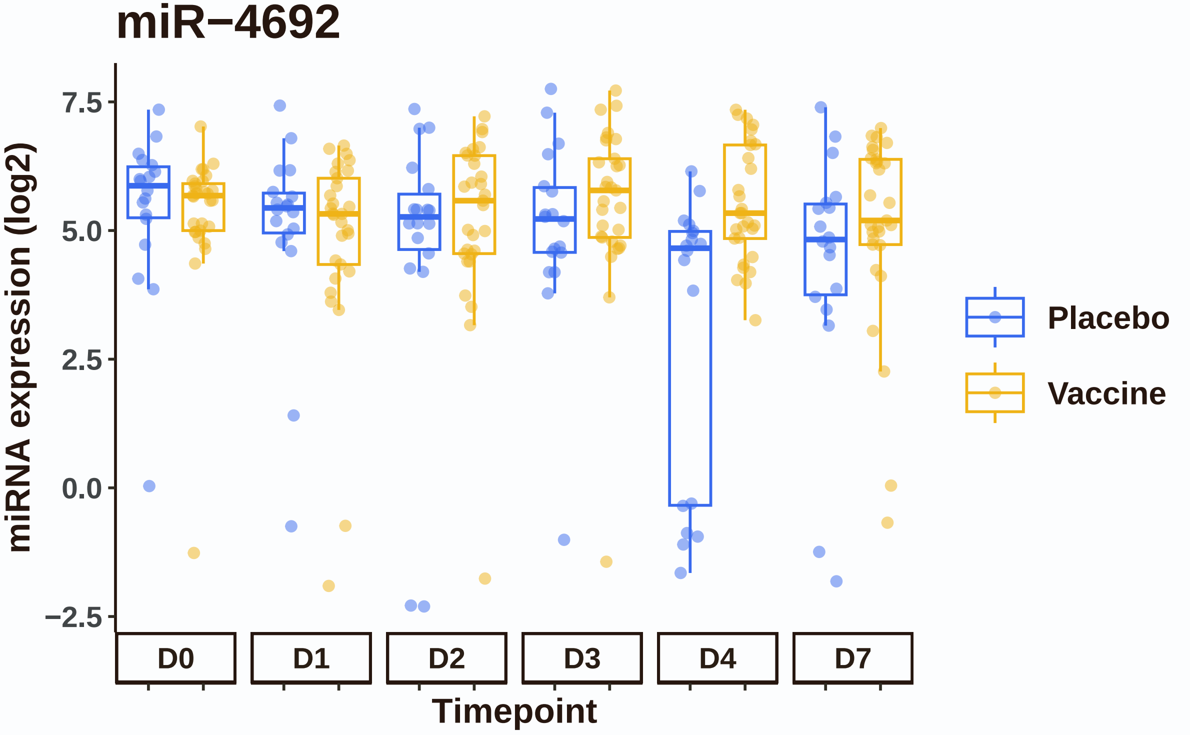
miR−4692
miRNA expression (log2)
7.5
5.0
2.5
0.0
−2.5
D0
D1
D2
D3
D4
D7
Timepoint
Placebo
Vaccine

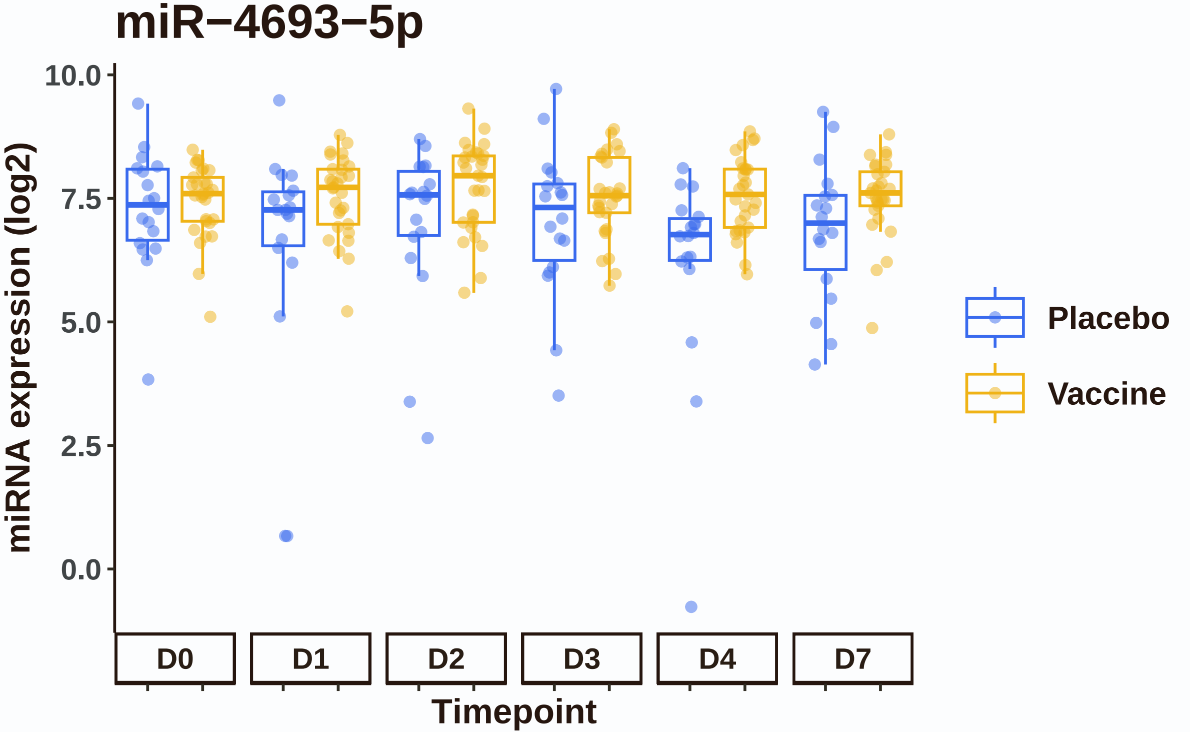
miR−4693−5p
miRNA expression (log2)
10.0
7.5
5.0
2.5
0.0
D0
D1
D2
D3
D4
D7
Timepoint
Placebo
Vaccine

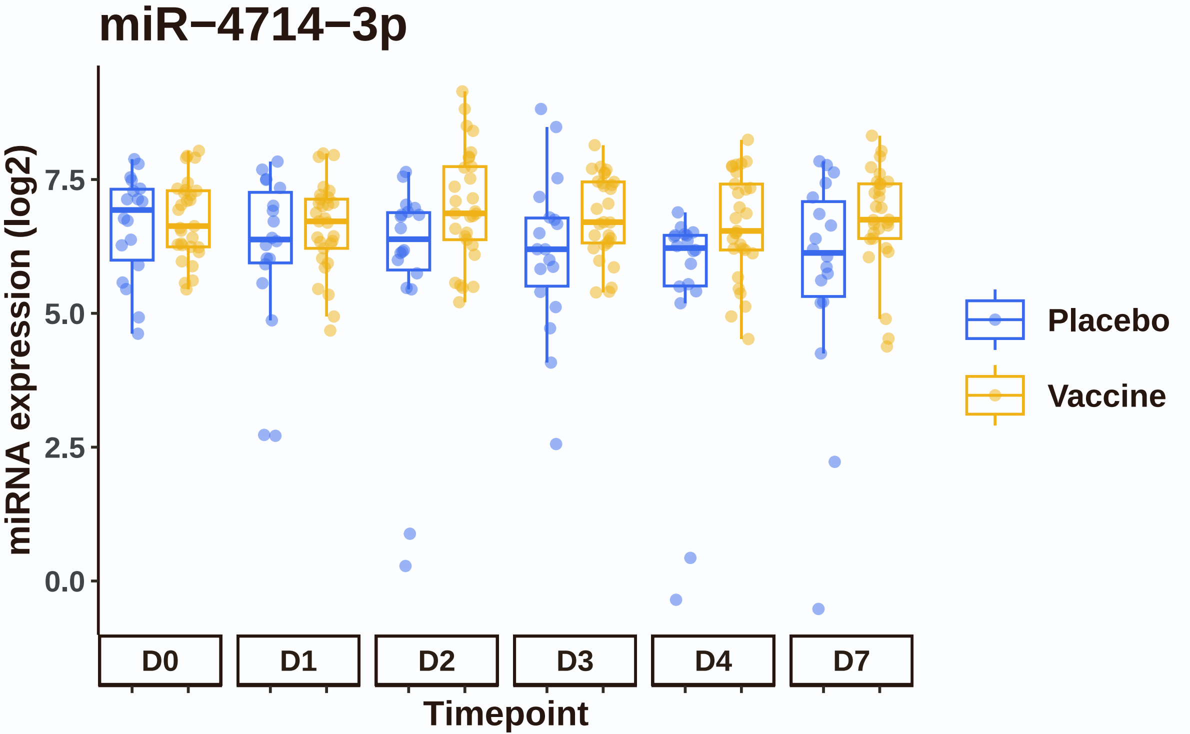
miR−4714−3p
miRNA expression (log2)
7.5
5.0
2.5
0.0
D0
D1
D2
D3
D4
D7
Timepoint
Placebo
Vaccine

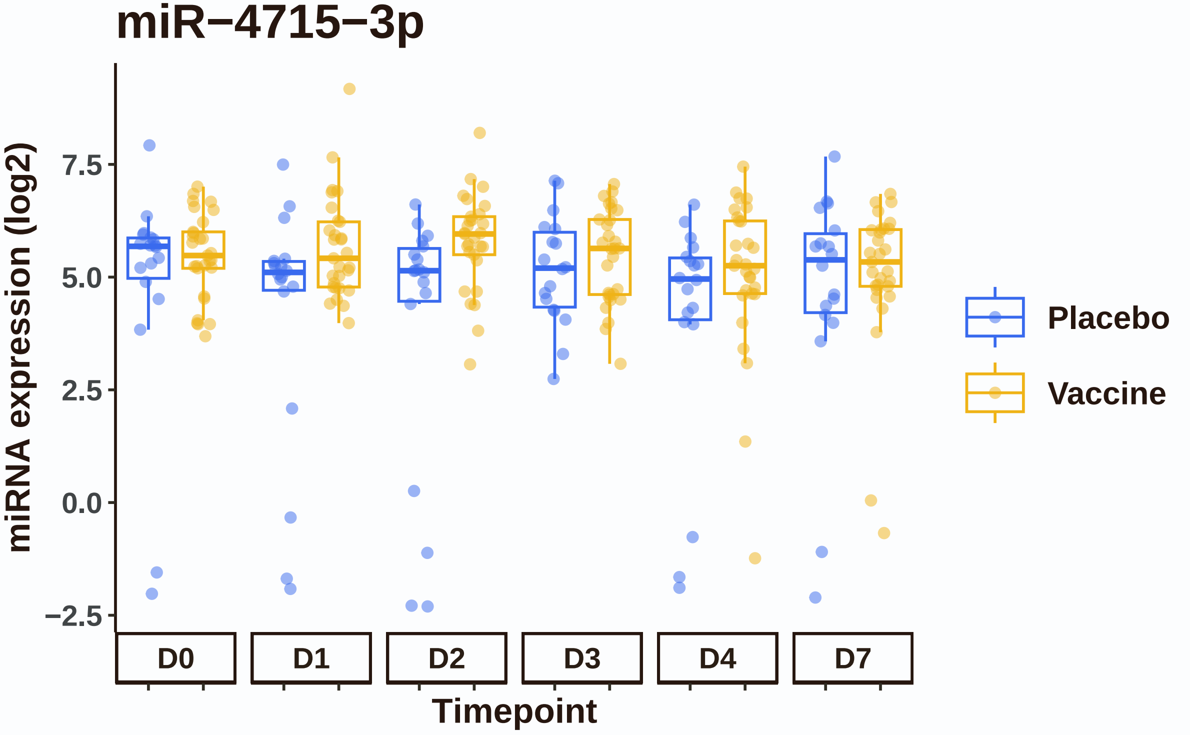

miR−4715−3p
miRNA expression (log2)
7.5
5.0
2.5
0.0
−2.5
D0
D1
D2
D3
D4
D7
Timepoint
Placebo
Vaccine

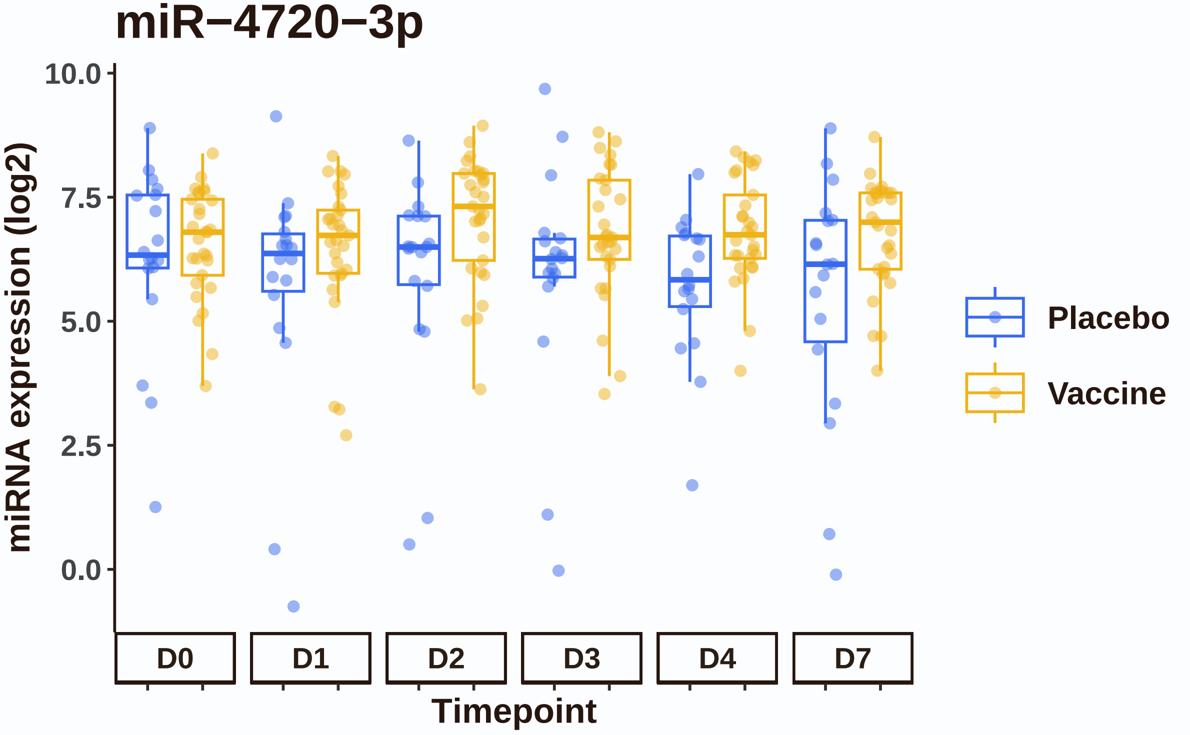
miR−4720−3p
miRNA expression (log2)
10.0
7.5
5.0
2.5
0.0
D0
D1
D2
D3
D4
D7
Timepoint
Placebo
Vaccine

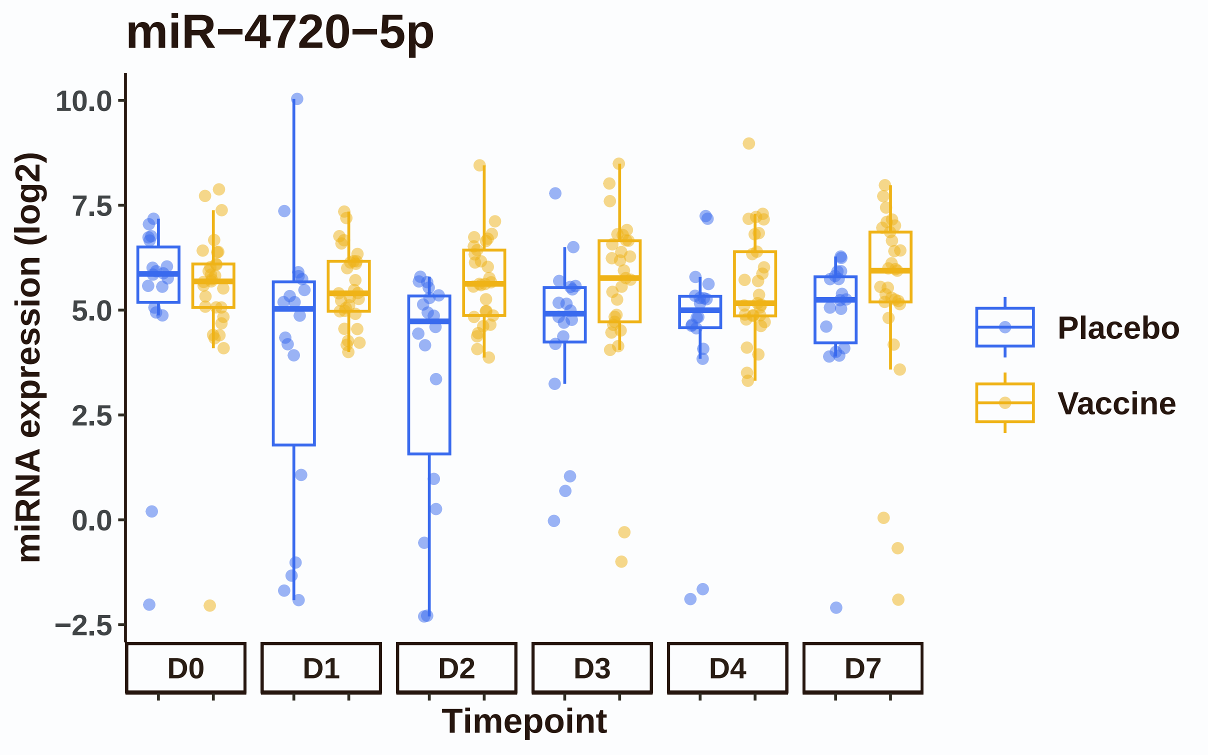
miR−4720−5p
miRNA expression (log2)
10.0
7.5
5.0
2.5
0.0
−2.5
D0
D1
D2
D3
D4
D7
Timepoint
Placebo
Vaccine

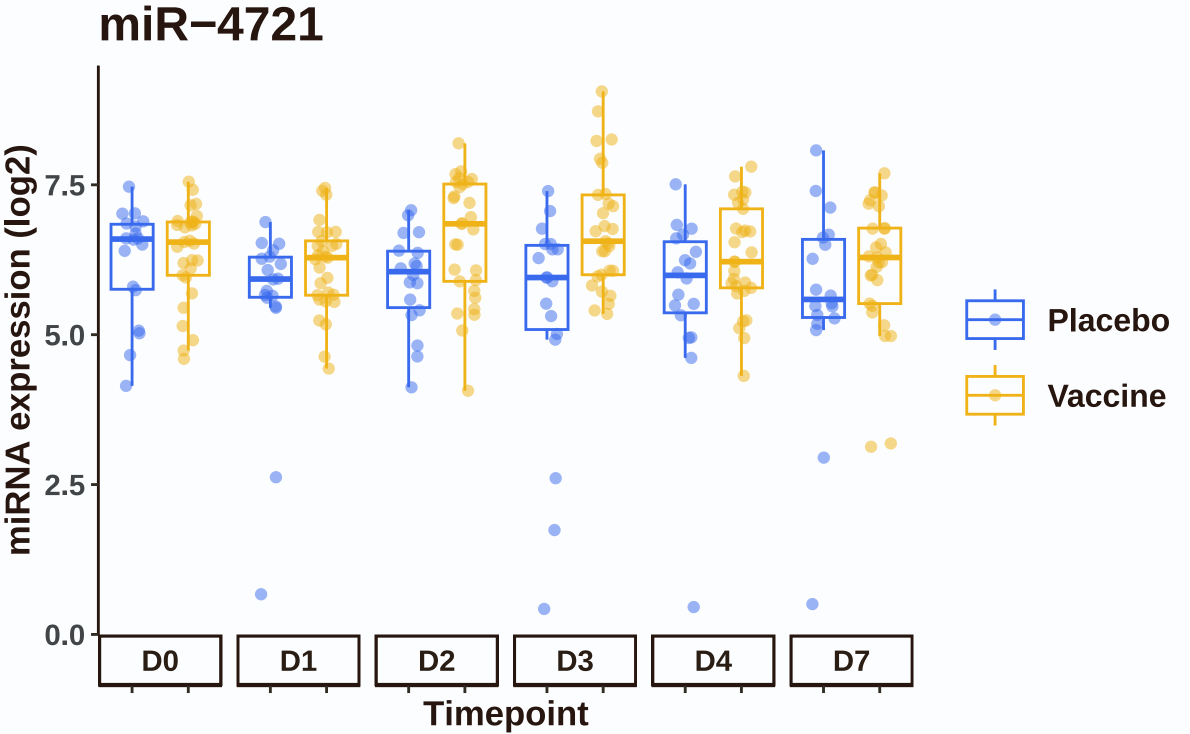
miR−4721
miRNA expression (log2)
7.5
5.0
2.5
0.0
D0
D1
D2
D3
D4
D7
Timepoint
Placebo
Vaccine

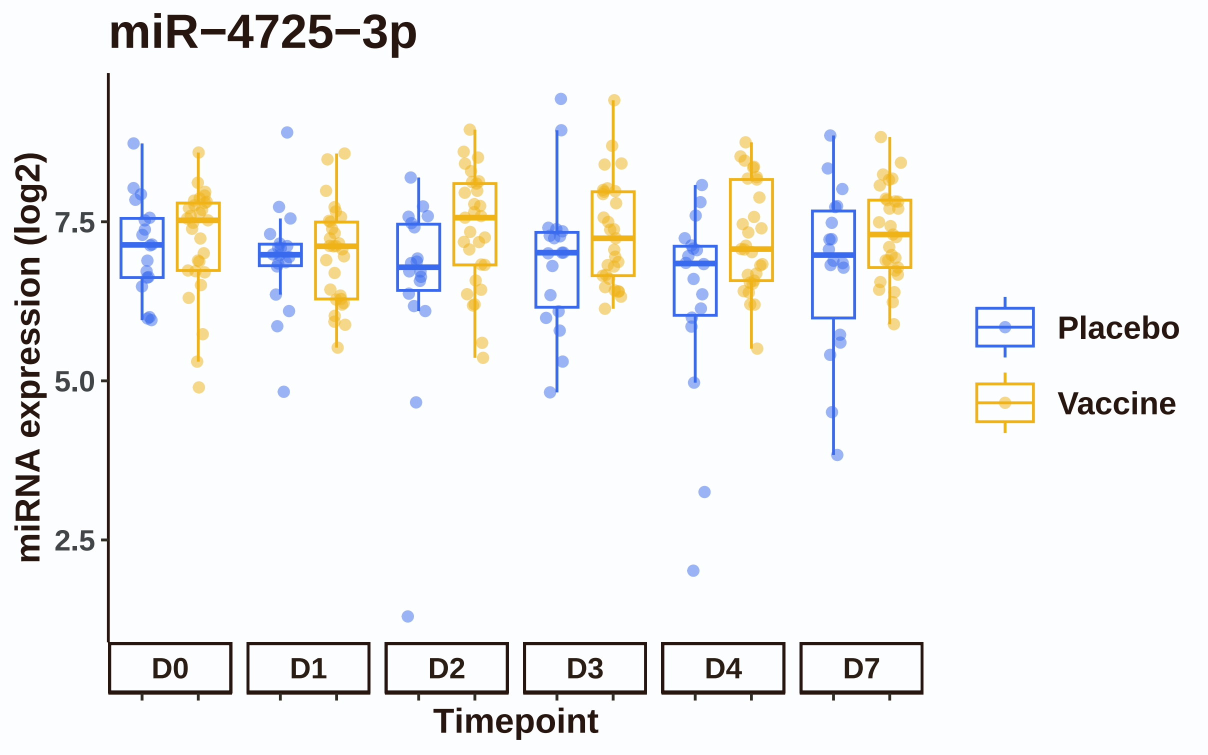

miR−4725−3p
miRNA expression (log2)
7.5
5.0
2.5
D0
D1
D2
D3
D4
D7
Timepoint
Placebo
Vaccine

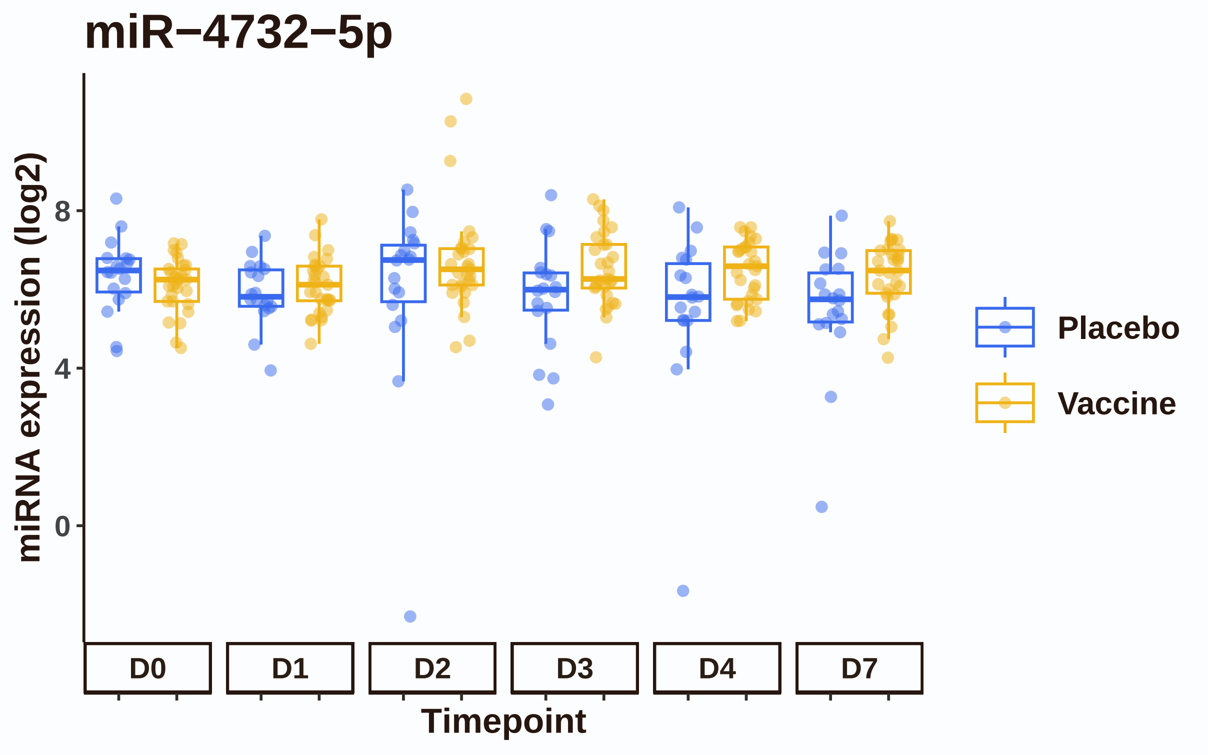

miR−4732−5p
miRNA expression (log2)
8
4
0
D0
D1
D2
D3
D4
D7
Timepoint
Placebo
Vaccine

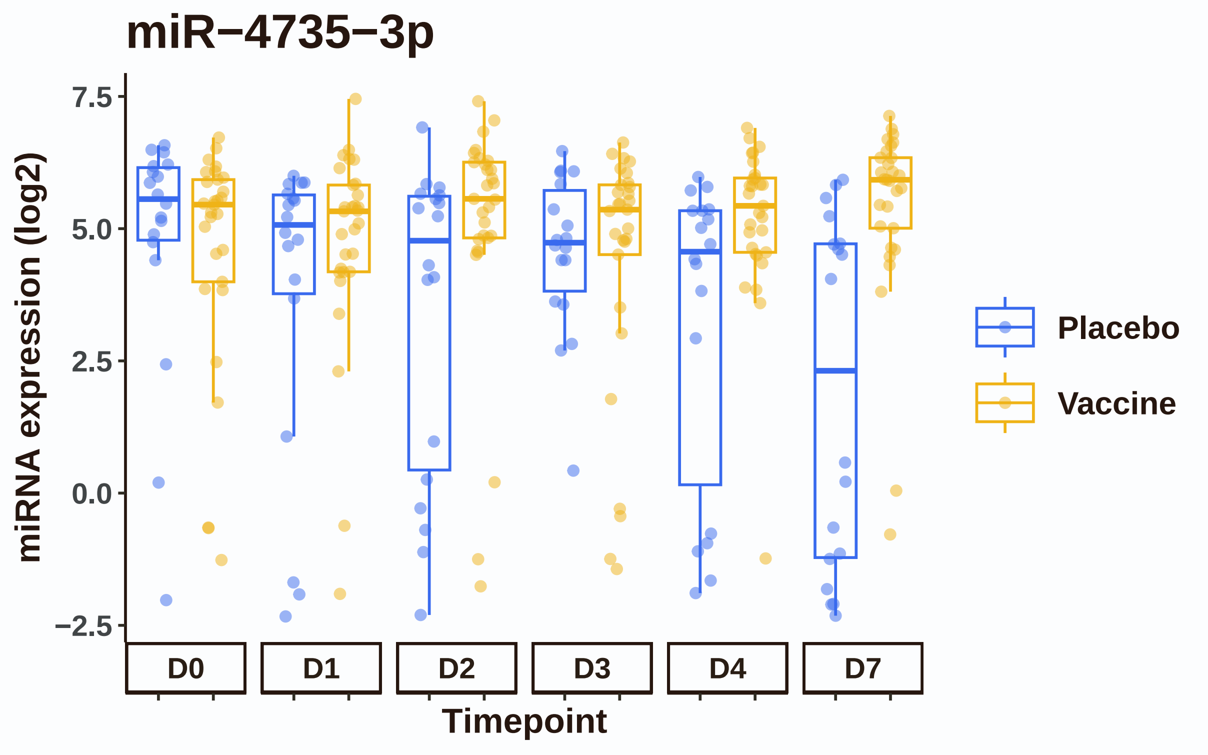
miR−4735−3p
miRNA expression (log2)
7.5
5.0
2.5
0.0
−2.5
D0
D1
D2
D3
D4
D7
Timepoint
Placebo
Vaccine

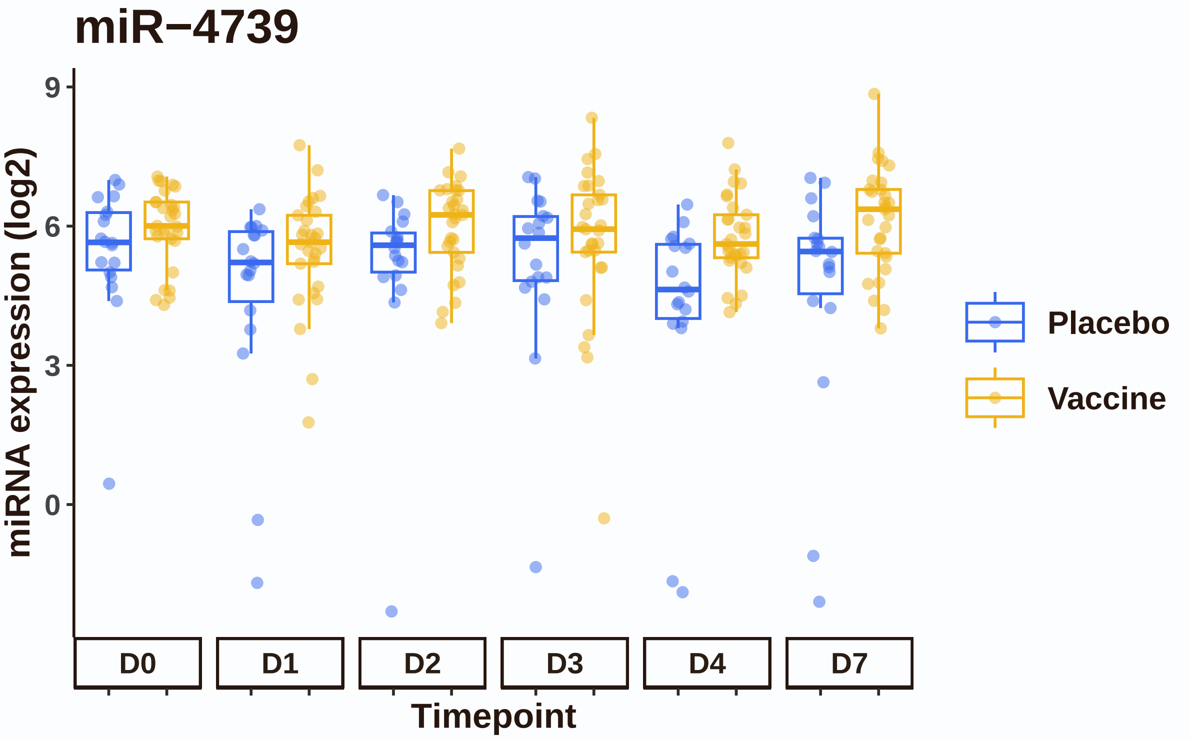
miR−4739
miRNA expression (log2)
9
6
3
0
D0
D1
D2
D3
D4
D7
Timepoint
Placebo
Vaccine

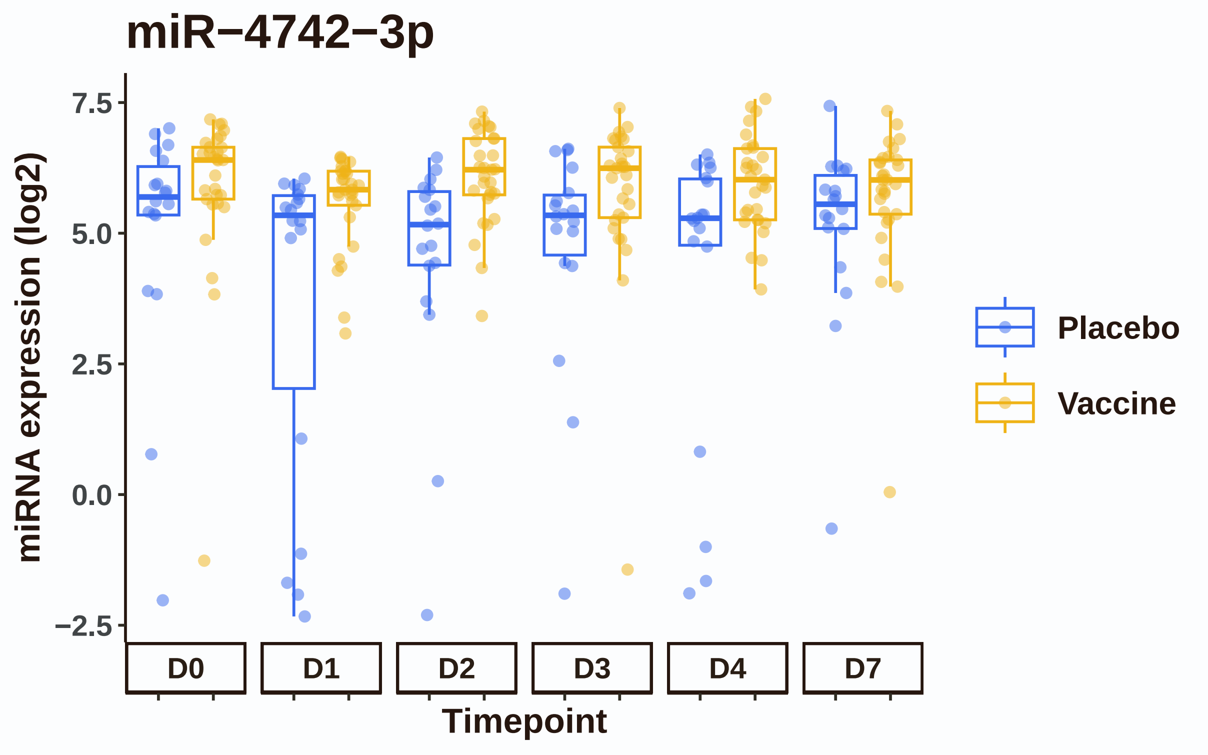

miR−4742−3p
miRNA expression (log2)
7.5
5.0
2.5
0.0
−2.5
D0
D1
D2
D3
D4
D7
Timepoint
Placebo
Vaccine

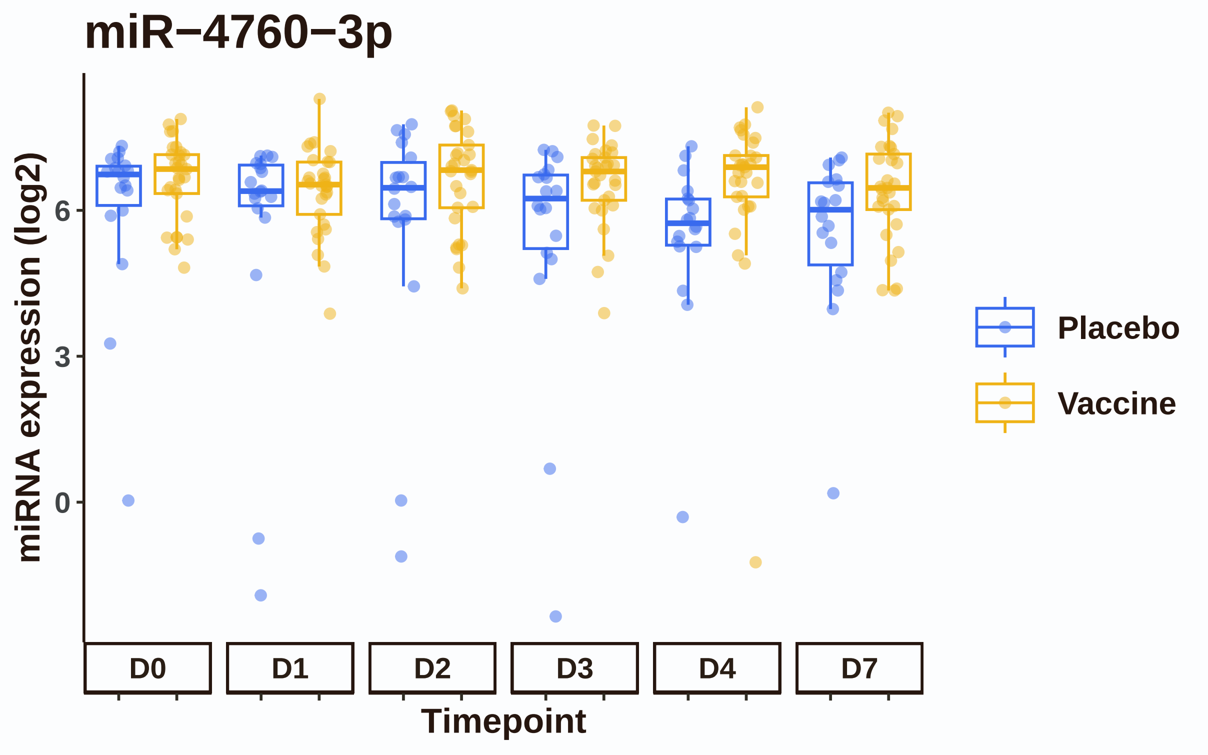
miR−4760−3p
miRNA expression (log2)
6
3
0
D0
D1
D2
D3
D4
D7
Timepoint
Placebo
Vaccine

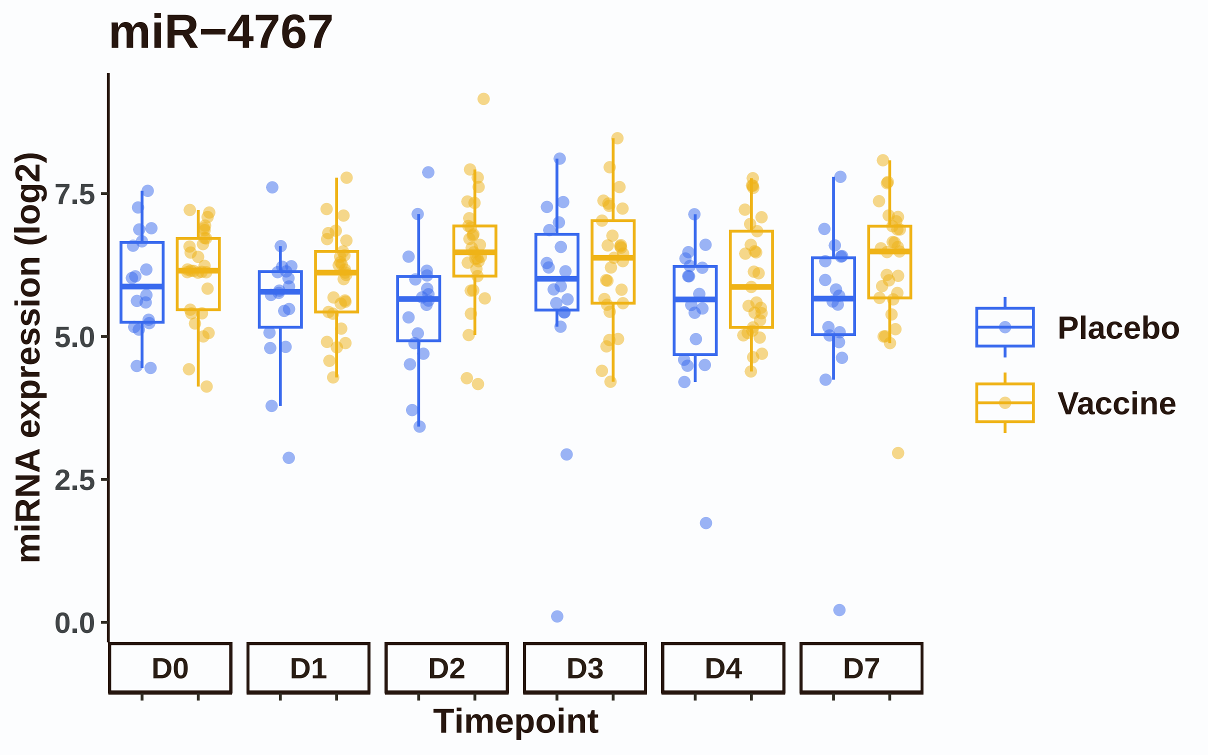
miR−4767
miRNA expression (log2)
7.5
5.0
2.5
0.0
D0
D1
D2
D3
D4
D7
Timepoint
Placebo
Vaccine

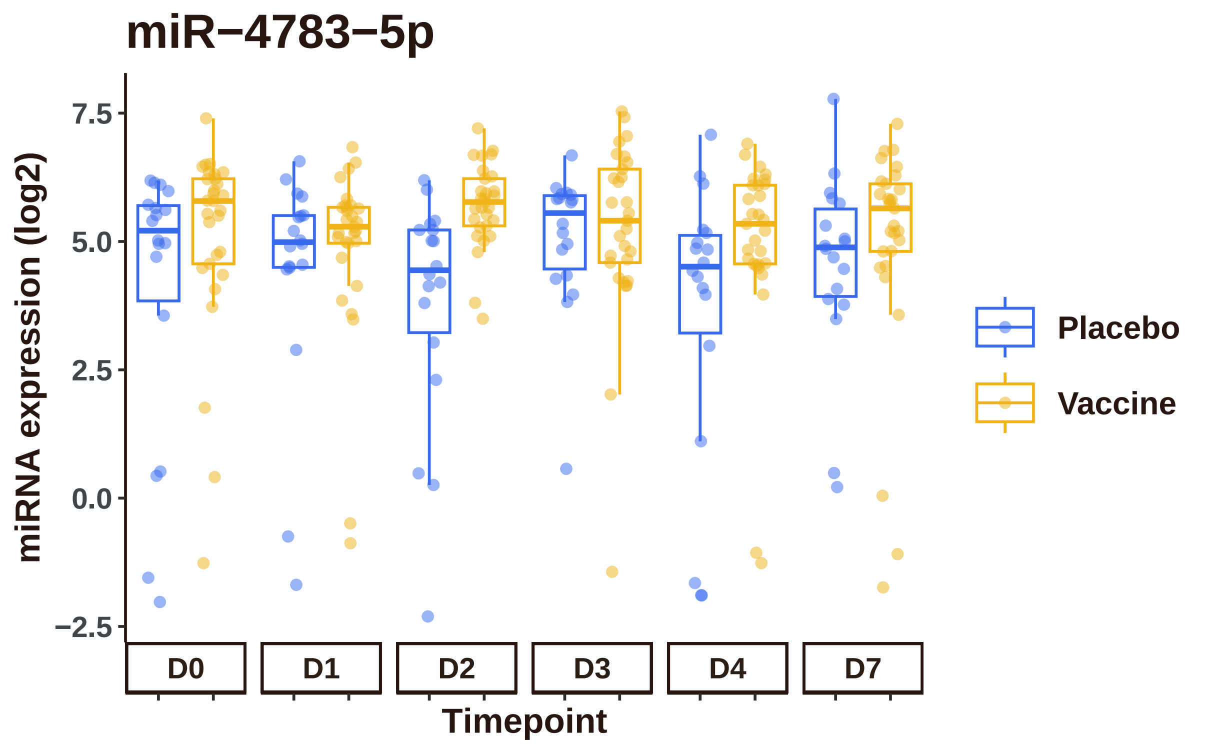

miR−4783−5p
miRNA expression (log2)
7.5
5.0
2.5
0.0
−2.5
D0
D1
D2
D3
D4
D7
Timepoint
Placebo
Vaccine

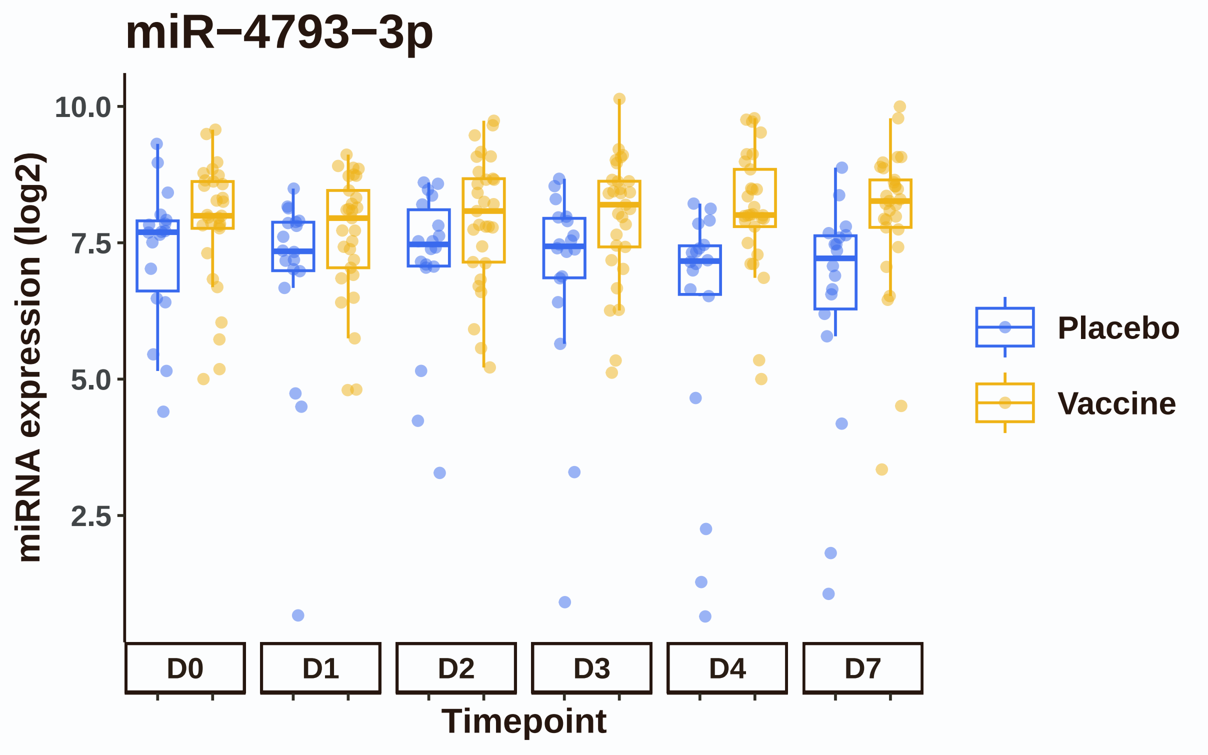
miR−4793−3p
miRNA expression (log2)
10.0
7.5
5.0
2.5
D0
D1
D2
D3
D4
D7
Timepoint
Placebo
Vaccine

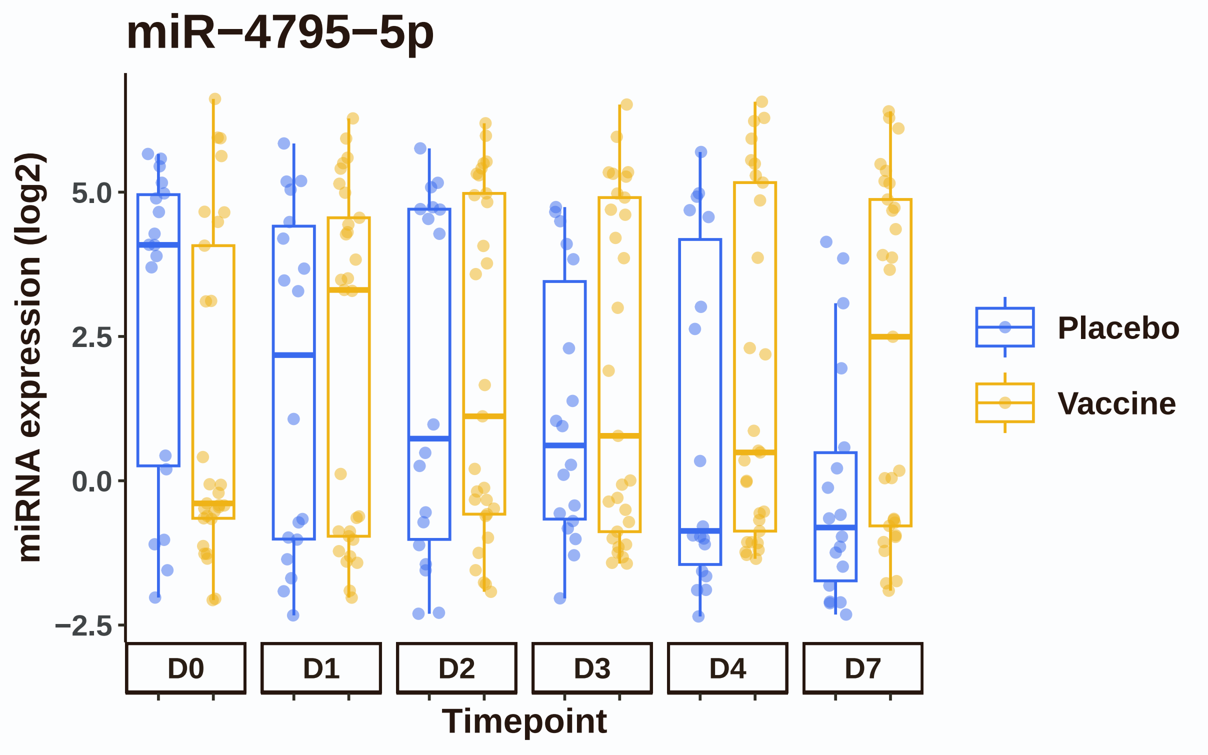
miR−4795−5p
miRNA expression (log2)
5.0
2.5
0.0
−2.5
D0
D1
D2
D3
D4
D7
Timepoint
Placebo
Vaccine

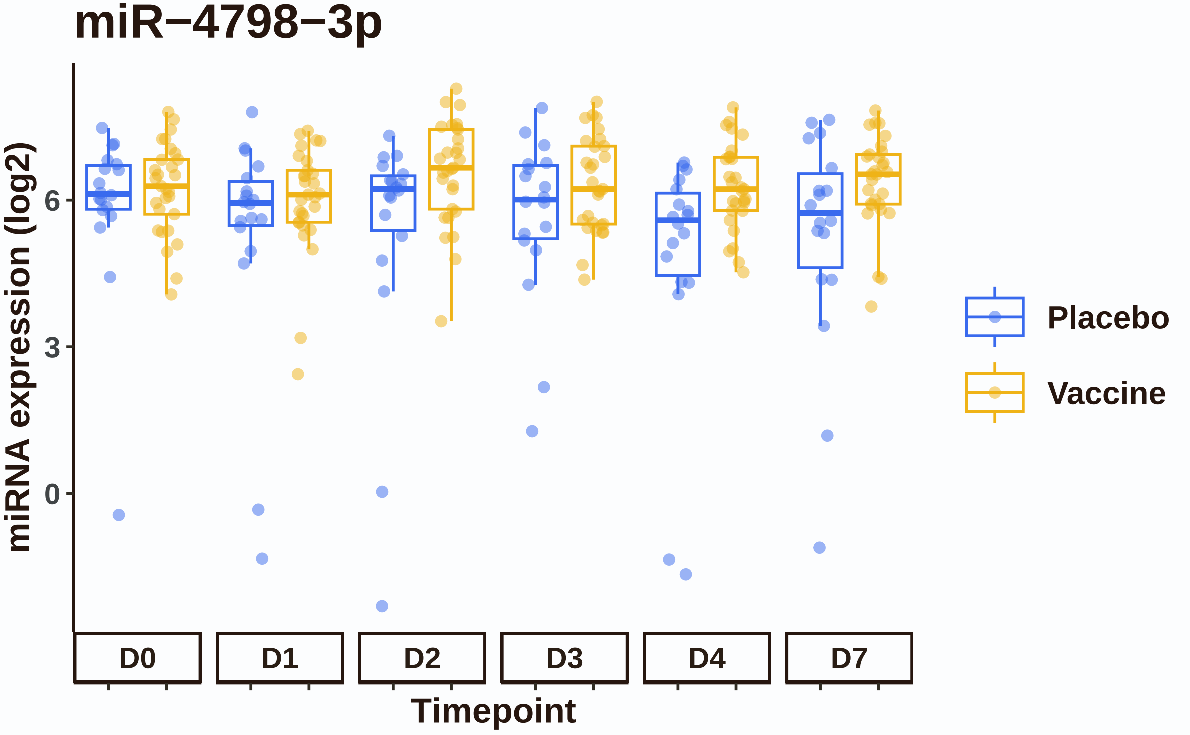

miR−4798−3p
miRNA expression (log2)
6
3
0
D0
D1
D2
D3
D4
D7
Timepoint
Placebo
Vaccine

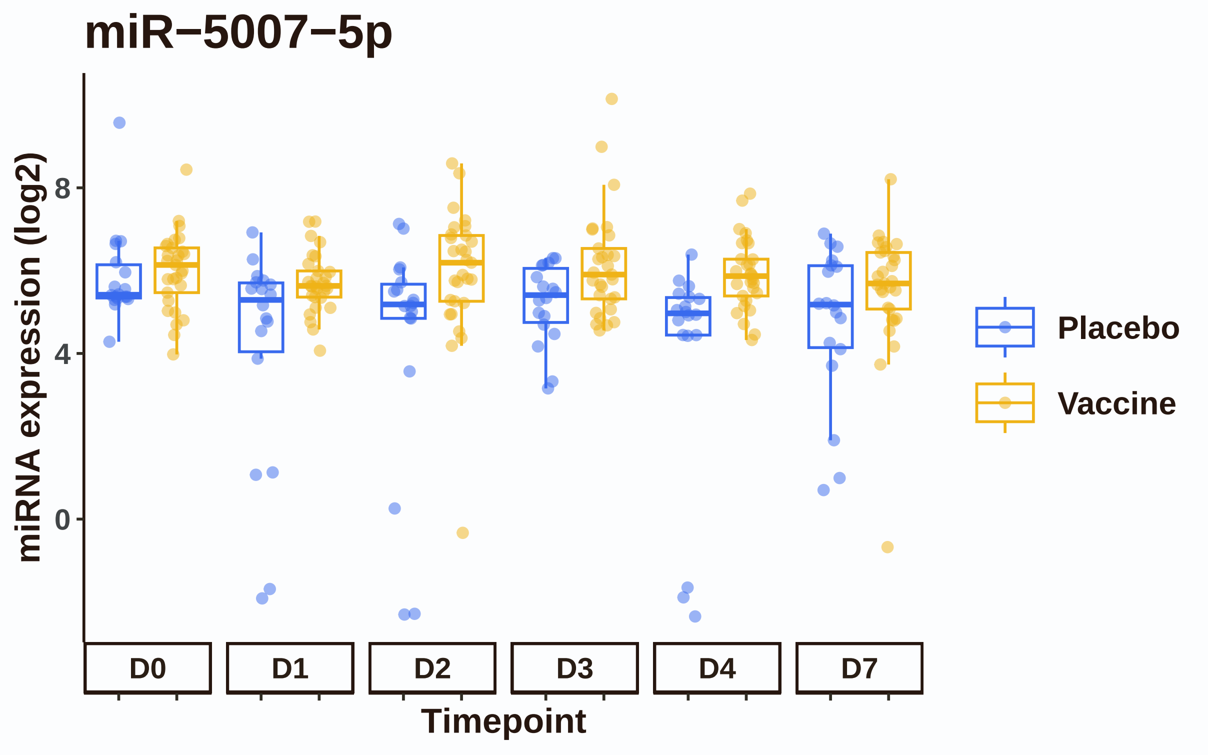

miR−5007−5p
miRNA expression (log2)
8
4
0
D0
D1
D2
D3
D4
D7
Timepoint
Placebo
Vaccine

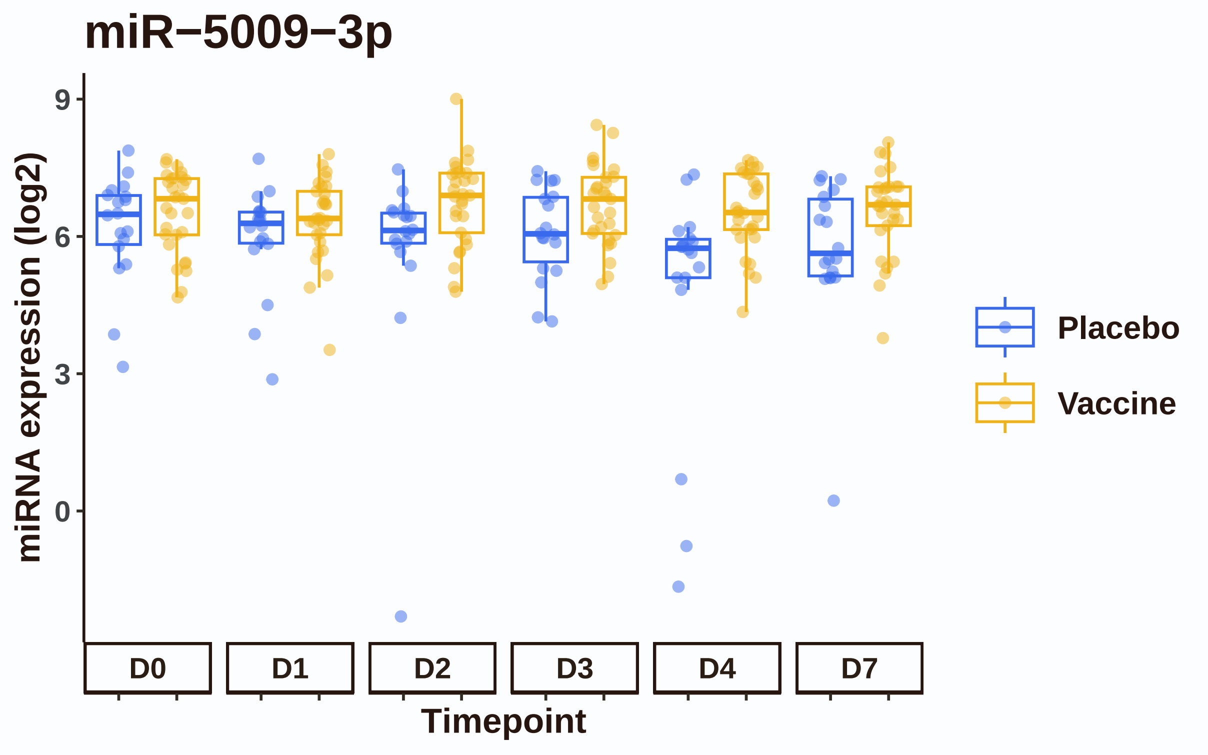

miR−5009−3p
miRNA expression (log2)
9
6
3
0
D0
D1
D2
D3
D4
D7
Timepoint
Placebo
Vaccine

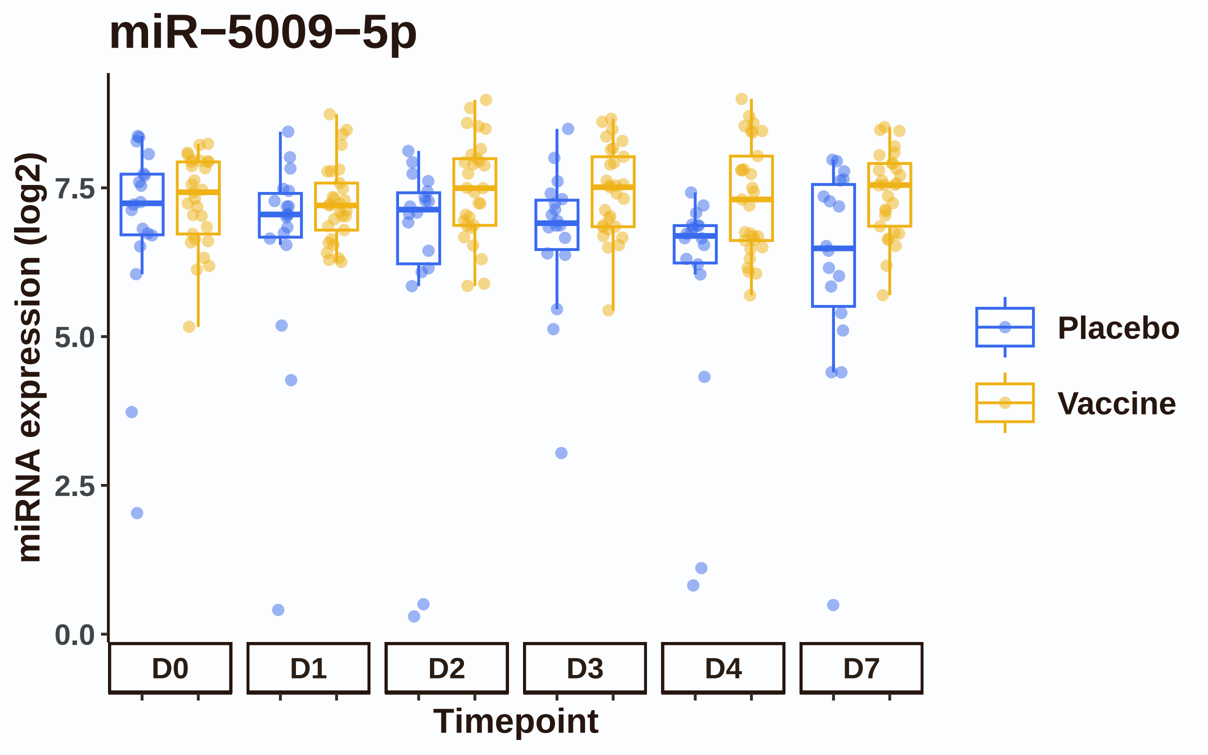

miR−5009−5p
miRNA expression (log2)
7.5
5.0
2.5
0.0
D0
D1
D2
D3
D4
D7
Timepoint
Placebo
Vaccine

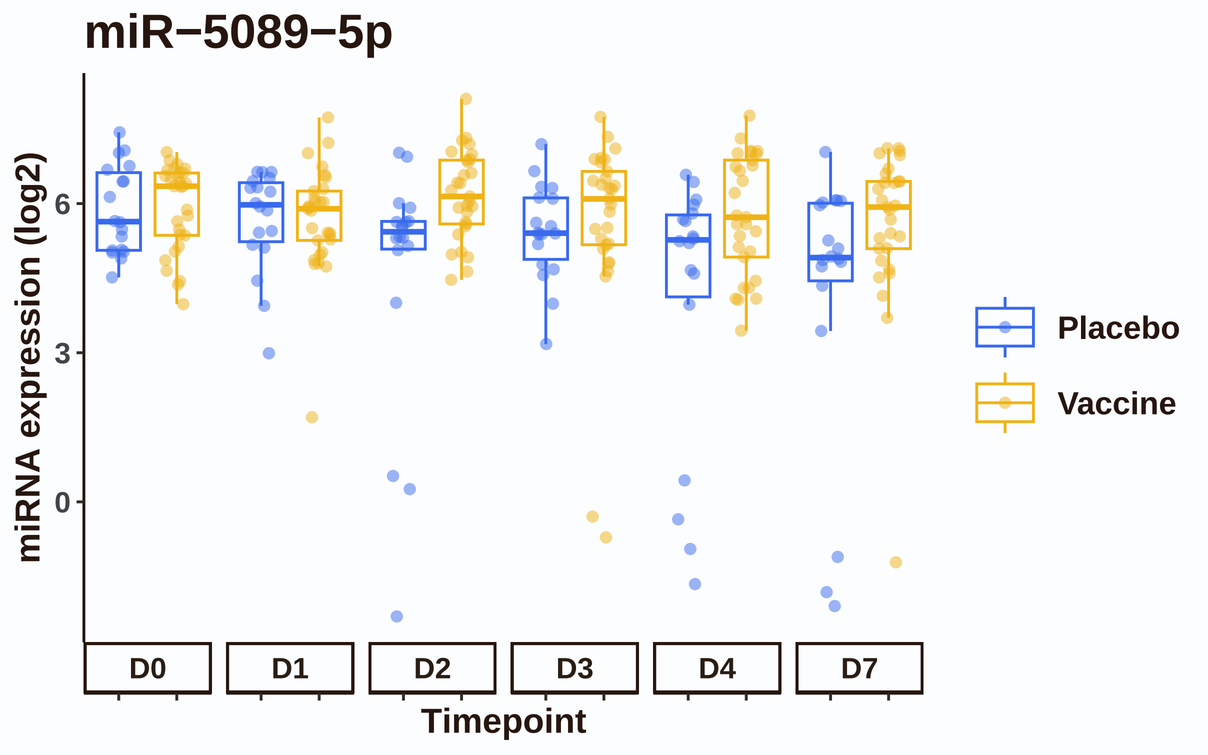
miR−5089−5p
miRNA expression (log2)
0
3
6
D0
D1
D2
D3
D4
D7
Timepoint
Placebo
Vaccine

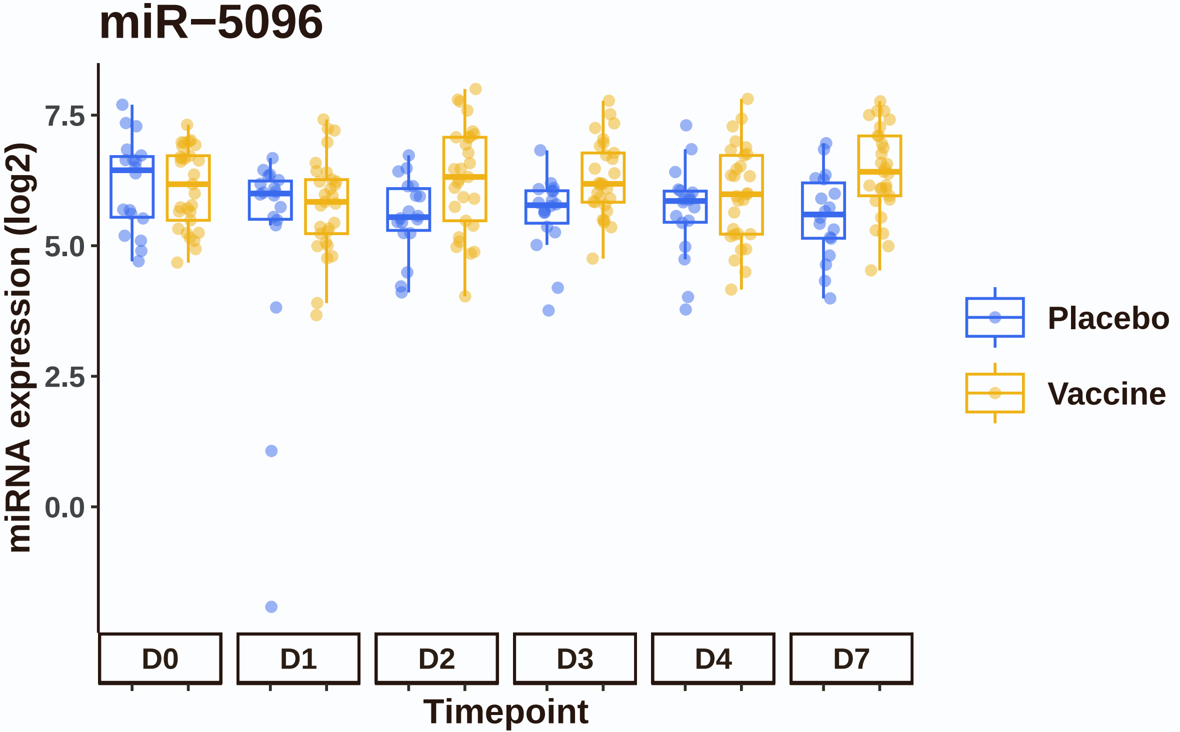

miR−5096
miRNA expression (log2)
7.5
5.0
2.5
0.0
D0
D1
D2
D3
D4
D7
Timepoint
Placebo
Vaccine

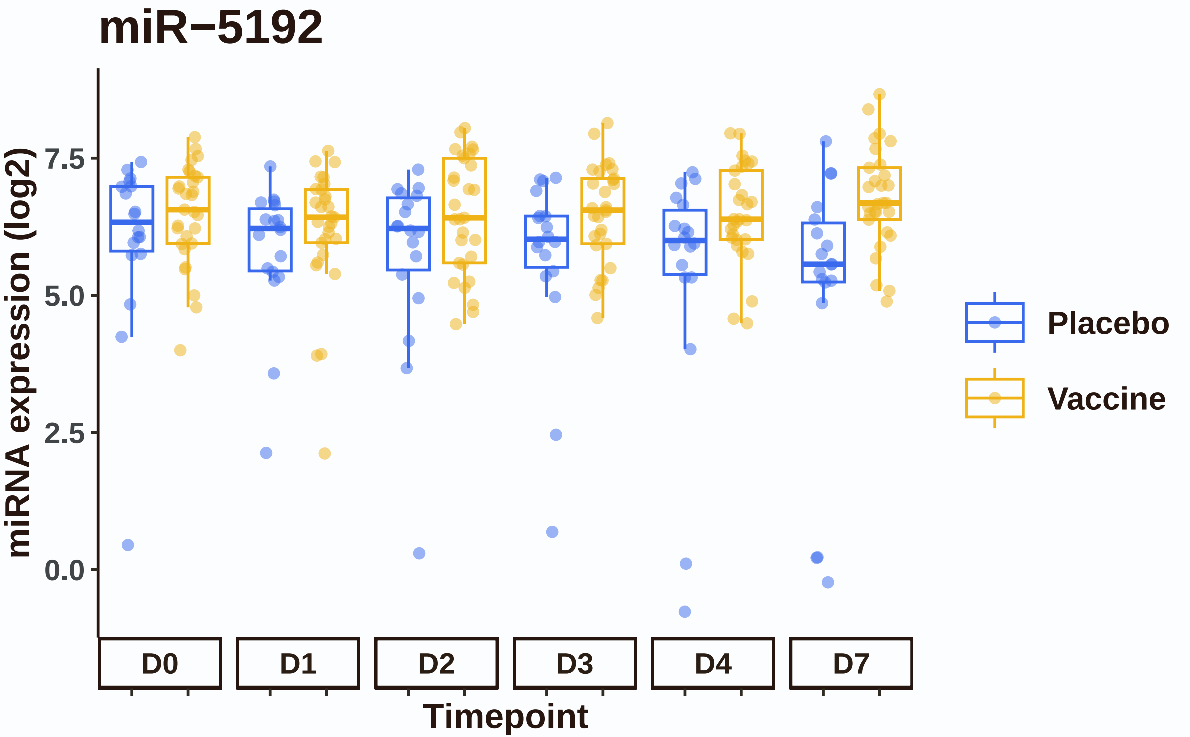

miR−5192
miRNA expression (log2)
7.5
5.0
2.5
0.0
D0
D1
D2
D3
D4
D7
Timepoint
Placebo
Vaccine

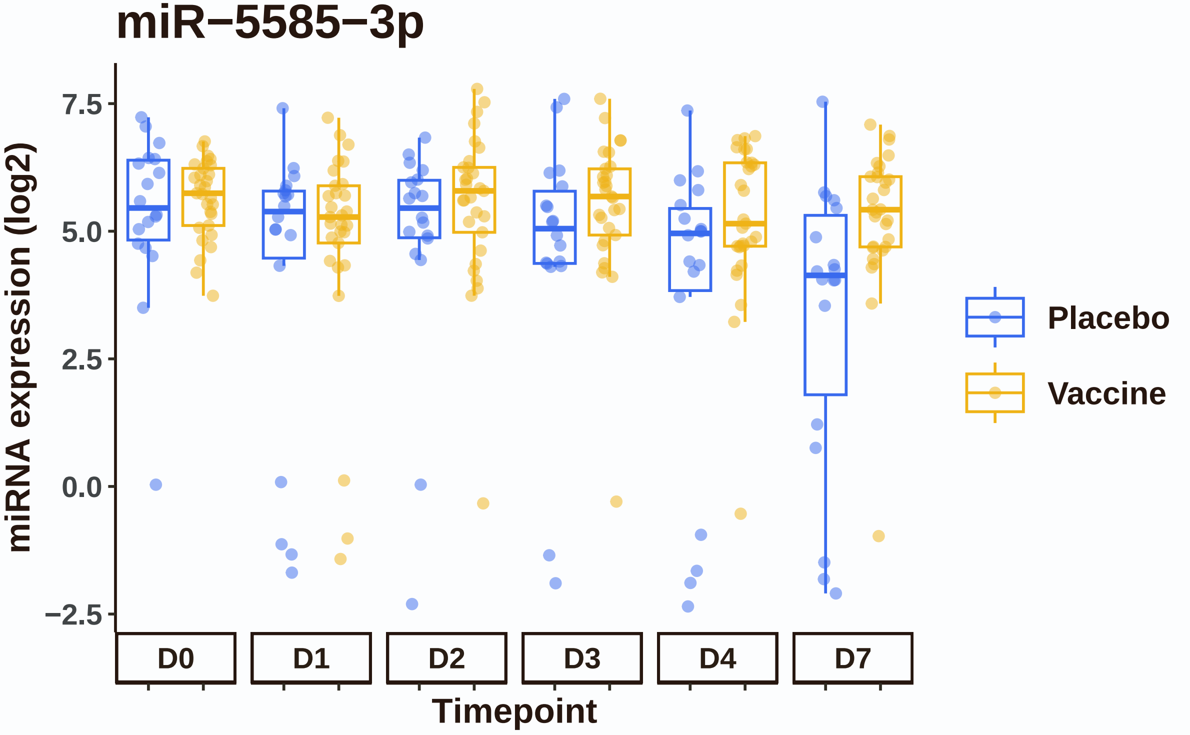
miR−5585−3p
miRNA expression (log2)
7.5
5.0
2.5
0.0
−2.5
D0
D1
D2
D3
D4
D7
Timepoint
Placebo
Vaccine

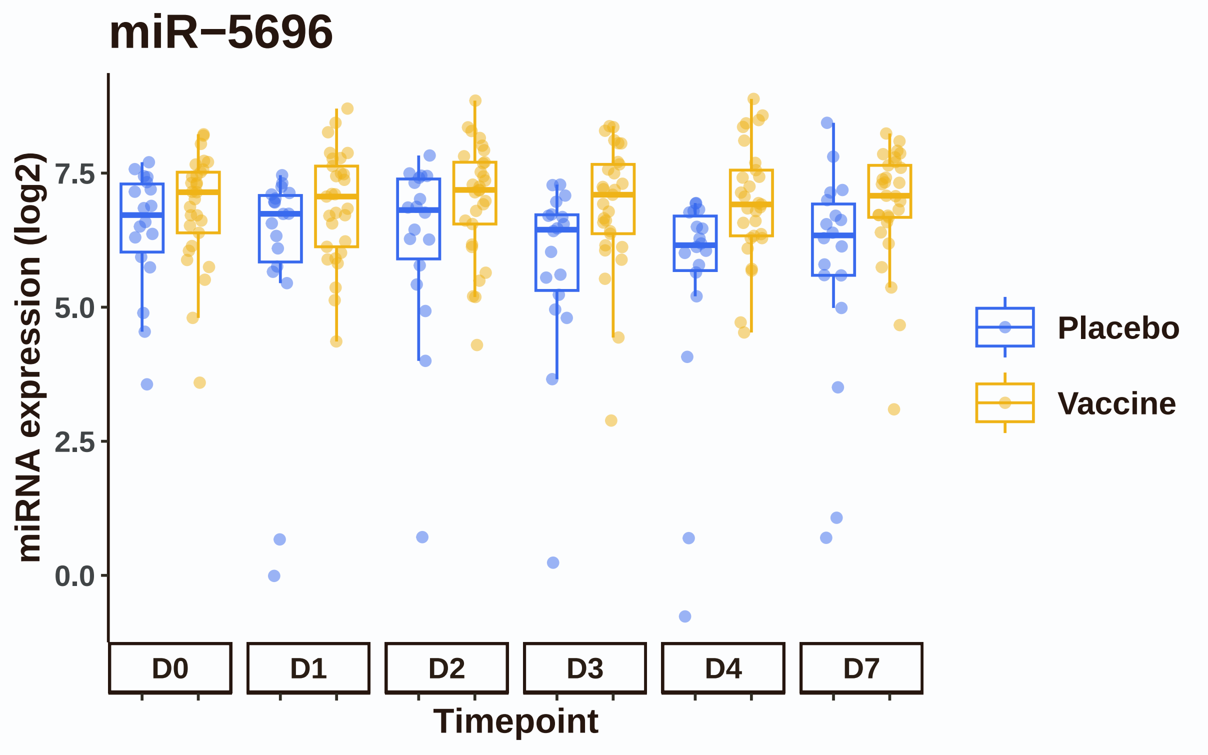

miR−5696
miRNA expression (log2)
7.5
5.0
2.5
0.0
D0
D1
D2
D3
D4
D7
Timepoint
Placebo
Vaccine

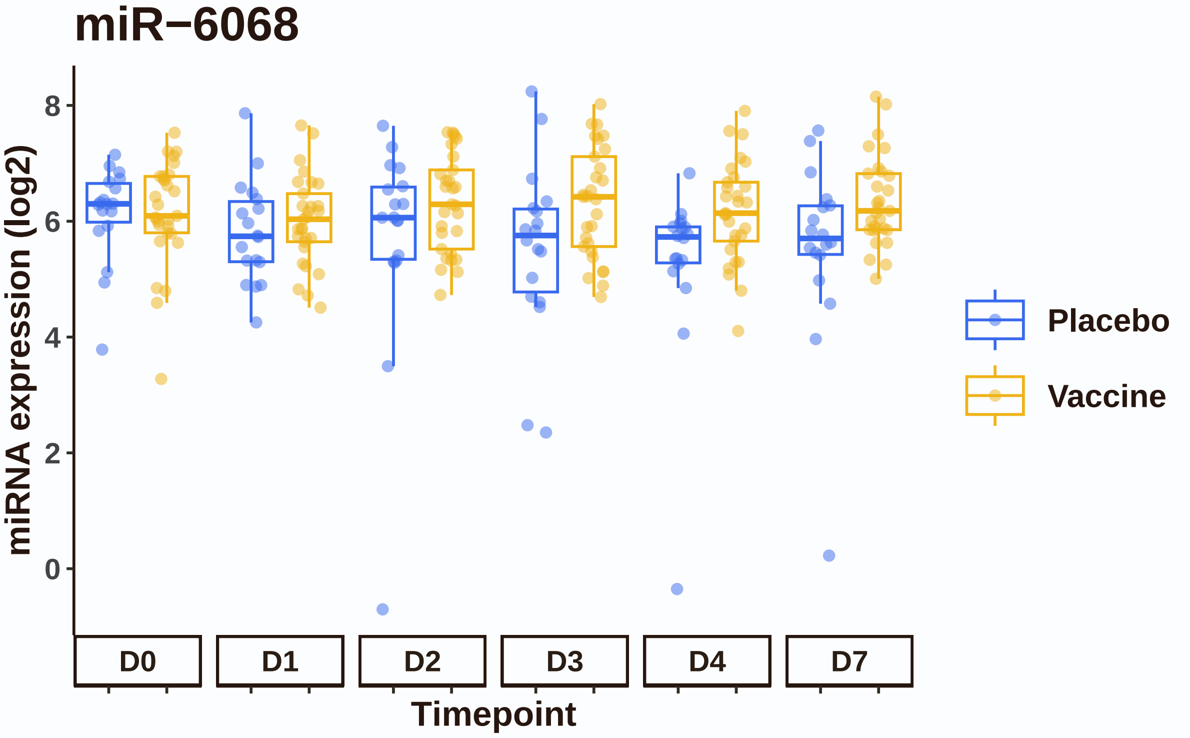

miR−6068
miRNA expression (log2)
8
6
4
2
0
D0
D1
D2
D3
D4
D7
Timepoint
Placebo
Vaccine

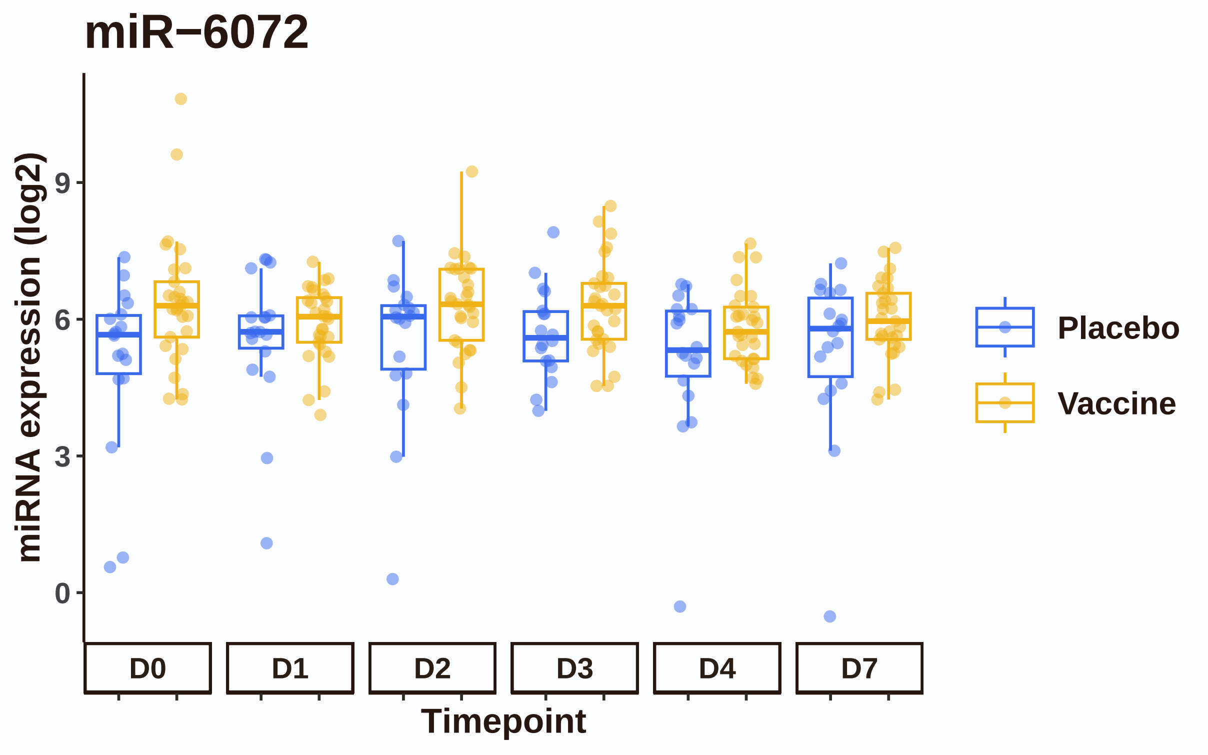

miR−6072
miRNA expression (log2)
0
3
6
9
D0
D1
D2
D3
D4
D7
Timepoint
Placebo
Vaccine

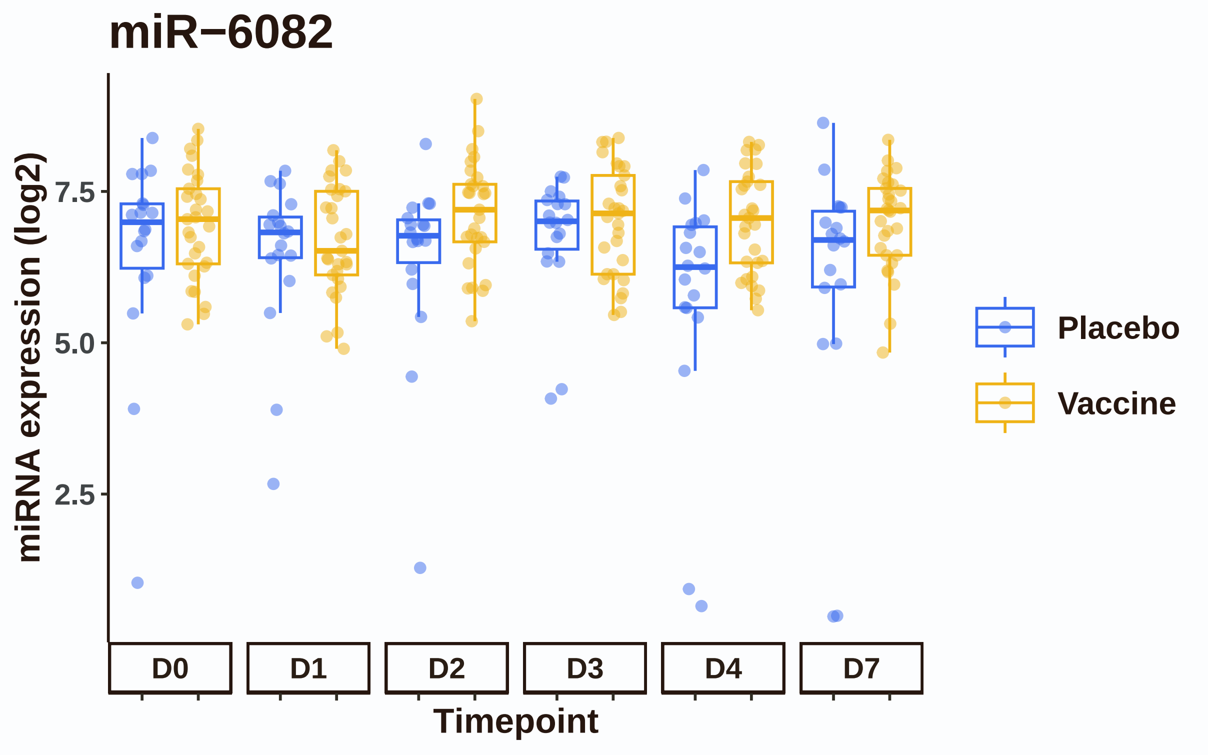
miR−6082
miRNA expression (log2)
7.5
5.0
2.5
D0
D1
D2
D3
D4
D7
Timepoint
Placebo
Vaccine

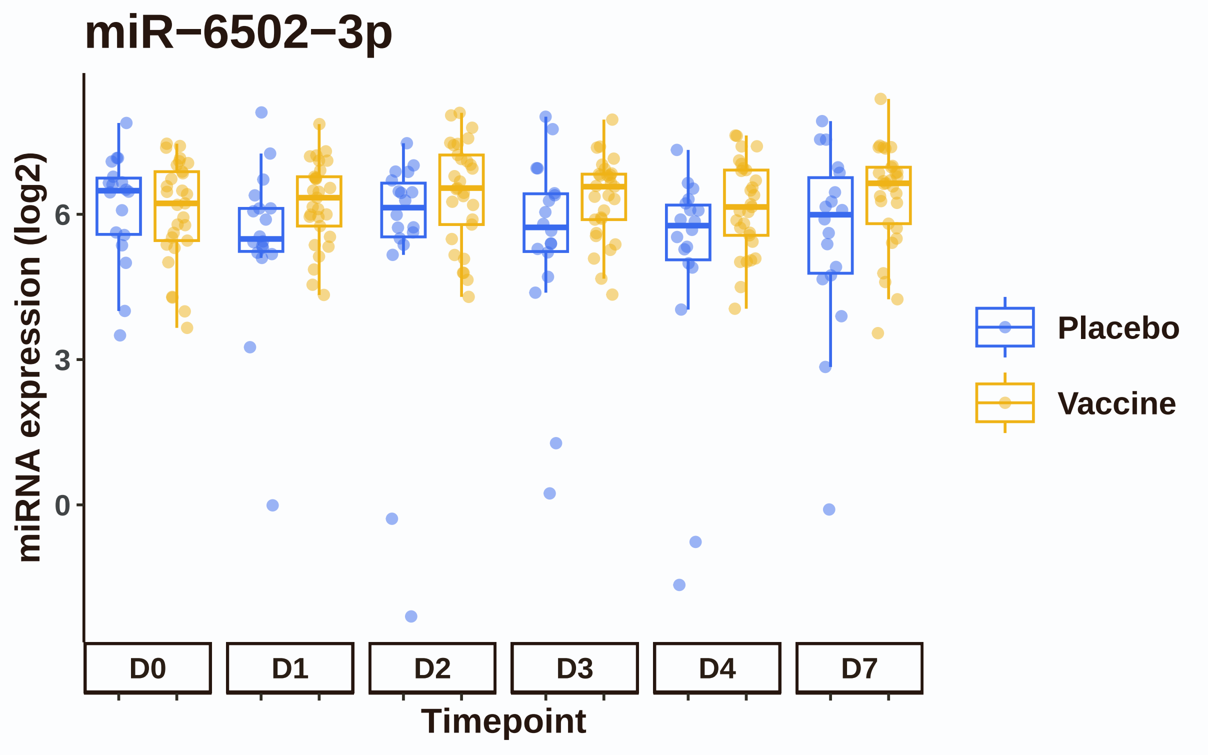

miR−6502−3p
miRNA expression (log2)
6
3
0
D0
D1
D2
D3
D4
D7
Timepoint
Placebo
Vaccine

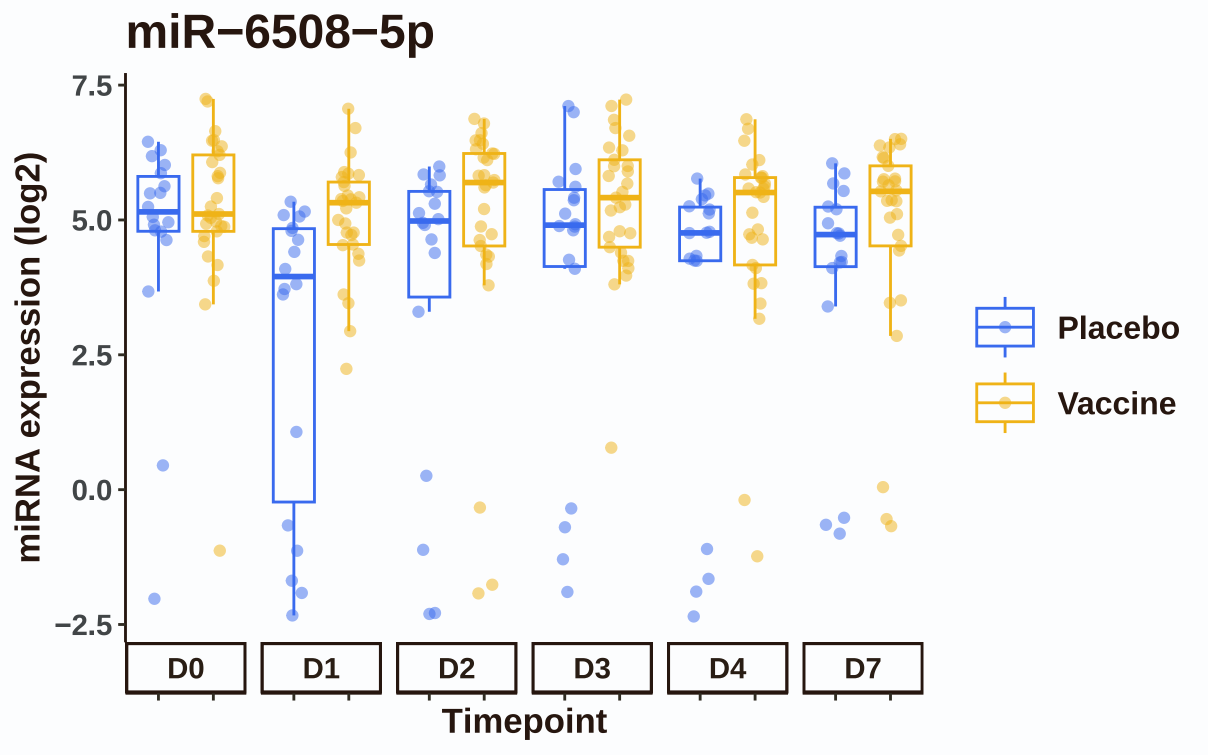
miR−6508−5p
miRNA expression (log2)
7.5
5.0
2.5
0.0
−2.5
D0
D1
D2
D3
D4
D7
Timepoint
Placebo
Vaccine

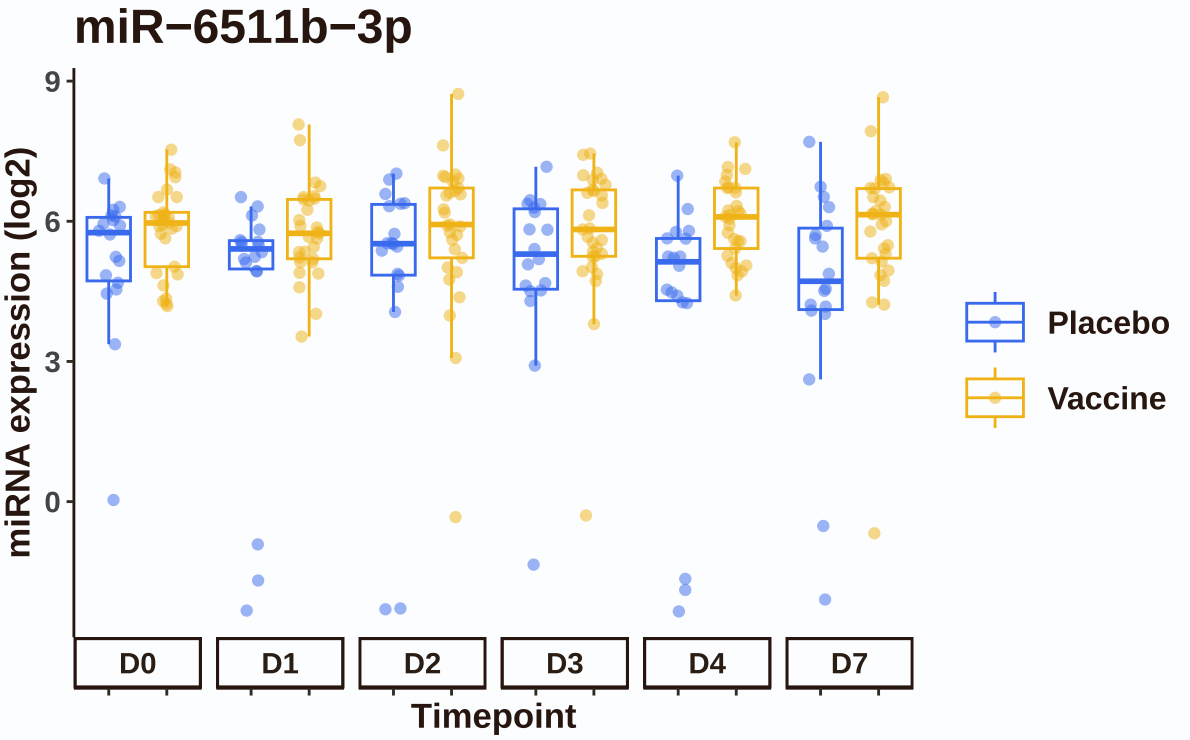

miR−6511b−3p
miRNA expression (log2)
9
6
3
0
D0
D1
D2
D3
D4
D7
Timepoint
Placebo
Vaccine

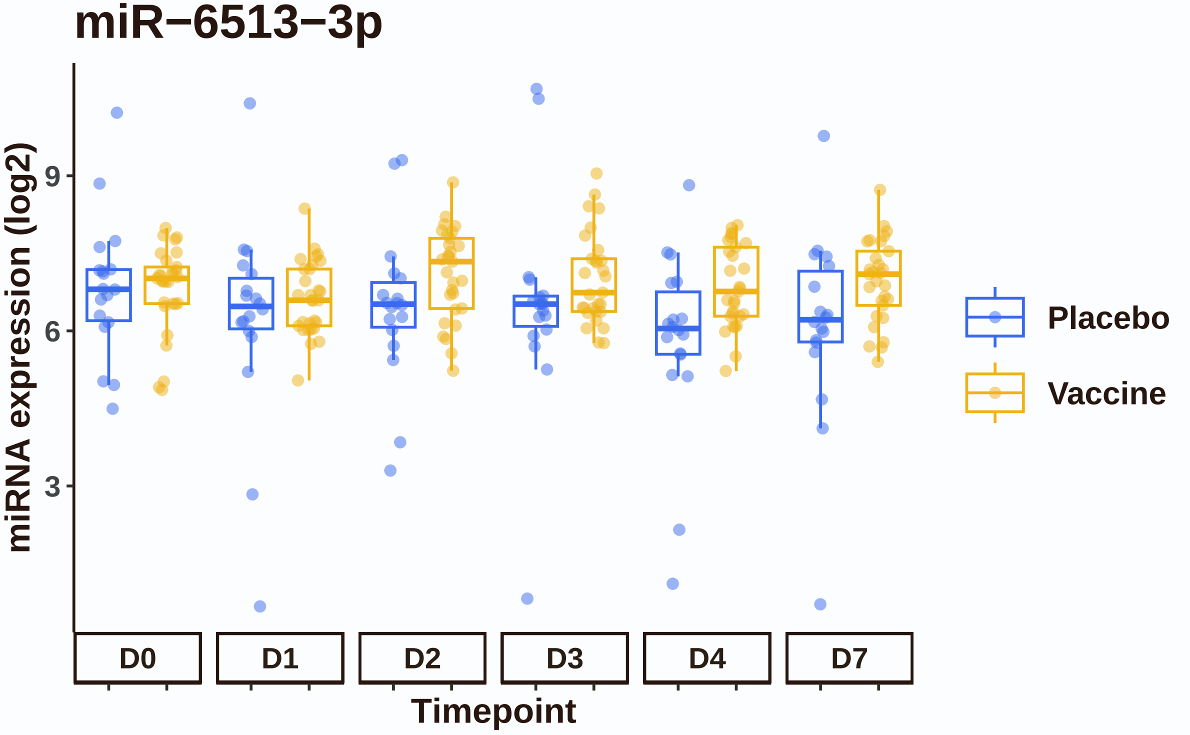
miR−6513−3p
miRNA expression (log2)
9
6
3
D0
D1
D2
D3
D4
D7
Timepoint
Placebo
Vaccine

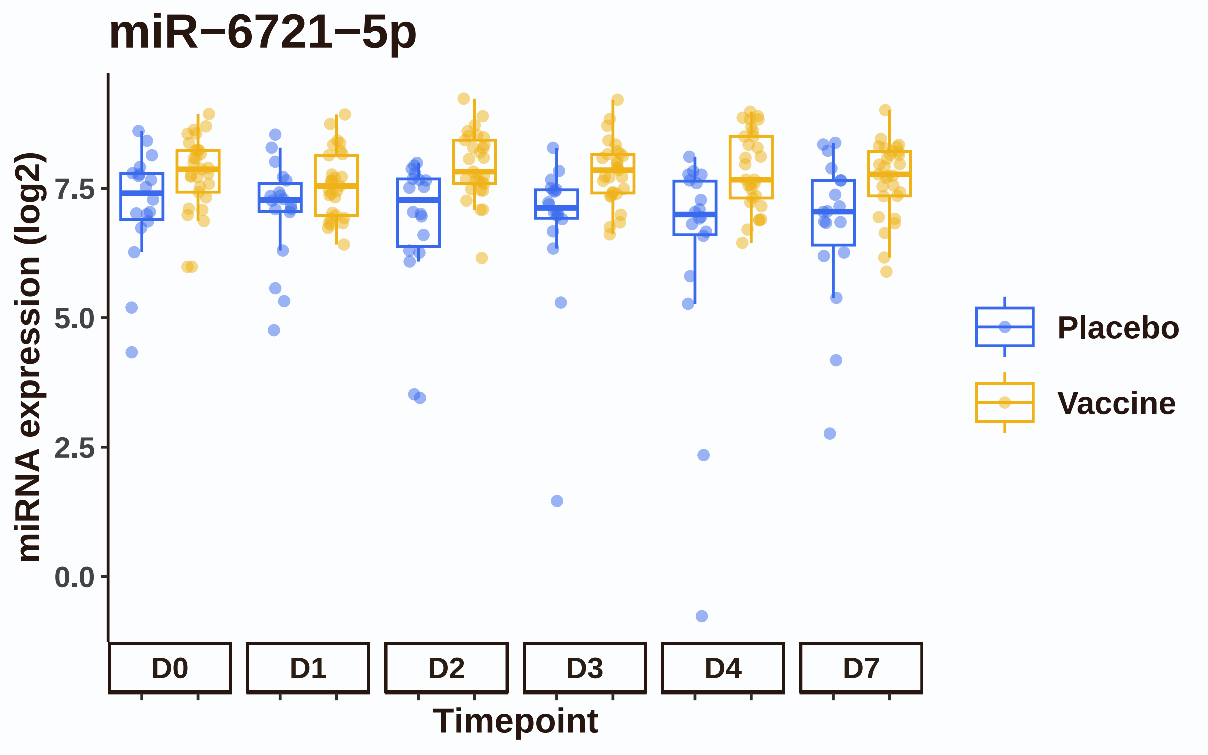

miR−6721−5p
miRNA expression (log2)
7.5
5.0
2.5
0.0
D0
D1
D2
D3
D4
D7
Timepoint
Placebo
Vaccine

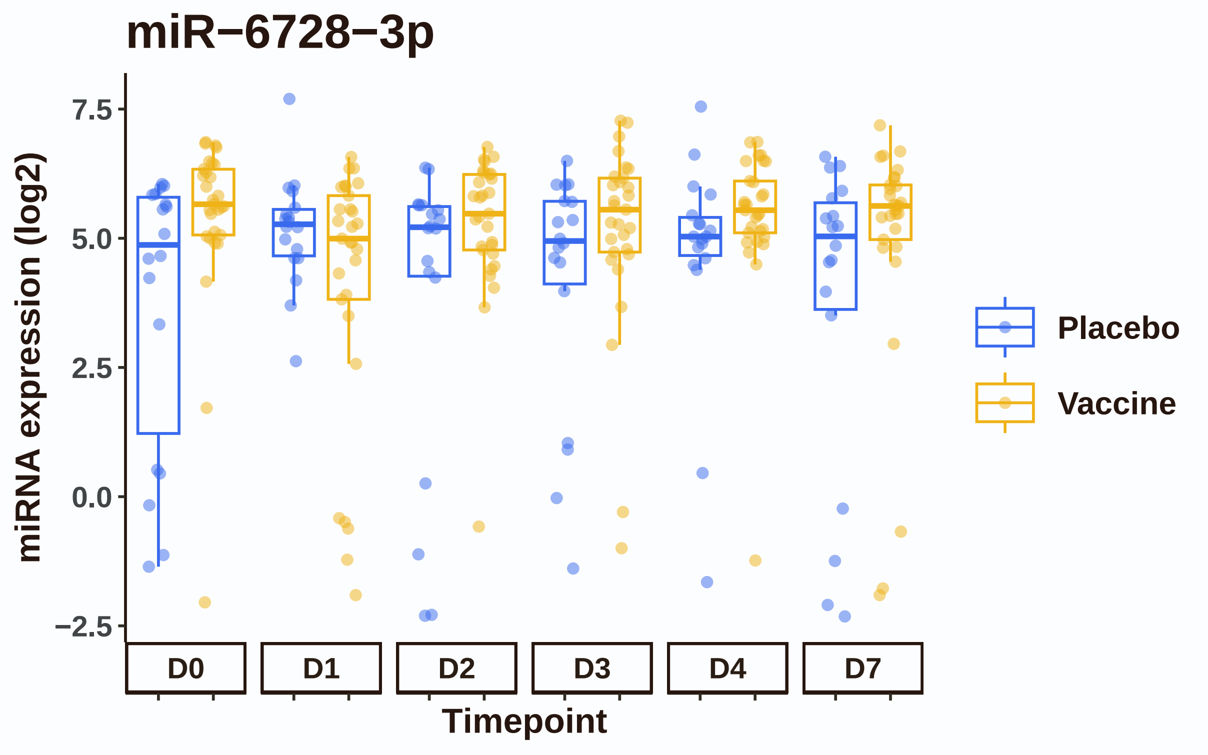

miR−6728−3p
miRNA expression (log2)
7.5
5.0
2.5
0.0
−2.5
D0
D1
D2
D3
D4
D7
Timepoint
Placebo
Vaccine

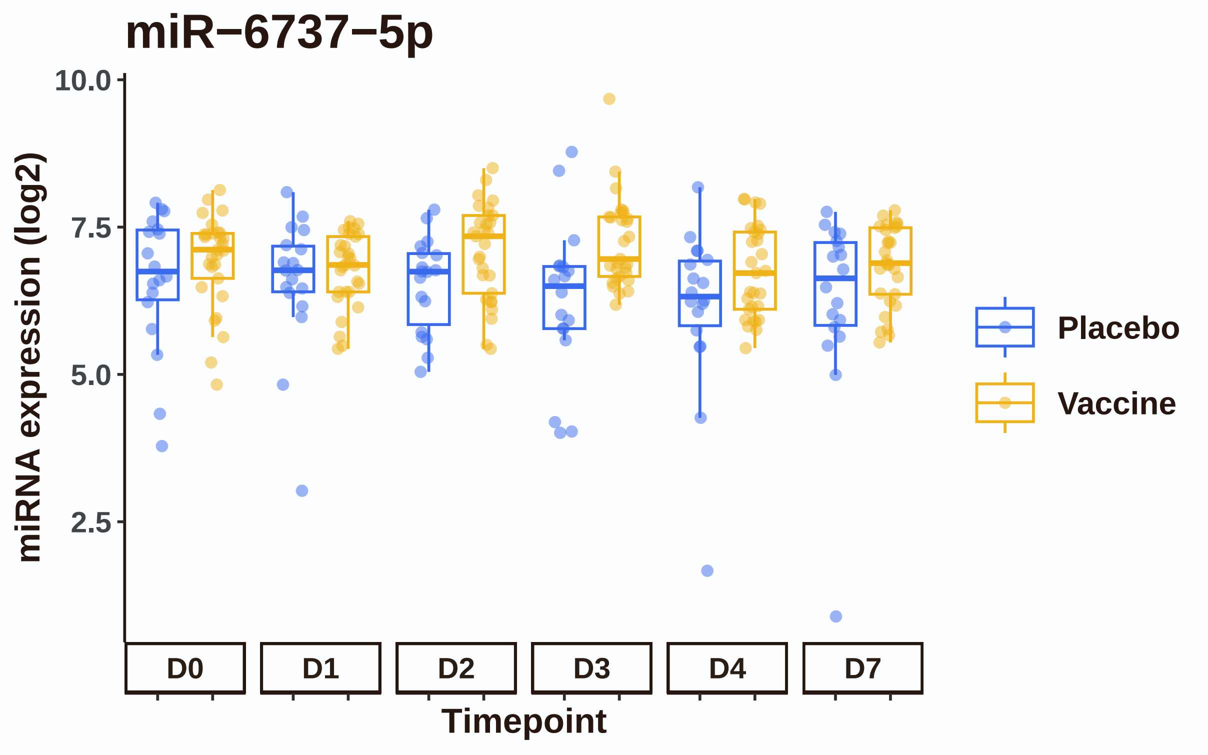
miR−6737−5p
miRNA expression (log2)
10.0
7.5
5.0
2.5
D0
D1
D2
D3
D4
D7
Timepoint
Placebo
Vaccine

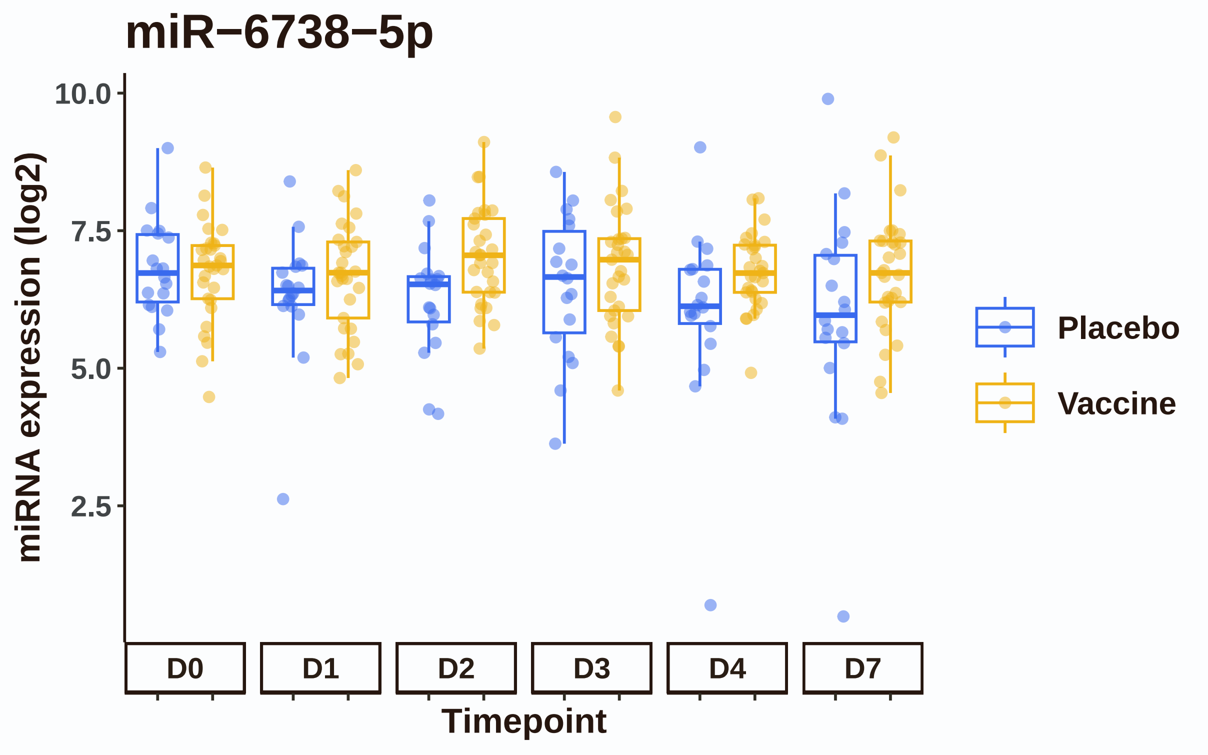

miR−6738−5p
miRNA expression (log2)
10.0
7.5
5.0
2.5
D0
D1
D2
D3
D4
D7
Timepoint
Placebo
Vaccine

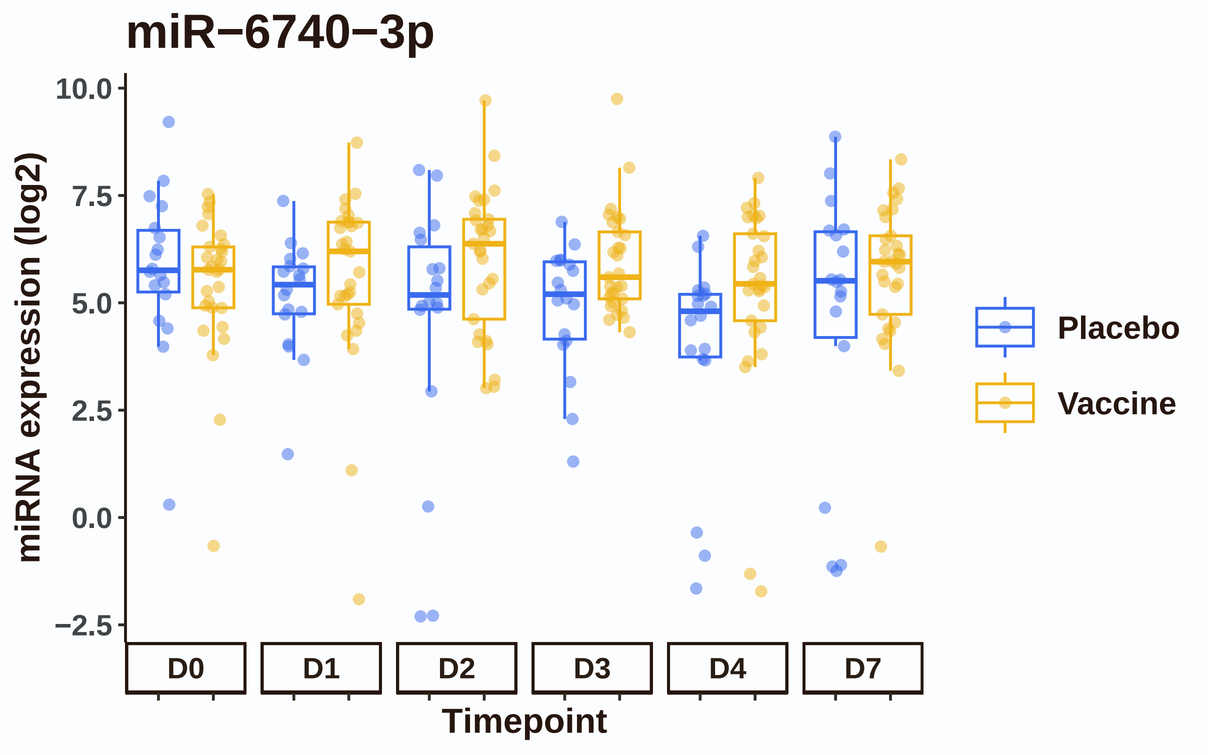

miR−6740−3p
miRNA expression (log2)
10.0
7.5
5.0
2.5
0.0
−2.5
D0
D1
D2
D3
D4
D7
Timepoint
Placebo
Vaccine

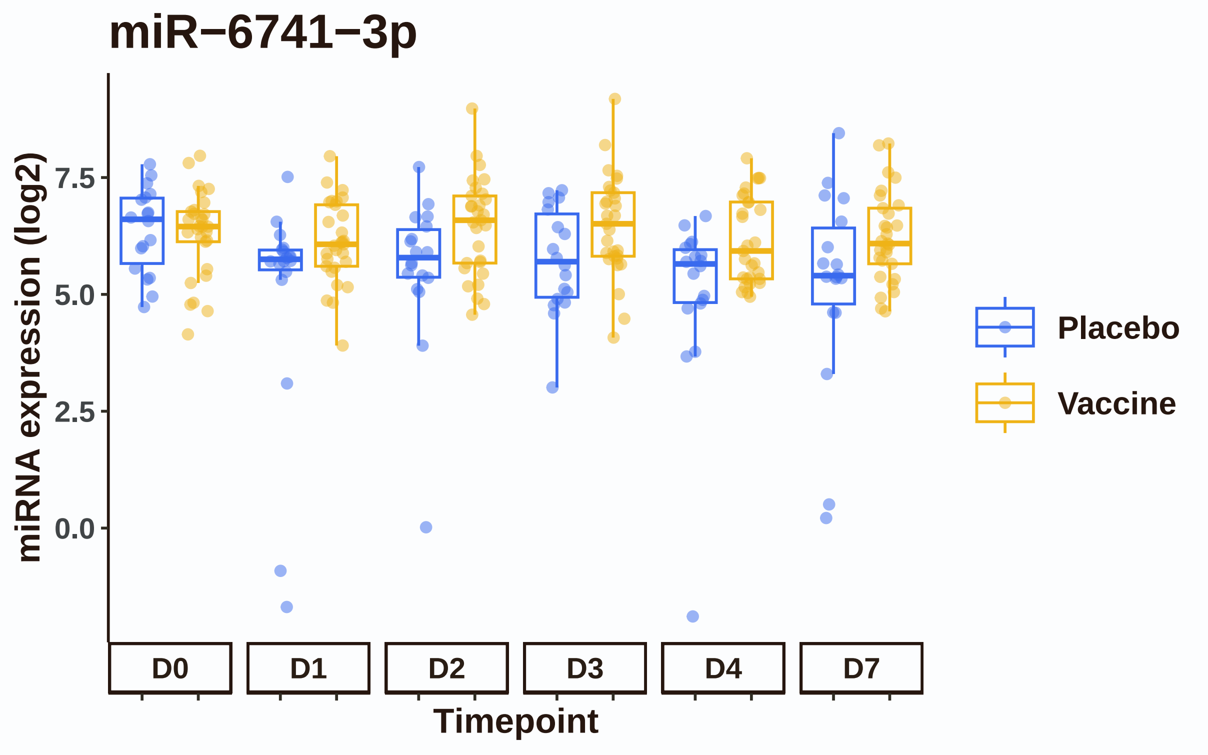

miR−6741−3p
miRNA expression (log2)
7.5
5.0
2.5
0.0
D0
D1
D2
D3
D4
D7
Timepoint
Placebo
Vaccine

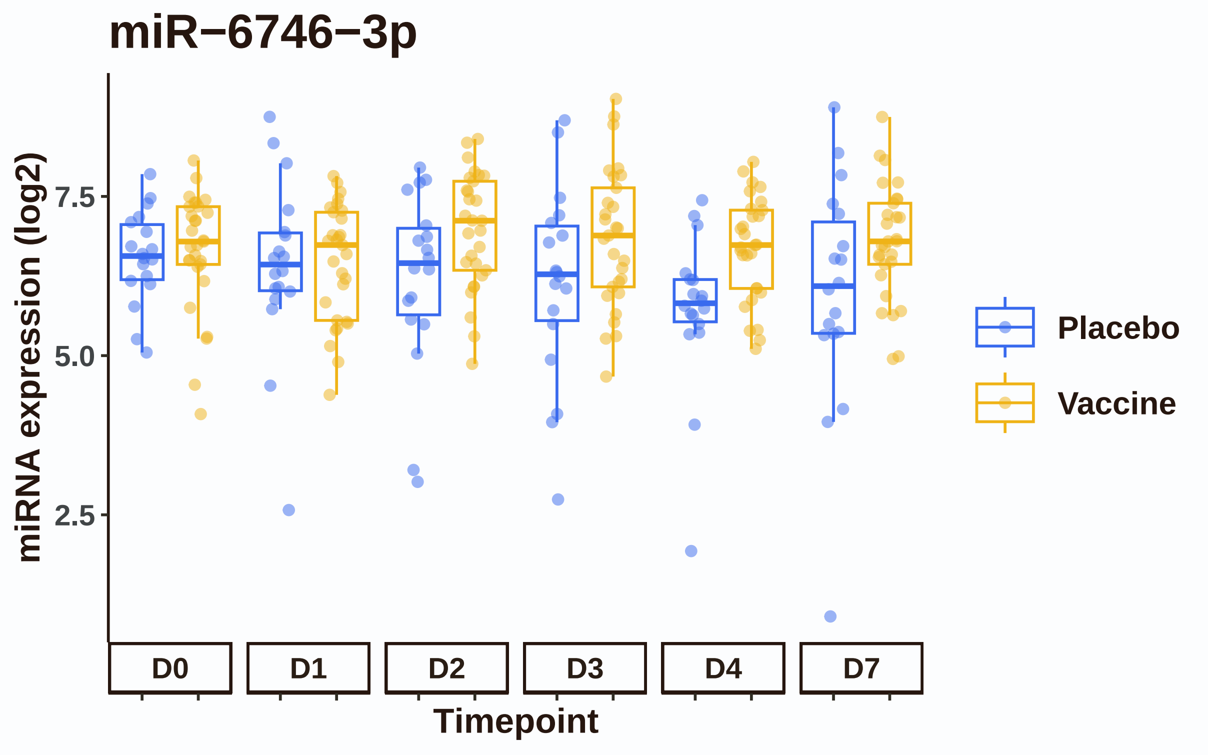
miR−6746−3p
miRNA expression (log2)
7.5
5.0
2.5
D0
D1
D2
D3
D4
D7
Timepoint
Placebo
Vaccine

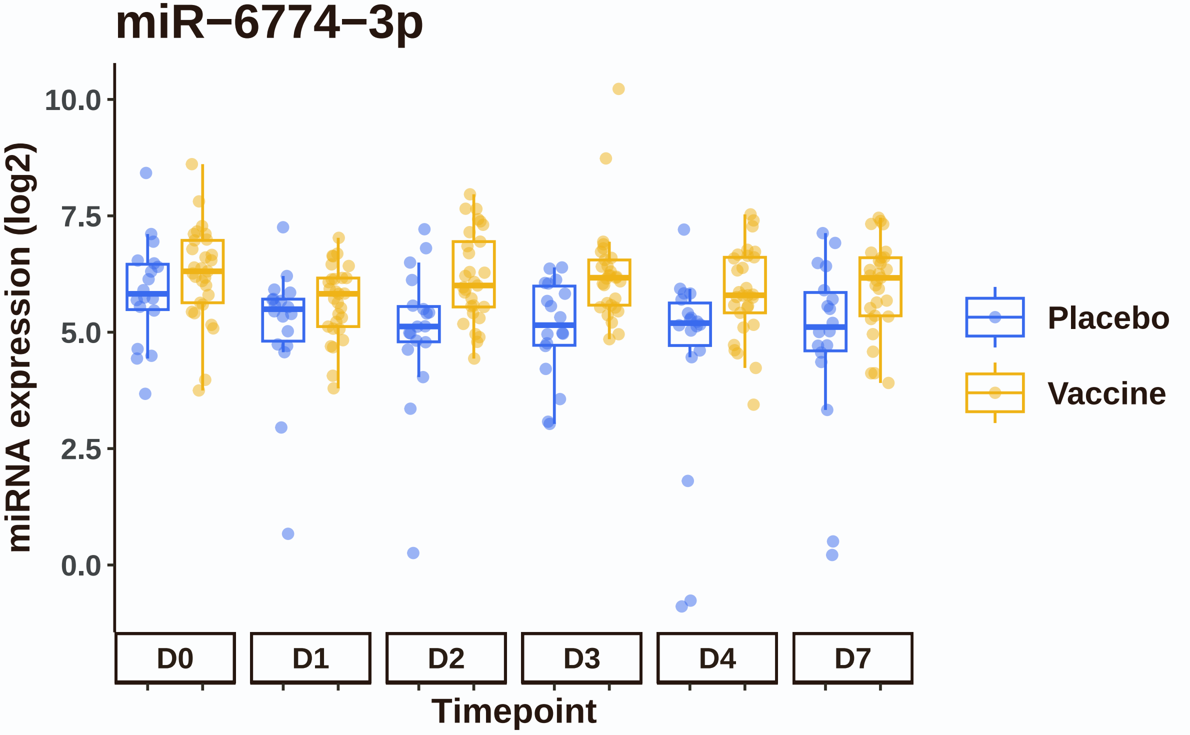
miR−6774−3p
miRNA expression (log2)
10.0
7.5
5.0
2.5
0.0
D0
D1
D2
D3
D4
D7
Timepoint
Placebo
Vaccine

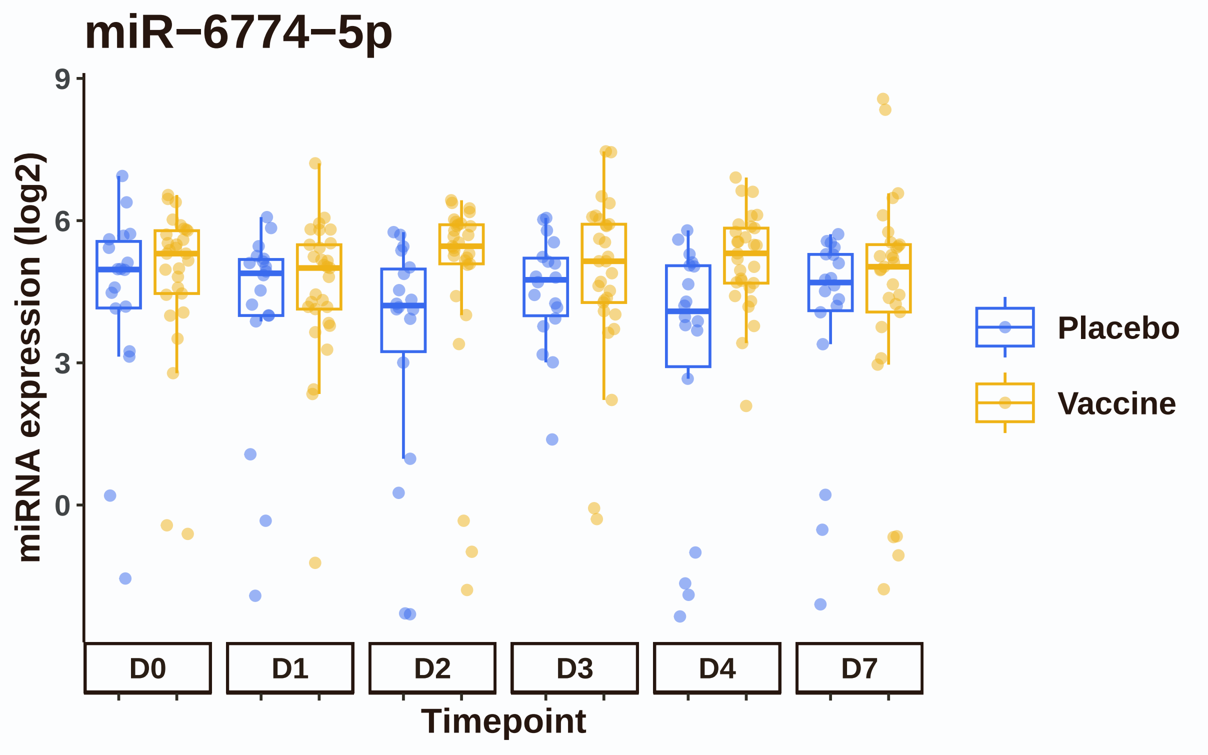
miR−6774−5p
miRNA expression (log2)
9
6
3
0
D0
D1
D2
D3
D4
D7
Timepoint
Placebo
Vaccine

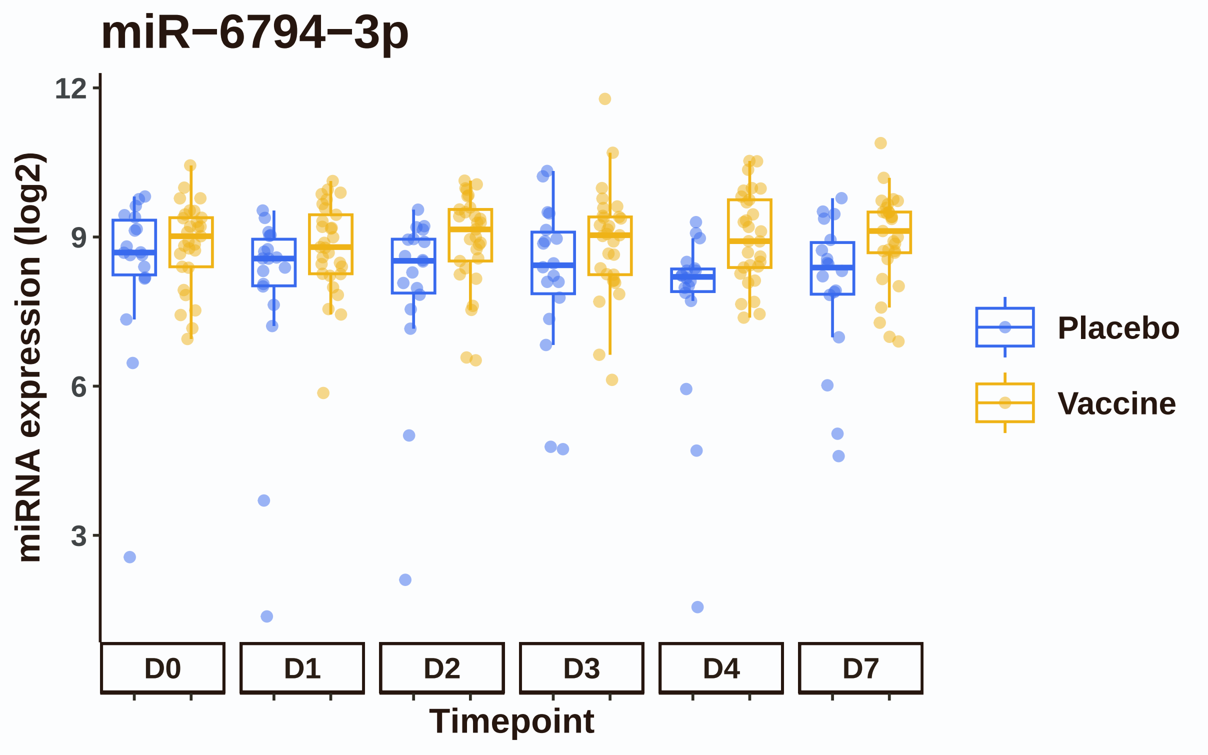
miR−6794−3p
miRNA expression (log2)
12
9
6
3
D0
D1
D2
D3
D4
D7
Timepoint
Placebo
Vaccine

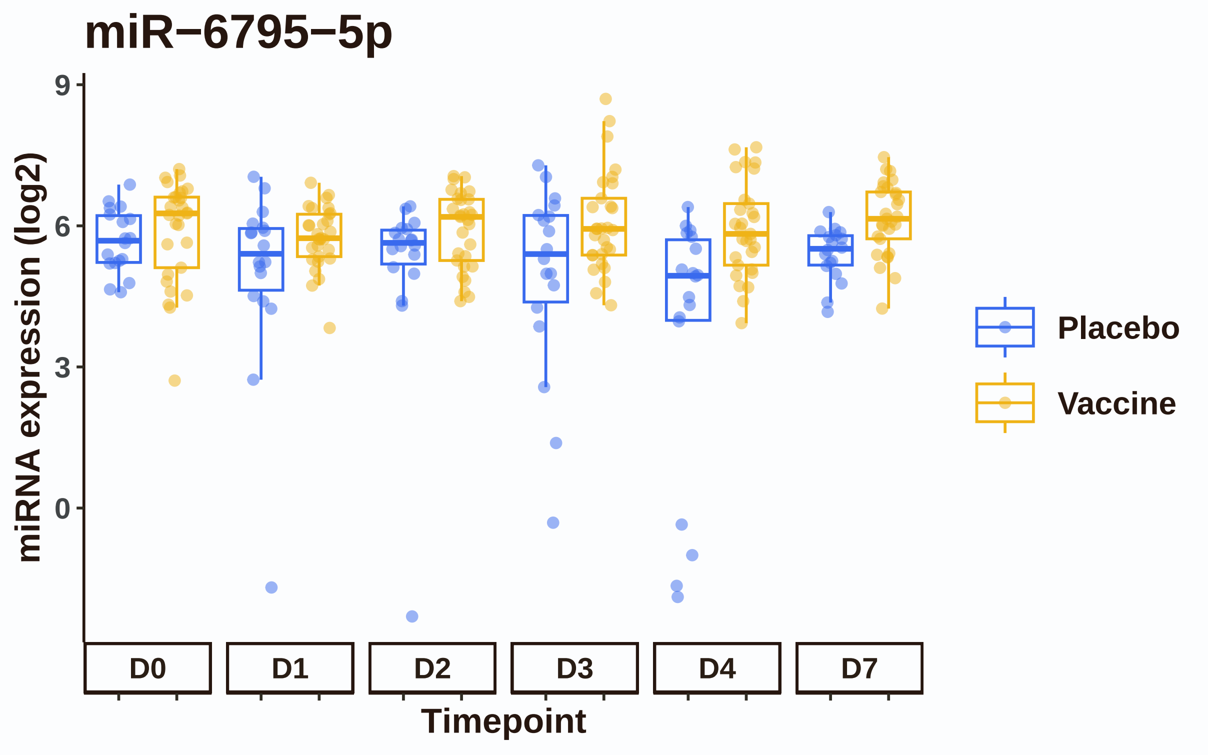
miR−6795−5p
miRNA expression (log2)
9
6
3
0
D0
D1
D2
D3
D4
D7
Timepoint
Placebo
Vaccine

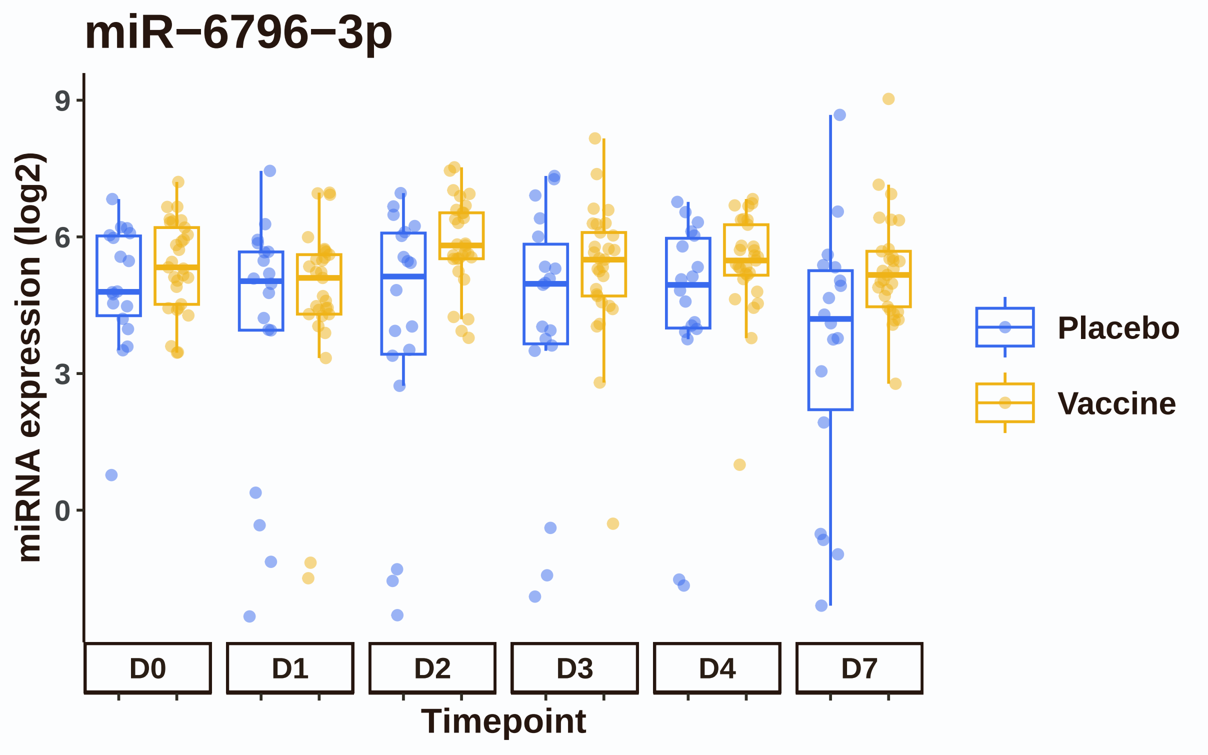
miR−6796−3p
miRNA expression (log2)
9
6
3
0
D0
D1
D2
D3
D4
D7
Timepoint
Placebo
Vaccine

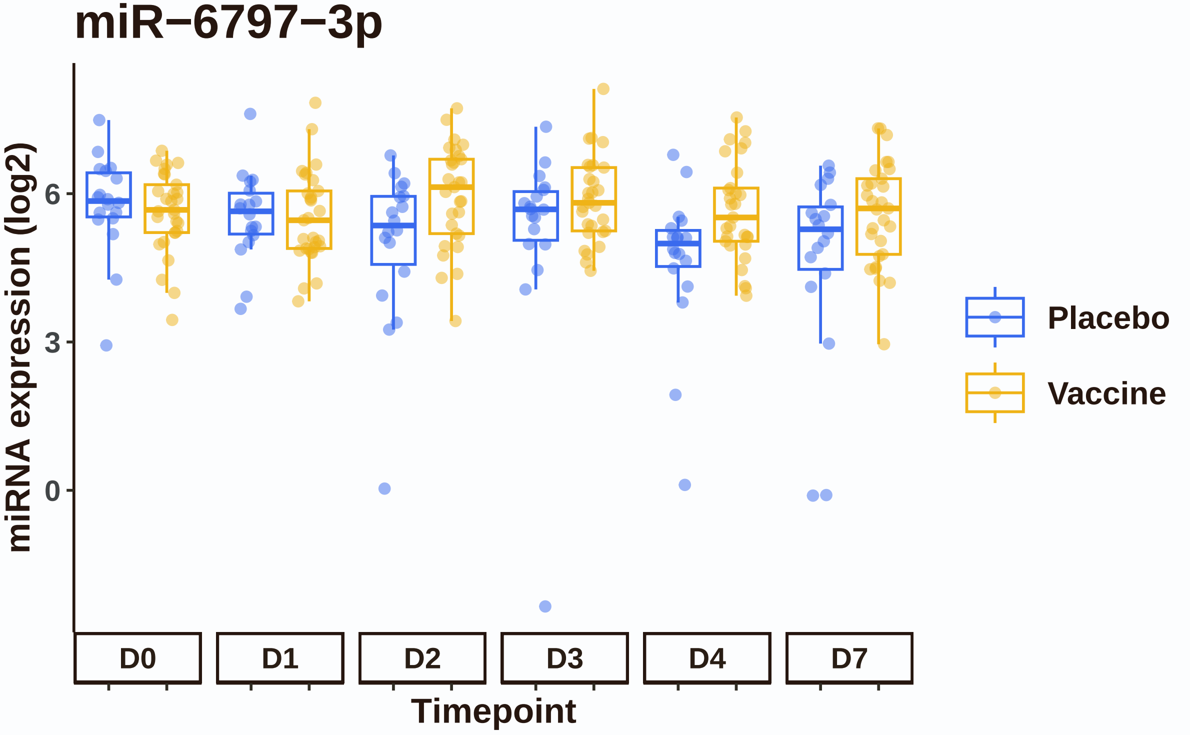
miR−6797−3p
miRNA expression (log2)
6
3
0
D0
D1
D2
D3
D4
D7
Timepoint
Placebo
Vaccine

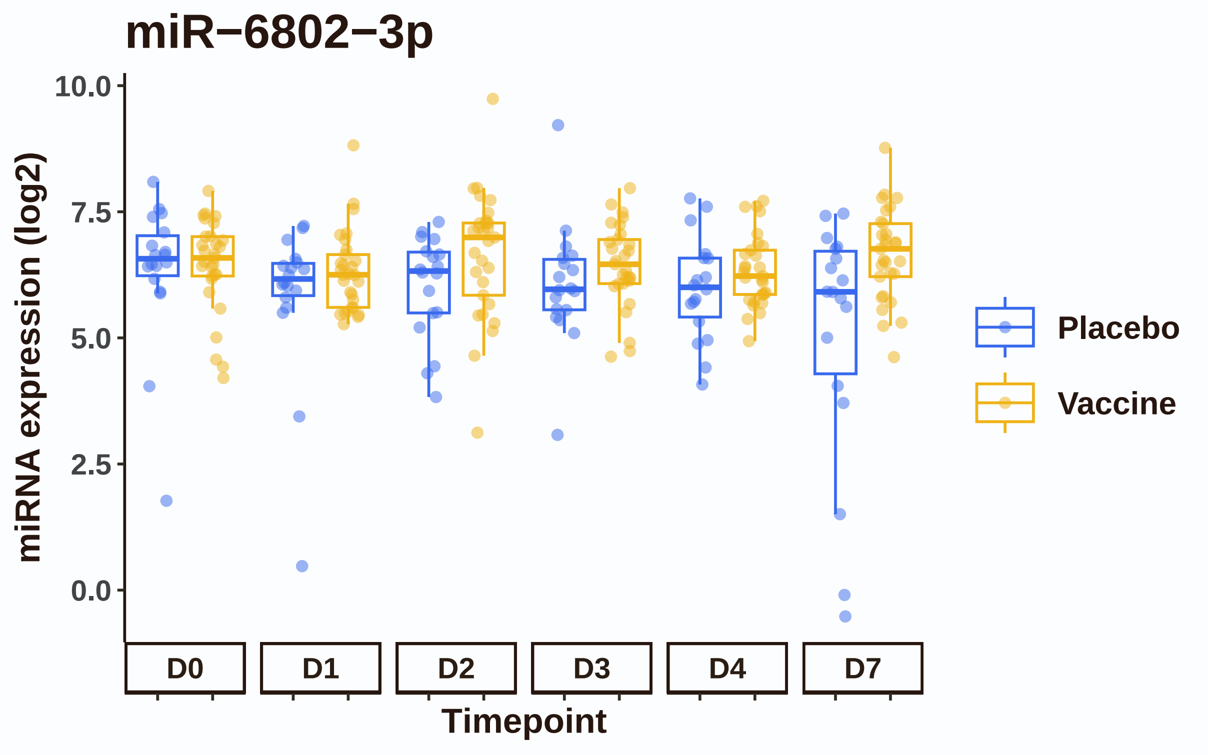

miR−6802−3p
10.0
7.5
5.0
2.5
0.0
miRNA expression (log2)
D0
D1
D2
D3
D4
D7
Timepoint
Placebo
Vaccine

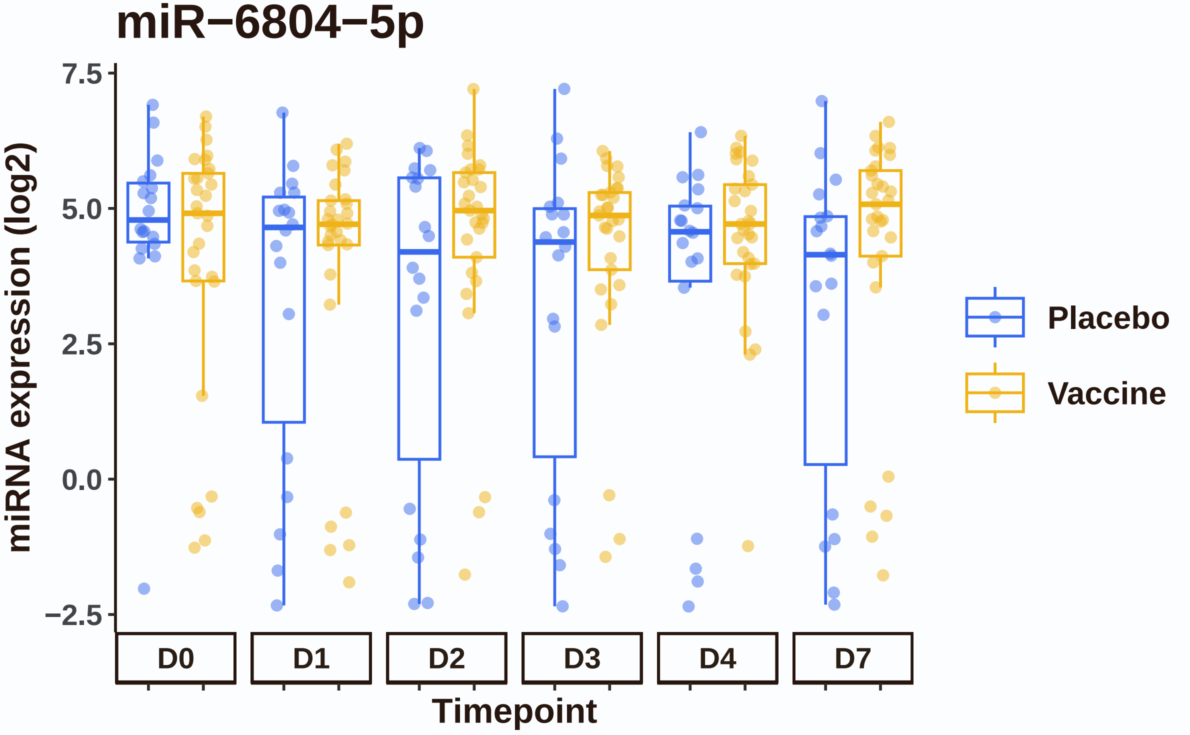
miR−6804−5p
miRNA expression (log2)
7.5
5.0
2.5
0.0
−2.5
D0
D1
D2
D3
D4
D7
Timepoint
Placebo
Vaccine

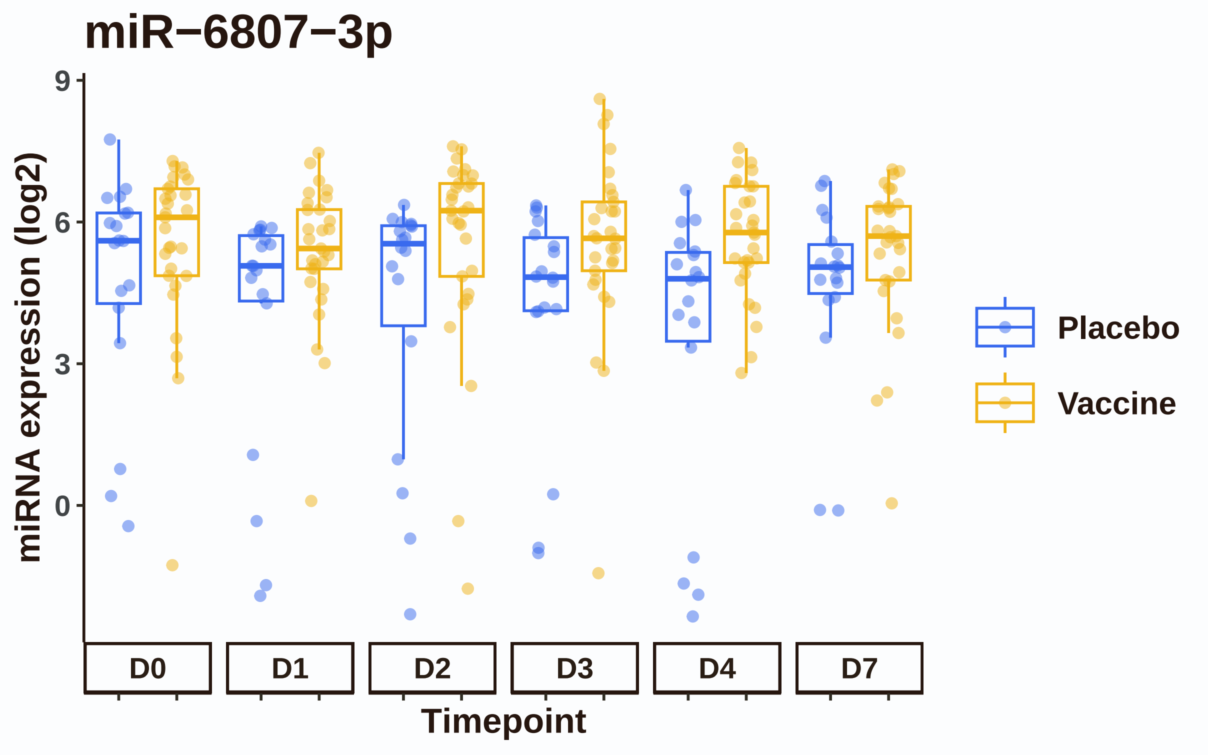

miR−6807−3p
miRNA expression (log2)
9
6
3
0
D0
D1
D2
D3
D4
D7
Timepoint
Placebo
Vaccine

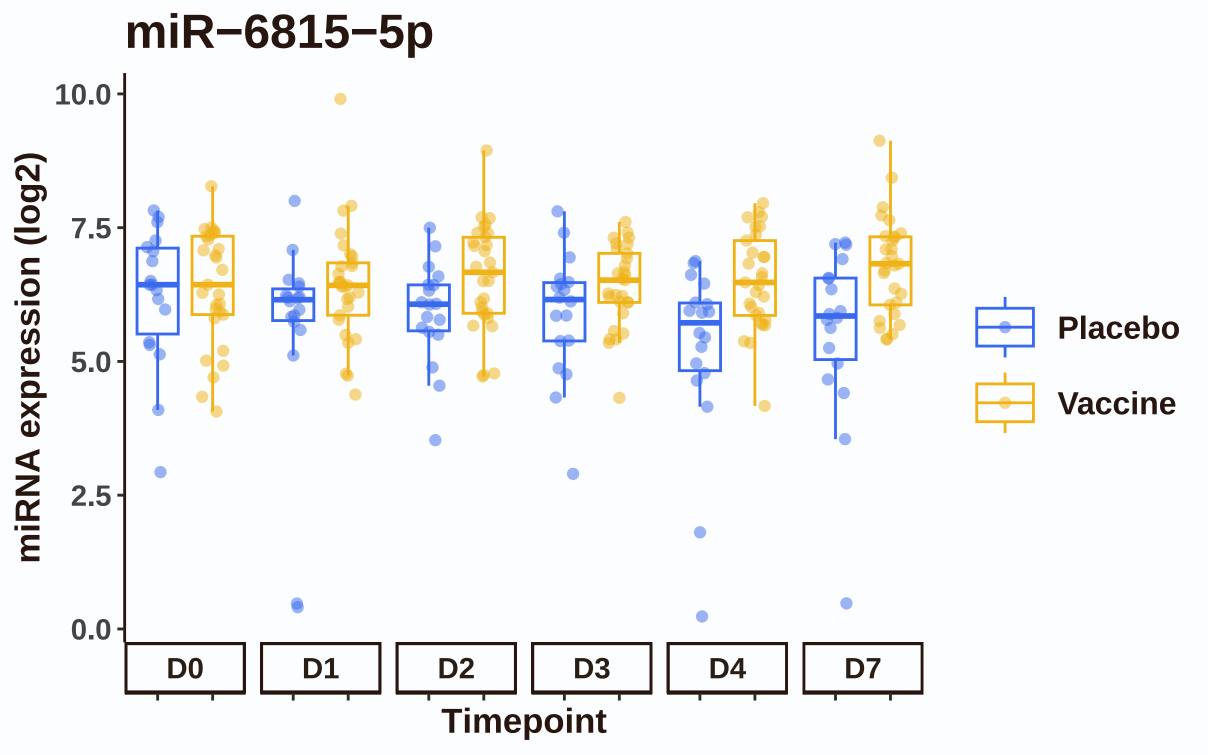
miR−6815−5p
miRNA expression (log2)
10.0
7.5
5.0
2.5
0.0
D0
D1
D2
D3
D4
D7
Timepoint
Placebo
Vaccine

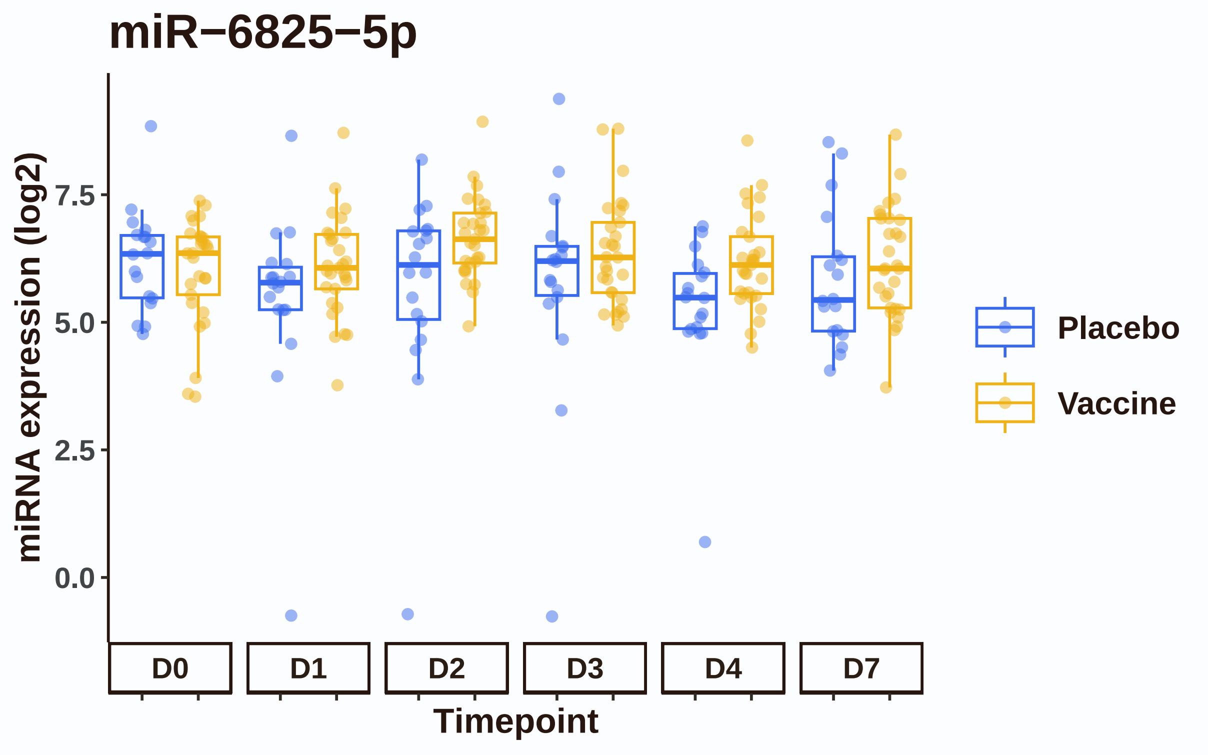

miR−6825−5p
miRNA expression (log2)
7.5
5.0
2.5
0.0
D0
D1
D2
D3
D4
D7
Timepoint
Placebo
Vaccine

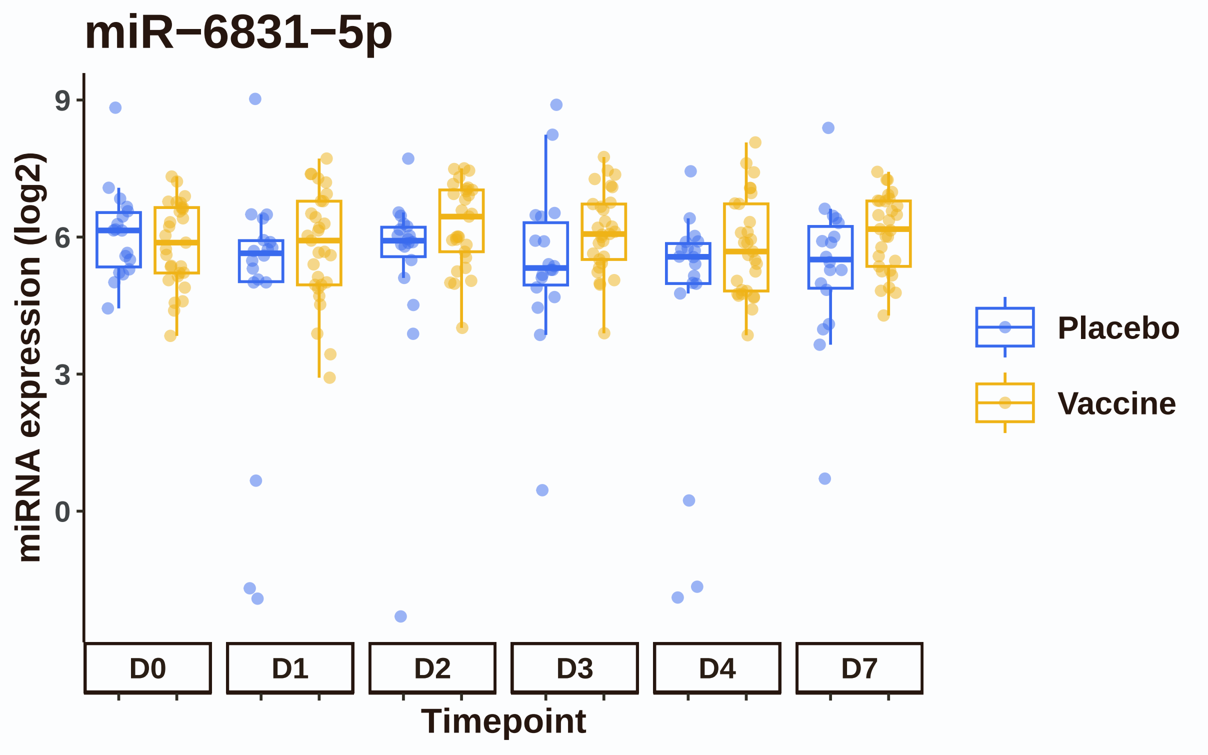

miR−6831−5p
miRNA expression (log2)
9
6
3
0
D0
D1
D2
D3
D4
D7
Timepoint
Placebo
Vaccine

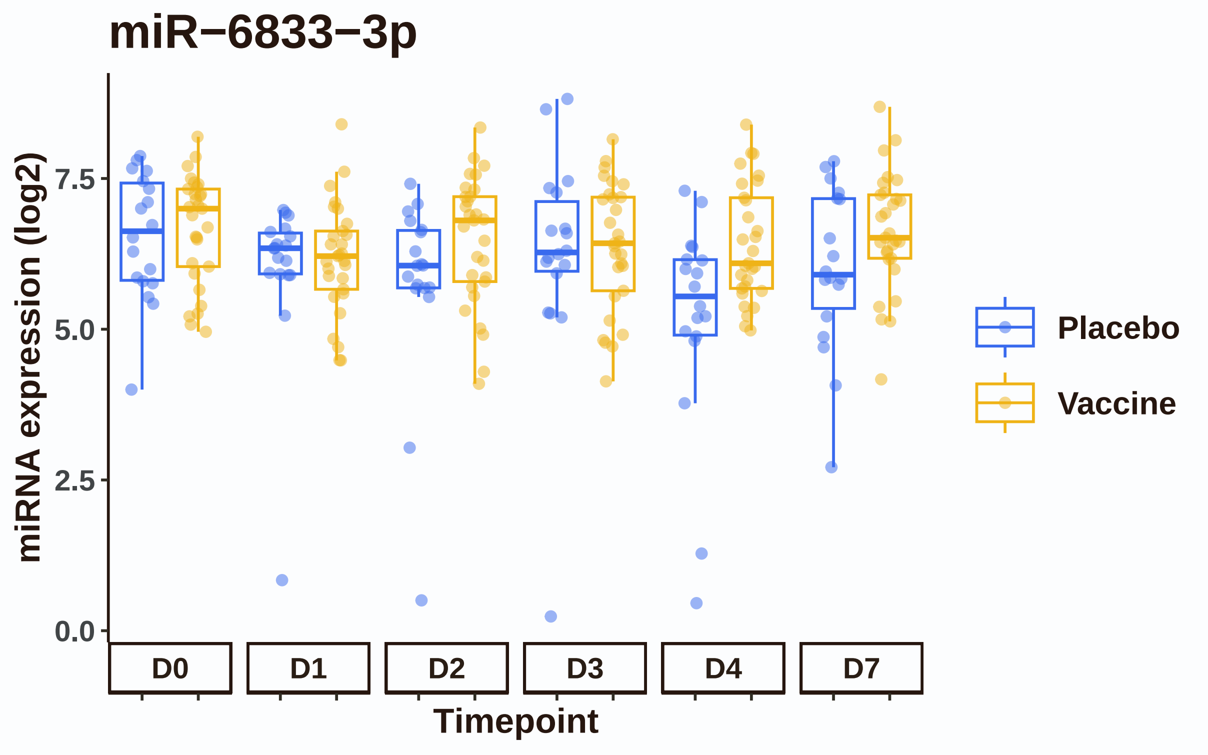
miR−6833−3p
miRNA expression (log2)
0.0
2.5
5.0
7.5
D0
D1
D2
D3
D4
D7
Timepoint
Placebo
Vaccine

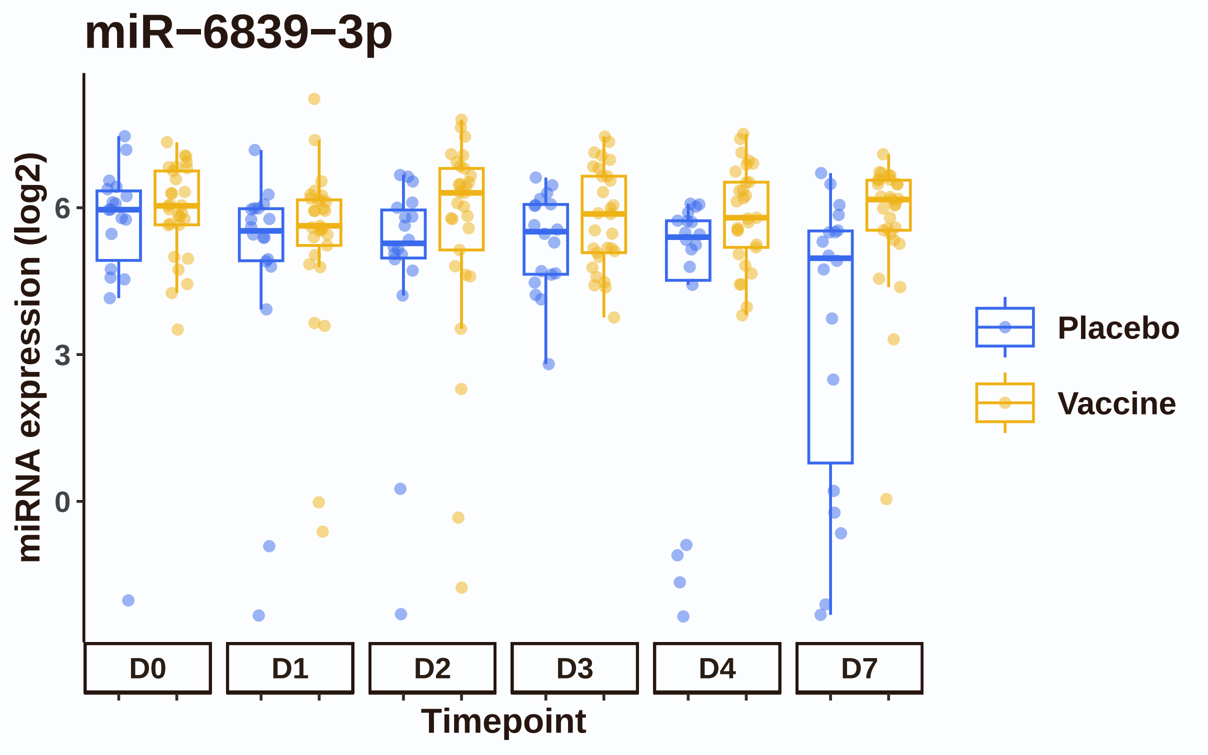
miR−6839−3p
miRNA expression (log2)
6
3
0
D0
D1
D2
D3
D4
D7
Timepoint
Placebo
Vaccine

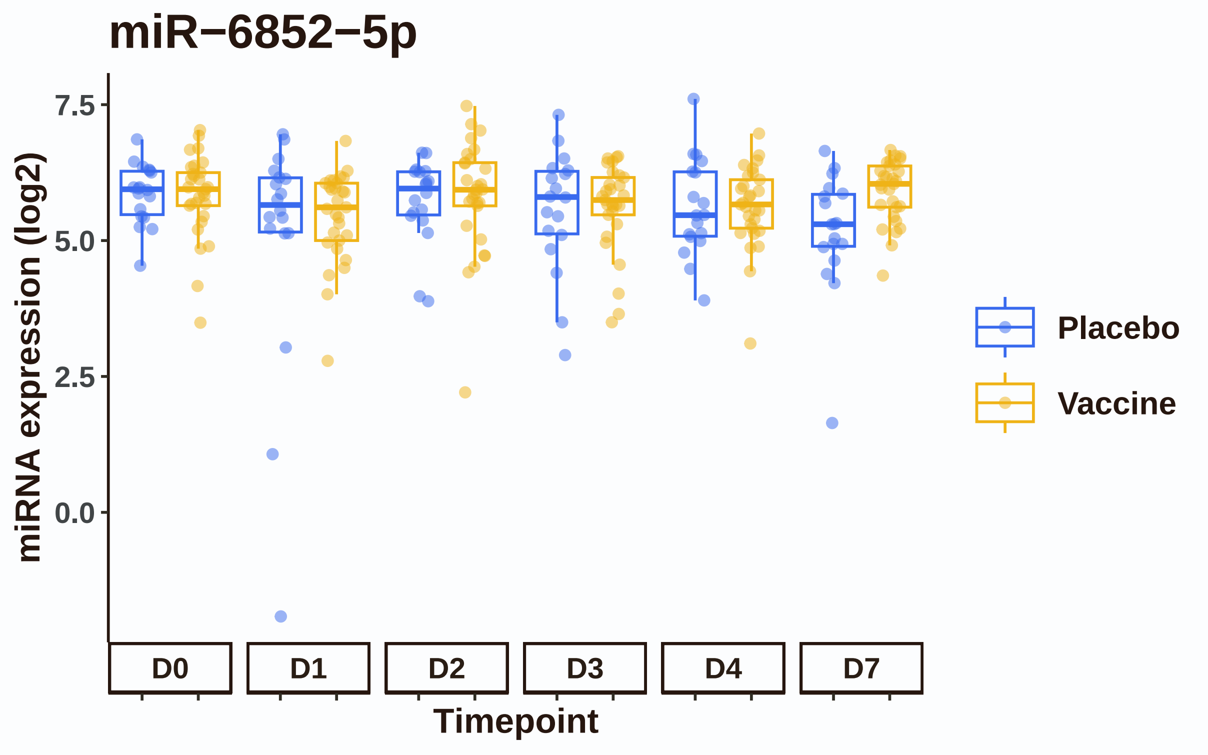
miR−6852−5p
miRNA expression (log2)
7.5
5.0
2.5
0.0
D0
D1
D2
D3
D4
D7
Timepoint
Placebo
Vaccine

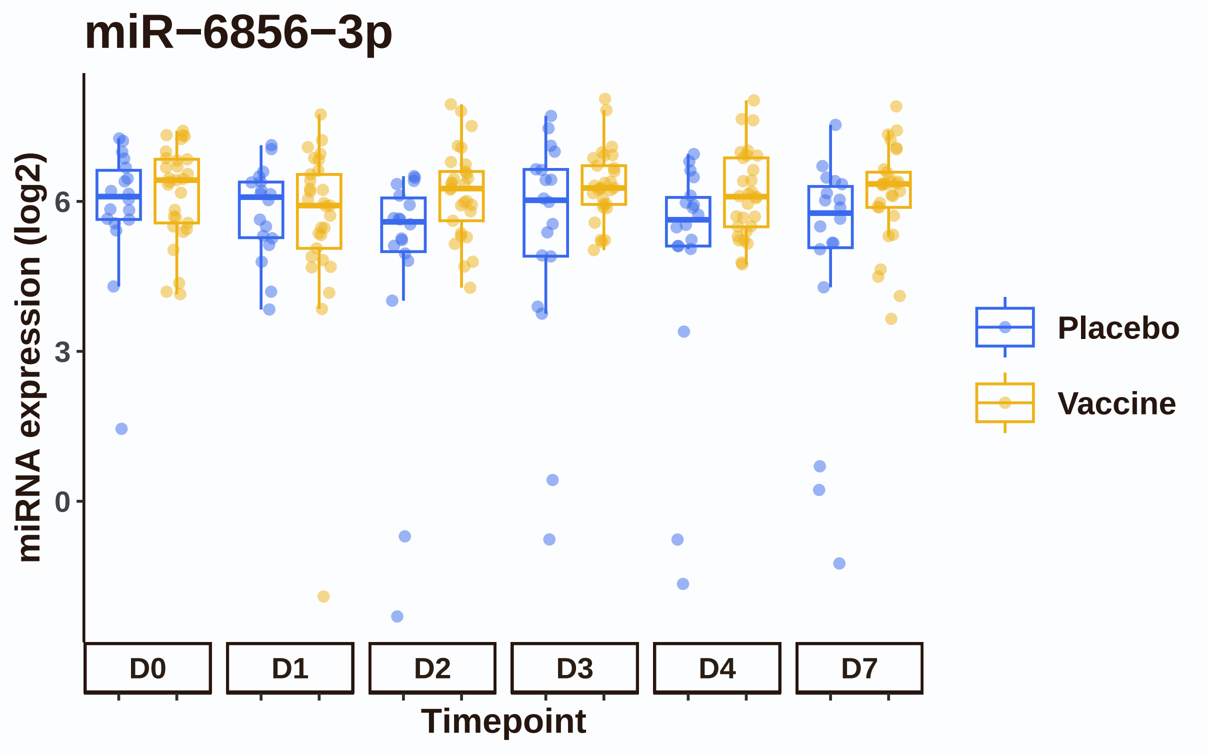
miR−6856−3p
miRNA expression (log2)
6
3
0
D0
D1
D2
D3
D4
D7
Timepoint
Placebo
Vaccine

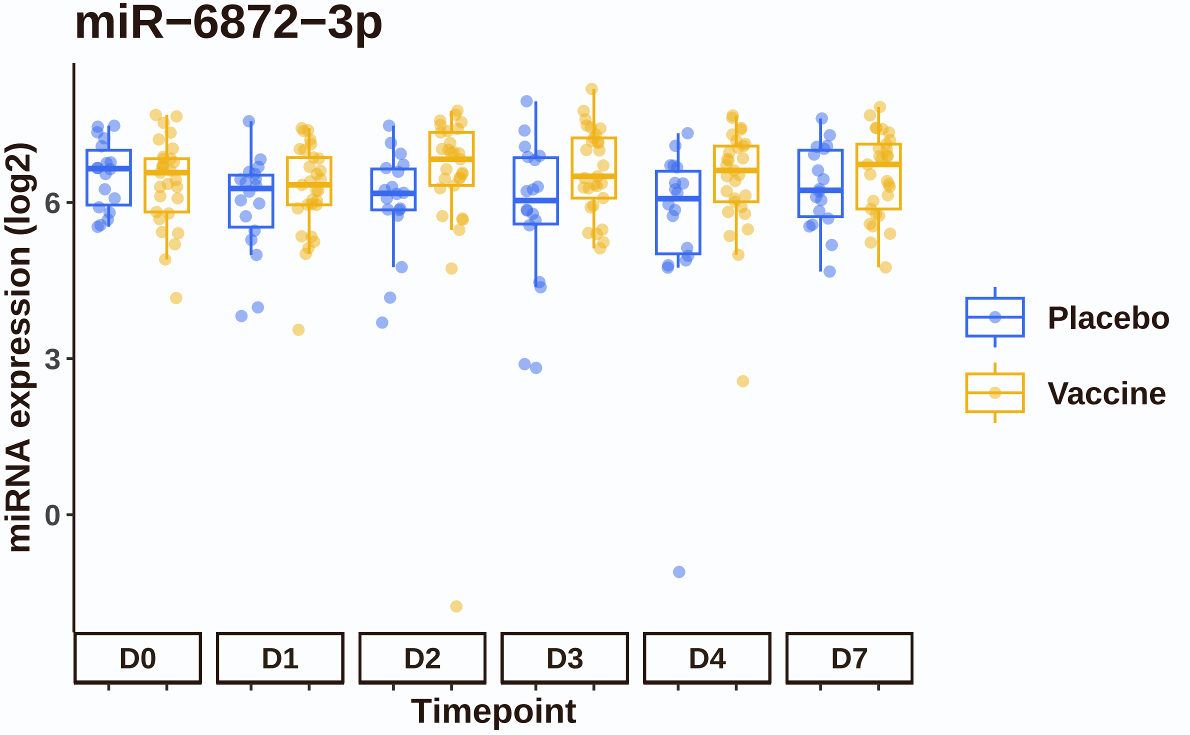

miR−6872−3p
miRNA expression (log2)
6
3
0
D0
D1
D2
D3
D4
D7
Timepoint
Placebo
Vaccine

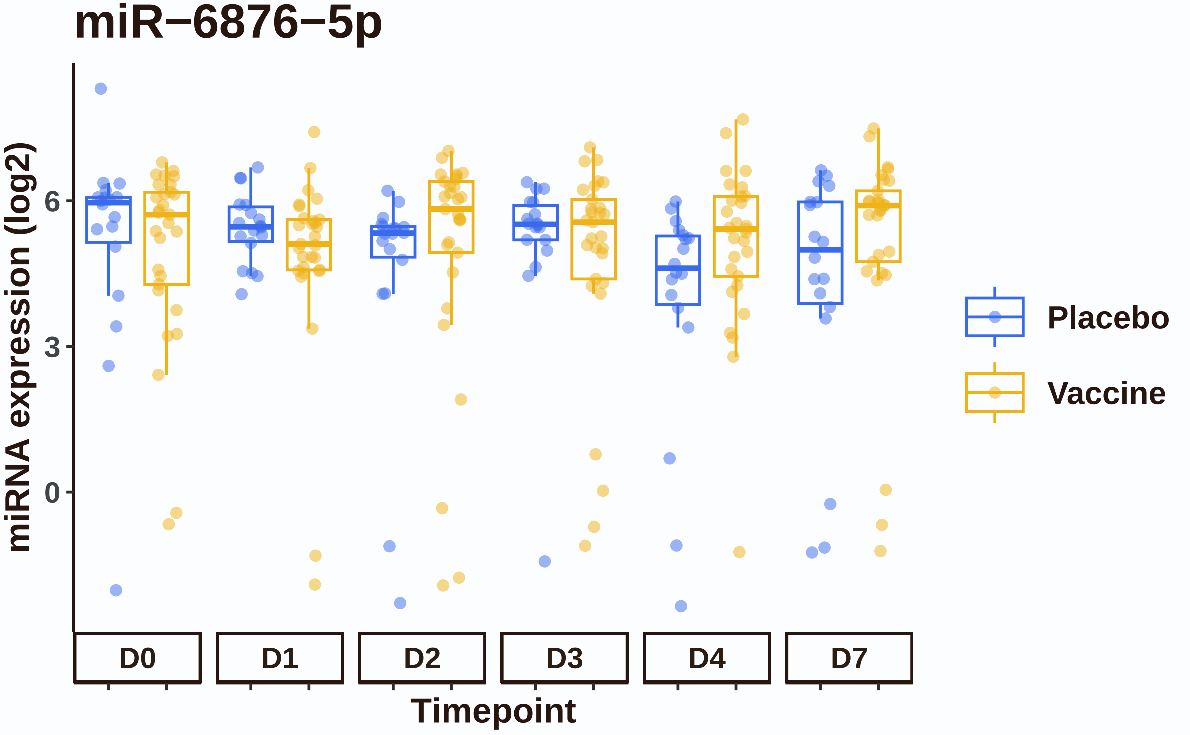

miR−6876−5p
miRNA expression (log2)
6
3
0
D0
D1
D2
D3
D4
D7
Timepoint
Placebo
Vaccine

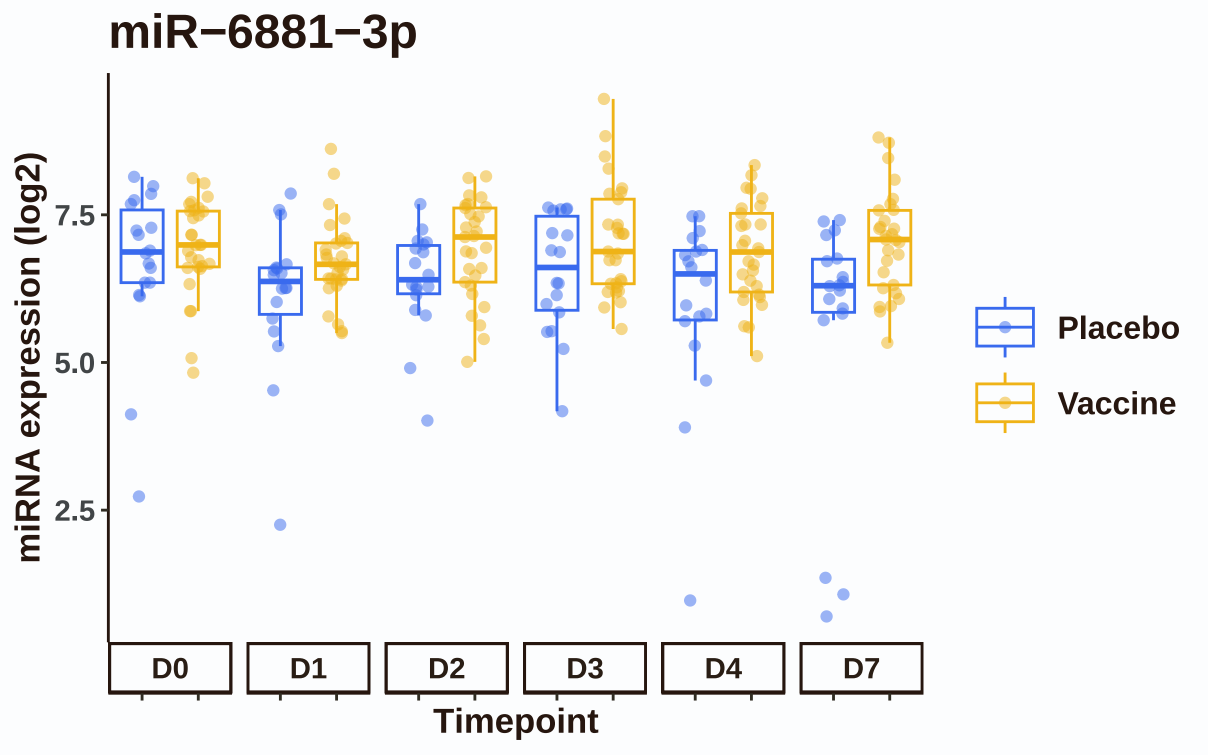
miR−6881−3p
miRNA expression (log2)
7.5
5.0
2.5
D0
D1
D2
D3
D4
D7
Timepoint
Placebo
Vaccine

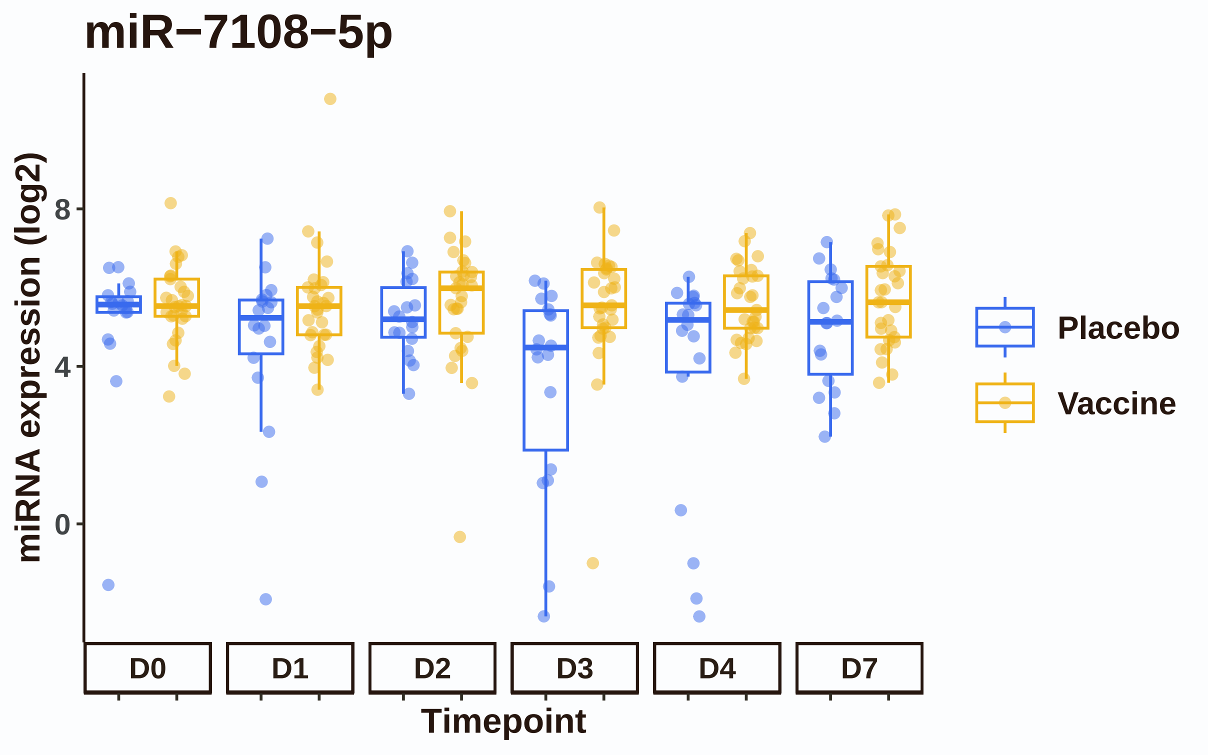
miR−7108−5p
miRNA expression (log2)
8
4
0
D0
D1
D2
D3
D4
D7
Timepoint
Placebo
Vaccine

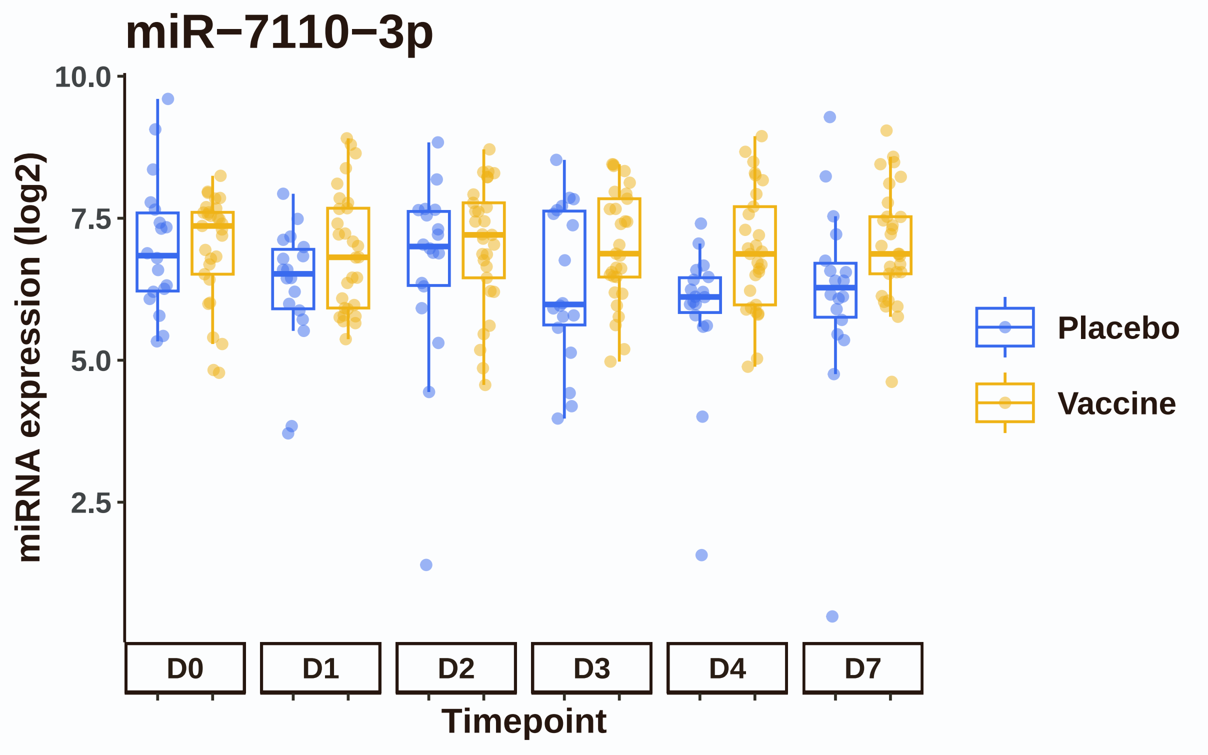

miR−7110−3p
miRNA expression (log2)
10.0
7.5
5.0
2.5
D0
D1
D2
D3
D4
D7
Timepoint
Placebo
Vaccine

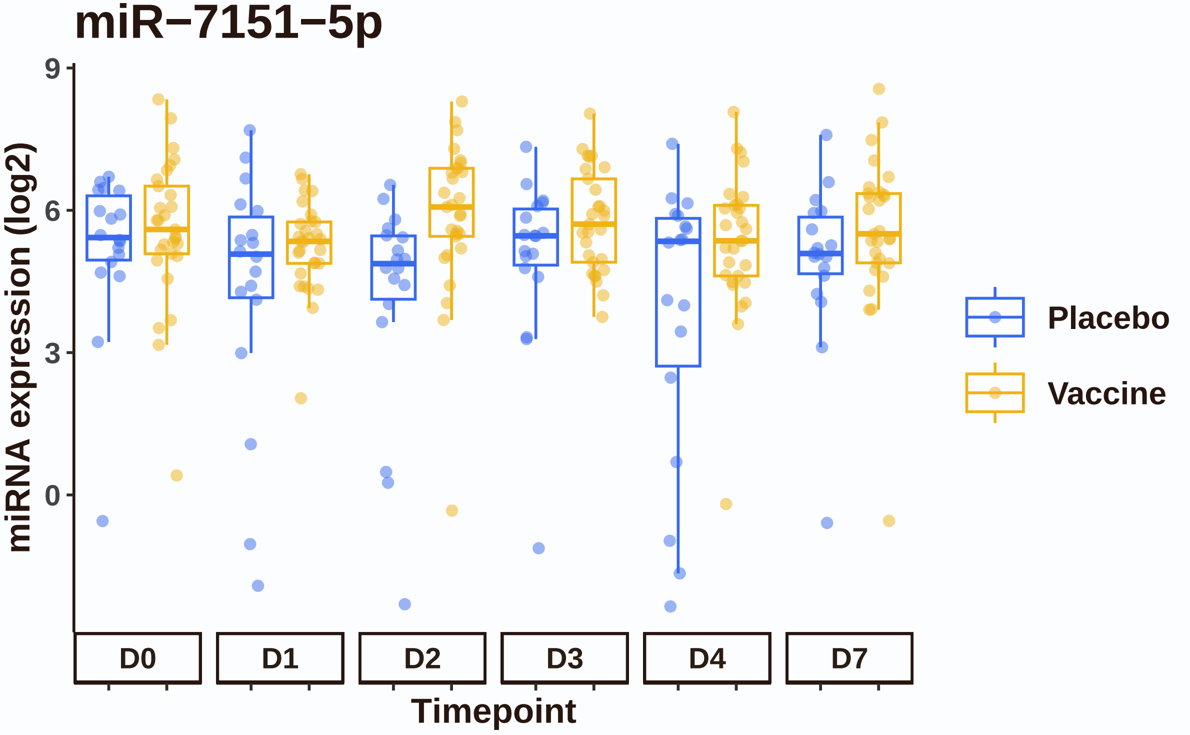

miR−7151−5p
miRNA expression (log2)
9
6
3
0
D0
D1
D2
D3
D4
D7
Timepoint
Placebo
Vaccine

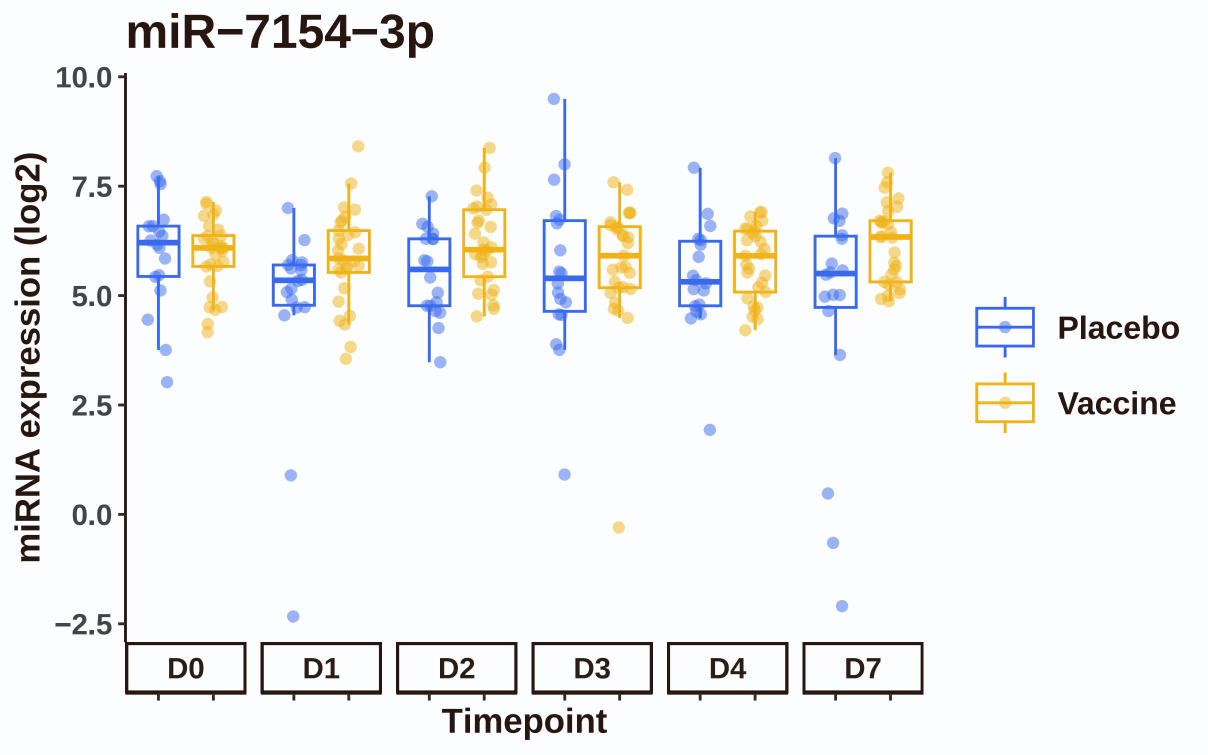
miR−7154−3p
miRNA expression (log2)
10.0
7.5
5.0
2.5
0.0
−2.5
D0
D1
D2
D3
D4
D7
Timepoint
Placebo
Vaccine

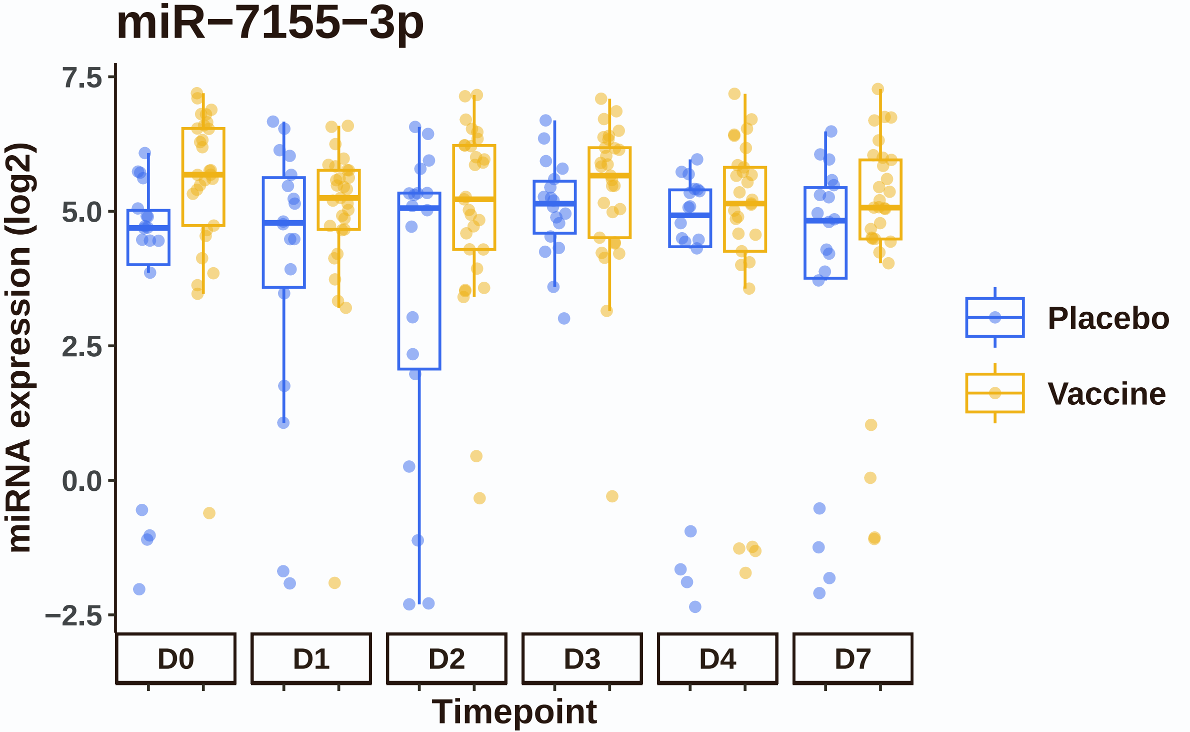
miR−7155−3p
miRNA expression (log2)
7.5
5.0
2.5
0.0
−2.5
D0
D1
D2
D3
D4
D7
Timepoint
Placebo
Vaccine

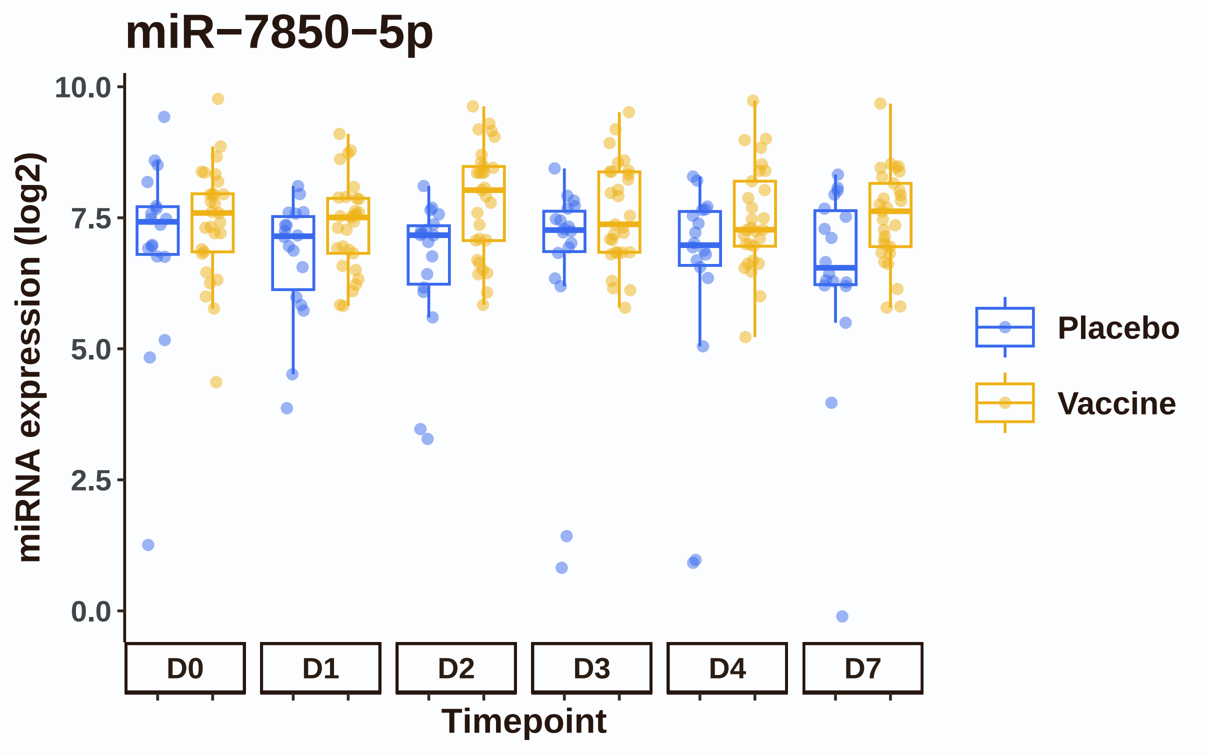

miR−7850−5p
miRNA expression (log2)
10.0
7.5
5.0
2.5
0.0
D0
D1
D2
D3
D4
D7
Timepoint
Placebo
Vaccine

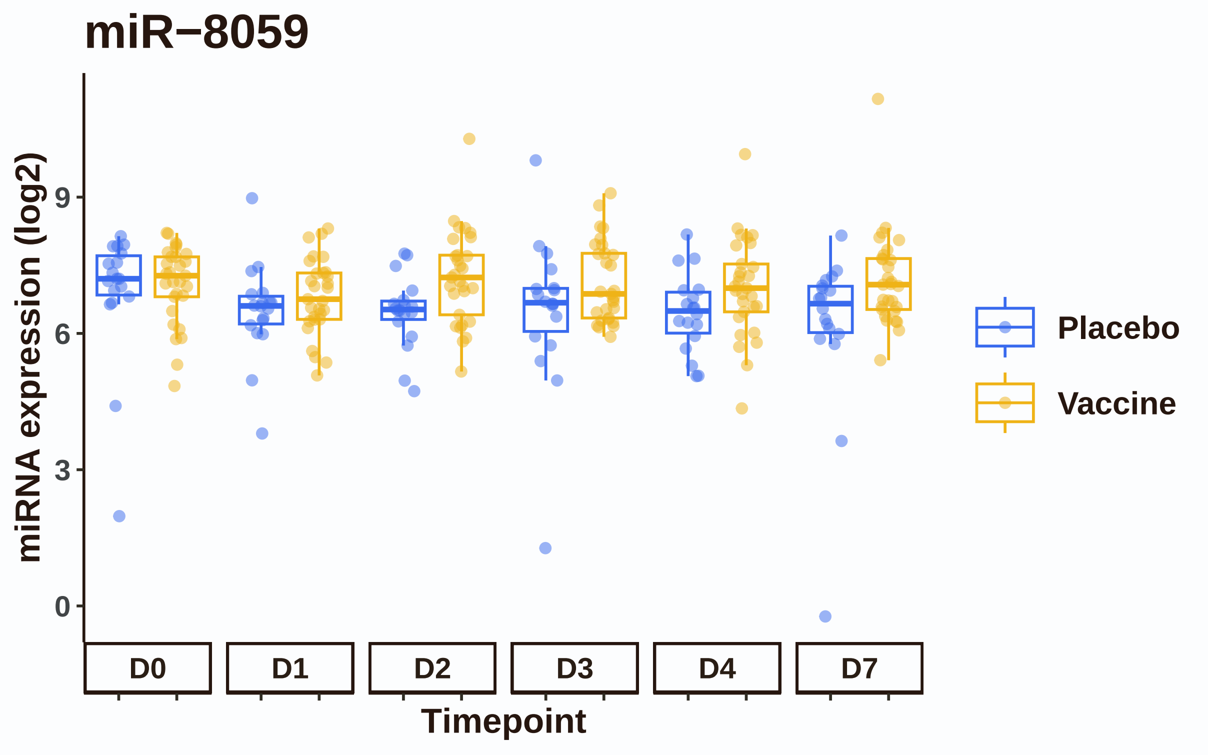
miR−8059
miRNA expression (log2)
0
3
6
9
D0
D1
D2
D3
D4
D7
Timepoint
Placebo
Vaccine

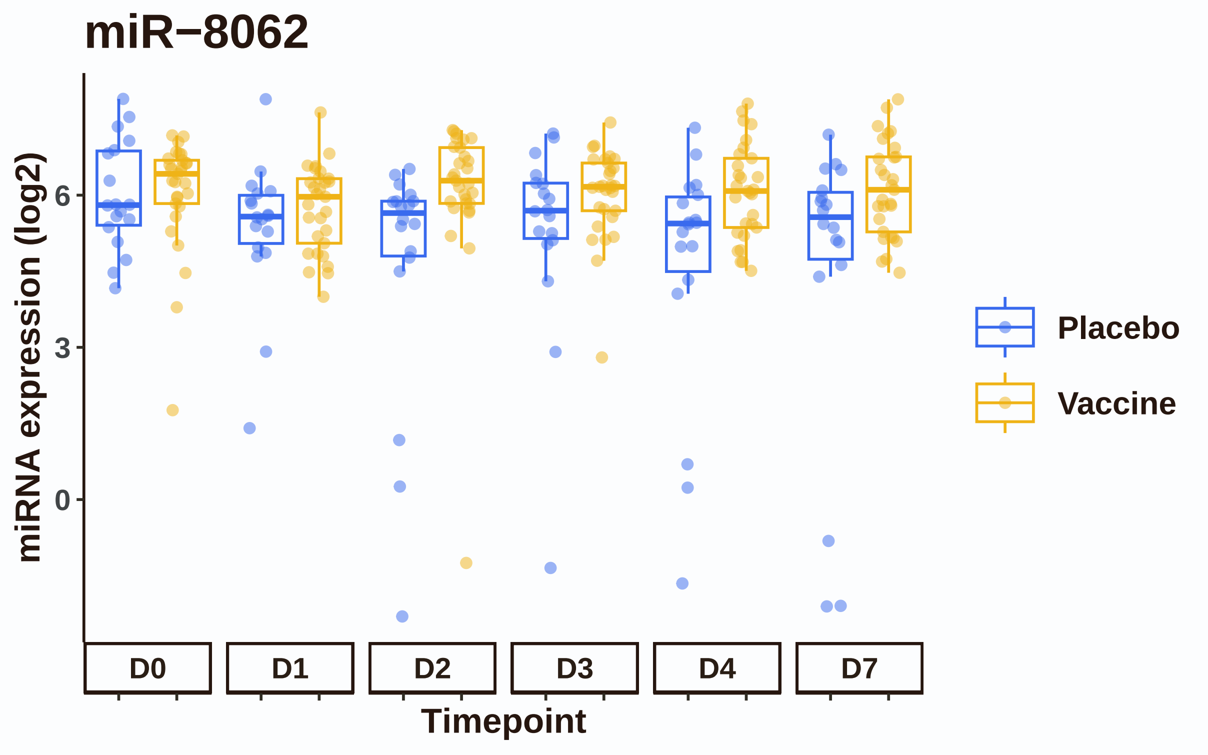
miR−8062
miRNA expression (log2)
6
3
0
D0
D1
D2
D3
D4
D7
Timepoint
Placebo
Vaccine

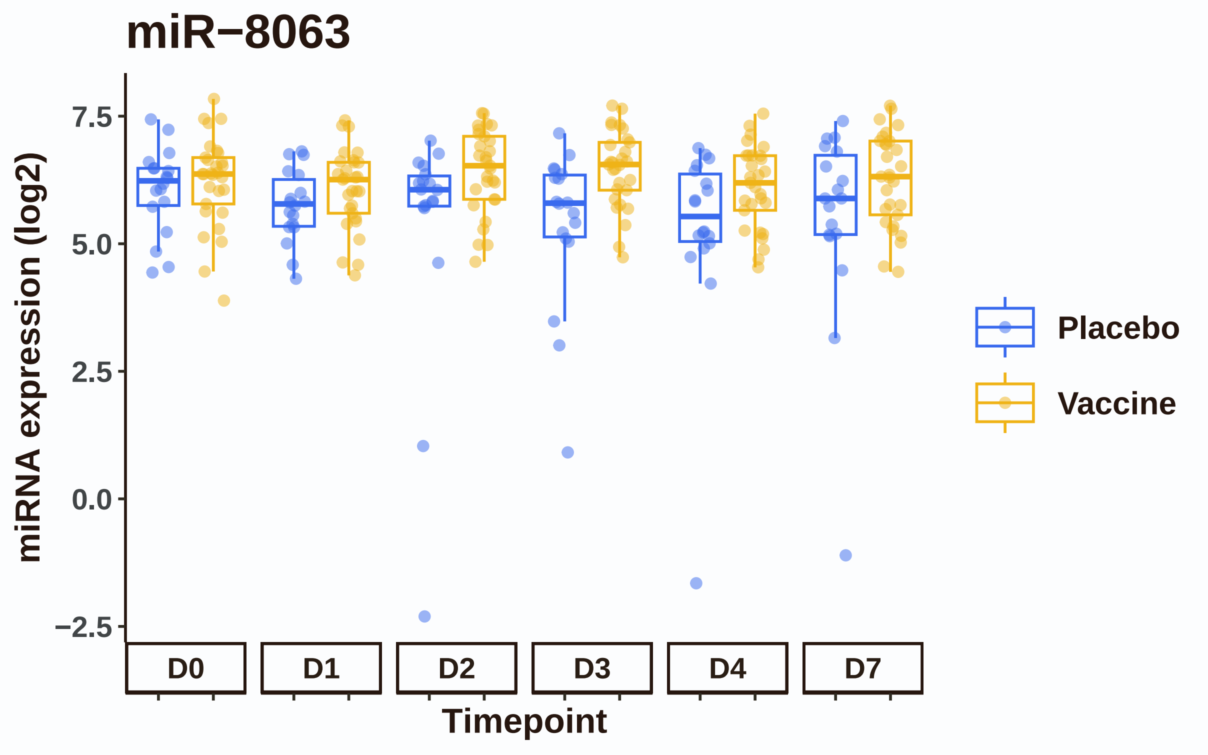
miR−8063
miRNA expression (log2)
7.5
5.0
2.5
0.0
−2.5
D0
D1
D2
D3
D4
D7
Timepoint
Placebo
Vaccine

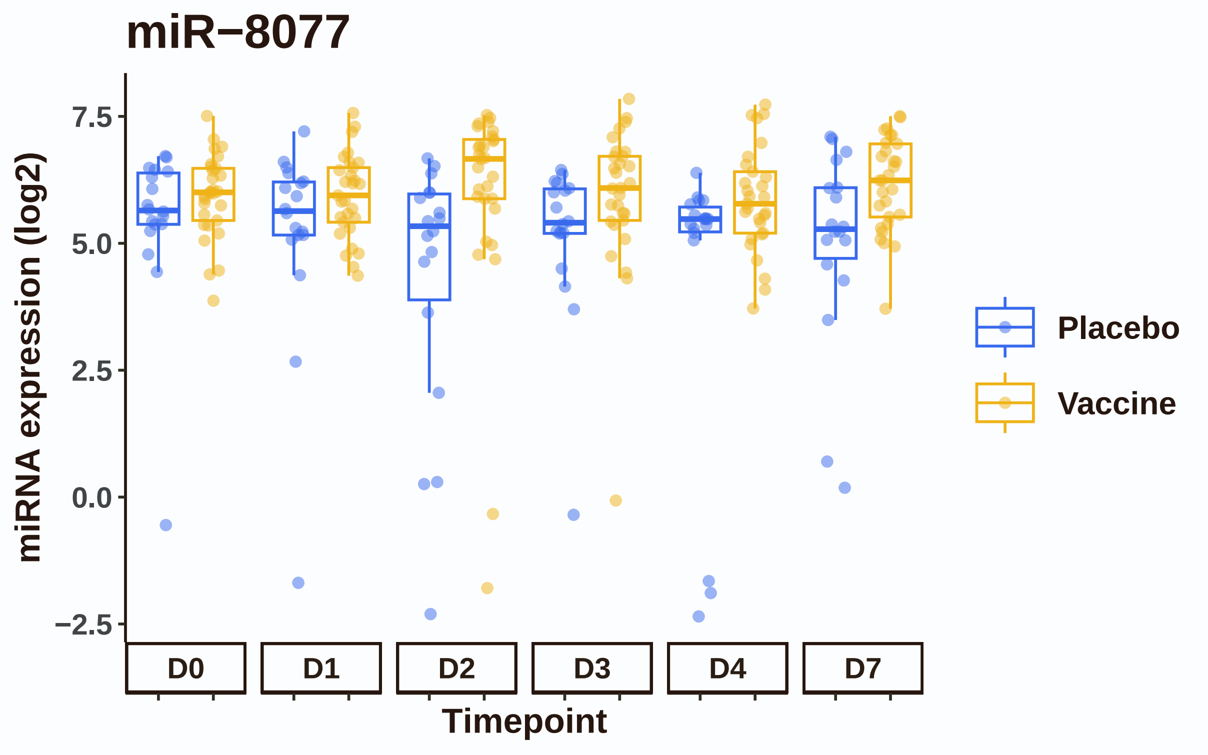
miR−8077
miRNA expression (log2)
7.5
5.0
2.5
0.0
−2.5
D0
D1
D2
D3
D4
D7
Timepoint
Placebo
Vaccine

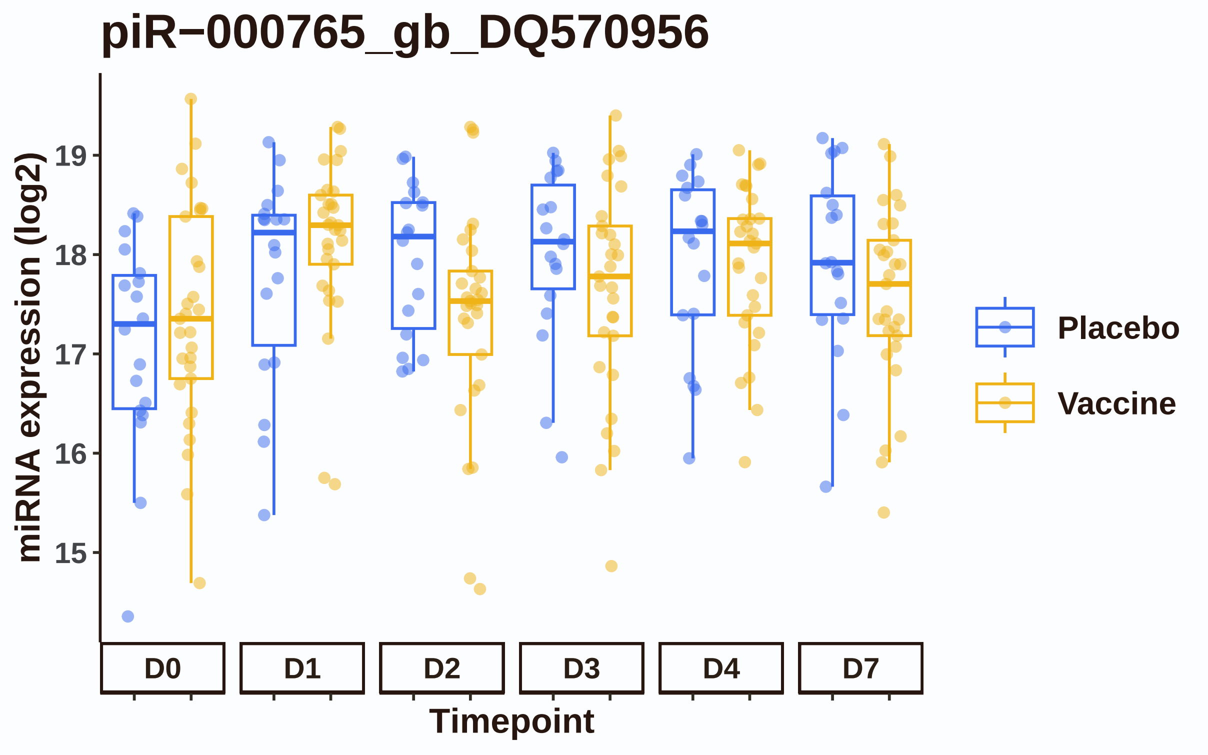

piR−000765_gb_DQ570956
miRNA expression (log2)
19
18
17
16
15
D0
D1
D2
D3
D4
D7
Timepoint
Placebo
Vaccine

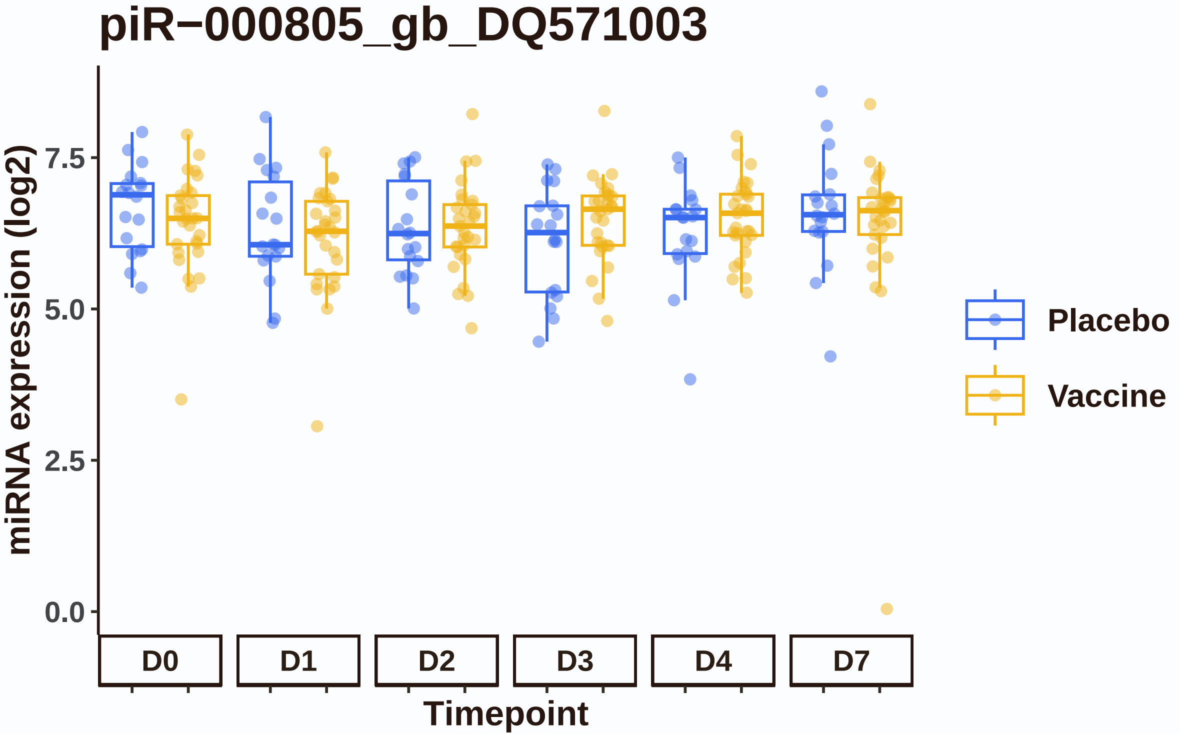
piR−000805_gb_DQ571003
miRNA expression (log2)
0.0
2.5
5.0
7.5
D0
D1
D2
D3
D4
D7
Timepoint
Placebo
Vaccine

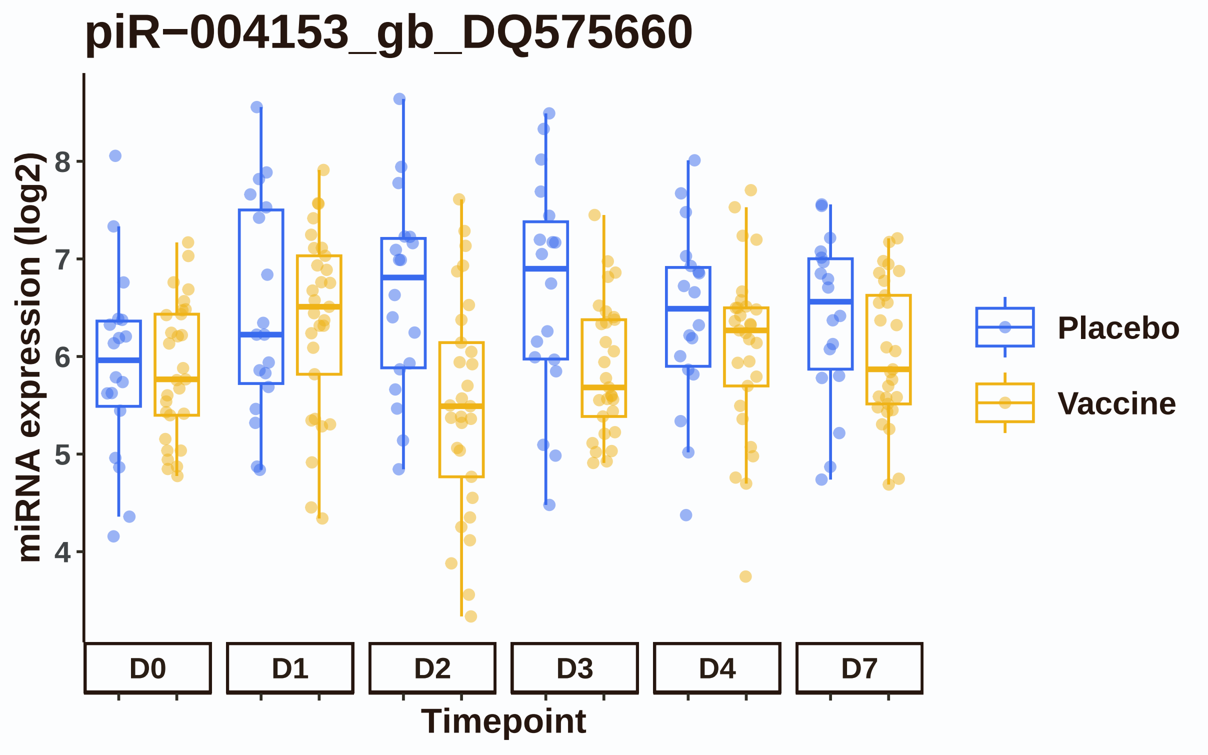

piR−004153_gb_DQ575660
miRNA expression (log2)
4
5
6
7
8
D0
D1
D2
D3
D4
D7
Timepoint
Placebo
Vaccine

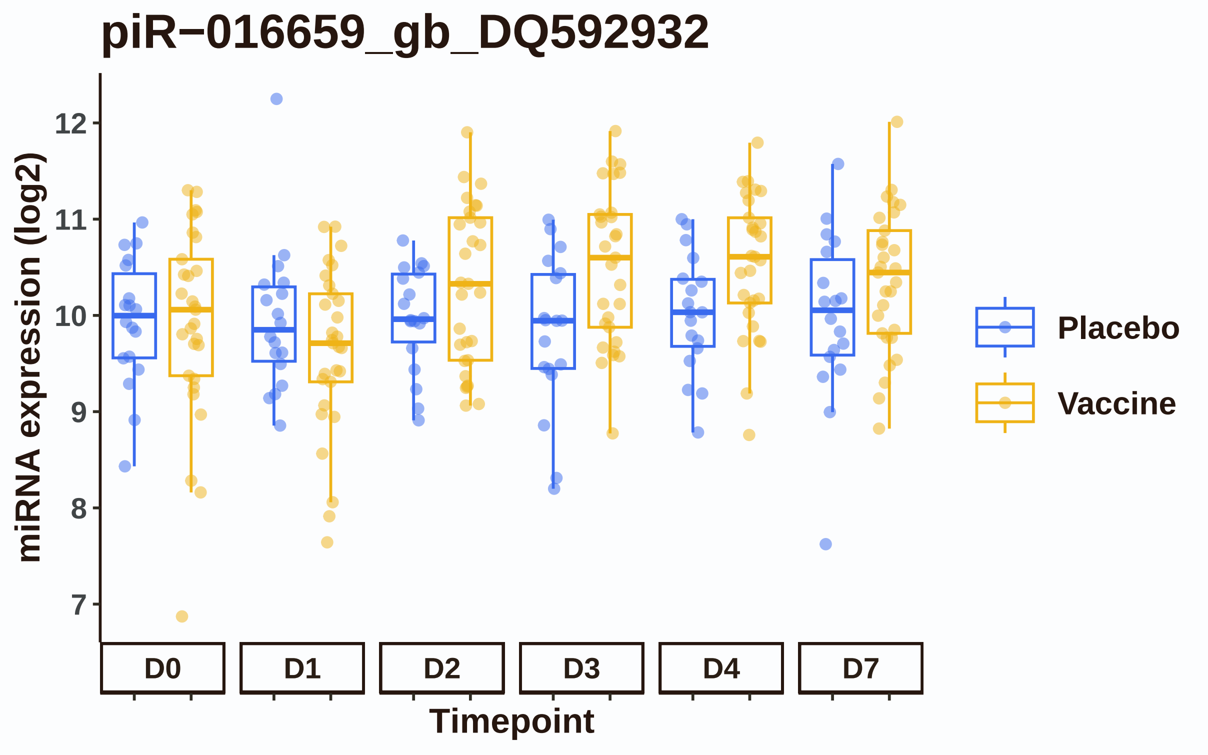

piR−016659_gb_DQ592932
miRNA expression (log2)
12
11
10
9
8
7
D0
D1
D2
D3
D4
D7
Timepoint
Placebo
Vaccine

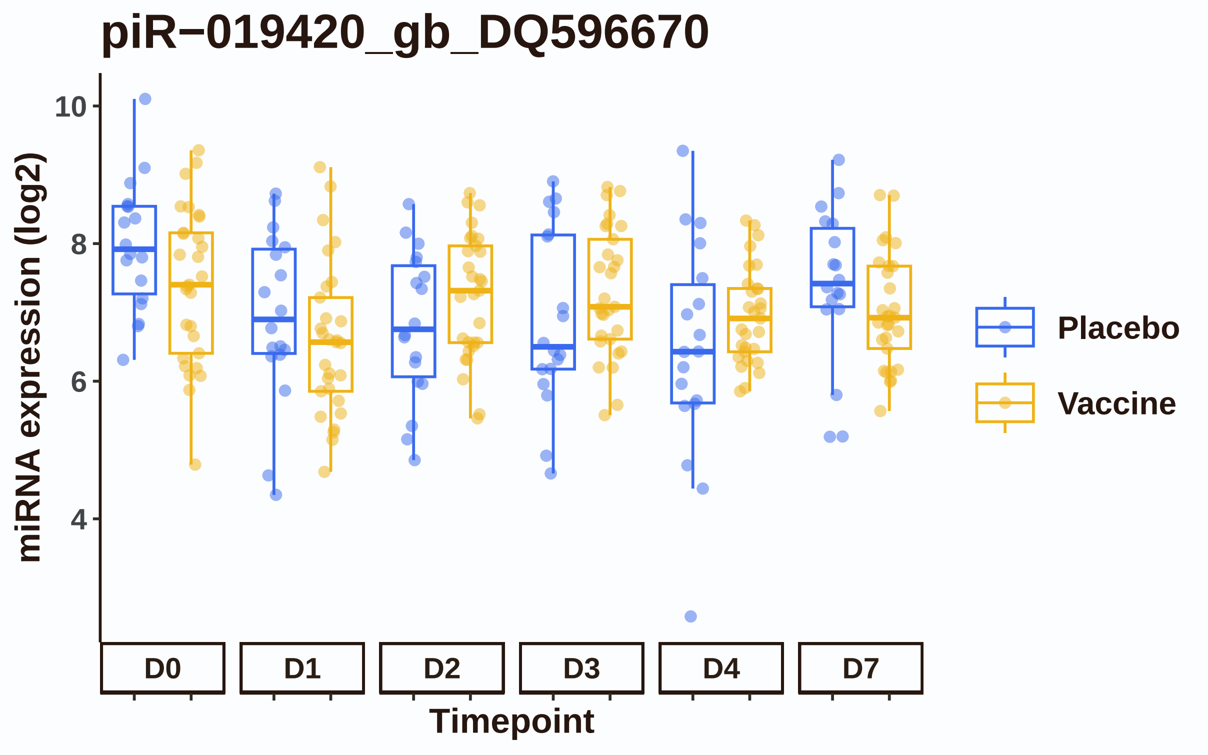

piR−019420_gb_DQ596670
miRNA expression (log2)
10
8
6
4
D0
D1
D2
D3
D4
D7
Timepoint
Placebo
Vaccine

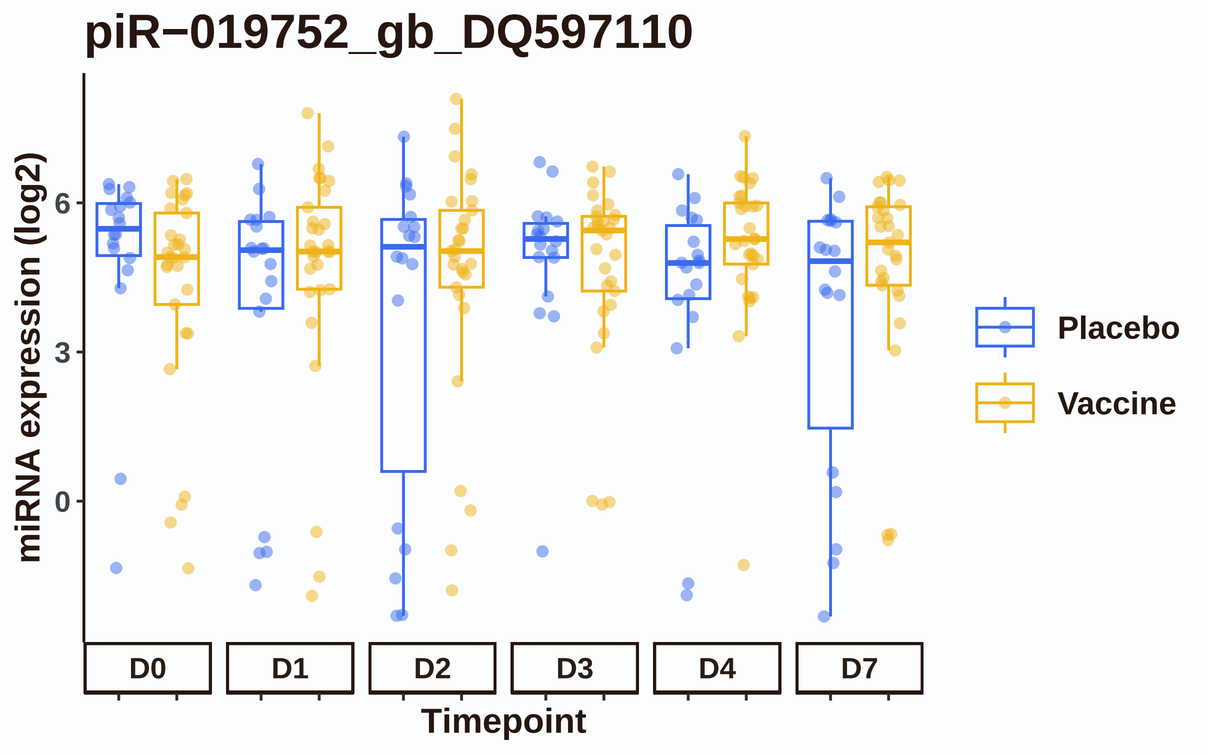

piR−019752_gb_DQ597110
miRNA expression (log2)
6
3
0
D0
D1
D2
D3
D4
D7
Timepoint
Placebo
Vaccine

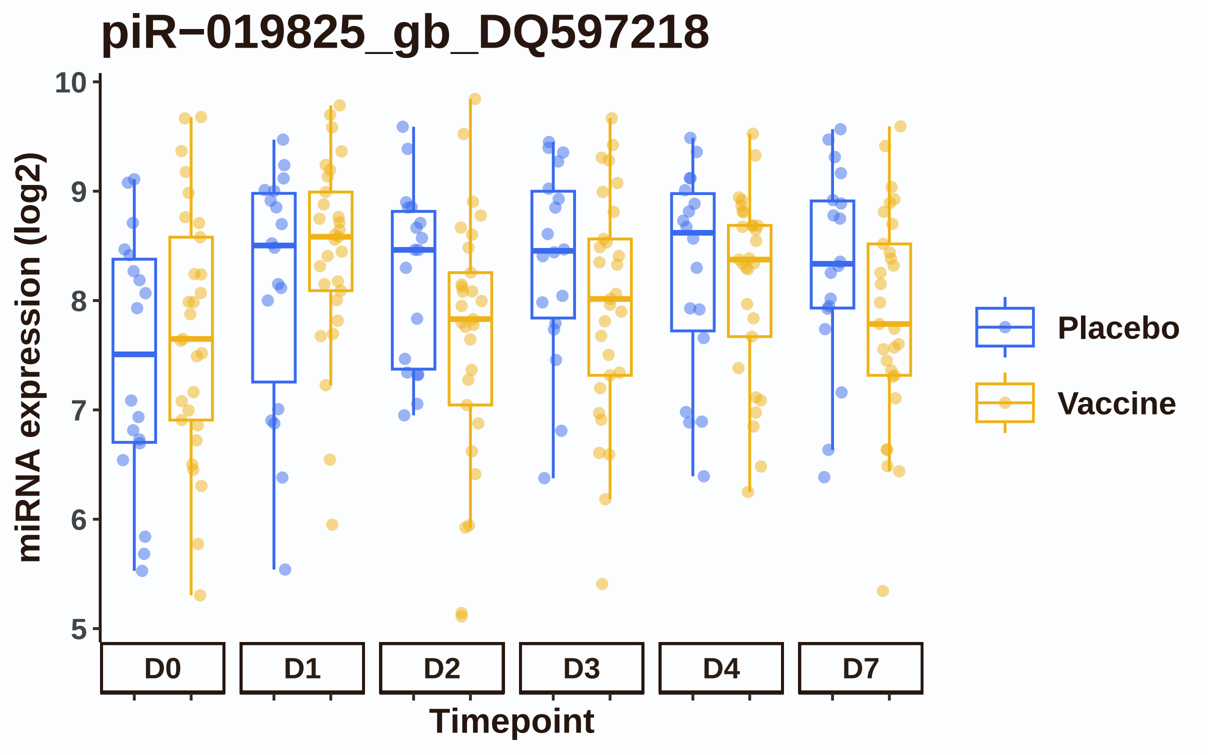
piR−019825_gb_DQ597218
10
9
8
7
6
5
miRNA expression (log2)
D0
D1
D2
D3
D4
D7
Timepoint
Placebo
Vaccine

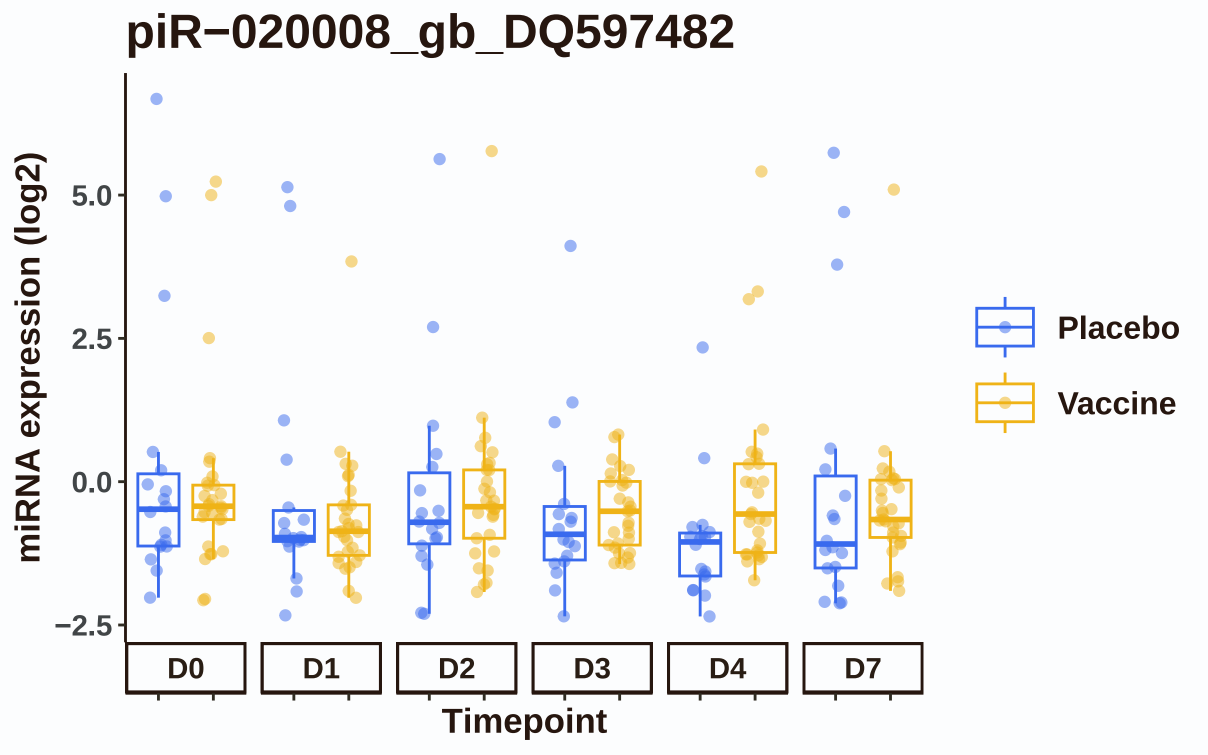

piR−020008_gb_DQ597482
miRNA expression (log2)
5.0
2.5
0.0
−2.5
D0
D1
D2
D3
D4
D7
Timepoint
Placebo
Vaccine

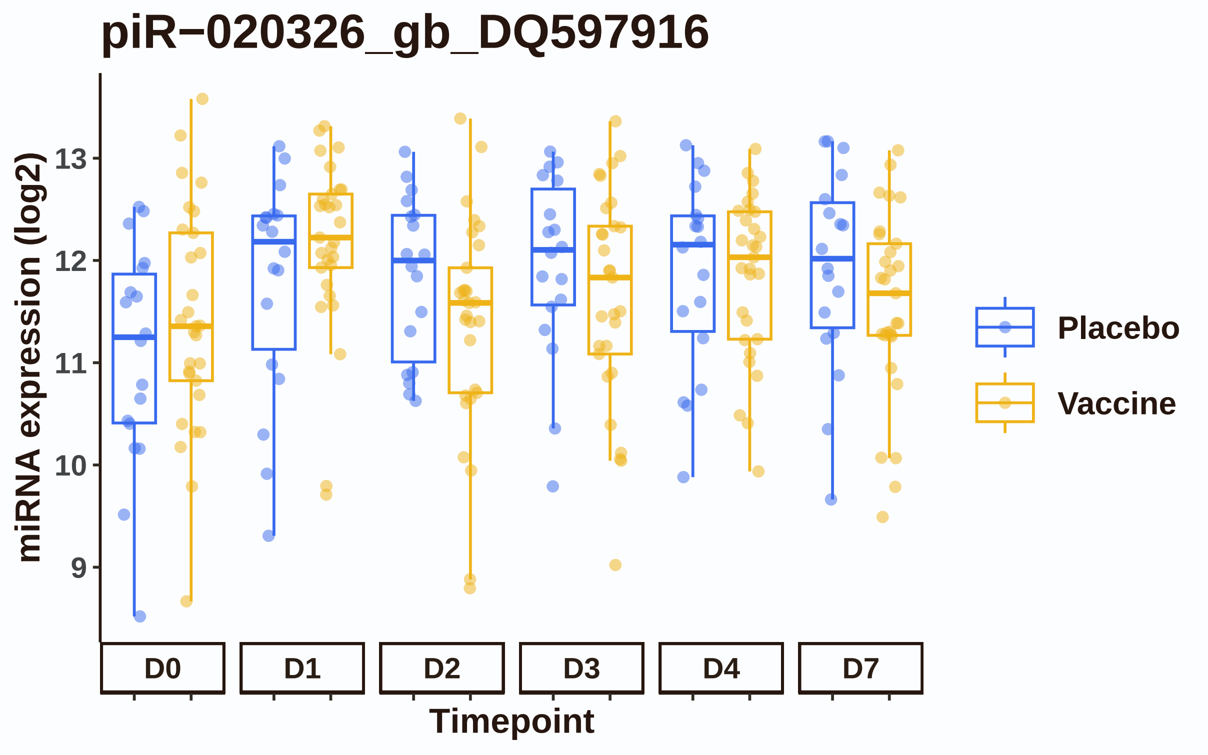
piR−020326_gb_DQ597916
miRNA expression (log2)
13
12
11
10
9
D0
D1
D2
D3
D4
D7
Timepoint
Placebo
Vaccine

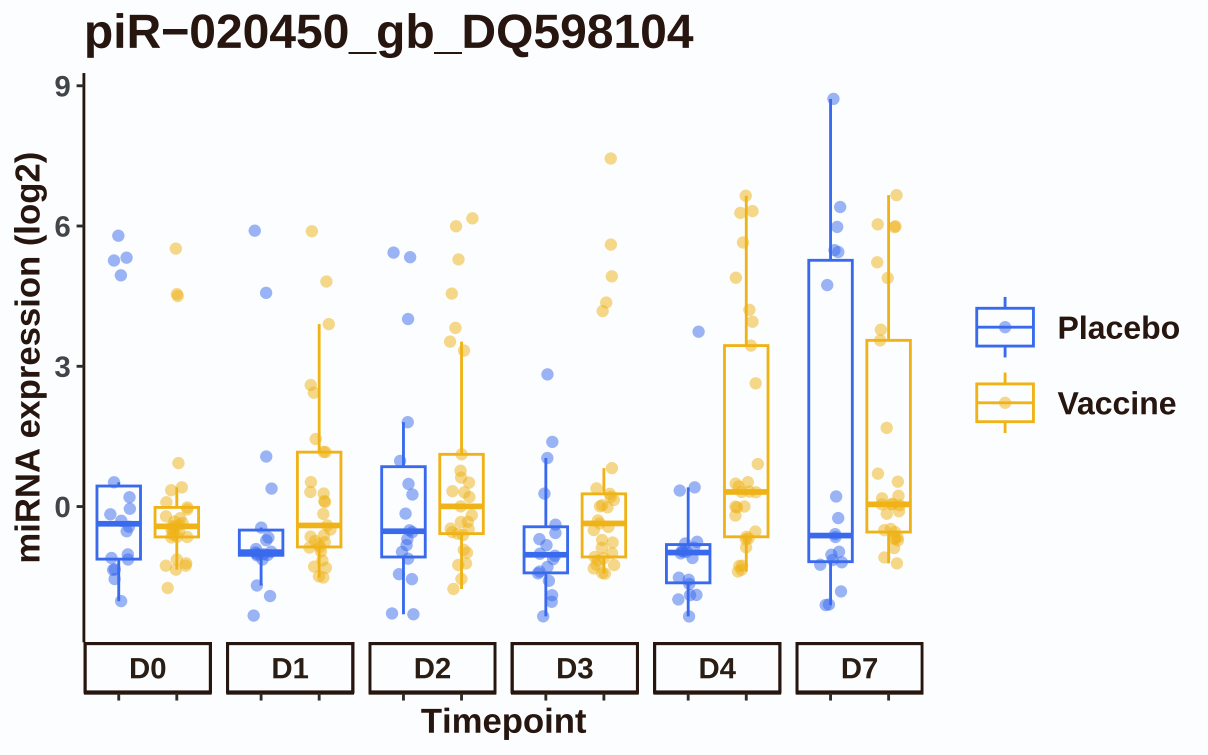

piR−020450_gb_DQ598104
miRNA expression (log2)
9
6
3
0
D0
D1
D2
D3
D4
D7
Timepoint
Placebo
Vaccine

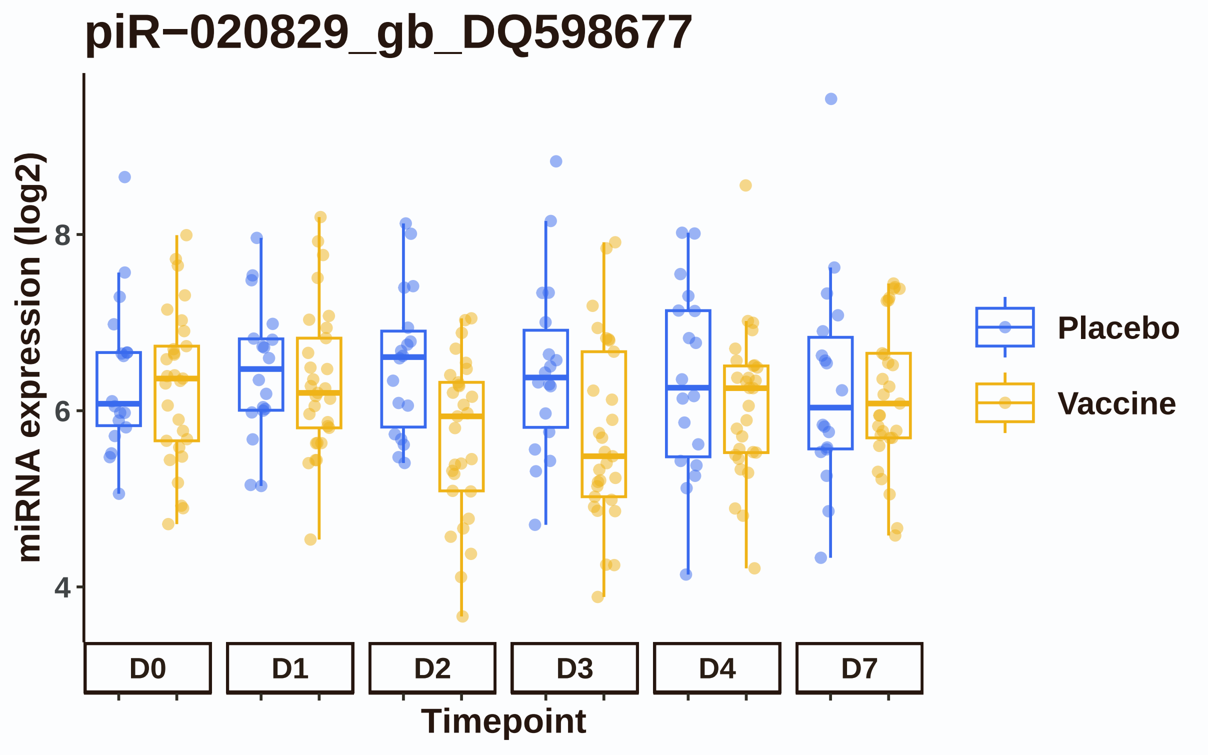

piR−020829_gb_DQ598677
miRNA expression (log2)
8
6
4
D0
D1
D2
D3
D4
D7
Timepoint
Placebo
Vaccine
